# Is mammalian chromosomal evolution driven by regions of genome fragility?

**DOI:** 10.1186/gb-2006-7-12-r115

**Published:** 2006-12-08

**Authors:** Aurora Ruiz-Herrera, Jose Castresana, Terence J Robinson

**Affiliations:** 1 Evolutionary Genomics Group, Department of Botany & Zoology, University of Stellenbosch, Private Bag X1, Matieland 7602, South Africa; 2 Institut de Biologia Molecular de Barcelona, CSIC, Department of Physiology and Molecular Biodiversity, Jordi Girona 18, 08034 Barcelona, Spain

## Abstract

**Background:**

A fundamental question in comparative genomics concerns the identification of mechanisms that underpin chromosomal change. In an attempt to shed light on the dynamics of mammalian genome evolution, we analyzed the distribution of syntenic blocks, evolutionary breakpoint regions, and evolutionary breakpoints taken from public databases available for seven eutherian species (mouse, rat, cattle, dog, pig, cat, and horse) and the chicken, and examined these for correspondence with human fragile sites and tandem repeats.

**Results:**

Our results confirm previous investigations that showed the presence of chromosomal regions in the human genome that have been repeatedly used as illustrated by a high breakpoint accumulation in certain chromosomes and chromosomal bands. We show, however, that there is a striking correspondence between fragile site location, the positions of evolutionary breakpoints, and the distribution of tandem repeats throughout the human genome, which similarly reflect a non-uniform pattern of occurrence.

**Conclusion:**

These observations provide further evidence that certain chromosomal regions in the human genome have been repeatedly used in the evolutionary process. As a consequence, the genome is a composite of fragile regions prone to reorganization that have been conserved in different lineages, and genomic tracts that do not exhibit the same levels of evolutionary plasticity.

## Abstract 2

An analysis of the distribution of evolutionary breakpoints in eight species suggests that certain human chromosomal regions are repeatedly used during the evolutionary process, are associated with fragile sites, and show an enrichment of tandem repeats.

## Background

Evolutionary biologists have long sought to explain the mechanisms of chromosomal evolution in order to better understand the dynamics of mammalian genome organization. Early work in this area led Nadeau and Taylor [1] to propose the 'random breakage model' of genomic evolution, based on linkage maps of human and mouse. Their thesis relied on two assumptions: first, that many chromosomal segments are expected to be conserved among species and, second, that chromosomal rearrangements are randomly distributed within genomes. More than 20 years later, in large part due to molecular cytogenetic studies, large-scale genome sequencing efforts, and new mathematical algorithms developed for whole-genome analysis, the first assumption has been confirmed. However, the second has been questioned by the 'fragile breakage model' [2], which considers that there are regions ('hotspots') throughout the mammalian genome that are prone to breakage and reorganization [3,4].

Most recently, Murphy and colleagues [5] extended these analyses to include homologous synteny block (HSB) data from radiation hybrid maps of dog, cat, pig, and horse. Their findings corroborate the 'hotspot' theory and that some chromosome regions are reused [2] during mammalian chromosomal evolution. Indeed, that about 20% of the evolutionary breakpoint regions reported show reuse [5], particularly among the more rapidly evolving genomes (cattle, dog, and rodents), led us [6] to question whether 'hotspots' identified *in silico *correspond to fragile sites that can be expressed in culture under specific conditions, thus mirroring findings of a correlation between the location of fragile sites and evolutionary breakpoints in primates, including human [7,8]. Our preliminary survey showed that at least 33 of the 88 cytogenetically defined common human fragile sites contain evolutionary breakpoints in at least three of the seven species analyzed by Murphy and colleagues [5].

But what are fragile sites? These are heritable loci located in specific regions of chromosomes that are expressed as gaps or breaks when cells are exposed to specific culture conditions or certain chemical agents such as inhibitors of DNA replication or repair [9]. According to frequency of expression in the human population, and the mechanism of their induction, fragile sites have been classically divided into two groups: common and rare. Common fragile sites are considered part of the chromosome structure since they have been described in different mammalian species (Rodentia [10], Carnivora [11,12], Perissodactyla [13], Cetartiodactyla [14] and Primates [7,15,16]), whereas rare fragile sites are found expressed in a small percentage of the human population [17]. In total, 21 human fragile sites have been molecularly characterized: eight rare fragile sites (FRAXA [18], FRAXE [19], FRAXF [20], FRA10A [21], FRA10B [22], FRA11B [23], FRA16B [24], and FRA16A [25]), and 13 common human fragile sites (FRA1E [26], FRA2G [27], FRA3B [28], FRA4F [29], FRA6E [30], FRA6F [31], FRA7E [32], FRA7G [33], FRA7H [34], FRA9E [35], FRA13A [36], FRA16D [37], and FRAXB [38]). Whereas the expression of rare fragile sites is known to be related to the amplification of specific repeat motifs (CCG repeats and AT-rich regions), no simple repeat sequences have been found to be responsible for the instability observed at common fragile sites. Rather, they appear to have a high A/T content with fragility extending over large regions (from 150 kilobases [kb] to 1 megabase [Mb]) in which the DNA can adopt structures of high flexibility and low stability [39]. Clearly, resolution differences exist between cytogenetically defined fragile sites in human chromosomes and the molecular delimitation of evolutionary breakpoints (themselves fairly gross approximations given that radiation hybrid mapping data for five of the eight species resulted in an average of 1.2 Mb for breakpoint regions [5]). Nonetheless, the fact that fragile sites represent large 'unstable' regions of the genome [39] that in many instances span evolutionary breakpoints [7] is an observation that warrants further detailed analysis.

An intriguing aspect to emerge from comparative genomic studies performed largely on primates and rodents is the finding that breakpoint regions are rich in repetitive elements. In other words, there may be a causal link between the process of chromosome rearrangement, segmental duplications [40-44], and some simple tandem repeats (for instance, the dinucleotide [TA]n [45] and [TCTG]n, [CT]n and [GTCTCT]n [46]). In addition, microsatellites have been implicated in the mechanism underlying the chromosomal instability that characterizes some human fragile sites and constitutional human chromosomal disorders. For example, some human rare and common fragile sites have been found to be particularly rich in A/T minisatellites [39], and certain human chromosomal aberrations have been related to palindromic AT-rich repeats [47,48], underscoring the presence of repetitive elements in regions of chromosomal instability.

With this as the background, we analyze the distribution of 1,638 syntenic blocks, 1,152 evolutionary breakpoint regions, and 2,304 evolutionary breakpoints taken from public databases available for seven eutherian species (mouse, rat, cattle, dog, pig, cat and horse) and chicken, and examine these for correspondence with fragile sites and tandem repeat locations in the human genome. We show that evolutionary breakpoints are not uniformly distributed and that there are certain human chromosomes and chromosomal bands with high breakpoint accumulation. Additionally, there is a striking correspondence between human fragile site location, the positions of evolutionary breakpoints, and the distribution of tandem repeats throughout the human genome.

## Results

### Multispecies alignments

We analyzed homologous regions between the human genome and those of the rat, mouse, cattle, pig, cat, horse, dog, and chicken. By using the HSBs described by Murphy and coworkers [5] and adding data from the human/chicken and human/dog whole-genome sequence assemblies, we were able to identify 1,638 syntenic blocks in the human genome (Additional data file 4). (The dog radiation hybrid genome map data used by Murphy and coworkers [5] was replaced by the dog whole-genome assembly, which is now available.) The analysis of the human/chicken and human/dog whole-genome sequence assemblies revealed a total of 550 syntenic blocks among the three compared species (Additional data file 4). The homologous chromosomal segments of the seven mammals and the chicken were plotted against the 550 band human ideogram (Additional data file 1). We excluded the human chromosome Y from our study of evolutionary breakpoint regions (see Materials and methods, below).

**Figure 1:**
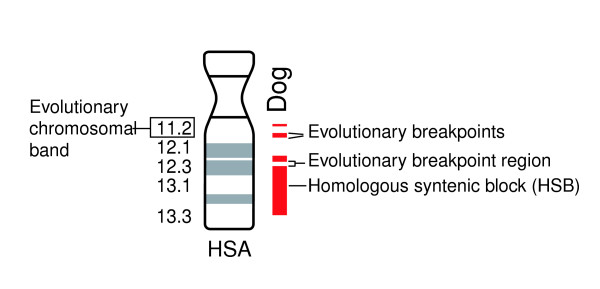
Schematic representation of evolutionary breakpoint regions, evolutionary breakpoints, and evolutionary chromosomal bands. An evolutionary breakpoint region is defined as the interval between two syntenic blocks 4 megabases (Mb) or less in size. This is done in order to avoid problems of low comparative coverage. Evolutionary breakpoints are defined by sequences coordinates in any of the seven mammalian species compared with human plus the chicken, and serve to delimit the start and end of each breakpoint region. Evolutionary chromosomal bands correspond to any band in the human ideogram that contains at least one evolutionary breakpoint in any of the eight species compared with the human genome.

In addition we identified the chromosomal position of 1,152 evolutionary breakpoint regions of 4 Mb or less in size (Additional data file 5) in the human karyotype and their corresponding evolutionary breakpoints (*n *= 2,304; Additional data files 1 and 5). The 2,304 evolutionary breakpoints grouped within 352 evolutionary chromosomal bands, which represents 67.77% of the human genome (2,217.46 Mb of the 3,272.19 Mb of the total human genome, NCBI35; Additional data file 5). See Figure 1 for a schematic representation of evolutionary breakpoint regions, evolutionary breakpoints and evolutionary chromosomal bands, as well as the Materials and methods section (below) for definitions of these terms. Approximately 45% (159 out of 352) of the evolutionary chromosomal bands contain evolutionary breakpoints in three or more of the eight species compared herein (Additional data file 6). These data clearly show that the distribution of the evolutionary breakpoints and breakpoint regions is concentrated in specific bands and/or chromosomes.

An analysis of the distribution of evolutionary breakpoints among the evolutionary chromosomal bands using JMP software (see Materials and methods, below) revealed a mean of six evolutionary breakpoints per evolutionary chromosomal band. Out of the 352 evolutionary chromosomal bands that were identified, 296 contain between one and ten evolutionary breakpoints, whereas 16 human chromosomal bands contain 20 or more evolutionary breakpoints each (10p11.2, 10q11.2, 15q13, 15q24, 15q25, 17p13, 17q24, 1q42.1, 22q11.2, 2p13, 2q14.3, 3p25, 3q21, 4p16, 7q22 and 8p23.1; Additional data file 6). Otherwise stated, 4.21% of the human genome (137.9 Mb of 3,272.19 Mb) accumulates 17.79% of all evolutionary breakpoints (410 of the 2,304 identified). Similarly, not all human chromosomes have been equally affected by the evolutionary process. Human chromosomes 1, 2, 3, 4, 7, 8, 10, 15, 17, and 22 carry most of the evolutionary breakpoints, whereas human chromosomes 14 and 21 are the least frequently involved.

### Distribution of evolutionary breakpoints regions, breakpoints, and fragile sites

Given the distribution of evolutionary breakpoints outlined above, we proceeded to determine whether there is a significant correlation between the position of evolutionary breakpoints and the known location of fragile sites. We mapped all fragile sites (both rare and common) and evolutionary breakpoint regions (regions ≤ 4 Mb; Table 1 and Additional data file 1) to their location on the human ideogram at the 550 band resolution. Our examination reveals that 147 chromosomal bands express fragile sites (both common and rare). A contingency analysis shows that those bands that express fragility (they contain either rare or common fragile sites) have a tendency, although not significantly so (*P *= 0.09), to concentrate evolutionary breakpoints as compared with bands that do not express fragile sites. In fact, we observed 104 bands that contain fragile sites (rare and common) and evolutionary breakpoints, in contrast to the 95.4 bands expected if the distribution were random. A more refined analysis was subsequently conducted in which four categories of chromosomal bands (those that contain common fragile sites, those with rare fragile sites, bands with both common and rare fragile sites, and finally bands with no fragile sites) were examined using contingency analysis. There is a significant tendency (*P *= 0.01) for bands with rare fragile sites to accumulate evolutionary breakpoints (22 of the 24 bands known to express rare fragile sites contain evolutionary breakpoints versus the 15.6 bands expected if the distribution were random). The same tendency does not hold in the case of common fragile sites, where 73 of 111 bands that express common fragile sites contain evolutionary breakpoints (72.2 expected), or bands that contain evolutionary breakpoints but no fragile sites (248 observed versus 256.3 expected).

As stated above, resolution differences exist between cytogenetically defined fragile sites in human chromosomes and the molecular delimitation of evolutionary breakpoints. That differences in resolution may confound the association between them is clearly of concern. However, of the 12 autosomal common fragile sites that have been characterized at the molecular level (Additional data file 8), six (FRA4F, FRA6E, FRA7E, FRA7G, FRA7H, and FRA9E) were shown to span evolutionary breakpoints in at least one of the species analyzed with an additional two fragile sites (FRA3B and FRA16D) located within 1 Mb of evolutionary breakpoints (Additional data file 8). Importantly, of the four autosomal common fragile sites with the highest expression frequencies (FRA3B [28], FRA6E [30], FRA7H [34], and FRA16D [37]), two (FRA6E and FRA7H) are localized within evolutionary breakpoints, and two (FRA3B and FRA16D) lie within 1 Mb of breakpoint boundaries. With respect to the eight cloned rare fragile sites [18-25], three (FRA10A, FRA16A, and FRA16B) are located in bands that contain evolutionary breakpoints in at least one of the species analyzed by us.

### Distribution of tandem repeats

The distribution of tandem repeats in human chromosomes was analyzed using 250,000 bp search windows in order to determine whether there is any correspondence between tandem repeats, fragile sites (both rare and common), and the location of evolutionary breakpoints (Additional data files 2 and 8). The tandem repeats range from microsatellites (unit size 1 bp to 6 bp) to different types of minisatellites (from 7 bp to 300 bp). We identified a high concentration of tandem repeats in the telomeres and the pericentromeric regions of each chromosome (Additional data file 2), mirroring earlier findings (for instance, see Näslund and coworkers [49]). The distribution of tandem repeats (1 to 300 bp) along human chromosomes showed that on average 3,738.56 bp of the 250,000 bp of genomic sequence contained in each window comprised tandem repeats (about 1.5%). Chromosome 19 is exceptional for the high number of repeats found along its length [50], which is almost double (8,377.27 bp) the average for the whole genome (Table 2 and Additional data file 3). Additionally, chromosome 19 has been shown to be exceptional in many other genomic features, most of which (including the high number of repeats) may be due to the extremely high GC content of this chromosome [51,52].

#### Tandem repeats and evolutionary chromosomal bands

When analyzing the human genome in its entirety, but excluding the centromeric and telomeric regions from the analysis, evolutionary chromosomal bands (E bands) tend to contain significantly more (*P *< 0.05) tandem repeats than chromosomal bands not implicated in evolutionary change (B bands; Table 2). It is noteworthy that in the case of human chromosomes 3, 15, 17, 18, and 21, E bands contain significantly more tandem repeats than do the B bands (*P *< 0.05), whereas the converse holds for human chromosomes 8 and 16. In all other instances no statistically supported differences were noted. Elimination of chromosome 19 from the analysis, with its singularly high repeat content, reduces the difference between E bands and B bands but not significantly so. In addition, we detected 256 human chromosomal bands that contain regions with more than 6,000 bp of tandem repeats in the 250,000 bp of genomic sequence contained in each window. Of these high-density repeat loci, 76.95% (197 of 256) contain evolutionary breakpoints.

#### Tandem repeats and fragile sites

Overall, chromosomal bands that express fragile sites (rare and common combined) contain significantly more tandem repeats (*P *< 0.05) than do bands that do not (Table 2 and Additional data file 9). There are, however, differences evident among chromosomes. In the case of human chromosomes 1, 5, 7, 8, 11, 12, and 22, chromosomal bands that express fragile sites contain more tandem repeats than do bands that do not show fragility (*P *< 0.05). The converse holds for chromosomes 10, 14, 17, and 20, where regions of fragility are not characterized by elevated tandem repeat levels. In the remaining human chromosomes (2, 3, 4, 6, 9, 13, 15, 16, 18, and 19), there is no statistical relationship between those bands that express fragile sites and have high numbers of tandem repeats, and bands that do not (Table 2). Moreover, the statistically significant differences detailed above hold irrespective of whether chromosome 19 is omitted from the analysis or not. Interestingly, 62.6% (92 out of 147; Table 1) of the human bands that contain human fragile sites are localized in regions that contain high densities of repeats (for instance, regions containing >6,000 bp of tandem repeats in the 250,000 bp of genomic sequence contained in each window; see above). No fragile sites have been described in the literature for human chromosome 21.

We examined the repeat content of the four categories of chromosomal bands (those that express common fragile sites, bands with rare fragile sites, bands with both common and rare fragile sites, and finally bands that do not contain fragile sites; Additional data file 9). Those containing rare fragile sites were shown to have significantly (*P *< 0.05) greater numbers of tandem repeats (average of 4,852.53 bp per 250,000 bp of genomic sequence contained in each window) than any other category (3,714.86 bp per 250,000 bp of genomic sequence contained in each window in the case of common fragile sites, the next most frequent category).

## Discussion

### Evolutionary breakpoints

Evolutionary breakpoints can be defined by levels of resolution [53]. The holistic perspective of evolutionary breakpoints has traditionally been underpinned by molecular cytogenetic studies that assign regions of chromosomal homology to species of the same or different orders of mammals at the chromosomal band level. Investigations using comparative chromosome painting (ZOO-fluorescence *in situ *hybridization [ZOO-FISH]) involving more than 80 different species from almost all of the recognized eutherian orders have defined regions of the human genome that are implicated in chromosomal evolution (for review, see Froenicke [54]). The integration of cross-species chromosome painting data published from 30 nonprimate species [54], and even greater numbers of primate species [8], clearly demonstrate that evolutionary breakpoints are not uniformly distributed along the length human chromosomes, and in some cases they are conserved during chromosome evolution.

The use of whole-genome comparisons (the reductionist view) allows for the delimitation of evolutionary breakpoints at a finer level of resolution than can be obtained by chromosome painting. By analyzing published data [5], and adding complementary information from the human/chicken and human/dog whole-genome sequence assemblies, we were able to identify 1,152 evolutionary breakpoint regions throughout the human genome at a resolution of 4 Mb or less, which contain 2,304 evolutionary breakpoints. Plotting the evolutionary breakpoints included in our data onto the 550 chromosomal band human ideogram provided a means of combining the cytogenetic and the sequence comparisons. This identified 352 human chromosomal bands that contain evolutionary breakpoints and showed that the distribution of evolutionary breakpoints is not uniform in the human genome. Quite clearly, there are evolutionary 'hot spots', defined by chromosomal bands, which are coincidental with genomic reorganization characterizing different lineages during the evolutionary process (breakpoint reuse [5]).

### Evolutionary implications of fragile sites

Although the exact number of fragile sites described in the human genome is a matter of interpretation, a recent revision lists 119 fragile sites, 88 of which are defined as common and 31 as rare [39]. Our data show that human chromosomal bands that express fragile sites (both common and rare combined) have a tendency to contain evolutionary breakpoints (Table 1), although the association is statistically supported only in the case of rare fragile sites. This association suggests an important role for fragile sites in genome reorganization, most likely by functioning as regions of chromosomal instability.

Although the mechanisms underlying the breakage at common fragile sites are still poorly understood, rare fragile sites are associated with the amplification of repeat motifs (CCG repeats and AT-rich regions). The molecular characterization of 13 common fragile sites has revealed that there are no simple repeat sequences responsible for their instability (for review, see Schwartz and coworkers [39] and Glover [55]). Rather they are enriched in A/T content, have the potential to form secondary structures, and contain clusters of flexible sequences (flexibility clusters). These are all features that may affect DNA replication and chromatin condensation, suggesting a common basis for fragility (presence of repeat sequences) that would characterize all fragile sites (both common and rare).

Previously, evolutionary studies involving fragile sites have attempted to address two important questions. First, because fragile sites are considered part of the chromosome structure, are the characteristics underlying their susceptibility to breakage conserved during evolution? Also, can fragile sites be considered 'targets' for evolutionary reorganization? In terms of the first question recent studies have shown that some human common fragile sites have been conserved in homologous regions in mouse and some primate species [29,56,57], suggesting that the characteristics governing a chromatid's susceptibility to breakage are conserved during evolution. The high degree of correspondence between the location of fragile sites and evolutionary breakpoints shown by our study has a bearing on the second question posed above, namely whether fragile sites are 'targets' for evolutionary reorganization. Comparative cytogenetic studies performed in primate families such as Hominidae, Cebidae, and Cercopithecidae [7,16,58-60] revealed that a high proportion of chromosomal bands implicated in evolutionary reorganization, centromeric shifts, and delimiting heterochromatic regions also contain fragile sites in the human genome. By increasing the number of species analyzed (mouse, rat, cattle, dog, pig, cat, horse, and chicken), as well as improving the resolution of evolutionary breakpoints using whole-genome comparisons, we have been able to draw more precise conclusions on the distribution of evolutionary breakpoints and their correspondence to human bands that are known to contain fragile sites. Our data show that fragile sites appear to be conserved as 'fragile chromosomal bands', in which evolutionary breakpoints accumulate in much the same way that human fragile sites may be considered to signal regions of chromosomal instability observed in cancer cells [61].

### Repetitive DNA, fragile sites and chromosomal evolution

Given the 'hot spot' theory, one may question whether repetitive elements are driving chromosomal evolution by triggering reorganization in these regions (for instance, see the reports by Armengol [42] and Cáceres [62] and their coworkers) or, alternatively, that the repeats accumulate preferentially in these regions following reorganization. That our study shows that rare fragile sites in particular have a highly significant association (*P *= 0.01) with both evolutionary breakpoints and tandem repeats has important implications for the role of this particular type of fragile site in chromosomal instability, and hence genome evolution. The molecular characterization of chromosomal regions implicated in evolutionary breakpoints in human, mouse, and primate genomes has similarly shown that large-scale reorganization tends to occur at, or close to, regions rich in segmental duplications and some type of simple tandem repeat (for example, the dinucleotide [TA]n) [41,63-65].

The analysis of the distribution of tandem repeats in human chromosomes and their spatial relationship to evolutionary breakpoints presented here highlights two important points. First, it emphasizes the high concentration of base pair repeats found at the telomeres and the pericentromeric areas (which is in agreement with previous reports on the distribution of duplicated regions; see Murphy and coworkers [5]), and the distribution of polymorphic minisatellites [49] throughout the human genome. The second, possibly more remarkable finding is the concentration of tandem repeats at evolutionary chromosomal bands. Although this is by no means ubiquitous, the correspondence is typified by human chromosome 3 (Table 2 and Additional data file 1). Bands with the greatest number of tandem repeats in this chromosome (3p25, 3p21.3, 3p12, 3q13.1, 3q21, and 3q29) are also chromosomal regions that have been implicated in evolutionary rearrangements. It is noteworthy that the chromosomal bands 3p25, 3p21, 3p12, and 3q21 have previously been identified as breakpoints in primate evolution [66], and that the evolutionary breakpoints at 3p25.1, 3p12.3, and 3q21.3 are associated with duplications in hominid evolution [67-69].

**Figure 2:**
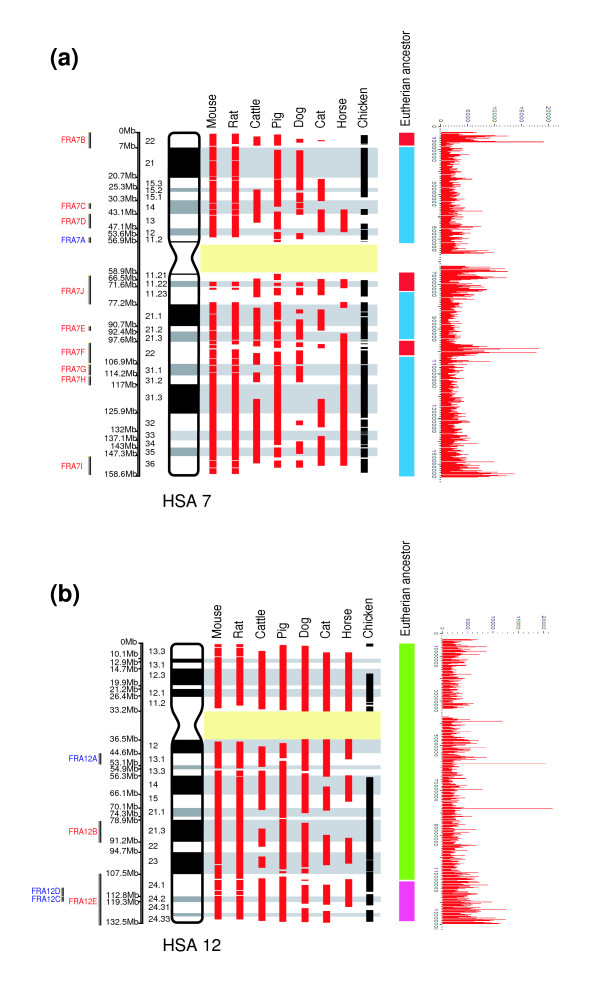
Multispecies alignments to human chromosomes. Shown are the multispecies alignments of human chromosomes **(a) **7 and **(b) **12. Red bars represent the homology synteny blocks (HSBs) in the seven eutherian species that were compared in our analyses. Black bars represent the regions of homology to the chicken genome [74]. Segments 7a (red), 7b (blue), 12a (green), and 12b (pink) are portions of human chromosomes comprising the ancestral eutherian karyotype [8,22]. The location of human fragile sites (FRA) is given to the left of the ideogram of each human chromosome: red for common fragile sites and blue for rare fragile sites. The distribution of tandem repeats along these human chromosomes is shown on the extreme right. The high concentrations of tandem repeats in the pericentromeric and telomeric areas of each chromosome have been the omitted to provide clearer resolution of the distribution of repeats elsewhere on the chromosomes (see text for details).

In particular, human chromosome 7 (Figure 2a) is interesting both from the evolutionary as well as clinical perspective. Our analysis shows that there are six bands on this chromosome that contain the greatest concentration of tandem repeats in the human genome: 7p22, 7p13, 7p11, 7q11, 7q22, and 7q36. All six bands incorporate fragile sites (FRA7B, FRA7D, FRA7A, FRA7J, FRA7F, and FRA7I) and all but one of them (7p13) correspond to regions where evolutionary breakpoints tend to concentrate, as indicated by comparisons of the human genome with those of mouse, rat, cattle, pig, dog, cat, chicken (present study), and different primate species [8]. Three of these chromosomal bands (7p22, 7q11, and 7q22) appear to be the boundaries for mammalian ancestral chromosomes 7a and 7b (Figure 2a) and have been implicated in almost all mammalian species studied to date by comparative chromosome painting using human painting probes [8,54]. A recent study of the evolutionary history of human chromosome 7 [70] demonstrated that this chromosome may be derived from the orangutan homolog by two inversions (one paracentric and another pericentric) that involved three chromosomal breakpoints that map to 7p22.1, 7q11.23, and 7q22.1 in human (one of these, 7q22.1, is common to both rearrangements). All three bands have the greatest number of tandem repeats (present study) and are particularly rich in segmental duplications [40]. Moreover, they are considered 'hot spots' for human diseases such as the Williams-Beuren syndrome [71,72] and leukemias [73].

Other notable associations between tandem repeats, fragile site location, and evolutionary breakpoints include the greatest concentration of tandem repeats found in the human genome - those in bands 12q13.1 and 12q24. The band 12q13.1 contains one fragile site (FRA12A) and two evolutionary breakpoints, whereas 12q24 contains three fragile sites (FRA12C, FRA12D, and FRA12E) and seven evolutionary breakpoints (Figure 2b). Human chromosome 12 is considered to be the result of the fusion of two ancestral chromosomal segments 12a and 12b (Figure 2b) that are thought to have occurred in the Simiiformes (Catarrhini and Platyrrhini) ancestor. Chromosomal band 12q24 forms the boundary of these segments [8], once again highlighting a chromosomal region that is characterized both by its fragility and involvement in evolutionary change.

## Conclusion

Our results provide clear evidence of the existence of chromosomal regions in the human genome that have been repeatedly used in the evolutionary process, thus confirming and extending earlier observations [2,5,8]. As a consequence, the human genome can be considered a mosaic comprising regions of fragility that are prone to reorganization that have been conserved in different lineages during the evolutionary process, and regions that do not exhibit the same levels of evolutionary plasticity. Although we cannot unequivocally suggest a mechanistic role for tandem repeats and fragile sites in sculpting modern genomes, our data will serve to focus further detailed investigations on this fundamental aspect of genome evolution.

## Materials and methods

### Whole-genome comparisons and breakpoints analysis

The Ensembl genome browser of Sanger Center and EMBL [74] as well as published data [5] were used as sources for determining homologies between the human genome and those of the mouse, rat, cattle, pig, dog, cat, horse, and chicken. We used the sequence coordinates described by Murphy and coworkers [5] to delimit homologous synteny blocks (HSBs), where the data from cattle, pig, cat, and horse are based on RH maps; the homologous regions between human, rat, and mouse are based on whole-genome assemblies. To determine syntenic regions between the human genome (NCBI Build 35) and that of the dog and chicken, we used the completed human/chicken (WASHUC 1) and human/dog (CanFam 1.0) whole-genome sequence assemblies available from the Ensembl genome browser. In the case of the dog and chicken we analyzed homologous syntenic blocks that varied in size between 0.1 Mb and 84 Mb (Additional data file 4), according to the Ensembl genome browser.

For all species analyzed, we follow Murphy and coworkers [5] in viewing an 'evolutionary breakpoint region' as the interval between two syntenic blocks. As did those authors, we use evolutionary breakpoint regions that are 4 Mb in size or less in order to avoid problems of low comparative coverage. 'Evolutionary breakpoints' are defined by sequence coordinates in any of the seven mammalian species compared with human plus the chicken. They serve to delimit the start and end of each breakpoint region. Likewise, the limits of each chromosomal band in the human karyotype can be defined by sequence coordinates using the Ensembl database [74]. Following this procedure, evolutionary breakpoints of each homologous segment were mapped to the human ideogram at the 550 band resolution, allowing us to identify 'evolutionary chromosomal bands' (E bands), which are defined as any band in the human ideogram that contains at least one evolutionary breakpoint in any of the eight species compared with the human genome (Figure 1). We used the JMP software (version 5.1.2; SAS Institute Inc., Cary, NC, USA) to investigate the distribution of evolutionary breakpoints.

### Fragile site analysis

The data reported by Schwartz and coworkers [39] were used as reference for the location, classification, and number of fragile sites described in the human genome. Human fragile sites may be classified into two groups based on frequency of occurrence and mechanisms of expression, and are generally referred to as either common or rare fragile sites [17]. In this investigation we considered a total of 119 fragile sites [39], of which 88 are defined as common and 31 as rare fragile sites (Additional data file 7). These were mapped to specific chromosomal bands on the human ideogram at the 550 band resolution (Table 1). The evolutionary chromosome breakpoint boundaries, each identified by human reference coordinates (see above), were similarly treated in order to determine whether these fell within a specific chromosomal band region that is known to express fragility. It is important to note that in some cases a chromosomal band described as containing a fragile site in the literature can, at higher resolution (for example, the 550 band ideogram), be shown to comprise several sub-bands. For example, the common fragile site FRAJ is mapped to 7q11, which corresponds to four sub-bands in the 550 band ideogram (7q11.1, 7q11.21, 7q11.22, and 7q11.23).

We defined the chromosomal location of 12 autosomal common fragile sites that have been characterized at the molecular level by the position provided by the Ensembl [74] and NIH databases [75] for the molecular markers and/or the BAC clones described in the original papers (Additional data file 8). These fragile sites were examined to determine whether any evolutionary breakpoint spanned these regions in at least one of the species compared herein.

### Tandem repeat analysis

We analyzed the distribution of tandem repeats in the human genome sequence (NCBI Build 35) using the 'Tandem Repeats Finder' (TRF) algorithm (version 3.21 [76]) in all human chromosomes (HSA) except HSA X and HSA Y. The complete sequences of each chromosome were scanned for tandem repeats using the program TRF with the parameters established by default (+2 -7 -7 0.80 0.10 50 500).

We scrutinized each chromosome's complete sequence using moving non-overlapping windows of 0.250 Mb in order to analyze the density and distribution of tandem repeats in the human genome. Given the high incidence of repeats at the telomeres/subtelomeric and the centromeric/pericentromeric areas [49] (confirmed by our study; Additional data file 9), we excluded a 3 Mb section at each of these localities, which are referred to herein as the T (telomeric) and C (centromeric) regions. A further classification involves chromosomal bands that contain evolutionary breakpoints in at least one of the eight species compared with the human genome (E bands); all remaining bands were designated as B bands (for example, non-evolutionary chromosomal bands). Additionally, the presence/absence of a fragile site (rare or common) was recorded for each chromosomal band based on their published location [39], as defined in the human ideogram at the 550 band resolution (Additional data file 9). Tukey-Kramer tests were used (JMP package version 5.1.2; SAS Institute Inc.) to evaluate whether tandem repeats concentrate significantly (*P *≤ 0.05) in evolutionary chromosomal bands (E bands) and/or fragile sites (FS bands). In both cases, the centromeric and telomeric regions were excluded before statistical analysis because they had much higher repeat values overall.

## Additional data files

The following additional data are available with the online version of this paper. Additional data file 1 is a figure showing the multispecies alignments of all human chromosomes. Additional data file 2 is a figure showing the distribution of base pair tandem repeats along all human chromosomes represented as windows of 250,000 bp each. Additional data file 3 is a figure showing base pairs implicated in tandem repeats per chromosome. Additional data file 4 is a table listing all of the homologous syntenic blocks (HSB) detected. Additional data file 5 is a table listing evolutionary breakpoint regions (EBR) less than 4 Mb and their chromosomal positions in the human genome. Additional data file 6 is a table listing the evolutionary chromosomal bands detected. Additional data file 7 is a table listing all human fragile sites described in the literature. Additional data file 8 is a table listing common human fragile sites that have been cloned and analyzed at the molecular level. Additional data file 9 is a table showing the human genome divided into windows of 0.250 Mb.

## Supplementary Material

Additional data file 1 Multispecies alignments to all human chromosomes. Click here for file

Additional data file 2 Distribution of base pair tandem repeats along all human chromosomes represented as windows of 250,000 bp each. Click here for file

Additional data file 3 Base pairs implicated in tandem repeats per chromosome. Click here for file

Additional data file 4 List of all of the homologous syntenic blocks (HSB) detected. Click here for file

Additional data file 5 List of evolutionary breakpoint regions (EBR) less than 4 Mb and their chromosomal positions in the human genome. Click here for file

Additional data file 6 List of evolutionary chromosomal bands detected. Click here for file

Additional data file 7 List of all human fragile sites described in the literature. Click here for file

Additional data file 8 List of common human fragile sites that have been cloned and analyzed at the molecular level. Click here for file

Additional data file 9 Human genome divided into windows of 0.250 Mb. Click here for file

## Figures and Tables

**Table 1:** The human ideogram at the 550 band resolution showing the location of fragile sites and evolutionary breakpoints

Chromosomal band	Type fs	EB	Chromosomal band	Type fs	EB
10p11.1	No fs	No EB	22q13.3^b^	r-fs	EB
10p11.2	No fs	EB	2p11.1	No fs	No EB
10p12.1	No fs	EB	2p11.2^b^	r-fs	EB
10p12.2	No fs	No EB	2p12	No fs	EB
10p12.3	No fs	EB	2p13^b^	c-fs	EB
10p13	No fs	EB	2p14	No fs	EB
10p14	No fs	EB	2p15	No fs	EB
10p15	No fs	EB	2p16	c-fs	EB
10q11.1	No fs	No EB	2p21	No fs	EB
10q11.2^a^	c-fs	EB	2p22	No fs	EB
10q21.1^a^	c-fs	EB	2p23	No fs	EB
10q21.2^a^	c-fs	EB	2p24	c-fs	No EB
10q21.3^a^	c-fs	EB	2p25.1	No fs	No EB
10q22.1^a^	c-fs	EB	2p25.2	No fs	No EB
10q22.2	No fs	EB	2p25.3	No fs	EB
10q22.3	No fs	EB	2q11.1	No fs	No EB
10q23.1	No fs	EB	2q11.2^a^	r-fs	EB
10q23.2	No fs	EB	2q12	No fs	EB
10q23.3^a^	r-fs	EB	2q13^a^	r-fs	EB
10q24.1	No fs	EB	2q14.1	No fs	EB
10q24.2	No fs	EB	2q14.2	No fs	EB
10q24.3	No fs	EB	2q14.3	No fs	EB
10q25.1	No fs	EB	2q21.1	No fs	EB
10q25.2	c-fs and r-fs	No EB	2q21.2	No fs	EB
10q25.3	No fs	No EB	2q21.3	c-fs	EB
10q26.1^a^	c-fs	EB	2q22	r-fs	EB
10q26.2	No fs	EB	2q23	No fs	EB
10q26.3	No fs	No EB	2q24.1	No fs	No EB
11p11.1	No fs	EB	2q24.2	No fs	EB
11p11.21	No fs	No EB	2q24.3	No fs	No EB
11p11.22	No fs	EB	2q31^a^	c-fs	No EB
11p12	No fs	No EB	2q32.1^a^	c-fs	EB
11p13	c-fs	EB	2q32.2	No fs	EB
11p14	c-fs	EB	2q32.3	No fs	EB
11p15.1^b^	c-fs and r-fs	EB	2q33^a^	c-fs	EB
11p15.2	No fs	EB	2q34	No fs	EB
11p15.3	No fs	No EB	2q35	No fs	EB
11p15.4	No fs	EB	2q36	No fs	EB
11p15.5	No fs	EB	2q37.1	No fs	EB
11q11	No fs	No EB	2q37.2	No fs	EB
11q12	No fs	EB	2q37.3^b^	c-fs	EB
11q13.1^b^	c-fs	EB	3p11	No fs	No EB
11q13.2^b^	c-fs	No EB	3p12	No fs	EB
11q13.3^b^	c-fs and r-fs	EB	3p13	No fs	No EB
11q13.4^b^	c-fs	EB	3p14.1	No fs	EB
11q13.5^b^	c-fs	EB	3p14.2	c-fs	No EB
11q14.1	No fs	EB	3p14.3	No fs	EB
11q14.2^a^	c-fs	No EB	3p21.1	No fs	No EB
11q14.3	No fs	EB	3p21.2	No fs	EB
11q21	No fs	No EB	3p21.3	No fs	EB
11q22.1	No fs	EB	3p22	No fs	EB
11q22.2	No fs	EB	3p23	No fs	EB
11q22.3	No fs	EB	3p24	c-fs	EB
11q23.3^a^	c-fs and r-fs	No EB	3p25	No fs	EB
11q23.2	No fs	No EB	3p26	No fs	No EB
11q23.1	No fs	EB	3q11.1	No fs	No EB
11q24	No fs	EB	3q11.2	No fs	EB
11q25	No fs	No EB	3q12	No fs	EB
12p11.1	No fs	No EB	3q13.1	No fs	No EB
12p11.2	No fs	EB	3q13.2	No fs	EB
12p12.1	No fs	No EB	3q13.3	No fs	EB
12p12.2	No fs	No EB	3q21	No fs	EB
12p12.3	No fs	No EB	3q22	No fs	EB
12p13.3	No fs	EB	3q23	No fs	EB
12p13.2	No fs	No EB	3q24	No fs	EB
12p13.1	No fs	No EB	3q25.1	c-fs	No EB
12q11	No fs	No EB	3q25.2	c-fs	No EB
12q12	No fs	No EB	3q25.3	c-fs	EB
12q13.1^a^	r-fs	EB	3q26.1	No fs	EB
12q13.2	No fs	EB	3q26.2	No fs	EB
12q13.3	No fs	EB	3q26.3	No fs	EB
12q14	No fs	EB	3q27^a^	c-fs	EB
12q15	No fs	No EB	3q28	No fs	No EB
12q21.1	No fs	EB	3q29	No fs	EB
12q21.2	No fs	EB	4p11	No fs	No EB
12q21.3^a^	c-fs	No EB	4p12	No fs	EB
12q22	No fs	EB	4p13	No fs	EB
12q23	No fs	EB	4p14	No fs	No EB
12q24.1^b^	c-fs and r-fs	EB	4p15.1^b^	c-fs	No EB
12q24.2^b^	c-fs and r-fs	No EB	4p15.2^b^	c-fs	No EB
12q24.31^b^	c-fs	EB	4p15.3^b^	c-fs	EB
12q24.32^b^	c-fs	No EB	4p16^a^	c-fs	EB
12q24.33^b^	c-fs	No EB	4q11	No fs	No EB
13p13	No fs	No EB	4q12	c-fs	EB
13p12	No fs	No EB	4q13.1	No fs	No EB
13p11.2	No fs	No EB	4q13.2	No fs	No EB
13p11.1	No fs	No EB	4q13.3	No fs	EB
13q11	No fs	No EB	4q21.1	No fs	EB
13q12.1	No fs	EB	4q21.2	No fs	EB
13q12.2	No fs	No EB	4q21.3	No fs	No EB
13q12.3	No fs	No EB	4q22	c-fs	EB
13q13	c-fs	EB	4q23	No fs	No EB
13q14.1	No fs	EB	4q24	No fs	No EB
13q14.2	No fs	EB	4q25	No fs	No EB
13q14.3	No fs	EB	4q26	No fs	EB
13q21.1^b^	c-fs	No EB	4q27	No fs	EB
13q21.2^b^	c-fs	No EB	4q28	No fs	EB
13q21.3^b^	c-fs	No EB	4q31.1	c-fs	EB
13q22	No fs	No EB	4q31.2	No fs	EB
13q31	No fs	EB	4q31.3	No fs	EB
13q32	c-fs	No EB	4q32	No fs	EB
13q33	No fs	EB	4q33	No fs	EB
13q34	No fs	No EB	4q34	No fs	EB
14p13	No fs	No EB	4q35	No fs	EB
14p12	No fs	No EB	5p11	No fs	No EB
14p11.2	No fs	No EB	5p12	No fs	EB
14p11.1	No fs	No EB	5p13.1^a^	c-fs	No EB
14q11.1	No fs	No EB	5p13.2^a^	c-fs	No EB
14q11.2	No fs	EB	5p13.3^a^	c-fs	No EB
14q12	No fs	No EB	5p14^b^	c-fs	No EB
14q13	No fs	EB	5p15.1	No fs	EB
14q21	No fs	EB	5p15.2	No fs	EB
14q22	No fs	EB	5p15.3	No fs	EB
14q23^a^	c-fs	EB	5q11.1	No fs	No EB
14q24.1	c-fs	No EB	5q11.2	No fs	EB
14q24.2	No fs	No EB	5q12	No fs	EB
14q24.3	No fs	No EB	5q13.1	No fs	EB
14q31	No fs	No EB	5q13.2	No fs	EB
14q32.1	No fs	No EB	5q13.3	No fs	EB
14q32.2	No fs	No EB	5q14	No fs	EB
14q32.3	No fs	EB	5q15	c-fs	EB
15p13	No fs	No EB	5q21^b^	c-fs	EB
15p12	No fs	No EB	5q22	No fs	EB
15p11.2	No fs	No EB	5q23.1	No fs	No EB
15p11.1	No fs	No EB	5q23.2	No fs	No EB
15q11.1	No fs	No EB	5q23.3	No fs	EB
15q11.2	No fs	EB	5q31.1^b^	c-fs	EB
15q12	No fs	EB	5q31.2	No fs	No EB
15q13	No fs	EB	5q31.3	No fs	No EB
15q14	No fs	EB	5q32	No fs	EB
15q15	No fs	EB	5q33.1	No fs	EB
15q21.1	No fs	EB	5q33.2	No fs	EB
15q21.2	No fs	No EB	5q33.3	No fs	EB
15q21.3	No fs	No EB	5q34	No fs	EB
15q22.1^a^	c-fs	No EB	5q35.1^b^	r-fs	EB
15q22.2^a^	c-fs	No EB	5q35.2^b^	r-fs	EB
15q22.3^a^	c-fs	EB	5q35.3^b^	r-fs	EB
15q23	No fs	EB	6p11.1	No fs	No EB
15q24	No fs	EB	6p11.2	No fs	EB
15q25	No fs	EB	6p12	No fs	EB
15q26.1	No fs	EB	6p21.1	No fs	EB
15q26.2	No fs	No EB	6p21.2	No fs	EB
15q26.3	No fs	EB	6p21.3	No fs	EB
16p11.1	No fs	No EB	6p22.1	No fs	EB
16p11.2	No fs	EB	6p22.2	c-fs	EB
16p12^a^	r-fs	EB	6p22.3	No fs	EB
16p13.1^b^	r-fs	EB	6p23^a^	r-fs	No EB
16p13.2	No fs	No EB	6p24	No fs	No EB
16p13.3	No fs	EB	6p25^a^	c-fs	No EB
16q11.1	No fs	No EB	6q11	No fs	No EB
16q11.2	No fs	No EB	6q12	No fs	EB
16q12.1	No fs	EB	6q13^a^	c-fs	EB
16q12.2	No fs	EB	6q14	No fs	EB
16q13	No fs	EB	6q15	c-fs	EB
16q21	No fs	EB	6q16.1	No fs	EB
16q22^a^	c-fs and r-fs	EB	6q16.2	No fs	No EB
16q23^a^	c-fs	EB	6q16.3	No fs	EB
16q24	No fs	No EB	6q21^a^	c-fs	No EB
17p11.1	No fs	No EB	6q22.1	No fs	EB
17p11.2	No fs	EB	6q22.2	No fs	No EB
17p12	r-fs	EB	6q22.3	No fs	EB
17p13	No fs	EB	6q23.1	No fs	No EB
17q11.1	No fs	No EB	6q23.2	No fs	EB
17q11.2	No fs	EB	6q23.3	No fs	No EB
17q12	No fs	EB	6q24	No fs	EB
17q21.1	No fs	EB	6q25.1	No fs	EB
17q21.2	No fs	EB	6q25.2	No fs	EB
17q21.3	No fs	EB	6q25.3	No fs	EB
17q22	No fs	No EB	6q26^b^	c-fs	No EB
17q23	c-fs	EB	6q27	No fs	EB
17q24	No fs	EB	7p11.1	No fs	No EB
17q25	No fs	EB	7p11.2^a^	r-fs	EB
18p11.1	No fs	No EB	7p12	No fs	EB
18p11.2	No fs	EB	7p13^a^	c-fs	EB
18p11.32	No fs	EB	7p14	c-fs	EB
18p11.31	No fs	No EB	7p15.1	No fs	No EB
18q11.1	No fs	No EB	7p15.2	No fs	EB
18q11.2	No fs	EB	7p15.3	No fs	EB
18q12.1	No fs	No EB	7p21	No fs	EB
18q12.2	c-fs	EB	7p22^b^	c-fs	EB
18q12.3	No fs	EB	7q11.1^b^	c-fs	No EB
18q21.1	No fs	EB	7q11.21^b^	c-fs	No EB
18q21.2	No fs	No EB	7q11.22^b^	c-fs	EB
18q21.3^a^	c-fs	EB	7q11.23^b^	c-fs	EB
18q22	r-fs	EB	7q21.1	No fs	EB
18q23	No fs	EB	7q21.2	c-fs	EB
19p11	No fs	No EB	7q21.3	No fs	EB
19p12	No fs	EB	7q22^b^	c-fs	EB
19p13.1^b^	r-fs	EB	7q31.1	No fs	EB
19p13.2^b^	r-fs	EB	7q31.2	c-fs	No EB
19p13.3^b^	r-fs	EB	7q31.3	No fs	EB
19q11	No fs	No EB	7q32	c-fs	EB
19q12	No fs	EB	7q33	No fs	EB
19q13.1	c-fs	EB	7q34	No fs	EB
19q13.2	c-fs	EB	7q35	No fs	EB
19q13.3	c-fs	EB	7q36^b^	c-fs	EB
19q13.4	c-fs	EB	8p11.1	No fs	No EB
1p11	No fs	No EB	8p11.2	No fs	EB
1p12	No fs	No EB	8p12	No fs	EB
1p13.1	No fs	EB	8p21.1	No fs	EB
1p13.2	No fs	EB	8p21.2	No fs	EB
1p13.3	No fs	EB	8p21.3	No fs	EB
1p21	c-fs and r-fs	EB	8p22	No fs	EB
1p22.1	c-fs	EB	8p23.1	No fs	EB
1p22.2	c-fs	EB	8p23.2	No fs	No EB
1p22.3	c-fs	EB	8p23.3	No fs	EB
1p31.1	c-fs	EB	8q11.1	No fs	No EB
1p31.2	c-fs	EB	8q11.21	No fs	EB
1p31.3	c-fs	No EB	8q11.23	No fs	EB
1p32.3^b^	c-fs	EB	8q12	No fs	EB
1p32.1^b^	c-fs	No EB	8q13	No fs	EB
1p32.2^b^	c-fs	EB	8q21.1	No fs	EB
1p33	No fs	No EB	8q21.2	No fs	EB
1p34.1	No fs	No EB	8q21.3	No fs	No EB
1p34.2	No fs	EB	8q22.1^a^	c-fs	EB
1p34.3	No fs	EB	8q22.2	No fs	No EB
1p35	No fs	EB	8q22.3	r-fs	EB
1p36.1^b^	c-fs	EB	8q23	No fs	EB
1p36.2^b^	c-fs	EB	8q24.1	c-fs and r-fs	EB
1p36.3^b^	c-fs	EB	8q24.2	No fs	No EB
1q11	No fs	No EB	8q24.3^b^	c-fs	No EB
1q12	c-fs	No EB	9p11	No fs	No EB
1q21.1^b^	c-fs	No EB	9p12	No fs	No EB
1q21.2^b^	c-fs	No EB	9p13	No fs	EB
1q21.3^b^	c-fs	EB	9p21	c-fs and r-fs	EB
1q22	No fs	No EB	9p22	No fs	No EB
1q23	No fs	EB	9p23	No fs	EB
1q24	No fs	EB	9p24	No fs	EB
1q25	c-fs	EB	9q11	No fs	No EB
1q31	c-fs	EB	9q12^b^	c-fs	No EB
1q32.1	No fs	EB	9q13	No fs	No EB
1q32.2	No fs	EB	9q21.1	No fs	No EB
1q32.3	No fs	EB	9q21.2	No fs	EB
1q41	No fs	EB	9q21.3	No fs	EB
1q42.1^b^	c-fs	EB	9q22.1	c-fs	EB
1q42.2^b^	c-fs	No EB	9q22.2	No fs	EB
1q42.3^b^	c-fs	EB	9q22.3	No fs	EB
1q43	No fs	EB	9q31	No fs	EB
1q44^b^	c-fs	EB	9q32^a^	c-fs and r-fs	EB
20p11.1	No fs	No EB	9q33	No fs	EB
20p11.2	r-fs	EB	9q34.1	No fs	EB
20p12	c-fs	EB	9q34.2	No fs	No EB
20p13	No fs	EB	9q34.3	No fs	EB
20q11.1	No fs	No EB	Xp11.1	No fs	No EB
20q11.2	No fs	EB	Xp11.21	No fs	No EB
20q12	No fs	No EB	Xp11.22	No fs	EB
20q13.1	No fs	EB	Xp11.23	No fs	EB
20q13.2	No fs	No EB	Xp11.3	No fs	EB
20q13.3	No fs	No EB	Xp11.4	No fs	No EB
21p13	No fs	No EB	Xp21.3	No fs	No EB
21p12	No fs	No EB	Xp21.2	No fs	No EB
21p11.2	No fs	No EB	Xp21.1	No fs	EB
21p11.1	No fs	No EB	Xp22.1	No fs	EB
21q11	No fs	EB	Xp22.2	No fs	No EB
21q21.1	No fs	No EB	Xp22.3	c-fs	No EB
21q21.2	No fs	No EB	Xq11	No fs	EB
21q22.1	No fs	EB	Xq12.1	No fs	No EB
21q22.2	No fs	No EB	Xq12.2	No fs	No EB
21q22.3	No fs	EB	Xq13	No fs	EB
22p13	No fs	No EB	Xq21.1	No fs	EB
22p12	No fs	No EB	Xq21.2	No fs	No EB
22p11.2	No fs	No EB	Xq21.3	No fs	EB
22p11.1	No fs	No EB	Xq22.1	c-fs	EB
22q11.1	No fs	No EB	Xq22.2	No fs	EB
22q11.2	No fs	EB	Xq22.3	No fs	EB
22q12.1	No fs	EB	Xq23	No fs	EB
22q12.2^a^	c-fs	EB	Xq24	No fs	EB
22q12.3	No fs	EB	Xq25	No fs	EB
22q13.1^b^	r-fs	EB	Xq26	No fs	EB
22q13.2^b^	r-fs	EB	Xq27	c-fs and r-fs	EB
			Xq28	r-fs	No EB

All rare fragile sites (r-fs) and common fragile sites (c-fs) described by Schwartz and coworkers [39] and the evolutionary breakpoints (EBs) were plotted on the ideogram. Note that human chromosome Y is not included in the study. ^a^Fragile sites situated in bands comprising ≥ 6000 base pairs (bp) of tandem repeats in windows of 0.250 megabases (Mb). ^b^Fragile sites in bands with ≥ 10,000 bp of tandem repeats in windows of 0.250 Mb.

**Table 2:** Mean repeat size in base pairs per window of 0.250 megabases in each human chromosome analyzed.

Human chromosome	Mean number of repeats in B bands	Mean number of repeats in E bands	Mean number of repeats in FS bands	Mean number of repeats in no-FS bands
1	3,338.28	3,467.81	3,647.44**	3,213.66**
2	3,166.83	3,314.78	3,343.16	3,261.81
3	2,523.47**	3,104.42**	2,807.56	3,042.48
4	2,884.52	3,101.81	3,267.70	2,966.80
5	3,012.61	3,114.22	3,335.30*	2,943.73*
6	3,286.71	3,039.27	3,220.15	3,056.08
7	3,799.18	3,571.16	5,060.35**	2,830.71**
8	3,693.84*	3,065.77*	3,552.78*	3,118.19*
9	4,018.38	3,375.42	2,877.28	3,604.23
10	3,232.03	3,437.11	3,098.81*	3,651.40*
11	3,440.03	3,269.92	3,971.77**	3,017.47**
12	3,419.76	3,715.78	4,456.85**	3,112.39**
13	2,994.29	2,959.70	2,876.41	3,014.15
14	3,252.94	3,112.29	2,689.67*	3,267.68*
15	2,789.40*	3,302.97*	2,960.15	3,212.32
16	6,114.65**	4,414.38**	4,669.36	4,553.13
17	2,279.38**	4,346.49**	3,546.25*	4,314.01*
18	2,675.72*	3,175.75*	3,074.19	3,045.68
19	-	8,377.27	8,889.31	8,050.54
20	3,875.58	3,548.22	2,952.96*	3,755.64*
21	3,199.52*	5,062.95*	-	3,924.18
22	-	5,533.07	6,132.57*	5,020.60*
				
Total	3,237.46**	3,501.78**	3,735.08**	3,333.41**

Tukey-Kramer tests were calculated to evaluate the statistical difference among means in each chromosome and in the whole genome. In chromosomes 19 and 22 all bands are E bands and so no test is performed. No fragile sites have been described in the literature for human chromosome 21. Significant differences among band types are indicated as follows: *P = 0.05; **P = 0.002 (after Bonferroni correction applied to 22 samples). B bands, non-evolutionary bands; E bands, evolutionary bands; FS bands, bands containing fragile sites; no-FS bands, bands without fragile sites.
