# Supplementary material for: Is mammalian chromosomal evolution driven by regions of genome fragility?
Source: Genome Biol. 2006 Dec 8;7(12):R115. doi: 10.1186/gb-2006-7-12-r115 (PMC1794428; doi:10.1186/gb-2006-7-12-r115)
Supplement: Additional data file 1 — Multispecies alignments to all human chromosomes. [file gb-2006-7-12-r115-S1.pdf]

Supplementary Figure 1: Multispecies alignment of all human chromosomes. Red blocks represent the homology synteny blocks in the seven eutherian species compared and black blocks represent the homology regions with the chicken genome (http://www.ensembl.org). The location of human fragile sites (FRA) is given to the left of the ideogram of each human chromosome, red for common fragile sites and blue for rare fragile sites.

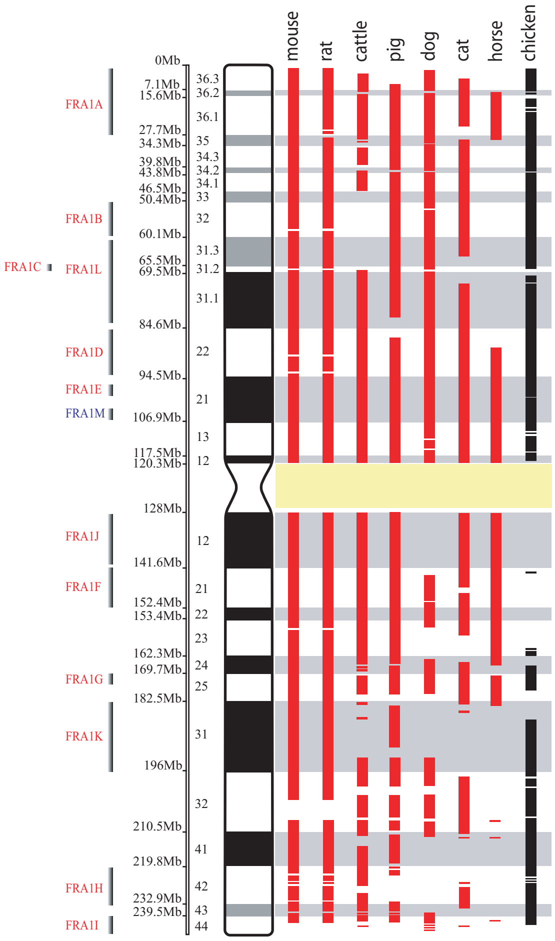

HSA 1

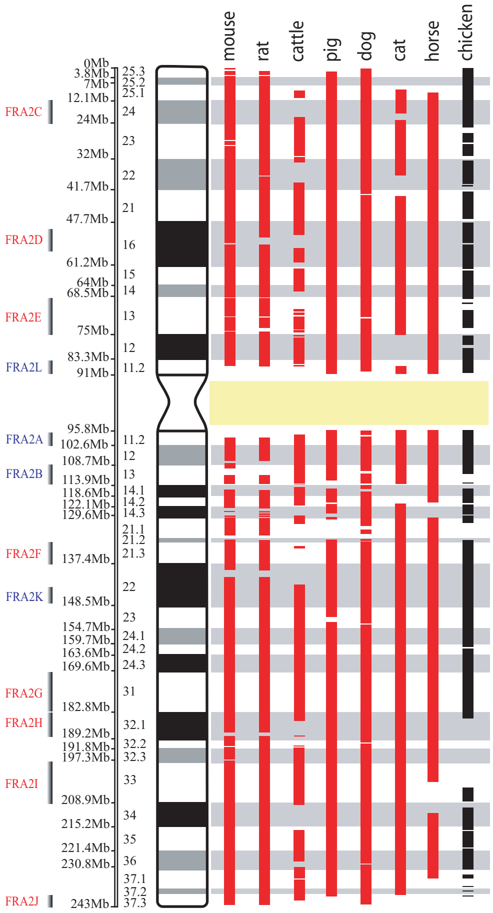

HSA 2

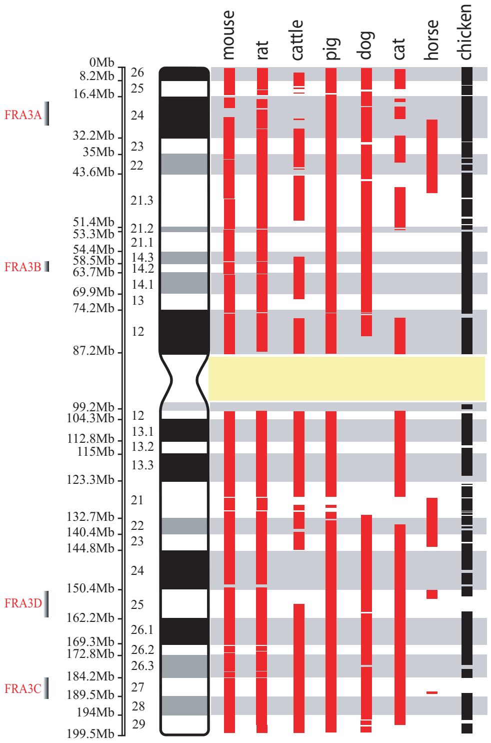

HSA 3

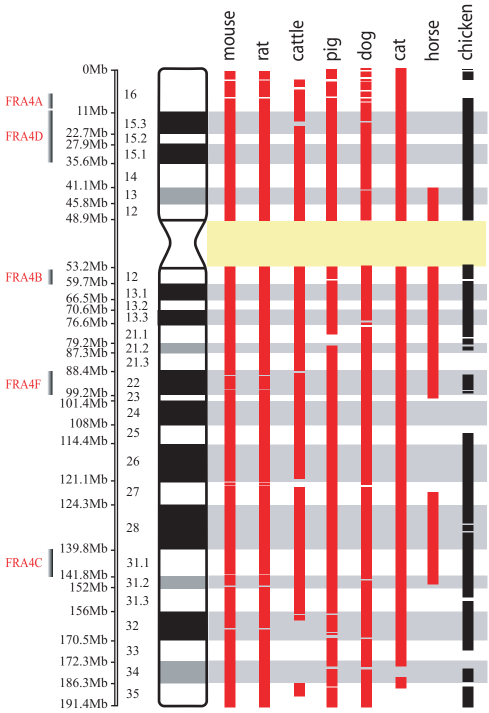

HSA 4

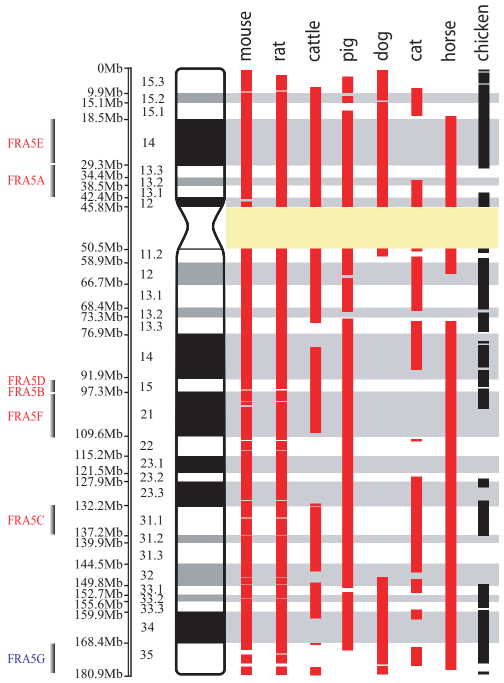

HSA 5

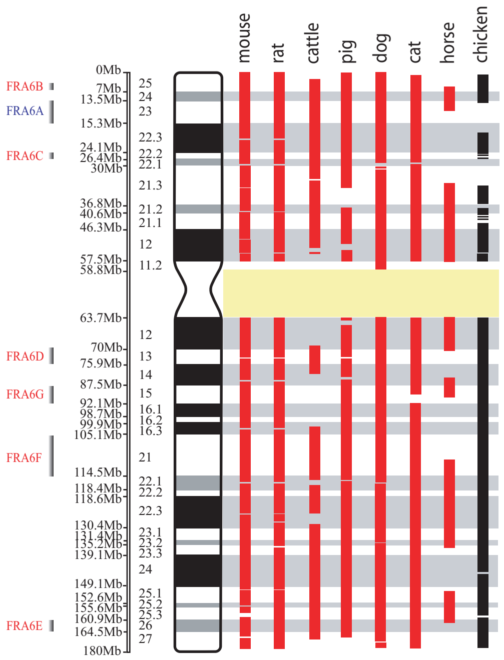

HSA 6

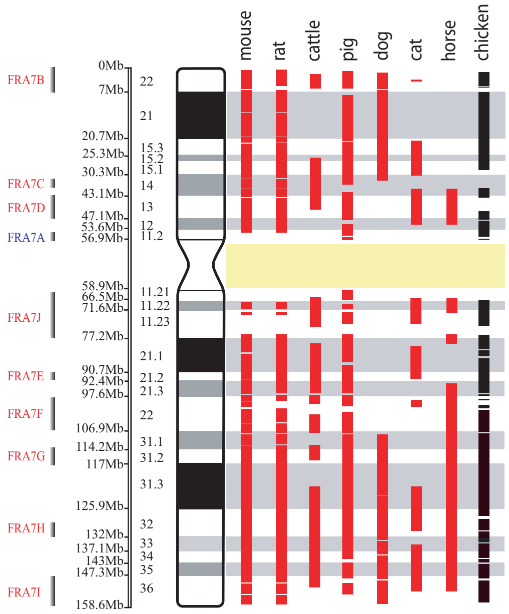

HSA 7

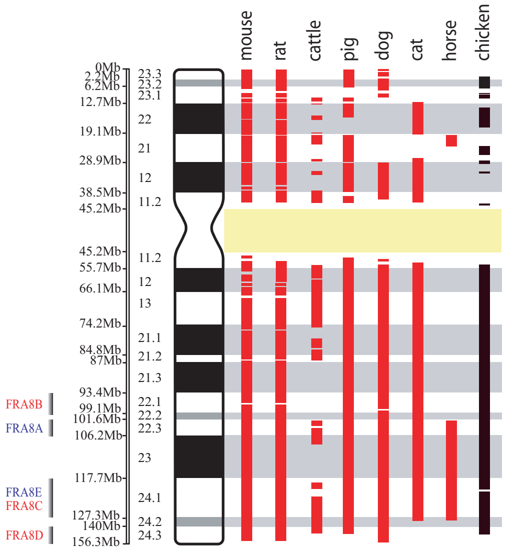

HSA 8

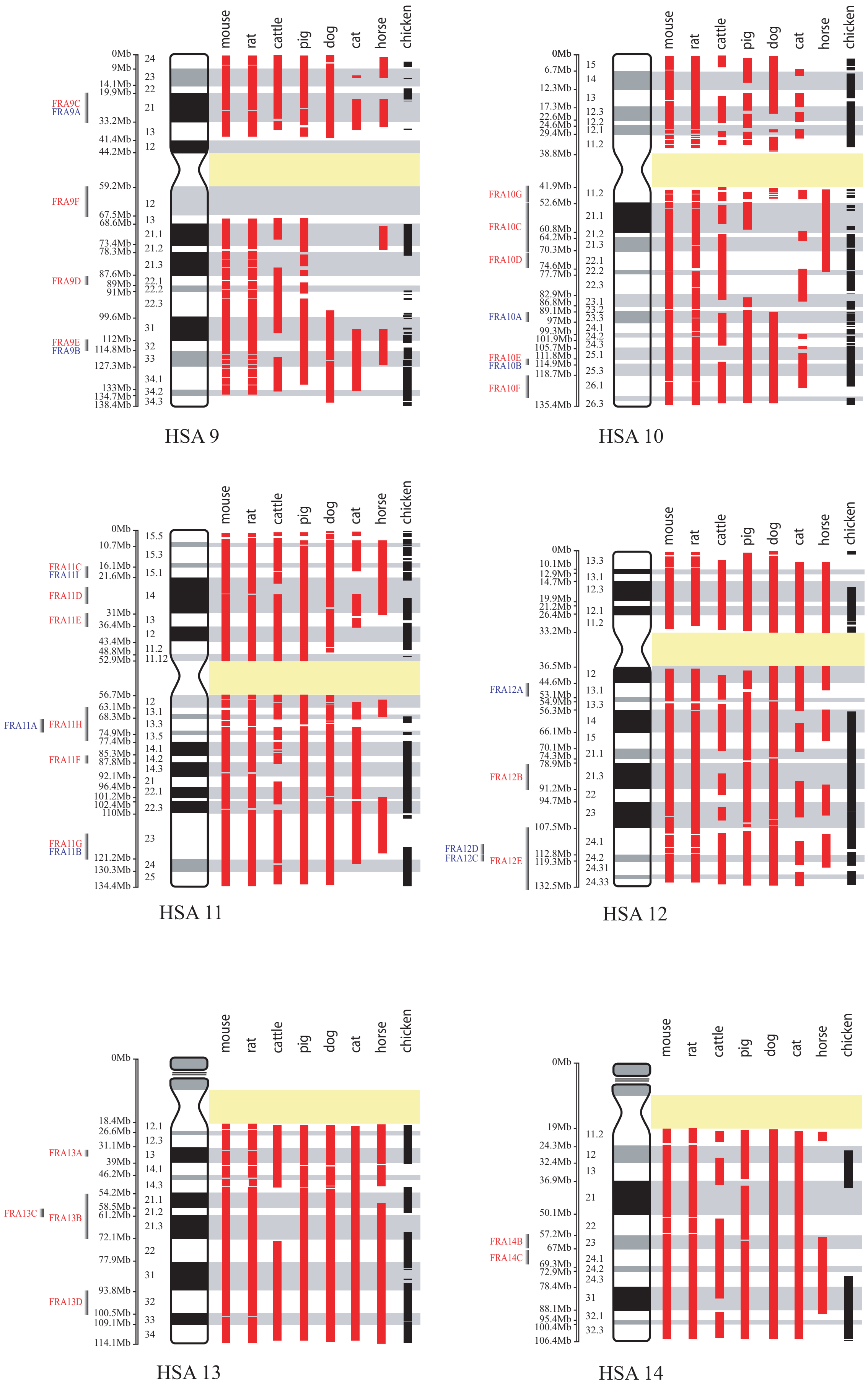
mouse
rat
cattle
pig
dog
cat
horse
chicken
FRA9C
FRA9A
FRA9F
FRA9D
FRA9E
FRA9B
HSA 9
FRA10G
FRA10C
FRA10D
FRA10A
FRA10E
FRA10B
FRA10F
HSA 10
FRA11C
FRA11I
FRA11D
FRA11E
FRA11A
FRA11H
FRA11F
FRA11G
FRA11B
HSA 11
FRA12A
FRA12B
FRA12D
FRA12C
FRA12E
HSA 12
FRA13A
FRA13C
FRA13B
FRA13D
HSA 13
FRA14B
FRA14C
HSA 14

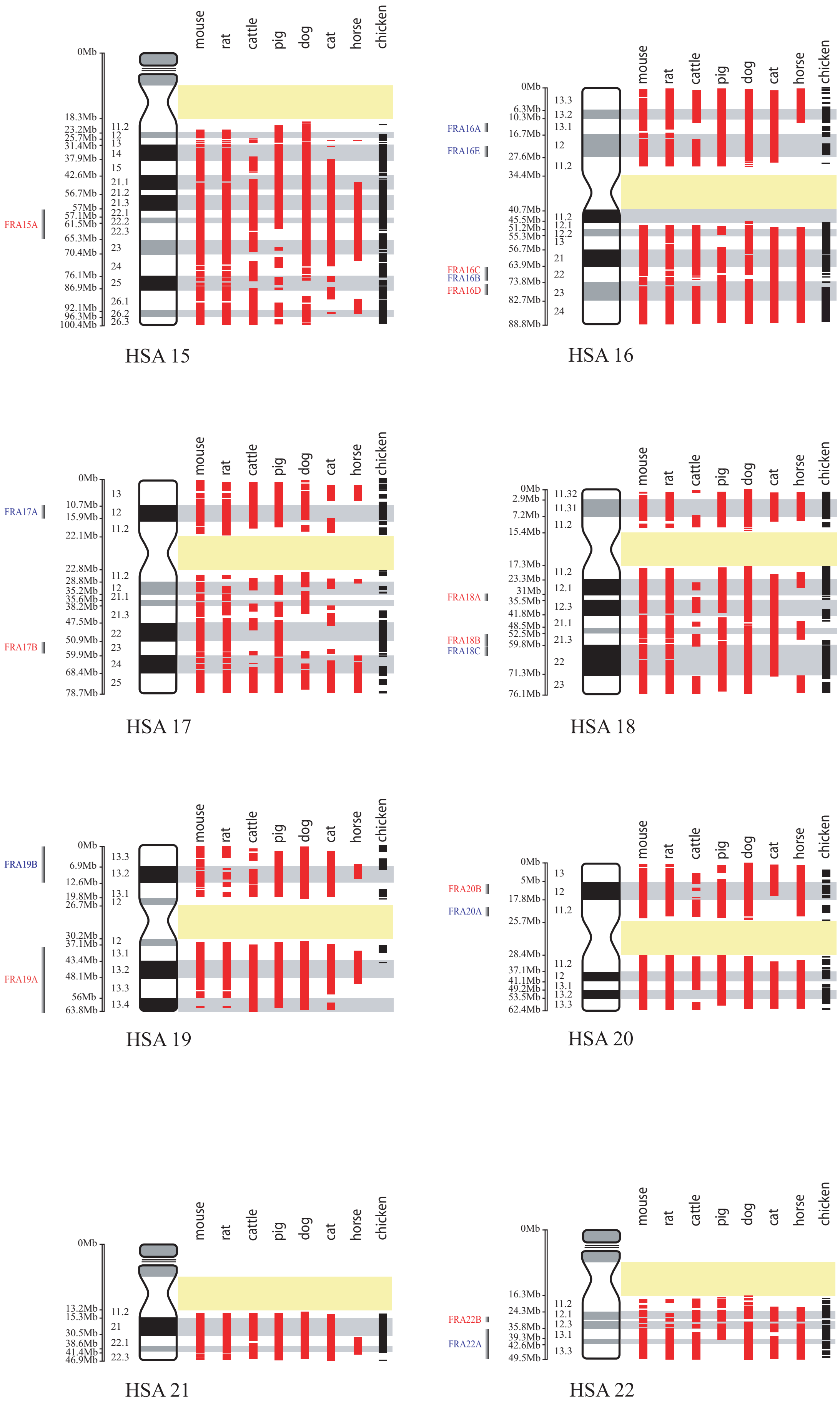

mouse
rat
cattle
pig
dog
cat
horse
chicken
FRA15A
HSA 15
FRA16A
FRA16E
FRA16C
FRA16B
FRA16D
HSA 16
FRA17A
FRA17B
HSA 17
FRA18A
FRA18B
FRA18C
HSA 18
FRA19B
FRA19A
HSA 19
FRA20B
FRA20A
HSA 20
HSA 21
FRA22B
FRA22A
HSA 22
