# Supplementary material for: Is mammalian chromosomal evolution driven by regions of genome fragility?
Source: Genome Biol. 2006 Dec 8;7(12):R115. doi: 10.1186/gb-2006-7-12-r115 (PMC1794428; doi:10.1186/gb-2006-7-12-r115)
Supplement: Additional data file 2 — Distribution of base pair tandem repeats along all human chromosomes represented as windows of 250,000 bp each. [file gb-2006-7-12-r115-S2.pdf]

Supplementary Figure 2: Distribution of base pair implicated tandem repeats along all human chromosomes represented as windows of 250000bp each. The distribution of base pair implicated tandem repeats in the pericentromeric and subtelomeric areas has been omitted in order to clarify the presence of outlayers.

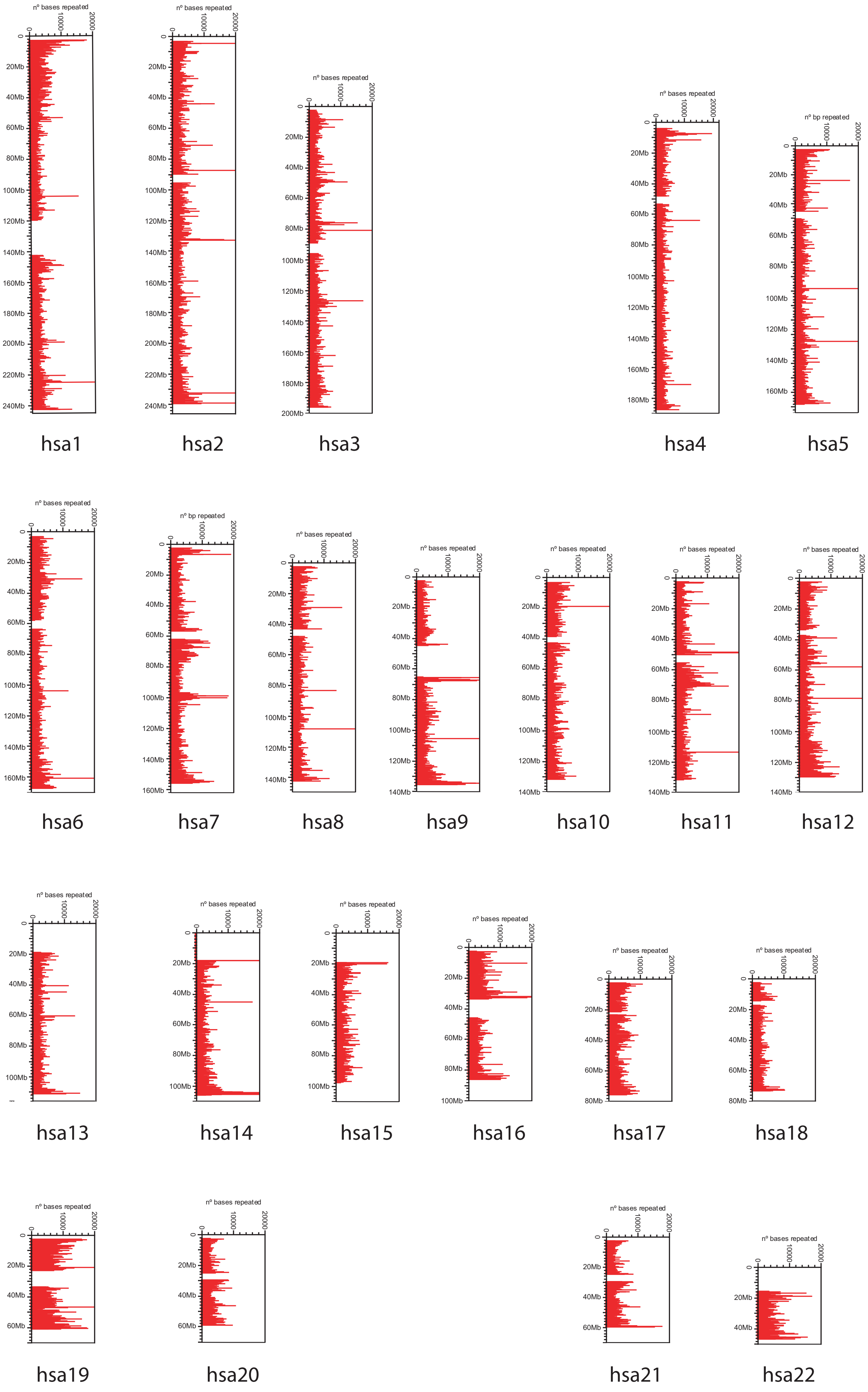
nº bases repeated
nº bp repeated
0
10000
20000
20Mb
40Mb
60Mb
80Mb
100Mb
120Mb
140Mb
160Mb
180Mb
200Mb
220Mb
240Mb
hsa1
hsa2
hsa3
hsa4
hsa5
hsa6
hsa7
hsa8
hsa9
hsa10
hsa11
hsa12
hsa13
hsa14
hsa15
hsa16
hsa17
hsa18
hsa19
hsa20
hsa21
hsa22
