# Supplementary material for: Is mammalian chromosomal evolution driven by regions of genome fragility?
Source: Genome Biol. 2006 Dec 8;7(12):R115. doi: 10.1186/gb-2006-7-12-r115 (PMC1794428; doi:10.1186/gb-2006-7-12-r115)

Supplementary Figure 3: Base pairs implicated in tandem repeats per chromosome. The blue line connects the mean values of all chromosomes and the horizontal black line represents the mean of all chromosomes. The Tukey-Kramer test reveals that chromosomes 19 differs statistically from the rest of the human chromosomes (P=0.05). Y axis indicates the number of bp implicated in repeats, whereas the X axis represents each human chromosome analysed.

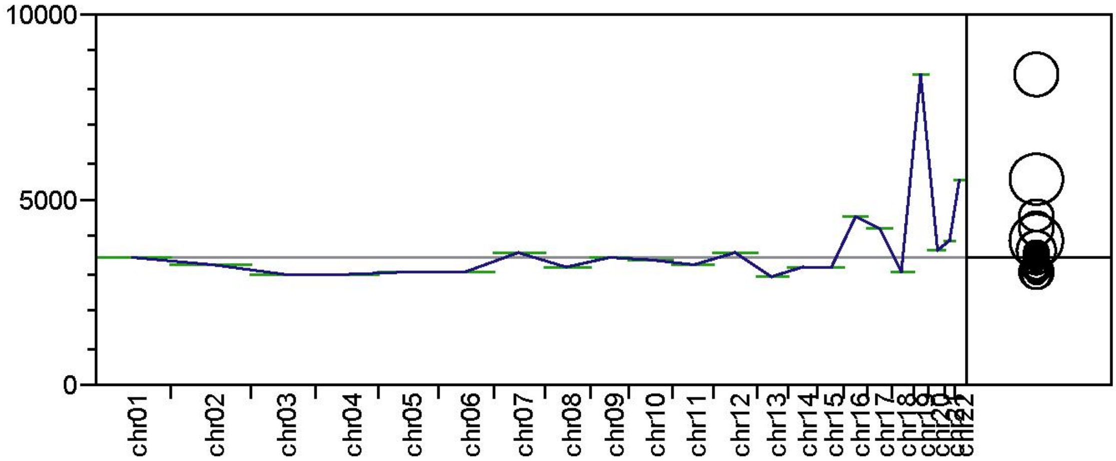

Supplement: Additional data file 3 — Base pairs implicated in tandem repeats per chromosome. [file gb-2006-7-12-r115-S3.pdf]
