# Supplementary material for: Is mammalian chromosomal evolution driven by regions of genome fragility?
Source: Genome Biol. 2006 Dec 8;7(12):R115. doi: 10.1186/gb-2006-7-12-r115 (PMC1794428; doi:10.1186/gb-2006-7-12-r115)
Supplement: Additional data file 4 — List of all of the homologous syntenic blocks (HSB) detected. [file gb-2006-7-12-r115-S4.pdf]

Table S1: List of the homologous syntenic blocks (HSB) indicating the chromosomal position (band) and nucleotide (nt) position, the species where they have been detected and the size of each evolutionary breakpoint region (EBR).

Human chr	from (band)	from (nt position)	to (band)	to (nt position)	species	HSB's size	EBR's size
1	1p36.3	1585750	1p36.1	27194712	cat	25608962	4349779
1	1p35	31544491	1p31.3	65460889	cat	33916398	5241871
1	1p31.1	70702760	1q21	144725443	cat	74022683	6013100
1	1q21	150738543	1q23	161580755	cat	10842212	2515146
1	1q24	164095901	1q31	182950099	cat	18854198	3388849
1	1q31	186338948	1q31	186348198	cat	9250	11446400
1	1q32	197794598	1q41	210818867	cat	13024269	4304261
1	1q41	215123128	1q41	215219029	cat	95901	9494297
1	1q42	224713326	1q42	224722192	cat	8866	970791
1	1q42	225692983	1q43	236369302	cat	10676319	8745705
1	1q44	245115007	1q44	245124084	cat	9077	
2	2p25.1	9729021	2p24	16125926	cat	6396905	5056086
2	2p24	21182012	2p22	35873540	cat	14691528	3192735
2	2p22	39066275	2p22	39067575	cat	1300	4340841
2	2p21	43408416	2p12	75076821	cat	31668405	11892752
2	2p11.2	86969573	2q13	113448884	cat	26479311	6869832
2	2q14.2	120318716	2q37.3	241838347	cat	121519631	
3	3p26	1451745	3p25	13166476	cat	11714731	3453681
3	3p24	16620157	3p24	17806005	cat	1185848	1556165
3	3p24	19362170	3p24	25489220	cat	6127050	5008588
3	3p24	30497808	3p22	38951627	cat	8453819	6804014
3	3p21.3	45755641	3p21.2	51753995	cat	5998354	1296085
3	3p21.2	53050080	3p21.2	53081586	cat	31506	23684218
3	3p12	76765804	3q21	123699021	cat	46933217	6830072
3	3q21	130529093	3q21	130535798	cat	6705	4210912
3	3q22	134746710	3q29	197215954	cat	62469244	
4	4p16	657750	4q34	175316794	cat	174659044	7211134
4	4q34	182527928	4q35	186652876	cat	4124948	
5	5p15.3	6632091	5p15.1	17309480	cat	10677389	18547653
5	5p13.2	35857133	5q11.2	52508337	cat	16651204	2182710
5	5q11.2	54691047	5q13.2	70405708	cat	15714661	3614016
5	5q13.3	74019724	5q14	88105156	cat	14085432	22485354
5	5q22	110590510	5q22	111490607	cat	900097	12608190
5	5q23.2	124098797	5q32	145444492	cat	21345695	2746505
5	5q32	148190997	5q33.1	151050039	cat	2859042	7627295
5	5q33.3	158677334	5q34	162035708	cat	3358374	9024340
5	5q35	171060048	5q35	176460698	cat	5400650	4265267
5	5q35	180725965	5q35	180732931	cat	6966	
6	6p25	282097	6p22.1	28564483	cat	28282386	1362383
6	6p22.1	29926866	6q15	88825718	cat	58898852	3313092
6	6q16.1	92138810	6q27	170557420	cat	78418610	
7	7p22	4840400	7p22	4840520	cat	120	17634548
7	7p15.3	22475068	7p14	30980430	cat	8505362	7005766
7	7p14	37986196	7p12	50340856	cat	12354660	14482633
7	7q11.21	64823489	7q11.23	75669671	cat	10846182	5256903
7	7q21.1	80926574	7q21.3	92802263	cat	11875689	6485422
7	7q22	99287685	7q22	101473628	cat	2185943	2408800
7	7q22	103882428	7q22	103884909	cat	2481	16898382
7	7q31.3	120783291	7q32	131601627	cat	10818336	4500197
7	7q33	136101824	7q36	154560138	cat	18458314	
8	8p23.1	12616348	8p21	19634073	cat	7017725	7641715

8	8p21	27275788	8p11.2	42016234	cat	14740446	11241970
8	8q11.2	53258204	8q24.2	134539163	cat	81280959	
9	9p23	11905550	9p23	12700223	cat	794673	8366883
9	9p21	21067106	9p13	35747923	cat	14680817	70715622
9	9q31	106463545	9q34.2	133317257	cat	26853712	
10	10p15	5940612	10p14	8267092	cat	2326480	5146913
10	10p13	13414005	10p12.3	17429831	cat	4015826	1150014
10	10p12.3	18579845	10p12.2	22770461	cat	4190616	8936078
10	10p11.2	31706539	10p11.2	35651317	cat	3944778	7696391
10	10q11.2	43347708	10q11.2	50765558	cat	7417850	9399669
10	10q21.1	60165227	10q21.3	65674798	cat	5509571	8835377
10	10q22.1	74510175	10q23.1	86044527	cat	11534352	13621564
10	10q24.2	99666091	10q24.3	102403107	cat	2737016	2315845
10	10q24.3	104718952	10q24.3	106017367	cat	1298415	2639739
10	10q25.1	108657106	10q26.1	126717000	cat	18059894	
11	11p15.5	181724	11p15.5	2829324	cat	2647600	2376409
11	11p15.4	5205733	11p15.1	18468547	cat	13262814	8272110
11	11p14	26740657	11p13	32496231	cat	5755574	394324
11	11p13	32890555	11p12	36658940	cat	3768385	24600613
11	11q12	61259553	11q13.3	68958076	cat	7698523	3431547
11	11q13.4	72389623	11q22.1	101035333	cat	28645710	1389980
11	11q22.2	102425313	11q24	126166349	cat	23741036	
12	12p13.3	5420747	12q14	56835830	cat	51415083	5303350
12	12q14	62139180	12q21.1	71496217	cat	9357037	16394251
12	12q21.3	87890468	12q23	103277571	cat	15387103	7335060
12	12q24.1	110612631	12q24.31	121223165	cat	10610534	4240113
12	12q24.31	125463278	12q24.33	133089157	cat	7625879	
13	13q12.1	21693700	13q34	113064379	cat	91370679	
14	14q11.2	18913418	14q32.3	104293998	cat	85380580	
15	15q13	27067283	15q13	27070326	cat	3043	502114
15	15q13	27572440	15q13	27694790	cat	122350	4867752
15	15q14	32562542	15q14	32762771	cat	200229	4689652
15	15q14	37452423	15q24	74939446	cat	37487023	1192962
15	15q24	76132408	15q25	81314475	cat	5182067	2954314
15	15q25	84268789	15q25	89026239	cat	4757450	7759209
15	15q26.3	96785448	15q26.3	97094240	cat	308792	
16	16p13.3	672593	16p11.2	29857874	cat	29185281	17377477
16	16q12.1	47235351	16q24	89688677	cat	42453326	
17	17p13	1275985	17p13	9751740	cat	8475755	4498750
17	17p12	14250490	17p11.2	19675749	cat	5425259	7206612
17	17q11.2	26882361	17q12	32312773	cat	5430412	5769238
17	17q21.2	38082011	17q21.3	41186169	cat	3104158	1107133
17	17q21.3	42293302	17q22	48595350	cat	6302048	8169268
17	17q23	56764618	17q23	59679495	cat	2914877	2655879
17	17q24	62335374	17q25	80354230	cat	18018856	
18	18p11.32	970800	18p11.2	10113453	cat	9142653	11665587
18	18q11.2	21779040	18q23	71611061	cat	49832021	
19	19p13.3	104393	19p13.1	19314097	cat	19209704	2839603
19	19p12	22153700	19p12	22155800	cat	2100	12218488
19	19q12	34374288	19q13.3	55108809	cat	20734521	3952766
19	19q13.4	59061575	19q13.4	62808963	cat	3747388	
20	20p13	411734	20p12	14155629	cat	13743895	19361218
20	20q11.2	33516847	20q13.3	62172325	cat	28655478	
21	21q11.2	15255433	21q22.3	46941497	cat	31686064	
22	22q11.2	17537649	22q11.2	17540987	cat	3338	4039613
22	22q11.2	21580600	22q12.3	30831199	cat	9250599	1857288
22	22q12.3	32688487	22q13.1	37884099	cat	5195612	2840471
22	22q13.2	40724570	22q13.3	49015920	cat	8291350	

X	Xp22.2	10607902	Xq28	151592786	cat	140984884	
1	1p36.3	656945	1p36.3	657101	cattle	156	2225
1	1p36.3	659326	1p36.3	659491	cattle	165	526168
1	1p36.3	1185659	1p36.2	15066499	cattle	13880840	929603
1	1p36.1	15996102	1p35	32178408	cattle	16182306	650282
1	1p35	32828690	1p35	32856534	cattle	27844	2213902
1	1p34.3	35070436	1p34.3	36041724	cattle	971288	2878731
1	1p34.3	38920455	1p34.1	46148253	cattle	7227798	12457868
1	1p32	58606121	1q24	164602310	cattle	105996189	1753530
1	1q24	166355840	1q24	166559098	cattle	203258	1502853
1	1q24	168061951	1q24	168143429	cattle	81478	1883807
1	1q25	170027236	1q25	180080269	cattle	10053033	2705590
1	1q31	182785859	1q31	183169425	cattle	383566	6445208
1	1q31	189614633	1q31	189622181	cattle	7548	3971151
1	1q31	193593332	1q32	198313571	cattle	4720239	1206000
1	1q32	199519571	1q32	203961724	cattle	4442153	739303
1	1q32	204701027	1q41	211126688	cattle	6425661	6288680
1	1q41	217415368	1q42	223312565	cattle	5897197	1343338
1	1q42	224655903	1q42	224672451	cattle	16548	40875
1	1q42	224713326	1q42	224980083	cattle	266757	1696061
1	1q42	226676144	1q43	236369146	cattle	9693002	866256
1	1q43	237235402	1q43	237817065	cattle	581663	550999
1	1q43	238368064	1q44	241073844	cattle	2705780	3956391
1	1q44	245030235	1q44	245030340	cattle	105	
2	2p25.1	9568707	2p25.1	10756219	cattle	1187512	9659935
2	2p24	20416154	2p23	31448970	cattle	11032816	67012
2	2p23	31515982	2p22	33582120	cattle	2066138	3804300
2	2p22	37386420	2p16	51217330	cattle	13830910	3493886
2	2p16	54711216	2p16	58344712	cattle	3633496	3027597
2	2p15	61372309	2p14	67595383	cattle	6223074	2331453
2	2p13	69926836	2p13	70274004	cattle	347168	191660
2	2p13	70465664	2p13	70466012	cattle	348	1050591
2	2p13	71516603	2p13	74400121	cattle	2883518	243030
2	2p13	74643151	2p13	74714596	cattle	71445	962556
2	2p12	75677152	2p12	75816416	cattle	139264	3525425
2	2p12	79341841	2p11.2	85513118	cattle	6171277	220798
2	2p11.2	85733916	2p11.2	86962867	cattle	1228951	8257528
2	2q11.2	95220395	2q13	111831391	cattle	16610996	1606917
2	2q13	113438308	2q13	113867305	cattle	428997	687046
2	2q14.1	114554351	2q14.2	121015788	cattle	6461437	6694753
2	2q14.3	127710541	2q21.1	131827274	cattle	4116733	3408385
2	2q21.3	135235659	2q21.3	135943001	cattle	707342	7966210
2	2q22	143909211	2q31	183695912	cattle	39786701	4598359
2	2q32.1	188294271	2q32.2	188294465	cattle	194	2738919
2	2q32.2	191033384	2q32.2	191148604	cattle	115220	23615
2	2q32.2	191172219	2q32.2	191200413	cattle	28194	64631
2	2q32.2	191265044	2q32.2	191337954	cattle	72910	459830
2	2q32.2	191797784	2q34	209083854	cattle	17286070	7056857
2	2q35	216140711	2q36	224431140	cattle	8290429	3869747
2	2q36	228300887	2q37.1	232637976	cattle	4337089	1594843
2	2q37.1	234232819	2q37.3	242142249	cattle	7909430	495566
2	2q37.3	242637815	2q37.3	242639521	cattle	1706	431869
2	2q37.3	243071390	2q37.3	243071502	cattle	112	
3	3p26	4526981	3p25	10160699	cattle	5633718	1125547
3	3p25	11286246	3p25	11296261	cattle	10015	3991957
3	3p25	15288218	3p25	15374753	cattle	86535	5935035
3	3p24	21309788	3p24	21309938	cattle	150	5954077
3	3p24	27264015	3p22	40167131	cattle	12903116	2275443

3	3p22	42442574	3p22	42528308	cattle	85734	3021779
3	3p21.3	45550087	3p21.3	50149543	cattle	4599456	6829322
3	3p14.3	56978865	3p13	71608550	cattle	14629685	5344235
3	3p12	76952785	3q21	126014321	cattle	49061536	2584573
3	3q21	128598894	3q21	130535798	cattle	1936904	1164778
3	3q21	131700576	3q21	131701180	cattle	604	193266
3	3q21	131894446	3q22	137949578	cattle	6055132	1995101
3	3q22	139944679	3q23	144060636	cattle	4115957	5636573
3	3q24	149697209	3q24	149742427	cattle	45218	4091920
3	3q25	153834347	3q29	198918819	cattle	45084472	
4	4p16	657092	4p16	657757	cattle	665	3604402
4	4p16	4262159	4p16	5815903	cattle	1553744	894641
4	4p16	6710544	4p15.3	17202205	cattle	10491661	2741026
4	4p15.3	19943231	4q22	88978287	cattle	69035056	135235
4	4q22	89113522	4q26	120943179	cattle	31829657	2626041
4	4q27	123569220	4q32	156604414	cattle	33035194	496470
4	4q32	157100884	4q32	160110729	cattle	3009845	14727044
4	4q34	174837773	4q34	174838785	cattle	1012	11361297
4	4q34	186200082	4q35	188229437	cattle	2029355	
5	5p15.3	6747464	5q13.3	74052866	cattle	67305402	6592876
5	5q14	80645742	5q14	80647195	cattle	1453	17344
5	5q14	80664539	5q21	108558257	cattle	27893718	14154241
5	5q23.2	122712498	5q23.2	122790149	cattle	77651	8637061
5	5q23.3	131427210	5q23.3	132144366	cattle	717156	98210
5	5q23.3	132242576	5q32	146444172	cattle	14201596	3320190
5	5q32	149764362	5q34	161261955	cattle	11497593	1538165
5	5q34	162800120	5q34	162807563	cattle	7443	6192273
5	5q35	168999836	5q34	169660774	cattle	660938	6835593
5	5q35	176496367	5q35	176658350	cattle	161983	88708
5	5q35	176747058	5q35	180306851	cattle	3559793	
6	6p25	2938393	6p21.3	32560586	cattle	29622193	44050
6	6p21.3	32604636	6p12	53135692	cattle	20531056	2485960
6	6p12	55621652	6p12	56893536	cattle	1271884	14434101
6	6q13	71327637	6q14	83026802	cattle	11699165	16821559
6	6q16.2	99848361	6q22.1	116447426	cattle	16599065	1434714
6	6q22.1	117882140	6q22.3	127599865	cattle	9717725	1539607
6	6q22.3	129139472	6q27	168047271	cattle	38907799	
7	7p22	2104812	7p22	6456942	cattle	4352130	5863598
7	7p21	12320540	7p21	12403564	cattle	83024	10815838
7	7p15.3	23219402	7p15.3	23274348	cattle	54946	2765439
7	7p15.2	26039787	7p13	45667651	cattle	19627864	9788866
7	7p11.2	55456517	7p11.2	55488695	cattle	32178	251338
7	7p11.2	55740033	7p11.2	55775620	cattle	35587	9292508
7	7q11.21	65068128	7q11.23	75845912	cattle	10777784	3516478
7	7q21.1	79362390	7q21.3	96967711	cattle	17605321	876493
7	7q22	97844204	7q22	100328878	cattle	2484674	2984642
7	7q22	103313520	7q31.1	107193133	cattle	3879613	4200239
7	7q31.1	111393372	7q31.2	116849416	cattle	5456044	4204471
7	7q31.3	121053887	7q36	151447936	cattle	30394049	
8	8p23.1	9782861	8p23.1	11567741	cattle	1784880	1148323
8	8p22	12716064	8p22	12909249	cattle	193185	2831333
8	8p22	15740582	8p22	17751798	cattle	2011216	1319179
8	8p22	19070977	8p21	19269574	cattle	198597	336497
8	8p21	19606071	8p21	22698277	cattle	3092206	4577517
8	8p21	27275794	8p21	28021411	cattle	745617	2834977
8	8p12	30856388	8p12	32478311	cattle	1621923	5219387
8	8p12	37697698	8p11.2	42492813	cattle	4795115	9578639
8	8q11.2	52071452	8q11.2	52071773	cattle	321	1186430

8	8q11.2	53258203	8q12	59218602	cattle	5960399	222560
8	8q12	59441162	8q21.1	74465892	cattle	15024730	4845275
8	8q21.1	79311167	8q21.1	82118658	cattle	2807491	174717
8	8q21.1	82293375	8q21.2	86141302	cattle	3847927	10553597
8	8q22.1	96694899	8q22.1	96695260	cattle	361	4688677
8	8q22.2	101383937	8q22.3	103242039	cattle	1858102	837621
8	8q22.3	104079660	8q23	109800606	cattle	5720946	7525264
8	8q23	117325870	8q23	117447292	cattle	121422	754458
8	8q24.1	118201750	8q24.1	120105393	cattle	1903643	306420
8	8q24.1	120411813	8q24.1	120513872	cattle	102059	1780466
8	8q24.1	122294338	8q24.3	144938033	cattle	22643695	
9	9p24	356238	9p21	32563350	cattle	32207112	530731
9	9p21	33094081	9p13	37856457	cattle	4762376	26858072
9	9q13	64714529	9q21.1	72692449	cattle	7977920	7138380
9	9q21.3	79830829	9q21.3	79831533	cattle	704	1362542
9	9q21.3	81194075	9q21.3	81194393	cattle	318	2941994
9	9q21.3	84136387	9q31	110814313	cattle	26677926	6846617
9	9q33	117660930	9q34.2	133812198	cattle	16151268	
10	10p15	17643	10p15	5600634	cattle	5582991	7869405
10	10p13	13470039	10p12.2	23561177	cattle	10091138	4552749
10	10p12.1	28113926	10p11.2	30174969	cattle	2061043	3014204
10	10p11.2	33189173	10p11.2	33189252	cattle	79	668330
10	10p11.2	33857582	10p11.2	36080601	cattle	2223019	2416208
10	10q11.2	38496809	10q11.2	43537563	cattle	5040754	1103247
10	10q11.2	44640810	10q11.2	51023128	cattle	6382318	935629
10	10q11.2	51958757	10q11.2	51959007	cattle	250	1390416
10	10q21.1	53349423	10q21.1	58013421	cattle	4663998	3667140
10	10q21.2	61680561	10q23.1	86150931	cattle	24470370	4252713
10	10q23.3	90403644	10q24.3	105084506	cattle	14680862	7099519
10	10q25.2	112184025	10q26.3	135121309	cattle	22937284	
11	11p15.5	945884	11p15.5	2972880	cattle	2026996	634042
11	11p15.4	3606922	11p15.1	18086368	cattle	14479446	253131
11	11p15.1	18339499	11p14	23516713	cattle	5177214	2853375
11	11p14	26370088	11q12	59884931	cattle	33514843	1024144
11	11q12	60909075	11q13.3	68708644	cattle	7799569	3156579
11	11q13.4	71865223	11q13.5	76385891	cattle	4520668	1057193
11	11q14.1	77443084	11q14.1	79558251	cattle	2115167	1291879
11	11q14.1	80850130	11q14.1	82834337	cattle	1984207	558482
11	11q14.1	83392819	11q14.3	88767869	cattle	5375050	5982849
11	11q21	94750718	11q22.3	104299321	cattle	9548603	3348645
11	11q22.3	107647966	11q24	122966757	cattle	15318791	1704268
11	11q24	124671025	11q25	134055118	cattle	9384093	
12	12p13.3	4262199	12p11.2	32950041	cattle	28687842	7639582
12	12p12	40589623	12q13.2	54689770	cattle	14100147	1105333
12	12q13.3	55795103	12q21.1	70543513	cattle	14748410	9557621
12	12q21.3	80101134	12q22	93768986	cattle	13667852	2827149
12	12q23	96596135	12q23	104099339	cattle	7503204	4872496
12	12q24.1	108971835	12q24.33	132906549	cattle	23934714	
13	13q12.1	20823750	13q12.1	20824838	cattle	1088	33210
13	13q12.1	20858048	13q14.3	52250584	cattle	31392536	20015595
13	13q22	72266179	13q34	113064619	cattle	40798440	
14	14q11.2	18904698	14q11.2	22900668	cattle	3995970	6433091
14	14q12	29333759	14q21	37562478	cattle	8228719	6186963
14	14q21	43749441	14q21	43749643	cattle	202	4815751
14	14q21	48565394	14q21	48573670	cattle	8276	2436374
14	14q22	51010044	14q22	51093480	cattle	83436	220551
14	14q22	51314031	14q31	84084304	cattle	32770273	5643406
14	14q32.1	89727710	14q32.3	103233692	cattle	13505982	

15	15q11.2	20423754	15q11.2	20452813	cattle	29059	5370199
15	15q13	25823012	15q13	26034151	cattle	211139	1106522
15	15q13	27140673	15q13	29349843	cattle	2209170	7009599
15	15q14	36359442	15q15	41092619	cattle	4733177	151727
15	15q15	41244346	15q15	41673925	cattle	429579	908928
15	15q15	42582853	15q15	42589480	cattle	6627	4159750
15	15q21.1	46749230	15q22.3	62574353	cattle	15825123	908250
15	15q22.3	63482603	15q23	70139318	cattle	6656715	2572680
15	15q24	72711998	15q25	80399292	cattle	7687294	3480214
15	15q25	83879506	15q26.3	99785313	cattle	15905807	
16	16p13.3	1218338	16p13.1	11341619	cattle	10123281	7380109
16	16p12	18721728	16p11.2	31200072	cattle	12478344	15263811
16	16q12.1	46463883	16q22	69496123	cattle	23032240	387479
16	16q22	69883602	16q23	70570481	cattle	686879	1090249
16	16q22	71660730	16q23	72828038	cattle	1167308	1396493
16	16q23	74224531	16q24	89509172	cattle	15284641	
17	17p13	962865	17p11.2	18056321	cattle	17093456	8590387
17	17q11.2	26646708	17q11.2	26647099	cattle	391	589335
17	17q11.2	27236434	17q12	33264971	cattle	6028537	4208221
17	17q21.2	37473192	17q21.3	40823819	cattle	3350627	2382334
17	17q21.3	43206153	17q21.3	43206254	cattle	101	2744307
17	17q21.3	45950561	17q22	48905415	cattle	2954854	9133727
17	17q23	58039142	17q23	59812060	cattle	1772918	2433562
17	17q24	62245622	17q24	62956701	cattle	711079	1668993
17	17q24	64625694	17q25	81429122	cattle	16803428	
18	18p11.32	148520	18p11.32	901054	cattle	752534	6168942
18	18p11.31	7069996	18p11.2	13875520	cattle	6805524	10448449
18	18q12.1	24323969	18q21.1	28824451	cattle	4500482	3979500
18	18q12.2	32803951	18q12.3	40737968	cattle	7934017	3540942
18	18q21.1	44278910	18q21.1	48362259	cattle	4083349	4466860
18	18q21.2	52829119	18q22	60432639	cattle	7603520	907277
18	18q22	61339916	18q22	61356094	cattle	16178	10349412
18	18q23	71705506	18q23	77632403	cattle	5926897	
19	19p13.3	737411	19p13.3	752327	cattle	14916	12770
19	19p13.3	765097	19p13.3	1921309	cattle	1156212	288214
19	19p13.3	2209523	19p13.3	5093605	cattle	2884082	2786001
19	19p13.2	7879606	19p13.1	12837529	cattle	4957923	271560
19	19p13.1	13109089	19p13.1	19463359	cattle	6354270	4323065
19	19p12	23786424	19p12	23786909	cattle	485	10587307
19	19q12	34374216	19q12	34380163	cattle	5947	393076
19	19q12	34773239	19q13.4	63627610	cattle	28854371	
20	20p13	336697	20p13	367193	cattle	30496	2219848
20	20p13	2587041	20p12	5854003	cattle	3266962	2207293
20	20p12	8061296	20p12	10555474	cattle	2494178	5645276
20	20p12	16200750	20p12	16670419	cattle	469669	752132
20	20p12	17422551	20p11.2	23566574	cattle	6144023	7911733
20	20q11.2	31478307	20q13.1	49194684	cattle	17716377	6377760
20	20q13.3	55572444	20q13.3	63246709	cattle	7674265	
21	21q11.2	14665471	21q22.1	34224306	cattle	19558835	2138385
21	21q22.1	36362691	21q22.3	45205395	cattle	8842704	
22	22q11.2	16644972	22q11.2	16685103	cattle	40131	589660
22	22q11.2	17274763	22q11.2	22451056	cattle	5176293	1469323
22	22q11.2	23920379	22q12.2	30013052	cattle	6092673	1509143
22	22q12.3	31522195	22q12.3	35668902	cattle	4146707	744130
22	22q13.1	36413032	22q13.1	36413528	cattle	496	516209
22	22q13.1	36929737	22q13.3	49316132	cattle	12386395	
X	Xp22.3	1081075	Xq13.3	75082961	cattle	74001886	25981742
X	Xq22.1	101064703	Xq23	112920937	cattle	11856234	2916034

X	Xq23	115836971	Xq28	151591310	cattle	35754339	
1	1p36.3	891967	1p36.2	8014674	chicken	7122707	332039
1	1p36.2	8346713	1p36.2	11280458	chicken	2933745	4507562
1	1p36.1	15788020	1p36.1	19966204	chicken	4178184	2184001
1	1p36.1	22150205	1p36.1	23037279	chicken	887074	54394
1	1p36.1	23091673	1p36.1	26808478	chicken	3716805	28691
1	1p36.1	26837169	1p35	31511526	chicken	4674357	246620
1	1p35	31758146	1p34.3	39001878	chicken	7243732	213843
1	1p34.3	39215721	1p34.2	40232231	chicken	1016510	761012
1	1p34.2	40993243	1p34.2	42132810	chicken	1139567	1149889
1	1p34.2	43282699	1p31.2	66552275	chicken	23269576	3385611
1	1p31.1	69937886	1p31.1	75478444	chicken	5540558	773992
1	1p31.1	76252436	1p21	103834130	chicken	27581694	109465
1	1p21	103943595	1p13	109278244	chicken	5334649	983388
1	1p13	110261632	1p13	110944090	chicken	682458	753892
1	1p13	111697982	1p13	115305369	chicken	3607387	600523
1	1p13	115905892	1p12	119700778	chicken	3794886	38820215
1	1q23	158520993	1q23	158771765	chicken	250772	1356465
1	1q23	160128230	1q23	162129946	chicken	2001716	4177896
1	1q24	166307842	1q25	177159077	chicken	10851235	12351659
1	1q31	189510736	1q32	197561509	chicken	8050773	274824
1	1q32	197836333	1q32	199875897	chicken	2039564	130131
1	1q32	200006028	1q32	200444937	chicken	438909	364359
1	1q32	200809296	1q32	206363172	chicken	5553876	28608
1	1q32	206391780	1q42	222902670	chicken	16510890	223354
1	1q42	223126024	1q42	223813187	chicken	687163	599878
1	1q42	224413065	1q42	224873215	chicken	460150	840392
1	1q42	225713607	1q44	243424274	chicken	17710667	
2	2p25.3	35395	2p23	24462884	chicken	24427489	1223325
2	2p23	25686209	2p23	27784720	chicken	2098511	3219
2	2p23	27787939	2p23	28551956	chicken	764017	398549
2	2p23	28950505	2p23	31402964	chicken	2452459	1011648
2	2p22	32414612	2p22	38353401	chicken	5938789	0
2	2p22	38353401	2p22	40844899	chicken	2491498	1171063
2	2p21	42015962	2p21	47378320	chicken	5362358	543628
2	2p16	47921948	2p16	53253989	chicken	5332041	585024
2	2p16	53839013	2p15	61677308	chicken	7838295	950646
2	2p15	62627954	2p13	68716960	chicken	6089006	196581
2	2p13	68913541	2p13	69695825	chicken	782284	2041333
2	2p13	71737158	2p13	73432621	chicken	1695463	2817590
2	2p12	76250211	2p12	77699659	chicken	1449448	2297029
2	2p12	79996688	2p11.2	88752637	chicken	8755949	9842303
2	2q11.2	98594940	2q13	109734101	chicken	11139161	2512044
2	2q13	112246145	2q13	112807186	chicken	561041	1380330
2	2q14.1	114187516	2q14.2	120089519	chicken	5902003	193999
2	2q14.2	120283518	2q14.3	127356291	chicken	7072773	535917
2	2q14.3	127892208	2q21.1	131738252	chicken	3846044	1266438
2	2q21.2	133004690	2q24.2	160450291	chicken	27445601	36795
2	2q24.2	160487086	2q32.1	183465674	chicken	22978588	20266307
2	2q33	203731981	2q33	208730414	chicken	4998433	1539115
2	2q34	210269529	2q35	216408870	chicken	6139341	377319
2	2q35	216786189	2q35	220332157	chicken	3545968	38660
2	2q35	220370817	2q36	230759214	chicken	10388397	987890
2	2q37.1	231747104	2q37.1	233271732	chicken	1524628	51577
2	2q37.1	233323309	2q37.1	234162762	chicken	839453	2140197
2	2q37.2	236302959	2q37.3	239853848	chicken	3550889	1522789
2	2q37.3	241376637	2q37.3	242244186	chicken	867549	
3	3p26	38251	3p25	9401361	chicken	9363110	38119

3	3p25	9439480	3p25	9968818	chicken	529338	373689
3	3p25	10342507	3p25	13896319	chicken	3553812	0
3	3p25	13896319	3p25	15112644	chicken	1216325	355150
3	3p25	15467794	3p23	32805550	chicken	17337756	164357
3	3p23	32969907	3p22	37067234	chicken	4097327	2256
3	3p22	37069490	3p22	37840031	chicken	770541	112818
3	3p22	37952849	3p22	39170169	chicken	1217320	1715850
3	3p22	40886019	3p22	42280144	chicken	1394125	769103
3	3p22	42547254	3p22	42892325	chicken	345071	
3	3p22	43049247	3p21.3	46425544	chicken	3376297	1774797
3	3p21.3	46920035	3p21.3	48200341	chicken	1280306	0
3	3p21.3	48200341	3p21.3	49188285	chicken	987944	334548
3	3p21.3	49522833	3p21.2	52454845	chicken	2932012	779828
3	3p21.2	53234673	3p12	75384120	chicken	22149447	572703
3	3p12	75956823	3p12	87417864	chicken	11461041	769302
3	3q11.2	88187166	3q11.2	90390621	chicken	2203455	4684763
3	3q11.2	95075384	3q11.2	98986611	chicken	3911227	1570815
3	3q12	100557426	3q13.2	113163762	chicken	12606336	570161
3	3q13.2	113733923	3q13.3	122746458	chicken	9012535	709006
3	3q21	123455464	3q21	123668981	chicken	213517	272884
3	3q21	123941865	3q21	126784631	chicken	2842766	1149963
3	3q21	127934594	3q21	130016373	chicken	2081779	69735
3	3q21	130086108	3q21	130373357	chicken	287249	209418
3	3q21	130582775	3q21	131030058	chicken	447283	547660
3	3q21	131577718	3q22	134860720	chicken	3283002	146687
3	3q22	135007407	3q22	137100488	chicken	2093081	102427
3	3q22	137202915	3q22	137832526	chicken	629611	408061
3	3q22	138240587	3q23	142267635	chicken	4027048	318960
3	3q23	142586595	3q24	146396455	chicken	3809860	1245343
3	3q24	147641798	3q24	149667183	chicken	2025385	527345
3	3q24	150194528	3q25	152657728	chicken	2463200	4022225
3	3q25	156679953	3q26.3	177663502	chicken	20983549	4139067
3	3q26.3	181802569	3q27	184542346	chicken	2739777	3884886
3	3q27	188427232	3q29	195890199	chicken	7462967	1648780
3	3q29	197538979	3q29	199171045	chicken	1632066	
4	4p16	833223	4p16	951495	chicken	118272	185693
4	4p16	1137188	4p16	3532198	chicken	2395010	5927840
4	4p16	9460038	4p12	48535002	chicken	39074964	4766307
4	4q12	53301309	4q12	57166633	chicken	3865324	1266591
4	4q12	58433224	4q21.1	78344753	chicken	19911529	1147037
4	4q21.2	79491790	4q21.2	79819632	chicken	327842	111161
4	4q21.2	79930793	4q21.2	86138934	chicken	6208141	3522514
4	4q22	89661448	4q22	95596429	chicken	5934981	205013
4	4q22	95801442	4q22	97970827	chicken	2169385	12832212
4	4q25	110803039	4q28	128763245	chicken	17960206	158617
4	4q28	128921862	4q28	130154003	chicken	1232141	305250
4	4q28	130459253	4q31.3	153767367	chicken	23308114	663595
4	4q31.3	154430962	4q33	171049735	chicken	16618773	1259942
4	4q34	172309677	4q33	172648942	chicken	339265	5876385
4	4q34	178525327	4q35	186718890	chicken	8193563	6707
4	4q35	186725597	4q35	191201761	chicken	4476164	
5	5p15.3	526079	5p15.3	1147393	chicken	621314	107180
5	5p15.3	1254573	5p15.3	4902020	chicken	3647447	1523601
5	5p15.3	6425621	5p13.3	31589007	chicken	25163386	9127761
5	5p13.1	40716768	5p12	45729795	chicken	5013027	4269310
5	5p11.2	49999105	5q11.2	53553691	chicken	3554586	1034189
5	5q11.2	54587880	5q13.2	68600910	chicken	14013030	2864783
5	5q13.2	71465693	5q13.3	76313068	chicken	4847375	2296359

5	5q14	78609427	5q14	79534645	chicken	925218	199650
5	5q14	79734295	5q14	86265663	chicken	6531368	1847073
5	5q14	88112736	5q15	95873089	chicken	7760353	466745
5	5q15	96339834	5q21	104502724	chicken	8162890	21461656
5	5q23.2	125964380	5q23.3	128192279	chicken	2227899	2595225
5	5q23.3	130787504	5q31.2	137466957	chicken	6679453	11887366
5	5q32	149354323	5q33.3	158645657	chicken	9291334	630316
5	5q33.3	159275973	5q35	174203875	chicken	14927902	4269359
5	5q35	178473234	5q35	179400258	chicken	927024	
6	6p25	281821	6p23	13595107	chicken	13313286	4634506
6	6p22.3	18229613	6p22.2	24704874	chicken	6475261	54121
6	6p22.3	24758995	6p22.2	25809635	chicken	1050640	24476
6	6p22.2	25834111	6p22.1	26430578	chicken	596467	7218809
6	6p21.3	33649387	6p21.3	35546505	chicken	1897118	2855
6	6p21.3	35549360	6p21.3	36308562	chicken	759202	1121055
6	6p21.2	37429617	6p21.1	41039056	chicken	3609439	408769
6	6p21.1	41447825	6p21.1	42153061	chicken	705236	404925
6	6p21.1	42557986	6p21.1	42944344	chicken	386358	137885
6	6p21.1	43082229	6p21.1	43300164	chicken	217935	111554
6	6p21.1	43411718	6p21.1	44388292	chicken	976574	75096
6	6p21.1	44463388	6p12	52489510	chicken	8026122	222845
6	6p12	52712355	6p12	58195502	chicken	5483147	4110704
6	6q12	62306206	6q24	144336485	chicken	82030279	473871
6	6q24	144810356	6q25.3	159167979	chicken	14357623	905739
6	6q25.3	160073718	6q27	170811262	chicken	10737544	
7	7p22	490865	7p22	5573946	chicken	5083081	408958
7	7p22	5982904	7p22	6297062	chicken	314158	749900
7	7p21	7046962	7p15.1	29364849	chicken	22317887	8825649
7	7p14	38190498	7p13	43453589	chicken	5263091	1933895
7	7p13	45387484	7p12	47252589	chicken	1865105	655323
7	7p12	47907912	7p11.2	54457923	chicken	6550011	526197
7	7p11.2	54984120	7p11.2	55275641	chicken	291521	11024724
7	7q11.21	66300365	7q11.23	75788139	chicken	9487774	1003668
7	7q11.23	76791807	7q21.1	83000325	chicken	6208518	206528
7	7q21.1	83206853	7q21.1	86473042	chicken	3266189	149970
7	7q21.1	86623012	7q21.3	96391869	chicken	9768857	851094
7	7q21.3	97242963	7q21.3	97594284	chicken	351321	675405
7	7q22	98269689	7q22	98701555	chicken	431866	1850294
7	7q22	100551849	7q22	101802475	chicken	1250626	0
7	7q22	101802475	7q31.1	107357576	chicken	5555101	24349
7	7q31.1	107381925	7q32	126771269	chicken	19389344	1666066
7	7q32	128437335	7q32	129759213	chicken	1321878	1109276
7	7q32	130868489	7q33	132174783	chicken	1306294	220116
7	7q33	132394899	7q33	133525046	chicken	1130147	278361
7	7q33	133803407	7q33	134112641	chicken	309234	46110
7	7q33	134158751	7q33	134790746	chicken	631995	25333
7	7q33	134816079	7q34	140903503	chicken	6087424	1010172
7	7q34	141913675	7q34	142725902	chicken	812227	1137961
7	7q35	143863863	7q36	148163633	chicken	4299770	2316405
7	7q36	150480038	7q36	158238099	chicken	7758061	
8	8p23.3	1414919	8p23.1	6643537	chicken	5228618	2831468
8	8p23.1	9475005	8p23.1	9677262	chicken	202257	362736
8	8p23.1	10039998	8p23.1	11748385	chicken	1708387	1159588
8	8p22	12907973	8p22	17973193	chicken	5065220	5186875
8	8p21	23160068	8p21	25961234	chicken	2801166	2052006
8	8p21	28013240	8p12	29104530	chicken	1091290	3182138
8	8p12	32286668	8p12	32902572	chicken	615904	9802307
8	8p11.2	42704879	8p11.2	43060066	chicken	355187	5368218

8	8q11.2	48428284	8q24.1	120187610	chicken	71759326	434183
8	8q24.1	120621793	8q24.3	144770093	chicken	24148300	
9	9p24	4813532	9p24	8643415	chicken	3829883	3849796
9	9p23	12493211	9p23	12841201	chicken	347990	2601797
9	9p13	15442998	9p21	21049498	chicken	5606500	2216479
9	9p21	23265977	9p21	23928674	chicken	662697	12642988
9	9p13	36571662	9p13	37017964	chicken	446302	31690290
9	9q21.1	68708254	9q21.3	79748088	chicken	11039834	10937644
9	9q22.2	90685732	9q22.3	91715989	chicken	1030257	354828
9	9q22.3	92070817	9q22.3	92494637	chicken	423820	576042
9	9q22.3	93070679	9q22.3	94020993	chicken	950314	4108909
9	9q22.3	98129902	9q22.3	98419936	chicken	290034	407447
9	9q22.3	98827383	9q22.3	98995468	chicken	168085	23032
9	9q22.3	99018500	9q31	100292759	chicken	1274259	818574
9	9q31	101111333	9q31	101539322	chicken	427989	3496849
9	9q31	105036171	9q31	106891529	chicken	1855358	1085031
9	9q31	107976560	9q31	108258424	chicken	281864	616713
9	9q31	108875137	9q31	109310005	chicken	434868	265576
9	9q31	109575581	9q32	112732207	chicken	3156626	366723
9	9q32	113098930	9q32	113898629	chicken	799699	61473
9	9q32	113960102	9q33	120464612	chicken	6504510	175663
9	9q33	120640275	9q33	127235485	chicken	6595210	323538
9	9q34.1	127559023	9q34.3	136897210	chicken	9338187	29416
9	9q34.3	136926626	9q34.3	138311658	chicken	1385032	
10	10p15	21542	10p15	4812889	chicken	4791347	592759
10	10p15	5405648	10p15	5971424	chicken	565776	1631228
10	10p14	7602652	10p13	14323662	chicken	6721010	1268497
10	10p13	15592159	10p11.2	37131882	chicken	21539723	5467967
10	10q11.2	42599849	10q11.2	43021605	chicken	421756	2168011
10	10q11.2	45189616	10q11.2	45479311	chicken	289695	3576776
10	10q11.2	49056087	10q11.2	50449782	chicken	1393695	39516
10	10q11.2	50489298	10q11.2	50758433	chicken	269135	2890357
10	10q21.1	53648790	10q21.1	54051981	chicken	403191	1183965
10	10q21.1	55235946	10q21.1	56015683	chicken	779737	3852489
10	10q21.1	59868172	10q21.1	60261224	chicken	393052	821042
10	10q21.2	61082266	10q21.3	68715724	chicken	7633458	632367
10	10q21.3	69348091	10q22.1	74576201	chicken	5228110	290173
10	10q22.2	74866374	10q22.3	80875574	chicken	6009200	1378821
10	10q22.3	82254395	10q22.3	82396943	chicken	142548	3494273
10	10q23.1	85891216	10q23.2	88940694	chicken	3049478	314348
10	10q23.3	89255042	10q23.3	89872817	chicken	617775	1080282
10	10q23.3	90953099	10q23.3	96285673	chicken	5332574	533476
10	10q23.3	96819149	10q24.1	97286106	chicken	466957	508019
10	10q24.1	97794125	10q24.1	98085815	chicken	291690	28548
10	10q24.1	98114363	10q24.1	98459744	chicken	345381	194450
10	10q24.1	98654194	10q24.2	101825872	chicken	3171678	199258
10	10q24.3	102025130	10q24.3	102632157	chicken	607027	33439
10	10q24.3	102665596	10q24.3	103900153	chicken	1234557	1844
10	10q24.3	103901997	10q24.3	104174942	chicken	272945	43847
10	10q24.3	104218789	10q26.1	125796126	chicken	21577337	3932971
10	10q26.2	129729097	10q26.3	134469962	chicken	4740865	
11	11p15.5	194122	11p15.5	480528	chicken	286406	468888
11	11p15.5	949416	11p15.5	3108914	chicken	2159498	1236261
11	11p15.4	4345175	11p15.4	6619322	chicken	2274147	661506
11	11p15.4	7280828	11p15.4	7682866	chicken	402038	391926
11	11p15.4	8074792	11p15.2	13723547	chicken	5648755	130236
11	11p15.2	13853783	11p15.2	14246001	chicken	392218	9921
11	11p15.2	14255922	11p15.1	18722630	chicken	4466708	968074

11	11p15.1	19690704	11p14	22806005	chicken	3115301	4287531
11	11p14	27093536	11p13	33662373	chicken	6568837	795812
11	11p13	34458185	11p11.12	47568862	chicken	13110677	1556022
11	11p11.12	49124884	11p11.12	49762223	chicken	637339	18119469
11	11q13.1	67881692	11q13.4	70536070	chicken	2654378	1689355
11	11q13.4	72225425	11q13.5	74955638	chicken	2730213	2361387
11	11q13.5	77317025	11q22.3	109984997	chicken	32667972	1220072
11	11q23	111205069	11q23	112813858	chicken	1608789	5688990
11	11q23	118502848	11q25	134035906	chicken	15533058	
12	12p13.3	105031	12p13.3	2677452	chicken	2572421	12850562
12	12p12.3	15528014	12p11.2	27072760	chicken	11544746	687687
12	12p11.2	27760447	12p11.2	29012215	chicken	1251768	496476
12	12p11.2	29508691	12p11.2	32963699	chicken	3455008	6483571
12	12p12	39447270	12q13.1	45760489	chicken	6313219	10861318
12	12q14	56621807	12q23	100302277	chicken	43680470	69173
12	12q23	100371450	12q23	106679140	chicken	6307690	114065
12	12q23	106793205	12q24.1	111444658	chicken	4651453	646973
12	12q24.1	112091631	12q24.31	119917101	chicken	7825470	1845335
12	12q24.31	121762436	12q24.33	132074589	chicken	10312153	
13	13q12.1	19457601	13q14.1	40296935	chicken	20839334	11841884
13	13q14.3	52138819	13q21.2	60886228	chicken	8747409	9417440
13	13q21.3	70303668	13q31	79413807	chicken	9110139	59500
13	13q31	79473307	13q31	80773032	chicken	1299725	4325654
13	13q31	85098686	13q31	85273279	chicken	174593	4777699
13	13q31	90050978	13q33	107431655	chicken	17380677	481752
13	13q33	107913407	13q34	114110259	chicken	6196852	
14	14q12	25018730	14q21	38941572	chicken	13922842	34467339
14	14q24.3	73408911	14q32.3	104706611	chicken	31297700	0
14	14q32.3	104706611	14q32.3	105067346	chicken	360735	
15	15q11.2	20384846	15q11.2	20613794	chicken	228948	2861491
15	15q12	23475285	15q13	26255370	chicken	2780085	1524203
15	15q13	27779573	15q13	29156503	chicken	1376930	1542958
15	15q13	30699461	15q15	38172249	chicken	7472788	76504
15	15q15	38248753	15q15	38858827	chicken	610074	65275
15	15q15	38924102	15q15	41264077	chicken	2339975	186219
15	15q15	41450296	15q21.1	42740113	chicken	1289817	379762
15	15q21.1	43119875	15q21.1	43354999	chicken	235124	458811
15	15q21.1	43813810	15q22.3	62063017	chicken	18249207	128798
15	15q22.3	62191815	15q22.3	62842540	chicken	650725	433033
15	15q22.3	63275573	15q23	68779125	chicken	5503552	132177
15	15q23	68911302	15q23	69862655	chicken	951353	23274
15	15q23	69885929	15q24	72881488	chicken	2995559	1052288
15	15q24	73933776	15q25	76714973	chicken	2781197	1176763
15	15q25	77891736	15q25	82586947	chicken	4695211	0
15	15q25	82586947	15q25	83289442	chicken	702495	588279
15	15q25	83877721	15q25	88047379	chicken	4169658	1048639
15	15q26.1	89096018	15q26.1	89297551	chicken	201533	270336
15	15q26.1	89567887	15q26.3	99902596	chicken	10334709	
16	16p13.3	4653	16p13.3	367902	chicken	363249	20718
16	16p13.3	388620	16p13.3	713176	chicken	324556	0
16	16p13.3	713176	16p13.3	1211786	chicken	498610	280558
16	16p13.3	1492344	16p13.3	1690111	chicken	197767	107620
16	16p13.3	1797731	16p13.3	2227488	chicken	429757	1231838
16	16p13.3	3459326	16p13.3	4464150	chicken	1004824	373671
16	16p13.3	4837821	16p13.3	5087800	chicken	249979	1038960
16	16p13.3	6126760	16p13.1	11256892	chicken	5130132	1222141
16	16p13.1	12479033	16p13.1	14691984	chicken	2212951	890559
16	16p13.1	15582543	16p12	18845522	chicken	3262979	3069630

16	16p12	21915152	16p12	27145727	chicken	5230575	1885920
16	16p11.2	29031647	16p11.2	29246265	chicken	214618	15925310
16	16q11.2	45171575	16q21	57104792	chicken	11933217	303422
16	16q21	57408214	16q22	65534181	chicken	8125967	89885
16	16q22	65624066	16q22	66963693	chicken	1339627	2008531
16	16q22	68972224	16q22	69877357	chicken	905133	3046599
16	16q22	72923956	16q23	73911511	chicken	987555	1962374
16	16q23	75873885	16q24	88314857	chicken	12440972	
17	17p13	5979	17p13	2186447	chicken	2180468	25217
17	17p13	2211664	17p13	3582554	chicken	1370890	129729
17	17p13	3712283	17p13	4157201	chicken	444918	1300185
17	17p13	5457386	17p13	6237594	chicken	780208	1999135
17	17p13	8236729	17p12	15175628	chicken	6938899	643311
17	17p12	15818939	17p11.2	16277751	chicken	458812	0
17	17p11.2	16277751	17p11.2	21267116	chicken	4989365	1378363
17	17q11.2	22645479	17q11.2	23695741	chicken	1050262	3290
17	17q11.2	23699031	17q11.2	25877954	chicken	2178923	6045940
17	17q12	31923894	17q12	33224540	chicken	1300646	6865450
17	17q21.3	40089990	17q21.3	44844292	chicken	4754302	2216862
17	17q21.3	47061154	17q23	52438820	chicken	5377666	98824
17	17q23	52537644	17q23	57679550	chicken	5141906	158142
17	17q23	57837692	17q23	59250410	chicken	1412718	218263
17	17q23	59468673	17q24	60193977	chicken	725304	4181837
17	17q24	64375814	17q25	69567432	chicken	5191618	1546054
17	17q25	71113486	17q25	73615159	chicken	2501673	3870366
17	17q25	77485525	17q25	78451936	chicken	966411	
18	18p11.32	106808	18p11.2	8396947	chicken	8290139	1585721
18	18p11.2	9982668	18p11.2	13880099	chicken	3897431	2903695
18	18q11.2	16783794	18q12.2	31002282	chicken	14218488	300149
18	18q12.2	31302431	18q12.2	32623593	chicken	1321162	1099712
18	18q12.2	33723305	12q12.2	35195192	chicken	1471887	4115861
18	18q12.3	39311053	18q21.1	43136264	chicken	3825211	477378
18	18q21.1	43613642	18q21.1	44010145	chicken	396503	12496724
18	18q21.3	56506869	18q22	59805496	chicken	3298627	60238
18	18q21.3	59865734	18q23	72873406	chicken	13007672	779134
18	18q23	73652540	18q23	75997696	chicken	2345156	
19	19p13.3	258125	19p13.3	2259575	chicken	2001450	2343251
19	19p13.3	4602826	19p13.2	8669945	chicken	4067119	7369104
19	19p13.1	16039049	19p13.1	19477919	chicken	3438870	3008084
19	19p12	22486003	19p12	22589748	chicken	103745	11647335
19	19q12	34237083	19q13.1	39684108	chicken	5447025	3951242
19	19q13.2	43635350	19q13.2	43769906	chicken	134556	
20	20p13	2915386	20p13	4178097	chicken	1262711	534339
20	20p13	4712436	20p12	5126548	chicken	414112	0
20	20p12	5126548	20p12	13746140	chicken	8619592	325824
20	20p12	14071964	20p12	16679764	chicken	2607800	3285075
20	20p11.2	19964839	20p11.2	22497809	chicken	2532970	2254333
20	20p11.2	24752142	20p11.2	25079253	chicken	327111	4454498
20	20q11.2	29533751	20q11.2	30901944	chicken	1368193	695381
20	20q11.2	31597325	20q11.2	34605332	chicken	3008007	1083835
20	20q11.2	35689167	20q11.2	36223597	chicken	534430	461761
20	20q11.2	36685358	20q12	39429119	chicken	2743761	5174543
20	20q13.1	44603662	20q13.1	47532990	chicken	2929328	149851
20	20q13.1	47682841	20q13.3	57203103	chicken	9520262	4447644
20	20q13.3	61650747	20q13.3	62375657	chicken	724910	
21	21q11.2	14459219	21q22.3	44000557	chicken	29541338	2136985
21	21q22.3	46137542	21q22.3	46849606	chicken	712064	
22	22q11.2	15953171	22q11.2	16988468	chicken	1035297	704316

22	22q11.2	17692784	22q11.2	20654768	chicken	2961984	1107184
22	22q11.2	21761952	22q12.1	27778011	chicken	6016059	214578
22	22q12.2	27992589	22q12.3	30830867	chicken	2838278	277625
22	22q12.3	31108492	22q13.2	41335900	chicken	10227408	4375
22	22q13.2	41340275	22q13.3	44687806	chicken	3347531	821095
22	22q13.3	45508901	22q13.3	47541745	chicken	2032844	222886
22	22q13.3	47764631	22q13.3	48628371	chicken	863740	
X	Xp22.33	147302	Xp11.3	45492563	chicken	45345261	729748
X	Xp11.3	46222311	Xp11.3	46708676	chicken	486365	7282092
X	Xq11.2	53990768	Xq11.2	54220775	chicken	230007	8417082
X	Xq11.2	62637857	Xq11.2	62895349	chicken	257492	6111894
X	Xq13.1	69007243	Xq13.1	70577663	chicken	1570420	2946159
X	Xq13.2	73523822	Xq21.1	77188027	chicken	3664205	621222
X	Xq21.1	77809249	Xq21.1	78851521	chicken	1042272	1286855
X	Xq21.1	80138376	Xq22.1	99990589	chicken	19852213	3226615
X	Xq22.2	103217204	Xq22.3	105923657	chicken	2706453	3129071
X	Xq23	109052728	Xq23	111469562	chicken	2416834	6424531
X	Xq24	117894093	Xq25	128378662	chicken	10484569	2535504
X	Xq26.2	130914166	Xq26.3	137667500	chicken	6753334	1223458
X	Xq27.1	138890958	Xq27.2	140619971	chicken	1729013	6088592
X	Xq27.3	146708563	Xq28	149826349	chicken	3117786	
1	1p36.33	885422	1p36.22	10382321	dog	9496899	11278
1	1p36.22	10393599	1p35.1	33641688	dog	23248089	6656
1	1p35.1	33648344	1p34.2	43218471	dog	9570127	60369
1	1p34.2	43278840	1p32.3	53002763	dog	9723923	870
1	1p32.3	53003633	1p31.2	67643657	dog	14640024	1
1	1p31.2	67643658	1p13.2	111718814	dog	44075156	3899
1	1p13.2	111722713	1p13.2	113947730	dog	2225017	855
1	1p13.2	113948585	1p11.2	120409185	dog	6460600	23386096
1	1q21.1	143795281	1q21.3	150079679	dog	6284398	58459
1	1q21.3	150138138	1q23.1	154730542	dog	4592404	8797840
1	1q24.1	163528382	1q25.2	176541386	dog	13013004	17135974
1	1q31.3	193677360	1q32.1	199920606	dog	6243246	12048
1	1q32.1	199932654	1q32.1	203176244	dog	3243590	18428
1	1q32.1	203194672	1q41	212088558	dog	8893886	24449519
1	1q43	236538077	1q44	243460072	dog	6921995	313942
1	1q44	243774014	1q44	244065183	dog	291169	262817
1	1q44	244328000	1q44	245065271	dog	737271	241224
1	1q44	245306495	1q44	245422849	dog	116354	
2	2p25.3	3575	2p21	42896353	dog	42892778	2658
2	2p21	42899011	2p13.3	71199984	dog	28300973	1
2	2p13.3	71199985	2p11.2	89446527	dog	18246542	5617505
2	2q11.2	95064032	2q11.2	96656378	dog	1592346	22967
2	2q11.2	96679345	2q13	109821753	dog	13142408	356240
2	2q13	110177993	2q13	113855973	dog	3677980	28159
2	2q13	113884132	2q14.1	114038202	dog	154070	107764
2	2q14.1	114145966	2q21.1	130390523	dog	16244557	921035
2	2q21.1	131311558	2q21.1	131747793	dog	436235	690920
2	2q21.2	132438713	2q21.2	132599047	dog	160334	373060
2	2q21.2	132972107	2q23.3	153457546	dog	20485439	204019
2	2q23.3	153661565	2q32.2	190041649	dog	36380084	90013
2	2q32.2	190131662	2q36.2	225096837	dog	34965175	521
2	2q36.2	225097358	2q37.3	242478402	dog	17381044	
3	3p26	35001	3p25.2	12872767	dog	12837766	36806
3	3p25.2	12909573	3p25.1	15138279	dog	2228706	24660
3	3p25.1	15162939	3p23	32673929	dog	17510990	1415
3	3p23	32675344	3p21.3	44600317	dog	11924973	119075
3	3p21.3	44719392	3p12.3	75411831	dog	30692439	0

3	3p12.3	75411831	3p12.3	75722985	dog	311154	221937
3	3p12.3	75944922	3p12.2	80346955	dog	4402033	51067370
3	3q21.3	131414325	3q25.32	159979299	dog	28564974	12
3	3q25.32	159979311	3q26.32	180198708	dog	20219397	15698
3	3q26.32	180214406	3q29	194780147	dog	14565741	180
3	3q29	194780327	3q29	195495418	dog	715091	43511
3	3q29	195538929	3q29	199260422	dog	3721493	
4	4p16.3	482856	4p16.3	1231195	dog	748339	41492
4	4p16.3	1272687	4p16.3	3919912	dog	2647225	135125
4	4p16.3	4055037	4p16.3	4238573	dog	183536	63676
4	4p16.3	4302249	4p16.1	6333095	dog	2030846	106714
4	4p16.1	6439809	4p16.1	8850821	dog	2411012	348513
4	4p16.1	9199334	4p16.1	9388939	dog	189605	52072
4	4p16.1	9441011	4p15.3	17436502	dog	7995491	1
4	4p15.3	17436503	4p13	41164479	dog	23727976	1940
4	4p13	41166419	4p12	48924940	dog	7758521	3604885
4	4q12	52529825	4q13.3	75714013	dog	23184188	269563
4	4q13.3	75983576	4q21.1	76653750	dog	670174	98145
4	4q21.1	76751895	4q27	121350309	dog	44598414	324
4	4q27	121350633	4q31.21	144761733	dog	23411100	24382
4	4q31.21	144786115	4q32.3	169618366	dog	24832251	105297
4	4q32.3	169723663	4q34.2	176804918	dog	7081255	12029
4	4q34.2	176816947	4q35.2	191268547	dog	14451600	
5	5p15.3	134661	15p15.2	14749628	dog	14614967	199695
5	15p15.2	14949323	5p12	45939847	dog	30990524	3665710
5	5q11.2	49605557	5q11.2	54374852	dog	4769295	93251539
5	5q32	147626391	5q35.3	176931766	dog	29305375	491250
5	5q35.3	177423016	5q35.3	180031037	dog	2608021	
6	6p25.3	126289	6p22.1	29757150	dog	29630861	372371
6	6p21.33	30129521	6p21.33	30289669	dog	160148	110234
6	6p21.33	30399903	6p11.2	58195618	dog	27795715	3829438
6	6q12	62025056	6q22.3	116717761	dog	54692705	850
6	6q22.3	116718611	6q23.2	131729655	dog	15011044	166104
6	6q23.2	131895759	6q23.2	132113394	dog	217635	1
6	6q23.2	132113395	6q23.2	132280804	dog	167409	4831
6	6q23.2	132285635	6q27	169912904	dog	37627269	7215
6	6q27	169920119	6q27	170811514	dog	891395	
7	7p22	1122707	7p22	6520044	dog	5397337	293054
7	7p22	6813098	7p14.3	37558287	dog	30745189	70216477
7	7q31.1	107774764	7q31.32	122749795	dog	14975031	20421
7	7q31.32	122770216	7q33	133750212	dog	10979996	7535
7	7q33	133757747	7q34	137385080	dog	3627333	183249
7	7q34	137568329	7q36.1	148170205	dog	10601876	27876
7	7q36.1	148198081	7q36.3	158618905	dog	10420824	
8	8p23.3	171204	8p23.3	458855	dog	287651	13919
8	8p23.3	472774	8p23.3	1736205	dog	1263431	368003
8	8p23.3	2104208	8p23.1	7404055	dog	5299847	736722
8	8p23.1	8140777	8p23.1	9690212	dog	1549435	19552028
8	8p12	29242240	8p11.2	43178734	dog	13936494	5154974
8	8q11.2	48333708	8q11.2	48819182	dog	485474	27873
8	8q11.2	48847055	8q22.1	98817981	dog	49970926	13954
8	8q22.1	98831935	8q24.3	146199185	dog	47367250	
9	9p24	41356	9p24	5843772	dog	5802416	33936
9	9p24	5877708	9p13	38462144	dog	32584436	58655001
9	9q22.3	97117145	9q33.2	121295766	dog	24178621	2
9	9q33.2	121295768	9q34.3	138294946	dog	16999178	
10	10p15	12916	10p12.3	19023073	dog	19010157	8411949
10	10p12.1	27435022	10p12.1	27658010	dog	222988	2118475

10	10p11.23	29776485	10p11.21	37137798	dog	7361313	91974
10	10p11.21	37229772	10p11.21	37442197	dog	212425	12634
10	10p11.21	37454831	10p11.21	37575497	dog	120666	574616
10	10p11.21	38150113	10p11.21	38450766	dog	300653	109704
10	10p11.21	38560470	10p11.21	38677520	dog	117050	3906045
10	10q11.21	42583565	10q11.22	46102264	dog	3518699	181255
10	10q11.22	46283519	10q11.22	46594244	dog	310725	1343586
10	10q11.22	47937830	10q11.22	48232895	dog	295065	107062
10	10q11.22	48339957	10q11.23	50801571	dog	2461614	40320253
10	10q23.3	91121824	10q26.3	132784264	dog	41662440	
11	11p15.5	182148	11p15.5	783824	dog	601676	3330
11	11p15.5	787154	11p15.4	3226658	dog	2439504	152670
11	11p15.4	3379328	11p15.4	3569927	dog	190599	12165
11	11p15.4	3582092	11p14	30237414	dog	26655322	10245
11	11p14	30247659	11p11.2	44035700	dog	13788041	712
11	11p11.2	44036412	11p11.2	48505093	dog	4468681	6621097
11	11q12.1	55126190	11q12.1	59275943	dog	4149753	15378
11	11q12.1	59291321	11q12.2	60003192	dog	711871	33477
11	11q12.2	60036669	11q13.4	70893857	dog	10857188	123395
11	11q13.4	71017252	11q13.4	71193468	dog	176216	110823
11	11q13.4	71304291	11q22.1	100814003	dog	29509712	13811
11	11q22.1	100827814	11q25	134394148	dog	33566334	
12	12p13.3	17706	12p13.3	2723684	dog	2705978	6696
12	12p13.3	2730380	12q13.2	54012775	dog	51282395	274988
12	12q13.2	54287763	12q21.1	74177243	dog	19889480	1
12	12q21.1	74177244	12q23.3	103086679	dog	28909435	16244
12	12q23.3	103102923	12q23.3	106684856	dog	3581933	129231
12	12q23.3	106814087	12q24.11	108949566	dog	2135479	1925
12	12q24.11	108951491	12q24.33	132389811	dog	23438320	
13	13q12.11	18820724	13q14.11	40332943	dog	21512219	32300
13	13q14.11	40365243	13q14.3	51584594	dog	11219351	84623
13	13q14.3	51669217	13q14.3	52111818	dog	442601	1486
13	13q14.3	52113304	13q34	114112545	dog	61999241	
14	14q11.2	19712896	14q11.2	21253497	dog	1540601	142421
14	14q11.2	21395918	14q32.3	105451370	dog	84055452	
15	15q11.2	19763721	15q11.2	19915888	dog	152167	468909
15	15q11.2	20384797	15q11.2	20945083	dog	560286	400571
15	15q11.2	21345654	15q13.1	26256384	dog	4910730	361826
15	15q11.2	26618210	15q13.1	26726450	dog	108240	270299
15	15q13.1	26996749	15q13.3	30410087	dog	3413338	279638
15	15q13.1	30689725	15q24.2	73815789	dog	43126064	97551
15	15q13.3	73913340	15q24.3	75985591	dog	2072251	64226
15	15q24.2	76049817	15q25.1	76479556	dog	429739	34035
15	15q24.3	76513591	15q25.1	76788398	dog	274807	42384
15	15q25.1	76830782	15q25.2	80366675	dog	3535893	8293631
15	15q25.1	88660306	15q26.1	89369223	dog	708917	23782
15	15q26.1	89393005	15q26.3	100084049	dog	10691044	74743
15	15q26.1	100158792	15q26.3	100307935	dog	149143	
16	16p13.3	15856	16p11.2	28521388	dog	28505532	100020
16	16p11.2	28621408	16p11.2	28953950	dog	332542	73013
16	16p11.2	29026963	16p11.2	29252547	dog	225584	308793
16	16p11.2	29561340	16p11.2	31448482	dog	1887142	13721306
16	16q11.2	45169788	16q12.1	45539453	dog	369665	4553
16	16q12.1	45544006	16q21	57366106	dog	11822100	40545
16	16q21	57406651	16q22.1	68534470	dog	11127819	4401401
16	16q22.1	72935871	16q24.3	88663224	dog	15727353	
17	17p13.3	2	17p13.3	1184941	dog	1184939	6181
17	17p13.3	1191122	17p13.2	3767755	dog	2576633	85992

17	17p13.2	3853747	17p12	15568027	dog	11714280	5243527
17	17p11.2	20811554	17p11.2	21281418	dog	469864	1268298
17	17q11.2	22549716	17q12	31512484	dog	8962768	2092523
17	17q12	33605007	17q21.32	42484284	dog	8879277	15246327
17	17q23.3	57730611	17q24.1	60190842	dog	2460231	3536159
17	17q24.2	63727001	17q24.3	65862760	dog	2135759	23233
17	17q24.3	65885993	17q25.3	77869187	dog	11983194	
18	18p11.32	102544	18p11.21	13122946	dog	13020402	30951
18	18p11.21	13153897	18p11.21	14020900	dog	867003	986316
18	18p11.21	15007216	18p11.21	15152965	dog	145749	1621374
18	18q11.2	16774339	18q21.1	43823147	dog	27048808	1
18	18q21.1	43823148	18q21.1	46143061	dog	2319913	417467
18	18q21.1	46560528	18q23	76109129	dog	29548601	
19	19p13.3	228639	19p13.11	19638900	dog	19410261	13707199
19	19q12	33346099	19q13.43	63788597	dog	30442498	
20	20p13	15908	20p11.2	23470914	dog	23455006	408077
20	20p11.2	23878991	20p11.2	25553589	dog	1674598	3743412
20	20q11.2	29297001	20q13.3	62378111	dog	33081110	
21	21q11.2	13449523	21q11.2	13636377	dog	186854	764242
21	21q11.2	14400619	21q22.3	45632833	dog	31232214	
22	22q11.21	15920299	22q11.21	17035708	dog	1115409	349053
22	22q11.21	17384761	22q11.21	18719485	dog	1334724	3536368
22	22q11.21	22255853	22q11.21	23363676	dog	1107823	180810
22	22q11.21	23544486	22q12.3	30977686	dog	7433200	130187
22	22q12.3	31107873	22q13.33	49510847	dog	18402974	
X	Xp22.33	368307	Xp11.22	56727470	dog	56359163	5827587
X	Xq11.2	62555057	Xq11.2	63440737	dog	885680	475973
X	Xq11.2	63916710	Xq21.3	86731882	dog	22815172	3579966
X	Xq21.3	90311848	Xq28	154807256	dog	64495408	
1	1p36.2	10068551	1p35	32572749	horse	22504198	53890126
1	1p22	86462875	1q21	152049085	horse	65586210	18404729
1	1q25	170453814	1q31	183169411	horse	12715597	20414952
1	1q32	203584363	1q32	203589226	horse	4863	11533901
1	1q41	215123127	1q41	215219029	horse	95902	25999442
1	1q44	241218471	1q44	241229338	horse	10867	
2	2p25.1	10585428	2q24.2	119512573	horse	108927145	8568380
2	2q14.3	128080953	2q33	204564411	horse	76483458	5844014
2	2q34	210408425	2q37.1	233374445	horse	22966020	
3	3p24	25318809	3p21.2	52452269	horse	27133460	50197251
3	3q12	102649520	3q12	102677146	horse	27626	23053739
3	3q21	125730885	3q23	143000800	horse	17269915	7172192
3	3q24	150172992	3q25	153458727	horse	3285735	17627898
3	3q26.2	171086625	3q26.2	171114157	horse	27532	16723161
3	3q27	187837318	3q27	188790133	horse	952815	
4	4p14	41108850	4q23	100666682	horse	59557832	23099442
4	4q27	123766124	4q31.2	144940754	horse	21174630	11050720
4	4q31.3	155991474	4q31.3	155999990	horse	8516	31771867
4	4q35	187771857	4q35	187794384	horse	22527	
5	5p15.1	17250628	5q12	64853897	horse	47603269	9165843
5	5q13.3	74019740	5q35	179222232	horse	105202492	
6	6p25	6134315	6p24	12359335	horse	6225020	20251584
6	6p21.3	32610919	6q13	70962931	horse	38352012	12908481
6	6q14	83871412	6q15	90534306	horse	6662894	20661883
6	6q21	111196189	6q23.3	137170189	horse	25974000	12855833
6	6q25.1	150026022	6q26	161007766	horse	10981744	
7	7p14	37906259	7p13	43935802	horse	6029543	20887689
7	7q11.21	64823491	7q11.23	72881325	horse	8057834	3883768
7	7q11.23	76765093	7q21.1	80146916	horse	3381823	13506426

7	7q21.3	93653342	7q36	154560138	horse	60906796	
8	8p21	19606081	8p21	23361090	horse	3755009	3914964
8	8p21	27276054	8p21	27292752	horse	16698	74587198
8	8q22.2	101879950	8q24.2	133818041	horse	31938091	
9	9p24	2612134	9p23	12700257	horse	10088123	8366848
9	9p21	21067105	9p13	34627730	horse	13560625	34457977
9	9q21.1	69085707	9q21.2	75910531	horse	6824824	8685950
9	9q21.3	84596481	9q21.3	84645218	horse	48737	21818531
9	9q31	106463749	9q33	127083179	horse	20619430	
10	10p15	5236818	10p15	5250109	horse	13291	12171427
10	10p12.3	17421536	10p12.3	17429831	horse	8295	10204553
10	10p12.1	27634384	10p12.1	27657250	horse	22866	15713960
10	10q11.2	43371210	10q22.2	75569689	horse	32198479	56294597
10	10q26.3	131864286	10q26.3	131912390	horse	48104	
11	11p15.5	2113209	11p15.5	2119251	horse	6042	3086512
11	11p15.4	5205763	11p13	31871919	horse	26666156	28235152
11	11q12	60107071	11q13.2	67303886	horse	7196815	33640038
11	11q22.1	100943924	11q23	119327766	horse	18383842	
12	12p13.3	5937407	12q13.1	48256634	horse	42319227	7808001
12	12q13.3	56064635	12q15	68270233	horse	12205598	20553311
12	12q21.3	88823544	12q23	102807288	horse	13983744	6042015
12	12q24.1	108849303	12q24.31	122819896	horse	13970593	10662282
12	12q24.33	133482178	12q24.33	133514783	horse	32605	
13	13q12.1	19462922	13q14.1	39302438	horse	19839516	1269894
13	13q14.1	40572332	13q14.3	51961371	horse	11389039	5181209
13	13q21.1	57142580	13q34	114059928	horse	56917348	
14	14q11.2	18927692	14q11.2	22967584	horse	4039892	37184951
14	14q23	60152535	14q32.1	92844981	horse	32692446	
15	15q13	27572439	15q13	27694790	horse	122351	22489657
15	15q21.1	50184447	15q24	72624862	horse	22440415	16374255
15	15q26.1	88999117	15q26.3	97094240	horse	8095123	
16	16p13.3	166984	16p13.1	11341617	horse	11174633	35893932
16	16q12.1	47235549	16q24	89688683	horse	42453134	
17	17p13	854103	17p13	9751740	horse	8897637	19520520
17	17q12	29272260	17q12	30546178	horse	1273918	15082665
17	17q21.3	45628843	17q21.3	45643124	horse	14281	14721779
17	17q24	60364903	17q24	62391090	horse	2026187	522189
17	17q24	62913279	17q25	80590141	horse	17676862	
18	18p11.32	647622	18p11.2	9944825	horse	9297203	11212935
18	18q11.2	21157760	18q12.1	28569959	horse	7412199	15999449
18	18q21.1	44569408	18q21.3	55074637	horse	10505229	19650849
18	18q23	74725486	18q23	74743843	horse	18357	
19	19p13.3	6617877	19p13.2	11088048	horse	4470171	27287570
19	19q13.1	38375618	19q13.3	50502183	horse	12126565	
20	20p13	1297622	20p11.2	23350093	horse	22052471	9314345
20	20q11.2	32664438	20q13.3	62172325	horse	29507887	
21	21q22.1	31105460	21q22.3	43486924	horse	12381464	
22	22q11.2	20246572	22q12.2	30000274	horse	9753702	1579608
22	22q12.3	31579882	22q13.3	49198966	horse	17619084	
X	Xp22.33	1291158	Xq23	113607456	horse	112316298	1399757
X	Xq23	115007213	Xq24	117578597	horse	2571384	2691761
X	Xq24	120270358	Xq28	152547026	horse	32276668	
1	1p36.3	1008947	1p32	58369551	mouse	57360604	108508
1	1p32	58478059	1p31.2	66969862	mouse	8491803	978120
1	1p31.2	67947982	1p22	89101147	mouse	21153165	200
1	1p22	89101347	1p22	93175140	mouse	4073793	87730
1	1p22	93262870	1q23	154874654	mouse	61611784	425434
1	1q23	155300088	1q32	204164021	mouse	48863933	55557

1	1q32	204219578	1q42	220829162	mouse	16609584	200
1	1q42	220829362	1q42	222368518	mouse	1539156	49188
1	1q42	222417706	1q42	224030267	mouse	1612561	1205385
1	1q42	225235652	1q42	231615019	mouse	6379367	20039
1	1q42	231635058	1q43	236366875	mouse	4731817	151015
1	1q43	236517890	1q44	243221610	mouse	6703720	
2	2p25.3	23769	2p25.3	1581123	mouse	1557354	144319
2	2p25.3	1725442	2p25.3	3119186	mouse	1393744	115846
2	2p25.3	3235032	2p23	26308539	mouse	23073507	45255
2	2p23	26353794	2p23	28962129	mouse	2608335	31802
2	2p23	28993931	2p16	53300424	mouse	24306493	545354
2	2p16	53845778	2p13	68650089	mouse	14804311	2450
2	2p13	68652539	2p13	70975429	mouse	2322890	14735
2	2p13	70990164	2p13	74026347	mouse	3036183	200
2	2p13	74026547	2p11.2	87042812	mouse	13016265	9589141
2	2q11.2	96631953	2q12	106560466	mouse	9928513	428319
2	2q12	106988785	2q12	108672097	mouse	1683312	1747870
2	2q13	110419967	2q13	113554679	mouse	3134712	790503
2	2q14.1	114345182	2q14.3	122483460	mouse	8138278	1393544
2	2q14.3	123877004	2q14.3	124910919	mouse	1033915	2210776
2	2q14.3	127121695	2q14.3	128690762	mouse	1569067	35308
2	2q14.3	128726070	2q21.1	131913009	mouse	3186939	1248115
2	2q21.2	133161124	2q22	138624340	mouse	5463216	1330995
2	2q22	139955335	2q32.1	188356222	mouse	48400887	189960
2	2q32.1	188546182	2q32.2	190478738	mouse	1932556	200
2	2q32.2	190478938	2q32.3	196737493	mouse	6258555	470
2	2q32.3	196737963	2q37.3	242764207	mouse	46026244	
3	3p26	78258	3p25	12864927	mouse	12786669	70612
3	3p25	12935539	3p25	15135116	mouse	2199577	1166897
3	3p25	16302013	3p24	20077784	mouse	3775771	2919228
3	3p24	22997012	3p24	27550695	mouse	4553683	54777
3	3p24	27605472	3p22	37067985	mouse	9462513	14968
3	3p22	37082953	3p21.3	46264937	mouse	9181984	5941232
3	3p21.2	52206169	3p14.3	57784230	mouse	5578061	16285
3	3p14.3	57800515	3p14.1	63864162	mouse	6063647	8531
3	3p14.1	63872693	3p12	75092934	mouse	11220241	640629
3	3p12	75733563	3q21	126621496	mouse	50887933	394445
3	3q21	127015941	3q21	130317226	mouse	3301285	896037
3	3q21	131213263	3q24	149369030	mouse	18155767	986607
3	3q24	150355637	3q26.1	169224070	mouse	18868433	74746
3	3q26.2	169298816	3q26.2	171956943	mouse	2658127	200
3	3q26.2	171957143	3q26.3	179505847	mouse	7548704	4206
3	3q26.3	179510053	3q27	184220160	mouse	4710107	23408
3	3q26.3	184243568	3q29	196687900	mouse	12444332	148996
3	3q29	196836896	3q29	198725190	mouse	1888294	
4	4p16	1151072	4p16	3792758	mouse	2641686	405599
4	4p16	4198357	4p16	8859586	mouse	4661229	615355
4	4p16	9474941	4q22	89368712	mouse	79893771	200
4	4q22	89368912	4q22	95665419	mouse	6296507	21066
4	4q22	95686485	4q26	121086919	mouse	25400434	245410
4	4q27	121332329	4q27	122734117	mouse	1401788	200
4	4q27	122734317	4q31.1	141656124	mouse	18921807	65613
4	4q31.1	141721737	4q31.2	151341774	mouse	9620037	90922
4	4q31.2	151432696	4q32	163691876	mouse	12259180	279671
4	4q32	163971547	4q35	191462724	mouse	27491177	
5	5p15.3	224239	5p15.3	8302649	mouse	8078410	200
5	5p15.3	8302849	5p12	42875358	mouse	34572509	611682
5	5p12	43487040	5q15	96161775	mouse	52674735	71680

5	5q15	96233455	5q21	98428174	mouse	2194719	200
5	5q21	98428374	5q21	101907132	mouse	3478758	883494
5	5q21	102790626	5q22	110093254	mouse	7302628	236320
5	5q22	110329574	5q22	112320424	mouse	1990850	200
5	5q22	112320624	5q23.3	130393506	mouse	18072882	121559
5	5q23.3	130515065	5q31.1	134084748	mouse	3569683	20422
5	5q31.1	134105170	5q31.1	137121370	mouse	3016200	135591
5	5q31.1	137256961	5q32	147606363	mouse	10349402	24949
5	5q32	147631312	5q33.1	150267493	mouse	2636181	101395
5	5q33.1	150368888	5q33.2	154303864	mouse	3934976	6205
5	5q33.2	154310069	5q35	171867674	mouse	17557605	1830752
5	5q35	173698426	5q35	177106680	mouse	3408254	377237
5	5q35	177483917	5q35	180744042	mouse	3260125	
6	6p25	171287	6p22.3	20123166	mouse	19951879	4996
6	6p22.3	20128162	6p22.1	29077605	mouse	8949443	63303
6	6p22.1	29140908	6p21.3	33298631	mouse	4157723	62324
6	6p21.3	33360955	6p21.2	39059211	mouse	5698256	220851
6	6p21.2	39280062	6p12	49700593	mouse	10420531	103832
6	6p12	49804425	6p12	52714965	mouse	2910540	10282
6	6p12	52725247	6p12	55784269	mouse	3059022	441631
6	6p12	56225900	6q13	73858776	mouse	17632876	154406
6	6q13	74013182	6q14	86338899	mouse	12325717	1406474
6	6q14	87745373	6q16.3	100244557	mouse	12499184	290710
6	6q16.3	100535267	6q22.1	116988488	mouse	16453221	60550
6	6q22.1	117049038	6q22.3	123084295	mouse	6035257	50712
6	6q22.3	123135007	6q25.1	150140235	mouse	27005228	61007
6	6q25.1	150201242	6q25.2	154942085	mouse	4740843	67467
6	6q25.2	155009552	6q25.3	158794650	mouse	3785098	1142933
6	6q25.3	159937583	6q27	167369970	mouse	7432387	391160
6	6q27	167761130	6q27	170547318	mouse	2786188	
7	7p22	176757	7p22	640366	mouse	463609	6113567
7	7p22	6753933	7p21	12241969	mouse	5488036	30502
7	7p21	12272471	7p21	19456148	mouse	7183677	7609
7	7p21	19463757	7p15.3	22236982	mouse	2773225	726976
7	7p15.3	22963958	7p14	32800883	mouse	9836925	77263
7	7p14	32878146	7p14	36045970	mouse	3167824	185192
7	7p14	36231162	7p13	43312709	mouse	7081547	308729
7	7p13	43621438	7p11.2	55012415	mouse	11390977	11193852
7	7q11.21	66206267	7q11.23	71582029	mouse	5375762	546803
7	7q11.23	72128832	7q11.23	73887786	mouse	1758954	2420521
7	7q11.23	76308307	7q21.1	84871677	mouse	8563370	60858
7	7q21.1	84932535	7q21.3	92243945	mouse	7311410	90100
7	7q21.2	92334045	7q21.3	97098638	mouse	4764593	106262
7	7q21.3	97204900	7q22	98816819	mouse	1611919	306844
7	7q22	99123663	7q22	101660815	mouse	2537152	218131
7	7q22	101878946	7q22	104742436	mouse	2863490	12403
7	7q22	104754839	7q31.1	107320074	mouse	2565235	3227
7	7q31.1	107323301	7q31.1	111682409	mouse	4359108	3780
7	7q31.1	111686189	7q36	149864544	mouse	38178355	38834
7	7q36	149903378	7q36	156511361	mouse	6607983	20564
7	7q36	156531925	7q36	158238121	mouse	1706196	
8	8p23.3	553737	8p23.1	7056332	mouse	6502595	925656
8	8p23.1	7981988	8p23.1	9510886	mouse	1528898	68406
8	8p23.1	9579292	8p23.1	11711299	mouse	2132007	642961
8	8p23.1	12354260	8p22	17763961	mouse	5409701	38349
8	8p22	17802310	8p21	19985868	mouse	2183558	22416
8	8p21	20008284	8p12	28970491	mouse	8962207	40586
8	8p12	29011077	8p12	36514720	mouse	7503643	200

8	8p12	36514920	8p12	37697674	mouse	1182754	6312
8	8p12	37703986	8p11.2	42246576	mouse	4542590	5669032
8	8q11.2	47915608	8q11.2	49588255	mouse	1672647	809064
8	8q11.2	50397319	8q12	56285211	mouse	5887892	101410
8	8q12	56386621	8q12	62430547	mouse	6043926	200
8	8q12	62430747	8q12	63737128	mouse	1306381	62124
8	8q12	63799252	8q13	67059557	mouse	3260305	15017
8	8q13	67074574	8q21.1	75849464	mouse	8774890	72224
8	8q21.1	75921688	8q21.2	86722830	mouse	10801142	2352
8	8q21.2	86725182	8q22.1	96908031	mouse	10182849	174801
8	8q22.1	97082832	8q24.3	145883574	mouse	48800742	
9	9p24	100687	9p24	6655150	mouse	6554463	77984
9	9p24	6733134	9p21	27287159	mouse	20554025	28023
9	9p21	27315182	9p13	38461949	mouse	11146767	25095711
9	9q13	63557660	9q21.2	76348435	mouse	12790775	217695
9	9q21.2	76566130	9q21.3	79707435	mouse	3141305	80230
9	9q21.3	79787665	9q21.3	83878430	mouse	4090765	455066
9	9q21.3	84333496	9q22.1	88142795	mouse	3809299	184093
9	9q22.1	88326888	9q22.2	90365482	mouse	2038594	290090
9	9q22.2	90655572	9q22.3	93266234	mouse	2610662	230054
9	9q22.3	93496288	9q33	116942209	mouse	23445921	51576
9	9q33	116993785	9q33	121409752	mouse	4415967	200
9	9q33	121409952	9q33	124499927	mouse	3089975	200
9	9q33	124500127	9q34.1	127752163	mouse	3252036	23693
9	9q34.1	127775856	9q34.1	129390603	mouse	1614747	95857
9	9q34.1	129486460	9q34.1	131919071	mouse	2432611	22363
9	9q34.1	131941434	9q34.2	134321460	mouse	2380026	
10	10p15	128739	10p15	6009060	mouse	5880321	56761
10	10p15	6065821	10p13	15603605	mouse	9537784	21514
10	10p13	15625119	10p12.1	27681482	mouse	12056363	19708
10	10p12.1	27701190	10p12.1	29062204	mouse	1361014	38912
10	10p12.1	29101116	10p11.2	33217351	mouse	4116235	200
10	10p11.2	33217551	10p11.2	35326646	mouse	2109095	449121
10	10p11.2	35775767	10p11.2	37242075	mouse	1466308	5826923
10	10q11.2	43068998	10q11.2	45932301	mouse	2863303	871195
10	10q11.2	46803496	10q11.2	51487686	mouse	4684190	407430
10	10q11.2	51895116	10q21.1	54461632	mouse	2566516	589271
10	10q21.1	55050903	10q22.2	74749467	mouse	19698564	13121
10	10q22.2	74762588	10q22.3	81955250	mouse	7192662	200
10	10q22.3	81955450	10q23.2	89096592	mouse	7141142	294790
10	10q23.3	89391382	10q26.1	121315237	mouse	31923855	200
10	10q26.1	121315437	10q26.3	135288586	mouse	13973149	
11	11p15.5	1206762	11p15.5	3213050	mouse	2006288	377291
11	11p15.4	3590341	11p15.1	17495811	mouse	13905470	200
11	11p15.1	17496011	11p14	25978536	mouse	8482525	270987
11	11p14	26249523	11q12	58001448	mouse	31751925	443508
11	11q12	58444956	11q13.3	63137398	mouse	4692442	1311577
11	11q13.1	64448975	11q13.3	68957204	mouse	4508229	62693
11	11q13.3	69019897	11q13.4	71432282	mouse	2412385	417529
11	11q13.4	71849811	11q14.3	89511911	mouse	17662100	535522
11	11q14.3	90047433	11q22.3	107468989	mouse	17421556	17954
11	11q22.3	107486943	11q25	134965945	mouse	27479002	
12	12p13.3	61014	12p13.3	2682266	mouse	2621252	102138
12	12p13.3	2784404	12p13.3	9184429	mouse	6400025	96939
12	12p13.3	9281368	12p11.2	32437652	mouse	23156284	5891021
12	12p12	38328673	12q13.2	54789653	mouse	16460980	283668
12	12q13.3	55073321	12q23	104257973	mouse	49184652	34416
12	12q23	104292389	12q24.1	108109684	mouse	3817295	148753

12	12q24.1	108258437	12q24.1	110367340	mouse	2108903	20755
12	12q24.1	110388095	12q24.31	121280245	mouse	10892150	60116
12	12q24.31	121340361	12q24.33	132216098	mouse	10875737	
13	13q12.1	18871102	13q12.1	22293650	mouse	3422548	3421521
13	13q12.1	25715171	13q13	33271776	mouse	7556605	152978
13	13q13	33424754	13q14.1	40183356	mouse	6758602	259477
13	13q14.1	40442833	13q14.2	48735681	mouse	8292848	405181
13	13q14.2	49140862	13q14.3	51291453	mouse	2150591	391933
13	13q14.3	51683386	13q33	101982034	mouse	50298648	391134
13	13q33	102373168	13q34	114114558	mouse	11741390	
14	14q11.2	18231817	14q11.2	23133159	mouse	4901342	15235
14	14q11.2	23148394	14q22	50233231	mouse	27084837	445779
14	14q21	50679010	14q22	56613453	mouse	5934443	43508
14	14q22	56656961	14q32.3	104347485	mouse	47690524	
15	15q11.2	22618175	15q13	25992349	mouse	3374174	835400
15	15q13	26827749	15q13	27946389	mouse	1118640	830458
15	15q13	28776847	15q13	30021254	mouse	1244407	491963
15	15q13	30513217	15q14	32238453	mouse	1725236	17305510
15	15q21.1	49543963	15q24	73595971	mouse	24052008	82158
15	15q24	73678129	15q24	75746357	mouse	2068228	122429
15	15q24	75868786	15q25	77705431	mouse	1836645	140861
15	15q25	77846292	15q25	80141631	mouse	2295339	657967
15	15q25	80799598	15q25	82356077	mouse	1556479	1090247
15	15q25	83446324	15q25	89148393	mouse	5702069	43304
15	15q26.1	89191697	15q26.3	99857344	mouse	10665647	
16	16p13.3	179003	16p13.3	3238113	mouse	3059110	46692
16	16p13.3	3284805	16p12	16153065	mouse	12868260	505005
16	16p13.1	16658070	16p12	18128289	mouse	1470219	523976
16	16p12	18652265	16p11.2	31516640	mouse	12864375	14917245
16	16q12.1	46433885	16q22	69711086	mouse	23277201	211619
16	16q22	69922705	16q23	74093638	mouse	4170933	131890
16	16q23	74225528	16q24	89810999	mouse	15585471	
17	17p13	18172	17p13	4482673	mouse	4464501	42820
17	17p13	4525493	17p13	6691646	mouse	2166153	101837
17	17p13	6793483	17p11.2	16499527	mouse	9706044	368084
17	17p11.2	16867611	17p11.2	20762124	mouse	3894513	4653638
17	17q11.2	25415762	17q11.2	28705665	mouse	3289903	205653
17	17q12	28911318	17q12	34312844	mouse	5401526	256392
17	17q12	34569236	17q21.2	35922531	mouse	1353295	399409
17	17q21.2	36321940	17q21.3	45860244	mouse	9538304	155516
17	17q21.3	46015760	17q23	58383936	mouse	12368176	164268
17	17q23	58548204	17q24	60630038	mouse	2081834	88265
17	17q24	60718303	17q24	63138272	mouse	2419969	233143
17	17q24	63371415	17q24	66677334	mouse	3305919	21152
17	17q24	66698486	17q25	81588123	mouse	14889637	
18	18p11.32	856048	18p11.32	2458344	mouse	1602296	74994
18	18p11.32	2533338	18p11.2	9962250	mouse	7428912	1619242
18	18p11.2	11581492	18p11.2	13926692	mouse	2345200	4501844
18	18q11.2	18428536	18q12.3	40945714	mouse	22517178	299289
18	18q12.3	41245003	18q21.3	54027458	mouse	12782455	26311
18	18q21.3	54053769	18q21.3	58048268	mouse	3994499	72662
18	18q21.3	58120930	18q22	65726427	mouse	7605497	401440
18	18q22	66127867	18q23	77740271	mouse	11612404	
19	19p13.3	221385	19p13.3	4164504	mouse	3943119	6694
19	19p13.3	4171198	19p13.2	7004895	mouse	2833697	47367
19	19p13.2	7052262	19p13.2	8217865	mouse	1165603	546352
19	19p13.2	8764217	19p13.2	12423093	mouse	3658876	179562
19	19p13.2	12602655	19p13.1	14516393	mouse	1913738	2498424

19	19p13.1	17014817	19p13.1	19619771	mouse	2604954	13646311
19	19q12	33266082	19q12	34763281	mouse	1497199	12244
19	19q12	34775525	19q13.3	53019374	mouse	18243849	456871
19	19q13.3	53476245	19q13.4	56776884	mouse	3300639	2476303
19	19q13.4	59253187	19q13.4	60885084	mouse	1631897	
20	20p13	16324	20p13	1390878	mouse	1374554	304604
20	20p13	1695482	20p11.2	24665495	mouse	22970013	5877883
20	20q11.2	30543378	20q13.3	63578987	mouse	33035609	
21	21q11.2	14438806	21q22.3	42332141	mouse	27893335	53407
21	21q22.3	42385548	21q22.3	44025134	mouse	1639586	200
21	21q22.3	44025334	21q22.3	46940887	mouse	2915553	
22	22q11.2	17713396	22q11.2	20662815	mouse	2949419	502162
22	22q11.2	21164977	22q11.2	23328753	mouse	2163776	198190
22	22q11.2	23526943	22q12.1	27480781	mouse	3953838	12363
22	22q12.1	27493144	22q12.2	30335430	mouse	2842286	1655212
22	22q12.3	31990642	22q12.3	34221854	mouse	2231212	18148
22	22q12.3	34240002	22q13.3	49316371	mouse	15076369	
X	Xp22.2	9762041	Xp22.11	23051645	mouse	13289604	998
X	Xp22.11	23052643	Xp21.1	36457204	mouse	13404561	200
X	Xp21.1	36457404	Xp11.3	46711758	mouse	10254354	67110
X	Xp11.3	46778868	Xp11.23	48895270	mouse	2116402	137
X	Xp11.23	48895407	Xp11.22	50082060	mouse	1186653	1677593
X	Xp11.22	51759653	Xp11.22	53792537	mouse	2032884	7007648
X	Xq11.2	60800185	Xq21.1	81290506	mouse	20490321	35817
X	Xq21.1	81326323	Xq21.31	91417469	mouse	10091146	372303
X	Xq21.31	91789772	Xq23	112595429	mouse	20805657	539446
X	Xq23	113134875	Xq28	152009549	mouse	38874674	
1	1p36.3	2916481	1p34.2	40197771	pig	37281290	1506067
1	1p34.2	41703838	1p31.1	83798949	pig	42095111	1450819
1	1p22	85249768	1q24	167431107	pig	82181339	1443766
1	1q24	168874873	1q25	181731277	pig	12856404	2917436
1	1q31	184648713	1q31	190994647	pig	6345934	2875682
1	1q31	193870329	1q32	198449609	pig	4579280	2303801
1	1q32	200753410	1q32	202039491	pig	1286081	2962346
1	1q32	205001837	1q32	210456462	pig	5454625	1920166
1	1q41	212376628	1q41	219283155	pig	6906527	1176861
1	1q42	220460016	1q42	220752892	pig	292876	653633
1	1q42	221406525	1q42	223968729	pig	2562204	7043506
1	1q42	231012235	1q43	236369159	pig	5356924	1596786
1	1q43	237965945	1q43	238022215	pig	56270	1568421
1	1q43	239590636	1q44	242033847	pig	2443211	
2	2p25.3	1564865	2q13	112349922	pig	110785057	2867465
2	2q14.1	115217387	2q14.2	119249570	pig	4032183	1015362
2	2q14.2	120264932	2q14.3	126294676	pig	6029744	3066395
2	2q14.3	129361071	2q21.1	129726694	pig	365623	3818018
2	2q21.2	133544712	2q23	150240956	pig	16696244	3294920
2	2q23	153535876	2q37.3	238743000	pig	85207124	
3	3p26	140869	3p25	14703453	pig	14562584	997772
3	3p25	15701225	3p12	75044362	pig	59343137	863964
3	3p12	75908326	3q21	126435252	pig	50526926	1969389
3	3q21	128404641	3q21	129808134	pig	1403493	1444709
3	3q21	131252843	3q22	133084059	pig	1831216	410493
3	3q22	133494552	3q29	198464213	pig	64969661	
4	4p16	541248	4p16	3429821	pig	2888573	1091496
4	4p16	4521317	4p16	8358037	pig	3836720	1407306
4	4p16	9765343	4q12	56080333	pig	46314990	457143
4	4q12	56537476	4q21.1	78265810	pig	21728334	3477274
4	4q21.2	81743084	4q32	156602424	pig	74859340	139828

4	4q32	156742252	4q32	156762371	pig	20119	346999
4	4q32	157109370	4q32	165895860	pig	8786490	621639
4	4q32	166517499	4q32	167376267	pig	858768	862724
4	4q32	168238991	4q34	176027692	pig	7788701	1111775
4	4q34	177139467	4q35	187764074	pig	10624607	275218
4	4q35	188039292	4q35	191457896	pig	3418604	
5	5p15.3	2463071	5p15.3	9816235	pig	7353164	1668480
5	5p15.2	11484715	5p15.1	15881962	pig	4397247	1551910
5	5p15.1	17433872	5q12	63253626	pig	45819754	1277934
5	5q13.1	64531560	5q13.2	71841995	pig	7310435	1657066
5	5q13.3	73499061	5q33.1	150116348	pig	76617287	1691586
5	5q33.1	151807934	5q35	172328339	pig	20520405	
6	6p25	706219	6p21.3	33972374	pig	33266155	3850373
6	6p21.2	37822747	6p12	51972043	pig	14149296	1912919
6	6p12	53884962	6p12	63665829	pig	9780867	1203697
6	6q12	64869526	6q13	73338720	pig	8469194	1124283
6	6q13	74463003	6q14	83886431	pig	9423428	1295429
6	6q14	85181860	6q22.1	116138228	pig	30956368	828782
6	6q22.1	116967010	6q27	168734515	pig	51767505	
7	7p22	654100	7p22	6441062	pig	5786962	1186185
7	7p21	7627247	7p15.3	23255428	pig	15628181	277956
7	7p15.3	23533384	7p14	37089533	pig	13556149	1270328
7	7p14	38359861	7p14	38359955	pig	94	2951429
7	7p14	41311384	7p12	47724972	pig	6413588	1336412
7	7p12	49061384	7p11.2	54932101	pig	5870717	255251
7	7p11.2	55187352	7p11.2	55187528	pig	176	662497
7	7p11.2	55850025	7q11.22	66101340	pig	10251315	1427104
7	7q11.22	67528444	7q11.23	71047205	pig	3518761	1200457
7	7q11.23	72247662	7q11.23	75109793	pig	2862131	1314085
7	7q11.23	76423878	7q21.1	86671814	pig	10247936	1486627
7	7q21.1	88158441	7q21.3	96965209	pig	8806768	662500
7	7q21.3	97627709	7q22	101774951	pig	4147242	1038977
7	7q22	102813928	7q31.1	107467978	pig	4654050	1447211
7	7q31.1	108915189	7q34	142089425	pig	33174236	1384967
7	7q35	143474392	7q36	148209911	pig	4735519	1764176
7	7q36	149974087	7q36	156351125	pig	6377038	
8	8p23.3	554441	8p23.1	6230255	pig	5675814	2421403
8	8p23.1	8651658	8p23.1	8651735	pig	77	1201301
8	8p23.1	9853036	8p23.1	11567139	pig	1714103	1323644
8	8p22	12890783	8p22	16731148	pig	3840365	963288
8	8p22	17694436	8p22	17694591	pig	155	652308
8	8p22	18346899	8p22	18347274	pig	375	1258970
8	8p21	19606244	8p21	28742821	pig	9136577	688660
8	8p12	29431481	8p12	38581060	pig	9149579	1461214
8	8p11.2	40042274	8p11.2	42598262	pig	2555988	5892674
8	8q11.2	48490936	8q24.3	144879682	pig	96388746	
9	9p24	234824	9p21	26715212	pig	26480388	932222
9	9p21	27647434	9p13	34121663	pig	6474229	1414657
9	9p13	35536320	9p13	37851756	pig	2315436	27042867
9	9q13	64894623	9q21.3	78377606	pig	13482983	1317748
9	9q21.3	79695354	9q21.3	82892439	pig	3197085	2355600
9	9q21.3	85248039	9q22.1	87692667	pig	2444628	646602
9	9q22.1	88339269	9q22.3	92303697	pig	3964428	1397135
9	9q22.3	93700832	9q34.1	131074882	pig	37374050	
10	10p15	1837378	10p14	10877661	pig	9040283	2389374
10	10p13	13267035	10p12.3	18876007	pig	5608972	920909
10	10p12.3	19796916	10p12.1	26608481	pig	6811565	1574560
10	10p12.1	28183041	10p11.2	32263726	pig	4080685	1259874

10	10p11.2	33523600	10p11.2	36515040	pig	2991440	8081607
10	10q11.2	44596647	10q11.2	50573505	pig	5976858	1366073
10	10q11.2	51939578	10q21.1	59933761	pig	7994183	24554789
10	10q23.1	84488550	10q23.2	88326768	pig	3838218	1143768
10	10q23.3	89470536	10q26.3	135267504	pig	45796968	
11	11p15.5	979822	11p15.5	3009963	pig	2030141	2479580
11	11p15.4	5489543	11p15.4	8973254	pig	3483711	1394336
11	11p15.4	10367590	11q13.3	70729241	pig	60361651	1425367
11	11q13.4	72154608	11q25	134342530	pig	62187922	
12	12p13.3	447518	12p11.2	33716713	pig	33269195	5463711
12	12p12	39180424	12q13.1	47699745	pig	8519321	1357736
12	12q13.1	49057481	12q21.2	76569350	pig	27511869	673241
12	12q21.2	77242591	12q23	105469295	pig	28226704	1198075
12	12q23	106667370	12q23	107089525	pig	422155	1469929
12	12q24.1	108559454	12q24.33	133008803	pig	24449349	
13	13q12.1	20644761	13q14.1	40176697	pig	19531936	921654
13	13q14.1	41098351	13q14.3	51972222	pig	10873871	658977
13	13q14.3	52631199	13q34	112986189	pig	60354990	
14	14q11.2	18460413	14q13	36324614	pig	17864201	1427185
14	14q21	37751799	14q23	61777163	pig	24025364	1153857
14	14q23	62931020	14q32.3	104256064	pig	41325044	
15	15q11.2	21326601	15q13	29402166	pig	8075565	1341827
15	15q13	30743993	15q22.3	62718616	pig	31974623	5033393
15	15q23	67752009	15q23	69197530	pig	1445521	1746717
15	15q24	70944247	15q24	74986799	pig	4042552	1469771
15	15q25	76456570	15q25	83207550	pig	6750980	1463933
15	15q25	84671483	15q25	86128267	pig	1456784	1623626
15	15q26.1	87751893	15q25	87751992	pig	99	1735910
15	15q26.1	89487902	15q26.3	96367453	pig	6879551	1210366
15	15q26.3	97577819	15q26.3	99198739	pig	1620920	
16	16p13.3	189059	16p11.2	31368021	pig	31178962	15727089
16	16q12.1	47095110	16q12.2	54868307	pig	7773197	1658646
16	16q13	56526953	16q22	68066139	pig	11539186	1216807
16	16q22	69282946	16q24	89488396	pig	20205450	
17	17p13	299201	17p11.2	17657721	pig	17358520	2892053
17	17p11.2	20549774	17p11.2	20549847	pig	73	4860204
17	17q11.2	25410051	17q21.1	35262313	pig	9852262	1129722
17	17q21.2	36392035	17q21.3	45255531	pig	8863496	1510831
17	17q21.3	46766362	17q24	60291685	pig	13525323	1395594
17	17q24	61687279	17q25	79768530	pig	18081251	
18	18p11.32	609209	18p11.2	13750204	pig	13140995	4828048
18	18q11.2	18578252	18q12.3	40509868	pig	21931616	1915618
18	18q21.1	42425486	18q21.3	54938704	pig	12513218	1332720
18	18q21.3	56271424	18q23	76537891	pig	20266467	
19	19p13.3	1698869	19p13.1	19521121	pig	17822252	13800964
19	19q12	33322085	19q13.4	61741374	pig	28419289	
20	20p13	473725	20p13	2630616	pig	2156891	2157301
20	20p13	4787917	20p11.2	25209754	pig	20421837	5731785
20	20q11.2	30941539	20q13.3	59813043	pig	28871504	
21	21q11.2	14747772	21q22.3	46764840	pig	32017068	
22	22q11.2	15004087	22q11.2	16689189	pig	1685102	737785
22	22q11.2	17426974	22q11.2	22450228	pig	5023254	1259918
22	22q11.2	23710146	22q12.2	29660438	pig	5950292	1861764
22	22q12.3	31522202	22q13.3	42802580	pig	11280378	
X	Xp22.33	6363408	Xq28	151250564	pig	144887156	
1	1p36.3	1008921	1p36.1	26032778	rat	25023857	91489
1	1p36.1	26124267	1p36.1	27828854	rat	1704587	686355
1	1p35	28515209	1p32	58369437	rat	29854228	110134

1	1p32	58479571	1p31.2	66969868	rat	8490297	978114
1	1p31.2	67947982	1p22	89073417	rat	21125435	113453
1	1p22	89186870	1p22	93175146	rat	3988276	83037
1	1p22	93258183	1q23	154930185	rat	61672002	377653
1	1q23	155307838	1q32	204176920	rat	48869082	70329
1	1q32	204247249	1q42	220819436	rat	16572187	3116
1	1q42	220822552	1q42	222372946	rat	1550394	44741
1	1q42	222417687	1q42	224015581	rat	1597894	1307467
1	1q42	225323048	1q42	231579499	rat	6256451	680683
1	1q42	232260182	1q43	236371426	rat	4111244	146508
1	1q43	236517934	1q44	243221431	rat	6703497	
2	2p25.3	1725528	2p25.3	3119186	rat	1393658	115846
2	2p25.3	3235032	2p22	35636081	rat	32401049	130407
2	2p22	35766488	2p16	52670599	rat	16904111	1176978
2	2p16	53847577	2p13	68650881	rat	14803304	1285
2	2p13	68652166	2p13	70975429	rat	2323263	29857
2	2p13	71005286	2p13	73965750	rat	2960464	48036
2	2p13	74013786	2p11.2	87050678	rat	13036892	9581163
2	2q11.2	96631841	2q12	106315238	rat	9683397	4092030
2	2q13	110407268	2q13	113552965	rat	3145697	790600
2	2q14.1	114343565	2q14.3	122430979	rat	8087414	1050505
2	2q14.3	123481484	2q14.3	124108818	rat	627334	200
2	2q14.3	124109018	2q14.3	124817492	rat	708474	386490
2	2q14.3	125203982	2q14.3	127021977	rat	1817995	69720
2	2q14.3	127091697	2q14.3	128691458	rat	1599761	34612
2	2q14.3	128726070	2q21.1	131934198	rat	3208128	1227241
2	2q21.2	133161439	2q22	138624341	rat	5462902	1331023
2	2q22	139955364	2q32.1	188356222	rat	48400858	347825
2	2q32.1	188704047	2q37.3	242775335	rat	54071288	
3	3p26	96736	3p25	12864965	rat	12768229	132443
3	3p25	12997408	3p25	15135116	rat	2137708	1722155
3	3p24	16857271	3p24	20077802	rat	3220531	1106596
3	3p24	21184398	3p24	27569115	rat	6384717	36357
3	3p24	27605472	3p22	37074253	rat	9468781	8697
3	3p22	37082950	3p21.3	46267172	rat	9184222	200
3	3p21.3	46267372	3p21.2	52076095	rat	5808723	131703
3	3p21.2	52207798	3p14.3	57767106	rat	5559308	32811
3	3p14.3	57799917	3p14.1	63864176	rat	6064259	8480
3	3p14.1	63872656	3p12	75103631	rat	11230975	577389
3	3p12	75681020	3p12	90029395	rat	14348375	5035097
3	3q11.2	95064492	3q21	126603810	rat	31539318	656424
3	3q21	127260234	3q21	130317226	rat	3056992	896011
3	3q21	131213237	3q24	149369030	rat	18155793	970250
3	3q24	150339280	3q26.1	169224071	rat	18884791	80829
3	3q26.2	169304900	3q26.2	170409406	rat	1104506	62224
3	3q26.2	170471630	3q26.3	173034682	rat	2563052	161919
3	3q26.3	173196601	3q26.3	179507657	rat	6311056	17682
3	3q26.3	179525339	3q27	184219239	rat	4693900	36278
3	3q26.3	184255517	3q29	196679158	rat	12423641	157537
3	3q29	196836695	3q29	198739716	rat	1903021	
4	4p16	1151685	4p16	3792758	rat	2641073	407714
4	4p16	4200472	4p16	8859586	rat	4659114	643131
4	4p16	9502717	4q22	89376336	rat	79873619	200
4	4q22	89376536	4q22	95664969	rat	6288433	19765
4	4q22	95684734	4q26	121086934	rat	25402200	245552
4	4q27	121332486	4q27	122837455	rat	1504969	200
4	4q27	122837655	4q31.1	141655864	rat	18818209	61932
4	4q31.1	141717796	4q31.2	151353028	rat	9635232	79895

4	4q31.2	151432923	4q32	163624597	rat	12191674	353105
4	4q32	163977702	4q35	191375700	rat	27397998	
5	5p15.3	1780926	5p15.3	7932916	rat	6151990	31041
5	5p15.3	7963957	5q15	96161776	rat	88197819	53687
5	5q15	96215463	5q21	98424895	rat	2209432	200
5	5q21	98425095	5q22	110087815	rat	11662720	242441
5	5q22	110330256	5q22	112325253	rat	1994997	14451
5	5q22	112339704	5q23.3	130393520	rat	18053816	121545
5	5q23.3	130515065	5q31.1	134075456	rat	3560391	29714
5	5q31.1	134105170	5q31.1	137121696	rat	3016526	135251
5	5q31.1	137256947	5q32	147607546	rat	10350599	23817
5	5q32	147631363	5q33.1	150210048	rat	2578685	157741
5	5q33.1	150367789	5q33.2	154317378	rat	3949589	200
5	5q33.2	154317578	5q35	173646386	rat	19328808	52040
5	5q35	173698426	5q35	177105027	rat	3406601	385429
5	5q35	177490456	5q35	180737497	rat	3247041	
6	6p25	171329	6p22.3	20093794	rat	19922465	33967
6	6p22.3	20127761	6p22.1	29101707	rat	8973946	39193
6	6p22.1	29140900	6p21.2	39059211	rat	9918311	270329
6	6p21.2	39329540	6p12	52755093	rat	13425553	19653
6	6p12	52774746	6p12	55784286	rat	3009540	442177
6	6p12	56226463	6q13	73859514	rat	17633051	153949
6	6q13	74013463	6q14	86415489	rat	12402026	1330106
6	6q14	87745595	6q16.3	100244557	rat	12498962	383869
6	6q16.3	100628426	6q22.1	116674806	rat	16046380	262895
6	6q22.1	116937701	6q22.3	123081845	rat	6144144	35626
6	6q22.3	123117471	6q22.3	127848358	rat	4730887	100538
6	6q22.3	127948896	6q23.2	135138941	rat	7190045	18172
6	6q23.2	135157113	6q25.1	150165506	rat	15008393	1129
6	6q25.1	150166635	6q27	170557912	rat	20391277	
7	7p22	150540	7p22	5502967	rat	5352427	1298498
7	7p22	6801465	7p21	12240731	rat	5439266	34202
7	7p21	12274933	7p21	19459102	rat	7184169	4658
7	7p21	19463760	7p15.3	22306265	rat	2842505	657862
7	7p15.3	22964127	7p14	32734941	rat	9770814	200
7	7p14	32735141	7p14	36046504	rat	3311363	184634
7	7p14	36231138	7p13	43312412	rat	7081274	310633
7	7p13	43623045	7p11.2	55012427	rat	11389382	11193798
7	7q11.21	66206225	7q11.23	71402623	rat	5196398	722214
7	7q11.23	72124837	7q11.23	73887786	rat	1762949	2528151
7	7q11.23	76415937	7q21.3	97029493	rat	20613556	236256
7	7q21.3	97265749	7q22	99027359	rat	1761610	2935930
7	7q22	101963289	7q22	104742436	rat	2779147	13043
7	7q22	104755479	7q31.1	107320004	rat	2564525	2856
7	7q31.1	107322860	7q31.1	111678926	rat	4356066	7417
7	7q31.1	111686343	7q36	149864544	rat	38178201	38834
7	7q36	149903378	7q36	156529297	rat	6625919	24065
7	7q36	156553362	7q36	158237917	rat	1684555	
8	8p23.3	510046	8p23.1	7500178	rat	6990132	481710
8	8p23.1	7981888	8p23.1	9510886	rat	1528998	68406
8	8p23.1	9579292	8p23.1	11711648	rat	2132356	643897
8	8p23.1	12355545	8p22	17763927	rat	5408382	38383
8	8p22	17802310	8p21	19985979	rat	2183669	22305
8	8p21	20008284	8p12	28970543	rat	8962259	40514
8	8p12	29011057	8p12	36492063	rat	7481006	4200
8	8p12	36496263	8p12	37734651	rat	1238388	200
8	8p12	37734851	8p11.2	42229687	rat	4494836	8160578
8	8q11.2	50390265	8q12	62410873	rat	12020608	6557

8	8q12	62417430	8q12	63721790	rat	1304360	84653
8	8q12	63806443	8q13	67053743	rat	3247300	200
8	8q13	67053943	8q21.1	75892926	rat	8838983	28363
8	8q21.1	75921289	8q21.2	86714895	rat	10793606	7629
8	8q21.2	86722524	8q22.1	96911668	rat	10189144	4856
8	8q22.1	96916524	8q24.3	145731624	rat	48815100	
9	9p24	195689	9p24	6655150	rat	6459461	77984
9	9p24	6733134	9p21	27287159	rat	20554025	27931
9	9p21	27315090	9p13	38461949	rat	11146859	25821090
9	9q13	64283039	9q21.2	76347373	rat	12064334	216375
9	9q21.2	76563748	9q21.3	79693982	rat	3130234	99125
9	9q21.3	79793107	9q21.3	83878609	rat	4085502	247191
9	9q21.3	84125800	9q22.2	88142780	rat	4016980	159455
9	9q22.1	88302235	9q22.2	90365402	rat	2063167	283313
9	9q22.2	90648715	9q22.3	93280543	rat	2631828	215751
9	9q22.3	93496294	9q33	116942049	rat	23445755	52881
9	9q33	116994930	9q33	121405175	rat	4410245	562
9	9q33	121405737	9q33	124512769	rat	3107032	20241
9	9q33	124533010	9q34.1	127758645	rat	3225635	89421
9	9q34.1	127848066	9q34.1	129385133	rat	1537067	69066
9	9q34.1	129454199	9q34.1	131919050	rat	2464851	42997
9	9q34.1	131962047	9q34.2	134317723	rat	2355676	
10	10p15	128903	10p12.1	27676319	rat	27547416	61196
10	10p12.1	27737515	10p12.1	29062258	rat	1324743	844647
10	10p11.2	29906905	10p11.2	31026991	rat	1120086	76616
10	10p11.2	31103607	10p11.2	32605919	rat	1502312	289294
10	10p11.2	32895213	10p11.2	35324029	rat	2428816	446576
10	10p11.2	35770605	10p11.2	37244664	rat	1474059	5823396
10	10q11.2	43068060	10q11.2	46002988	rat	2934928	479655
10	10q11.2	46482643	10q11.2	51472743	rat	4990100	422617
10	10q11.2	51895360	10q21.1	54432491	rat	2537131	617385
10	10q21.1	55049876	10q21.3	68040493	rat	12990617	104644
10	10q21.3	68145137	10q22.1	70347467	rat	2202330	25729
10	10q21.3	70373196	10q22.1	74077294	rat	3704098	685639
10	10q22.2	74762933	10q22.3	79620307	rat	4857374	13098
10	10q22.3	79633405	10q22.3	81955638	rat	2322233	200
10	10q22.3	81955838	10q23.2	89041450	rat	7085612	350358
10	10q23.3	89391808	10q24.1	99061758	rat	9669950	362247
10	10q24.2	99424005	10q24.2	101662335	rat	2238330	101556
10	10q24.2	101763891	10q26.1	121313866	rat	19549975	200
10	10q26.1	121314066	10q26.3	135319316	rat	14005250	
11	11p15.5	1206762	11p15.5	3213050	rat	2006288	377291
11	11p15.4	3590341	11p15.1	17406125	rat	13815784	11208
11	11p15.1	17417333	11p14	25987264	rat	8569931	259985
11	11p14	26247249	11q12	58011814	rat	31764565	53955
11	11q12	58065769	11q13.3	71432282	rat	13366513	417499
11	11q13.4	71849781	11q14.3	89523970	rat	17674189	521121
11	11q14.3	90045091	11q22.3	106828099	rat	16783008	658845
11	11q22.3	107486944	11q25	134934771	rat	27447827	
12	12p13.3	61065	12p13.3	2682525	rat	2621460	100974
12	12p13.3	2783499	12p13.3	9174615	rat	6391116	107601
12	12p13.3	9282216	12p11.2	30652860	rat	21370644	7786188
12	12p12	38439048	12q13.2	54965452	rat	16526404	475826
12	12q13.3	55441278	12q14	56862885	rat	1421607	86023
12	12q14	56948908	12q24.1	108109650	rat	51160742	148796
12	12q24.1	108258446	12q24.1	110355800	rat	2097354	54625
12	12q24.1	110410425	12q24.31	121274782	rat	10864357	100922
12	12q24.31	121375704	12q24.33	132216098	rat	10840394	

13	13q12.1	18706513	13q12.1	22757233	rat	4050720	2967160
13	13q12.1	25724393	13q13	33345993	rat	7621600	55990
13	13q13	33401983	13q14.1	40188271	rat	6786288	238254
13	13q14.1	40426525	13q14.2	48715417	rat	8288892	415495
13	13q14.2	49130912	13q14.3	51291706	rat	2160794	883008
13	13q14.3	52174714	13q33	101917852	rat	49743138	458672
13	13q33	102376524	13q34	114114599	rat	11738075	
14	14q11.2	18307555	14q11.2	23137497	rat	4829942	4469
14	14q11.2	23141966	14q22	50241915	rat	27099949	444943
14	14q21	50686858	14q22	56619173	rat	5932315	38762
14	14q22	56657935	14q32.3	104300259	rat	47642324	
15	15q11.2	22618175	15q13	26033210	rat	3415035	774134
15	15q13	26807344	15q13	27946401	rat	1139057	606694
15	15q13	28553095	15q13	29997953	rat	1444858	506623
15	15q13	30504576	15q14	32238453	rat	1733877	277705
15	15q14	32516158	15q21.1	48877128	rat	16360970	666798
15	15q21.1	49543926	15q24	73600262	rat	24056336	83677
15	15q24	73683939	15q24	75763740	rat	2079801	106132
15	15q24	75869872	15q25	77858828	rat	1988956	17064
15	15q25	77875892	15q25	80152765	rat	2276873	646833
15	15q25	80799598	15q25	82374406	rat	1574808	1059203
15	15q25	83433609	15q25	89153038	rat	5719429	36437
15	15q26.1	89189475	15q26.3	99857350	rat	10667875	
16	16p13.3	43893	16p13.1	15528691	rat	15484798	1099940
16	16p13.1	16628631	16p12	18203373	rat	1574742	534908
16	16p12	18738281	16p11.2	31536969	rat	12798688	14898959
16	16q12.1	46435928	16q13	55602137	rat	9166209	12562
16	16q13	55614699	16q22	66704022	rat	11089323	26641
16	16q22	66730663	16q22	69711086	rat	2980423	152492
16	16q22	69863578	16q23	74093662	rat	4230084	75779
16	16q23	74169441	16q24	89810196	rat	15640755	
17	17p13	1199805	17p13	4482673	rat	3282868	42818
17	17p13	4525491	17p13	6691571	rat	2166080	56816
17	17p13	6748387	17p11.2	21227769	rat	14479382	4160586
17	17q11.2	25388355	17q11.2	27510680	rat	2122325	2802602
17	17q12	30313282	17q12	34303780	rat	3990498	269683
17	17q12	34573463	17q21.2	35922531	rat	1349068	397358
17	17q21.2	36319889	17q21.3	44302688	rat	7982799	1608697
17	17q21.3	45911385	17q23	58283088	rat	12371703	265116
17	17q23	58548204	17q24	60629869	rat	2081665	88415
17	17q24	60718284	17q24	63138270	rat	2419986	242548
17	17q24	63380818	17q24	66449451	rat	3068633	283836
17	17q24	66733287	17q25	81293587	rat	14560300	
18	18p11.32	605216	18p11.2	9962013	rat	9356797	1696502
18	18p11.2	11658515	18p11.2	13912027	rat	2253512	4519440
18	18q11.2	18431467	18q12.3	40945714	rat	22514247	299289
18	18q12.3	41245003	18q21.3	53943062	rat	12698059	110405
18	18q21.3	54053467	18q21.3	58048268	rat	3994801	70800
18	18q21.3	58119068	18q22	65639416	rat	7520348	803808
18	18q22	66443224	18q23	77744767	rat	11301543	
19	19p13.3	221430	19p13.3	4164193	rat	3942763	2890282
19	19p13.2	7054475	19p13.2	8217968	rat	1163493	1019786
19	19p13.2	9237754	19p13.2	11509875	rat	2272121	1092782
19	19p13.2	12602657	19p13.1	14528147	rat	1925490	263191
19	19p13.1	14791338	19p13.1	15948062	rat	1156724	79772
19	19p13.1	16027834	19p13.1	19620024	rat	3592190	13553296
19	19q12	33173320	19q12	34786782	rat	1613462	200
19	19q12	34786982	19q13.3	53042981	rat	18255999	434289

19	19q13.3	53477270	19q13.4	56776887	rat	3299617	2621348
19	19q13.4	59398235	19q13.4	60932972	rat	1534737	
20	20p13	16192	20p13	1390268	rat	1374076	305615
20	20p13	1695883	20p11.2	23584193	rat	21888310	6959182
20	20q11.2	30543375	20q13.3	63584301	rat	33040926	
21	21q11.2	14421381	21q22.3	42332141	rat	27910760	53363
21	21q22.3	42385504	21q22.3	46940182	rat	4554678	
22	22q11.2	17931957	22q11.2	20654635	rat	2722678	5060167
22	22q12.1	25714802	22q12.1	27480724	rat	1765922	12420
22	22q12.1	27493144	22q12.3	30824528	rat	3331384	1168058
22	22q12.3	31992586	22q12.3	34221854	rat	2229268	19440
22	22q12.3	34241294	22q13.3	49309226	rat	15067932	
X	Xp22.2	9318225	Xp22.11	23022644	rat	13704419	30269
X	Xp22.11	23052913	Xp21.1	36161279	rat	13108366	53642
X	Xp21.1	36214921	Xp11.3	46346466	rat	10131545	553547
X	Xp11.3	46900013	Xp11.23	48894439	rat	1994426	2864587
X	Xp11.22	51759026	Xp11.21	55750419	rat	3991393	5049672
X	Xq11.2	60800091	Xq22.3	105353823	rat	44553732	82426
X	Xq22.3	105436249	Xq23	113262020	rat	7825771	151359
X	Xq23	113413379	Xq25	123820873	rat	10407494	219917
X	Xq25	124040790	Xq28	151414871	rat	27374081	
