# Supplementary material for: Is mammalian chromosomal evolution driven by regions of genome fragility?
Source: Genome Biol. 2006 Dec 8;7(12):R115. doi: 10.1186/gb-2006-7-12-r115 (PMC1794428; doi:10.1186/gb-2006-7-12-r115)
Supplement: Additional data file 5 — List of evolutionary breakpoint regions (EBR) less than 4 Mb and their chromosomal positions in the human genome. [file gb-2006-7-12-r115-S5.pdf]

Table S2: List representing the evolutionary breakpoint regions (EBR) less than 4Mb and their chromosomal position in the human genome.

Human chr	from (band)	from (bp)	to (band)	to (bp)	species	EBR's size
1	1q23	161580755	1q24	164095901	cat	2515146
1	1q31	182950099	1q31	186338948	cat	3388849
1	1q42	224722192	1q42	225692983	cat	970791
2	2p22	35873540	2p22	39066275	cat	3192735
3	3p24	17806005	3p24	19362170	cat	1556165
3	3p21.2	51753995	3p21.2	53050080	cat	1296085
5	5q11.2	52508337	5q11.2	54691047	cat	2182710
5	5q13.2	70405708	5q13.3	74019724	cat	3614016
5	5q32	145444492	5q32	148190997	cat	2746505
6	6p22.1	28564483	6p22.1	29926866	cat	1362383
6	6q15	88825718	6q16.1	92138810	cat	3313092
7	7q22	101473628	7q22	103882428	cat	2408800
10	10p12.3	17429831	10p12.3	18579845	cat	1150014
10	10q24.3	102403107	10q24.3	104718952	cat	2315845
10	10q24.3	106017367	10q25.1	108657106	cat	2639739
11	11p15.5	2829324	11p15.4	5205733	cat	2376409
11	11p13	32496231	11p13	32890555	cat	394324
11	11q13.3	68958076	11q13.4	72389623	cat	3431547
11	11q22.1	101035333	11q22.2	102425313	cat	1389980
15	15q13	27070326	15q13	27572440	cat	502114
15	15q24	74939446	15q24	76132408	cat	1192962
15	15q25	81314475	15q25	84268789	cat	2954314
17	17q21.3	41186169	17q21.3	42293302	cat	1107133
17	17q23	59679495	17q24	62335374	cat	2655879
19	19p13.1	19314097	19p12	22153700	cat	2839603
19	19q13.3	55108809	19q13.4	59061575	cat	3952766
22	22q12.3	30831199	22q12.3	32688487	cat	1857288
22	22q13.1	37884099	22q13.2	40724570	cat	2840471
1	1p36.3	657101	1p36.3	659326	cattle	2225
1	1p36.3	659491	1p36.3	1185659	cattle	526168
1	1p36.2	15066499	1p36.1	15996102	cattle	929603
1	1p35	32178408	1p35	32828690	cattle	650282
1	1p35	32856534	1p34.3	35070436	cattle	2213902
1	1p34.3	36041724	1p34.3	38920455	cattle	2878731
1	1q24	164602310	1q24	166355840	cattle	1753530
1	1q24	166559098	1q24	168061951	cattle	1502853
1	1q24	168143429	1q25	170027236	cattle	1883807
1	1q25	180080269	1q31	182785859	cattle	2705590
1	1q31	189622181	1q31	193593332	cattle	3971151
1	1q32	198313571	1q32	199519571	cattle	1206000
1	1q32	203961724	1q32	204701027	cattle	739303
1	1q42	223312565	1q42	224655903	cattle	1343338
1	1q42	224672451	1q42	224713326	cattle	40875
1	1q42	224980083	1q42	226676144	cattle	1696061
1	1q43	236369146	1q43	237235402	cattle	866256
1	1q43	237817065	1q43	238368064	cattle	550999
1	1q44	241073844	1q44	245030235	cattle	3956391
2	2p23	31448970	2p23	31515982	cattle	67012
2	2p22	33582120	2p22	37386420	cattle	3804300
2	2p16	51217330	2p16	54711216	cattle	3493886
2	2p16	58344712	2p15	61372309	cattle	3027597
2	2p14	67595383	2p13	69926836	cattle	2331453

2	2p13	70274004	2p13	70465664	cattle	191660
2	2p13	70466012	2p13	71516603	cattle	1050591
2	2p13	74400121	2p13	74643151	cattle	243030
2	2p13	74714596	2p12	75677152	cattle	962556
2	2p12	75816416	2p12	79341841	cattle	3525425
2	2p11.2	85513118	2p11.2	85733916	cattle	220798
2	2q13	111831391	2q13	113438308	cattle	1606917
2	2q13	113867305	2q14.1	114554351	cattle	687046
2	2q21.1	131827274	2q21.3	135235659	cattle	3408385
2	2q32.2	188294465	2q32.2	191033384	cattle	2738919
2	2q32.2	191148604	2q32.2	191172219	cattle	23615
2	2q32.2	191200413	2q32.2	191265044	cattle	64631
2	2q32.2	191337954	2q32.2	191797784	cattle	459830
2	2q36	224431140	2q36	228300887	cattle	3869747
2	2q37.1	232637976	2q37.1	234232819	cattle	1594843
2	2q37.3	242142249	2q37.3	242637815	cattle	495566
2	2q37.3	242639521	2q37.3	243071390	cattle	431869
3	3p25	10160699	3p25	11286246	cattle	1125547
3	3p25	11296261	3p25	15288218	cattle	3991957
3	3p22	40167131	3p22	42442574	cattle	2275443
3	3p22	42528308	3p21.3	45550087	cattle	3021779
3	3q21	126014321	3q21	128598894	cattle	2584573
3	3q21	130535798	3q21	131700576	cattle	1164778
3	3q21	131701180	3q21	131894446	cattle	193266
3	3q22	137949578	3q22	139944679	cattle	1995101
4	4p16	657757	4p16	4262159	cattle	3604402
4	4p16	5815903	4p16	6710544	cattle	894641
4	4p15.3	17202205	4p15.3	19943231	cattle	2741026
4	4q22	88978287	4q22	89113522	cattle	135235
4	4q26	120943179	4q27	123569220	cattle	2626041
4	4q32	156604414	4q32	157100884	cattle	496470
5	5q14	80647195	5q14	80664539	cattle	17344
5	5q23.3	132144366	5q23.3	132242576	cattle	98210
5	5q32	146444172	5q32	149764362	cattle	3320190
5	5q34	161261955	5q34	162800120	cattle	1538165
5	5q35	176658350	5q35	176747058	cattle	88708
6	6p21.3	32560586	6p21.3	32604636	cattle	2485960
6	6q22.1	116447426	6q22.1	117882140	cattle	1434714
6	6q22.3	127599865	6q22.3	129139472	cattle	1539607
7	7p15.3	23274348	7p15.2	26039787	cattle	2765439
7	7p11.2	55488695	7p11.2	55740033	cattle	251338
7	7q11.23	75845912	7q21.1	79362390	cattle	3516478
7	7q21.3	96967711	7q22	97844204	cattle	876493
7	7q22	100328878	7q22	103313520	cattle	2984642
8	8p23.1	11567741	8p22	12716064	cattle	1148323
8	8p22	12909249	8p22	15740582	cattle	2831333
8	8p22	17751798	8p22	19070977	cattle	1319179
8	8p21	19269574	8p21	19606071	cattle	336497
8	8p21	28021411	8p12	30856388	cattle	2834977
8	8q11.2	52071773	8q11.2	53258203	cattle	1186430
8	8q12	59218602	8q12	59441162	cattle	222560
8	8q21.1	82118658	8q21.1	82293375	cattle	174717
8	8q22.3	103242039	8q22.3	104079660	cattle	837621
8	8q23	117447292	8q24.1	118201750	cattle	754458
8	8q24.1	120105393	8q24.1	120411813	cattle	306420
8	8q24.1	120513872	8q24.1	122294338	cattle	1780466
9	9p21	32563350	9p21	33094081	cattle	530731
9	9q21.3	79831533	9q21.3	81194075	cattle	1362542

9	9q21.3	81194393	9q21.3	84136387	cattle	2941994
10	10p11.2	30174969	10p11.2	33189173	cattle	3014204
10	10p11.2	33189252	10p11.2	33857582	cattle	668330
10	10p11.2	36080601	10q11.2	38496809	cattle	2416208
10	10q11.2	43537563	10q11.2	44640810	cattle	1103247
10	10q11.2	51023128	10q11.2	51958757	cattle	935629
10	10q11.2	51959007	10q21.1	53349423	cattle	1390416
10	10q21.1	58013421	10q21.2	61680561	cattle	3667140
11	11p15.5	2972880	11p15.4	3606922	cattle	634042
11	11p15.1	18086368	11p15.1	18339499	cattle	253131
11	11p14	23516713	11p14	26370088	cattle	2853375
11	11q12	59884931	11q12	60909075	cattle	1024144
11	11q13.3	68708644	11q13.4	71865223	cattle	3156579
11	11q13.5	76385891	11q14.1	77443084	cattle	1057193
11	11q14.1	79558251	11q14.1	80850130	cattle	1291879
11	11q14.1	82834337	11q14.1	83392819	cattle	558482
11	11q22.3	104299321	11q22.3	107647966	cattle	3348645
11	11q24	122966757	11q24	124671025	cattle	1704268
12	12q13.2	54689770	12q13.3	55795103	cattle	1105333
12	12q22	93768986	12q23	96596135	cattle	2827149
13	13q12.1	20824838	13q12.1	20858048	cattle	33210
14	14q21	48573670	14q22	51010044	cattle	2436374
14	14q22	51093480	14q22	51314031	cattle	220551
15	15q13	26034151	15q13	27140673	cattle	1106522
15	15q15	41092619	15q15	41244346	cattle	151727
15	15q15	41673925	15q15	42582853	cattle	908928
15	15q22.3	62574353	15q22.3	63482603	cattle	908250
15	15q23	70139318	15q24	72711998	cattle	2572680
15	15q25	80399292	15q25	83879506	cattle	3480214
16	16q22	69496123	16q22	69883602	cattle	387479
16	16q23	70570481	16q22	71660730	cattle	1090249
16	16q23	72828038	16q23	74224531	cattle	1396493
17	17q11.2	26647099	17q11.2	27236434	cattle	589335
17	17q21.3	40823819	17q21.3	43206153	cattle	2382334
17	17q21.3	43206254	17q21.3	45950561	cattle	2744307
17	17q23	59812060	17q24	62245622	cattle	2433562
17	17q24	62956701	17q24	64625694	cattle	1668993
18	18q21.1	28824451	18q12.2	32803951	cattle	3979500
18	18q12.3	40737968	18q21.1	44278910	cattle	3540942
18	18q22	60432639	18q22	61339916	cattle	907277
19	19p13.3	752327	19p13.3	765097	cattle	12770
19	19p13.3	1921309	19p13.3	2209523	cattle	288214
19	19p13.3	5093605	19p13.2	7879606	cattle	2786001
19	19p13.1	12837529	19p13.1	13109089	cattle	271560
19	19q12	34380163	19q12	34773239	cattle	393076
20	20p13	367193	20p13	2587041	cattle	2219848
20	20p12	5854003	20p12	8061296	cattle	2207293
20	20p12	16670419	20p12	17422551	cattle	752132
21	21q22.1	34224306	21q22.1	36362691	cattle	2138385
22	22q11.2	16685103	22q11.2	17274763	cattle	589660
22	22q11.2	22451056	22q11.2	23920379	cattle	1469323
22	22q12.2	30013052	22q12.3	31522195	cattle	1509143
22	22q12.3	35668902	22q13.1	36413032	cattle	744130
22	22q13.1	36413528	22q13.1	36929737	cattle	516209
X	Xq23	112920937	Xq23	115836971	cattle	2916034
1	1p36.2	8014674	1p36.2	8346713	chicken	332039
1	1p36.1	19966204	1p36.1	22150205	chicken	2184001
1	1p36.1	23037279	1p36.1	23091673	chicken	54394

1	1p36.1	26808478	1p36.1	26837169	chicken	28691
1	1p35	31511526	1p35	31758146	chicken	246620
1	1p34.3	39001878	1p34.3	39215721	chicken	213843
1	1p34.2	40232231	1p34.2	40993243	chicken	761012
1	1p34.2	42132810	1p34.2	43282699	chicken	1149889
1	1p31.2	66552275	1p31.1	69937886	chicken	3385611
1	1p31.1	75478444	1p31.1	76252436	chicken	773992
1	1p21	103834130	1p21	103943595	chicken	109465
1	1p13	109278244	1p13	110261632	chicken	983388
1	1p13	110944090	1p13	111697982	chicken	753892
1	1p13	115305369	1p13	115905892	chicken	600523
1	1q23	158771765	1q23	160128230	chicken	1356465
1	1q32	197561509	1q32	197836333	chicken	274824
1	1q32	199875897	1q32	200006028	chicken	130131
1	1q32	200444937	1q32	200809296	chicken	364359
1	1q32	206363172	1q32	206391780	chicken	28608
1	1q42	222902670	1q42	223126024	chicken	223354
1	1q42	223813187	1q42	224413065	chicken	599878
1	1q42	224873215	1q42	225713607	chicken	840392
2	2p23	24462884	2p23	25686209	chicken	1223325
2	2p23	27784720	2p23	27787939	chicken	3219
2	2p23	28551956	2p23	28950505	chicken	398549
2	2p23	31402964	2p22	32414612	chicken	1011648
2	2p22	38353401	2p22	38353401	chicken	0
2	2p22	40844899	2p21	42015962	chicken	1171063
2	2p21	47378320	2p16	47921948	chicken	543628
2	2p16	53253989	2p16	53839013	chicken	585024
2	2p15	61677308	2p15	62627954	chicken	950646
2	2p13	68716960	2p13	68913541	chicken	196581
2	2p13	69695825	2p13	71737158	chicken	2041333
2	2p13	73432621	2p12	76250211	chicken	2817590
2	2p12	77699659	2p12	79996688	chicken	2297029
2	2q13	109734101	2q13	112246145	chicken	2512044
2	2q13	112807186	2q14.1	114187516	chicken	1380330
2	2q14.2	120089519	2q14.2	120283518	chicken	193999
2	2q14.3	127356291	2q14.3	127892208	chicken	535917
2	2q21.1	131738252	2q21.2	133004690	chicken	1266438
2	2q24.2	160450291	2q24.2	160487086	chicken	36795
2	2q33	208730414	2q34	210269529	chicken	1539115
2	2q35	216408870	2q35	216786189	chicken	377319
2	2q35	220332157	2q35	220370817	chicken	38660
2	2q36	230759214	2q37.1	231747104	chicken	987890
2	2q37.1	233271732	2q37.1	233323309	chicken	51577
2	2q37.1	234162762	2q37.2	236302959	chicken	2140197
2	2q37.3	239853848	2q37.3	241376637	chicken	1522789
3	3p25	9401361	3p25	9439480	chicken	38119
3	3p25	9968818	3p25	10342507	chicken	373689
3	3p25	13896319	3p25	13896319	chicken	0
3	3p25	15112644	3p25	15467794	chicken	355150
3	3p23	32805550	3p23	32969907	chicken	164357
3	3p22	37067234	3p22	37069490	chicken	2256
3	3p22	37840031	3p22	37952849	chicken	112818
3	3p22	39170169	3p22	40886019	chicken	1715850
3	3p22	42280144	3p22	42547254	chicken	769103
3	3p22	42892325	3p22	43049247	chicken	156922
3	3p21.3	46425544	3p21.3	46920035	chicken	1774797
3	3p21.3	48200341	3p21.3	48200341	chicken	0
3	3p21.3	49188285	3p21.3	49522833	chicken	334548

3	3p21.2	52454845	3p21.2	53234673	chicken	779828
3	3p12	75384120	3p12	75956823	chicken	572703
3	3p12	87417864	3q11.2	88187166	chicken	769302
3	3q11.2	98986611	3q12	100557426	chicken	1570815
3	3q13.2	113163762	3q13.2	113733923	chicken	570161
3	3q13.3	122746458	3q21	123455464	chicken	709006
3	3q21	123668981	3q21	123941865	chicken	272884
3	3q21	126784631	3q21	127934594	chicken	1149963
3	3q21	130016373	3q21	130086108	chicken	69735
3	3q21	130373357	3q21	130582775	chicken	209418
3	3q21	131030058	3q21	131577718	chicken	547660
3	3q22	134860720	3q22	135007407	chicken	146687
3	3q22	137100488	3q22	137202915	chicken	102427
3	3q22	137832526	3q22	138240587	chicken	408061
3	3q23	142267635	3q23	142586595	chicken	318960
3	3q24	146396455	3q24	147641798	chicken	1245343
3	3q24	149667183	3q24	150194528	chicken	527345
3	3q27	184542346	3q27	188427232	chicken	3884886
3	3q29	195890199	3q29	197538979	chicken	1648780
4	4p16	951495	4p16	1137188	chicken	185693
4	4q12	57166633	4q12	58433224	chicken	1266591
4	4q21.1	78344753	4q21.2	79491790	chicken	1147037
4	4q21.2	79819632	4q21.2	79930793	chicken	111161
4	4q21.2	86138934	4q22	89661448	chicken	3522514
4	4q22	95596429	4q22	95801442	chicken	205013
4	4q28	128763245	4q28	128921862	chicken	158617
4	4q28	130154003	4q28	130459253	chicken	305250
4	4q31.3	153767367	4q31.3	154430962	chicken	663595
4	4q33	171049735	4q34	172309677	chicken	1259942
4	4q35	186718890	4q35	186725597	chicken	6707
5	5p15.3	1147393	5p15.3	1254573	chicken	107180
5	5p15.3	4902020	5p15.3	6425621	chicken	1523601
5	5q11.2	53553691	5q11.2	54587880	chicken	1034189
5	5q13.2	68600910	5q13.2	71465693	chicken	2864783
5	5q13.3	76313068	5q14	78609427	chicken	2296359
5	5q14	79534645	5q14	79734295	chicken	199650
5	5q14	86265663	5q14	88112736	chicken	1847073
5	5q15	95873089	5q15	96339834	chicken	466745
5	5q23.3	128192279	5q23.3	130787504	chicken	2595225
5	5q33.3	158645657	5q33.3	159275973	chicken	630316
6	6p22.2	24704874	6p22.3	24758995	chicken	54121
6	6p22.2	25809635	6p22.2	25834111	chicken	24476
6	6p21.3	35546505	6p21.3	35549360	chicken	2855
6	6p21.3	36308562	6p21.2	37429617	chicken	1121055
6	6p21.1	41039056	6p21.1	41447825	chicken	408769
6	6p21.1	42153061	6p21.1	42557986	chicken	404925
6	6p21.1	42944344	6p21.1	43082229	chicken	137885
6	6p21.1	43300164	6p21.1	43411718	chicken	111554
6	6p21.1	44388292	6p21.1	44463388	chicken	75096
6	6p12	52489510	6p12	52712355	chicken	222845
6	6q24	144336485	6q24	144810356	chicken	473871
6	6q25.3	159167979	6q25.3	160073718	chicken	905739
7	7p22	5573946	7p22	5982904	chicken	408958
7	7p22	6297062	7p21	7046962	chicken	749900
7	7p13	43453589	7p13	45387484	chicken	1933895
7	7p12	47252589	7p12	47907912	chicken	655323
7	7p11.2	54457923	7p11.2	54984120	chicken	526197
7	7q11.23	75788139	7q11.23	76791807	chicken	1003668

7	7q21.1	83000325	7q21.1	83206853	chicken	206528
7	7q21.1	86473042	7q21.1	86623012	chicken	149970
7	7q21.3	96391869	7q21.3	97242963	chicken	851094
7	7q21.3	97594284	7q22	98269689	chicken	675405
7	7q22	98701555	7q22	100551849	chicken	1850294
7	7q22	101802475	7q22	101802475	chicken	0
7	7q31.1	107357576	7q31.1	107381925	chicken	24349
7	7q32	126771269	7q32	128437335	chicken	1666066
7	7q32	129759213	7q32	130868489	chicken	1109276
7	7q33	132174783	7q33	132394899	chicken	220116
7	7q33	133525046	7q33	133803407	chicken	278361
7	7q33	134112641	7q33	134158751	chicken	46110
7	7q33	134790746	7q33	134816079	chicken	25333
7	7q34	140903503	7q34	141913675	chicken	1010172
7	7q34	142725902	7q35	143863863	chicken	1137961
7	7q36	148163633	7q36	150480038	chicken	2316405
8	8p23.1	6643537	8p23.1	9475005	chicken	2831468
8	8p23.1	9677262	8p23.1	10039998	chicken	362736
8	8p23.1	11748385	8p22	12907973	chicken	1159588
8	8p21	25961234	8p21	28013240	chicken	2052006
8	8p12	29104530	8p12	32286668	chicken	3182138
8	8q24.1	120187610	8q24.1	120621793	chicken	434183
9	9p24	8643415	9p23	12493211	chicken	3849796
9	9p23	12841201	9p13	15442998	chicken	2601797
9	9p21	21049498	9p21	23265977	chicken	2216479
9	9q22.3	91715989	9q22.3	92070817	chicken	354828
9	9q22.3	92494637	9q22.3	93070679	chicken	576042
9	9q22.3	98419936	9q22.3	98827383	chicken	407447
9	9q22.3	98995468	9q22.3	99018500	chicken	23032
9	9q31	100292759	9q31	101111333	chicken	818574
9	9q31	101539322	9q31	105036171	chicken	3496849
9	9q31	106891529	9q31	107976560	chicken	1085031
9	9q31	108258424	9q31	108875137	chicken	616713
9	9q31	109310005	9q31	109575581	chicken	265576
9	9q32	112732207	9q32	113098930	chicken	366723
9	9q32	113898629	9q32	113960102	chicken	61473
9	9q33	120464612	9q33	120640275	chicken	175663
9	9q33	127235485	9q34.1	127559023	chicken	323538
9	9q34.3	136897210	9q34.3	136926626	chicken	29416
10	10p15	4812889	10p15	5405648	chicken	592759
10	10p15	5971424	10p14	7602652	chicken	1631228
10	10p13	14323662	10p13	15592159	chicken	1268497
10	10q11.2	43021605	10q11.2	45189616	chicken	2168011
10	10q11.2	45479311	10q11.2	49056087	chicken	3576776
10	10q11.2	50449782	10q11.2	50489298	chicken	39516
10	10q11.2	50758433	10q21.1	53648790	chicken	2890357
10	10q21.1	54051981	10q21.1	55235946	chicken	1183965
10	10q21.1	56015683	10q21.1	59868172	chicken	3852489
10	10q21.1	60261224	10q21.2	61082266	chicken	821042
10	10q21.3	68715724	10q21.3	69348091	chicken	632367
10	10q22.1	74576201	10q22.2	74866374	chicken	290173
10	10q22.3	80875574	10q22.3	82254395	chicken	1378821
10	10q22.3	82396943	10q23.1	85891216	chicken	3494273
10	10q23.2	88940694	10q23.3	89255042	chicken	314348
10	10q23.3	89872817	10q23.3	90953099	chicken	1080282
10	10q23.3	96285673	10q23.3	96819149	chicken	533476
10	10q24.1	97286106	10q24.1	97794125	chicken	508019
10	10q24.1	98085815	10q24.1	98114363	chicken	28548

10	10q24.1	98459744	10q24.1	98654194	chicken	194450
10	10q24.2	101825872	10q24.3	102025130	chicken	199258
10	10q24.3	102632157	10q24.3	102665596	chicken	33439
10	10q24.3	103900153	10q24.3	103901997	chicken	1844
10	10q24.3	104174942	10q24.3	104218789	chicken	43847
10	10q26.1	125796126	10q26.2	129729097	chicken	3932971
11	11p15.5	480528	11p15.5	949416	chicken	468888
11	11p15.5	3108914	11p15.4	4345175	chicken	1236261
11	11p15.4	6619322	11p15.4	7280828	chicken	661506
11	11p15.4	7682866	11p15.4	8074792	chicken	391926
11	11p15.2	13723547	11p15.2	13853783	chicken	130236
11	11p15.2	14246001	11p15.2	14255922	chicken	9921
11	11p15.1	18722630	11p15.1	19690704	chicken	968074
11	11p13	33662373	11p13	34458185	chicken	795812
11	11p11.12	47568862	11p11.12	49124884	chicken	1556022
11	11q13.4	70536070	11q13.4	72225425	chicken	1689355
11	11q13.5	74955638	11q13.5	77317025	chicken	2361387
11	11q22.3	109984997	11q23	111205069	chicken	1220072
12	12p11.2	27072760	12p11.2	27760447	chicken	687687
12	12p11.2	29012215	12p11.2	29508691	chicken	496476
12	12q23	100302277	12q23	100371450	chicken	69173
12	12q23	106679140	12q23	106793205	chicken	114065
12	12q24.1	111444658	12q24.1	112091631	chicken	646973
12	12q24.31	119917101	12q24.31	121762436	chicken	1845335
13	13q31	79413807	13q31	79473307	chicken	59500
13	13q33	107431655	13q33	107913407	chicken	481752
14	14q32.3	104706611	14q32.3	104706611	chicken	0
15	15q11.2	20613794	15q12	23475285	chicken	2861491
15	15q13	26255370	15q13	27779573	chicken	1524203
15	15q13	29156503	15q13	30699461	chicken	1542958
15	15q15	38172249	15q15	38248753	chicken	76504
15	15q15	38858827	15q15	38924102	chicken	65275
15	15q15	41264077	15q15	41450296	chicken	186219
15	15q21.1	42740113	15q21.1	43119875	chicken	379762
15	15q21.1	43354999	15q21.1	43813810	chicken	458811
15	15q22.3	62063017	15q22.3	62191815	chicken	128798
15	15q22.3	62842540	15q22.3	63275573	chicken	433033
15	15q23	68779125	15q23	68911302	chicken	132177
15	15q23	69862655	15q23	69885929	chicken	23274
15	15q24	72881488	15q24	73933776	chicken	1052288
15	15q25	76714973	15q25	77891736	chicken	1176763
15	15q25	82586947	15q25	82586947	chicken	0
15	15q25	83289442	15q25	83877721	chicken	588279
15	15q25	88047379	15q26.1	89096018	chicken	1048639
15	15q26.1	89297551	15q26.1	89567887	chicken	270336
16	16p13.3	367902	16p13.3	388620	chicken	20718
16	16p13.3	713176	16p13.3	713176	chicken	0
16	16p13.3	1211786	16p13.3	1492344	chicken	280558
16	16p13.3	1690111	16p13.3	1797731	chicken	107620
16	16p13.3	2227488	16p13.3	3459326	chicken	1231838
16	16p13.3	4464150	16p13.3	4837821	chicken	373671
16	16p13.3	5087800	16p13.3	6126760	chicken	1038960
16	16p13.1	11256892	16p13.1	12479033	chicken	1222141
16	16p13.1	14691984	16p13.1	15582543	chicken	890559
16	16p12	18845522	16p12	21915152	chicken	3069630
16	16p12	27145727	16p11.2	29031647	chicken	1885920
16	16q21	57104792	16q21	57408214	chicken	303422
16	16q22	65534181	16q22	65624066	chicken	89885

16	16q22	66963693	16q22	68972224	chicken	2008531
16	16q22	69877357	16q22	72923956	chicken	3046599
16	16q23	73911511	16q23	75873885	chicken	1962374
17	17p13	2186447	17p13	2211664	chicken	25217
17	17p13	3582554	17p13	3712283	chicken	129729
17	17p13	4157201	17p13	5457386	chicken	1300185
17	17p13	6237594	17p13	8236729	chicken	1999135
17	17p12	15175628	17p12	15818939	chicken	643311
17	17p11.2	16277751	17p11.2	16277751	chicken	0
17	17p11.2	21267116	17q11.2	22645479	chicken	1378363
17	17q11.2	23695741	17q11.2	23699031	chicken	3290
17	17q21.3	44844292	17q21.3	47061154	chicken	2216862
17	17q23	52438820	17q23	52537644	chicken	98824
17	17q23	57679550	17q23	57837692	chicken	158142
17	17q23	59250410	17q23	59468673	chicken	218263
17	17q25	69567432	17q25	71113486	chicken	1546054
17	17q25	73615159	17q25	77485525	chicken	3870366
18	18p11.2	8396947	18p11.2	9982668	chicken	1585721
18	18p11.2	13880099	18q11.2	16783794	chicken	2903695
18	18q12.2	31002282	18q12.2	31302431	chicken	300149
18	18q12.2	32623593	18q12.2	33723305	chicken	1099712
18	18q21.1	43136264	18q21.1	43613642	chicken	477378
18	18q22	59805496	18q21.3	59865734	chicken	60238
18	18q23	72873406	18q23	73652540	chicken	779134
19	19p13.3	2259575	19p13.3	4602826	chicken	2343251
19	19p13.2	8669945	19p13.1	16039049	chicken	7369104
19	19p13.1	19477919	19p12	22486003	chicken	3008084
19	19q13.1	39684108	19q13.2	43635350	chicken	3951242
20	20p13	4178097	20p13	4712436	chicken	534339
20	20p12	5126548	20p12	5126548	chicken	0
20	20p12	13746140	20p12	14071964	chicken	325824
20	20p12	16679764	20p11.2	19964839	chicken	3285075
20	20p11.2	22497809	20p11.2	24752142	chicken	2254333
20	20q11.2	30901944	20q11.2	31597325	chicken	695381
20	20q11.2	34605332	20q11.2	35689167	chicken	1083835
20	20q11.2	36223597	20q11.2	36685358	chicken	461761
20	20q13.1	47532990	20q13.1	47682841	chicken	149851
21	21q22.3	44000557	21q22.3	46137542	chicken	2136985
22	22q11.2	16988468	22q11.2	17692784	chicken	704316
22	22q11.2	20654768	22q11.2	21761952	chicken	1107184
22	22q12.1	27778011	22q12.2	27992589	chicken	214578
22	22q12.3	30830867	22q12.3	31108492	chicken	277625
22	22q13.2	41335900	22q13.2	41340275	chicken	4375
22	22q13.3	44687806	22q13.3	45508901	chicken	821095
22	22q13.3	47541745	22q13.3	47764631	chicken	222886
X	Xp11.3	45492563	Xp11.3	46222311	chicken	729748
X	Xq13.1	70577663	Xq13.2	73523822	chicken	2946159
X	Xq21.1	77188027	Xq21.1	77809249	chicken	621222
X	Xq21.1	78851521	Xq21.1	80138376	chicken	1286855
X	Xq22.1	99990589	Xq22.2	103217204	chicken	3226615
X	Xq22.3	105923657	Xq23	109052728	chicken	3129071
X	Xq25	128378662	Xq26.2	130914166	chicken	2535504
X	Xq26.3	137667500	Xq27.1	138890958	chicken	1223458
1	1p36.22	10382321	1p36.22	10393599	dog	11278
1	1p35.1	33641688	1p35.1	33648344	dog	6656
1	1p34.2	43218471	1p34.2	43278840	dog	60369
1	1p32.3	53002763	1p32.3	53003633	dog	870
1	1p31.2	67643657	1p31.2	67643658	dog	1

1	1p13.2	111718814	1p13.2	111722713	dog	3899
1	1p13.2	113947730	1p13.2	113948585	dog	855
1	1q21.3	150079679	1q21.3	150138138	dog	58459
1	1q32.1	199920606	1q32.1	199932654	dog	12048
1	1q32.1	203176244	1q32.1	203194672	dog	18428
1	1q44	243460072	1q44	243774014	dog	313942
1	1q44	244065183	1q44	244328000	dog	262817
1	1q44	245065271	1q44	245306495	dog	241224
2	2p21	42896353	2p21	42899011	dog	2658
2	2p13.3	71199984	2p13.3	71199985	dog	1
2	2q11.2	96656378	2q11.2	96679345	dog	22967
2	2q13	109821753	2q13	110177993	dog	356240
2	2q13	113855973	2q13	113884132	dog	28159
2	2q14.1	114038202	2q14.1	114145966	dog	107764
2	2q21.1	130390523	2q21.1	131311558	dog	921035
2	2q21.1	131747793	2q21.2	132438713	dog	690920
2	2q21.2	132599047	2q21.2	132972107	dog	373060
2	2q23.3	153457546	2q23.3	153661565	dog	204019
2	2q32.2	190041649	2q32.2	190131662	dog	90013
2	2q36.2	225096837	2q36.2	225097358	dog	521
3	3p25.2	12872767	3p25.2	12909573	dog	36806
3	3p25.1	15138279	3p25.1	15162939	dog	24660
3	3p23	32673929	3p23	32675344	dog	1415
3	3p21.3	44600317	3p21.3	44719392	dog	119075
3	3p12.3	75411831	3p12.3	75411831	dog	0
3	3p12.3	75722985	3p12.3	75944922	dog	221937
3	3q25.32	159979299	3q25.32	159979311	dog	12
3	3q26.32	180198708	3q26.32	180214406	dog	15698
3	3q29	194780147	3q29	194780327	dog	180
3	3q29	195495418	3q29	195538929	dog	43511
4	4p16.3	1231195	4p16.3	1272687	dog	41492
4	4p16.3	3919912	4p16.3	4055037	dog	135125
4	4p16.3	4238573	4p16.3	4302249	dog	63676
4	4p16.1	6333095	4p16.1	6439809	dog	106714
4	4p16.1	8850821	4p16.1	9199334	dog	348513
4	4p16.1	9388939	4p16.1	9441011	dog	52072
4	4p15.3	17436502	4p15.3	17436503	dog	1
4	4p13	41164479	4p13	41166419	dog	1940
4	4p12	48924940	4q12	52529825	dog	3604885
4	4q13.3	75714013	4q13.3	75983576	dog	269563
4	4q21.1	76653750	4q21.1	76751895	dog	98145
4	4q27	121350309	4q27	121350633	dog	324
4	4q31.21	144761733	4q31.21	144786115	dog	24382
4	4q32.3	169618366	4q32.3	169723663	dog	105297
4	4q34.2	176804918	4q34.2	176816947	dog	12029
5	5p15.2	14749628	5p15.2	14949323	dog	199695
5	5p12	45939847	5q11.2	49605557	dog	3665710
5	5q35.3	176931766	5q35.3	177423016	dog	491250
6	6p22.1	29757150	6p21.33	30129521	dog	372371
6	6p21.33	30289669	6p21.33	30399903	dog	110234
6	6p11.2	58195618	6q12	62025056	dog	3829438
6	6q22.3	116717761	6q22.3	116718611	dog	850
6	6q23.2	131729655	6q23.2	131895759	dog	166104
6	6q23.2	132113394	6q23.2	132113395	dog	1
6	6q23.2	132280804	6q23.2	132285635	dog	4831
6	6q27	169912904	6q27	169920119	dog	7215
7	7p22	6520044	7p22	6813098	dog	293054
7	7q31.32	122749795	7q31.32	122770216	dog	20421

7	7q33	133750212	7q33	133757747	dog	7535
7	7q34	137385080	7q34	137568329	dog	183249
7	7q36.1	148170205	7q36.1	148198081	dog	27876
8	8p23.3	458855	8p23.3	472774	dog	13919
8	8p23.3	1736205	8p23.3	2104208	dog	368003
8	8p23.1	7404055	8p23.1	8140777	dog	736722
8	8q11.2	48819182	8q11.2	48847055	dog	27873
8	8q22.1	98817981	8q22.1	98831935	dog	13954
9	9p24	5843772	9p24	5877708	dog	33936
9	9q33.2	121295766	9q33.2	121295768	dog	2
10	10p12.1	27658010	10p11.23	29776485	dog	2118475
10	10p11.21	37137798	10p11.21	37229772	dog	91974
10	10p11.21	37442197	10p11.21	37454831	dog	12634
10	10p11.21	37575497	10p11.21	38150113	dog	574616
10	10p11.21	38450766	10p11.21	38560470	dog	109704
10	10p11.21	38677520	10q11.21	42583565	dog	3906045
10	10q11.22	46102264	10q11.22	46283519	dog	181255
10	10q11.22	46594244	10q11.22	47937830	dog	1343586
10	10q11.22	48232895	10q11.22	48339957	dog	107062
11	11p15.5	783824	11p15.5	787154	dog	3330
11	11p15.4	3226658	11p15.4	3379328	dog	152670
11	11p15.4	3569927	11p15.4	3582092	dog	12165
11	11p14	30237414	11p14	30247659	dog	10245
11	11p11.2	44035700	11p11.2	44036412	dog	712
11	11q12.1	59275943	11q12.1	59291321	dog	15378
11	11q12.2	60003192	11q12.2	60036669	dog	33477
11	11q13.4	70893857	11q13.4	71017252	dog	123395
11	11q13.4	71193468	11q13.4	71304291	dog	110823
11	11q22.1	100814003	11q22.1	100827814	dog	13811
12	12p13.3	2723684	12p13.3	2730380	dog	6696
12	12q13.2	54012775	12q13.2	54287763	dog	274988
12	12q21.1	74177243	12q21.1	74177244	dog	1
12	12q23.3	103086679	12q23.3	103102923	dog	16244
12	12q23.3	106684856	12q23.3	106814087	dog	129231
12	12q24.11	108949566	12q24.11	108951491	dog	1925
13	13q14.11	40332943	13q14.11	40365243	dog	32300
13	13q14.3	51584594	13q14.3	51669217	dog	84623
13	13q14.3	52111818	13q14.3	52113304	dog	1486
14	14q11.2	21253497	14q11.2	21395918	dog	142421
15	15q11.2	19915888	15q11.2	20384797	dog	468909
15	15q11.2	20945083	15q11.2	21345654	dog	400571
15	15q13.1	26256384	15q11.2	26618210	dog	361826
15	15q13.1	26726450	15q13.1	26996749	dog	270299
15	15q13.3	30410087	15q13.1	30689725	dog	279638
15	15q24.2	73815789	15q13.3	73913340	dog	97551
15	15q24.3	75985591	15q24.2	76049817	dog	64226
15	15q25.1	76479556	15q24.3	76513591	dog	34035
15	15q25.1	76788398	15q25.1	76830782	dog	42384
15	15q26.1	89369223	15q26.1	89393005	dog	23782
15	15q26.3	100084049	15q26.1	100158792	dog	74743
16	16p11.2	28521388	16p11.2	28621408	dog	100020
16	16p11.2	28953950	16p11.2	29026963	dog	73013
16	16p11.2	29252547	16p11.2	29561340	dog	308793
16	16q12.1	45539453	16q12.1	45544006	dog	4553
16	16q21	57366106	16q21	57406651	dog	40545
17	17p13.3	1184941	17p13.3	1191122	dog	6181
17	17p13.2	3767755	17p13.2	3853747	dog	85992
17	17p11.2	21281418	17q11.2	22549716	dog	1268298

17	17q12	31512484	17q12	33605007	dog	2092523
17	17q24.1	60190842	17q24.2	63727001	dog	3536159
17	17q24.3	65862760	17q24.3	65885993	dog	23233
18	18p11.21	13122946	18p11.21	13153897	dog	30951
18	18p11.21	14020900	18p11.21	15007216	dog	986316
18	18p11.21	15152965	18q11.2	16774339	dog	1621374
18	18q21.1	43823147	18q21.1	43823148	dog	1
18	18q21.1	46143061	18q21.1	46560528	dog	417467
20	20p11.2	23470914	20p11.2	23878991	dog	408077
20	20p11.2	25553589	20q11.2	29297001	dog	3743412
21	21q11.2	13636377	21q11.2	14400619	dog	764242
22	22q11.21	17035708	22q11.21	17384761	dog	349053
22	22q11.21	18719485	22q11.21	22255853	dog	3536368
22	22q11.21	23363676	22q11.21	23544486	dog	180810
22	22q12.3	30977686	22q12.3	31107873	dog	130187
X	Xq11.2	63440737	Xq11.2	63916710	dog	475973
X	Xq21.3	86731882	Xq21.3	90311848	dog	3579966
7	7q11.23	72881325	7q11.23	76765093	horse	3883768
8	8p21	23361090	8p21	27276054	horse	3914964
11	11p15.5	2119251	11p15.4	5205763	horse	3086512
13	13q14.1	39302438	13q14.1	40572332	horse	1269894
17	17q24	62391090	17q24	62913279	horse	522189
22	22q12.2	30000274	22q12.3	31579882	horse	1579608
X	Xq23	113607456	Xq23	115007213	horse	1399757
X	Xq24	117578597	Xq24	120270358	horse	2691761
1	1p32	58369551	1p32	58478059	mouse	108508
1	1p31.2	66969862	1p31.2	67947982	mouse	978120
1	1p22	89101147	1p22	89101347	mouse	200
1	1p22	93175140	1p22	93262870	mouse	87730
1	1q23	154874654	1q23	155300088	mouse	425434
1	1q32	204164021	1q32	204219578	mouse	55557
1	1q42	220829162	1q42	220829362	mouse	200
1	1q42	222368518	1q42	222417706	mouse	49188
1	1q42	224030267	1q42	225235652	mouse	1205385
1	1q42	231615019	1q42	231635058	mouse	20039
1	1q43	236366875	1q43	236517890	mouse	151015
2	2p25.3	1581123	2p25.3	1725442	mouse	144319
2	2p25.3	3119186	2p25.3	3235032	mouse	115846
2	2p23	26308539	2p23	26353794	mouse	45255
2	2p23	28962129	2p23	28993931	mouse	31802
2	2p16	53300424	2p16	53845778	mouse	545354
2	2p13	68650089	2p13	68652539	mouse	2450
2	2p13	70975429	2p13	70990164	mouse	14735
2	2p13	74026347	2p13	74026547	mouse	200
2	2q12	106560466	2q12	106988785	mouse	428319
2	2q12	108672097	2q13	110419967	mouse	1747870
2	2q13	113554679	2q14.1	114345182	mouse	790503
2	2q14.3	122483460	2q14.3	123877004	mouse	1393544
2	2q14.3	124910919	2q14.3	127121695	mouse	2210776
2	2q14.3	128690762	2q14.3	128726070	mouse	35308
2	2q21.1	131913009	2q21.2	133161124	mouse	1248115
2	2q22	138624340	2q22	139955335	mouse	1330995
2	2q32.1	188356222	2q32.1	188546182	mouse	189960
2	2q32.2	190478738	2q32.2	190478938	mouse	200
2	2q32.3	196737493	2q32.3	196737963	mouse	470
3	3p25	12864927	3p25	12935539	mouse	70612
3	3p25	15135116	3p25	16302013	mouse	1166897
3	3p24	20077784	3p24	22997012	mouse	2919228

3	3p24	27550695	3p24	27605472	mouse	54777
3	3p22	37067985	3p22	37082953	mouse	14968
3	3p14.3	57784230	3p14.3	57800515	mouse	16285
3	3p14.1	63864162	3p14.1	63872693	mouse	8531
3	3p12	75092934	3p12	75733563	mouse	640629
3	3q21	126621496	3q21	127015941	mouse	394445
3	3q21	130317226	3q21	131213263	mouse	896037
3	3q24	149369030	3q24	150355637	mouse	986607
3	3q26.1	169224070	3q26.2	169298816	mouse	74746
3	3q26.2	171956943	3q26.2	171957143	mouse	200
3	3q26.3	179505847	3q26.3	179510053	mouse	4206
3	3q27	184220160	3q26.3	184243568	mouse	23408
3	3q29	196687900	3q29	196836896	mouse	148996
4	4p16	3792758	4p16	4198357	mouse	405599
4	4p16	8859586	4p16	9474941	mouse	615355
4	4q22	89368712	4q22	89368912	mouse	200
4	4q22	95665419	4q22	95686485	mouse	21066
4	4q26	121086919	4q27	121332329	mouse	245410
4	4q27	122734117	4q27	122734317	mouse	200
4	4q31.1	141656124	4q31.1	141721737	mouse	65613
4	4q31.2	151341774	4q31.2	151432696	mouse	90922
4	4q32	163691876	4q32	163971547	mouse	279671
5	5p15.3	8302649	5p15.3	8302849	mouse	200
5	5p12	42875358	5p12	43487040	mouse	611682
5	5q15	96161775	5q15	96233455	mouse	71680
5	5q21	98428174	5q21	98428374	mouse	200
5	5q21	101907132	5q21	102790626	mouse	883494
5	5q22	110093254	5q22	110329574	mouse	236320
5	5q22	112320424	5q22	112320624	mouse	200
5	5q23.3	130393506	5q23.3	130515065	mouse	121559
5	5q31.1	134084748	5q31.1	134105170	mouse	20422
5	5q31.1	137121370	5q31.1	137256961	mouse	135591
5	5q32	147606363	5q32	147631312	mouse	24949
5	5q33.1	150267493	5q33.1	150368888	mouse	101395
5	5q33.2	154303864	5q33.2	154310069	mouse	6205
5	5q35	171867674	5q35	173698426	mouse	1830752
5	5q35	177106680	5q35	177483917	mouse	377237
6	6p22.3	20123166	6p22.3	20128162	mouse	4996
6	6p22.1	29077605	6p22.1	29140908	mouse	63303
6	6p21.3	33298631	6p21.3	33360955	mouse	62324
6	6p21.2	39059211	6p21.2	39280062	mouse	220851
6	6p12	49700593	6p12	49804425	mouse	103832
6	6p12	52714965	6p12	52725247	mouse	10282
6	6p12	55784269	6p12	56225900	mouse	441631
6	6q13	73858776	6q13	74013182	mouse	154406
6	6q14	86338899	6q14	87745373	mouse	1406474
6	6q16.3	100244557	6q16.3	100535267	mouse	290710
6	6q22.1	116988488	6q22.1	117049038	mouse	60550
6	6q22.3	123084295	6q22.3	123135007	mouse	50712
6	6q25.1	150140235	6q25.1	150201242	mouse	61007
6	6q25.2	154942085	6q25.2	155009552	mouse	67467
6	6q25.3	158794650	6q25.3	159937583	mouse	1142933
6	6q27	167369970	6q27	167761130	mouse	391160
7	7p21	12241969	7p21	12272471	mouse	30502
7	7p21	19456148	7p21	19463757	mouse	7609
7	7p15.3	22236982	7p15.3	22963958	mouse	726976
7	7p14	32800883	7p14	32878146	mouse	77263
7	7p14	36045970	7p14	36231162	mouse	185192

7	7p13	43312709	7p13	43621438	mouse	308729
7	7q11.23	71582029	7q11.23	72128832	mouse	546803
7	7q11.23	73887786	7q11.23	76308307	mouse	2420521
7	7q21.1	84871677	7q21.1	84932535	mouse	60858
7	7q21.3	92243945	7q21.2	92334045	mouse	90100
7	7q21.3	97098638	7q21.3	97204900	mouse	106262
7	7q22	98816819	7q22	99123663	mouse	306844
7	7q22	101660815	7q22	101878946	mouse	218131
7	7q22	104742436	7q22	104754839	mouse	12403
7	7q31.1	107320074	7q31.1	107323301	mouse	3227
7	7q31.1	111682409	7q31.1	111686189	mouse	3780
7	7q36	149864544	7q36	149903378	mouse	38834
7	7q36	156511361	7q36	156531925	mouse	20564
8	8p23.1	7056332	8p23.1	7981988	mouse	925656
8	8p23.1	9510886	8p23.1	9579292	mouse	68406
8	8p23.1	11711299	8p23.1	12354260	mouse	642961
8	8p22	17763961	8p22	17802310	mouse	38349
8	8p21	19985868	8p21	20008284	mouse	22416
8	8p12	28970491	8p12	29011077	mouse	40586
8	8p12	36514720	8p12	36514920	mouse	200
8	8p12	37697674	8p12	37703986	mouse	6312
8	8q11.2	49588255	8q11.2	50397319	mouse	809064
8	8q12	56285211	8q12	56386621	mouse	101410
8	8q12	62430547	8q12	62430747	mouse	200
8	8q12	63737128	8q12	63799252	mouse	62124
8	8q13	67059557	8q13	67074574	mouse	15017
8	8q21.1	75849464	8q21.1	75921688	mouse	72224
8	8q21.2	86722830	8q21.2	86725182	mouse	2352
8	8q22.1	96908031	8q22.1	97082832	mouse	174801
9	9p24	6655150	9p24	6733134	mouse	77984
9	9p21	27287159	9p21	27315182	mouse	28023
9	9q21.2	76348435	9q21.2	76566130	mouse	217695
9	9q21.3	79707435	9q21.3	79787665	mouse	80230
9	9q21.3	83878430	9q21.3	84333496	mouse	455066
9	9q22.1	88142795	9q22.1	88326888	mouse	184093
9	9q22.2	90365482	9q22.2	90655572	mouse	290090
9	9q22.3	93266234	9q22.3	93496288	mouse	230054
9	9q33	116942209	9q33	116993785	mouse	51576
9	9q33	121409752	9q33	121409952	mouse	200
9	9q33	124499927	9q33	124500127	mouse	200
9	9q34.1	127752163	9q34.1	127775856	mouse	23693
9	9q34.1	129390603	9q34.1	129486460	mouse	95857
9	9q34.1	131919071	9q34.1	131941434	mouse	22363
10	10p15	6009060	10p15	6065821	mouse	56761
10	10p13	15603605	10p13	15625119	mouse	21514
10	10p12.1	27681482	10p12.1	27701190	mouse	19708
10	10p12.1	29062204	10p12.1	29101116	mouse	38912
10	10p11.2	33217351	10p11.2	33217551	mouse	200
10	10p11.2	35326646	10p11.2	35775767	mouse	449121
10	10q11.2	45932301	10q11.2	46803496	mouse	871195
10	10q11.2	51487686	10q11.2	51895116	mouse	407430
10	10q21.1	54461632	10q21.1	55050903	mouse	589271
10	10q22.2	74749467	10q22.2	74762588	mouse	13121
10	10q22.3	81955250	10q22.3	81955450	mouse	200
10	10q23.2	89096592	10q23.3	89391382	mouse	294790
10	10q26.1	121315237	10q26.1	121315437	mouse	200
11	11p15.5	3213050	11p15.4	3590341	mouse	377291
11	11p15.1	17495811	11p15.1	17496011	mouse	200

11	11p14	25978536	11p14	26249523	mouse	270987
11	11q12	58001448	11q12	58444956	mouse	443508
11	11q13.3	63137398	11q13.1	64448975	mouse	1311577
11	11q13.3	68957204	11q13.3	69019897	mouse	62693
11	11q13.4	71432282	11q13.4	71849811	mouse	417529
11	11q14.3	89511911	11q14.3	90047433	mouse	535522
11	11q22.3	107468989	11q22.3	107486943	mouse	17954
12	12p13.3	2682266	12p13.3	2784404	mouse	102138
12	12p13.3	9184429	12p13.3	9281368	mouse	96939
12	12q13.2	54789653	12q13.3	55073321	mouse	283668
12	12q23	104257973	12q23	104292389	mouse	34416
12	12q24.1	108109684	12q24.1	108258437	mouse	148753
12	12q24.1	110367340	12q24.1	110388095	mouse	20755
12	12q24.31	121280245	12q24.31	121340361	mouse	60116
13	13q12.1	22293650	13q12.1	25715171	mouse	3421521
13	13q13	33271776	13q13	33424754	mouse	152978
13	13q14.1	40183356	13q14.1	40442833	mouse	259477
13	13q14.2	48735681	13q14.2	49140862	mouse	405181
13	13q14.3	51291453	13q14.3	51683386	mouse	391933
13	13q33	101982034	13q33	102373168	mouse	391134
14	14q11.2	23133159	14q11.2	23148394	mouse	15235
14	14q22	50233231	14q21	50679010	mouse	445779
14	14q22	56613453	14q22	56656961	mouse	43508
15	15q13	25992349	15q13	26827749	mouse	835400
15	15q13	27946389	15q13	28776847	mouse	830458
15	15q13	30021254	15q13	30513217	mouse	491963
15	15q24	73595971	15q24	73678129	mouse	82158
15	15q24	75746357	15q24	75868786	mouse	122429
15	15q25	77705431	15q25	77846292	mouse	140861
15	15q25	80141631	15q25	80799598	mouse	657967
15	15q25	82356077	15q25	83446324	mouse	1090247
15	15q25	89148393	15q26.1	89191697	mouse	43304
16	16p13.3	3238113	16p13.3	3284805	mouse	46692
16	16p12	16153065	16p13.1	16658070	mouse	505005
16	16p12	18128289	16p12	18652265	mouse	523976
16	16q22	69711086	16q22	69922705	mouse	211619
16	16q23	74093638	16q23	74225528	mouse	131890
17	17p13	4482673	17p13	4525493	mouse	42820
17	17p13	6691646	17p13	6793483	mouse	101837
17	17p11.2	16499527	17p11.2	16867611	mouse	368084
17	17q11.2	28705665	17q12	28911318	mouse	205653
17	17q12	34312844	17q12	34569236	mouse	256392
17	17q21.2	35922531	17q21.2	36321940	mouse	399409
17	17q21.3	45860244	17q21.3	46015760	mouse	155516
17	17q23	58383936	17q23	58548204	mouse	164268
17	17q24	60630038	17q24	60718303	mouse	88265
17	17q24	63138272	17q24	63371415	mouse	233143
17	17q24	66677334	17q24	66698486	mouse	21152
18	18p11.32	2458344	18p11.32	2533338	mouse	74994
18	18p11.2	9962250	18p11.2	11581492	mouse	1619242
18	18q12.3	40945714	18q12.3	41245003	mouse	299289
18	18q21.3	54027458	18q21.3	54053769	mouse	26311
18	18q21.3	58048268	18q21.3	58120930	mouse	72662
18	18q22	65726427	18q22	66127867	mouse	401440
19	19p13.3	4164504	19p13.3	4171198	mouse	6694
19	19p13.2	7004895	19p13.2	7052262	mouse	47367
19	19p13.2	8217865	19p13.2	8764217	mouse	546352
19	19p13.2	12423093	19p13.2	12602655	mouse	179562

19	19p13.1	14516393	19p13.1	17014817	mouse	2498424
19	19q12	34763281	19q12	34775525	mouse	12244
19	19q13.3	53019374	19q13.3	53476245	mouse	456871
19	19q13.4	56776884	19q13.4	59253187	mouse	2476303
20	20p13	1390878	20p13	1695482	mouse	304604
21	21q22.3	42332141	21q22.3	42385548	mouse	53407
21	21q22.3	44025134	21q22.3	44025334	mouse	200
22	22q11.2	20662815	22q11.2	21164977	mouse	502162
22	22q11.2	23328753	22q11.2	23526943	mouse	198190
22	22q12.1	27480781	22q12.1	27493144	mouse	12363
22	22q12.2	30335430	22q12.3	31990642	mouse	1655212
22	22q12.3	34221854	22q12.3	34240002	mouse	18148
X	Xp22.11	23051645	Xp22.11	23052643	mouse	998
X	Xp21.1	36457204	Xp21.1	36457404	mouse	200
X	Xp11.3	46711758	Xp11.3	46778868	mouse	67110
X	Xp11.23	48895270	Xp11.23	48895407	mouse	137
X	Xp11.22	50082060	Xp11.22	51759653	mouse	1677593
X	Xq21.1	81290506	Xq21.1	81326323	mouse	35817
X	Xq21.31	91417469	Xq21.31	91789772	mouse	372303
X	Xq23	112595429	Xq23	113134875	mouse	539446
1	1p34.2	40197771	1p34.2	41703838	pig	1506067
1	1p31.1	83798949	1p22	85249768	pig	1450819
1	1q24	167431107	1q24	168874873	pig	1443766
1	1q25	181731277	1q31	184648713	pig	2917436
1	1q31	190994647	1q31	193870329	pig	2875682
1	1q32	198449609	1q32	200753410	pig	2303801
1	1q32	202039491	1q32	205001837	pig	2962346
1	1q32	210456462	1q41	212376628	pig	1920166
1	1q41	219283155	1q42	220460016	pig	1176861
1	1q42	220752892	1q42	221406525	pig	653633
1	1q43	236369159	1q43	237965945	pig	1596786
1	1q43	238022215	1q43	239590636	pig	1568421
2	2q13	112349922	2q14.1	115217387	pig	2867465
2	2q14.2	119249570	2q14.2	120264932	pig	1015362
2	2q14.3	126294676	2q14.3	129361071	pig	3066395
2	2q21.1	129726694	2q21.2	133544712	pig	3818018
2	2q23	150240956	2q23	153535876	pig	3294920
3	3p25	14703453	3p25	15701225	pig	997772
3	3p12	75044362	3p12	75908326	pig	863964
3	3q21	126435252	3q21	128404641	pig	1969389
3	3q21	129808134	3q21	131252843	pig	1444709
3	3q22	133084059	3q22	133494552	pig	410493
4	4p16	3429821	4p16	4521317	pig	1091496
4	4p16	8358037	4p16	9765343	pig	1407306
4	4q12	56080333	4q12	56537476	pig	457143
4	4q21.1	78265810	4q21.2	81743084	pig	3477274
4	4q32	156602424	4q32	156742252	pig	139828
4	4q32	156762371	4q32	157109370	pig	346999
4	4q32	165895860	4q32	166517499	pig	621639
4	4q32	167376267	4q32	168238991	pig	862724
4	4q34	176027692	4q34	177139467	pig	1111775
4	4q35	187764074	4q35	188039292	pig	275218
5	5p15.3	9816235	5p15.2	11484715	pig	1668480
5	5p15.1	15881962	5p15.1	17433872	pig	1551910
5	5q12	63253626	5q13.1	64531560	pig	1277934
5	5q13.2	71841995	5q13.3	73499061	pig	1657066
5	5q33.1	150116348	5q33.1	151807934	pig	1691586
6	6p21.3	33972374	6p21.2	37822747	pig	3850373

6	6p12	51972043	6p12	53884962	pig	1912919
6	6p12	63665829	6q12	64869526	pig	1203697
6	6q13	73338720	6q13	74463003	pig	1124283
6	6q14	83886431	6q14	85181860	pig	1295429
6	6q22.1	116138228	6q22.1	116967010	pig	828782
7	7p22	6441062	7p21	7627247	pig	1186185
7	7p15.3	23255428	7p15.3	23533384	pig	277956
7	7p14	37089533	7p14	38359861	pig	1270328
7	7p14	38359955	7p14	41311384	pig	2951429
7	7p12	47724972	7p12	49061384	pig	1336412
7	7p11.2	54932101	7p11.2	55187352	pig	255251
7	7p11.2	55187528	7p11.2	55850025	pig	662497
7	7q11.22	66101340	7q11.22	67528444	pig	1427104
7	7q11.23	71047205	7q11.23	72247662	pig	1200457
7	7q11.23	75109793	7q11.23	76423878	pig	1314085
7	7q21.1	86671814	7q21.1	88158441	pig	1486627
7	7q21.3	96965209	7q21.3	97627709	pig	662500
7	7q22	101774951	7q22	102813928	pig	1038977
7	7q31.1	107467978	7q31.1	108915189	pig	1447211
7	7q34	142089425	7q35	143474392	pig	1384967
7	7q36	148209911	7q36	149974087	pig	1764176
8	8p23.1	6230255	8p23.1	8651658	pig	2421403
8	8p23.1	8651735	8p23.1	9853036	pig	1201301
8	8p23.1	11567139	8p22	12890783	pig	1323644
8	8p22	16731148	8p22	17694436	pig	963288
8	8p22	17694591	8p22	18346899	pig	652308
8	8p22	18347274	8p21	19606244	pig	1258970
8	8p21	28742821	8p12	29431481	pig	688660
8	8p12	38581060	8p11.2	40042274	pig	1461214
9	9p21	26715212	9p21	27647434	pig	932222
9	9p13	34121663	9p13	35536320	pig	1414657
9	9q21.3	78377606	9q21.3	79695354	pig	1317748
9	9q21.3	82892439	9q21.3	85248039	pig	2355600
9	9q22.1	87692667	9q22.1	88339269	pig	646602
9	9q22.3	92303697	9q22.3	93700832	pig	1397135
10	10p14	10877661	10p13	13267035	pig	2389374
10	10p12.3	18876007	10p12.3	19796916	pig	920909
10	10p12.1	26608481	10p12.1	28183041	pig	1574560
10	10p11.2	32263726	10p11.2	33523600	pig	1259874
10	10q11.2	50573505	10q11.2	51939578	pig	1366073
10	10q23.2	88326768	10q23.3	89470536	pig	1143768
11	11p15.5	3009963	11p15.4	5489543	pig	2479580
11	11p15.4	8973254	11p15.4	10367590	pig	1394336
11	11q13.3	70729241	11q13.4	72154608	pig	1425367
12	12q13.1	47699745	12q13.1	49057481	pig	1357736
12	12q21.2	76569350	12q21.2	77242591	pig	673241
12	12q23	105469295	12q23	106667370	pig	1198075
12	12q23	107089525	12q24.1	108559454	pig	1469929
13	13q14.1	40176697	13q14.1	41098351	pig	921654
13	13q14.3	51972222	13q14.3	52631199	pig	658977
14	14q13	36324614	14q21	37751799	pig	1427185
14	14q23	61777163	14q23	62931020	pig	1153857
15	15q13	29402166	15q13	30743993	pig	1341827
15	15q23	69197530	15q24	70944247	pig	1746717
15	15q24	74986799	15q25	76456570	pig	1469771
15	15q25	83207550	15q25	84671483	pig	1463933
15	15q25	86128267	15q26.1	87751893	pig	1623626
15	15q25	87751992	15q26.1	89487902	pig	1735910

15	15q26.3	96367453	15q26.3	97577819	pig	1210366
16	16q12.2	54868307	16q13	56526953	pig	1658646
16	16q22	68066139	16q22	69282946	pig	1216807
17	17p11.2	17657721	17p11.2	20549774	pig	2892053
17	17q21.1	35262313	17q21.2	36392035	pig	1129722
17	17q21.3	45255531	17q21.3	46766362	pig	1510831
17	17q24	60291685	17q24	61687279	pig	1395594
18	18q12.3	40509868	18q21.1	42425486	pig	1915618
18	18q21.3	54938704	18q21.3	56271424	pig	1332720
20	20p13	2630616	20p13	4787917	pig	2157301
22	22q11.2	16689189	22q11.2	17426974	pig	737785
22	22q11.2	22450228	22q11.2	23710146	pig	1259918
22	22q12.2	29660438	22q12.3	31522202	pig	1861764
1	1p36.1	26032778	1p36.1	26124267	rat	91489
1	1p36.1	27828854	1p35	28515209	rat	686355
1	1p32	58369437	1p32	58479571	rat	110134
1	1p31.2	66969868	1p31.2	67947982	rat	978114
1	1p22	89073417	1p22	89186870	rat	113453
1	1p22	93175146	1p22	93258183	rat	83037
1	1q23	154930185	1q23	155307838	rat	377653
1	1q32	204176920	1q32	204247249	rat	70329
1	1q42	220819436	1q42	220822552	rat	3116
1	1q42	222372946	1q42	222417687	rat	44741
1	1q42	224015581	1q42	225323048	rat	1307467
1	1q42	231579499	1q42	232260182	rat	680683
1	1q43	236371426	1q43	236517934	rat	146508
2	2p25.3	3119186	2p25.3	3235032	rat	115846
2	2p22	35636081	2p22	35766488	rat	130407
2	2p13	68650881	2p13	68652166	rat	1285
2	2p13	70975429	2p13	71005286	rat	29857
2	2p13	73965750	2p13	74013786	rat	48036
2	2q13	113552965	2q14.1	114343565	rat	790600
2	2q14.3	122430979	2q14.3	123481484	rat	1050505
2	2q14.3	124108818	2q14.3	124109018	rat	200
2	2q14.3	124817492	2q14.3	125203982	rat	386490
2	2q14.3	127021977	2q14.3	127091697	rat	69720
2	2q14.3	128691458	2q14.3	128726070	rat	34612
2	2q21.1	131934198	2q21.2	133161439	rat	1227241
2	2q22	138624341	2q22	139955364	rat	1331023
2	2q32.1	188356222	2q32.1	188704047	rat	347825
3	3p25	12864965	3p25	12997408	rat	132443
3	3p25	15135116	3p24	16857271	rat	1722155
3	3p24	20077802	3p24	21184398	rat	1106596
3	3p24	27569115	3p24	27605472	rat	36357
3	3p22	37074253	3p22	37082950	rat	8697
3	3p21.3	46267172	3p21.3	46267372	rat	200
3	3p21.2	52076095	3p21.2	52207798	rat	131703
3	3p14.3	57767106	3p14.3	57799917	rat	32811
3	3p14.1	63864176	3p14.1	63872656	rat	8480
3	3p12	75103631	3p12	75681020	rat	577389
3	3q21	126603810	3q21	127260234	rat	656424
3	3q21	130317226	3q21	131213237	rat	896011
3	3q24	149369030	3q24	150339280	rat	970250
3	3q26.1	169224071	3q26.2	169304900	rat	80829
3	3q26.2	170409406	3q26.2	170471630	rat	62224
3	3q26.3	173034682	3q26.3	173196601	rat	161919
3	3q26.3	179507657	3q26.3	179525339	rat	17682
3	3q27	184219239	3q26.3	184255517	rat	36278

3	3q29	196679158	3q29	196836695	rat	157537
4	4p16	3792758	4p16	4200472	rat	407714
4	4p16	8859586	4p16	9502717	rat	643131
4	4q22	89376336	4q22	89376536	rat	200
4	4q22	95664969	4q22	95684734	rat	19765
4	4q26	121086934	4q27	121332486	rat	245552
4	4q27	122837455	4q27	122837655	rat	200
4	4q31.1	141655864	4q31.1	141717796	rat	61932
4	4q31.2	151353028	4q31.2	151432923	rat	79895
4	4q32	163624597	4q32	163977702	rat	353105
5	5p15.3	7932916	5p15.3	7963957	rat	31041
5	5q15	96161776	5q15	96215463	rat	53687
5	5q21	98424895	5q21	98425095	rat	200
5	5q22	110087815	5q22	110330256	rat	242441
5	5q22	112325253	5q22	112339704	rat	14451
5	5q23.3	130393520	5q23.3	130515065	rat	121545
5	5q31.1	134075456	5q31.1	134105170	rat	29714
5	5q31.1	137121696	5q31.1	137256947	rat	135251
5	5q32	147607546	5q32	147631363	rat	23817
5	5q33.1	150210048	5q33.1	150367789	rat	157741
5	5q33.2	154317378	5q33.2	154317578	rat	200
5	5q35	173646386	5q35	173698426	rat	52040
5	5q35	177105027	5q35	177490456	rat	385429
6	6p22.3	20093794	6p22.3	20127761	rat	33967
6	6p22.1	29101707	6p22.1	29140900	rat	39193
6	6p21.2	39059211	6p21.2	39329540	rat	270329
6	6p12	52755093	6p12	52774746	rat	19653
6	6p12	55784286	6p12	56226463	rat	442177
6	6q13	73859514	6q13	74013463	rat	153949
6	6q14	86415489	6q14	87745595	rat	1330106
6	6q16.3	100244557	6q16.3	100628426	rat	383869
6	6q22.1	116674806	6q22.1	116937701	rat	262895
6	6q22.3	123081845	6q22.3	123117471	rat	35626
6	6q22.3	127848358	6q22.3	127948896	rat	100538
6	6q23.2	135138941	6q23.2	135157113	rat	18172
6	6q25.1	150165506	6q25.1	150166635	rat	1129
7	7p22	5502967	7p22	6801465	rat	1298498
7	7p21	12240731	7p21	12274933	rat	34202
7	7p21	19459102	7p21	19463760	rat	4658
7	7p15.3	22306265	7p15.3	22964127	rat	657862
7	7p14	32734941	7p14	32735141	rat	200
7	7p14	36046504	7p14	36231138	rat	184634
7	7p13	43312412	7p13	43623045	rat	310633
7	7q11.23	71402623	7q11.23	72124837	rat	722214
7	7q11.23	73887786	7q11.23	76415937	rat	2528151
7	7q21.3	97029493	7q21.3	97265749	rat	236256
7	7q22	99027359	7q22	101963289	rat	2935930
7	7q22	104742436	7q22	104755479	rat	13043
7	7q31.1	107320004	7q31.1	107322860	rat	2856
7	7q31.1	111678926	7q31.1	111686343	rat	7417
7	7q36	149864544	7q36	149903378	rat	38834
7	7q36	156529297	7q36	156553362	rat	24065
8	8p23.1	7500178	8p23.1	7981888	rat	481710
8	8p23.1	9510886	8p23.1	9579292	rat	68406
8	8p23.1	11711648	8p23.1	12355545	rat	643897
8	8p22	17763927	8p22	17802310	rat	38383
8	8p21	19985979	8p21	20008284	rat	22305
8	8p12	28970543	8p12	29011057	rat	40514

8	8p12	36492063	8p12	36496263	rat	4200
8	8p12	37734651	8p12	37734851	rat	200
8	8q12	62410873	8q12	62417430	rat	6557
8	8q12	63721790	8q12	63806443	rat	84653
8	8q13	67053743	8q13	67053943	rat	200
8	8q21.1	75892926	8q21.1	75921289	rat	28363
8	8q21.2	86714895	8q21.2	86722524	rat	7629
8	8q22.1	96911668	8q22.1	96916524	rat	4856
9	9p24	6655150	9p24	6733134	rat	77984
9	9p21	27287159	9p21	27315090	rat	27931
9	9q21.2	76347373	9q21.2	76563748	rat	216375
9	9q21.3	79693982	9q21.3	79793107	rat	99125
9	9q21.3	83878609	9q21.3	84125800	rat	247191
9	9q22.2	88142780	9q22.1	88302235	rat	159455
9	9q22.2	90365402	9q22.2	90648715	rat	283313
9	9q22.3	93280543	9q22.3	93496294	rat	215751
9	9q33	116942049	9q33	116994930	rat	52881
9	9q33	121405175	9q33	121405737	rat	562
9	9q33	124512769	9q33	124533010	rat	20241
9	9q34.1	127758645	9q34.1	127848066	rat	89421
9	9q34.1	129385133	9q34.1	129454199	rat	69066
9	9q34.1	131919050	9q34.1	131962047	rat	42997
10	10p12.1	27676319	10p12.1	27737515	rat	61196
10	10p12.1	29062258	10p11.2	29906905	rat	844647
10	10p11.2	31026991	10p11.2	31103607	rat	76616
10	10p11.2	32605919	10p11.2	32895213	rat	289294
10	10p11.2	35324029	10p11.2	35770605	rat	446576
10	10q11.2	46002988	10q11.2	46482643	rat	479655
10	10q11.2	51472743	10q11.2	51895360	rat	422617
10	10q21.1	54432491	10q21.1	55049876	rat	617385
10	10q21.3	68040493	10q21.3	68145137	rat	104644
10	10q22.1	70347467	10q21.3	70373196	rat	25729
10	10q22.1	74077294	10q22.2	74762933	rat	685639
10	10q22.3	79620307	10q22.3	79633405	rat	13098
10	10q22.3	81955638	10q22.3	81955838	rat	200
10	10q23.2	89041450	10q23.3	89391808	rat	350358
10	10q24.1	99061758	10q24.2	99424005	rat	362247
10	10q24.2	101662335	10q24.2	101763891	rat	101556
10	10q26.1	121313866	10q26.1	121314066	rat	200
11	11p15.5	3213050	11p15.4	3590341	rat	377291
11	11p15.1	17406125	11p15.1	17417333	rat	11208
11	11p14	25987264	11p14	26247249	rat	259985
11	11q12	58011814	11q12	58065769	rat	53955
11	11q13.3	71432282	11q13.4	71849781	rat	417499
11	11q14.3	89523970	11q14.3	90045091	rat	521121
11	11q22.3	106828099	11q22.3	107486944	rat	658845
12	12p13.3	2682525	12p13.3	2783499	rat	100974
12	12p13.3	9174615	12p13.3	9282216	rat	107601
12	12q13.2	54965452	12q13.3	55441278	rat	475826
12	12q14	56862885	12q14	56948908	rat	86023
12	12q24.1	108109650	12q24.1	108258446	rat	148796
12	12q24.1	110355800	12q24.1	110410425	rat	54625
12	12q24.31	121274782	12q24.31	121375704	rat	100922
13	13q12.1	22757233	13q12.1	25724393	rat	2967160
13	13q13	33345993	13q13	33401983	rat	55990
13	13q14.1	40188271	13q14.1	40426525	rat	238254
13	13q14.2	48715417	13q14.2	49130912	rat	415495
13	13q14.3	51291706	13q14.3	52174714	rat	883008

13	13q33	101917852	13q33	102376524	rat	458672
14	14q11.2	23137497	14q11.2	23141966	rat	4469
14	14q22	50241915	14q21	50686858	rat	444943
14	14q22	56619173	14q22	56657935	rat	38762
15	15q13	26033210	15q13	26807344	rat	774134
15	15q13	27946401	15q13	28553095	rat	606694
15	15q13	29997953	15q13	30504576	rat	506623
15	15q14	32238453	15q14	32516158	rat	277705
15	15q21.1	48877128	15q21.1	49543926	rat	666798
15	15q24	73600262	15q24	73683939	rat	83677
15	15q24	75763740	15q24	75869872	rat	106132
15	15q25	77858828	15q25	77875892	rat	17064
15	15q25	80152765	15q25	80799598	rat	646833
15	15q25	82374406	15q25	83433609	rat	1059203
15	15q25	89153038	15q26.1	89189475	rat	36437
16	16p13.1	15528691	16p13.1	16628631	rat	1099940
16	16p12	18203373	16p12	18738281	rat	534908
16	16q13	55602137	16q13	55614699	rat	12562
16	16q22	66704022	16q22	66730663	rat	26641
16	16q22	69711086	16q22	69863578	rat	152492
16	16q23	74093662	16q23	74169441	rat	75779
17	17p13	4482673	17p13	4525491	rat	42818
17	17p13	6691571	17p13	6748387	rat	56816
17	17q11.2	27510680	17q12	30313282	rat	2802602
17	17q12	34303780	17q12	34573463	rat	269683
17	17q21.2	35922531	17q21.2	36319889	rat	397358
17	17q21.3	44302688	17q21.3	45911385	rat	1608697
17	17q23	58283088	17q23	58548204	rat	265116
17	17q24	60629869	17q24	60718284	rat	88415
17	17q24	63138270	17q24	63380818	rat	242548
17	17q24	66449451	17q24	66733287	rat	283836
18	18p11.2	9962013	18p11.2	11658515	rat	1696502
18	18q12.3	40945714	18q12.3	41245003	rat	299289
18	18q21.3	53943062	18q21.3	54053467	rat	110405
18	18q21.3	58048268	18q21.3	58119068	rat	70800
18	18q22	65639416	18q22	66443224	rat	803808
19	19p13.3	4164193	19p13.2	7054475	rat	2890282
19	19p13.2	8217968	19p13.2	9237754	rat	1019786
19	19p13.2	11509875	19p13.2	12602657	rat	1092782
19	19p13.1	14528147	19p13.1	14791338	rat	263191
19	19p13.1	15948062	19p13.1	16027834	rat	79772
19	19q12	34786782	19q12	34786982	rat	200
19	19q13.3	53042981	19q13.3	53477270	rat	434289
19	19q13.4	56776887	19q13.4	59398235	rat	2621348
20	20p13	1390268	20p13	1695883	rat	305615
21	21q22.3	42332141	21q22.3	42385504	rat	53363
22	22q12.1	27480724	22q12.1	27493144	rat	12420
22	22q12.3	30824528	22q12.3	31992586	rat	1168058
22	22q12.3	34221854	22q12.3	34241294	rat	19440
X	Xp22.11	23022644	Xp22.11	23052913	rat	30269
X	Xp21.1	36161279	Xp21.1	36214921	rat	53642
X	Xp11.3	46346466	Xp11.3	46900013	rat	553547
X	Xp11.23	48894439	Xp11.22	51759026	rat	2864587
X	Xq22.3	105353823	Xq22.3	105436249	rat	82426
X	Xq23	113262020	Xq23	113413379	rat	151359
X	Xq25	123820873	Xq25	124040790	rat	219917
