# Supplementary material for: Is mammalian chromosomal evolution driven by regions of genome fragility?
Source: Genome Biol. 2006 Dec 8;7(12):R115. doi: 10.1186/gb-2006-7-12-r115 (PMC1794428; doi:10.1186/gb-2006-7-12-r115)
Supplement: Additional data file 6 — List of evolutionary chromosomal bands detected. [file gb-2006-7-12-r115-S6.pdf]

Table S3: Evolutionary chromosomal bands detected.

Evolutionary chromosomal band	from	to	nº evolutionary breakpoints	species	fs/no fs	type fs
10p11.2	29,401,000	38,801,000	28	cattle mouse pig rat dog	no fs	
10p12.1	24,599,001	29,401,000	10	dog mouse pig rat	no fs	
10p12.3	17,301,000	22,601,000	4	cat pig	no fs	
10p13	12,301,000	17,301,000	5	mouse pig + chicken	no fs	
10p14	6,699,001	12,301,000	2	pig + chicken	no fs	
10p15	1	6,701,000	5	mouse + chicken	no fs	
10q11.2	41,899,001	52,601,000	30	cattle mouse pig rat dog + chicken	fs	common
10q21.1	52,599,001	60,801,000	12	cattle mouse rat + chicken	fs	common
10q21.2	60,801,000	64,201,000	2	cattle + chicken	fs	common
10q21.3	64,201,000	70,301,000	5	rat + chicken	fs	common
10q22.1	70,301,000	74,601,000	3	rat + chicken	fs	common
10q22.2	74,601,000	77,699,001	4	mouse rat + chicken	no fs	
10q22.3	77,699,001	82,901,000	9	mouse rat + chicken	no fs	
10q23.1	82,901,000	86,799,001	1	chicken	no fs	
10q23.2	86,799,001	89,101,000	4	mouse pig rat + chicken	no fs	
10q23.3	89,101,000	96,999,001	8	mouse pig rat + chicken	fs	rare
10q24.1	96,999,001	99,299,001	7	rat + chicken	no fs	
10q24.2	99,299,001	101,901,000	4	rat + chicken	no fs	
10q24.3	101,901,000	105,699,001	10	cat + chicken	no fs	
10q25.1	105,699,001	111,801,000	1	cat	no fs	
10q26.1	118,699,001	127,401,000	5	mouse rat + chicken	fs	common
10q26.2	127,401,000	130,501,000	1	chicken	no fs	
11p11.12	48,801,000	51,500,000	2	chicken	no fs	
11p11.2	43,399,001	48,801,000	2	dog	no fs	
11p13	31,001,000	36,401,000	4	cat + chicken	fs	common
11p14	21,601,000	31,001,000	8	cattle mouse rat dog	fs (11p14.2)	common
11p15.1	16,101,000	21,601,000	8	mouse rat cattle + chicken	fs	common/rare
11p15.2	12,601,000	16,101,000	4	chicken	no fs	
11p15.4	2,799,001	10,701,000	17	dog pig cat cattle horse mouse rat + chicken	no fs	

11p15.5	1	2,799,001	11	dog pig cat cattle horse mouse rat + chicken	no fs	
11q12	56,699,001	63,101,000	10	cattle mouse rat dog	no fs	
11q13.1	63,101,000	65,601,000	1	mouse	fs	common
11q13.3	68,299,001	70,001,000	7	cat cattle mouse pig rat	fs	common/rare
11q13.4	70,001,000	74,899,001	12	cat cattle mouse pig rat dog + chicken	fs	common
11q13.5	74,899,001	77,401,000	3	cattle + chicken	fs	common
11q14.1	77,401,000	85,301,000	5	cattle	no fs	
11q14.3	87,799,001	92,101,000	4	mouse rat	no fs	
11q22.1	96,399,001	101,201,000	3	cat dog	no fs	
11q22.2	101,201,000	102,399,001	1	cat	no fs	
11q22.3	102,399,001	110,001,000	7	cattle mouse rat + chicken	no fs	
11q23.1	110,001,000	112,701,000	1	chicken	no fs	
11q24	121,199,001	130,301,000	2	cattle	no fs	
12p11.2	26,399,001	33,201,000	4	chicken	no fs	
12p13.3	1	10,101,000	10	dog mouse rat	no fs	
12q13.1	44,599,001	53,101,000	2	pig	fs	rare
12q13.2	53,101,000	54,899,001	5	cattle dog mouse rat	no fs	
12q13.3	54,899,001	56,301,000	3	cattle mouse rat	no fs	
12q14	56,301,000	66,101,000	2	rat	no fs	
12q21.1	70,099,001	74,301,000	2	dog	no fs	
12q21.2	74,301,000	78,901,000	2	pig	no fs	
12q22	91,199,001	94,701,000	1	cattle	no fs	
12q23	94,701,000	107,501,000	14	mouse pig cattle dog + chicken	no fs	
12q24.1	107,501,000	112,801,000	13	mouse rat pig dog + chicken	fs	common/rare
12q24.31	119,299,001	125,401,000	6	mouse rat + chicken	fs	common
13q12.1	18,399,001	26,601,000	6	cattle mouse rat	no fs	
13q13	31,099,001	39,001,000	4	mouse rat	fs	common
13q14.1	39,001,000	46,199,001	10	horse mouse pig rat dog	no fs	
13q14.2	46,199,001	49,801,000	4	mouse rat	no fs	
13q14.3	49,801,000	54,201,000	10	dog mouse pig rat	no fs	
13q31	77,899,001	93,801,000	2	chicken	no fs	
13q33	100,499,001	109,101,000	6	mouse rat + chicken	no fs	
14q11.2	18,999,001	24,301,000	6	dog mouse rat	no fs	
14q13	32,399,001	36,901,000	1	pig	no fs	

14q21	36,901,000	50,099,001	4	cattle mouse pig rat	no fs	
14q22	50,099,001	57,201,000	9	cattle mouse rat	no fs	
14q23	57,201,000	67,001,000	2	pig	fs	common
14q32.3	100,399,001	106,368,585	2	chicken	no fs	
15q11.2	18,299,001	23,201,000	6	dog + chicken	no fs	
15q12	23,201,000	25,701,000	1	chicken	no fs	
15q13	25,701,000	31,399,001	28	cat cattle mouse pig rat dog + chicken	no fs	
15q14	31,399,001	37,901,000	2	rat	no fs	
15q15	37,901,000	42,601,000	10	cattle + chicken	no fs	
15q21.1	42,601,000	47,501,000	6	rat + chicken	no fs	
15q22.3	61,499,001	65,301,000	6	cattle + chicken	fs	common
15q23	65,301,000	70,399,001	6	cattle pig + chicken	no fs	
15q24	70,399,001	76,101,000	19	cat mouse pig rat dog + chicken	no fs	
15q25	76,101,000	86,899,001	33	cat cattle mouse pig rat dog + chicken	no fs	
15q26.1	86,899,001	92,101,000	10	dog mouse pig rat + chicken	no fs	
15q26.3	96,299,001	100,338,915	3	dog pig	no fs	
16p11.2	27,601,000	34,401,000	7	dog + chicken	no fs	
16p12	16,701,000	27,601,000	7	mouse rat + chicken	fs (16p12.1)	rare
16p13.1	10,299,001	16,701,000	8	mouse rat + chicken	fs	rare
16p13.3	1	6,301,000	16	mouse + chicken	no fs	
16q12.1	45,499,001	51,201,000	2	dog	no fs	
16q12.2	51,201,000	55,301,000	1	pig	no fs	
16q13	55,301,000	56,701,000	3	rat pig	no fs	
16q21	56,701,000	63,899,001	4	dog + chicken	no fs	
16q22	63,899,001	73,801,000	17	cattle mouse pig rat + chicken	fs (16q22.1)	common/rare
16q23	73,801,000	82,701,000	9	cattle mouse rat + chicken	no fs	
17p11.2	15,899,001	22,101,000	8	dog mouse pig + chicken	no fs	
17p12	10,699,001	15,899,001	2	chicken	fs	rare
17p13	1	10,699,001	20	mouse rat dog + chicken	no fs	
17q11.2	22,799,001	28,801,000	8	cattle mouse rat dog + chicken	no fs	
17q12	28,799,001	35,201,000	8	dog mouse rat	no fs	
17q21.1	35,201,000	35,599,001	1	pig	no fs	
17q21.2	35,599,001	38,201,000	5	mouse rat pig	no fs	
17q21.3	38,201,000	47,501,000	14	cat cattle mouse pig rat + chicken	no fs	

17q23	50,899,001	59,899,001	12	cat cattle mouse rat + chicken	fs (17q23.1)	common
17q24	59,899,001	68,401,000	24	cattle horse mouse rat cat dog pig	no fs	
17q25	68,401,000	78,774,742	4	chicken	no fs	
18p11.2	7,199,001	15,401,000	12	mouse rat dog + chicken	no fs	
18p11.32	1	2,901,000	2	mouse	no fs	
18q11.2	17,299,001	23,301,000	2	dog + chicken	no fs	
18q12.2	30,999,001	35,501,000	5	cattle + chicken	fs	common
18q12.3	35,501,000	41,801,000	6	cattle mouse pig rat	no fs	
18q21.1	41,801,000	48,501,000	9	cattle dog pig + chicken	no fs	
18q21.3	52,499,001	59,801,000	11	mouse pig rat + chicken	fs	common
18q22	59,801,000	71,299,001	7	cattle mouse rat + chicken	fs (18q22.1)	rare
18q23	71,299,001	76,117,153	2	chicken	no fs	
19p12	19,801,000	26,701,000	2	cat + chicken	no fs	
19p13.1	12,599,001	19,801,000	11	cat cattle mouse rat + chicken	fs	rare
19p13.2	6,901,000	12,599,001	13	mouse rat cattle + chicken	fs	rare
19p13.3	1	6,901,000	10	cattle mouse rat + chicken	fs	rare
19q12	30,199,001	37,101,000	6	cattle mouse rat	no fs	
19q13.1	37,101,000	43,399,001	1	chicken	fs	common
19q13.2	43,399,001	48,101,000	1	chicken	fs	common
19q13.3	48,101,000	55,999,001	5	cat mouse rat	fs	common
19q13.4	55,999,001	63,811,651	5	mouse rat cat	fs	common
1p13.1	114,899,001	117,501,000	1	chicken	no fs	
1p13.2	111,501,000	114,899,001	6	dog + chicken	no fs	
1p13.3	106,899,001	111,501,000	3	chicken	no fs	
1p21.1	101,999,001	106,899,001	2	chicken	no fs	
1p22.1	91,699,001	94,501,000	4	mouse rat	fs	common
1p22.2	88,101,000	91,699,001	4	mouse rat	fs	common
1p22.3	84,599,001	88,101,000	1	pig	fs	common
1p31.1	69,499,001	84,599,001	4	pig + chicken	fs	common
1p31.2	65,499,001	69,499,001	7	dog mouse rat + chicken	fs	common
1p32.3	50,399,001	55,801,000	2	dog	fs	common
1p32.2	55,801,000	58,701,000	4	mouse rat	fs	common
1p34.2	39,799,001	43,801,000	8	dog pig + chicken	no fs	
1p34.3	34,299,001	39,799,001	5	cattle chicken	no fs	

1p35	27,699,001	34,299,001	8	cattle rat dog + chicken	no fs	
1p36.1	15,599,001	27,699,001	10	rat cattle + chicken	fs	common
1p36.2	7,101,000	15,599,001	5	cattle dog + chicken	fs	common
1p36.3	1	7,101,000	4	cattle	fs	common
1q21.3	148,099,001	151,401,000	2	dog	fs	common
1q23	153,399,001	162,301,000	7	mouse rat cat + chicken	no fs	
1q24	162,301,000	169,699,001	8	cattle pig cat	no fs	
1q25	169,699,001	182,501,000	3	cattle pig	fs (1q25.1)	common
1q31	182,501,000	196,001,000	8	cattle pig cat	fs	common
1q32.1	195,999,001	203,601,000	14	cattle pig dog + chicken	no fs	
1q32.2	203,601,000	207,799,001	10	cattle pig dog mouse rat + chicken	no fs	
1q32.3	207,799,001	210,501,000	1	pig	no fs	
1q41	210,501,000	219,799,001	2	pig	no fs	
1q42.1	219,799,001	227,001,000	28	cat cattle mouse pig rat + chicken	fs	common
1q42.3	230,999,001	232,901,000	5	mouse rat cattle	fs	common
1q43	232,899,001	239,501,000	12	cattle mouse pig rat	no fs	
1q44	239,501,000	245,522,847	8	cattle dog	fs	common
20p11.2	17,799,001	25,701,000	5	dog + chicken	fs (20p11.23)	rare
20p12	4,999,001	17,799,001	10	cattle + chicken	fs (20p12.2)	common
20p13	1	4,999,001	10	cattle mouse pig rat + chicken	no fs	
20q11.2	28,399,001	37,101,000	6	dog + chicken	no fs	
20q13.1	41,099,001	49,201,000	2	chicken	no fs	
21q11	12,299,001	15,301,000	2	dog	no fs	
21q22.1	30,499,001	38,601,000	2	cattle	no fs	
21q22.3	41,399,001	46,944,323	8	mouse rat + chicken	no fs	
22q11.2	16,299,001	24,301,000	22	cattle mouse pig dog + chicken	no fs	
22q12.1	24,301,000	27,901,000	5	mouse rat + chicken	no fs	
22q12.2	27,901,000	30,499,001	5	cattle horse mouse pig + chicken	fs	common
22q12.3	30,499,001	35,801,000	17	cattle horse mouse rat cat dog + chicken	no fs	
22q13.1	35,801,000	39,299,001	4	cat cattle	fs	common
22q13.2	39,299,001	42,601,000	3	cat + chicken	fs	common
22q13.3	42,601,000	49,554,710	4	chicken	fs	common
2p11.2	83,299,001	91,001,000	2	cattle	fs	rare
2p12	74,999,001	83,301,000	6	cattle + chicken	no fs	

2p13	68,501,000	74,999,001	27	cattle mouse rat dog + chicken	fs	common
2p14	63,999,001	68,501,000	1	cattle	no fs	
2p15	61,201,000	63,999,001	3	cattle + chicken	no fs	
2p16	47,699,001	61,201,000	8	cattle mouse + chicken	fs (2p16.2)	common
2p21	41,701,000	47,699,001	4	dog + chicken	no fs	
2p22	31,999,001	41,701,000	10	cat cattle rat + chicken	no fs	
2p23	23,999,001	31,999,001	13	cattle mouse + chicken	no fs	
2p25.3	1	3,801,000	6	mouse + chicken	no fs	
2q11.2	95,799,001	102,601,000	2	dog	fs	rare
2q12	102,601,000	108,701,000	3	mouse	no fs	
2q13	108,701,000	113,901,000	14	cattle dog mouse pig rat + chicken	fs	rare
2q14.1	113,901,000	118,601,000	7	cattle dog mouse pig rat + chicken	no fs	
2q14.2	118,601,000	122,099,001	4	pig + chicken	no fs	
2q14.3	122,099,001	129,601,000	20	mouse pig rat mouse + chicken	no fs	
2q21.1	129,599,001	132,301,000	8	cattle dog mouse pig rat + chicken	no fs	
2q21.2	132,301,000	134,899,001	7	dog mouse pig rat + chicken	no fs	
2q21.3	134,899,001	137,401,000	1	cattle	fs	common
2q22	137,401,000	148,501,000	4	mouse rat	fs (2q22.3)	rare
2q23	148,501,000	154,701,000	4	pig dog	no fs	
2q24.2	159,699,001	163,601,000	2	chicken	no fs	
2q32.1	182,799,001	189,201,000	4	mouse rat	fs	common
2q32.2	189,201,000	191,799,001	12	cattle dog mouse	no fs	
2q32.3	191,799,001	197,301,000	2	mouse	no fs	
2q33	197,301,000	208,901,000	1	chicken	fs	common
2q34	208,901,000	215,199,001	1	chicken	no fs	
2q35	215,199,001	221,401,000	4	chicken	no fs	
2q36	221,401,000	230,799,001	5	cattle dog + chicken	no fs	
2q37.1	230,799,001	235,401,000	6	cattle + chicken	no fs	
2q37.2	235,401,000	237,099,001	1	chicken	no fs	
2q37.3	237,099,001	243,018,229	6	cattle + chicken	fs	common
3p12	74,199,001	87,201,000	13	mouse pig rat dog + chicken	no fs	
3p14.1	63,699,001	69,901,000	4	mouse rat	no fs	
3p14.3	54,399,001	58,501,000	4	mouse rat	no fs	
3p21.2	51,399,001	52,301,000	6	cat rat + chicken	no fs	

3p21.3	43,599,001	51,401,000	11	cattle rat dog + chicken	no fs	
3p22	34,999,001	43,599,001	17	cattle mouse rat + chicken	no fs	
3p23	32,199,001	34,999,001	4	dog + chicken	no fs	
3p24	16,399,001	32,199,001	11	mouse rat cat	fs (3p24.2)	common
3p25	1	16,399,001	25	cattle dog mouse pig rat + chicken	no fs	
3q11.2	93,199,001	99,201,000	2	chicken	no fs	
3q12	99,201,000	104,301,000	1	chicken	no fs	
3q13.2	112,799,001	115,001,000	2	chicken	no fs	
3q13.3	115,001,000	123,301,000	1	chicken	no fs	
3q21	123,301,000	132,701,000	29	cattle chicken mouse pig rat	no fs	
3q22	132,701,000	140,399,001	10	cattle pig + chicken	no fs	
3q23	140,399,001	144,401,000	2	chicken	no fs	
3q24	144,401,000	150,401,000	8	mouse rat + chicken	no fs	
3q25.3	156,499,001	162,201,000	2	dog	fs	common
3q26.1	162,201,000	169,301,000	3	mouse rat	no fs	
3q26.2	169,301,000	172,799,001	6	mouse rat	no fs	
3q26.3	172,799,001	184,201,000	10	mouse rat dog	no fs	
3q27	184,201,000	189,501,000	4	mouse rat + chicken	fs	common
3q29	193,999,001	199,505,740	10	mouse rat dog + chicken	no fs	
4p12	45,799,001	48,901,000	1	dog	no fs	
4p13	41,099,001	45,801,000	2	dog	no fs	
4p15.3	10,999,001	22,701,000	4	cattle dog	fs	common
4p16	1	10,999,001	30	cattle mouse pig rat dog + chicken	fs (4p16.1)	common
4q12	53,199,001	59,701,000	5	pig dog + chicken	fs	common
4q13.3	70,599,001	76,601,000	2	dog	no fs	
4q21.1	76,601,000	79,201,000	4	dog pig + chicken	no fs	
4q21.2	79,201,000	87,301,000	5	pig + chicken	no fs	
4q22	88,399,001	99,201,000	13	cattle mouse rat + chicken	fs	common
4q26	114,399,001	121,101,000	3	cattle mouse rat	no fs	
4q27	121,101,000	124,299,001	9	dog mouse rat cattle	no fs	
4q28	124,299,001	139,801,000	4	chicken	no fs	
4q31.1	139,801,000	141,801,000	4	mouse rat	fs	common
4q31.2	141,801,000	151,999,001	6	mouse rat dog	no fs	
4q31.3	151,999,001	155,901,000	2	chicken	no fs	

4q32	155,901,000	170,499,001	16	cattle mouse pig rat dog	no fs	
4q33	170,499,001	172,301,000	1	chicken	no fs	
4q34	172,301,000	183,599,001	5	pig dog + chicken	no fs	
4q35	183,599,001	191,411,218	4	pig + chicken	no fs	
5p12	42,399,001	45,801,000	3	dog mouse	no fs	
5p15.1	15,099,001	18,501,000	2	pig	no fs	
5p15.2	9,899,001	15,099,001	3	dog pig	no fs	
5p15.3	1	9,899,001	9	mouse pig rat + chicken	no fs	
5q11.2	50,499,001	58,901,000	5	cat dog + chicken	no fs	
5q12	58,901,000	66,701,000	1	pig	no fs	
5q13.1	66,701,000	68,401,000	1	pig	no fs	
5q13.2	68,401,000	73,299,001	4	cat pig + chicken	no fs	
5q13.3	73,299,001	76,901,000	3	cat pig + chicken	no fs	
5q14	76,901,000	91,901,000	7	cattle + chicken	no fs	
5q15	91,901,000	97,301,000	6	mouse rat + chicken	fs	common
5q21	97,301,000	109,599,001	6	mouse rat	fs	common
5q22	109,599,001	115,201,000	8	mouse rat	no fs	
5q23.3	127,899,001	132,201,000	8	cattle mouse rat + chicken	no fs	
5q31.1	132,201,000	137,201,000	8	mouse rat	fs	common
5q32	144,499,001	149,801,000	8	cat cattle mouse rat	no fs	
5q33.1	149,801,000	152,701,000	6	mouse pig rat	no fs	
5q33.2	152,701,000	155,599,001	4	mouse rat	no fs	
5q33.3	155,599,001	159,901,000	2	chicken	no fs	
5q34	159,901,000	168,399,001	2	cattle	no fs	
5q35.1	168,399,001	172,701,000	1	mouse	fs	rare
5q35.2	172,701,000	176,499,001	3	rat mouse	fs	rare
5q35.3	176,499,001	180,857,866	7	mouse rat cattle dog	fs	rare
6p11.2	57,499,001	58,801,000	2	dog + chicken	no fs	
6p12	46,299,001	57,499,001	15	mouse pig rat + chicken	no fs	
6p21.1	40,601,000	46,299,001	10	chicken	no fs	
6p21.2	36,799,001	40,601,000	6	mouse pig rat + chicken	no fs	
6p21.3	29,999,001	36,799,001	11	mouse pig cattle dog + chicken	no fs	
6p22.1	26,401,000	29,999,001	7	cat dog mouse rat	no fs	
6p22.2	24,099,001	26,401,000	3	chicken	fs	common

6p22.3	15,299,001	24,099,001	5	mouse rat + chicken	no fs	
6q12	63,499,001	70,001,000	2	dog pig	no fs	
6q13	70,001,000	75,899,001	6	mouse pig rat	fs	common
6q14	75,899,001	87,501,000	6	mouse pig rat	no fs	
6q15	87,501,000	92,099,001	1	cat	fs	common
6q16.1	92,099,001	98,701,000	1	cat	no fs	
6q16.3	99,999,001	105,101,000	4	mouse rat	no fs	
6q22.1	114,499,001	118,401,000	8	cattle mouse pig rat	no fs	
6q22.3	118,599,001	130,401,000	10	cattle dog mouse rat	no fs	
6q23.2	131,399,001	135,201,000	8	dog rat	no fs	
6q24	139,099,001	149,101,000	2	chicken	no fs	
6q25.1	149,101,000	152,601,000	4	mouse rat	no fs	
6q25.2	152,601,000	155,599,001	2	mouse	no fs	
6q25.3	155,599,001	161,001,000	4	mouse + chicken	no fs	
6q27	164,499,001	170,975,699	4	dog mouse	no fs	
7p11.2	53,599,001	56,901,000	8	cattle pig + chicken	fs	rare
7p12	47,099,001	53,599,001	4	pig + chicken	no fs	
7p13	43,101,000	47,099,001	6	mouse rat + chicken	fs	common
7p14	30,299,001	43,101,000	12	mouse pig rat	fs (7p14.2)	common
7p15.2	25,299,001	27,701,000	1	cattle	no fs	
7p15.3	20,699,001	25,299,001	7	cattle mouse pig rat	no fs	
7p21	6,999,001	20,701,000	10	mouse pig rat + chicken	no fs	
7p22	1	6,999,001	8	dog pig rat + chicken	fs	common
7q11.22	66,499,001	71,601,000	2	pig	fs	common
7q11.23	71,601,000	77,201,000	17	cattle horse mouse pig rat + chicken	fs	common
7q21.1	77,201,000	90,701,000	9	mouse pig cattle + chicken	no fs	
7q21.2	90,701,000	92,399,001	1	mouse	fs	common
7q21.3	92,399,001	97,601,000	11	cattle mouse pig rat + chicken	no fs	
7q22	97,601,000	106,901,000	22	cat cattle mouse pig rat + chicken	fs	common
7q31.1	106,901,000	114,201,000	12	mouse pig rat + chicken	no fs	
7q31.3	116,999,001	125,901,000	2	dog	no fs	
7q32	125,901,000	132,001,000	4	chicken	fs (7q32.3)	common
7q33	132,001,000	137,099,001	10	dog + chicken	no fs	
7q34	137,099,001	143,001,000	6	dog pig + chicken	no fs	

7q35	143,001,000	147,299,001	2	pig + chicken	no fs	
7q36	147,299,001	158,628,139	14	mouse pig rat dog + chicken	fs	common
8p11.2	38,499,001	43,201,000	1	pig	no fs	
8p12	28,899,001	38,499,001	17	mouse pig rat cattle + chicken	no fs	
8p21.1	27,399,001	28,899,001	3	cattle pig + chicken	no fs	
8p21.2	23,401,000	27,399,001	2	horse + chicken	no fs	
8p21.3	19,099,001	23,401,000	8	cattle mouse rat horse pig	no fs	
8p22	12,699,001	19,099,001	16	cattle mouse pig rat + chicken	no fs	
8p23.1	6,199,001	12,699,001	25	dog mouse rat pig + chicken	no fs	
8p23.3	1	2,201,000	3	dog	no fs	
8q11.21	48,099,001	52,401,000	5	cattle dog mouse	no fs	
8q11.23	52,799,001	55,701,000	1	cattle	no fs	
8q12	55,701,000	66,101,000	12	cattle mouse rat	no fs	
8q13	66,101,000	74,201,000	4	mouse rat	no fs	
8q21.1	74,201,000	84,801,000	6	cattle mouse rat	no fs	
8q21.2	84,801,000	87,001,000	4	mouse rat	no fs	
8q22.1	93,399,001	99,101,000	6	mouse rat dog	fs	common
8q22.3	101,599,001	106,201,000	2	cattle	fs	rare
8q23	106,201,000	117,701,000	1	cattle	no fs	
8q24.1	117,701,000	127,301,000	7	cattle + chicken	fs	common/rare
9p13	33,199,001	41,401,000	3	pig + chicken	no fs	
9p21	19,899,001	33,201,000	10	cattle mouse pig rat + chicken	fs	common/rare
9p23	8,999,001	14,101,000	2	chicken	no fs	
9p24	1	9,001,000	7	mouse rat dog + chicken	no fs	
9q21.2	76,399,001	78,301,000	4	mouse rat	no fs	
9q21.3	78,301,000	87,601,000	16	cattle mouse pig rat	no fs	
9q22.1	87,601,000	89,001,000	5	mouse pig rat	fs	common
9q22.2	89,001,000	90,999,001	5	mouse rat	no fs	
9q22.3	90,999,001	99,601,000	14	mouse pig rat + chicken	no fs	
9q31	99,601,000	112,001,000	10	chicken	no fs	
9q32	112,001,000	114,801,000	4	chicken	fs	common/rare
9q33	114,801,000	127,301,000	17	mouse rat dog + chicken	no fs	
9q34.1	127,301,000	133,001,000	13	mouse rat + chicken	no fs	
9q34.3	134,699,001	138,429,268	2	chicken	no fs	

Xp11.22	49,499,001	54,501,000	3	mouse rat	no fs	
Xp11.23	47,199,001	49,499,001	3	mouse rat	no fs	
Xp11.3	42,099,001	47,199,001	6	mouse rat + chicken	no fs	
Xp21.1	31,299,001	37,401,000	4	mouse rat	no fs	
Xp22.1	17,199,001	24,701,000	4	mouse rat	no fs	
Xq11	59,399,001	64,001,000	2	dog	no fs	
Xq13	67,499,001	75,801,000	2	chicken	no fs	
Xq21.1	75,801,000	84,401,000	7	mouse + chicken	no fs	
Xq21.3	86,099,001	98,201,000	4	dog mouse	no fs	
Xq22.1	98,201,000	102,401,000	1	chicken	fs	common
Xq22.2	102,401,000	103,499,001	1	chicken	no fs	
Xq22.3	103,499,001	108,501,000	3	rat + chicken	no fs	
Xq23	108,501,000	116,301,000	9	cattle mouse horse rat + chicken	no fs	
Xq24	116,301,000	120,599,001	2	horse	no fs	
Xq25	120,599,001	129,701,000	3	rat + chicken	no fs	
Xq26	129,701,000	137,699,001	2	rat + chicken	no fs	
Xq27	137,699,001	146,801,000	1	chicken	fs	common/rare
