# Supplementary material for: Is mammalian chromosomal evolution driven by regions of genome fragility?
Source: Genome Biol. 2006 Dec 8;7(12):R115. doi: 10.1186/gb-2006-7-12-r115 (PMC1794428; doi:10.1186/gb-2006-7-12-r115)
Supplement: Additional data file 7 — List of all human fragile sites described in the literature. [file gb-2006-7-12-r115-S7.pdf]

Table S4. **Distribution of human fragile sites (classification based on [39]).**
The position of each chromosomal band in Mb based on the Ensemble data base [74] has been plotted to the human ideogram. The species in which the evolutionary breakpoints were identified are presented. An asterisk indicates fragile sites situated in bands comprising ≥6000bp of tandem repeats in windows of 0.250 Mb. Two asterisks denote fragile sites in bands with ≥10000bp of tandem repeats in windows of 0.250 Mb.

Chromosomal band	Position (from)	Position (to)	Fragile site	Type	Species
10q11.2*	41.9Mb	52.6Mb	FRA10G	common	cattle mouse pig rat dog chicken
10q21*	52.6Mb	70.3Mb	FRA10C	common	cattle mouse rat chicken
10q22.1*	70.3Mb	74.6Mb	FRA10D	common	rat chicken
10q23.3*	89.1Mb	97Mb	FRA10A	rare	mouse pig rat chicken
10q25.2	111.8Mb	114.9Mb	FRA10E	common	
10q25.2	111.8Mb	114.9Mb	FRA10B	rare	
10q26.1*	118.7Mb	127.4Mb	FRA10F	common	mouse rat chicken
11p13	31Mb	36.4Mb	FRA11E	common	cat chicken
11p14.2	26Mb	27.2Mb	FRA11D	common	cattle mouse rat
11p15.1**	16.1Mb	21.6Mb	FRA11C	common	cattle mouse rat chicken
11p15.1**	16.1Mb	21.6Mb	FRA11I	rare	cattle mouse rat chicken
11q13**	63.1Mb	77.4Mb	FRA11H	common	mouse cat cattle pig rat dog chicken
11q13.3**	68.3Mb	70Mb	FRA11A	rare	mouse cat cattle pig rat dog chicken
11q14.2*	85.3Mb	87.8Mb	FRA11F	common	
11q23.3*	114.4Mb	121.2Mb	FRA11G	common	
11q23.3*	114.4Mb	121.2Mb	FRA11B	rare	
12q13.1*	44.6Mb	53.1Mb	FRA12A	rare	pig
12q21.3*	78.9Mb	91.2Mb	FRA12B	common	
12q24**	107.5Mb	132.5Mb	FRA12E	common	mouse rat pig dog chicken
12q24.13*	110.8Mb	112.8Mb	FRA12D	rare	chicken
12q24.2*	112.8Mb	119.3Mb	FRA12C	rare	
13q13.2	32.9Mb	34.5Mb	FRA13A	common	mouse rat
13q21**	54.2Mb	72.1Mb	FRA13B	common	
13q21.2*	58.5Mb	61.2Mb	FRA13C	common	
13q32	93.8Mb	100.5Mb	FRA13D	common	
14q23*	57.2Mb	67Mb	FRA14B	common	pig
14q24.1	67Mb	69.3Mb	FRA14C	common	
15q22*	57Mb	65.3Mb	FRA15A	common	cattle chicken
16p12.1*	21.7Mb	27.6Mb	FRA16E	rare	chicken
16p13.11**	14.7Mb	16.7Mb	FRA16A	rare	mouse rat chicken
16q22.1*	63.9Mb	69.4Mb	FRA16C	common	cattle mouse pig rat chicken
16q22.1*	63.9Mb	69.4Mb	FRA16B	rare	cattle mouse pig rat chicken
16q23.2*	78.5Mb	80.6Mb	FRA16D	common	
17p12	10.7Mb	15.9Mb	FRA17A	rare	chicken
17q23.1	50.9Mb	51.2Mb	FRA17B	common	
18q12.2	31Mb	35.5Mb	FRA18A	common	cattle chicken
18q21.3*	52.5Mb	59.8Mb	FRA18B	common	mouse rat pig chicken
18q22.1	59.8Mb	64.9Mb	FRA18C	rare	cattle chicken
19p13**	1Mb	19.8Mb	FRA19B	rare	cat cattle mouse rat chicken
19q13	37.1Mb	63.8Mb	FRA19A	common	mouse rat cat chicken
1p21.2	99.5Mb	102Mb	FRA1E	common	
1p21.3	94.49Mb	99.5Mb	FRA1M	rare	
1p22	84.6Mb	94.5Mb	FRA1D	common	mouse rat pig
1p31	60.1Mb	84.6Mb	FRA1L	common	pig dog mouse rat chicken

1p31.2	65.5Mb	69.5Mb	FRA1C	common	dog mouse rat chicken
1p32**	50.4Mb	60.1Mb	FRA1B	common	mouse rat dog
1p36**	7.1Mb	27.7Mb	FRA1A	common	cattle rat dog chicken
1q12	heterochr	heterochr	FRA1J	common	
1q21**	141.6Mb	151.4Mb	FRA1F	common	dog
1q25.1	169.7Mb	172.8Mb	FRA1G	common	cattle
1q31	182.5Mb	196Mb	FRA1K	common	cattle pig cat
1q42**	219.8Mb	233Mb	FRA1H	common	cat cattle mouse pig rat chicken
1q44**	239.5Mb	245.5Mb	FRA1I	common	cattle dog
20p11.23	17.8Mb	21.2Mb	FRA20A	rare	chicken
20p12.2	9Mb	11.9Mb	FRA20B	common	
22q12.2*	27.9Mb	30.5Mb	FRA22B	common	cattle horse mouse pig chicken
22q13**	35.8Mb	49.5Mb	FRA22A	rare	cat cattle chicken
2p11.2**	83.3Mb	91Mb	FRA2L	rare	cattle
2p13**	68.5Mb	75Mb	FRA2E	common	cattle mouse rat dog chicken
2p16.2	52.8Mb	54.9Mb	FRA2D	common	cattle mouse chicken
2p24.2	16.7Mb	19.2Mb	FRA2C	common	
2q11.2*	95.8Mb	102.6Mb	FRA2A	rare	dog
2q13*	108.7Mb	113.9Mb	FRA2B	rare	cattle mouse pig rat dog chicken
2q21.3	134.9Mb	137.4Mb	FRA2F	common	cattle
2q22.3	144Mb	148.5Mb	FRA2K	rare	
2q31*	169.6Mb	182.8Mb	FRA2G	common	
2q32.1*	182.8Mb	189.2Mb	FRA2H	common	mouse rat
2q33*	197.3Mb	208.9Mb	FRA2I	common	chicken
2q37.3**	237.1Mb	243Mb	FRA2J	common	cattle chicken
3p14.2	58.5Mb	63.7Mb	FRA3B	common	
3p24.2	23.8Mb	27.1Mb	FRA3A	common	mouse rat
3q25	150.4Mb	162.2Mb	FRA3D	common	dog
3q27*	184.2Mb	189.5Mb	FRA3C	common	mouse rat chicken
4p15**	11Mb	35.6Mb	FRA4D	common	cattle dog
4p16.1**	5.9Mb	11Mb	FRA4A	common	cattle mouse pig rat dog
4q12	53.2Mb	59.7Mb	FRA4B	common	pig dog chicken
4q22	88.4Mb	99.2Mb	FRA4F	common	cattle mouse rat chicken
4q31.1	139.8Mb	141.8Mb	FRA4C	common	mouse rat
5p13*	29.3Mb	42.4Mb	FRA5A	common	
5p14**	18.5Mb	29.3Mb	FRA5E	common	
5q15	91.9Mb	97.3Mb	FRA5D	common	mouse rat chicken
5q15	91.9Mb	97.3Mb	FRA5B	common	mouse rat chicken
5q21**	97.3Mb	109.6Mb	FRA5F	common	mouse rat
5q31.1**	132.2Mb	137.2Mb	FRA5C	common	mouse rat
5q35**	169.4Mb	180.9Mb	FRA5G	rare	mouse rat cattle dog
6p22.2	24.1Mb	26.4Mb	FRA6C	common	chicken
6p23*	13.5Mb	15.3Mb	FRA6A	rare	
6p25.1*	4.1Mb	7Mb	FRA6B	common	
6q13*	70Mb	75.9Mb	FRA6D	common	mouse pig rat
6q15	87.5Mb	92.1Mb	FRA6G	common	cat
6q21*	105.1Mb	114.5Mb	FRA6F	common	
6q26**	161Mb	164.5Mb	FRA6E	common	
7p11.2*	53.6Mb	56.9Mb	FRA7A	rare	cattle pig chicken
7p13*	43.1Mb	47.1Mb	FRA7D	common	mouse rat chicken
7p14.2	34.7Mb	37Mb	FRA7C	common	mouse pig rat
7p22**	1Mb	7Mb	FRA7B	common	dog pig rat chicken
7q11**	58.9Mb	77.2Mb	FRA7J	common	cattle horse mouse pig rat chicken
7q21.2	90.7Mb	92.4Mb	FRA7E	common	mouse

7q22**	97.6Mb	106.9Mb	FRA7F	common	cattle cat mouse pig rat chicken
7q31.2	114.2Mb	117Mb	FRA7G	common	
7q32.3	129.9Mb	132Mb	FRA7H	common	chicken
7q36**	147.3Mb	158.6Mb	FRA7I	common	mouse rat pig dog chicken
8q22.1*	93.4Mb	99.1Mb	FRA8B	common	mouse rat dog
8q22.3	101.6Mb	106.2Mb	FRA8A	rare	cattle
8q24.1	117.7Mb	127.3Mb	FRA8C	common	cattle chicken
8q24.1	117.7Mb	127.3Mb	FRA8E	rare	cattle chicken
8q24.3**	140Mb	146.3Mb	FRA8D	common	
9p21	19.9Mb	33.2Mb	FRA9C	common	cattle mouse pig rat chicken
9p21	19.9Mb	33.2Mb	FRA9A	rare	cattle mouse pig rat chicken
9q12**	heterochr	heterochr	FRA9F	common	
9q22.1	87.6Mb	89Mb	FRA9D	common	mouse pig rat
9q32*	112Mb	114.8Mb	FRA9E	common	chicken
9q32*	112Mb	114.8Mb	FRA9B	rare	chicken
Xp22.31	5.8Mb	9.3Mb	FRAXB	common	
Xq22.1	98.2Mb	102.4Mb	FRAXC	common	chicken
Xq27.2	140Mb	141.8Mb	FRAXD	common	
Xq27.3	141.8Mb	146.8Mb	FRAXA	rare	
Xq28	146.8Mb	154.8Mb	FRAXE	rare	
Xq28	146.8Mb	154.8Mb	FRAXF	rare	
