# Supplementary material for: Is mammalian chromosomal evolution driven by regions of genome fragility?
Source: Genome Biol. 2006 Dec 8;7(12):R115. doi: 10.1186/gb-2006-7-12-r115 (PMC1794428; doi:10.1186/gb-2006-7-12-r115)
Supplement: Additional data file 8 — List of common human fragile sites that have been cloned and analyzed at the molecular level. [file gb-2006-7-12-r115-S8.pdf]

Table S5: List of common human fragile sites analysed at the molecular level, indicating their position defined by markers or BAC clones and their content of evolutionary breakpoints (EB).

human cFS	clones or markers	reference	spanning region (Mb)	spanning EB	surronding EB (Mb)	species
FRA 1E	526F14-145D11	Hormozian et al. 2006	97,6-98,1	no		
FRA 2G	285F23-724O16	Limongi et al. 2003	169,3-170,3	no		
FRA 3B	SHGC84349-RH16786	Wilke et al. 1996	59,6-63,8	no	yes (1Mb)	rat, mouse
FRA 4F	549C16-145G20	Rozier et al. 2004	90,56-97,6	yes		cattle, mouse, rat, chicken
FRA 6E	D6S1581-D6S1719	Denison et al. 2003	160-165,9	yes		mouse, chicken
FRA 6F	RH124087-SHGC82095	Morelli et al. 2002	111,5-112,6	no		
FRA 9E	D9S1832-D9S177	Callahan et al. 2003	106-115,5	yes		chicken
FRA 7E	D7S2443-D7S524	Zlotorynski et al. 2003	79-84,5	yes		chicken
FRA 7H	D7S788-D7S649	Mishmar et al. 1998	129,86-130,3	yes		chicken
FRA 7G	AC003080-AC002465	Hellman et al. 2002	110,2-116,7	yes		rat, mouse, dog
FRA 13A	RP1166B8-RP1198D3	Savelyeva et al. 2006	34,4-35,2	no		
FRA 16D	D16S518-D16S3029	Krummel et al. 2000	76,7-77,5	no	yes (1Mb)	chicken
FRA XB	n.a.	Arlt et al. 2002	n.a.	n.a.	n.a.	n.a.
