# Supplementary material for: Is mammalian chromosomal evolution driven by regions of genome fragility?
Source: Genome Biol. 2006 Dec 8;7(12):R115. doi: 10.1186/gb-2006-7-12-r115 (PMC1794428; doi:10.1186/gb-2006-7-12-r115)
Supplement: Additional data file 9 — Human genome divided into windows of 0.250 Mb. [file gb-2006-7-12-r115-S9.pdf]

Table S6: The human genome divided into windows of 0.250Mb.

chr	band type	chromosomal band	nº species with EB	position (nt)	nº bases repeated	fs	type of fs
chr01	T	1p36.3	1	125000	6204	fs	fs common
chr01	T	1p36.3	1	375000	8902	fs	fs common
chr01	T	1p36.3	1	625000	8829	fs	fs common
chr01	T	1p36.3	1	875000	22007	fs	fs common
chr01	T	1p36.3	1	1125000	27027	fs	fs common
chr01	T	1p36.3	1	1375000	13386	fs	fs common
chr01	T	1p36.3	1	1625000	7267	fs	fs common
chr01	T	1p36.3	1	1875000	12515	fs	fs common
chr01	T	1p36.3	1	2125000	17014	fs	fs common
chr01	T	1p36.3	1	2375000	9450	fs	fs common
chr01	T	1p36.3	1	2625000	59087	fs	fs common
chr01	T	1p36.3	1	2875000	18004	fs	fs common
chr01	T	1p36.3	1	3125000	15241	fs	fs common
chr01	E	1p36.3	1	3375000	12208	fs	fs common
chr01	E	1p36.3	1	3625000	17202	fs	fs common
chr01	E	1p36.3	1	3875000	9935	fs	fs common
chr01	E	1p36.3	1	4125000	10145	fs	fs common
chr01	E	1p36.3	1	4375000	9834	fs	fs common
chr01	E	1p36.3	1	4625000	8246	fs	fs common
chr01	E	1p36.3	1	4875000	3199	fs	fs common
chr01	E	1p36.3	1	5125000	5209	fs	fs common
chr01	E	1p36.3	1	5375000	7123	fs	fs common
chr01	E	1p36.3	1	5625000	9219	fs	fs common
chr01	E	1p36.3	1	5875000	11121	fs	fs common
chr01	E	1p36.3	1	6125000	12661	fs	fs common
chr01	E	1p36.3	1	6375000	8817	fs	fs common
chr01	E	1p36.3	1	6625000	6396	fs	fs common
chr01	E	1p36.3	1	6875000	4412	fs	fs common
chr01	E	1p36.3	1	7125000	5791	fs	fs common
chr01	E	1p36.2	3	7375000	8160	fs	fs common
chr01	E	1p36.2	3	7625000	9930	fs	fs common

chr01	E	1p36.2	3	7875000	5794	fs	fs common
chr01	E	1p36.2	3	8125000	4496	fs	fs common
chr01	E	1p36.2	3	8375000	6843	fs	fs common
chr01	E	1p36.2	3	8625000	3666	fs	fs common
chr01	E	1p36.2	3	8875000	4215	fs	fs common
chr01	E	1p36.2	3	9125000	7485	fs	fs common
chr01	E	1p36.2	3	9375000	6006	fs	fs common
chr01	E	1p36.2	3	9625000	4530	fs	fs common
chr01	E	1p36.2	3	9875000	6711	fs	fs common
chr01	E	1p36.2	3	10125000	8368	fs	fs common
chr01	E	1p36.2	3	10375000	3266	fs	fs common
chr01	E	1p36.2	3	10625000	5174	fs	fs common
chr01	E	1p36.2	3	10875000	4759	fs	fs common
chr01	E	1p36.2	3	11125000	5921	fs	fs common
chr01	E	1p36.2	3	11375000	6994	fs	fs common
chr01	E	1p36.2	3	11625000	4419	fs	fs common
chr01	E	1p36.2	3	11875000	4724	fs	fs common
chr01	E	1p36.2	3	12125000	7581	fs	fs common
chr01	E	1p36.2	3	12375000	3245	fs	fs common
chr01	E	1p36.2	3	12625000	4805	fs	fs common
chr01	E	1p36.2	3	12875000	3887	fs	fs common
chr01	E	1p36.2	3	13125000	5139	fs	fs common
chr01	E	1p36.2	3	13375000	5607	fs	fs common
chr01	E	1p36.2	3	13625000	5822	fs	fs common
chr01	E	1p36.2	3	13875000	957	fs	fs common
chr01	E	1p36.2	3	14125000	2817	fs	fs common
chr01	E	1p36.2	3	14375000	2196	fs	fs common
chr01	E	1p36.2	3	14625000	3281	fs	fs common
chr01	E	1p36.2	3	14875000	3988	fs	fs common
chr01	E	1p36.2	3	15125000	4815	fs	fs common
chr01	E	1p36.2	3	15375000	3289	fs	fs common
chr01	E	1p36.2	3	15625000	4928	fs	fs common
chr01	E	1p36.1	3	15875000	4948	fs	fs common
chr01	E	1p36.1	3	16125000	4264	fs	fs common
chr01	E	1p36.1	3	16375000	4675	fs	fs common
chr01	E	1p36.1	3	16625000	2918	fs	fs common

chr01	E	1p36.1	3	16875000	2245	fs	fs common
chr01	E	1p36.1	3	17125000	2858	fs	fs common
chr01	E	1p36.1	3	17375000	4708	fs	fs common
chr01	E	1p36.1	3	17625000	5944	fs	fs common
chr01	E	1p36.1	3	17875000	4264	fs	fs common
chr01	E	1p36.1	3	18125000	5601	fs	fs common
chr01	E	1p36.1	3	18375000	4924	fs	fs common
chr01	E	1p36.1	3	18625000	4427	fs	fs common
chr01	E	1p36.1	3	18875000	5177	fs	fs common
chr01	E	1p36.1	3	19125000	3904	fs	fs common
chr01	E	1p36.1	3	19375000	1938	fs	fs common
chr01	E	1p36.1	3	19625000	2861	fs	fs common
chr01	E	1p36.1	3	19875000	3575	fs	fs common
chr01	E	1p36.1	3	20125000	2431	fs	fs common
chr01	E	1p36.1	3	20375000	3619	fs	fs common
chr01	E	1p36.1	3	20625000	3953	fs	fs common
chr01	E	1p36.1	3	20875000	5773	fs	fs common
chr01	E	1p36.1	3	21125000	4104	fs	fs common
chr01	E	1p36.1	3	21375000	6183	fs	fs common
chr01	E	1p36.1	3	21625000	4339	fs	fs common
chr01	E	1p36.1	3	21875000	3341	fs	fs common
chr01	E	1p36.1	3	22125000	2797	fs	fs common
chr01	E	1p36.1	3	22375000	7077	fs	fs common
chr01	E	1p36.1	3	22625000	6944	fs	fs common
chr01	E	1p36.1	3	22875000	4289	fs	fs common
chr01	E	1p36.1	3	23125000	2641	fs	fs common
chr01	E	1p36.1	3	23375000	5069	fs	fs common
chr01	E	1p36.1	3	23625000	5171	fs	fs common
chr01	E	1p36.1	3	23875000	8283	fs	fs common
chr01	E	1p36.1	3	24125000	6605	fs	fs common
chr01	E	1p36.1	3	24375000	4117	fs	fs common
chr01	E	1p36.1	3	24625000	4432	fs	fs common
chr01	E	1p36.1	3	24875000	7963	fs	fs common
chr01	E	1p36.1	3	25125000	5305	fs	fs common
chr01	E	1p36.1	3	25375000	3045	fs	fs common
chr01	E	1p36.1	3	25625000	3190	fs	fs common

chr01	E	1p36.1	3	25875000	4911	fs	fs common
chr01	E	1p36.1	3	26125000	2983	fs	fs common
chr01	E	1p36.1	3	26375000	4873	fs	fs common
chr01	E	1p36.1	3	26625000	6812	fs	fs common
chr01	E	1p36.1	3	26875000	6189	fs	fs common
chr01	E	1p36.1	3	27125000	6924	fs	fs common
chr01	E	1p36.1	3	27375000	6175	fs	fs common
chr01	E	1p36.1	3	27625000	5512	fs	fs common
chr01	E	1p35	4	27875000	6345	nofs	nofs
chr01	E	1p35	4	28125000	3150	nofs	nofs
chr01	E	1p35	4	28375000	3994	nofs	nofs
chr01	E	1p35	4	28625000	3642	nofs	nofs
chr01	E	1p35	4	28875000	5686	nofs	nofs
chr01	E	1p35	4	29125000	3485	nofs	nofs
chr01	E	1p35	4	29375000	4903	nofs	nofs
chr01	E	1p35	4	29625000	2379	nofs	nofs
chr01	E	1p35	4	29875000	6708	nofs	nofs
chr01	E	1p35	4	30125000	4414	nofs	nofs
chr01	E	1p35	4	30375000	4132	nofs	nofs
chr01	E	1p35	4	30625000	7122	nofs	nofs
chr01	E	1p35	4	30875000	6415	nofs	nofs
chr01	E	1p35	4	31125000	4374	nofs	nofs
chr01	E	1p35	4	31375000	6225	nofs	nofs
chr01	E	1p35	4	31625000	6720	nofs	nofs
chr01	E	1p35	4	31875000	1847	nofs	nofs
chr01	E	1p35	4	32125000	4067	nofs	nofs
chr01	E	1p35	4	32375000	6031	nofs	nofs
chr01	E	1p35	4	32625000	3724	nofs	nofs
chr01	E	1p35	4	32875000	4978	nofs	nofs
chr01	E	1p35	4	33125000	6884	nofs	nofs
chr01	E	1p35	4	33375000	3830	nofs	nofs
chr01	E	1p35	4	33625000	2673	nofs	nofs
chr01	E	1p35	4	33875000	2142	nofs	nofs
chr01	E	1p35	4	34125000	3811	nofs	nofs
chr01	E	1p35	4	34375000	3376	nofs	nofs
chr01	E	1p34.3	2	34625000	2021	nofs	nofs

chr01	E	1p34.3	2	34875000	2613	nofs	nofs
chr01	E	1p34.3	2	35125000	4513	nofs	nofs
chr01	E	1p34.3	2	35375000	5718	nofs	nofs
chr01	E	1p34.3	2	35625000	2675	nofs	nofs
chr01	E	1p34.3	2	35875000	4857	nofs	nofs
chr01	E	1p34.3	2	36125000	2995	nofs	nofs
chr01	E	1p34.3	2	36375000	5573	nofs	nofs
chr01	E	1p34.3	2	36625000	6770	nofs	nofs
chr01	E	1p34.3	2	36875000	4143	nofs	nofs
chr01	E	1p34.3	2	37125000	6751	nofs	nofs
chr01	E	1p34.3	2	37375000	2981	nofs	nofs
chr01	E	1p34.3	2	37625000	4905	nofs	nofs
chr01	E	1p34.3	2	37875000	2108	nofs	nofs
chr01	E	1p34.3	2	38125000	6061	nofs	nofs
chr01	E	1p34.3	2	38375000	2566	nofs	nofs
chr01	E	1p34.3	2	38625000	2729	nofs	nofs
chr01	E	1p34.3	2	38875000	2623	nofs	nofs
chr01	E	1p34.3	2	39125000	5945	nofs	nofs
chr01	E	1p34.3	2	39375000	5183	nofs	nofs
chr01	E	1p34.3	2	39625000	4249	nofs	nofs
chr01	E	1p34.3	2	39875000	2704	nofs	nofs
chr01	E	1p34.2	3	40125000	4683	nofs	nofs
chr01	E	1p34.2	3	40375000	3133	nofs	nofs
chr01	E	1p34.2	3	40625000	4500	nofs	nofs
chr01	E	1p34.2	3	40875000	1668	nofs	nofs
chr01	E	1p34.2	3	41125000	2117	nofs	nofs
chr01	E	1p34.2	3	41375000	2364	nofs	nofs
chr01	E	1p34.2	3	41625000	6952	nofs	nofs
chr01	E	1p34.2	3	41875000	2425	nofs	nofs
chr01	E	1p34.2	3	42125000	2204	nofs	nofs
chr01	E	1p34.2	3	42375000	3900	nofs	nofs
chr01	E	1p34.2	3	42625000	3226	nofs	nofs
chr01	E	1p34.2	3	42875000	3046	nofs	nofs
chr01	E	1p34.2	3	43125000	3392	nofs	nofs
chr01	E	1p34.2	3	43375000	2523	nofs	nofs
chr01	E	1p34.2	3	43625000	2770	nofs	nofs

chr01	E	1p34.2	3	43875000	3681	nofs	nofs
chr01	B	1p34.1	.	44125000	5433	nofs	nofs
chr01	B	1p34.1	.	44375000	4576	nofs	nofs
chr01	B	1p34.1	.	44625000	4213	nofs	nofs
chr01	B	1p34.1	.	44875000	7630	nofs	nofs
chr01	B	1p34.1	.	45125000	6542	nofs	nofs
chr01	B	1p34.1	.	45375000	5308	nofs	nofs
chr01	B	1p34.1	.	45625000	6289	nofs	nofs
chr01	B	1p34.1	.	45875000	5454	nofs	nofs
chr01	B	1p34.1	.	46125000	2500	nofs	nofs
chr01	B	1p34.1	.	46375000	3726	nofs	nofs
chr01	B	1p34.1	.	46625000	3556	nofs	nofs
chr01	B	1p33	.	46875000	2567	nofs	nofs
chr01	B	1p33	.	47125000	1374	nofs	nofs
chr01	B	1p33	.	47375000	3279	nofs	nofs
chr01	B	1p33	.	47625000	3009	nofs	nofs
chr01	B	1p33	.	47875000	2845	nofs	nofs
chr01	B	1p33	.	48125000	2945	nofs	nofs
chr01	B	1p33	.	48375000	3517	nofs	nofs
chr01	B	1p33	.	48625000	5547	nofs	nofs
chr01	B	1p33	.	48875000	2103	nofs	nofs
chr01	B	1p33	.	49125000	1687	nofs	nofs
chr01	B	1p33	.	49375000	2127	nofs	nofs
chr01	B	1p33	.	49625000	3041	nofs	nofs
chr01	B	1p33	.	49875000	1476	nofs	nofs
chr01	B	1p33	.	50125000	2878	nofs	nofs
chr01	E	1p32.3	1	50375000	2597	fs	fs common
chr01	E	1p32.3	1	50625000	3776	fs	fs common
chr01	E	1p32.3	1	50875000	2868	fs	fs common
chr01	E	1p32.3	1	51125000	4276	fs	fs common
chr01	E	1p32.3	1	51375000	2248	fs	fs common
chr01	E	1p32.3	1	51625000	2774	fs	fs common
chr01	E	1p32.3	1	51875000	3944	fs	fs common
chr01	E	1p32.3	1	52125000	2661	fs	fs common
chr01	E	1p32.3	1	52375000	3721	fs	fs common
chr01	E	1p32.3	1	52625000	3611	fs	fs common

chr01	E	1p32.3	1	52875000	6137	fs	fs common
chr01	E	1p32.3	1	53125000	3880	fs	fs common
chr01	E	1p32.3	1	53375000	10284	fs	fs common
chr01	E	1p32.3	1	53625000	5201	fs	fs common
chr01	E	1p32.3	1	53875000	3721	fs	fs common
chr01	E	1p32.3	1	54125000	2251	fs	fs common
chr01	E	1p32.3	1	54375000	3391	fs	fs common
chr01	E	1p32.3	1	54625000	6437	fs	fs common
chr01	E	1p32.3	1	54875000	3196	fs	fs common
chr01	E	1p32.3	1	55125000	2825	fs	fs common
chr01	E	1p32.3	1	55375000	1603	fs	fs common
chr01	E	1p32.3	1	55625000	1907	fs	fs common
chr01	E	1p32.3	1	55875000	6625	fs	fs common
chr01	E	1p32.2	2	56125000	1381	fs	fs common
chr01	E	1p32.2	2	56375000	1746	fs	fs common
chr01	E	1p32.2	2	56625000	2505	fs	fs common
chr01	E	1p32.2	2	56875000	2692	fs	fs common
chr01	E	1p32.2	2	57125000	1940	fs	fs common
chr01	E	1p32.2	2	57375000	2499	fs	fs common
chr01	E	1p32.2	2	57625000	2602	fs	fs common
chr01	E	1p32.2	2	57875000	3451	fs	fs common
chr01	E	1p32.2	2	58125000	1883	fs	fs common
chr01	E	1p32.2	2	58375000	2976	fs	fs common
chr01	E	1p32.2	2	58625000	1774	fs	fs common
chr01	B	1p32.1	.	58875000	3961	fs	fs common
chr01	B	1p32.1	.	59125000	2314	fs	fs common
chr01	B	1p32.1	.	59375000	2245	fs	fs common
chr01	B	1p32.1	.	59625000	2132	fs	fs common
chr01	B	1p32.1	.	59875000	1372	fs	fs common
chr01	B	1p32.1	.	60125000	3242	fs	fs common
chr01	B	1p31.3	.	60375000	3072	fs	fs common
chr01	B	1p31.3	.	60625000	1576	fs	fs common
chr01	B	1p31.3	.	60875000	2207	fs	fs common
chr01	B	1p31.3	.	61125000	2233	fs	fs common
chr01	B	1p31.3	.	61375000	1833	fs	fs common
chr01	B	1p31.3	.	61625000	3014	fs	fs common

chr01	B	1p31.3	.	61875000	3495	fs	fs common
chr01	B	1p31.3	.	62125000	1875	fs	fs common
chr01	B	1p31.3	.	62375000	4450	fs	fs common
chr01	B	1p31.3	.	62625000	4198	fs	fs common
chr01	B	1p31.3	.	62875000	3190	fs	fs common
chr01	B	1p31.3	.	63125000	2109	fs	fs common
chr01	B	1p31.3	.	63375000	1934	fs	fs common
chr01	B	1p31.3	.	63625000	5483	fs	fs common
chr01	B	1p31.3	.	63875000	2701	fs	fs common
chr01	B	1p31.3	.	64125000	1248	fs	fs common
chr01	B	1p31.3	.	64375000	2525	fs	fs common
chr01	B	1p31.3	.	64625000	5392	fs	fs common
chr01	B	1p31.3	.	64875000	2850	fs	fs common
chr01	B	1p31.3	.	65125000	4026	fs	fs common
chr01	B	1p31.3	.	65375000	2619	fs	fs common
chr01	E	1p31.2	4	65625000	2704	fs	fs common
chr01	E	1p31.2	4	65875000	2440	fs	fs common
chr01	E	1p31.2	4	66125000	1983	fs	fs common
chr01	E	1p31.2	4	66375000	2027	fs	fs common
chr01	E	1p31.2	4	66625000	2454	fs	fs common
chr01	E	1p31.2	4	66875000	3199	fs	fs common
chr01	E	1p31.2	4	67125000	3183	fs	fs common
chr01	E	1p31.2	4	67375000	1919	fs	fs common
chr01	E	1p31.2	4	67625000	3157	fs	fs common
chr01	E	1p31.2	4	67875000	2807	fs	fs common
chr01	E	1p31.2	4	68125000	2070	fs	fs common
chr01	E	1p31.2	4	68375000	2168	fs	fs common
chr01	E	1p31.2	4	68625000	2357	fs	fs common
chr01	E	1p31.2	4	68875000	2306	fs	fs common
chr01	E	1p31.2	4	69125000	1941	fs	fs common
chr01	E	1p31.2	4	69375000	2017	fs	fs common
chr01	E	1p31.2	4	69625000	1533	fs	fs common
chr01	E	1p31.1	2	69875000	2383	fs	fs common
chr01	E	1p31.1	2	70125000	1824	fs	fs common
chr01	E	1p31.1	2	70375000	2357	fs	fs common
chr01	E	1p31.1	2	70625000	2931	fs	fs common

chr01	E	1p31.1	2	70875000	4367	fs	fs common
chr01	E	1p31.1	2	71125000	2578	fs	fs common
chr01	E	1p31.1	2	71375000	1775	fs	fs common
chr01	E	1p31.1	2	71625000	2313	fs	fs common
chr01	E	1p31.1	2	71875000	2061	fs	fs common
chr01	E	1p31.1	2	72125000	3116	fs	fs common
chr01	E	1p31.1	2	72375000	2394	fs	fs common
chr01	E	1p31.1	2	72625000	1843	fs	fs common
chr01	E	1p31.1	2	72875000	1615	fs	fs common
chr01	E	1p31.1	2	73125000	2374	fs	fs common
chr01	E	1p31.1	2	73375000	2681	fs	fs common
chr01	E	1p31.1	2	73625000	2309	fs	fs common
chr01	E	1p31.1	2	73875000	1454	fs	fs common
chr01	E	1p31.1	2	74125000	3507	fs	fs common
chr01	E	1p31.1	2	74375000	1198	fs	fs common
chr01	E	1p31.1	2	74625000	1468	fs	fs common
chr01	E	1p31.1	2	74875000	3062	fs	fs common
chr01	E	1p31.1	2	75125000	1797	fs	fs common
chr01	E	1p31.1	2	75375000	1688	fs	fs common
chr01	E	1p31.1	2	75625000	1631	fs	fs common
chr01	E	1p31.1	2	75875000	1988	fs	fs common
chr01	E	1p31.1	2	76125000	3272	fs	fs common
chr01	E	1p31.1	2	76375000	2381	fs	fs common
chr01	E	1p31.1	2	76625000	1676	fs	fs common
chr01	E	1p31.1	2	76875000	1840	fs	fs common
chr01	E	1p31.1	2	77125000	1729	fs	fs common
chr01	E	1p31.1	2	77375000	1737	fs	fs common
chr01	E	1p31.1	2	77625000	4505	fs	fs common
chr01	E	1p31.1	2	77875000	2774	fs	fs common
chr01	E	1p31.1	2	78125000	4565	fs	fs common
chr01	E	1p31.1	2	78375000	2437	fs	fs common
chr01	E	1p31.1	2	78625000	2547	fs	fs common
chr01	E	1p31.1	2	78875000	1917	fs	fs common
chr01	E	1p31.1	2	79125000	1927	fs	fs common
chr01	E	1p31.1	2	79375000	3615	fs	fs common
chr01	E	1p31.1	2	79625000	3488	fs	fs common

chr01	E	1p31.1	2	79875000	3551	fs	fs common
chr01	E	1p31.1	2	80125000	2727	fs	fs common
chr01	E	1p31.1	2	80375000	1925	fs	fs common
chr01	E	1p31.1	2	80625000	4172	fs	fs common
chr01	E	1p31.1	2	80875000	2207	fs	fs common
chr01	E	1p31.1	2	81125000	1786	fs	fs common
chr01	E	1p31.1	2	81375000	1719	fs	fs common
chr01	E	1p31.1	2	81625000	2394	fs	fs common
chr01	E	1p31.1	2	81875000	2038	fs	fs common
chr01	E	1p31.1	2	82125000	2906	fs	fs common
chr01	E	1p31.1	2	82375000	1373	fs	fs common
chr01	E	1p31.1	2	82625000	2362	fs	fs common
chr01	E	1p31.1	2	82875000	2159	fs	fs common
chr01	E	1p31.1	2	83125000	3117	fs	fs common
chr01	E	1p31.1	2	83375000	2499	fs	fs common
chr01	E	1p31.1	2	83625000	1260	fs	fs common
chr01	E	1p31.1	2	83875000	1624	fs	fs common
chr01	E	1p31.1	2	84125000	3160	fs	fs common
chr01	E	1p31.1	2	84375000	1585	fs	fs common
chr01	E	1p22.3	1	84625000	1533	fs	fs common
chr01	E	1p22.3	1	84875000	2100	fs	fs common
chr01	E	1p22.3	1	85125000	3210	fs	fs common
chr01	E	1p22.3	1	85375000	2462	fs	fs common
chr01	E	1p22.3	1	85625000	1754	fs	fs common
chr01	E	1p22.3	1	85875000	2127	fs	fs common
chr01	E	1p22.3	1	86125000	1438	fs	fs common
chr01	E	1p22.3	1	86375000	3334	fs	fs common
chr01	E	1p22.3	1	86625000	1665	fs	fs common
chr01	E	1p22.3	1	86875000	4362	fs	fs common
chr01	E	1p22.3	1	87125000	3346	fs	fs common
chr01	E	1p22.3	1	87375000	1520	fs	fs common
chr01	E	1p22.3	1	87625000	1209	fs	fs common
chr01	E	1p22.3	1	87875000	1880	fs	fs common
chr01	E	1p22.3	1	88125000	2141	fs	fs common
chr01	E	1p22.2	2	88375000	1616	fs	fs common
chr01	E	1p22.2	2	88625000	3357	fs	fs common

chr01	E	1p22.2	2	88875000	2238	fs	fs common
chr01	E	1p22.2	2	89125000	3103	fs	fs common
chr01	E	1p22.2	2	89375000	2176	fs	fs common
chr01	E	1p22.2	2	89625000	1351	fs	fs common
chr01	E	1p22.2	2	89875000	2202	fs	fs common
chr01	E	1p22.2	2	90125000	1680	fs	fs common
chr01	E	1p22.2	2	90375000	2243	fs	fs common
chr01	E	1p22.2	2	90625000	3167	fs	fs common
chr01	E	1p22.2	2	90875000	2012	fs	fs common
chr01	E	1p22.2	2	91125000	1627	fs	fs common
chr01	E	1p22.2	2	91375000	5087	fs	fs common
chr01	E	1p22.2	2	91625000	3445	fs	fs common
chr01	E	1p22.1	2	91875000	3217	fs	fs common
chr01	E	1p22.1	2	92125000	3706	fs	fs common
chr01	E	1p22.1	2	92375000	2028	fs	fs common
chr01	E	1p22.1	2	92625000	2369	fs	fs common
chr01	E	1p22.1	2	92875000	3002	fs	fs common
chr01	E	1p22.1	2	93125000	1548	fs	fs common
chr01	E	1p22.1	2	93375000	2424	fs	fs common
chr01	E	1p22.1	2	93625000	2853	fs	fs common
chr01	E	1p22.1	2	93875000	1740	fs	fs common
chr01	E	1p22.1	2	94125000	2083	fs	fs common
chr01	E	1p22.1	2	94375000	1677	fs	fs common
chr01	B	1p21.3	.	94625000	2174	fs	fs common and rare
chr01	B	1p21.3	.	94875000	2810	fs	fs common and rare
chr01	B	1p21.3	.	95125000	1844	fs	fs common and rare
chr01	B	1p21.3	.	95375000	2651	fs	fs common and rare
chr01	B	1p21.3	.	95625000	2351	fs	fs common and rare
chr01	B	1p21.3	.	95875000	2312	fs	fs common and rare
chr01	B	1p21.3	.	96125000	1922	fs	fs common and rare
chr01	B	1p21.3	.	96375000	3230	fs	fs common and rare
chr01	B	1p21.3	.	96625000	2030	fs	fs common and rare
chr01	B	1p21.3	.	96875000	3005	fs	fs common and rare
chr01	B	1p21.3	.	97125000	1990	fs	fs common and rare
chr01	B	1p21.3	.	97375000	2653	fs	fs common and rare
chr01	B	1p21.3	.	97625000	2539	fs	fs common and rare

chr01	B	1p21.3	.	97875000	1736	fs	fs common and rare
chr01	B	1p21.3	.	98125000	1512	fs	fs common and rare
chr01	B	1p21.3	.	98375000	2110	fs	fs common and rare
chr01	B	1p21.3	.	98625000	2698	fs	fs common and rare
chr01	B	1p21.3	.	98875000	1795	fs	fs common and rare
chr01	B	1p21.3	.	99125000	1783	fs	fs common and rare
chr01	B	1p21.3	.	99375000	2727	fs	fs common and rare
chr01	B	1p21.3	.	99625000	2803	fs	fs common and rare
chr01	B	1p21.2	.	99875000	3049	fs	fs common and rare
chr01	B	1p21.2	.	100000000	4337	fs	fs common and rare
chr01	B	1p21.2	.	100000000	2176	fs	fs common and rare
chr01	B	1p21.2	.	101000000	2847	fs	fs common and rare
chr01	B	1p21.2	.	101000000	1808	fs	fs common and rare
chr01	B	1p21.2	.	101000000	1669	fs	fs common and rare
chr01	B	1p21.2	.	101000000	3065	fs	fs common and rare
chr01	B	1p21.2	.	102000000	3848	fs	fs common and rare
chr01	B	1p21.2	.	102000000	1320	fs	fs common and rare
chr01	E	1p21.1	1	102000000	1687	nofs	nofs
chr01	E	1p21.1	1	102000000	2915	nofs	nofs
chr01	E	1p21.1	1	103000000	2128	nofs	nofs
chr01	E	1p21.1	1	103000000	3736	nofs	nofs
chr01	E	1p21.1	1	103000000	2336	nofs	nofs
chr01	E	1p21.1	1	103000000	3504	nofs	nofs
chr01	E	1p21.1	1	104000000	1249	nofs	nofs
chr01	E	1p21.1	1	104000000	1846	nofs	nofs
chr01	E	1p21.1	1	104000000	15167	nofs	nofs
chr01	E	1p21.1	1	104000000	3601	nofs	nofs
chr01	E	1p21.1	1	105000000	1746	nofs	nofs
chr01	E	1p21.1	1	105000000	2695	nofs	nofs
chr01	E	1p21.1	1	105000000	4477	nofs	nofs
chr01	E	1p21.1	1	105000000	3078	nofs	nofs
chr01	E	1p21.1	1	106000000	2507	nofs	nofs
chr01	E	1p21.1	1	106000000	2763	nofs	nofs
chr01	E	1p21.1	1	106000000	3590	nofs	nofs
chr01	E	1p21.1	1	106000000	2379	nofs	nofs
chr01	E	1p21.1	1	107000000	2999	nofs	nofs

chr01	E	1p21.1	1	107000000	1964	nofs	nofs
chr01	E	1p13.3	1	107000000	2500	nofs	nofs
chr01	E	1p13.3	1	107000000	2360	nofs	nofs
chr01	E	1p13.3	1	108000000	1854	nofs	nofs
chr01	E	1p13.3	1	108000000	1258	nofs	nofs
chr01	E	1p13.3	1	108000000	1485	nofs	nofs
chr01	E	1p13.3	1	108000000	3192	nofs	nofs
chr01	E	1p13.3	1	109000000	4952	nofs	nofs
chr01	E	1p13.3	1	109000000	4453	nofs	nofs
chr01	E	1p13.3	1	109000000	3222	nofs	nofs
chr01	E	1p13.3	1	109000000	4318	nofs	nofs
chr01	E	1p13.3	1	110000000	2009	nofs	nofs
chr01	E	1p13.3	1	110000000	2047	nofs	nofs
chr01	E	1p13.3	1	110000000	1869	nofs	nofs
chr01	E	1p13.3	1	110000000	2520	nofs	nofs
chr01	E	1p13.3	1	111000000	1914	nofs	nofs
chr01	E	1p13.3	1	111000000	2399	nofs	nofs
chr01	E	1p13.3	1	111000000	1355	nofs	nofs
chr01	E	1p13.3	1	111000000	1430	nofs	nofs
chr01	E	1p13.3	1	112000000	1532	nofs	nofs
chr01	E	1p13.2	2	112000000	4042	nofs	nofs
chr01	E	1p13.2	2	112000000	1442	nofs	nofs
chr01	E	1p13.2	2	112000000	3428	nofs	nofs
chr01	E	1p13.2	2	113000000	4475	nofs	nofs
chr01	E	1p13.2	2	113000000	3266	nofs	nofs
chr01	E	1p13.2	2	113000000	7497	nofs	nofs
chr01	E	1p13.2	2	113000000	5132	nofs	nofs
chr01	E	1p13.2	2	114000000	2432	nofs	nofs
chr01	E	1p13.2	2	114000000	2160	nofs	nofs
chr01	E	1p13.2	2	114000000	2213	nofs	nofs
chr01	E	1p13.2	2	114000000	2947	nofs	nofs
chr01	E	1p13.2	2	115000000	3728	nofs	nofs
chr01	E	1p13.2	2	115000000	2533	nofs	nofs
chr01	E	1p13.1	1	115000000	2797	nofs	nofs
chr01	E	1p13.1	1	115000000	1379	nofs	nofs
chr01	E	1p13.1	1	116000000	3562	nofs	nofs

chr01	E	1p13.1	1	116000000	1844	nofs	nofs
chr01	E	1p13.1	1	116000000	2378	nofs	nofs
chr01	E	1p13.1	1	116000000	2597	nofs	nofs
chr01	E	1p13.1	1	117000000	2439	nofs	nofs
chr01	E	1p13.1	1	117000000	1998	nofs	nofs
chr01	E	1p13.1	1	117000000	2011	nofs	nofs
chr01	E	1p13.1	1	117000000	2309	nofs	nofs
chr01	B	1p12	.	118000000	3930	nofs	nofs
chr01	B	1p12	.	118000000	2249	nofs	nofs
chr01	C	1p12	.	118000000	1183	nofs	nofs
chr01	C	1p12	.	118000000	3021	nofs	nofs
chr01	C	1p12	.	119000000	3729	nofs	nofs
chr01	C	1p12	.	119000000	2383	nofs	nofs
chr01	C	1p12	.	119000000	1967	nofs	nofs
chr01	C	1p12	.	119000000	2904	nofs	nofs
chr01	C	1p12	.	120000000	3251	nofs	nofs
chr01	C	1p11	.	120000000	2010	nofs	nofs
chr01	C	1p11	.	120000000	2034	nofs	nofs
chr01	C	1p11	.	120000000	1283	nofs	nofs
chr01	C	1p11	.	121000000	1253	nofs	nofs
chr01	C	1p11	.	121000000	40583	nofs	nofs
chr01	C	1p11	.	121000000	95145	nofs	nofs
chr01	C		.	121000000	0	nofs	nofs
chr01	C		.	122000000	0	nofs	nofs
chr01	C		.	122000000	0	nofs	nofs
chr01	C		.	122000000	0	nofs	nofs
chr01	C		.	122000000	0	nofs	nofs
chr01	C		.	123000000	0	nofs	nofs
chr01	C		.	123000000	0	nofs	nofs
chr01	C		.	123000000	0	nofs	nofs
chr01	C		.	123000000	0	nofs	nofs
chr01	C		.	124000000	0	nofs	nofs
chr01	C		.	124000000	0	nofs	nofs
chr01	C		.	124000000	0	nofs	nofs
chr01	C		.	124000000	0	nofs	nofs
chr01	C		.	125000000	0	nofs	nofs

chr01	C	.	125000000	0	nofs	nofs
chr01	C	.	125000000	0	nofs	nofs
chr01	C	.	125000000	0	nofs	nofs
chr01	C	.	126000000	0	nofs	nofs
chr01	C	.	126000000	0	nofs	nofs
chr01	C	.	126000000	0	nofs	nofs
chr01	C	.	126000000	0	nofs	nofs
chr01	C	.	127000000	0	nofs	nofs
chr01	C	.	127000000	0	nofs	nofs
chr01	C	.	127000000	0	nofs	nofs
chr01	C	.	127000000	0	nofs	nofs
chr01	C	.	128000000	0	nofs	nofs
chr01	C	.	128000000	0	nofs	nofs
chr01	C	.	128000000	0	nofs	nofs
chr01	C	.	128000000	0	nofs	nofs
chr01	C	.	129000000	0	nofs	nofs
chr01	C	.	129000000	0	nofs	nofs
chr01	C	.	129000000	0	nofs	nofs
chr01	C	.	129000000	0	nofs	nofs
chr01	C	.	130000000	0	nofs	nofs
chr01	C	.	130000000	0	nofs	nofs
chr01	C	.	130000000	0	nofs	nofs
chr01	C	.	130000000	0	nofs	nofs
chr01	C	.	131000000	0	nofs	nofs
chr01	C	.	131000000	0	nofs	nofs
chr01	C	.	131000000	0	nofs	nofs
chr01	C	.	131000000	0	nofs	nofs
chr01	C	.	132000000	0	nofs	nofs
chr01	C	.	132000000	0	nofs	nofs
chr01	C	.	132000000	0	nofs	nofs
chr01	C	.	132000000	0	nofs	nofs
chr01	C	.	133000000	0	nofs	nofs
chr01	C	.	133000000	0	nofs	nofs
chr01	C	.	133000000	0	nofs	nofs
chr01	C	.	133000000	0	nofs	nofs
chr01	C	.	134000000	0	nofs	nofs

chr01	C		.	134000000	0	nofs	nofs
chr01	C		.	134000000	0	nofs	nofs
chr01	C		.	134000000	0	nofs	nofs
chr01	C		.	135000000	0	nofs	nofs
chr01	C		.	135000000	0	nofs	nofs
chr01	C		.	135000000	0	nofs	nofs
chr01	C		.	135000000	0	nofs	nofs
chr01	C		.	136000000	0	nofs	nofs
chr01	C		.	136000000	0	nofs	nofs
chr01	C		.	136000000	0	nofs	nofs
chr01	C		.	136000000	0	nofs	nofs
chr01	C		.	137000000	0	nofs	nofs
chr01	C		.	137000000	0	nofs	nofs
chr01	C		.	137000000	0	nofs	nofs
chr01	C		.	137000000	0	nofs	nofs
chr01	C		.	138000000	0	nofs	nofs
chr01	C		.	138000000	0	nofs	nofs
chr01	C		.	138000000	0	nofs	nofs
chr01	C		.	138000000	0	nofs	nofs
chr01	C		.	139000000	0	nofs	nofs
chr01	C		.	139000000	0	nofs	nofs
chr01	C		.	139000000	0	nofs	nofs
chr01	C		.	139000000	0	nofs	nofs
chr01	C		.	140000000	0	nofs	nofs
chr01	C		.	140000000	0	nofs	nofs
chr01	C		.	140000000	0	nofs	nofs
chr01	C		.	140000000	0	nofs	nofs
chr01	C		.	141000000	0	nofs	nofs
chr01	C		.	141000000	0	nofs	nofs
chr01	C		.	141000000	0	nofs	nofs
chr01	C		.	141000000	1306	nofs	nofs
chr01	C	1q12	.	142000000	934	fs	fs common
chr01	C	1q12	.	142000000	1007	fs	fs common
chr01	C	1q12	.	142000000	948	fs	fs common
chr01	C	1q12	.	142000000	948	fs	fs common
chr01	C	1q12	.	142000000	1462	fs	fs common

chr01	C	1q21.1	.	143000000	1946	fs	fs common
chr01	C	1q21.1	.	143000000	3714	fs	fs common
chr01	C	1q21.1	.	143000000	3995	fs	fs common
chr01	C	1q21.1	.	143000000	2034	fs	fs common
chr01	C	1q21.1	.	144000000	1750	fs	fs common
chr01	C	1q21.1	.	144000000	2705	fs	fs common
chr01	C	1q21.1	.	144000000	2002	fs	fs common
chr01	C	1q21.1	.	144000000	2267	fs	fs common
chr01	B	1q21.1	.	145000000	4231	fs	fs common
chr01	B	1q21.1	.	145000000	3464	fs	fs common
chr01	B	1q21.1	.	145000000	1328	fs	fs common
chr01	B	1q21.1	.	145000000	2314	fs	fs common
chr01	B	1q21.1	.	146000000	5995	fs	fs common
chr01	B	1q21.1	.	146000000	7754	fs	fs common
chr01	B	1q21.1	.	146000000	1165	fs	fs common
chr01	B	1q21.1	.	146000000	1471	fs	fs common
chr01	B	1q21.1	.	147000000	2947	fs	fs common
chr01	B	1q21.2	.	147000000	2736	fs	fs common
chr01	B	1q21.2	.	147000000	4470	fs	fs common
chr01	B	1q21.2	.	147000000	6229	fs	fs common
chr01	B	1q21.2	.	148000000	6335	fs	fs common
chr01	B	1q21.2	.	148000000	6691	fs	fs common
chr01	E	1q21.3	1	148000000	4274	fs	fs common
chr01	E	1q21.3	1	148000000	4984	fs	fs common
chr01	E	1q21.3	1	149000000	2491	fs	fs common
chr01	E	1q21.3	1	149000000	9532	fs	fs common
chr01	E	1q21.3	1	149000000	10338	fs	fs common
chr01	E	1q21.3	1	149000000	4174	fs	fs common
chr01	E	1q21.3	1	150000000	4347	fs	fs common
chr01	E	1q21.3	1	150000000	2147	fs	fs common
chr01	E	1q21.3	1	150000000	4256	fs	fs common
chr01	E	1q21.3	1	150000000	5316	fs	fs common
chr01	E	1q21.3	1	151000000	4392	fs	fs common
chr01	E	1q21.3	1	151000000	5428	fs	fs common
chr01	E	1q21.3	1	151000000	3734	fs	fs common
chr01	E	1q21.3	1	151000000	3390	fs	fs common

chr01	B	1q22	.	152000000	3027	nofs	nofs
chr01	B	1q22	.	152000000	4673	nofs	nofs
chr01	B	1q22	.	152000000	6803	nofs	nofs
chr01	B	1q22	.	152000000	4692	nofs	nofs
chr01	B	1q22	.	153000000	5598	nofs	nofs
chr01	B	1q22	.	153000000	3483	nofs	nofs
chr01	B	1q22	.	153000000	6095	nofs	nofs
chr01	B	1q22	.	153000000	4308	nofs	nofs
chr01	E	1q23	4	154000000	3268	nofs	nofs
chr01	E	1q23	4	154000000	2283	nofs	nofs
chr01	E	1q23	4	154000000	3367	nofs	nofs
chr01	E	1q23	4	154000000	2105	nofs	nofs
chr01	E	1q23	4	155000000	1669	nofs	nofs
chr01	E	1q23	4	155000000	1722	nofs	nofs
chr01	E	1q23	4	155000000	1741	nofs	nofs
chr01	E	1q23	4	155000000	2344	nofs	nofs
chr01	E	1q23	4	156000000	1991	nofs	nofs
chr01	E	1q23	4	156000000	2248	nofs	nofs
chr01	E	1q23	4	156000000	1756	nofs	nofs
chr01	E	1q23	4	156000000	2186	nofs	nofs
chr01	E	1q23	4	157000000	1941	nofs	nofs
chr01	E	1q23	4	157000000	2874	nofs	nofs
chr01	E	1q23	4	157000000	2263	nofs	nofs
chr01	E	1q23	4	157000000	3701	nofs	nofs
chr01	E	1q23	4	158000000	3629	nofs	nofs
chr01	E	1q23	4	158000000	7018	nofs	nofs
chr01	E	1q23	4	158000000	5522	nofs	nofs
chr01	E	1q23	4	158000000	2566	nofs	nofs
chr01	E	1q23	4	159000000	2490	nofs	nofs
chr01	E	1q23	4	159000000	3117	nofs	nofs
chr01	E	1q23	4	159000000	3075	nofs	nofs
chr01	E	1q23	4	159000000	3197	nofs	nofs
chr01	E	1q23	4	160000000	2251	nofs	nofs
chr01	E	1q23	4	160000000	2345	nofs	nofs
chr01	E	1q23	4	160000000	3501	nofs	nofs
chr01	E	1q23	4	160000000	3158	nofs	nofs

chr01	E	1q23	4	161000000	1249	nofs	nofs
chr01	E	1q23	4	161000000	1690	nofs	nofs
chr01	E	1q23	4	161000000	3021	nofs	nofs
chr01	E	1q23	4	161000000	2066	nofs	nofs
chr01	E	1q23	4	162000000	1694	nofs	nofs
chr01	E	1q23	4	162000000	1894	nofs	nofs
chr01	E	1q23	4	162000000	1905	nofs	nofs
chr01	E	1q24	3	162000000	3165	nofs	nofs
chr01	E	1q24	3	163000000	4098	nofs	nofs
chr01	E	1q24	3	163000000	2998	nofs	nofs
chr01	E	1q24	3	163000000	2531	nofs	nofs
chr01	E	1q24	3	163000000	2364	nofs	nofs
chr01	E	1q24	3	164000000	1963	nofs	nofs
chr01	E	1q24	3	164000000	3483	nofs	nofs
chr01	E	1q24	3	164000000	2174	nofs	nofs
chr01	E	1q24	3	164000000	2869	nofs	nofs
chr01	E	1q24	3	165000000	2818	nofs	nofs
chr01	E	1q24	3	165000000	4739	nofs	nofs
chr01	E	1q24	3	165000000	3074	nofs	nofs
chr01	E	1q24	3	165000000	2337	nofs	nofs
chr01	E	1q24	3	166000000	4490	nofs	nofs
chr01	E	1q24	3	166000000	2426	nofs	nofs
chr01	E	1q24	3	166000000	3795	nofs	nofs
chr01	E	1q24	3	166000000	4151	nofs	nofs
chr01	E	1q24	3	167000000	1878	nofs	nofs
chr01	E	1q24	3	167000000	2327	nofs	nofs
chr01	E	1q24	3	167000000	2224	nofs	nofs
chr01	E	1q24	3	167000000	2227	nofs	nofs
chr01	E	1q24	3	168000000	2394	nofs	nofs
chr01	E	1q24	3	168000000	2994	nofs	nofs
chr01	E	1q24	3	168000000	3189	nofs	nofs
chr01	E	1q24	3	168000000	3399	nofs	nofs
chr01	E	1q24	3	169000000	2583	nofs	nofs
chr01	E	1q24	3	169000000	1722	nofs	nofs
chr01	E	1q24	3	169000000	2378	nofs	nofs
chr01	E	1q24	3	169000000	3254	nofs	nofs

chr01	E	1q24	3	170000000	2421	nofs	nofs
chr01	E	1q25	2	170000000	1824	nofs	nofs
chr01	E	1q25	2	170000000	2673	fs	fs common
chr01	E	1q25	2	170000000	3278	fs	fs common
chr01	E	1q25	2	171000000	2529	fs	fs common
chr01	E	1q25	2	171000000	3840	fs	fs common
chr01	E	1q25	2	171000000	3983	fs	fs common
chr01	E	1q25	2	171000000	4066	fs	fs common
chr01	E	1q25	2	172000000	3302	fs	fs common
chr01	E	1q25	2	172000000	5783	fs	fs common
chr01	E	1q25	2	172000000	2553	fs	fs common
chr01	E	1q25	2	172000000	2495	fs	fs common
chr01	E	1q25	2	173000000	1267	fs	fs common
chr01	E	1q25	2	173000000	3544	fs	fs common
chr01	E	1q25	2	173000000	3387	nofs	nofs
chr01	E	1q25	2	173000000	3360	nofs	nofs
chr01	E	1q25	2	174000000	2232	nofs	nofs
chr01	E	1q25	2	174000000	1922	nofs	nofs
chr01	E	1q25	2	174000000	2608	nofs	nofs
chr01	E	1q25	2	174000000	1266	nofs	nofs
chr01	E	1q25	2	175000000	2525	nofs	nofs
chr01	E	1q25	2	175000000	3057	nofs	nofs
chr01	E	1q25	2	175000000	1767	nofs	nofs
chr01	E	1q25	2	175000000	2979	nofs	nofs
chr01	E	1q25	2	176000000	2826	nofs	nofs
chr01	E	1q25	2	176000000	3306	nofs	nofs
chr01	E	1q25	2	176000000	2900	nofs	nofs
chr01	E	1q25	2	176000000	1200	nofs	nofs
chr01	E	1q25	2	177000000	2299	nofs	nofs
chr01	E	1q25	2	177000000	2488	nofs	nofs
chr01	E	1q25	2	177000000	1610	nofs	nofs
chr01	E	1q25	2	177000000	3285	nofs	nofs
chr01	E	1q25	2	178000000	2415	nofs	nofs
chr01	E	1q25	2	178000000	2770	nofs	nofs
chr01	E	1q25	2	178000000	3692	nofs	nofs
chr01	E	1q25	2	178000000	2295	nofs	nofs

chr01	E	1q25	2	179000000	2032	nofs	nofs
chr01	E	1q25	2	179000000	1846	nofs	nofs
chr01	E	1q25	2	179000000	4666	nofs	nofs
chr01	E	1q25	2	179000000	3572	nofs	nofs
chr01	E	1q25	2	180000000	3013	nofs	nofs
chr01	E	1q25	2	180000000	2810	nofs	nofs
chr01	E	1q25	2	180000000	2753	nofs	nofs
chr01	E	1q25	2	180000000	2031	nofs	nofs
chr01	E	1q25	2	181000000	1737	nofs	nofs
chr01	E	1q25	2	181000000	1843	nofs	nofs
chr01	E	1q25	2	181000000	2639	nofs	nofs
chr01	E	1q25	2	181000000	1978	nofs	nofs
chr01	E	1q25	2	182000000	2242	nofs	nofs
chr01	E	1q25	2	182000000	1590	nofs	nofs
chr01	E	1q25	2	182000000	3180	nofs	nofs
chr01	E	1q25	2	182000000	2728	nofs	nofs
chr01	E	1q25	2	183000000	2289	nofs	nofs
chr01	E	1q31	3	183000000	1929	fs	fs common
chr01	E	1q31	3	183000000	3794	fs	fs common
chr01	E	1q31	3	183000000	2972	fs	fs common
chr01	E	1q31	3	184000000	3573	fs	fs common
chr01	E	1q31	3	184000000	2489	fs	fs common
chr01	E	1q31	3	184000000	1834	fs	fs common
chr01	E	1q31	3	184000000	4884	fs	fs common
chr01	E	1q31	3	185000000	1991	fs	fs common
chr01	E	1q31	3	185000000	2989	fs	fs common
chr01	E	1q31	3	185000000	2565	fs	fs common
chr01	E	1q31	3	185000000	2525	fs	fs common
chr01	E	1q31	3	186000000	4107	fs	fs common
chr01	E	1q31	3	186000000	3654	fs	fs common
chr01	E	1q31	3	186000000	2587	fs	fs common
chr01	E	1q31	3	186000000	4091	fs	fs common
chr01	E	1q31	3	187000000	2747	fs	fs common
chr01	E	1q31	3	187000000	2219	fs	fs common
chr01	E	1q31	3	187000000	3047	fs	fs common
chr01	E	1q31	3	187000000	1995	fs	fs common

chr01	E	1q31	3	188000000	3005	fs	fs common
chr01	E	1q31	3	188000000	2836	fs	fs common
chr01	E	1q31	3	188000000	1891	fs	fs common
chr01	E	1q31	3	188000000	2650	fs	fs common
chr01	E	1q31	3	189000000	3082	fs	fs common
chr01	E	1q31	3	189000000	2295	fs	fs common
chr01	E	1q31	3	189000000	2648	fs	fs common
chr01	E	1q31	3	189000000	3354	fs	fs common
chr01	E	1q31	3	190000000	3127	fs	fs common
chr01	E	1q31	3	190000000	3181	fs	fs common
chr01	E	1q31	3	190000000	2627	fs	fs common
chr01	E	1q31	3	190000000	2440	fs	fs common
chr01	E	1q31	3	191000000	2576	fs	fs common
chr01	E	1q31	3	191000000	2278	fs	fs common
chr01	E	1q31	3	191000000	3982	fs	fs common
chr01	E	1q31	3	191000000	3155	fs	fs common
chr01	E	1q31	3	192000000	2140	fs	fs common
chr01	E	1q31	3	192000000	3314	fs	fs common
chr01	E	1q31	3	192000000	2346	fs	fs common
chr01	E	1q31	3	192000000	2916	fs	fs common
chr01	E	1q31	3	193000000	2662	fs	fs common
chr01	E	1q31	3	193000000	2335	fs	fs common
chr01	E	1q31	3	193000000	2675	fs	fs common
chr01	E	1q31	3	193000000	2762	fs	fs common
chr01	E	1q31	3	194000000	4181	fs	fs common
chr01	E	1q31	3	194000000	1782	fs	fs common
chr01	E	1q31	3	194000000	3547	fs	fs common
chr01	E	1q31	3	194000000	2474	fs	fs common
chr01	E	1q31	3	195000000	2771	fs	fs common
chr01	E	1q31	3	195000000	1460	fs	fs common
chr01	E	1q31	3	195000000	3199	fs	fs common
chr01	E	1q31	3	195000000	2528	fs	fs common
chr01	E	1q31	3	196000000	1648	fs	fs common
chr01	E	1q31	3	196000000	2731	fs	fs common
chr01	E	1q32.1	4	196000000	2006	nofs	nofs
chr01	E	1q32.1	4	196000000	2633	nofs	nofs

chr01	E	1q32.1	4	197000000	3879	nofs	nofs
chr01	E	1q32.1	4	197000000	3750	nofs	nofs
chr01	E	1q32.1	4	197000000	4854	nofs	nofs
chr01	E	1q32.1	4	197000000	3460	nofs	nofs
chr01	E	1q32.1	4	198000000	3992	nofs	nofs
chr01	E	1q32.1	4	198000000	7758	nofs	nofs
chr01	E	1q32.1	4	198000000	2548	nofs	nofs
chr01	E	1q32.1	4	198000000	3795	nofs	nofs
chr01	E	1q32.1	4	199000000	4402	nofs	nofs
chr01	E	1q32.1	4	199000000	10305	nofs	nofs
chr01	E	1q32.1	4	199000000	3382	nofs	nofs
chr01	E	1q32.1	4	199000000	3291	nofs	nofs
chr01	E	1q32.1	4	200000000	2927	nofs	nofs
chr01	E	1q32.1	4	200000000	1976	nofs	nofs
chr01	E	1q32.1	4	200000000	3884	nofs	nofs
chr01	E	1q32.1	4	200000000	4737	nofs	nofs
chr01	E	1q32.1	4	201000000	5293	nofs	nofs
chr01	E	1q32.1	4	201000000	4458	nofs	nofs
chr01	E	1q32.1	4	201000000	2407	nofs	nofs
chr01	E	1q32.1	4	201000000	3083	nofs	nofs
chr01	E	1q32.1	4	202000000	1983	nofs	nofs
chr01	E	1q32.1	4	202000000	3281	nofs	nofs
chr01	E	1q32.1	4	202000000	2500	nofs	nofs
chr01	E	1q32.1	4	202000000	2850	nofs	nofs
chr01	E	1q32.1	4	203000000	2565	nofs	nofs
chr01	E	1q32.1	4	203000000	3062	nofs	nofs
chr01	E	1q32.1	4	203000000	5193	nofs	nofs
chr01	E	1q32.1	4	203000000	3787	nofs	nofs
chr01	E	1q32.1	4	204000000	2553	nofs	nofs
chr01	E	1q32.2	6	204000000	3595	nofs	nofs
chr01	E	1q32.2	6	204000000	2797	nofs	nofs
chr01	E	1q32.2	6	204000000	1487	nofs	nofs
chr01	E	1q32.2	6	205000000	1777	nofs	nofs
chr01	E	1q32.2	6	205000000	2174	nofs	nofs
chr01	E	1q32.2	6	205000000	2478	nofs	nofs
chr01	E	1q32.2	6	205000000	3302	nofs	nofs

chr01	E	1q32.2	6	206000000	2848	nofs	nofs
chr01	E	1q32.2	6	206000000	1944	nofs	nofs
chr01	E	1q32.2	6	206000000	2204	nofs	nofs
chr01	E	1q32.2	6	206000000	3791	nofs	nofs
chr01	E	1q32.2	6	207000000	1718	nofs	nofs
chr01	E	1q32.2	6	207000000	2412	nofs	nofs
chr01	E	1q32.2	6	207000000	1800	nofs	nofs
chr01	E	1q32.2	6	207000000	2412	nofs	nofs
chr01	E	1q32.2	6	208000000	3687	nofs	nofs
chr01	E	1q32.2	6	208000000	3223	nofs	nofs
chr01	E	1q32.3	1	208000000	4210	nofs	nofs
chr01	E	1q32.3	1	208000000	4494	nofs	nofs
chr01	E	1q32.3	1	209000000	2766	nofs	nofs
chr01	E	1q32.3	1	209000000	5226	nofs	nofs
chr01	E	1q32.3	1	209000000	2344	nofs	nofs
chr01	E	1q32.3	1	209000000	5937	nofs	nofs
chr01	E	1q32.3	1	210000000	4288	nofs	nofs
chr01	E	1q32.3	1	210000000	1883	nofs	nofs
chr01	E	1q32.3	1	210000000	1209	nofs	nofs
chr01	E	1q32.3	1	210000000	2103	nofs	nofs
chr01	E	1q32.3	1	211000000	2588	nofs	nofs
chr01	E	1q41	1	211000000	1876	nofs	nofs
chr01	E	1q41	1	211000000	3317	nofs	nofs
chr01	E	1q41	1	211000000	3817	nofs	nofs
chr01	E	1q41	1	212000000	2186	nofs	nofs
chr01	E	1q41	1	212000000	3803	nofs	nofs
chr01	E	1q41	1	212000000	2097	nofs	nofs
chr01	E	1q41	1	212000000	1965	nofs	nofs
chr01	E	1q41	1	213000000	2633	nofs	nofs
chr01	E	1q41	1	213000000	1690	nofs	nofs
chr01	E	1q41	1	213000000	1550	nofs	nofs
chr01	E	1q41	1	213000000	2613	nofs	nofs
chr01	E	1q41	1	214000000	1994	nofs	nofs
chr01	E	1q41	1	214000000	1346	nofs	nofs
chr01	E	1q41	1	214000000	2290	nofs	nofs
chr01	E	1q41	1	214000000	2072	nofs	nofs

chr01	E	1q41	1	215000000	1364	nofs	nofs
chr01	E	1q41	1	215000000	2173	nofs	nofs
chr01	E	1q41	1	215000000	2935	nofs	nofs
chr01	E	1q41	1	215000000	2616	nofs	nofs
chr01	E	1q41	1	216000000	1614	nofs	nofs
chr01	E	1q41	1	216000000	1889	nofs	nofs
chr01	E	1q41	1	216000000	3166	nofs	nofs
chr01	E	1q41	1	216000000	2384	nofs	nofs
chr01	E	1q41	1	217000000	3403	nofs	nofs
chr01	E	1q41	1	217000000	1997	nofs	nofs
chr01	E	1q41	1	217000000	3801	nofs	nofs
chr01	E	1q41	1	217000000	1682	nofs	nofs
chr01	E	1q41	1	218000000	3672	nofs	nofs
chr01	E	1q41	1	218000000	2116	nofs	nofs
chr01	E	1q41	1	218000000	2571	nofs	nofs
chr01	E	1q41	1	218000000	2597	nofs	nofs
chr01	E	1q41	1	219000000	4126	nofs	nofs
chr01	E	1q41	1	219000000	1665	nofs	nofs
chr01	E	1q41	1	219000000	4391	nofs	nofs
chr01	E	1q41	1	219000000	2107	nofs	nofs
chr01	E	1q41	1	220000000	3201	nofs	nofs
chr01	E	1q42.1	6	220000000	2519	fs	fs common
chr01	E	1q42.1	6	220000000	3348	fs	fs common
chr01	E	1q42.1	6	220000000	4060	fs	fs common
chr01	E	1q42.1	6	221000000	10431	fs	fs common
chr01	E	1q42.1	6	221000000	4009	fs	fs common
chr01	E	1q42.1	6	221000000	5236	fs	fs common
chr01	E	1q42.1	6	221000000	1787	fs	fs common
chr01	E	1q42.1	6	222000000	1603	fs	fs common
chr01	E	1q42.1	6	222000000	3402	fs	fs common
chr01	E	1q42.1	6	222000000	3295	fs	fs common
chr01	E	1q42.1	6	222000000	4890	fs	fs common
chr01	E	1q42.1	6	223000000	3544	fs	fs common
chr01	E	1q42.1	6	223000000	2225	fs	fs common
chr01	E	1q42.1	6	223000000	2881	fs	fs common
chr01	E	1q42.1	6	223000000	6392	fs	fs common

chr01	E	1q42.1	6	224000000	2821	fs	fs common
chr01	E	1q42.1	6	224000000	3336	fs	fs common
chr01	E	1q42.1	6	224000000	5426	fs	fs common
chr01	E	1q42.1	6	224000000	8934	fs	fs common
chr01	E	1q42.1	6	225000000	9229	fs	fs common
chr01	E	1q42.1	6	225000000	6092	fs	fs common
chr01	E	1q42.1	6	225000000	26869	fs	fs common
chr01	E	1q42.1	6	225000000	2595	fs	fs common
chr01	E	1q42.1	6	226000000	4185	fs	fs common
chr01	E	1q42.1	6	226000000	2323	fs	fs common
chr01	E	1q42.1	6	226000000	4256	fs	fs common
chr01	E	1q42.1	6	226000000	3683	fs	fs common
chr01	E	1q42.1	6	227000000	4348	fs	fs common
chr01	E	1q42.1	6	227000000	5341	fs	fs common
chr01	B	1q42.2	.	227000000	3785	fs	fs common
chr01	B	1q42.2	.	227000000	4308	fs	fs common
chr01	B	1q42.2	.	228000000	3959	fs	fs common
chr01	B	1q42.2	.	228000000	2452	fs	fs common
chr01	B	1q42.2	.	228000000	3181	fs	fs common
chr01	B	1q42.2	.	228000000	2741	fs	fs common
chr01	B	1q42.2	.	229000000	3013	fs	fs common
chr01	B	1q42.2	.	229000000	3802	fs	fs common
chr01	B	1q42.2	.	229000000	9335	fs	fs common
chr01	B	1q42.2	.	229000000	2308	fs	fs common
chr01	B	1q42.2	.	230000000	2930	fs	fs common
chr01	B	1q42.2	.	230000000	2190	fs	fs common
chr01	B	1q42.2	.	230000000	3723	fs	fs common
chr01	B	1q42.2	.	230000000	2748	fs	fs common
chr01	B	1q42.2	.	231000000	6939	fs	fs common
chr01	B	1q42.2	.	231000000	4156	fs	fs common
chr01	E	1q42.3	3	231000000	3551	fs	fs common
chr01	E	1q42.3	3	231000000	3952	fs	fs common
chr01	E	1q42.3	3	232000000	3863	fs	fs common
chr01	E	1q42.3	3	232000000	6018	fs	fs common
chr01	E	1q42.3	3	232000000	2949	fs	fs common
chr01	E	1q42.3	3	232000000	3491	fs	fs common

chr01	E	1q42.3	3	233000000	5605	fs	fs common
chr01	E	1q42.3	3	233000000	4382	fs	fs common
chr01	E	1q43	4	233000000	6499	nofs	nofs
chr01	E	1q43	4	233000000	2958	nofs	nofs
chr01	E	1q43	4	234000000	4658	nofs	nofs
chr01	E	1q43	4	234000000	3673	nofs	nofs
chr01	E	1q43	4	234000000	3941	nofs	nofs
chr01	E	1q43	4	234000000	2881	nofs	nofs
chr01	E	1q43	4	235000000	2347	nofs	nofs
chr01	E	1q43	4	235000000	4282	nofs	nofs
chr01	E	1q43	4	235000000	3861	nofs	nofs
chr01	E	1q43	4	235000000	3011	nofs	nofs
chr01	E	1q43	4	236000000	3027	nofs	nofs
chr01	E	1q43	4	236000000	2501	nofs	nofs
chr01	E	1q43	4	236000000	4647	nofs	nofs
chr01	E	1q43	4	236000000	3761	nofs	nofs
chr01	E	1q43	4	237000000	3851	nofs	nofs
chr01	E	1q43	4	237000000	2493	nofs	nofs
chr01	E	1q43	4	237000000	3267	nofs	nofs
chr01	E	1q43	4	237000000	3621	nofs	nofs
chr01	E	1q43	4	238000000	3775	nofs	nofs
chr01	E	1q43	4	238000000	2364	nofs	nofs
chr01	E	1q43	4	238000000	3267	nofs	nofs
chr01	E	1q43	4	238000000	3615	nofs	nofs
chr01	E	1q43	4	239000000	3738	nofs	nofs
chr01	E	1q43	4	239000000	3578	nofs	nofs
chr01	E	1q43	4	239000000	3638	nofs	nofs
chr01	E	1q43	4	239000000	2914	nofs	nofs
chr01	E	1q43	4	240000000	4127	nofs	nofs
chr01	E	1q44	2	240000000	4085	fs	fs common
chr01	E	1q44	2	240000000	1808	fs	fs common
chr01	E	1q44	2	240000000	2978	fs	fs common
chr01	E	1q44	2	241000000	2795	fs	fs common
chr01	E	1q44	2	241000000	5423	fs	fs common
chr01	E	1q44	2	241000000	2717	fs	fs common
chr01	E	1q44	2	241000000	4257	fs	fs common

chr01	E	1q44	2	242000000	3797	fs	fs common
chr01	E	1q44	2	242000000	8131	fs	fs common
chr01	T	1q44	2	242000000	7025	fs	fs common
chr01	T	1q44	2	242000000	7554	fs	fs common
chr01	T	1q44	2	243000000	8620	fs	fs common
chr01	T	1q44	2	243000000	12492	fs	fs common
chr01	T	1q44	2	243000000	7988	fs	fs common
chr01	T	1q44	2	243000000	7957	fs	fs common
chr01	T	1q44	2	244000000	10157	fs	fs common
chr01	T	1q44	2	244000000	7995	fs	fs common
chr01	T	1q44	2	244000000	3753	fs	fs common
chr01	T	1q44	2	244000000	4474	fs	fs common
chr01	T	1q44	2	245000000	4306	fs	fs common
chr01	T	1q44	2	245000000	5529	fs	fs common
chr01	T	1q44	2	245000000	5455	fs	fs common
chr02	T	2p25.3	2	125000	10555	nofs	nofs
chr02	T	2p25.3	2	375000	23974	nofs	nofs
chr02	T	2p25.3	2	625000	16623	nofs	nofs
chr02	T	2p25.3	2	875000	25693	nofs	nofs
chr02	T	2p25.3	2	1125000	23321	nofs	nofs
chr02	T	2p25.3	2	1375000	28119	nofs	nofs
chr02	T	2p25.3	2	1625000	37144	nofs	nofs
chr02	T	2p25.3	2	1875000	15891	nofs	nofs
chr02	T	2p25.3	2	2125000	14222	nofs	nofs
chr02	T	2p25.3	2	2375000	6696	nofs	nofs
chr02	T	2p25.3	2	2625000	15286	nofs	nofs
chr02	T	2p25.3	2	2875000	14254	nofs	nofs
chr02	T	2p25.3	2	3125000	11598	nofs	nofs
chr02	E	2p25.3	2	3375000	6600	nofs	nofs
chr02	E	2p25.3	2	3625000	4640	nofs	nofs
chr02	E	2p25.3	2	3875000	5609	nofs	nofs
chr02	B	2p25.2	.	4125000	3574	nofs	nofs
chr02	B	2p25.2	.	4375000	2590	nofs	nofs
chr02	B	2p25.2	.	4625000	9026	nofs	nofs
chr02	B	2p25.2	.	4875000	25059	nofs	nofs
chr02	B	2p25.2	.	5125000	4708	nofs	nofs

chr02	B	2p25.2	.	5375000	4755	nofs	nofs
chr02	B	2p25.2	.	5625000	4340	nofs	nofs
chr02	B	2p25.2	.	5875000	4671	nofs	nofs
chr02	B	2p25.2	.	6125000	2929	nofs	nofs
chr02	B	2p25.2	.	6375000	4601	nofs	nofs
chr02	B	2p25.2	.	6625000	3778	nofs	nofs
chr02	B	2p25.2	.	6875000	3949	nofs	nofs
chr02	B	2p25.2	.	7125000	5841	nofs	nofs
chr02	B	2p25.1	.	7375000	4411	nofs	nofs
chr02	B	2p25.1	.	7625000	4099	nofs	nofs
chr02	B	2p25.1	.	7875000	4155	nofs	nofs
chr02	B	2p25.1	.	8125000	3315	nofs	nofs
chr02	B	2p25.1	.	8375000	3370	nofs	nofs
chr02	B	2p25.1	.	8625000	4072	nofs	nofs
chr02	B	2p25.1	.	8875000	3986	nofs	nofs
chr02	B	2p25.1	.	9125000	3843	nofs	nofs
chr02	B	2p25.1	.	9375000	4176	nofs	nofs
chr02	B	2p25.1	.	9625000	3167	nofs	nofs
chr02	B	2p25.1	.	9875000	3387	nofs	nofs
chr02	B	2p25.1	.	10125000	8257	nofs	nofs
chr02	B	2p25.1	.	10375000	7542	nofs	nofs
chr02	B	2p25.1	.	10625000	7895	nofs	nofs
chr02	B	2p25.1	.	10875000	4915	nofs	nofs
chr02	B	2p25.1	.	11125000	7634	nofs	nofs
chr02	B	2p25.1	.	11375000	4184	nofs	nofs
chr02	B	2p25.1	.	11625000	3612	nofs	nofs
chr02	B	2p25.1	.	11875000	3866	nofs	nofs
chr02	B	2p25.1	.	12125000	3792	nofs	nofs
chr02	B	2p24.3	.	12375000	2207	nofs	nofs
chr02	B	2p24.3	.	12625000	3713	nofs	nofs
chr02	B	2p24.3	.	12875000	3253	nofs	nofs
chr02	B	2p24.3	.	13125000	2671	nofs	nofs
chr02	B	2p24.3	.	13375000	4509	nofs	nofs
chr02	B	2p24.3	.	13625000	3071	nofs	nofs
chr02	B	2p24.3	.	13875000	2088	nofs	nofs
chr02	B	2p24.3	.	14125000	4201	nofs	nofs

chr02	B	2p24.3	.	14375000	2640	nofs	nofs
chr02	B	2p24.3	.	14625000	1740	nofs	nofs
chr02	B	2p24.3	.	14875000	2169	nofs	nofs
chr02	B	2p24.3	.	15125000	3257	nofs	nofs
chr02	B	2p24.3	.	15375000	2184	nofs	nofs
chr02	B	2p24.3	.	15625000	3682	nofs	nofs
chr02	B	2p24.3	.	15875000	3230	nofs	nofs
chr02	B	2p24.3	.	16125000	5678	nofs	nofs
chr02	B	2p24.3	.	16375000	3026	nofs	nofs
chr02	B	2p24.3	.	16625000	1107	nofs	nofs
chr02	B	2p24.2	.	16875000	2388	fs	fs common
chr02	B	2p24.2	.	17125000	1785	fs	fs common
chr02	B	2p24.2	.	17375000	2367	fs	fs common
chr02	B	2p24.2	.	17625000	2025	fs	fs common
chr02	B	2p24.2	.	17875000	2993	fs	fs common
chr02	B	2p24.2	.	18125000	2914	fs	fs common
chr02	B	2p24.2	.	18375000	4381	fs	fs common
chr02	B	2p24.2	.	18625000	1714	fs	fs common
chr02	B	2p24.2	.	18875000	3558	fs	fs common
chr02	B	2p24.2	.	19125000	2370	fs	fs common
chr02	B	2p24.2	.	19375000	1979	fs	fs common
chr02	B	2p24.1	.	19625000	2650	nofs	nofs
chr02	B	2p24.1	.	19875000	2466	nofs	nofs
chr02	B	2p24.1	.	20125000	3066	nofs	nofs
chr02	B	2p24.1	.	20375000	4037	nofs	nofs
chr02	B	2p24.1	.	20625000	2420	nofs	nofs
chr02	B	2p24.1	.	20875000	2110	nofs	nofs
chr02	B	2p24.1	.	21125000	2673	nofs	nofs
chr02	B	2p24.1	.	21375000	2746	nofs	nofs
chr02	B	2p24.1	.	21625000	2630	nofs	nofs
chr02	B	2p24.1	.	21875000	1219	nofs	nofs
chr02	B	2p24.1	.	22125000	2725	nofs	nofs
chr02	B	2p24.1	.	22375000	2242	nofs	nofs
chr02	B	2p24.1	.	22625000	2160	nofs	nofs
chr02	B	2p24.1	.	22875000	2237	nofs	nofs
chr02	B	2p24.1	.	23125000	2072	nofs	nofs

chr02	B	2p24.1	.	23375000	2516	nofs	nofs
chr02	B	2p24.1	.	23625000	3565	nofs	nofs
chr02	E	2p23	3	23875000	2898	nofs	nofs
chr02	E	2p23	3	24125000	3264	nofs	nofs
chr02	E	2p23	3	24375000	4028	nofs	nofs
chr02	E	2p23	3	24625000	2842	nofs	nofs
chr02	E	2p23	3	24875000	2498	nofs	nofs
chr02	E	2p23	3	25125000	2494	nofs	nofs
chr02	E	2p23	3	25375000	3704	nofs	nofs
chr02	E	2p23	3	25625000	4476	nofs	nofs
chr02	E	2p23	3	25875000	4905	nofs	nofs
chr02	E	2p23	3	26125000	6651	nofs	nofs
chr02	E	2p23	3	26375000	1899	nofs	nofs
chr02	E	2p23	3	26625000	5450	nofs	nofs
chr02	E	2p23	3	26875000	3009	nofs	nofs
chr02	E	2p23	3	27125000	2393	nofs	nofs
chr02	E	2p23	3	27375000	2572	nofs	nofs
chr02	E	2p23	3	27625000	8147	nofs	nofs
chr02	E	2p23	3	27875000	3573	nofs	nofs
chr02	E	2p23	3	28125000	2525	nofs	nofs
chr02	E	2p23	3	28375000	1976	nofs	nofs
chr02	E	2p23	3	28625000	4258	nofs	nofs
chr02	E	2p23	3	28875000	1940	nofs	nofs
chr02	E	2p23	3	29125000	4371	nofs	nofs
chr02	E	2p23	3	29375000	4097	nofs	nofs
chr02	E	2p23	3	29625000	1353	nofs	nofs
chr02	E	2p23	3	29875000	2935	nofs	nofs
chr02	E	2p23	3	30125000	3075	nofs	nofs
chr02	E	2p23	3	30375000	3020	nofs	nofs
chr02	E	2p23	3	30625000	2954	nofs	nofs
chr02	E	2p23	3	30875000	1815	nofs	nofs
chr02	E	2p23	3	31125000	2532	nofs	nofs
chr02	E	2p23	3	31375000	2584	nofs	nofs
chr02	E	2p23	3	31625000	2055	nofs	nofs
chr02	E	2p23	3	31875000	3477	nofs	nofs
chr02	E	2p22	4	32125000	6459	nofs	nofs

chr02	E	2p22	4	32375000	5596	nofs	nofs
chr02	E	2p22	4	32625000	3418	nofs	nofs
chr02	E	2p22	4	32875000	2935	nofs	nofs
chr02	E	2p22	4	33125000	6063	nofs	nofs
chr02	E	2p22	4	33375000	2181	nofs	nofs
chr02	E	2p22	4	33625000	1829	nofs	nofs
chr02	E	2p22	4	33875000	4468	nofs	nofs
chr02	E	2p22	4	34125000	3848	nofs	nofs
chr02	E	2p22	4	34375000	3030	nofs	nofs
chr02	E	2p22	4	34625000	2875	nofs	nofs
chr02	E	2p22	4	34875000	4952	nofs	nofs
chr02	E	2p22	4	35125000	2487	nofs	nofs
chr02	E	2p22	4	35375000	1046	nofs	nofs
chr02	E	2p22	4	35625000	2392	nofs	nofs
chr02	E	2p22	4	35875000	3287	nofs	nofs
chr02	E	2p22	4	36125000	5370	nofs	nofs
chr02	E	2p22	4	36375000	5807	nofs	nofs
chr02	E	2p22	4	36625000	2151	nofs	nofs
chr02	E	2p22	4	36875000	2219	nofs	nofs
chr02	E	2p22	4	37125000	2066	nofs	nofs
chr02	E	2p22	4	37375000	2617	nofs	nofs
chr02	E	2p22	4	37625000	3338	nofs	nofs
chr02	E	2p22	4	37875000	1555	nofs	nofs
chr02	E	2p22	4	38125000	3650	nofs	nofs
chr02	E	2p22	4	38375000	2399	nofs	nofs
chr02	E	2p22	4	38625000	3539	nofs	nofs
chr02	E	2p22	4	38875000	5365	nofs	nofs
chr02	E	2p22	4	39125000	1845	nofs	nofs
chr02	E	2p22	4	39375000	3430	nofs	nofs
chr02	E	2p22	4	39625000	2098	nofs	nofs
chr02	E	2p22	4	39875000	2723	nofs	nofs
chr02	E	2p22	4	40125000	1981	nofs	nofs
chr02	E	2p22	4	40375000	2314	nofs	nofs
chr02	E	2p22	4	40625000	2139	nofs	nofs
chr02	E	2p22	4	40875000	2857	nofs	nofs
chr02	E	2p22	4	41125000	1745	nofs	nofs

chr02	E	2p22	4	41375000	1696	nofs	nofs
chr02	E	2p22	4	41625000	1481	nofs	nofs
chr02	E	2p22	4	41875000	4317	nofs	nofs
chr02	E	2p21	2	42125000	4563	nofs	nofs
chr02	E	2p21	2	42375000	2285	nofs	nofs
chr02	E	2p21	2	42625000	6684	nofs	nofs
chr02	E	2p21	2	42875000	2339	nofs	nofs
chr02	E	2p21	2	43125000	3245	nofs	nofs
chr02	E	2p21	2	43375000	4462	nofs	nofs
chr02	E	2p21	2	43625000	3292	nofs	nofs
chr02	E	2p21	2	43875000	8980	nofs	nofs
chr02	E	2p21	2	44125000	13350	nofs	nofs
chr02	E	2p21	2	44375000	3100	nofs	nofs
chr02	E	2p21	2	44625000	3129	nofs	nofs
chr02	E	2p21	2	44875000	1130	nofs	nofs
chr02	E	2p21	2	45125000	2682	nofs	nofs
chr02	E	2p21	2	45375000	4432	nofs	nofs
chr02	E	2p21	2	45625000	3449	nofs	nofs
chr02	E	2p21	2	45875000	2251	nofs	nofs
chr02	E	2p21	2	46125000	2247	nofs	nofs
chr02	E	2p21	2	46375000	2889	nofs	nofs
chr02	E	2p21	2	46625000	1955	nofs	nofs
chr02	E	2p21	2	46875000	2178	nofs	nofs
chr02	E	2p21	2	47125000	5045	nofs	nofs
chr02	E	2p21	2	47375000	3576	nofs	nofs
chr02	E	2p21	2	47625000	5353	nofs	nofs
chr02	E	2p16	3	47875000	2414	nofs	nofs
chr02	E	2p16	3	48125000	3920	nofs	nofs
chr02	E	2p16	3	48375000	2170	nofs	nofs
chr02	E	2p16	3	48625000	5295	nofs	nofs
chr02	E	2p16	3	48875000	2593	nofs	nofs
chr02	E	2p16	3	49125000	2440	nofs	nofs
chr02	E	2p16	3	49375000	2060	nofs	nofs
chr02	E	2p16	3	49625000	1780	nofs	nofs
chr02	E	2p16	3	49875000	1708	nofs	nofs
chr02	E	2p16	3	50125000	2593	nofs	nofs

chr02	E	2p16	3	50375000	2282	nofs	nofs
chr02	E	2p16	3	50625000	3331	nofs	nofs
chr02	E	2p16	3	50875000	2344	nofs	nofs
chr02	E	2p16	3	51125000	1461	nofs	nofs
chr02	E	2p16	3	51375000	3101	nofs	nofs
chr02	E	2p16	3	51625000	3822	nofs	nofs
chr02	E	2p16	3	51875000	2999	nofs	nofs
chr02	E	2p16	3	52125000	1531	nofs	nofs
chr02	E	2p16	3	52375000	1694	nofs	nofs
chr02	E	2p16	3	52625000	1835	nofs	nofs
chr02	E	2p16	3	52875000	1980	fs	fs common
chr02	E	2p16	3	53125000	3729	fs	fs common
chr02	E	2p16	3	53375000	2722	fs	fs common
chr02	E	2p16	3	53625000	2175	fs	fs common
chr02	E	2p16	3	53875000	2272	fs	fs common
chr02	E	2p16	3	54125000	5282	fs	fs common
chr02	E	2p16	3	54375000	2420	fs	fs common
chr02	E	2p16	3	54625000	2518	fs	fs common
chr02	E	2p16	3	54875000	2141	fs	fs common
chr02	E	2p16	3	55125000	1973	nofs	nofs
chr02	E	2p16	3	55375000	2728	nofs	nofs
chr02	E	2p16	3	55625000	2130	nofs	nofs
chr02	E	2p16	3	55875000	4244	nofs	nofs
chr02	E	2p16	3	56125000	1076	nofs	nofs
chr02	E	2p16	3	56375000	1619	nofs	nofs
chr02	E	2p16	3	56625000	1406	nofs	nofs
chr02	E	2p16	3	56875000	1979	nofs	nofs
chr02	E	2p16	3	57125000	2183	nofs	nofs
chr02	E	2p16	3	57375000	5140	nofs	nofs
chr02	E	2p16	3	57625000	2806	nofs	nofs
chr02	E	2p16	3	57875000	1871	nofs	nofs
chr02	E	2p16	3	58125000	1626	nofs	nofs
chr02	E	2p16	3	58375000	2523	nofs	nofs
chr02	E	2p16	3	58625000	2284	nofs	nofs
chr02	E	2p16	3	58875000	1296	nofs	nofs
chr02	E	2p16	3	59125000	1516	nofs	nofs

chr02	E	2p16	3	59375000	3033	nofs	nofs
chr02	E	2p16	3	59625000	2547	nofs	nofs
chr02	E	2p16	3	59875000	2477	nofs	nofs
chr02	E	2p16	3	60125000	2042	nofs	nofs
chr02	E	2p16	3	60375000	2161	nofs	nofs
chr02	E	2p16	3	60625000	4281	nofs	nofs
chr02	E	2p16	3	60875000	2793	nofs	nofs
chr02	E	2p16	3	61125000	2649	nofs	nofs
chr02	E	2p16	3	61375000	3986	nofs	nofs
chr02	E	2p15	2	61625000	5240	nofs	nofs
chr02	E	2p15	2	61875000	3788	nofs	nofs
chr02	E	2p15	2	62125000	2604	nofs	nofs
chr02	E	2p15	2	62375000	2789	nofs	nofs
chr02	E	2p15	2	62625000	1840	nofs	nofs
chr02	E	2p15	2	62875000	3290	nofs	nofs
chr02	E	2p15	2	63125000	2393	nofs	nofs
chr02	E	2p15	2	63375000	2398	nofs	nofs
chr02	E	2p15	2	63625000	2122	nofs	nofs
chr02	E	2p15	2	63875000	3742	nofs	nofs
chr02	E	2p14	1	64125000	2594	nofs	nofs
chr02	E	2p14	1	64375000	2460	nofs	nofs
chr02	E	2p14	1	64625000	3865	nofs	nofs
chr02	E	2p14	1	64875000	3076	nofs	nofs
chr02	E	2p14	1	65125000	2058	nofs	nofs
chr02	E	2p14	1	65375000	4261	nofs	nofs
chr02	E	2p14	1	65625000	3973	nofs	nofs
chr02	E	2p14	1	65875000	2126	nofs	nofs
chr02	E	2p14	1	66125000	2095	nofs	nofs
chr02	E	2p14	1	66375000	1785	nofs	nofs
chr02	E	2p14	1	66625000	1363	nofs	nofs
chr02	E	2p14	1	66875000	2818	nofs	nofs
chr02	E	2p14	1	67125000	1963	nofs	nofs
chr02	E	2p14	1	67375000	2981	nofs	nofs
chr02	E	2p14	1	67625000	1600	nofs	nofs
chr02	E	2p14	1	67875000	2951	nofs	nofs
chr02	E	2p14	1	68125000	2510	nofs	nofs

chr02	E	2p14	1	68375000	2732	nofs	nofs
chr02	E	2p13	5	68625000	7763	fs	fs common
chr02	E	2p13	5	68875000	1725	fs	fs common
chr02	E	2p13	5	69125000	2021	fs	fs common
chr02	E	2p13	5	69375000	1183	fs	fs common
chr02	E	2p13	5	69625000	3595	fs	fs common
chr02	E	2p13	5	69875000	2841	fs	fs common
chr02	E	2p13	5	70125000	4055	fs	fs common
chr02	E	2p13	5	70375000	3981	fs	fs common
chr02	E	2p13	5	70625000	4012	fs	fs common
chr02	E	2p13	5	70875000	2971	fs	fs common
chr02	E	2p13	5	71125000	12738	fs	fs common
chr02	E	2p13	5	71375000	3314	fs	fs common
chr02	E	2p13	5	71625000	3344	fs	fs common
chr02	E	2p13	5	71875000	5718	fs	fs common
chr02	E	2p13	5	72125000	2900	fs	fs common
chr02	E	2p13	5	72375000	1930	fs	fs common
chr02	E	2p13	5	72625000	1982	fs	fs common
chr02	E	2p13	5	72875000	2911	fs	fs common
chr02	E	2p13	5	73125000	1802	fs	fs common
chr02	E	2p13	5	73375000	3844	fs	fs common
chr02	E	2p13	5	73625000	5741	fs	fs common
chr02	E	2p13	5	73875000	2579	fs	fs common
chr02	E	2p13	5	74125000	3176	fs	fs common
chr02	E	2p13	5	74375000	4790	fs	fs common
chr02	E	2p13	5	74625000	3230	fs	fs common
chr02	E	2p13	5	74875000	2627	fs	fs common
chr02	E	2p13	5	75125000	2188	fs	fs common
chr02	E	2p12	2	75375000	1775	nofs	nofs
chr02	E	2p12	2	75625000	1584	nofs	nofs
chr02	E	2p12	2	75875000	3978	nofs	nofs
chr02	E	2p12	2	76125000	3053	nofs	nofs
chr02	E	2p12	2	76375000	1917	nofs	nofs
chr02	E	2p12	2	76625000	2033	nofs	nofs
chr02	E	2p12	2	76875000	1922	nofs	nofs
chr02	E	2p12	2	77125000	1967	nofs	nofs

chr02	E	2p12	2	77375000	3689	nofs	nofs
chr02	E	2p12	2	77625000	3074	nofs	nofs
chr02	E	2p12	2	77875000	3850	nofs	nofs
chr02	E	2p12	2	78125000	3009	nofs	nofs
chr02	E	2p12	2	78375000	2959	nofs	nofs
chr02	E	2p12	2	78625000	2735	nofs	nofs
chr02	E	2p12	2	78875000	2511	nofs	nofs
chr02	E	2p12	2	79125000	1477	nofs	nofs
chr02	E	2p12	2	79375000	2404	nofs	nofs
chr02	E	2p12	2	79625000	1524	nofs	nofs
chr02	E	2p12	2	79875000	1852	nofs	nofs
chr02	E	2p12	2	80125000	2908	nofs	nofs
chr02	E	2p12	2	80375000	2579	nofs	nofs
chr02	E	2p12	2	80625000	1718	nofs	nofs
chr02	E	2p12	2	80875000	2174	nofs	nofs
chr02	E	2p12	2	81125000	4200	nofs	nofs
chr02	E	2p12	2	81375000	1658	nofs	nofs
chr02	E	2p12	2	81625000	2721	nofs	nofs
chr02	E	2p12	2	81875000	1430	nofs	nofs
chr02	E	2p12	2	82125000	2343	nofs	nofs
chr02	E	2p12	2	82375000	4221	nofs	nofs
chr02	E	2p12	2	82625000	4198	nofs	nofs
chr02	E	2p12	2	82875000	2627	nofs	nofs
chr02	E	2p12	2	83125000	4550	nofs	nofs
chr02	E	2p11.2	1	83375000	5958	fs	fs rare
chr02	E	2p11.2	1	83625000	3327	fs	fs rare
chr02	E	2p11.2	1	83875000	2677	fs	fs rare
chr02	E	2p11.2	1	84125000	2641	fs	fs rare
chr02	E	2p11.2	1	84375000	2771	fs	fs rare
chr02	E	2p11.2	1	84625000	2088	fs	fs rare
chr02	E	2p11.2	1	84875000	2578	fs	fs rare
chr02	E	2p11.2	1	85125000	5224	fs	fs rare
chr02	E	2p11.2	1	85375000	3196	fs	fs rare
chr02	E	2p11.2	1	85625000	3710	fs	fs rare
chr02	E	2p11.2	1	85875000	3011	fs	fs rare
chr02	E	2p11.2	1	86125000	2416	fs	fs rare

chr02	E	2p11.2	1	86375000	1704	fs	fs rare
chr02	E	2p11.2	1	86625000	5115	fs	fs rare
chr02	E	2p11.2	1	86875000	2679	fs	fs rare
chr02	E	2p11.2	1	87125000	4004	fs	fs rare
chr02	E	2p11.2	1	87375000	6789	fs	fs rare
chr02	E	2p11.2	1	87625000	44012	fs	fs rare
chr02	E	2p11.2	1	87875000	2274	fs	fs rare
chr02	E	2p11.2	1	88125000	3221	fs	fs rare
chr02	E	2p11.2	1	88375000	3560	fs	fs rare
chr02	E	2p11.2	1	88625000	4160	fs	fs rare
chr02	C	2p11.2	1	88875000	1240	fs	fs rare
chr02	C	2p11.2	1	89125000	4704	fs	fs rare
chr02	C	2p11.2	1	89375000	3101	fs	fs rare
chr02	C	2p11.2	1	89625000	1771	fs	fs rare
chr02	C	2p11.2	1	89875000	8132	fs	fs rare
chr02	C	2p11.2	1	90125000	1566	fs	fs rare
chr02	C	2p11.1	.	90375000	0	nofs	nofs
chr02	C	2p11.1	.	90625000	0	nofs	nofs
chr02	C	2p11.1	.	90875000	0	nofs	nofs
chr02	C	2p11.1	.	90875000	0	nofs	nofs
chr02	C	2p11.1	.	91125000	41837	nofs	nofs
chr02	C	2p11.1	.	91375000	3807	nofs	nofs
chr02	C	2p11.1	.	91625000	65194	nofs	nofs
chr02	C		.	91875000	0	nofs	nofs
chr02	C		.	92125000	0	nofs	nofs
chr02	C		.	92375000	0	nofs	nofs
chr02	C		.	92625000	0	nofs	nofs
chr02	C		.	92875000	0	nofs	nofs
chr02	C		.	93125000	0	nofs	nofs
chr02	C		.	93375000	0	nofs	nofs
chr02	C		.	93625000	0	nofs	nofs
chr02	C		.	93875000	0	nofs	nofs
chr02	C		.	94125000	0	nofs	nofs
chr02	C		.	94375000	0	nofs	nofs
chr02	C		.	94625000	0	nofs	nofs
chr02	C	2q11.1	.	94875000	4643	nofs	nofs

chr02	C	2q11.1	.	95125000	7180	nofs	nofs
chr02	C	2q11.1	.	95375000	4818	nofs	nofs
chr02	C	2q11.1	.	95625000	4345	nofs	nofs
chr02	C	2q11.1	.	95875000	5664	fs	fs rare
chr02	C	2q11.1	.	96125000	2868	fs	fs rare
chr02	C	2q11.2	1	96375000	2775	fs	fs rare
chr02	C	2q11.2	1	96625000	4639	fs	fs rare
chr02	C	2q11.2	1	96875000	5322	fs	fs rare
chr02	C	2q11.2	1	97125000	3486	fs	fs rare
chr02	C	2q11.2	1	97375000	2952	fs	fs rare
chr02	C	2q11.2	1	97625000	7275	fs	fs rare
chr02	C	2q11.2	1	97875000	2482	fs	fs rare
chr02	E	2q11.2	1	98125000	4293	fs	fs rare
chr02	E	2q11.2	1	98375000	2292	fs	fs rare
chr02	E	2q11.2	1	98625000	2512	fs	fs rare
chr02	E	2q11.2	1	98875000	2861	fs	fs rare
chr02	E	2q11.2	1	99125000	4042	fs	fs rare
chr02	E	2q11.2	1	99375000	2911	fs	fs rare
chr02	E	2q11.2	1	99625000	2420	fs	fs rare
chr02	E	2q11.2	1	99875000	1457	fs	fs rare
chr02	E	2q11.2	1	100000000	3140	fs	fs rare
chr02	E	2q11.2	1	100000000	1509	fs	fs rare
chr02	E	2q11.2	1	101000000	3052	fs	fs rare
chr02	E	2q11.2	1	101000000	1436	fs	fs rare
chr02	E	2q11.2	1	101000000	3033	fs	fs rare
chr02	E	2q11.2	1	101000000	3058	fs	fs rare
chr02	E	2q11.2	1	102000000	4963	fs	fs rare
chr02	E	2q11.2	1	102000000	2199	fs	fs rare
chr02	E	2q11.2	1	102000000	4897	fs	fs rare
chr02	E	2q11.2	1	102000000	3624	fs	fs rare
chr02	E	2q11.2	1	103000000	2150	fs	fs rare
chr02	E	2q12	1	103000000	2383	nofs	nofs
chr02	E	2q12	1	103000000	2245	nofs	nofs
chr02	E	2q12	1	103000000	3044	nofs	nofs
chr02	E	2q12	1	104000000	3750	nofs	nofs
chr02	E	2q12	1	104000000	2398	nofs	nofs

chr02	E	2q12	1	104000000	3120	nofs	nofs
chr02	E	2q12	1	104000000	2380	nofs	nofs
chr02	E	2q12	1	105000000	2269	nofs	nofs
chr02	E	2q12	1	105000000	2896	nofs	nofs
chr02	E	2q12	1	105000000	3805	nofs	nofs
chr02	E	2q12	1	105000000	4445	nofs	nofs
chr02	E	2q12	1	106000000	2620	nofs	nofs
chr02	E	2q12	1	106000000	2370	nofs	nofs
chr02	E	2q12	1	106000000	3783	nofs	nofs
chr02	E	2q12	1	106000000	3290	nofs	nofs
chr02	E	2q12	1	107000000	5039	nofs	nofs
chr02	E	2q12	1	107000000	1924	nofs	nofs
chr02	E	2q12	1	107000000	2207	nofs	nofs
chr02	E	2q12	1	107000000	2431	nofs	nofs
chr02	E	2q12	1	108000000	4218	nofs	nofs
chr02	E	2q12	1	108000000	6087	nofs	nofs
chr02	E	2q12	1	108000000	2381	nofs	nofs
chr02	E	2q12	1	108000000	3402	nofs	nofs
chr02	E	2q12	1	109000000	2995	nofs	nofs
chr02	E	2q13	6	109000000	4611	fs	fs rare
chr02	E	2q13	6	109000000	3136	fs	fs rare
chr02	E	2q13	6	109000000	3388	fs	fs rare
chr02	E	2q13	6	110000000	2431	fs	fs rare
chr02	E	2q13	6	110000000	1694	fs	fs rare
chr02	E	2q13	6	110000000	3879	fs	fs rare
chr02	E	2q13	6	110000000	1850	fs	fs rare
chr02	E	2q13	6	111000000	3505	fs	fs rare
chr02	E	2q13	6	111000000	1024	fs	fs rare
chr02	E	2q13	6	111000000	2595	fs	fs rare
chr02	E	2q13	6	111000000	2834	fs	fs rare
chr02	E	2q13	6	112000000	4060	fs	fs rare
chr02	E	2q13	6	112000000	2029	fs	fs rare
chr02	E	2q13	6	112000000	4503	fs	fs rare
chr02	E	2q13	6	112000000	1949	fs	fs rare
chr02	E	2q13	6	113000000	7434	fs	fs rare
chr02	E	2q13	6	113000000	3634	fs	fs rare

chr02	E	2q13	6	113000000	3318	fs	fs rare
chr02	E	2q13	6	113000000	3332	fs	fs rare
chr02	E	2q13	6	114000000	5070	fs	fs rare
chr02	E	2q13	6	114000000	5104	fs	fs rare
chr02	E	2q14.1	6	114000000	8497	nofs	nofs
chr02	E	2q14.1	6	114000000	3701	nofs	nofs
chr02	E	2q14.1	6	115000000	3273	nofs	nofs
chr02	E	2q14.1	6	115000000	5563	nofs	nofs
chr02	E	2q14.1	6	115000000	2652	nofs	nofs
chr02	E	2q14.1	6	115000000	3158	nofs	nofs
chr02	E	2q14.1	6	116000000	2295	nofs	nofs
chr02	E	2q14.1	6	116000000	3715	nofs	nofs
chr02	E	2q14.1	6	116000000	2147	nofs	nofs
chr02	E	2q14.1	6	116000000	2238	nofs	nofs
chr02	E	2q14.1	6	117000000	1936	nofs	nofs
chr02	E	2q14.1	6	117000000	2150	nofs	nofs
chr02	E	2q14.1	6	117000000	2225	nofs	nofs
chr02	E	2q14.1	6	117000000	5109	nofs	nofs
chr02	E	2q14.1	6	118000000	8119	nofs	nofs
chr02	E	2q14.1	6	118000000	3197	nofs	nofs
chr02	E	2q14.1	6	118000000	2339	nofs	nofs
chr02	E	2q14.1	6	118000000	2412	nofs	nofs
chr02	E	2q14.1	6	119000000	3612	nofs	nofs
chr02	E	2q14.2	2	119000000	3902	nofs	nofs
chr02	E	2q14.2	2	119000000	2610	nofs	nofs
chr02	E	2q14.2	2	119000000	3338	nofs	nofs
chr02	E	2q14.2	2	120000000	4112	nofs	nofs
chr02	E	2q14.2	2	120000000	3262	nofs	nofs
chr02	E	2q14.2	2	120000000	3338	nofs	nofs
chr02	E	2q14.2	2	120000000	2991	nofs	nofs
chr02	E	2q14.2	2	121000000	3232	nofs	nofs
chr02	E	2q14.2	2	121000000	3788	nofs	nofs
chr02	E	2q14.2	2	121000000	2529	nofs	nofs
chr02	E	2q14.2	2	121000000	5233	nofs	nofs
chr02	E	2q14.2	2	122000000	4444	nofs	nofs
chr02	E	2q14.2	2	122000000	2810	nofs	nofs

chr02	E	2q14.2	2	122000000	6152	nofs	nofs
chr02	E	2q14.3	5	122000000	3156	nofs	nofs
chr02	E	2q14.3	5	123000000	5296	nofs	nofs
chr02	E	2q14.3	5	123000000	2142	nofs	nofs
chr02	E	2q14.3	5	123000000	2907	nofs	nofs
chr02	E	2q14.3	5	123000000	2088	nofs	nofs
chr02	E	2q14.3	5	124000000	2506	nofs	nofs
chr02	E	2q14.3	5	124000000	3588	nofs	nofs
chr02	E	2q14.3	5	124000000	3627	nofs	nofs
chr02	E	2q14.3	5	124000000	2389	nofs	nofs
chr02	E	2q14.3	5	125000000	1923	nofs	nofs
chr02	E	2q14.3	5	125000000	3468	nofs	nofs
chr02	E	2q14.3	5	125000000	5613	nofs	nofs
chr02	E	2q14.3	5	125000000	3129	nofs	nofs
chr02	E	2q14.3	5	126000000	3167	nofs	nofs
chr02	E	2q14.3	5	126000000	2790	nofs	nofs
chr02	E	2q14.3	5	126000000	3245	nofs	nofs
chr02	E	2q14.3	5	126000000	6128	nofs	nofs
chr02	E	2q14.3	5	127000000	2055	nofs	nofs
chr02	E	2q14.3	5	127000000	1434	nofs	nofs
chr02	E	2q14.3	5	127000000	2898	nofs	nofs
chr02	E	2q14.3	5	127000000	2795	nofs	nofs
chr02	E	2q14.3	5	128000000	5054	nofs	nofs
chr02	E	2q14.3	5	128000000	2189	nofs	nofs
chr02	E	2q14.3	5	128000000	4728	nofs	nofs
chr02	E	2q14.3	5	128000000	5566	nofs	nofs
chr02	E	2q14.3	5	129000000	5926	nofs	nofs
chr02	E	2q14.3	5	129000000	5718	nofs	nofs
chr02	E	2q14.3	5	129000000	5651	nofs	nofs
chr02	E	2q14.3	5	129000000	5302	nofs	nofs
chr02	E	2q14.3	5	130000000	3383	nofs	nofs
chr02	E	2q21.1	6	130000000	1392	nofs	nofs
chr02	E	2q21.1	6	130000000	4854	nofs	nofs
chr02	E	2q21.1	6	130000000	4685	nofs	nofs
chr02	E	2q21.1	6	131000000	3496	nofs	nofs
chr02	E	2q21.1	6	131000000	3083	nofs	nofs

chr02	E	2q21.1	6	131000000	1488	nofs	nofs
chr02	E	2q21.1	6	131000000	4173	nofs	nofs
chr02	E	2q21.1	6	132000000	2752	nofs	nofs
chr02	E	2q21.1	6	132000000	2635	nofs	nofs
chr02	E	2q21.1	6	132000000	2760	nofs	nofs
chr02	E	2q21.1	6	132000000	8677	nofs	nofs
chr02	E	2q21.2	5	133000000	16328	nofs	nofs
chr02	E	2q21.2	5	133000000	42957	nofs	nofs
chr02	E	2q21.2	5	133000000	2944	nofs	nofs
chr02	E	2q21.2	5	133000000	1460	nofs	nofs
chr02	E	2q21.2	5	134000000	2226	nofs	nofs
chr02	E	2q21.2	5	134000000	1404	nofs	nofs
chr02	E	2q21.2	5	134000000	2441	nofs	nofs
chr02	E	2q21.2	5	134000000	2793	nofs	nofs
chr02	E	2q21.2	5	135000000	5160	nofs	nofs
chr02	E	2q21.3	1	135000000	2584	fs	fs common
chr02	E	2q21.3	1	135000000	4859	fs	fs common
chr02	E	2q21.3	1	135000000	3674	fs	fs common
chr02	E	2q21.3	1	136000000	4404	fs	fs common
chr02	E	2q21.3	1	136000000	2380	fs	fs common
chr02	E	2q21.3	1	136000000	2696	fs	fs common
chr02	E	2q21.3	1	136000000	2415	fs	fs common
chr02	E	2q21.3	1	137000000	2143	fs	fs common
chr02	E	2q21.3	1	137000000	4486	fs	fs common
chr02	E	2q21.3	1	137000000	2336	fs	fs common
chr02	E	2q21.3	1	137000000	3159	fs	fs common
chr02	E	2q22	2	138000000	1872	nofs	nofs
chr02	E	2q22	2	138000000	2202	nofs	nofs
chr02	E	2q22	2	138000000	2424	nofs	nofs
chr02	E	2q22	2	138000000	2739	nofs	nofs
chr02	E	2q22	2	139000000	2907	nofs	nofs
chr02	E	2q22	2	139000000	2300	nofs	nofs
chr02	E	2q22	2	139000000	2171	nofs	nofs
chr02	E	2q22	2	139000000	1967	nofs	nofs
chr02	E	2q22	2	140000000	1874	nofs	nofs
chr02	E	2q22	2	140000000	2878	nofs	nofs

chr02	E	2q22	2	140000000	1887	nofs	nofs
chr02	E	2q22	2	140000000	2938	nofs	nofs
chr02	E	2q22	2	141000000	3290	nofs	nofs
chr02	E	2q22	2	141000000	3390	nofs	nofs
chr02	E	2q22	2	141000000	2267	nofs	nofs
chr02	E	2q22	2	141000000	3409	nofs	nofs
chr02	E	2q22	2	142000000	2179	nofs	nofs
chr02	E	2q22	2	142000000	2707	nofs	nofs
chr02	E	2q22	2	142000000	3576	nofs	nofs
chr02	E	2q22	2	142000000	2067	nofs	nofs
chr02	E	2q22	2	143000000	2322	nofs	nofs
chr02	E	2q22	2	143000000	2048	nofs	nofs
chr02	E	2q22	2	143000000	2533	nofs	nofs
chr02	E	2q22	2	143000000	2553	nofs	nofs
chr02	E	2q22	2	144000000	2146	nofs	nofs
chr02	E	2q22	2	144000000	1830	nofs	nofs
chr02	E	2q22	2	144000000	2017	fs	fs rare
chr02	E	2q22	2	144000000	2511	fs	fs rare
chr02	E	2q22	2	145000000	3282	fs	fs rare
chr02	E	2q22	2	145000000	2832	fs	fs rare
chr02	E	2q22	2	145000000	2344	fs	fs rare
chr02	E	2q22	2	145000000	1538	fs	fs rare
chr02	E	2q22	2	146000000	2544	fs	fs rare
chr02	E	2q22	2	146000000	1526	fs	fs rare
chr02	E	2q22	2	146000000	2519	fs	fs rare
chr02	E	2q22	2	146000000	3460	fs	fs rare
chr02	E	2q22	2	147000000	2721	fs	fs rare
chr02	E	2q22	2	147000000	2802	fs	fs rare
chr02	E	2q22	2	147000000	1990	fs	fs rare
chr02	E	2q22	2	147000000	1983	fs	fs rare
chr02	E	2q22	2	148000000	2835	fs	fs rare
chr02	E	2q22	2	148000000	1666	fs	fs rare
chr02	E	2q22	2	148000000	2303	fs	fs rare
chr02	E	2q22	2	148000000	2948	fs	fs rare
chr02	E	2q22	2	149000000	2902	fs	fs rare
chr02	E	2q23	2	149000000	2231	nofs	nofs

chr02	E	2q23	2	149000000	2042	nofs	nofs
chr02	E	2q23	2	149000000	3148	nofs	nofs
chr02	E	2q23	2	150000000	1002	nofs	nofs
chr02	E	2q23	2	150000000	1921	nofs	nofs
chr02	E	2q23	2	150000000	1377	nofs	nofs
chr02	E	2q23	2	150000000	1966	nofs	nofs
chr02	E	2q23	2	151000000	3838	nofs	nofs
chr02	E	2q23	2	151000000	2515	nofs	nofs
chr02	E	2q23	2	151000000	2300	nofs	nofs
chr02	E	2q23	2	151000000	2141	nofs	nofs
chr02	E	2q23	2	152000000	2604	nofs	nofs
chr02	E	2q23	2	152000000	2851	nofs	nofs
chr02	E	2q23	2	152000000	2318	nofs	nofs
chr02	E	2q23	2	152000000	1598	nofs	nofs
chr02	E	2q23	2	153000000	2264	nofs	nofs
chr02	E	2q23	2	153000000	3633	nofs	nofs
chr02	E	2q23	2	153000000	2262	nofs	nofs
chr02	E	2q23	2	153000000	2025	nofs	nofs
chr02	E	2q23	2	154000000	3157	nofs	nofs
chr02	E	2q23	2	154000000	1344	nofs	nofs
chr02	E	2q23	2	154000000	1814	nofs	nofs
chr02	E	2q23	2	154000000	1552	nofs	nofs
chr02	E	2q23	2	155000000	2024	nofs	nofs
chr02	B	2q24.1	.	155000000	3360	nofs	nofs
chr02	B	2q24.1	.	155000000	1643	nofs	nofs
chr02	B	2q24.1	.	155000000	2305	nofs	nofs
chr02	B	2q24.1	.	156000000	3424	nofs	nofs
chr02	B	2q24.1	.	156000000	2066	nofs	nofs
chr02	B	2q24.1	.	156000000	2625	nofs	nofs
chr02	B	2q24.1	.	156000000	1634	nofs	nofs
chr02	B	2q24.1	.	157000000	1807	nofs	nofs
chr02	B	2q24.1	.	157000000	2211	nofs	nofs
chr02	B	2q24.1	.	157000000	4271	nofs	nofs
chr02	B	2q24.1	.	157000000	1734	nofs	nofs
chr02	B	2q24.1	.	158000000	1791	nofs	nofs
chr02	B	2q24.1	.	158000000	1606	nofs	nofs

chr02	B	2q24.1	.	158000000	2413	nofs	nofs
chr02	B	2q24.1	.	158000000	1822	nofs	nofs
chr02	B	2q24.1	.	159000000	2647	nofs	nofs
chr02	B	2q24.1	.	159000000	2204	nofs	nofs
chr02	B	2q24.1	.	159000000	732	nofs	nofs
chr02	B	2q24.1	.	159000000	1797	nofs	nofs
chr02	E	2q24.2	1	160000000	8013	nofs	nofs
chr02	E	2q24.2	1	160000000	1613	nofs	nofs
chr02	E	2q24.2	1	160000000	1561	nofs	nofs
chr02	E	2q24.2	1	160000000	2775	nofs	nofs
chr02	E	2q24.2	1	161000000	3131	nofs	nofs
chr02	E	2q24.2	1	161000000	1704	nofs	nofs
chr02	E	2q24.2	1	161000000	3407	nofs	nofs
chr02	E	2q24.2	1	161000000	1834	nofs	nofs
chr02	E	2q24.2	1	162000000	1455	nofs	nofs
chr02	E	2q24.2	1	162000000	1085	nofs	nofs
chr02	E	2q24.2	1	162000000	1281	nofs	nofs
chr02	E	2q24.2	1	162000000	3151	nofs	nofs
chr02	E	2q24.2	1	163000000	2386	nofs	nofs
chr02	E	2q24.2	1	163000000	1879	nofs	nofs
chr02	E	2q24.2	1	163000000	2443	nofs	nofs
chr02	E	2q24.2	1	163000000	1847	nofs	nofs
chr02	E	2q24.2	1	164000000	2440	nofs	nofs
chr02	B	2q24.3	.	164000000	2023	nofs	nofs
chr02	B	2q24.3	.	164000000	2292	nofs	nofs
chr02	B	2q24.3	.	164000000	2215	nofs	nofs
chr02	B	2q24.3	.	165000000	1927	nofs	nofs
chr02	B	2q24.3	.	165000000	2832	nofs	nofs
chr02	B	2q24.3	.	165000000	2200	nofs	nofs
chr02	B	2q24.3	.	165000000	1792	nofs	nofs
chr02	B	2q24.3	.	166000000	2332	nofs	nofs
chr02	B	2q24.3	.	166000000	2876	nofs	nofs
chr02	B	2q24.3	.	166000000	1998	nofs	nofs
chr02	B	2q24.3	.	166000000	3570	nofs	nofs
chr02	B	2q24.3	.	167000000	2312	nofs	nofs
chr02	B	2q24.3	.	167000000	3815	nofs	nofs

chr02	B	2q24.3	.	167000000	2553	nofs	nofs
chr02	B	2q24.3	.	167000000	6406	nofs	nofs
chr02	B	2q24.3	.	168000000	3186	nofs	nofs
chr02	B	2q24.3	.	168000000	2013	nofs	nofs
chr02	B	2q24.3	.	168000000	2889	nofs	nofs
chr02	B	2q24.3	.	168000000	1718	nofs	nofs
chr02	B	2q24.3	.	169000000	2325	nofs	nofs
chr02	B	2q24.3	.	169000000	1735	nofs	nofs
chr02	B	2q24.3	.	169000000	2143	nofs	nofs
chr02	B	2q24.3	.	169000000	2660	nofs	nofs
chr02	B	2q31	.	170000000	2677	fs	fs common
chr02	B	2q31	.	170000000	2005	fs	fs common
chr02	B	2q31	.	170000000	8680	fs	fs common
chr02	B	2q31	.	170000000	4308	fs	fs common
chr02	B	2q31	.	171000000	4356	fs	fs common
chr02	B	2q31	.	171000000	2800	fs	fs common
chr02	B	2q31	.	171000000	2449	fs	fs common
chr02	B	2q31	.	171000000	3330	fs	fs common
chr02	B	2q31	.	172000000	4042	fs	fs common
chr02	B	2q31	.	172000000	2432	fs	fs common
chr02	B	2q31	.	172000000	3511	fs	fs common
chr02	B	2q31	.	172000000	2621	fs	fs common
chr02	B	2q31	.	173000000	5615	fs	fs common
chr02	B	2q31	.	173000000	1809	fs	fs common
chr02	B	2q31	.	173000000	3492	fs	fs common
chr02	B	2q31	.	173000000	4646	fs	fs common
chr02	B	2q31	.	174000000	1736	fs	fs common
chr02	B	2q31	.	174000000	2286	fs	fs common
chr02	B	2q31	.	174000000	2436	fs	fs common
chr02	B	2q31	.	174000000	4446	fs	fs common
chr02	B	2q31	.	175000000	1755	fs	fs common
chr02	B	2q31	.	175000000	2641	fs	fs common
chr02	B	2q31	.	175000000	3140	fs	fs common
chr02	B	2q31	.	175000000	4575	fs	fs common
chr02	B	2q31	.	176000000	1906	fs	fs common
chr02	B	2q31	.	176000000	2872	fs	fs common

chr02	B	2q31	.	176000000	2035	fs	fs common
chr02	B	2q31	.	176000000	1931	fs	fs common
chr02	B	2q31	.	177000000	1283	fs	fs common
chr02	B	2q31	.	177000000	1650	fs	fs common
chr02	B	2q31	.	177000000	2100	fs	fs common
chr02	B	2q31	.	177000000	1529	fs	fs common
chr02	B	2q31	.	178000000	1565	fs	fs common
chr02	B	2q31	.	178000000	3223	fs	fs common
chr02	B	2q31	.	178000000	3458	fs	fs common
chr02	B	2q31	.	178000000	1815	fs	fs common
chr02	B	2q31	.	179000000	5371	fs	fs common
chr02	B	2q31	.	179000000	2148	fs	fs common
chr02	B	2q31	.	179000000	1843	fs	fs common
chr02	B	2q31	.	179000000	4805	fs	fs common
chr02	B	2q31	.	180000000	2483	fs	fs common
chr02	B	2q31	.	180000000	1992	fs	fs common
chr02	B	2q31	.	180000000	4021	fs	fs common
chr02	B	2q31	.	180000000	1545	fs	fs common
chr02	B	2q31	.	181000000	4355	fs	fs common
chr02	B	2q31	.	181000000	1178	fs	fs common
chr02	B	2q31	.	181000000	1877	fs	fs common
chr02	B	2q31	.	181000000	3057	fs	fs common
chr02	B	2q31	.	182000000	1239	fs	fs common
chr02	B	2q31	.	182000000	2691	fs	fs common
chr02	B	2q31	.	182000000	1467	fs	fs common
chr02	B	2q31	.	182000000	1578	fs	fs common
chr02	B	2q31	.	183000000	1735	fs	fs common
chr02	B	2q31	.	183000000	1819	fs	fs common
chr02	E	2q32.1	2	183000000	2646	fs	fs common
chr02	E	2q32.1	2	183000000	3536	fs	fs common
chr02	E	2q32.1	2	184000000	1674	fs	fs common
chr02	E	2q32.1	2	184000000	2468	fs	fs common
chr02	E	2q32.1	2	184000000	2865	fs	fs common
chr02	E	2q32.1	2	184000000	2505	fs	fs common
chr02	E	2q32.1	2	185000000	1568	fs	fs common
chr02	E	2q32.1	2	185000000	2245	fs	fs common

chr02	E	2q32.1	2	185000000	2408	fs	fs common
chr02	E	2q32.1	2	185000000	1864	fs	fs common
chr02	E	2q32.1	2	186000000	2214	fs	fs common
chr02	E	2q32.1	2	186000000	2943	fs	fs common
chr02	E	2q32.1	2	186000000	2726	fs	fs common
chr02	E	2q32.1	2	186000000	1430	fs	fs common
chr02	E	2q32.1	2	187000000	3389	fs	fs common
chr02	E	2q32.1	2	187000000	3053	fs	fs common
chr02	E	2q32.1	2	187000000	2487	fs	fs common
chr02	E	2q32.1	2	187000000	2253	fs	fs common
chr02	E	2q32.1	2	188000000	2296	fs	fs common
chr02	E	2q32.1	2	188000000	2288	fs	fs common
chr02	E	2q32.1	2	188000000	3160	fs	fs common
chr02	E	2q32.1	2	188000000	3020	fs	fs common
chr02	E	2q32.1	2	189000000	6163	fs	fs common
chr02	E	2q32.1	2	189000000	2148	fs	fs common
chr02	E	2q32.1	2	189000000	1479	fs	fs common
chr02	E	2q32.2	3	189000000	2301	nofs	nofs
chr02	E	2q32.2	3	190000000	2531	nofs	nofs
chr02	E	2q32.2	3	190000000	985	nofs	nofs
chr02	E	2q32.2	3	190000000	2304	nofs	nofs
chr02	E	2q32.2	3	190000000	3033	nofs	nofs
chr02	E	2q32.2	3	191000000	2240	nofs	nofs
chr02	E	2q32.2	3	191000000	1854	nofs	nofs
chr02	E	2q32.2	3	191000000	3831	nofs	nofs
chr02	E	2q32.2	3	191000000	2098	nofs	nofs
chr02	E	2q32.2	3	192000000	2399	nofs	nofs
chr02	E	2q32.2	3	192000000	1847	nofs	nofs
chr02	E	2q32.3	1	192000000	3438	nofs	nofs
chr02	E	2q32.3	1	192000000	4139	nofs	nofs
chr02	E	2q32.3	1	193000000	2302	nofs	nofs
chr02	E	2q32.3	1	193000000	2330	nofs	nofs
chr02	E	2q32.3	1	193000000	2914	nofs	nofs
chr02	E	2q32.3	1	193000000	4791	nofs	nofs
chr02	E	2q32.3	1	194000000	3022	nofs	nofs
chr02	E	2q32.3	1	194000000	2041	nofs	nofs

chr02	E	2q32.3	1	194000000	3169	nofs	nofs
chr02	E	2q32.3	1	194000000	2695	nofs	nofs
chr02	E	2q32.3	1	195000000	4941	nofs	nofs
chr02	E	2q32.3	1	195000000	2641	nofs	nofs
chr02	E	2q32.3	1	195000000	4597	nofs	nofs
chr02	E	2q32.3	1	195000000	3828	nofs	nofs
chr02	E	2q32.3	1	196000000	1492	nofs	nofs
chr02	E	2q32.3	1	196000000	1353	nofs	nofs
chr02	E	2q32.3	1	196000000	2689	nofs	nofs
chr02	E	2q32.3	1	196000000	2893	nofs	nofs
chr02	E	2q32.3	1	197000000	2186	nofs	nofs
chr02	E	2q32.3	1	197000000	1754	nofs	nofs
chr02	E	2q32.3	1	197000000	1319	nofs	nofs
chr02	E	2q32.3	1	197000000	2316	nofs	nofs
chr02	E	2q33	1	198000000	2322	fs	fs common
chr02	E	2q33	1	198000000	1947	fs	fs common
chr02	E	2q33	1	198000000	3106	fs	fs common
chr02	E	2q33	1	198000000	3916	fs	fs common
chr02	E	2q33	1	199000000	3453	fs	fs common
chr02	E	2q33	1	199000000	2123	fs	fs common
chr02	E	2q33	1	199000000	2791	fs	fs common
chr02	E	2q33	1	199000000	1520	fs	fs common
chr02	E	2q33	1	200000000	3941	fs	fs common
chr02	E	2q33	1	200000000	1929	fs	fs common
chr02	E	2q33	1	200000000	1942	fs	fs common
chr02	E	2q33	1	200000000	1906	fs	fs common
chr02	E	2q33	1	201000000	1599	fs	fs common
chr02	E	2q33	1	201000000	2836	fs	fs common
chr02	E	2q33	1	201000000	1518	fs	fs common
chr02	E	2q33	1	201000000	3538	fs	fs common
chr02	E	2q33	1	202000000	2644	fs	fs common
chr02	E	2q33	1	202000000	4359	fs	fs common
chr02	E	2q33	1	202000000	2492	fs	fs common
chr02	E	2q33	1	202000000	2789	fs	fs common
chr02	E	2q33	1	203000000	3119	fs	fs common
chr02	E	2q33	1	203000000	4266	fs	fs common

chr02	E	2q33	1	203000000	6079	fs	fs common
chr02	E	2q33	1	203000000	5034	fs	fs common
chr02	E	2q33	1	204000000	2870	fs	fs common
chr02	E	2q33	1	204000000	5423	fs	fs common
chr02	E	2q33	1	204000000	4039	fs	fs common
chr02	E	2q33	1	204000000	3198	fs	fs common
chr02	E	2q33	1	205000000	2393	fs	fs common
chr02	E	2q33	1	205000000	1746	fs	fs common
chr02	E	2q33	1	205000000	2224	fs	fs common
chr02	E	2q33	1	205000000	2597	fs	fs common
chr02	E	2q33	1	206000000	3894	fs	fs common
chr02	E	2q33	1	206000000	2332	fs	fs common
chr02	E	2q33	1	206000000	3055	fs	fs common
chr02	E	2q33	1	206000000	1379	fs	fs common
chr02	E	2q33	1	207000000	2818	fs	fs common
chr02	E	2q33	1	207000000	5727	fs	fs common
chr02	E	2q33	1	207000000	5521	fs	fs common
chr02	E	2q33	1	207000000	1136	fs	fs common
chr02	E	2q33	1	208000000	1750	fs	fs common
chr02	E	2q33	1	208000000	1305	fs	fs common
chr02	E	2q33	1	208000000	2178	fs	fs common
chr02	E	2q33	1	208000000	4870	fs	fs common
chr02	E	2q33	1	209000000	3723	fs	fs common
chr02	E	2q33	1	209000000	3031	fs	fs common
chr02	E	2q34	1	209000000	2253	nofs	nofs
chr02	E	2q34	1	209000000	2254	nofs	nofs
chr02	E	2q34	1	210000000	1314	nofs	nofs
chr02	E	2q34	1	210000000	1913	nofs	nofs
chr02	E	2q34	1	210000000	2499	nofs	nofs
chr02	E	2q34	1	210000000	1968	nofs	nofs
chr02	E	2q34	1	211000000	2501	nofs	nofs
chr02	E	2q34	1	211000000	1945	nofs	nofs
chr02	E	2q34	1	211000000	2026	nofs	nofs
chr02	E	2q34	1	211000000	1596	nofs	nofs
chr02	E	2q34	1	212000000	3009	nofs	nofs
chr02	E	2q34	1	212000000	3141	nofs	nofs

chr02	E	2q34	1	212000000	2547	nofs	nofs
chr02	E	2q34	1	212000000	2473	nofs	nofs
chr02	E	2q34	1	213000000	2679	nofs	nofs
chr02	E	2q34	1	213000000	2148	nofs	nofs
chr02	E	2q34	1	213000000	2263	nofs	nofs
chr02	E	2q34	1	213000000	2525	nofs	nofs
chr02	E	2q34	1	214000000	1497	nofs	nofs
chr02	E	2q34	1	214000000	1633	nofs	nofs
chr02	E	2q34	1	214000000	2470	nofs	nofs
chr02	E	2q34	1	214000000	3228	nofs	nofs
chr02	E	2q34	1	215000000	3117	nofs	nofs
chr02	E	2q34	1	215000000	2282	nofs	nofs
chr02	E	2q34	1	215000000	1695	nofs	nofs
chr02	E	2q35	1	215000000	2177	nofs	nofs
chr02	E	2q35	1	216000000	2637	nofs	nofs
chr02	E	2q35	1	216000000	4752	nofs	nofs
chr02	E	2q35	1	216000000	2553	nofs	nofs
chr02	E	2q35	1	216000000	3247	nofs	nofs
chr02	E	2q35	1	217000000	4276	nofs	nofs
chr02	E	2q35	1	217000000	4216	nofs	nofs
chr02	E	2q35	1	217000000	2354	nofs	nofs
chr02	E	2q35	1	217000000	4205	nofs	nofs
chr02	E	2q35	1	218000000	2013	nofs	nofs
chr02	E	2q35	1	218000000	3669	nofs	nofs
chr02	E	2q35	1	218000000	2560	nofs	nofs
chr02	E	2q35	1	218000000	4749	nofs	nofs
chr02	E	2q35	1	219000000	4012	nofs	nofs
chr02	E	2q35	1	219000000	3254	nofs	nofs
chr02	E	2q35	1	219000000	1787	nofs	nofs
chr02	E	2q35	1	219000000	3234	nofs	nofs
chr02	E	2q35	1	220000000	5082	nofs	nofs
chr02	E	2q35	1	220000000	2191	nofs	nofs
chr02	E	2q35	1	220000000	2574	nofs	nofs
chr02	E	2q35	1	220000000	2753	nofs	nofs
chr02	E	2q35	1	221000000	1846	nofs	nofs
chr02	E	2q35	1	221000000	2875	nofs	nofs

chr02	E	2q35	1	221000000	2711	nofs	nofs
chr02	E	2q35	1	221000000	2598	nofs	nofs
chr02	E	2q36	3	222000000	3070	nofs	nofs
chr02	E	2q36	3	222000000	1760	nofs	nofs
chr02	E	2q36	3	222000000	6485	nofs	nofs
chr02	E	2q36	3	222000000	3110	nofs	nofs
chr02	E	2q36	3	223000000	2660	nofs	nofs
chr02	E	2q36	3	223000000	2944	nofs	nofs
chr02	E	2q36	3	223000000	3793	nofs	nofs
chr02	E	2q36	3	223000000	2729	nofs	nofs
chr02	E	2q36	3	224000000	5111	nofs	nofs
chr02	E	2q36	3	224000000	2785	nofs	nofs
chr02	E	2q36	3	224000000	1964	nofs	nofs
chr02	E	2q36	3	224000000	3010	nofs	nofs
chr02	E	2q36	3	225000000	3185	nofs	nofs
chr02	E	2q36	3	225000000	2463	nofs	nofs
chr02	E	2q36	3	225000000	1989	nofs	nofs
chr02	E	2q36	3	225000000	4761	nofs	nofs
chr02	E	2q36	3	226000000	4124	nofs	nofs
chr02	E	2q36	3	226000000	3711	nofs	nofs
chr02	E	2q36	3	226000000	2683	nofs	nofs
chr02	E	2q36	3	226000000	4138	nofs	nofs
chr02	E	2q36	3	227000000	2964	nofs	nofs
chr02	E	2q36	3	227000000	2820	nofs	nofs
chr02	E	2q36	3	227000000	5062	nofs	nofs
chr02	E	2q36	3	227000000	3033	nofs	nofs
chr02	E	2q36	3	228000000	1951	nofs	nofs
chr02	E	2q36	3	228000000	3103	nofs	nofs
chr02	E	2q36	3	228000000	3101	nofs	nofs
chr02	E	2q36	3	228000000	4099	nofs	nofs
chr02	E	2q36	3	229000000	2897	nofs	nofs
chr02	E	2q36	3	229000000	4990	nofs	nofs
chr02	E	2q36	3	229000000	3255	nofs	nofs
chr02	E	2q36	3	229000000	2880	nofs	nofs
chr02	E	2q36	3	230000000	2111	nofs	nofs
chr02	E	2q36	3	230000000	3224	nofs	nofs

chr02	E	2q36	3	230000000	3109	nofs	nofs
chr02	E	2q36	3	230000000	5749	nofs	nofs
chr02	E	2q36	3	231000000	2546	nofs	nofs
chr02	E	2q36	3	231000000	3464	nofs	nofs
chr02	E	2q37.1	2	231000000	2234	nofs	nofs
chr02	E	2q37.1	2	231000000	2924	nofs	nofs
chr02	E	2q37.1	2	232000000	4574	nofs	nofs
chr02	E	2q37.1	2	232000000	3298	nofs	nofs
chr02	E	2q37.1	2	232000000	5101	nofs	nofs
chr02	E	2q37.1	2	232000000	4655	nofs	nofs
chr02	E	2q37.1	2	233000000	30263	nofs	nofs
chr02	E	2q37.1	2	233000000	2232	nofs	nofs
chr02	E	2q37.1	2	233000000	2855	nofs	nofs
chr02	E	2q37.1	2	233000000	3669	nofs	nofs
chr02	E	2q37.1	2	234000000	9237	nofs	nofs
chr02	E	2q37.1	2	234000000	5154	nofs	nofs
chr02	E	2q37.1	2	234000000	3804	nofs	nofs
chr02	E	2q37.1	2	234000000	4577	nofs	nofs
chr02	E	2q37.1	2	235000000	3926	nofs	nofs
chr02	E	2q37.1	2	235000000	5183	nofs	nofs
chr02	E	2q37.1	2	235000000	4958	nofs	nofs
chr02	E	2q37.1	2	235000000	8393	nofs	nofs
chr02	E	2q37.2	1	236000000	5960	nofs	nofs
chr02	E	2q37.2	1	236000000	5264	nofs	nofs
chr02	E	2q37.2	1	236000000	5594	nofs	nofs
chr02	E	2q37.2	1	236000000	6957	nofs	nofs
chr02	E	2q37.2	1	237000000	3338	nofs	nofs
chr02	E	2q37.2	1	237000000	3930	nofs	nofs
chr02	E	2q37.3	2	237000000	6639	fs	fs common
chr02	E	2q37.3	2	237000000	6716	fs	fs common
chr02	E	2q37.3	2	238000000	5232	fs	fs common
chr02	E	2q37.3	2	238000000	3372	fs	fs common
chr02	E	2q37.3	2	238000000	9473	fs	fs common
chr02	E	2q37.3	2	238000000	3980	fs	fs common
chr02	E	2q37.3	2	239000000	3713	fs	fs common
chr02	E	2q37.3	2	239000000	7971	fs	fs common

chr02	E	2q37.3	2	239000000	25292	fs	fs common
chr02	E	2q37.3	2	239000000	9196	fs	fs common
chr02	T	2q37.3	2	240000000	9041	fs	fs common
chr02	T	2q37.3	2	240000000	11546	fs	fs common
chr02	T	2q37.3	2	240000000	10364	fs	fs common
chr02	T	2q37.3	2	240000000	17035	fs	fs common
chr02	T	2q37.3	2	241000000	10827	fs	fs common
chr02	T	2q37.3	2	241000000	15570	fs	fs common
chr02	T	2q37.3	2	241000000	9747	fs	fs common
chr02	T	2q37.3	2	241000000	12805	fs	fs common
chr02	T	2q37.3	2	242000000	19057	fs	fs common
chr02	T	2q37.3	2	242000000	8236	fs	fs common
chr02	T	2q37.3	2	242000000	10882	fs	fs common
chr02	T	2q37.3	2	242000000	20804	fs	fs common
chr02	T	2q37.3	2	243000000	23063	fs	fs common
chr03	T	3p25	6	125000	2999	nofs	nofs
chr03	T	3p25	6	375000	3788	nofs	nofs
chr03	T	3p25	6	625000	2641	nofs	nofs
chr03	T	3p25	6	875000	5923	nofs	nofs
chr03	T	3p25	6	1125000	3671	nofs	nofs
chr03	T	3p25	6	1375000	3151	nofs	nofs
chr03	T	3p25	6	1625000	4699	nofs	nofs
chr03	T	3p25	6	1875000	2243	nofs	nofs
chr03	T	3p25	6	2125000	2427	nofs	nofs
chr03	T	3p25	6	2375000	2948	nofs	nofs
chr03	T	3p25	6	2625000	2293	nofs	nofs
chr03	T	3p25	6	2875000	1784	nofs	nofs
chr03	T	3p25	6	3125000	1671	nofs	nofs
chr03	E	3p25	6	3375000	2497	nofs	nofs
chr03	E	3p25	6	3625000	1474	nofs	nofs
chr03	E	3p25	6	3875000	2352	nofs	nofs
chr03	E	3p25	6	4125000	2063	nofs	nofs
chr03	E	3p25	6	4375000	2656	nofs	nofs
chr03	E	3p25	6	4625000	1920	nofs	nofs
chr03	E	3p25	6	4875000	2607	nofs	nofs
chr03	E	3p25	6	5125000	4067	nofs	nofs

chr03	E	3p25	6	5375000	2546	nofs	nofs
chr03	E	3p25	6	5625000	2581	nofs	nofs
chr03	E	3p25	6	5875000	1971	nofs	nofs
chr03	E	3p25	6	6125000	2956	nofs	nofs
chr03	E	3p25	6	6375000	2853	nofs	nofs
chr03	E	3p25	6	6625000	3332	nofs	nofs
chr03	E	3p25	6	6875000	1735	nofs	nofs
chr03	E	3p25	6	7125000	2623	nofs	nofs
chr03	E	3p25	6	7375000	3140	nofs	nofs
chr03	E	3p25	6	7625000	1916	nofs	nofs
chr03	E	3p25	6	7875000	1764	nofs	nofs
chr03	E	3p25	6	8125000	3723	nofs	nofs
chr03	E	3p25	6	8375000	5161	nofs	nofs
chr03	E	3p25	6	8625000	10799	nofs	nofs
chr03	E	3p25	6	8875000	3068	nofs	nofs
chr03	E	3p25	6	9125000	1723	nofs	nofs
chr03	E	3p25	6	9375000	1873	nofs	nofs
chr03	E	3p25	6	9625000	5062	nofs	nofs
chr03	E	3p25	6	9875000	4516	nofs	nofs
chr03	E	3p25	6	10125000	4487	nofs	nofs
chr03	E	3p25	6	10375000	3434	nofs	nofs
chr03	E	3p25	6	10625000	3572	nofs	nofs
chr03	E	3p25	6	10875000	2794	nofs	nofs
chr03	E	3p25	6	11125000	5532	nofs	nofs
chr03	E	3p25	6	11375000	3340	nofs	nofs
chr03	E	3p25	6	11625000	4394	nofs	nofs
chr03	E	3p25	6	11875000	6053	nofs	nofs
chr03	E	3p25	6	12125000	1085	nofs	nofs
chr03	E	3p25	6	12375000	2925	nofs	nofs
chr03	E	3p25	6	12625000	2433	nofs	nofs
chr03	E	3p25	6	12875000	3977	nofs	nofs
chr03	E	3p25	6	13125000	3123	nofs	nofs
chr03	E	3p25	6	13375000	2582	nofs	nofs
chr03	E	3p25	6	13625000	8047	nofs	nofs
chr03	E	3p25	6	13875000	4426	nofs	nofs
chr03	E	3p25	6	14125000	4277	nofs	nofs

chr03	E	3p25	6	14375000	3285	nofs	nofs
chr03	E	3p25	6	14625000	1929	nofs	nofs
chr03	E	3p25	6	14875000	2899	nofs	nofs
chr03	E	3p25	6	15125000	3969	nofs	nofs
chr03	E	3p25	6	15375000	2367	nofs	nofs
chr03	E	3p25	6	15625000	3114	nofs	nofs
chr03	E	3p25	6	15875000	2945	nofs	nofs
chr03	E	3p25	6	16125000	2387	nofs	nofs
chr03	E	3p25	6	16375000	2851	nofs	nofs
chr03	E	3p24	2	16625000	2921	nofs	nofs
chr03	E	3p24	2	16875000	2201	nofs	nofs
chr03	E	3p24	2	17125000	1495	nofs	nofs
chr03	E	3p24	2	17375000	2343	nofs	nofs
chr03	E	3p24	2	17625000	3102	nofs	nofs
chr03	E	3p24	2	17875000	3459	nofs	nofs
chr03	E	3p24	2	18125000	1363	nofs	nofs
chr03	E	3p24	2	18375000	1662	nofs	nofs
chr03	E	3p24	2	18625000	2132	nofs	nofs
chr03	E	3p24	2	18875000	4069	nofs	nofs
chr03	E	3p24	2	19125000	1960	nofs	nofs
chr03	E	3p24	2	19375000	2366	nofs	nofs
chr03	E	3p24	2	19625000	1615	nofs	nofs
chr03	E	3p24	2	19875000	3280	nofs	nofs
chr03	E	3p24	2	20125000	4010	nofs	nofs
chr03	E	3p24	2	20375000	2327	nofs	nofs
chr03	E	3p24	2	20625000	1410	nofs	nofs
chr03	E	3p24	2	20875000	3919	nofs	nofs
chr03	E	3p24	2	21125000	2306	nofs	nofs
chr03	E	3p24	2	21375000	2831	nofs	nofs
chr03	E	3p24	2	21625000	2072	nofs	nofs
chr03	E	3p24	2	21875000	1744	nofs	nofs
chr03	E	3p24	2	22125000	1403	nofs	nofs
chr03	E	3p24	2	22375000	2396	nofs	nofs
chr03	E	3p24	2	22625000	1784	nofs	nofs
chr03	E	3p24	2	22875000	2621	nofs	nofs
chr03	E	3p24	2	23125000	2127	nofs	nofs

chr03	E	3p24	2	23375000	1516	nofs	nofs
chr03	E	3p24	2	23625000	2779	nofs	nofs
chr03	E	3p24	2	23875000	2701	fs	fs common
chr03	E	3p24	2	24125000	1880	fs	fs common
chr03	E	3p24	2	24375000	1947	fs	fs common
chr03	E	3p24	2	24625000	1944	fs	fs common
chr03	E	3p24	2	24875000	1615	fs	fs common
chr03	E	3p24	2	25125000	1468	fs	fs common
chr03	E	3p24	2	25375000	1129	fs	fs common
chr03	E	3p24	2	25625000	1835	fs	fs common
chr03	E	3p24	2	25875000	2309	fs	fs common
chr03	E	3p24	2	26125000	2326	fs	fs common
chr03	E	3p24	2	26375000	1258	fs	fs common
chr03	E	3p24	2	26625000	1679	fs	fs common
chr03	E	3p24	2	26875000	1653	fs	fs common
chr03	E	3p24	2	27125000	1906	fs	fs common
chr03	E	3p24	2	27375000	2602	fs	fs common
chr03	E	3p24	2	27625000	3075	nofs	nofs
chr03	E	3p24	2	27875000	3273	nofs	nofs
chr03	E	3p24	2	28125000	2133	nofs	nofs
chr03	E	3p24	2	28375000	2232	nofs	nofs
chr03	E	3p24	2	28625000	2657	nofs	nofs
chr03	E	3p24	2	28875000	2790	nofs	nofs
chr03	E	3p24	2	29125000	1571	nofs	nofs
chr03	E	3p24	2	29375000	2744	nofs	nofs
chr03	E	3p24	2	29625000	2292	nofs	nofs
chr03	E	3p24	2	29875000	2565	nofs	nofs
chr03	E	3p24	2	30125000	3779	nofs	nofs
chr03	E	3p24	2	30375000	1740	nofs	nofs
chr03	E	3p24	2	30625000	2735	nofs	nofs
chr03	E	3p24	2	30875000	1428	nofs	nofs
chr03	E	3p24	2	31125000	1368	nofs	nofs
chr03	E	3p24	2	31375000	2521	nofs	nofs
chr03	E	3p24	2	31625000	1794	nofs	nofs
chr03	E	3p24	2	31875000	3385	nofs	nofs
chr03	E	3p24	2	32125000	4122	nofs	nofs

chr03	E	3p24	2	32375000	2314	nofs	nofs
chr03	E	3p23	2	32625000	4468	nofs	nofs
chr03	E	3p23	2	32875000	4626	nofs	nofs
chr03	E	3p23	2	33125000	4070	nofs	nofs
chr03	E	3p23	2	33375000	3688	nofs	nofs
chr03	E	3p23	2	33625000	2009	nofs	nofs
chr03	E	3p23	2	33875000	3820	nofs	nofs
chr03	E	3p23	2	34125000	1522	nofs	nofs
chr03	E	3p23	2	34375000	2563	nofs	nofs
chr03	E	3p23	2	34625000	4667	nofs	nofs
chr03	E	3p23	2	34875000	1688	nofs	nofs
chr03	E	3p22	4	35125000	3179	nofs	nofs
chr03	E	3p22	4	35375000	2260	nofs	nofs
chr03	E	3p22	4	35625000	2957	nofs	nofs
chr03	E	3p22	4	35875000	2223	nofs	nofs
chr03	E	3p22	4	36125000	2197	nofs	nofs
chr03	E	3p22	4	36375000	3148	nofs	nofs
chr03	E	3p22	4	36625000	1596	nofs	nofs
chr03	E	3p22	4	36875000	3741	nofs	nofs
chr03	E	3p22	4	37125000	3354	nofs	nofs
chr03	E	3p22	4	37375000	2296	nofs	nofs
chr03	E	3p22	4	37625000	1721	nofs	nofs
chr03	E	3p22	4	37875000	1839	nofs	nofs
chr03	E	3p22	4	38125000	7391	nofs	nofs
chr03	E	3p22	4	38375000	2596	nofs	nofs
chr03	E	3p22	4	38625000	1639	nofs	nofs
chr03	E	3p22	4	38875000	2850	nofs	nofs
chr03	E	3p22	4	39125000	3027	nofs	nofs
chr03	E	3p22	4	39375000	3123	nofs	nofs
chr03	E	3p22	4	39625000	4576	nofs	nofs
chr03	E	3p22	4	39875000	1506	nofs	nofs
chr03	E	3p22	4	40125000	3812	nofs	nofs
chr03	E	3p22	4	40375000	2297	nofs	nofs
chr03	E	3p22	4	40625000	2210	nofs	nofs
chr03	E	3p22	4	40875000	2065	nofs	nofs
chr03	E	3p22	4	41125000	1517	nofs	nofs

chr03	E	3p22	4	41375000	2696	nofs	nofs
chr03	E	3p22	4	41625000	1614	nofs	nofs
chr03	E	3p22	4	41875000	3561	nofs	nofs
chr03	E	3p22	4	42125000	2500	nofs	nofs
chr03	E	3p22	4	42375000	2683	nofs	nofs
chr03	E	3p22	4	42625000	2330	nofs	nofs
chr03	E	3p22	4	42875000	5072	nofs	nofs
chr03	E	3p22	4	43125000	8180	nofs	nofs
chr03	E	3p22	4	43375000	1887	nofs	nofs
chr03	E	3p22	4	43625000	2385	nofs	nofs
chr03	E	3p21.3	4	43875000	2163	nofs	nofs
chr03	E	3p21.3	4	44125000	1964	nofs	nofs
chr03	E	3p21.3	4	44375000	1733	nofs	nofs
chr03	E	3p21.3	4	44625000	5611	nofs	nofs
chr03	E	3p21.3	4	44875000	3299	nofs	nofs
chr03	E	3p21.3	4	45125000	2394	nofs	nofs
chr03	E	3p21.3	4	45375000	2328	nofs	nofs
chr03	E	3p21.3	4	45625000	1509	nofs	nofs
chr03	E	3p21.3	4	45875000	1378	nofs	nofs
chr03	E	3p21.3	4	46125000	1963	nofs	nofs
chr03	E	3p21.3	4	46375000	2689	nofs	nofs
chr03	E	3p21.3	4	46625000	4140	nofs	nofs
chr03	E	3p21.3	4	46875000	2911	nofs	nofs
chr03	E	3p21.3	4	47125000	2086	nofs	nofs
chr03	E	3p21.3	4	47375000	3597	nofs	nofs
chr03	E	3p21.3	4	47625000	3419	nofs	nofs
chr03	E	3p21.3	4	47875000	5574	nofs	nofs
chr03	E	3p21.3	4	48125000	7428	nofs	nofs
chr03	E	3p21.3	4	48375000	2307	nofs	nofs
chr03	E	3p21.3	4	48625000	5432	nofs	nofs
chr03	E	3p21.3	4	48875000	8156	nofs	nofs
chr03	E	3p21.3	4	49125000	6395	nofs	nofs
chr03	E	3p21.3	4	49375000	12084	nofs	nofs
chr03	E	3p21.3	4	49625000	4996	nofs	nofs
chr03	E	3p21.3	4	49875000	5840	nofs	nofs
chr03	E	3p21.3	4	50125000	3621	nofs	nofs

chr03	E	3p21.3	4	50375000	3278	nofs	nofs
chr03	E	3p21.3	4	50625000	1367	nofs	nofs
chr03	E	3p21.3	4	50875000	3229	nofs	nofs
chr03	E	3p21.3	4	51125000	2601	nofs	nofs
chr03	E	3p21.3	4	51375000	3595	nofs	nofs
chr03	E	3p21.2	3	51625000	4863	nofs	nofs
chr03	E	3p21.2	3	51875000	4206	nofs	nofs
chr03	E	3p21.2	3	52125000	4564	nofs	nofs
chr03	E	3p21.2	3	52375000	3172	nofs	nofs
chr03	B	3p21.1	.	52625000	3408	nofs	nofs
chr03	B	3p21.1	.	52875000	3718	nofs	nofs
chr03	B	3p21.1	.	53125000	3755	nofs	nofs
chr03	B	3p21.1	.	53375000	2098	nofs	nofs
chr03	B	3p21.1	.	53625000	1812	nofs	nofs
chr03	B	3p21.1	.	53875000	3163	nofs	nofs
chr03	B	3p21.1	.	54125000	2480	nofs	nofs
chr03	B	3p21.1	.	54375000	2469	nofs	nofs
chr03	E	3p14.3	2	54625000	2663	nofs	nofs
chr03	E	3p14.3	2	54875000	2334	nofs	nofs
chr03	E	3p14.3	2	55125000	1779	nofs	nofs
chr03	E	3p14.3	2	55375000	1470	nofs	nofs
chr03	E	3p14.3	2	55625000	2222	nofs	nofs
chr03	E	3p14.3	2	55875000	2558	nofs	nofs
chr03	E	3p14.3	2	56125000	2131	nofs	nofs
chr03	E	3p14.3	2	56375000	2830	nofs	nofs
chr03	E	3p14.3	2	56625000	6508	nofs	nofs
chr03	E	3p14.3	2	56875000	3930	nofs	nofs
chr03	E	3p14.3	2	57125000	3527	nofs	nofs
chr03	E	3p14.3	2	57375000	3280	nofs	nofs
chr03	E	3p14.3	2	57625000	4989	nofs	nofs
chr03	E	3p14.3	2	57875000	4848	nofs	nofs
chr03	E	3p14.3	2	58125000	3422	nofs	nofs
chr03	E	3p14.3	2	58375000	3036	nofs	nofs
chr03	E	3p14.3	2	58625000	4264	fs	fs common
chr03	B	3p14.2	.	58875000	3722	fs	fs common
chr03	B	3p14.2	.	59125000	2129	fs	fs common

chr03	B	3p14.2	.	59375000	2344	fs	fs common
chr03	B	3p14.2	.	59625000	1968	fs	fs common
chr03	B	3p14.2	.	59875000	1737	fs	fs common
chr03	B	3p14.2	.	60125000	1725	fs	fs common
chr03	B	3p14.2	.	60375000	1264	fs	fs common
chr03	B	3p14.2	.	60625000	1663	fs	fs common
chr03	B	3p14.2	.	60875000	3450	fs	fs common
chr03	B	3p14.2	.	61125000	1565	fs	fs common
chr03	B	3p14.2	.	61375000	4569	fs	fs common
chr03	B	3p14.2	.	61625000	2061	fs	fs common
chr03	B	3p14.2	.	61875000	2578	fs	fs common
chr03	B	3p14.2	.	62125000	2039	fs	fs common
chr03	B	3p14.2	.	62375000	2146	fs	fs common
chr03	B	3p14.2	.	62625000	2003	fs	fs common
chr03	B	3p14.2	.	62875000	1636	fs	fs common
chr03	B	3p14.2	.	63125000	3910	fs	fs common
chr03	B	3p14.2	.	63375000	2673	fs	fs common
chr03	B	3p14.2	.	63625000	3008	fs	fs common
chr03	E	3p14.1	2	63875000	1656	nofs	nofs
chr03	E	3p14.1	2	64125000	2803	nofs	nofs
chr03	E	3p14.1	2	64375000	1997	nofs	nofs
chr03	E	3p14.1	2	64625000	1947	nofs	nofs
chr03	E	3p14.1	2	64875000	1630	nofs	nofs
chr03	E	3p14.1	2	65125000	3032	nofs	nofs
chr03	E	3p14.1	2	65375000	2903	nofs	nofs
chr03	E	3p14.1	2	65625000	2727	nofs	nofs
chr03	E	3p14.1	2	65875000	2397	nofs	nofs
chr03	E	3p14.1	2	66125000	1521	nofs	nofs
chr03	E	3p14.1	2	66375000	936	nofs	nofs
chr03	E	3p14.1	2	66625000	2696	nofs	nofs
chr03	E	3p14.1	2	66875000	3926	nofs	nofs
chr03	E	3p14.1	2	67125000	3144	nofs	nofs
chr03	E	3p14.1	2	67375000	1641	nofs	nofs
chr03	E	3p14.1	2	67625000	2809	nofs	nofs
chr03	E	3p14.1	2	67875000	1840	nofs	nofs
chr03	E	3p14.1	2	68125000	1913	nofs	nofs

chr03	E	3p14.1	2	68375000	2087	nofs	nofs
chr03	E	3p14.1	2	68625000	1795	nofs	nofs
chr03	E	3p14.1	2	68875000	2004	nofs	nofs
chr03	E	3p14.1	2	69125000	2594	nofs	nofs
chr03	E	3p14.1	2	69375000	3341	nofs	nofs
chr03	E	3p14.1	2	69625000	3291	nofs	nofs
chr03	E	3p14.1	2	69875000	3929	nofs	nofs
chr03	B	3p13	.	70125000	1964	nofs	nofs
chr03	B	3p13	.	70375000	2619	nofs	nofs
chr03	B	3p13	.	70625000	1585	nofs	nofs
chr03	B	3p13	.	70875000	1828	nofs	nofs
chr03	B	3p13	.	71125000	1913	nofs	nofs
chr03	B	3p13	.	71375000	2004	nofs	nofs
chr03	B	3p13	.	71625000	2714	nofs	nofs
chr03	B	3p13	.	71875000	1701	nofs	nofs
chr03	B	3p13	.	72125000	3331	nofs	nofs
chr03	B	3p13	.	72375000	2855	nofs	nofs
chr03	B	3p13	.	72625000	2059	nofs	nofs
chr03	B	3p13	.	72875000	3778	nofs	nofs
chr03	B	3p13	.	73125000	3505	nofs	nofs
chr03	B	3p13	.	73375000	3981	nofs	nofs
chr03	B	3p13	.	73625000	1432	nofs	nofs
chr03	B	3p13	.	73875000	1968	nofs	nofs
chr03	B	3p13	.	74125000	1000	nofs	nofs
chr03	E	3p12	5	74375000	1793	nofs	nofs
chr03	E	3p12	5	74625000	1702	nofs	nofs
chr03	E	3p12	5	74875000	3067	nofs	nofs
chr03	E	3p12	5	75125000	3148	nofs	nofs
chr03	E	3p12	5	75375000	4386	nofs	nofs
chr03	E	3p12	5	75625000	8102	nofs	nofs
chr03	E	3p12	5	75875000	15252	nofs	nofs
chr03	E	3p12	5	76125000	7389	nofs	nofs
chr03	E	3p12	5	76375000	6416	nofs	nofs
chr03	E	3p12	5	76625000	3069	nofs	nofs
chr03	E	3p12	5	76875000	4788	nofs	nofs
chr03	E	3p12	5	77125000	3433	nofs	nofs

chr03	E	3p12	5	77375000	11097	nofs	nofs
chr03	E	3p12	5	77625000	2644	nofs	nofs
chr03	E	3p12	5	77875000	2008	nofs	nofs
chr03	E	3p12	5	78125000	2442	nofs	nofs
chr03	E	3p12	5	78375000	3911	nofs	nofs
chr03	E	3p12	5	78625000	3641	nofs	nofs
chr03	E	3p12	5	78875000	3259	nofs	nofs
chr03	E	3p12	5	79125000	2696	nofs	nofs
chr03	E	3p12	5	79375000	1416	nofs	nofs
chr03	E	3p12	5	79625000	3362	nofs	nofs
chr03	E	3p12	5	79875000	2989	nofs	nofs
chr03	E	3p12	5	80125000	4383	nofs	nofs
chr03	E	3p12	5	80375000	2672	nofs	nofs
chr03	E	3p12	5	80625000	2821	nofs	nofs
chr03	E	3p12	5	80875000	3161	nofs	nofs
chr03	E	3p12	5	81125000	21051	nofs	nofs
chr03	E	3p12	5	81375000	3896	nofs	nofs
chr03	E	3p12	5	81625000	2098	nofs	nofs
chr03	E	3p12	5	81875000	2888	nofs	nofs
chr03	E	3p12	5	82125000	1848	nofs	nofs
chr03	E	3p12	5	82375000	2166	nofs	nofs
chr03	E	3p12	5	82625000	4660	nofs	nofs
chr03	E	3p12	5	82875000	2683	nofs	nofs
chr03	E	3p12	5	83125000	5269	nofs	nofs
chr03	E	3p12	5	83375000	2214	nofs	nofs
chr03	E	3p12	5	83625000	2679	nofs	nofs
chr03	E	3p12	5	83875000	3407	nofs	nofs
chr03	E	3p12	5	84125000	2695	nofs	nofs
chr03	E	3p12	5	84375000	3186	nofs	nofs
chr03	E	3p12	5	84625000	3121	nofs	nofs
chr03	E	3p12	5	84875000	2417	nofs	nofs
chr03	E	3p12	5	85125000	3498	nofs	nofs
chr03	E	3p12	5	85375000	2749	nofs	nofs
chr03	E	3p12	5	85625000	3002	nofs	nofs
chr03	E	3p12	5	85875000	4090	nofs	nofs
chr03	E	3p12	5	86125000	2700	nofs	nofs

chr03	E	3p12	5	86375000	3451	nofs	nofs
chr03	E	3p12	5	86625000	3086	nofs	nofs
chr03	E	3p12	5	86875000	5117	nofs	nofs
chr03	E	3p12	5	87125000	2173	nofs	nofs
chr03	E	3p12	5	87375000	1671	nofs	nofs
chr03	C	3p11	.	87625000	2011	nofs	nofs
chr03	C	3p11	.	87875000	2531	nofs	nofs
chr03	C	3p11	.	88125000	2949	nofs	nofs
chr03	C	3p11	.	88375000	2395	nofs	nofs
chr03	C	3p11	.	88625000	2724	nofs	nofs
chr03	C	3p11	.	88875000	2415	nofs	nofs
chr03	C	3p11	.	89125000	2645	nofs	nofs
chr03	C	3p11	.	89375000	2623	nofs	nofs
chr03	C	3p11	.	89625000	4053	nofs	nofs
chr03	C	3p11	.	89875000	2776	nofs	nofs
chr03	C	3p11	.	90125000	2421	nofs	nofs
chr03	C	3p11	.	90375000	84389	nofs	nofs
chr03	C	3p11	.	90625000	67775	nofs	nofs
chr03	C		.	90875000	0	nofs	nofs
chr03	C		.	91125000	0	nofs	nofs
chr03	C		.	91375000	0	nofs	nofs
chr03	C		.	91625000	0	nofs	nofs
chr03	C		.	91875000	0	nofs	nofs
chr03	C		.	92125000	0	nofs	nofs
chr03	C		.	92375000	0	nofs	nofs
chr03	C		.	92625000	0	nofs	nofs
chr03	C		.	92875000	0	nofs	nofs
chr03	C		.	93125000	0	nofs	nofs
chr03	C		.	93375000	0	nofs	nofs
chr03	C		.	93625000	0	nofs	nofs
chr03	C		.	93875000	0	nofs	nofs
chr03	C		.	94125000	0	nofs	nofs
chr03	C		.	94375000	0	nofs	nofs
chr03	C		.	94625000	0	nofs	nofs
chr03	C	3q11.1	.	94875000	10086	nofs	nofs
chr03	C	3q11.1	.	95125000	4803	nofs	nofs

chr03	C	3q11.1	.	95375000	1305	nofs	nofs
chr03	C	3q11.1	.	95625000	1695	nofs	nofs
chr03	C	3q11.1	.	95875000	2366	nofs	nofs
chr03	C	3q11.1	.	96125000	2840	nofs	nofs
chr03	C	3q11.2	1	96375000	1528	nofs	nofs
chr03	C	3q11.2	1	96625000	3066	nofs	nofs
chr03	C	3q11.2	1	96875000	3769	nofs	nofs
chr03	C	3q11.2	1	97125000	2906	nofs	nofs
chr03	C	3q11.2	1	97375000	3004	nofs	nofs
chr03	C	3q11.2	1	97625000	2579	nofs	nofs
chr03	C	3q11.2	1	97875000	4313	nofs	nofs
chr03	E	3q11.2	1	98125000	1426	nofs	nofs
chr03	E	3q11.2	1	98375000	2905	nofs	nofs
chr03	E	3q11.2	1	98625000	1784	nofs	nofs
chr03	E	3q11.2	1	98875000	1301	nofs	nofs
chr03	E	3q11.2	1	99125000	3139	nofs	nofs
chr03	E	3q12	1	99375000	2464	nofs	nofs
chr03	E	3q12	1	99625000	1607	nofs	nofs
chr03	E	3q12	1	99875000	4780	nofs	nofs
chr03	E	3q12	1	100000000	3999	nofs	nofs
chr03	E	3q12	1	100000000	2314	nofs	nofs
chr03	E	3q12	1	101000000	1267	nofs	nofs
chr03	E	3q12	1	101000000	1554	nofs	nofs
chr03	E	3q12	1	101000000	1788	nofs	nofs
chr03	E	3q12	1	101000000	1841	nofs	nofs
chr03	E	3q12	1	102000000	3582	nofs	nofs
chr03	E	3q12	1	102000000	1744	nofs	nofs
chr03	E	3q12	1	102000000	1461	nofs	nofs
chr03	E	3q12	1	102000000	1517	nofs	nofs
chr03	E	3q12	1	103000000	3443	nofs	nofs
chr03	E	3q12	1	103000000	4367	nofs	nofs
chr03	E	3q12	1	103000000	2980	nofs	nofs
chr03	E	3q12	1	103000000	1091	nofs	nofs
chr03	E	3q12	1	104000000	2304	nofs	nofs
chr03	E	3q12	1	104000000	1509	nofs	nofs
chr03	E	3q12	1	104000000	2743	nofs	nofs

chr03	E	3q12	1	104000000	3226	nofs	nofs
chr03	B	3q13.1	.	105000000	3024	nofs	nofs
chr03	B	3q13.1	.	105000000	3056	nofs	nofs
chr03	B	3q13.1	.	105000000	2613	nofs	nofs
chr03	B	3q13.1	.	105000000	1617	nofs	nofs
chr03	B	3q13.1	.	106000000	1836	nofs	nofs
chr03	B	3q13.1	.	106000000	1855	nofs	nofs
chr03	B	3q13.1	.	106000000	2283	nofs	nofs
chr03	B	3q13.1	.	106000000	2213	nofs	nofs
chr03	B	3q13.1	.	107000000	1802	nofs	nofs
chr03	B	3q13.1	.	107000000	2466	nofs	nofs
chr03	B	3q13.1	.	107000000	1688	nofs	nofs
chr03	B	3q13.1	.	107000000	6172	nofs	nofs
chr03	B	3q13.1	.	108000000	4357	nofs	nofs
chr03	B	3q13.1	.	108000000	2296	nofs	nofs
chr03	B	3q13.1	.	108000000	2416	nofs	nofs
chr03	B	3q13.1	.	108000000	1537	nofs	nofs
chr03	B	3q13.1	.	109000000	1582	nofs	nofs
chr03	B	3q13.1	.	109000000	1732	nofs	nofs
chr03	B	3q13.1	.	109000000	1931	nofs	nofs
chr03	B	3q13.1	.	109000000	1795	nofs	nofs
chr03	B	3q13.1	.	110000000	1384	nofs	nofs
chr03	B	3q13.1	.	110000000	2970	nofs	nofs
chr03	B	3q13.1	.	110000000	2387	nofs	nofs
chr03	B	3q13.1	.	110000000	2949	nofs	nofs
chr03	B	3q13.1	.	111000000	2991	nofs	nofs
chr03	B	3q13.1	.	111000000	1646	nofs	nofs
chr03	B	3q13.1	.	111000000	2599	nofs	nofs
chr03	B	3q13.1	.	111000000	2639	nofs	nofs
chr03	B	3q13.1	.	112000000	1340	nofs	nofs
chr03	B	3q13.1	.	112000000	1604	nofs	nofs
chr03	B	3q13.1	.	112000000	3339	nofs	nofs
chr03	B	3q13.1	.	112000000	1915	nofs	nofs
chr03	B	3q13.1	.	113000000	1485	nofs	nofs
chr03	B	3q13.1	.	113000000	1738	nofs	nofs
chr03	E	3q13.2	1	113000000	3484	nofs	nofs

chr03	E	3q13.2	1	113000000	2184	nofs	nofs
chr03	E	3q13.2	1	114000000	5288	nofs	nofs
chr03	E	3q13.2	1	114000000	1790	nofs	nofs
chr03	E	3q13.2	1	114000000	2062	nofs	nofs
chr03	E	3q13.2	1	114000000	2921	nofs	nofs
chr03	E	3q13.2	1	115000000	3391	nofs	nofs
chr03	E	3q13.2	1	115000000	4074	nofs	nofs
chr03	E	3q13.2	1	115000000	2113	nofs	nofs
chr03	E	3q13.3	1	115000000	3335	nofs	nofs
chr03	E	3q13.3	1	116000000	1854	nofs	nofs
chr03	E	3q13.3	1	116000000	2505	nofs	nofs
chr03	E	3q13.3	1	116000000	2624	nofs	nofs
chr03	E	3q13.3	1	116000000	1958	nofs	nofs
chr03	E	3q13.3	1	117000000	2506	nofs	nofs
chr03	E	3q13.3	1	117000000	2230	nofs	nofs
chr03	E	3q13.3	1	117000000	2151	nofs	nofs
chr03	E	3q13.3	1	117000000	2282	nofs	nofs
chr03	E	3q13.3	1	118000000	2634	nofs	nofs
chr03	E	3q13.3	1	118000000	2126	nofs	nofs
chr03	E	3q13.3	1	118000000	1694	nofs	nofs
chr03	E	3q13.3	1	118000000	3206	nofs	nofs
chr03	E	3q13.3	1	119000000	2082	nofs	nofs
chr03	E	3q13.3	1	119000000	2396	nofs	nofs
chr03	E	3q13.3	1	119000000	2935	nofs	nofs
chr03	E	3q13.3	1	119000000	2206	nofs	nofs
chr03	E	3q13.3	1	120000000	1453	nofs	nofs
chr03	E	3q13.3	1	120000000	2623	nofs	nofs
chr03	E	3q13.3	1	120000000	1757	nofs	nofs
chr03	E	3q13.3	1	120000000	3187	nofs	nofs
chr03	E	3q13.3	1	121000000	2046	nofs	nofs
chr03	E	3q13.3	1	121000000	2431	nofs	nofs
chr03	E	3q13.3	1	121000000	2938	nofs	nofs
chr03	E	3q13.3	1	121000000	2349	nofs	nofs
chr03	E	3q13.3	1	122000000	2052	nofs	nofs
chr03	E	3q13.3	1	122000000	2641	nofs	nofs
chr03	E	3q13.3	1	122000000	1617	nofs	nofs

chr03	E	3q13.3	1	122000000	3713	nofs	nofs
chr03	E	3q13.3	1	123000000	3488	nofs	nofs
chr03	E	3q13.3	1	123000000	5758	nofs	nofs
chr03	E	3q13.3	1	123000000	1645	nofs	nofs
chr03	E	3q13.3	1	123000000	3566	nofs	nofs
chr03	E	3q21	5	124000000	2984	nofs	nofs
chr03	E	3q21	5	124000000	4185	nofs	nofs
chr03	E	3q21	5	124000000	2271	nofs	nofs
chr03	E	3q21	5	124000000	2328	nofs	nofs
chr03	E	3q21	5	125000000	3022	nofs	nofs
chr03	E	3q21	5	125000000	3137	nofs	nofs
chr03	E	3q21	5	125000000	2114	nofs	nofs
chr03	E	3q21	5	125000000	2787	nofs	nofs
chr03	E	3q21	5	126000000	4499	nofs	nofs
chr03	E	3q21	5	126000000	5445	nofs	nofs
chr03	E	3q21	5	126000000	3693	nofs	nofs
chr03	E	3q21	5	126000000	1433	nofs	nofs
chr03	E	3q21	5	127000000	1984	nofs	nofs
chr03	E	3q21	5	127000000	17036	nofs	nofs
chr03	E	3q21	5	127000000	2734	nofs	nofs
chr03	E	3q21	5	127000000	3779	nofs	nofs
chr03	E	3q21	5	128000000	6123	nofs	nofs
chr03	E	3q21	5	128000000	2443	nofs	nofs
chr03	E	3q21	5	128000000	5804	nofs	nofs
chr03	E	3q21	5	128000000	7453	nofs	nofs
chr03	E	3q21	5	129000000	5321	nofs	nofs
chr03	E	3q21	5	129000000	5810	nofs	nofs
chr03	E	3q21	5	129000000	2597	nofs	nofs
chr03	E	3q21	5	129000000	2429	nofs	nofs
chr03	E	3q21	5	130000000	4810	nofs	nofs
chr03	E	3q21	5	130000000	5148	nofs	nofs
chr03	E	3q21	5	130000000	4430	nofs	nofs
chr03	E	3q21	5	130000000	4267	nofs	nofs
chr03	E	3q21	5	131000000	8692	nofs	nofs
chr03	E	3q21	5	131000000	2387	nofs	nofs
chr03	E	3q21	5	131000000	5765	nofs	nofs

chr03	E	3q21	5	131000000	3178	nofs	nofs
chr03	E	3q21	5	132000000	2444	nofs	nofs
chr03	E	3q21	5	132000000	2244	nofs	nofs
chr03	E	3q21	5	132000000	3230	nofs	nofs
chr03	E	3q21	5	132000000	2105	nofs	nofs
chr03	E	3q21	5	133000000	2456	nofs	nofs
chr03	E	3q22	3	133000000	1822	nofs	nofs
chr03	E	3q22	3	133000000	3875	nofs	nofs
chr03	E	3q22	3	133000000	3075	nofs	nofs
chr03	E	3q22	3	134000000	3692	nofs	nofs
chr03	E	3q22	3	134000000	1788	nofs	nofs
chr03	E	3q22	3	134000000	2758	nofs	nofs
chr03	E	3q22	3	134000000	1604	nofs	nofs
chr03	E	3q22	3	135000000	6714	nofs	nofs
chr03	E	3q22	3	135000000	1973	nofs	nofs
chr03	E	3q22	3	135000000	2898	nofs	nofs
chr03	E	3q22	3	135000000	3197	nofs	nofs
chr03	E	3q22	3	136000000	2910	nofs	nofs
chr03	E	3q22	3	136000000	2959	nofs	nofs
chr03	E	3q22	3	136000000	2774	nofs	nofs
chr03	E	3q22	3	136000000	1859	nofs	nofs
chr03	E	3q22	3	137000000	1173	nofs	nofs
chr03	E	3q22	3	137000000	1407	nofs	nofs
chr03	E	3q22	3	137000000	2520	nofs	nofs
chr03	E	3q22	3	137000000	2257	nofs	nofs
chr03	E	3q22	3	138000000	2772	nofs	nofs
chr03	E	3q22	3	138000000	5348	nofs	nofs
chr03	E	3q22	3	138000000	2552	nofs	nofs
chr03	E	3q22	3	138000000	2941	nofs	nofs
chr03	E	3q22	3	139000000	1458	nofs	nofs
chr03	E	3q22	3	139000000	2332	nofs	nofs
chr03	E	3q22	3	139000000	2523	nofs	nofs
chr03	E	3q22	3	139000000	3164	nofs	nofs
chr03	E	3q22	3	140000000	2768	nofs	nofs
chr03	E	3q22	3	140000000	5702	nofs	nofs
chr03	E	3q22	3	140000000	4637	nofs	nofs

chr03	E	3q22	3	140000000	3748	nofs	nofs
chr03	E	3q23	1	141000000	1420	nofs	nofs
chr03	E	3q23	1	141000000	2111	nofs	nofs
chr03	E	3q23	1	141000000	1556	nofs	nofs
chr03	E	3q23	1	141000000	2281	nofs	nofs
chr03	E	3q23	1	142000000	2038	nofs	nofs
chr03	E	3q23	1	142000000	2927	nofs	nofs
chr03	E	3q23	1	142000000	3428	nofs	nofs
chr03	E	3q23	1	142000000	2447	nofs	nofs
chr03	E	3q23	1	143000000	2067	nofs	nofs
chr03	E	3q23	1	143000000	3064	nofs	nofs
chr03	E	3q23	1	143000000	7581	nofs	nofs
chr03	E	3q23	1	143000000	3555	nofs	nofs
chr03	E	3q23	1	144000000	3013	nofs	nofs
chr03	E	3q23	1	144000000	2525	nofs	nofs
chr03	E	3q23	1	144000000	3031	nofs	nofs
chr03	E	3q23	1	144000000	2186	nofs	nofs
chr03	E	3q24	3	145000000	2986	nofs	nofs
chr03	E	3q24	3	145000000	1325	nofs	nofs
chr03	E	3q24	3	145000000	2246	nofs	nofs
chr03	E	3q24	3	145000000	2815	nofs	nofs
chr03	E	3q24	3	146000000	2630	nofs	nofs
chr03	E	3q24	3	146000000	2834	nofs	nofs
chr03	E	3q24	3	146000000	3824	nofs	nofs
chr03	E	3q24	3	146000000	2381	nofs	nofs
chr03	E	3q24	3	147000000	1731	nofs	nofs
chr03	E	3q24	3	147000000	3563	nofs	nofs
chr03	E	3q24	3	147000000	1887	nofs	nofs
chr03	E	3q24	3	147000000	2935	nofs	nofs
chr03	E	3q24	3	148000000	1625	nofs	nofs
chr03	E	3q24	3	148000000	3044	nofs	nofs
chr03	E	3q24	3	148000000	1852	nofs	nofs
chr03	E	3q24	3	148000000	3263	nofs	nofs
chr03	E	3q24	3	149000000	1972	nofs	nofs
chr03	E	3q24	3	149000000	1945	nofs	nofs
chr03	E	3q24	3	149000000	2646	nofs	nofs

chr03	E	3q24	3	149000000	2784	nofs	nofs
chr03	E	3q24	3	150000000	3046	nofs	nofs
chr03	E	3q24	3	150000000	2850	nofs	nofs
chr03	E	3q24	3	150000000	1620	nofs	nofs
chr03	E	3q24	3	150000000	2329	nofs	nofs
chr03	B	3q25.1	.	151000000	3947	fs	fs common
chr03	B	3q25.1	.	151000000	2555	fs	fs common
chr03	B	3q25.1	.	151000000	2385	fs	fs common
chr03	B	3q25.1	.	151000000	1914	fs	fs common
chr03	B	3q25.1	.	152000000	1746	fs	fs common
chr03	B	3q25.1	.	152000000	2178	fs	fs common
chr03	B	3q25.1	.	152000000	4722	fs	fs common
chr03	B	3q25.1	.	152000000	1921	fs	fs common
chr03	B	3q25.1	.	153000000	1590	fs	fs common
chr03	B	3q25.1	.	153000000	3138	fs	fs common
chr03	B	3q25.1	.	153000000	3696	fs	fs common
chr03	B	3q25.1	.	153000000	3063	fs	fs common
chr03	B	3q25.1	.	154000000	2292	fs	fs common
chr03	B	3q25.2	.	154000000	3610	fs	fs common
chr03	B	3q25.2	.	154000000	3095	fs	fs common
chr03	B	3q25.2	.	154000000	3449	fs	fs common
chr03	B	3q25.2	.	155000000	3437	fs	fs common
chr03	B	3q25.2	.	155000000	2458	fs	fs common
chr03	B	3q25.2	.	155000000	3182	fs	fs common
chr03	B	3q25.2	.	155000000	1694	fs	fs common
chr03	B	3q25.2	.	156000000	2239	fs	fs common
chr03	B	3q25.2	.	156000000	2378	fs	fs common
chr03	B	3q25.2	.	156000000	1971	fs	fs common
chr03	B	3q25.2	.	156000000	2590	fs	fs common
chr03	E	3q25.3	1	157000000	2558	fs	fs common
chr03	E	3q25.3	1	157000000	5140	fs	fs common
chr03	E	3q25.3	1	157000000	4600	fs	fs common
chr03	E	3q25.3	1	157000000	2842	fs	fs common
chr03	E	3q25.3	1	158000000	1588	fs	fs common
chr03	E	3q25.3	1	158000000	2201	fs	fs common
chr03	E	3q25.3	1	158000000	2337	fs	fs common

chr03	E	3q25.3	1	158000000	4205	fs	fs common
chr03	E	3q25.3	1	159000000	2240	fs	fs common
chr03	E	3q25.3	1	159000000	1617	fs	fs common
chr03	E	3q25.3	1	159000000	1694	fs	fs common
chr03	E	3q25.3	1	159000000	2207	fs	fs common
chr03	E	3q25.3	1	160000000	2216	fs	fs common
chr03	E	3q25.3	1	160000000	3267	fs	fs common
chr03	E	3q25.3	1	160000000	1382	fs	fs common
chr03	E	3q25.3	1	160000000	1581	fs	fs common
chr03	E	3q25.3	1	161000000	1274	fs	fs common
chr03	E	3q25.3	1	161000000	2653	fs	fs common
chr03	E	3q25.3	1	161000000	2822	fs	fs common
chr03	E	3q25.3	1	161000000	3557	fs	fs common
chr03	E	3q25.3	1	162000000	1847	fs	fs common
chr03	E	3q25.3	1	162000000	2044	fs	fs common
chr03	E	3q25.3	1	162000000	3500	fs	fs common
chr03	E	3q26.1	2	162000000	1565	nofs	nofs
chr03	E	3q26.1	2	163000000	8393	nofs	nofs
chr03	E	3q26.1	2	163000000	3109	nofs	nofs
chr03	E	3q26.1	2	163000000	2540	nofs	nofs
chr03	E	3q26.1	2	163000000	2950	nofs	nofs
chr03	E	3q26.1	2	164000000	3481	nofs	nofs
chr03	E	3q26.1	2	164000000	4494	nofs	nofs
chr03	E	3q26.1	2	164000000	2189	nofs	nofs
chr03	E	3q26.1	2	164000000	2609	nofs	nofs
chr03	E	3q26.1	2	165000000	3486	nofs	nofs
chr03	E	3q26.1	2	165000000	2574	nofs	nofs
chr03	E	3q26.1	2	165000000	2434	nofs	nofs
chr03	E	3q26.1	2	165000000	1561	nofs	nofs
chr03	E	3q26.1	2	166000000	3197	nofs	nofs
chr03	E	3q26.1	2	166000000	2578	nofs	nofs
chr03	E	3q26.1	2	166000000	2880	nofs	nofs
chr03	E	3q26.1	2	166000000	3288	nofs	nofs
chr03	E	3q26.1	2	167000000	2781	nofs	nofs
chr03	E	3q26.1	2	167000000	2458	nofs	nofs
chr03	E	3q26.1	2	167000000	1713	nofs	nofs

chr03	E	3q26.1	2	167000000	2676	nofs	nofs
chr03	E	3q26.1	2	168000000	2454	nofs	nofs
chr03	E	3q26.1	2	168000000	2311	nofs	nofs
chr03	E	3q26.1	2	168000000	3824	nofs	nofs
chr03	E	3q26.1	2	168000000	1785	nofs	nofs
chr03	E	3q26.1	2	169000000	2344	nofs	nofs
chr03	E	3q26.1	2	169000000	2679	nofs	nofs
chr03	E	3q26.1	2	169000000	2749	nofs	nofs
chr03	E	3q26.1	2	169000000	7434	nofs	nofs
chr03	E	3q26.2	2	170000000	1434	nofs	nofs
chr03	E	3q26.2	2	170000000	2014	nofs	nofs
chr03	E	3q26.2	2	170000000	4539	nofs	nofs
chr03	E	3q26.2	2	170000000	1943	nofs	nofs
chr03	E	3q26.2	2	171000000	2569	nofs	nofs
chr03	E	3q26.2	2	171000000	2759	nofs	nofs
chr03	E	3q26.2	2	171000000	1947	nofs	nofs
chr03	E	3q26.2	2	171000000	2528	nofs	nofs
chr03	E	3q26.2	2	172000000	2907	nofs	nofs
chr03	E	3q26.2	2	172000000	2231	nofs	nofs
chr03	E	3q26.2	2	172000000	3114	nofs	nofs
chr03	E	3q26.2	2	172000000	1174	nofs	nofs
chr03	E	3q26.2	2	173000000	2730	nofs	nofs
chr03	E	3q26.2	2	173000000	4619	nofs	nofs
chr03	E	3q26.3	3	173000000	3847	nofs	nofs
chr03	E	3q26.3	3	173000000	2347	nofs	nofs
chr03	E	3q26.3	3	174000000	2488	nofs	nofs
chr03	E	3q26.3	3	174000000	2562	nofs	nofs
chr03	E	3q26.3	3	174000000	2078	nofs	nofs
chr03	E	3q26.3	3	174000000	1926	nofs	nofs
chr03	E	3q26.3	3	175000000	2517	nofs	nofs
chr03	E	3q26.3	3	175000000	2271	nofs	nofs
chr03	E	3q26.3	3	175000000	3406	nofs	nofs
chr03	E	3q26.3	3	175000000	3433	nofs	nofs
chr03	E	3q26.3	3	176000000	3884	nofs	nofs
chr03	E	3q26.3	3	176000000	1641	nofs	nofs
chr03	E	3q26.3	3	176000000	3774	nofs	nofs

chr03	E	3q26.3	3	176000000	3273	nofs	nofs
chr03	E	3q26.3	3	177000000	2883	nofs	nofs
chr03	E	3q26.3	3	177000000	4398	nofs	nofs
chr03	E	3q26.3	3	177000000	2918	nofs	nofs
chr03	E	3q26.3	3	177000000	4993	nofs	nofs
chr03	E	3q26.3	3	178000000	2462	nofs	nofs
chr03	E	3q26.3	3	178000000	3609	nofs	nofs
chr03	E	3q26.3	3	178000000	2465	nofs	nofs
chr03	E	3q26.3	3	178000000	4365	nofs	nofs
chr03	E	3q26.3	3	179000000	4122	nofs	nofs
chr03	E	3q26.3	3	179000000	2408	nofs	nofs
chr03	E	3q26.3	3	179000000	3267	nofs	nofs
chr03	E	3q26.3	3	179000000	3305	nofs	nofs
chr03	E	3q26.3	3	180000000	1897	nofs	nofs
chr03	E	3q26.3	3	180000000	4771	nofs	nofs
chr03	E	3q26.3	3	180000000	3572	nofs	nofs
chr03	E	3q26.3	3	180000000	4256	nofs	nofs
chr03	E	3q26.3	3	181000000	2191	nofs	nofs
chr03	E	3q26.3	3	181000000	3380	nofs	nofs
chr03	E	3q26.3	3	181000000	2313	nofs	nofs
chr03	E	3q26.3	3	181000000	2726	nofs	nofs
chr03	E	3q26.3	3	182000000	1518	nofs	nofs
chr03	E	3q26.3	3	182000000	3067	nofs	nofs
chr03	E	3q26.3	3	182000000	2398	nofs	nofs
chr03	E	3q26.3	3	182000000	5043	nofs	nofs
chr03	E	3q26.3	3	183000000	4139	nofs	nofs
chr03	E	3q26.3	3	183000000	2623	nofs	nofs
chr03	E	3q26.3	3	183000000	5132	nofs	nofs
chr03	E	3q26.3	3	183000000	2516	nofs	nofs
chr03	E	3q26.3	3	184000000	1824	nofs	nofs
chr03	E	3q26.3	3	184000000	3412	nofs	nofs
chr03	E	3q26.3	3	184000000	3808	nofs	nofs
chr03	E	3q27	3	184000000	3884	fs	fs common
chr03	E	3q27	3	185000000	2584	fs	fs common
chr03	E	3q27	3	185000000	5352	fs	fs common
chr03	E	3q27	3	185000000	4980	fs	fs common

chr03	E	3q27	3	185000000	5763	fs	fs common
chr03	E	3q27	3	186000000	5859	fs	fs common
chr03	E	3q27	3	186000000	7409	fs	fs common
chr03	E	3q27	3	186000000	1962	fs	fs common
chr03	E	3q27	3	186000000	3719	fs	fs common
chr03	E	3q27	3	187000000	5581	fs	fs common
chr03	E	3q27	3	187000000	4036	fs	fs common
chr03	E	3q27	3	187000000	4480	fs	fs common
chr03	E	3q27	3	187000000	2776	fs	fs common
chr03	E	3q27	3	188000000	2876	fs	fs common
chr03	E	3q27	3	188000000	3456	fs	fs common
chr03	E	3q27	3	188000000	6410	fs	fs common
chr03	E	3q27	3	188000000	2315	fs	fs common
chr03	E	3q27	3	189000000	3714	fs	fs common
chr03	E	3q27	3	189000000	3751	fs	fs common
chr03	E	3q27	3	189000000	1457	fs	fs common
chr03	E	3q27	3	189000000	3737	fs	fs common
chr03	E	3q27	3	190000000	3365	fs	fs common
chr03	B	3q28	.	190000000	4097	nofs	nofs
chr03	B	3q28	.	190000000	1690	nofs	nofs
chr03	B	3q28	.	190000000	2807	nofs	nofs
chr03	B	3q28	.	191000000	3358	nofs	nofs
chr03	B	3q28	.	191000000	3044	nofs	nofs
chr03	B	3q28	.	191000000	3246	nofs	nofs
chr03	B	3q28	.	191000000	3622	nofs	nofs
chr03	B	3q28	.	192000000	2771	nofs	nofs
chr03	B	3q28	.	192000000	2250	nofs	nofs
chr03	B	3q28	.	192000000	3209	nofs	nofs
chr03	B	3q28	.	192000000	3710	nofs	nofs
chr03	B	3q28	.	193000000	1957	nofs	nofs
chr03	B	3q28	.	193000000	2355	nofs	nofs
chr03	B	3q28	.	193000000	2679	nofs	nofs
chr03	B	3q28	.	193000000	2464	nofs	nofs
chr03	B	3q28	.	194000000	1259	nofs	nofs
chr03	B	3q28	.	194000000	2462	nofs	nofs
chr03	E	3q29	4	194000000	4375	nofs	nofs

chr03	E	3q29	4	194000000	1845	nofs	nofs
chr03	E	3q29	4	195000000	2767	nofs	nofs
chr03	E	3q29	4	195000000	2837	nofs	nofs
chr03	E	3q29	4	195000000	2506	nofs	nofs
chr03	E	3q29	4	195000000	4433	nofs	nofs
chr03	E	3q29	4	196000000	4658	nofs	nofs
chr03	E	3q29	4	196000000	5952	nofs	nofs
chr03	T	3q29	4	196000000	5893	nofs	nofs
chr03	T	3q29	4	196000000	6911	nofs	nofs
chr03	T	3q29	4	197000000	36942	nofs	nofs
chr03	T	3q29	4	197000000	20737	nofs	nofs
chr03	T	3q29	4	197000000	32198	nofs	nofs
chr03	T	3q29	4	197000000	12721	nofs	nofs
chr03	T	3q29	4	198000000	10147	nofs	nofs
chr03	T	3q29	4	198000000	6134	nofs	nofs
chr03	T	3q29	4	198000000	7759	nofs	nofs
chr03	T	3q29	4	198000000	2821	nofs	nofs
chr03	T	3q29	4	199000000	18041	nofs	nofs
chr03	T	3q29	4	199000000	21931	nofs	nofs
chr03	T	3q29	4	199000000	17899	nofs	nofs
chr04	T	4p16	6	125000	7096	nofs	nofs
chr04	T	4p16	6	375000	9702	nofs	nofs
chr04	T	4p16	6	625000	32439	nofs	nofs
chr04	T	4p16	6	875000	21068	nofs	nofs
chr04	T	4p16	6	1125000	21385	nofs	nofs
chr04	T	4p16	6	1375000	27743	nofs	nofs
chr04	T	4p16	6	1625000	18083	nofs	nofs
chr04	T	4p16	6	1875000	4056	nofs	nofs
chr04	T	4p16	6	2125000	2886	nofs	nofs
chr04	T	4p16	6	2375000	11314	nofs	nofs
chr04	T	4p16	6	2625000	1378	nofs	nofs
chr04	T	4p16	6	2875000	5849	nofs	nofs
chr04	T	4p16	6	3125000	3588	nofs	nofs
chr04	E	4p16	6	3375000	8923	nofs	nofs
chr04	E	4p16	6	3625000	24231	nofs	nofs
chr04	E	4p16	6	3875000	10590	nofs	nofs

chr04	E	4p16	6	4125000	5684	nofs	nofs
chr04	E	4p16	6	4375000	3823	nofs	nofs
chr04	E	4p16	6	4625000	2380	nofs	nofs
chr04	E	4p16	6	4875000	3754	nofs	nofs
chr04	E	4p16	6	5125000	3793	nofs	nofs
chr04	E	4p16	6	5375000	4670	nofs	nofs
chr04	E	4p16	6	5625000	2369	nofs	nofs
chr04	E	4p16	6	5875000	6071	nofs	nofs
chr04	E	4p16	6	6125000	7739	fs	fs common
chr04	E	4p16	6	6375000	6569	fs	fs common
chr04	E	4p16	6	6625000	6426	fs	fs common
chr04	E	4p16	6	6875000	4603	fs	fs common
chr04	E	4p16	6	7125000	7007	fs	fs common
chr04	E	4p16	6	7375000	10220	fs	fs common
chr04	E	4p16	6	7625000	19569	fs	fs common
chr04	E	4p16	6	7875000	14115	fs	fs common
chr04	E	4p16	6	8125000	8958	fs	fs common
chr04	E	4p16	6	8375000	11027	fs	fs common
chr04	E	4p16	6	8625000	7215	fs	fs common
chr04	E	4p16	6	8875000	2927	fs	fs common
chr04	E	4p16	6	9125000	6056	fs	fs common
chr04	E	4p16	6	9375000	4535	fs	fs common
chr04	E	4p16	6	9625000	3357	fs	fs common
chr04	E	4p16	6	9875000	1708	fs	fs common
chr04	E	4p16	6	10125000	3105	fs	fs common
chr04	E	4p16	6	10375000	2396	fs	fs common
chr04	E	4p16	6	10625000	3616	fs	fs common
chr04	E	4p16	6	10875000	4195	fs	fs common
chr04	E	4p15.3	2	11125000	2098	fs	fs common
chr04	E	4p15.3	2	11375000	2477	fs	fs common
chr04	E	4p15.3	2	11625000	2750	fs	fs common
chr04	E	4p15.3	2	11875000	15645	fs	fs common
chr04	E	4p15.3	2	12125000	2888	fs	fs common
chr04	E	4p15.3	2	12375000	7040	fs	fs common
chr04	E	4p15.3	2	12625000	2883	fs	fs common
chr04	E	4p15.3	2	12875000	2591	fs	fs common

chr04	E	4p15.3	2	13125000	1454	fs	fs common
chr04	E	4p15.3	2	13375000	2927	fs	fs common
chr04	E	4p15.3	2	13625000	2031	fs	fs common
chr04	E	4p15.3	2	13875000	2621	fs	fs common
chr04	E	4p15.3	2	14125000	2228	fs	fs common
chr04	E	4p15.3	2	14375000	2940	fs	fs common
chr04	E	4p15.3	2	14625000	5721	fs	fs common
chr04	E	4p15.3	2	14875000	2165	fs	fs common
chr04	E	4p15.3	2	15125000	1974	fs	fs common
chr04	E	4p15.3	2	15375000	4825	fs	fs common
chr04	E	4p15.3	2	15625000	2772	fs	fs common
chr04	E	4p15.3	2	15875000	2577	fs	fs common
chr04	E	4p15.3	2	16125000	3121	fs	fs common
chr04	E	4p15.3	2	16375000	2495	fs	fs common
chr04	E	4p15.3	2	16625000	2821	fs	fs common
chr04	E	4p15.3	2	16875000	1486	fs	fs common
chr04	E	4p15.3	2	17125000	4436	fs	fs common
chr04	E	4p15.3	2	17375000	3033	fs	fs common
chr04	E	4p15.3	2	17625000	1792	fs	fs common
chr04	E	4p15.3	2	17875000	1679	fs	fs common
chr04	E	4p15.3	2	18125000	3114	fs	fs common
chr04	E	4p15.3	2	18375000	5105	fs	fs common
chr04	E	4p15.3	2	18625000	1833	fs	fs common
chr04	E	4p15.3	2	18875000	3438	fs	fs common
chr04	E	4p15.3	2	19125000	2937	fs	fs common
chr04	E	4p15.3	2	19375000	2542	fs	fs common
chr04	E	4p15.3	2	19625000	2013	fs	fs common
chr04	E	4p15.3	2	19875000	4069	fs	fs common
chr04	E	4p15.3	2	20125000	2231	fs	fs common
chr04	E	4p15.3	2	20375000	2392	fs	fs common
chr04	E	4p15.3	2	20625000	3018	fs	fs common
chr04	E	4p15.3	2	20875000	4006	fs	fs common
chr04	E	4p15.3	2	21125000	3464	fs	fs common
chr04	E	4p15.3	2	21375000	3562	fs	fs common
chr04	E	4p15.3	2	21625000	2704	fs	fs common
chr04	E	4p15.3	2	21875000	1879	fs	fs common

chr04	E	4p15.3	2	22125000	1897	fs	fs common
chr04	E	4p15.3	2	22375000	4337	fs	fs common
chr04	E	4p15.3	2	22625000	1770	fs	fs common
chr04	E	4p15.3	2	22875000	2838	fs	fs common
chr04	B	4p15.2	.	23125000	1922	fs	fs common
chr04	B	4p15.2	.	23375000	2243	fs	fs common
chr04	B	4p15.2	.	23625000	2965	fs	fs common
chr04	B	4p15.2	.	23875000	3069	fs	fs common
chr04	B	4p15.2	.	24125000	3360	fs	fs common
chr04	B	4p15.2	.	24375000	3203	fs	fs common
chr04	B	4p15.2	.	24625000	3010	fs	fs common
chr04	B	4p15.2	.	24875000	3169	fs	fs common
chr04	B	4p15.2	.	25125000	3043	fs	fs common
chr04	B	4p15.2	.	25375000	3754	fs	fs common
chr04	B	4p15.2	.	25625000	3000	fs	fs common
chr04	B	4p15.2	.	25875000	5131	fs	fs common
chr04	B	4p15.2	.	26125000	3415	fs	fs common
chr04	B	4p15.2	.	26375000	1774	fs	fs common
chr04	B	4p15.2	.	26625000	2186	fs	fs common
chr04	B	4p15.2	.	26875000	2231	fs	fs common
chr04	B	4p15.2	.	27125000	2349	fs	fs common
chr04	B	4p15.2	.	27375000	2276	fs	fs common
chr04	B	4p15.2	.	27625000	2340	fs	fs common
chr04	B	4p15.2	.	27875000	4354	fs	fs common
chr04	B	4p15.1	.	28125000	3160	fs	fs common
chr04	B	4p15.1	.	28375000	2512	fs	fs common
chr04	B	4p15.1	.	28625000	3865	fs	fs common
chr04	B	4p15.1	.	28875000	3369	fs	fs common
chr04	B	4p15.1	.	29125000	2852	fs	fs common
chr04	B	4p15.1	.	29375000	2218	fs	fs common
chr04	B	4p15.1	.	29625000	2661	fs	fs common
chr04	B	4p15.1	.	29875000	3372	fs	fs common
chr04	B	4p15.1	.	30125000	3031	fs	fs common
chr04	B	4p15.1	.	30375000	1653	fs	fs common
chr04	B	4p15.1	.	30625000	2876	fs	fs common
chr04	B	4p15.1	.	30875000	2964	fs	fs common

chr04	B	4p15.1	.	31125000	2227	fs	fs common
chr04	B	4p15.1	.	31375000	3108	fs	fs common
chr04	B	4p15.1	.	31625000	1859	fs	fs common
chr04	B	4p15.1	.	31875000	3334	fs	fs common
chr04	B	4p15.1	.	32125000	3064	fs	fs common
chr04	B	4p15.1	.	32375000	1955	fs	fs common
chr04	B	4p15.1	.	32625000	2280	fs	fs common
chr04	B	4p15.1	.	32875000	4961	fs	fs common
chr04	B	4p15.1	.	33125000	2918	fs	fs common
chr04	B	4p15.1	.	33375000	1914	fs	fs common
chr04	B	4p15.1	.	33625000	2385	fs	fs common
chr04	B	4p15.1	.	33875000	1594	fs	fs common
chr04	B	4p15.1	.	34125000	2349	fs	fs common
chr04	B	4p15.1	.	34375000	2762	fs	fs common
chr04	B	4p15.1	.	34625000	2335	fs	fs common
chr04	B	4p15.1	.	34875000	2591	fs	fs common
chr04	B	4p15.1	.	35125000	3937	fs	fs common
chr04	B	4p15.1	.	35375000	2382	fs	fs common
chr04	B	4p14	.	35625000	2423	nofs	nofs
chr04	B	4p14	.	35875000	1897	nofs	nofs
chr04	B	4p14	.	36125000	1940	nofs	nofs
chr04	B	4p14	.	36375000	2089	nofs	nofs
chr04	B	4p14	.	36625000	2133	nofs	nofs
chr04	B	4p14	.	36875000	3483	nofs	nofs
chr04	B	4p14	.	37125000	3169	nofs	nofs
chr04	B	4p14	.	37375000	4317	nofs	nofs
chr04	B	4p14	.	37625000	4143	nofs	nofs
chr04	B	4p14	.	37875000	1827	nofs	nofs
chr04	B	4p14	.	38125000	3895	nofs	nofs
chr04	B	4p14	.	38375000	2105	nofs	nofs
chr04	B	4p14	.	38625000	5583	nofs	nofs
chr04	B	4p14	.	38875000	2795	nofs	nofs
chr04	B	4p14	.	39125000	2451	nofs	nofs
chr04	B	4p14	.	39375000	4761	nofs	nofs
chr04	B	4p14	.	39625000	5816	nofs	nofs
chr04	B	4p14	.	39875000	6383	nofs	nofs

chr04	B	4p14	.	40125000	3193	nofs	nofs
chr04	B	4p14	.	40375000	4484	nofs	nofs
chr04	B	4p14	.	40625000	3292	nofs	nofs
chr04	B	4p14	.	40875000	3337	nofs	nofs
chr04	E	4p13	1	41125000	2411	nofs	nofs
chr04	E	4p13	1	41375000	4279	nofs	nofs
chr04	E	4p13	1	41625000	1700	nofs	nofs
chr04	E	4p13	1	41875000	1846	nofs	nofs
chr04	E	4p13	1	42125000	5423	nofs	nofs
chr04	E	4p13	1	42375000	1764	nofs	nofs
chr04	E	4p13	1	42625000	2265	nofs	nofs
chr04	E	4p13	1	42875000	1247	nofs	nofs
chr04	E	4p13	1	43125000	2929	nofs	nofs
chr04	E	4p13	1	43375000	4698	nofs	nofs
chr04	E	4p13	1	43625000	3406	nofs	nofs
chr04	E	4p13	1	43875000	3027	nofs	nofs
chr04	E	4p13	1	44125000	3300	nofs	nofs
chr04	E	4p13	1	44375000	1507	nofs	nofs
chr04	E	4p13	1	44625000	1504	nofs	nofs
chr04	E	4p13	1	44875000	5117	nofs	nofs
chr04	E	4p13	1	45125000	3942	nofs	nofs
chr04	E	4p13	1	45375000	3012	nofs	nofs
chr04	E	4p13	1	45625000	2537	nofs	nofs
chr04	E	4p13	1	45875000	3487	nofs	nofs
chr04	E	4p12	1	46125000	3141	nofs	nofs
chr04	C	4p12	1	46375000	1490	nofs	nofs
chr04	C	4p12	1	46625000	3696	nofs	nofs
chr04	C	4p12	1	46875000	3277	nofs	nofs
chr04	C	4p12	1	47125000	3170	nofs	nofs
chr04	C	4p12	1	47375000	1830	nofs	nofs
chr04	C	4p12	1	47625000	2690	nofs	nofs
chr04	C	4p12	1	47875000	3464	nofs	nofs
chr04	C	4p12	1	48125000	4439	nofs	nofs
chr04	C	4p11	.	48375000	3655	nofs	nofs
chr04	C	4p11	.	48625000	3524	nofs	nofs
chr04	C	4p11	.	48875000	72719	nofs	nofs

chr04	C	4p11	.	49125000	24652	nofs	nofs
chr04	C	4p11	.	49375000	38786	nofs	nofs
chr04	C		.	49625000	1013	nofs	nofs
chr04	C		.	49875000	0	nofs	nofs
chr04	C		.	50125000	0	nofs	nofs
chr04	C		.	50375000	0	nofs	nofs
chr04	C		.	50625000	0	nofs	nofs
chr04	C		.	50875000	0	nofs	nofs
chr04	C		.	51125000	0	nofs	nofs
chr04	C		.	51375000	0	nofs	nofs
chr04	C		.	51625000	0	nofs	nofs
chr04	C		.	51875000	0	nofs	nofs
chr04	C		.	52125000	0	nofs	nofs
chr04	C		.	52375000	0	nofs	nofs
chr04	C	4q11	.	52625000	27949	nofs	nofs
chr04	C	4q11	.	52875000	2236	nofs	nofs
chr04	C	4q11	.	53125000	4690	nofs	nofs
chr04	C	4q11	.	53375000	3029	nofs	nofs
chr04	C	4q11	.	53625000	2286	fs	fs common
chr04	C	4q11	.	53875000	4608	fs	fs common
chr04	C	4q12	3	54125000	2376	fs	fs common
chr04	C	4q12	3	54375000	1572	fs	fs common
chr04	C	4q12	3	54625000	2852	fs	fs common
chr04	C	4q12	3	54875000	4408	fs	fs common
chr04	C	4q12	3	55125000	3184	fs	fs common
chr04	C	4q12	3	55375000	2102	fs	fs common
chr04	C	4q12	3	55625000	1942	fs	fs common
chr04	E	4q12	3	55875000	2380	fs	fs common
chr04	E	4q12	3	56125000	1957	fs	fs common
chr04	E	4q12	3	56375000	3355	fs	fs common
chr04	E	4q12	3	56625000	3014	fs	fs common
chr04	E	4q12	3	56875000	3151	fs	fs common
chr04	E	4q12	3	57125000	1504	fs	fs common
chr04	E	4q12	3	57375000	4023	fs	fs common
chr04	E	4q12	3	57625000	4272	fs	fs common
chr04	E	4q12	3	57875000	2028	fs	fs common

chr04	E	4q12	3	58125000	2904	fs	fs common
chr04	E	4q12	3	58375000	2177	fs	fs common
chr04	E	4q12	3	58625000	5854	fs	fs common
chr04	E	4q12	3	58875000	2694	fs	fs common
chr04	E	4q12	3	59125000	3024	fs	fs common
chr04	E	4q12	3	59375000	3146	fs	fs common
chr04	E	4q12	3	59625000	2309	fs	fs common
chr04	B	4q13.1	.	59875000	2698	nofs	nofs
chr04	B	4q13.1	.	60125000	3118	nofs	nofs
chr04	B	4q13.1	.	60375000	1881	nofs	nofs
chr04	B	4q13.1	.	60625000	3135	nofs	nofs
chr04	B	4q13.1	.	60875000	3729	nofs	nofs
chr04	B	4q13.1	.	61125000	2824	nofs	nofs
chr04	B	4q13.1	.	61375000	3151	nofs	nofs
chr04	B	4q13.1	.	61625000	2338	nofs	nofs
chr04	B	4q13.1	.	61875000	1994	nofs	nofs
chr04	B	4q13.1	.	62125000	2741	nofs	nofs
chr04	B	4q13.1	.	62375000	3369	nofs	nofs
chr04	B	4q13.1	.	62625000	3346	nofs	nofs
chr04	B	4q13.1	.	62875000	2018	nofs	nofs
chr04	B	4q13.1	.	63125000	2260	nofs	nofs
chr04	B	4q13.1	.	63375000	4162	nofs	nofs
chr04	B	4q13.1	.	63625000	3085	nofs	nofs
chr04	B	4q13.1	.	63875000	5321	nofs	nofs
chr04	B	4q13.1	.	64125000	15196	nofs	nofs
chr04	B	4q13.1	.	64375000	1748	nofs	nofs
chr04	B	4q13.1	.	64625000	4645	nofs	nofs
chr04	B	4q13.1	.	64875000	2260	nofs	nofs
chr04	B	4q13.1	.	65125000	1678	nofs	nofs
chr04	B	4q13.1	.	65375000	2153	nofs	nofs
chr04	B	4q13.1	.	65625000	3450	nofs	nofs
chr04	B	4q13.1	.	65875000	2651	nofs	nofs
chr04	B	4q13.1	.	66125000	2986	nofs	nofs
chr04	B	4q13.1	.	66375000	2231	nofs	nofs
chr04	B	4q13.1	.	66625000	1614	nofs	nofs
chr04	B	4q13.1	.	66875000	1791	nofs	nofs

chr04	B	4q13.2	.	67125000	2109	nofs	nofs
chr04	B	4q13.2	.	67375000	3124	nofs	nofs
chr04	B	4q13.2	.	67625000	2933	nofs	nofs
chr04	B	4q13.2	.	67875000	3198	nofs	nofs
chr04	B	4q13.2	.	68125000	6077	nofs	nofs
chr04	B	4q13.2	.	68375000	2780	nofs	nofs
chr04	B	4q13.2	.	68625000	1483	nofs	nofs
chr04	B	4q13.2	.	68875000	1936	nofs	nofs
chr04	B	4q13.2	.	69125000	1660	nofs	nofs
chr04	B	4q13.2	.	69375000	2123	nofs	nofs
chr04	B	4q13.2	.	69625000	1769	nofs	nofs
chr04	B	4q13.2	.	69875000	2228	nofs	nofs
chr04	B	4q13.2	.	70125000	1788	nofs	nofs
chr04	E	4q13.3	1	70375000	2009	nofs	nofs
chr04	E	4q13.3	1	70625000	3630	nofs	nofs
chr04	E	4q13.3	1	70875000	2739	nofs	nofs
chr04	E	4q13.3	1	71125000	3172	nofs	nofs
chr04	E	4q13.3	1	71375000	1814	nofs	nofs
chr04	E	4q13.3	1	71625000	5831	nofs	nofs
chr04	E	4q13.3	1	71875000	1814	nofs	nofs
chr04	E	4q13.3	1	72125000	2249	nofs	nofs
chr04	E	4q13.3	1	72375000	2900	nofs	nofs
chr04	E	4q13.3	1	72625000	1512	nofs	nofs
chr04	E	4q13.3	1	72875000	1737	nofs	nofs
chr04	E	4q13.3	1	73125000	2611	nofs	nofs
chr04	E	4q13.3	1	73375000	1804	nofs	nofs
chr04	E	4q13.3	1	73625000	3636	nofs	nofs
chr04	E	4q13.3	1	73875000	2852	nofs	nofs
chr04	E	4q13.3	1	74125000	1594	nofs	nofs
chr04	E	4q13.3	1	74375000	2171	nofs	nofs
chr04	E	4q13.3	1	74625000	2849	nofs	nofs
chr04	E	4q13.3	1	74875000	3145	nofs	nofs
chr04	E	4q13.3	1	75125000	1935	nofs	nofs
chr04	E	4q13.3	1	75375000	2021	nofs	nofs
chr04	E	4q13.3	1	75625000	2506	nofs	nofs
chr04	E	4q13.3	1	75875000	1407	nofs	nofs

chr04	E	4q13.3	1	76125000	1471	nofs	nofs
chr04	E	4q13.3	1	76375000	2251	nofs	nofs
chr04	E	4q13.3	1	76625000	1252	nofs	nofs
chr04	E	4q21.1	3	76875000	2113	nofs	nofs
chr04	E	4q21.1	3	77125000	3119	nofs	nofs
chr04	E	4q21.1	3	77375000	3768	nofs	nofs
chr04	E	4q21.1	3	77625000	1864	nofs	nofs
chr04	E	4q21.1	3	77875000	1936	nofs	nofs
chr04	E	4q21.1	3	78125000	3490	nofs	nofs
chr04	E	4q21.1	3	78375000	2297	nofs	nofs
chr04	E	4q21.1	3	78625000	1485	nofs	nofs
chr04	E	4q21.1	3	78875000	1978	nofs	nofs
chr04	E	4q21.1	3	79125000	2483	nofs	nofs
chr04	E	4q21.2	2	79375000	3327	nofs	nofs
chr04	E	4q21.2	2	79625000	1462	nofs	nofs
chr04	E	4q21.2	2	79875000	2953	nofs	nofs
chr04	E	4q21.2	2	80125000	2602	nofs	nofs
chr04	E	4q21.2	2	80375000	1929	nofs	nofs
chr04	E	4q21.2	2	80625000	4415	nofs	nofs
chr04	E	4q21.2	2	80875000	2166	nofs	nofs
chr04	E	4q21.2	2	81125000	1957	nofs	nofs
chr04	E	4q21.2	2	81375000	2612	nofs	nofs
chr04	E	4q21.2	2	81625000	2458	nofs	nofs
chr04	E	4q21.2	2	81875000	1717	nofs	nofs
chr04	E	4q21.2	2	82125000	4113	nofs	nofs
chr04	E	4q21.2	2	82375000	1512	nofs	nofs
chr04	E	4q21.2	2	82625000	1952	nofs	nofs
chr04	E	4q21.2	2	82875000	3155	nofs	nofs
chr04	E	4q21.2	2	83125000	2775	nofs	nofs
chr04	E	4q21.2	2	83375000	2212	nofs	nofs
chr04	E	4q21.2	2	83625000	4206	nofs	nofs
chr04	E	4q21.2	2	83875000	3247	nofs	nofs
chr04	E	4q21.2	2	84125000	2517	nofs	nofs
chr04	E	4q21.2	2	84375000	5667	nofs	nofs
chr04	E	4q21.2	2	84625000	2082	nofs	nofs
chr04	E	4q21.2	2	84875000	5364	nofs	nofs

chr04	E	4q21.2	2	85125000	3056	nofs	nofs
chr04	E	4q21.2	2	85375000	1108	nofs	nofs
chr04	E	4q21.2	2	85625000	1046	nofs	nofs
chr04	E	4q21.2	2	85875000	2286	nofs	nofs
chr04	E	4q21.2	2	86125000	1776	nofs	nofs
chr04	E	4q21.2	2	86375000	1971	nofs	nofs
chr04	E	4q21.2	2	86625000	1999	nofs	nofs
chr04	E	4q21.2	2	86875000	3650	nofs	nofs
chr04	E	4q21.2	2	87125000	3196	nofs	nofs
chr04	E	4q21.2	2	87375000	1681	nofs	nofs
chr04	B	4q21.3	.	87625000	2674	nofs	nofs
chr04	B	4q21.3	.	87875000	2672	nofs	nofs
chr04	B	4q21.3	.	88125000	2898	nofs	nofs
chr04	B	4q21.3	.	88375000	3107	nofs	nofs
chr04	E	4q22	4	88625000	2824	fs	fs common
chr04	E	4q22	4	88875000	5277	fs	fs common
chr04	E	4q22	4	89125000	2277	fs	fs common
chr04	E	4q22	4	89375000	2742	fs	fs common
chr04	E	4q22	4	89625000	5143	fs	fs common
chr04	E	4q22	4	89875000	2695	fs	fs common
chr04	E	4q22	4	90125000	2417	fs	fs common
chr04	E	4q22	4	90375000	2628	fs	fs common
chr04	E	4q22	4	90625000	1560	fs	fs common
chr04	E	4q22	4	90875000	2788	fs	fs common
chr04	E	4q22	4	91125000	1516	fs	fs common
chr04	E	4q22	4	91375000	1351	fs	fs common
chr04	E	4q22	4	91625000	2443	fs	fs common
chr04	E	4q22	4	91875000	1828	fs	fs common
chr04	E	4q22	4	92125000	3023	fs	fs common
chr04	E	4q22	4	92375000	2462	fs	fs common
chr04	E	4q22	4	92625000	1640	fs	fs common
chr04	E	4q22	4	92875000	2867	fs	fs common
chr04	E	4q22	4	93125000	2541	fs	fs common
chr04	E	4q22	4	93375000	2655	fs	fs common
chr04	E	4q22	4	93625000	2878	fs	fs common
chr04	E	4q22	4	93875000	2671	fs	fs common

chr04	E	4q22	4	94125000	1319	fs	fs common
chr04	E	4q22	4	94375000	2387	fs	fs common
chr04	E	4q22	4	94625000	3282	fs	fs common
chr04	E	4q22	4	94875000	1282	fs	fs common
chr04	E	4q22	4	95125000	2609	fs	fs common
chr04	E	4q22	4	95375000	3170	fs	fs common
chr04	E	4q22	4	95625000	2222	fs	fs common
chr04	E	4q22	4	95875000	2192	fs	fs common
chr04	E	4q22	4	96125000	1864	fs	fs common
chr04	E	4q22	4	96375000	4507	fs	fs common
chr04	E	4q22	4	96625000	1216	fs	fs common
chr04	E	4q22	4	96875000	1338	fs	fs common
chr04	E	4q22	4	97125000	1663	fs	fs common
chr04	E	4q22	4	97375000	2238	fs	fs common
chr04	E	4q22	4	97625000	2027	fs	fs common
chr04	E	4q22	4	97875000	2534	fs	fs common
chr04	E	4q22	4	98125000	2310	fs	fs common
chr04	E	4q22	4	98375000	2102	fs	fs common
chr04	E	4q22	4	98625000	3274	fs	fs common
chr04	E	4q22	4	98875000	2432	fs	fs common
chr04	E	4q22	4	99125000	2304	fs	fs common
chr04	E	4q22	4	99375000	2266	fs	fs common
chr04	B	4q23	.	99625000	2593	nofs	nofs
chr04	B	4q23	.	99875000	1053	nofs	nofs
chr04	B	4q23	.	100000000	2761	nofs	nofs
chr04	B	4q23	.	100000000	2631	nofs	nofs
chr04	B	4q23	.	101000000	3130	nofs	nofs
chr04	B	4q23	.	101000000	1894	nofs	nofs
chr04	B	4q23	.	101000000	2335	nofs	nofs
chr04	B	4q23	.	101000000	3340	nofs	nofs
chr04	B	4q24	.	102000000	2067	nofs	nofs
chr04	B	4q24	.	102000000	3176	nofs	nofs
chr04	B	4q24	.	102000000	2423	nofs	nofs
chr04	B	4q24	.	102000000	2807	nofs	nofs
chr04	B	4q24	.	103000000	2845	nofs	nofs
chr04	B	4q24	.	103000000	1914	nofs	nofs

chr04	B	4q24	.	103000000	3315	nofs	nofs
chr04	B	4q24	.	103000000	4053	nofs	nofs
chr04	B	4q24	.	104000000	6337	nofs	nofs
chr04	B	4q24	.	104000000	2814	nofs	nofs
chr04	B	4q24	.	104000000	1744	nofs	nofs
chr04	B	4q24	.	104000000	2605	nofs	nofs
chr04	B	4q24	.	105000000	2389	nofs	nofs
chr04	B	4q24	.	105000000	2914	nofs	nofs
chr04	B	4q24	.	105000000	4146	nofs	nofs
chr04	B	4q24	.	105000000	2419	nofs	nofs
chr04	B	4q24	.	106000000	1592	nofs	nofs
chr04	B	4q24	.	106000000	2282	nofs	nofs
chr04	B	4q24	.	106000000	2022	nofs	nofs
chr04	B	4q24	.	106000000	1897	nofs	nofs
chr04	B	4q24	.	107000000	2100	nofs	nofs
chr04	B	4q24	.	107000000	1945	nofs	nofs
chr04	B	4q24	.	107000000	1822	nofs	nofs
chr04	B	4q24	.	107000000	2421	nofs	nofs
chr04	B	4q24	.	108000000	2637	nofs	nofs
chr04	B	4q24	.	108000000	2213	nofs	nofs
chr04	B	4q24	.	108000000	1948	nofs	nofs
chr04	B	4q25	.	108000000	2521	nofs	nofs
chr04	B	4q25	.	109000000	3682	nofs	nofs
chr04	B	4q25	.	109000000	1914	nofs	nofs
chr04	B	4q25	.	109000000	1805	nofs	nofs
chr04	B	4q25	.	109000000	2636	nofs	nofs
chr04	B	4q25	.	110000000	3121	nofs	nofs
chr04	B	4q25	.	110000000	3271	nofs	nofs
chr04	B	4q25	.	110000000	3851	nofs	nofs
chr04	B	4q25	.	110000000	2748	nofs	nofs
chr04	B	4q25	.	111000000	2128	nofs	nofs
chr04	B	4q25	.	111000000	4239	nofs	nofs
chr04	B	4q25	.	111000000	3958	nofs	nofs
chr04	B	4q25	.	111000000	2530	nofs	nofs
chr04	B	4q25	.	112000000	3868	nofs	nofs
chr04	B	4q25	.	112000000	1535	nofs	nofs

chr04	B	4q25	.	112000000	2847	nofs	nofs
chr04	B	4q25	.	112000000	2291	nofs	nofs
chr04	B	4q25	.	113000000	1703	nofs	nofs
chr04	B	4q25	.	113000000	2291	nofs	nofs
chr04	B	4q25	.	113000000	2739	nofs	nofs
chr04	B	4q25	.	113000000	1998	nofs	nofs
chr04	B	4q25	.	114000000	1678	nofs	nofs
chr04	B	4q25	.	114000000	2895	nofs	nofs
chr04	B	4q25	.	114000000	3356	nofs	nofs
chr04	B	4q25	.	114000000	2326	nofs	nofs
chr04	E	4q26	3	115000000	2709	nofs	nofs
chr04	E	4q26	3	115000000	1643	nofs	nofs
chr04	E	4q26	3	115000000	2014	nofs	nofs
chr04	E	4q26	3	115000000	3209	nofs	nofs
chr04	E	4q26	3	116000000	2549	nofs	nofs
chr04	E	4q26	3	116000000	3836	nofs	nofs
chr04	E	4q26	3	116000000	2433	nofs	nofs
chr04	E	4q26	3	116000000	3158	nofs	nofs
chr04	E	4q26	3	117000000	2423	nofs	nofs
chr04	E	4q26	3	117000000	2502	nofs	nofs
chr04	E	4q26	3	117000000	3117	nofs	nofs
chr04	E	4q26	3	117000000	2067	nofs	nofs
chr04	E	4q26	3	118000000	2072	nofs	nofs
chr04	E	4q26	3	118000000	2543	nofs	nofs
chr04	E	4q26	3	118000000	2017	nofs	nofs
chr04	E	4q26	3	118000000	2979	nofs	nofs
chr04	E	4q26	3	119000000	2307	nofs	nofs
chr04	E	4q26	3	119000000	2024	nofs	nofs
chr04	E	4q26	3	119000000	2674	nofs	nofs
chr04	E	4q26	3	119000000	2508	nofs	nofs
chr04	E	4q26	3	120000000	2574	nofs	nofs
chr04	E	4q26	3	120000000	3633	nofs	nofs
chr04	E	4q26	3	120000000	2624	nofs	nofs
chr04	E	4q26	3	120000000	2317	nofs	nofs
chr04	E	4q26	3	121000000	2507	nofs	nofs
chr04	E	4q26	3	121000000	1236	nofs	nofs

chr04	E	4q26	3	121000000	1578	nofs	nofs
chr04	E	4q27	4	121000000	3040	nofs	nofs
chr04	E	4q27	4	122000000	2178	nofs	nofs
chr04	E	4q27	4	122000000	2076	nofs	nofs
chr04	E	4q27	4	122000000	1959	nofs	nofs
chr04	E	4q27	4	122000000	2214	nofs	nofs
chr04	E	4q27	4	123000000	2902	nofs	nofs
chr04	E	4q27	4	123000000	3187	nofs	nofs
chr04	E	4q27	4	123000000	1723	nofs	nofs
chr04	E	4q27	4	123000000	1626	nofs	nofs
chr04	E	4q27	4	124000000	1796	nofs	nofs
chr04	E	4q27	4	124000000	1976	nofs	nofs
chr04	E	4q27	4	124000000	4083	nofs	nofs
chr04	E	4q27	4	124000000	4416	nofs	nofs
chr04	E	4q28	1	125000000	2077	nofs	nofs
chr04	E	4q28	1	125000000	4038	nofs	nofs
chr04	E	4q28	1	125000000	1586	nofs	nofs
chr04	E	4q28	1	125000000	1356	nofs	nofs
chr04	E	4q28	1	126000000	2041	nofs	nofs
chr04	E	4q28	1	126000000	1983	nofs	nofs
chr04	E	4q28	1	126000000	3023	nofs	nofs
chr04	E	4q28	1	126000000	3239	nofs	nofs
chr04	E	4q28	1	127000000	2183	nofs	nofs
chr04	E	4q28	1	127000000	2266	nofs	nofs
chr04	E	4q28	1	127000000	3120	nofs	nofs
chr04	E	4q28	1	127000000	2354	nofs	nofs
chr04	E	4q28	1	128000000	2250	nofs	nofs
chr04	E	4q28	1	128000000	2488	nofs	nofs
chr04	E	4q28	1	128000000	4682	nofs	nofs
chr04	E	4q28	1	128000000	1814	nofs	nofs
chr04	E	4q28	1	129000000	2883	nofs	nofs
chr04	E	4q28	1	129000000	1881	nofs	nofs
chr04	E	4q28	1	129000000	3160	nofs	nofs
chr04	E	4q28	1	129000000	3713	nofs	nofs
chr04	E	4q28	1	130000000	1778	nofs	nofs
chr04	E	4q28	1	130000000	3115	nofs	nofs

chr04	E	4q28	1	130000000	2868	nofs	nofs
chr04	E	4q28	1	130000000	1526	nofs	nofs
chr04	E	4q28	1	131000000	2051	nofs	nofs
chr04	E	4q28	1	131000000	1783	nofs	nofs
chr04	E	4q28	1	131000000	1579	nofs	nofs
chr04	E	4q28	1	131000000	3110	nofs	nofs
chr04	E	4q28	1	132000000	1707	nofs	nofs
chr04	E	4q28	1	132000000	2219	nofs	nofs
chr04	E	4q28	1	132000000	2693	nofs	nofs
chr04	E	4q28	1	132000000	3157	nofs	nofs
chr04	E	4q28	1	133000000	2708	nofs	nofs
chr04	E	4q28	1	133000000	1679	nofs	nofs
chr04	E	4q28	1	133000000	2633	nofs	nofs
chr04	E	4q28	1	133000000	2546	nofs	nofs
chr04	E	4q28	1	134000000	3469	nofs	nofs
chr04	E	4q28	1	134000000	2031	nofs	nofs
chr04	E	4q28	1	134000000	1682	nofs	nofs
chr04	E	4q28	1	134000000	2035	nofs	nofs
chr04	E	4q28	1	135000000	2447	nofs	nofs
chr04	E	4q28	1	135000000	2391	nofs	nofs
chr04	E	4q28	1	135000000	2562	nofs	nofs
chr04	E	4q28	1	135000000	1836	nofs	nofs
chr04	E	4q28	1	136000000	1559	nofs	nofs
chr04	E	4q28	1	136000000	5125	nofs	nofs
chr04	E	4q28	1	136000000	2760	nofs	nofs
chr04	E	4q28	1	136000000	3330	nofs	nofs
chr04	E	4q28	1	137000000	2060	nofs	nofs
chr04	E	4q28	1	137000000	2948	nofs	nofs
chr04	E	4q28	1	137000000	1764	nofs	nofs
chr04	E	4q28	1	137000000	2235	nofs	nofs
chr04	E	4q28	1	138000000	3535	nofs	nofs
chr04	E	4q28	1	138000000	2437	nofs	nofs
chr04	E	4q28	1	138000000	1810	nofs	nofs
chr04	E	4q28	1	138000000	3814	nofs	nofs
chr04	E	4q28	1	139000000	1547	nofs	nofs
chr04	E	4q28	1	139000000	1939	nofs	nofs

chr04	E	4q28	1	139000000	3117	nofs	nofs
chr04	E	4q28	1	139000000	2632	nofs	nofs
chr04	E	4q28	1	140000000	1675	nofs	nofs
chr04	E	4q28	1	140000000	2206	nofs	nofs
chr04	E	4q31.1	2	140000000	3703	fs	fs common
chr04	E	4q31.1	2	140000000	3660	fs	fs common
chr04	E	4q31.1	2	141000000	3147	fs	fs common
chr04	E	4q31.1	2	141000000	2547	fs	fs common
chr04	E	4q31.1	2	141000000	2386	fs	fs common
chr04	E	4q31.1	2	141000000	2623	fs	fs common
chr04	E	4q31.1	2	142000000	2394	fs	fs common
chr04	E	4q31.1	2	142000000	1532	fs	fs common
chr04	E	4q31.2	3	142000000	4385	nofs	nofs
chr04	E	4q31.2	3	142000000	3560	nofs	nofs
chr04	E	4q31.2	3	143000000	1970	nofs	nofs
chr04	E	4q31.2	3	143000000	3427	nofs	nofs
chr04	E	4q31.2	3	143000000	1965	nofs	nofs
chr04	E	4q31.2	3	143000000	1436	nofs	nofs
chr04	E	4q31.2	3	144000000	1957	nofs	nofs
chr04	E	4q31.2	3	144000000	2426	nofs	nofs
chr04	E	4q31.2	3	144000000	2393	nofs	nofs
chr04	E	4q31.2	3	144000000	1774	nofs	nofs
chr04	E	4q31.2	3	145000000	2191	nofs	nofs
chr04	E	4q31.2	3	145000000	1086	nofs	nofs
chr04	E	4q31.2	3	145000000	2788	nofs	nofs
chr04	E	4q31.2	3	145000000	1824	nofs	nofs
chr04	E	4q31.2	3	146000000	2780	nofs	nofs
chr04	E	4q31.2	3	146000000	1327	nofs	nofs
chr04	E	4q31.2	3	146000000	1983	nofs	nofs
chr04	E	4q31.2	3	146000000	3738	nofs	nofs
chr04	E	4q31.2	3	147000000	2122	nofs	nofs
chr04	E	4q31.2	3	147000000	2902	nofs	nofs
chr04	E	4q31.2	3	147000000	3773	nofs	nofs
chr04	E	4q31.2	3	147000000	2967	nofs	nofs
chr04	E	4q31.2	3	148000000	2703	nofs	nofs
chr04	E	4q31.2	3	148000000	2465	nofs	nofs

chr04	E	4q31.2	3	148000000	1866	nofs	nofs
chr04	E	4q31.2	3	148000000	2568	nofs	nofs
chr04	E	4q31.2	3	149000000	3344	nofs	nofs
chr04	E	4q31.2	3	149000000	3582	nofs	nofs
chr04	E	4q31.2	3	149000000	3937	nofs	nofs
chr04	E	4q31.2	3	149000000	1172	nofs	nofs
chr04	E	4q31.2	3	150000000	2041	nofs	nofs
chr04	E	4q31.2	3	150000000	5725	nofs	nofs
chr04	E	4q31.2	3	150000000	2943	nofs	nofs
chr04	E	4q31.2	3	150000000	1979	nofs	nofs
chr04	E	4q31.2	3	151000000	3002	nofs	nofs
chr04	E	4q31.2	3	151000000	1171	nofs	nofs
chr04	E	4q31.2	3	151000000	2519	nofs	nofs
chr04	E	4q31.2	3	151000000	2776	nofs	nofs
chr04	E	4q31.2	3	152000000	3699	nofs	nofs
chr04	E	4q31.2	3	152000000	4046	nofs	nofs
chr04	E	4q31.3	1	152000000	4986	nofs	nofs
chr04	E	4q31.3	1	152000000	4363	nofs	nofs
chr04	E	4q31.3	1	153000000	4237	nofs	nofs
chr04	E	4q31.3	1	153000000	2244	nofs	nofs
chr04	E	4q31.3	1	153000000	1832	nofs	nofs
chr04	E	4q31.3	1	153000000	3041	nofs	nofs
chr04	E	4q31.3	1	154000000	1165	nofs	nofs
chr04	E	4q31.3	1	154000000	3690	nofs	nofs
chr04	E	4q31.3	1	154000000	5928	nofs	nofs
chr04	E	4q31.3	1	154000000	5599	nofs	nofs
chr04	E	4q31.3	1	155000000	2944	nofs	nofs
chr04	E	4q31.3	1	155000000	3486	nofs	nofs
chr04	E	4q31.3	1	155000000	2625	nofs	nofs
chr04	E	4q31.3	1	155000000	2301	nofs	nofs
chr04	E	4q31.3	1	156000000	1983	nofs	nofs
chr04	E	4q31.3	1	156000000	2178	nofs	nofs
chr04	E	4q32	5	156000000	1712	nofs	nofs
chr04	E	4q32	5	156000000	2433	nofs	nofs
chr04	E	4q32	5	157000000	2598	nofs	nofs
chr04	E	4q32	5	157000000	3616	nofs	nofs

chr04	E	4q32	5	157000000	2292	nofs	nofs
chr04	E	4q32	5	157000000	2614	nofs	nofs
chr04	E	4q32	5	158000000	1905	nofs	nofs
chr04	E	4q32	5	158000000	1941	nofs	nofs
chr04	E	4q32	5	158000000	3574	nofs	nofs
chr04	E	4q32	5	158000000	1878	nofs	nofs
chr04	E	4q32	5	159000000	3262	nofs	nofs
chr04	E	4q32	5	159000000	2577	nofs	nofs
chr04	E	4q32	5	159000000	2284	nofs	nofs
chr04	E	4q32	5	159000000	4444	nofs	nofs
chr04	E	4q32	5	160000000	2126	nofs	nofs
chr04	E	4q32	5	160000000	3015	nofs	nofs
chr04	E	4q32	5	160000000	4026	nofs	nofs
chr04	E	4q32	5	160000000	2279	nofs	nofs
chr04	E	4q32	5	161000000	4462	nofs	nofs
chr04	E	4q32	5	161000000	2772	nofs	nofs
chr04	E	4q32	5	161000000	3635	nofs	nofs
chr04	E	4q32	5	161000000	3094	nofs	nofs
chr04	E	4q32	5	162000000	3267	nofs	nofs
chr04	E	4q32	5	162000000	3334	nofs	nofs
chr04	E	4q32	5	162000000	3243	nofs	nofs
chr04	E	4q32	5	162000000	2089	nofs	nofs
chr04	E	4q32	5	163000000	1992	nofs	nofs
chr04	E	4q32	5	163000000	2942	nofs	nofs
chr04	E	4q32	5	163000000	2983	nofs	nofs
chr04	E	4q32	5	163000000	2808	nofs	nofs
chr04	E	4q32	5	164000000	5392	nofs	nofs
chr04	E	4q32	5	164000000	3807	nofs	nofs
chr04	E	4q32	5	164000000	4121	nofs	nofs
chr04	E	4q32	5	164000000	3276	nofs	nofs
chr04	E	4q32	5	165000000	1995	nofs	nofs
chr04	E	4q32	5	165000000	1748	nofs	nofs
chr04	E	4q32	5	165000000	2955	nofs	nofs
chr04	E	4q32	5	165000000	3281	nofs	nofs
chr04	E	4q32	5	166000000	2373	nofs	nofs
chr04	E	4q32	5	166000000	1482	nofs	nofs

chr04	E	4q32	5	166000000	4969	nofs	nofs
chr04	E	4q32	5	166000000	4286	nofs	nofs
chr04	E	4q32	5	167000000	1993	nofs	nofs
chr04	E	4q32	5	167000000	2373	nofs	nofs
chr04	E	4q32	5	167000000	2158	nofs	nofs
chr04	E	4q32	5	167000000	1288	nofs	nofs
chr04	E	4q32	5	168000000	2242	nofs	nofs
chr04	E	4q32	5	168000000	2053	nofs	nofs
chr04	E	4q32	5	168000000	2518	nofs	nofs
chr04	E	4q32	5	168000000	2842	nofs	nofs
chr04	E	4q32	5	169000000	6196	nofs	nofs
chr04	E	4q32	5	169000000	2331	nofs	nofs
chr04	E	4q32	5	169000000	3176	nofs	nofs
chr04	E	4q32	5	169000000	2968	nofs	nofs
chr04	E	4q32	5	170000000	3361	nofs	nofs
chr04	E	4q32	5	170000000	3211	nofs	nofs
chr04	E	4q32	5	170000000	2618	nofs	nofs
chr04	E	4q32	5	170000000	3744	nofs	nofs
chr04	E	4q32	5	171000000	2092	nofs	nofs
chr04	E	4q33	1	171000000	3337	nofs	nofs
chr04	E	4q33	1	171000000	12281	nofs	nofs
chr04	E	4q33	1	171000000	3513	nofs	nofs
chr04	E	4q33	1	172000000	2307	nofs	nofs
chr04	E	4q33	1	172000000	2909	nofs	nofs
chr04	E	4q33	1	172000000	2449	nofs	nofs
chr04	E	4q33	1	172000000	2455	nofs	nofs
chr04	E	4q34	3	173000000	3092	nofs	nofs
chr04	E	4q34	3	173000000	3114	nofs	nofs
chr04	E	4q34	3	173000000	1954	nofs	nofs
chr04	E	4q34	3	173000000	2305	nofs	nofs
chr04	E	4q34	3	174000000	2762	nofs	nofs
chr04	E	4q34	3	174000000	2433	nofs	nofs
chr04	E	4q34	3	174000000	2118	nofs	nofs
chr04	E	4q34	3	174000000	3859	nofs	nofs
chr04	E	4q34	3	175000000	3119	nofs	nofs
chr04	E	4q34	3	175000000	4163	nofs	nofs

chr04	E	4q34	3	175000000	2976	nofs	nofs
chr04	E	4q34	3	175000000	1455	nofs	nofs
chr04	E	4q34	3	176000000	4132	nofs	nofs
chr04	E	4q34	3	176000000	2897	nofs	nofs
chr04	E	4q34	3	176000000	1925	nofs	nofs
chr04	E	4q34	3	176000000	3171	nofs	nofs
chr04	E	4q34	3	177000000	2889	nofs	nofs
chr04	E	4q34	3	177000000	3313	nofs	nofs
chr04	E	4q34	3	177000000	2951	nofs	nofs
chr04	E	4q34	3	177000000	2303	nofs	nofs
chr04	E	4q34	3	178000000	3000	nofs	nofs
chr04	E	4q34	3	178000000	2500	nofs	nofs
chr04	E	4q34	3	178000000	3576	nofs	nofs
chr04	E	4q34	3	178000000	3471	nofs	nofs
chr04	E	4q34	3	179000000	3074	nofs	nofs
chr04	E	4q34	3	179000000	2841	nofs	nofs
chr04	E	4q34	3	179000000	1980	nofs	nofs
chr04	E	4q34	3	179000000	2276	nofs	nofs
chr04	E	4q34	3	180000000	2359	nofs	nofs
chr04	E	4q34	3	180000000	2717	nofs	nofs
chr04	E	4q34	3	180000000	2437	nofs	nofs
chr04	E	4q34	3	180000000	2922	nofs	nofs
chr04	E	4q34	3	181000000	2851	nofs	nofs
chr04	E	4q34	3	181000000	3117	nofs	nofs
chr04	E	4q34	3	181000000	3394	nofs	nofs
chr04	E	4q34	3	181000000	1720	nofs	nofs
chr04	E	4q34	3	182000000	3963	nofs	nofs
chr04	E	4q34	3	182000000	3341	nofs	nofs
chr04	E	4q34	3	182000000	2944	nofs	nofs
chr04	E	4q34	3	182000000	3440	nofs	nofs
chr04	E	4q34	3	183000000	4387	nofs	nofs
chr04	E	4q34	3	183000000	2857	nofs	nofs
chr04	E	4q34	3	183000000	2820	nofs	nofs
chr04	E	4q34	3	183000000	3666	nofs	nofs
chr04	E	4q34	3	184000000	2010	nofs	nofs
chr04	E	4q35	2	184000000	1990	nofs	nofs

chr04	E	4q35	2	184000000	6573	nofs	nofs
chr04	E	4q35	2	184000000	4223	nofs	nofs
chr04	E	4q35	2	185000000	3570	nofs	nofs
chr04	E	4q35	2	185000000	4580	nofs	nofs
chr04	E	4q35	2	185000000	8555	nofs	nofs
chr04	E	4q35	2	185000000	2971	nofs	nofs
chr04	E	4q35	2	186000000	5318	nofs	nofs
chr04	E	4q35	2	186000000	5063	nofs	nofs
chr04	E	4q35	2	186000000	4154	nofs	nofs
chr04	E	4q35	2	186000000	5003	nofs	nofs
chr04	E	4q35	2	187000000	4148	nofs	nofs
chr04	E	4q35	2	187000000	2690	nofs	nofs
chr04	E	4q35	2	187000000	4569	nofs	nofs
chr04	E	4q35	2	187000000	8070	nofs	nofs
chr04	E	4q35	2	188000000	13467	nofs	nofs
chr04	E	4q35	2	188000000	10880	nofs	nofs
chr04	T	4q35	2	188000000	4097	nofs	nofs
chr04	T	4q35	2	188000000	18775	nofs	nofs
chr04	T	4q35	2	189000000	4158	nofs	nofs
chr04	T	4q35	2	189000000	3738	nofs	nofs
chr04	T	4q35	2	189000000	5181	nofs	nofs
chr04	T	4q35	2	189000000	11023	nofs	nofs
chr04	T	4q35	2	190000000	12245	nofs	nofs
chr04	T	4q35	2	190000000	10751	nofs	nofs
chr04	T	4q35	2	190000000	8610	nofs	nofs
chr04	T	4q35	2	190000000	12838	nofs	nofs
chr04	T	4q35	2	191000000	14902	nofs	nofs
chr04	T	4q35	2	191000000	22504	nofs	nofs
chr04	T	4q35	2	191000000	16288	nofs	nofs
chr05	T	5p15.3	4	125000	21633	nofs	nofs
chr05	T	5p15.3	4	375000	37325	nofs	nofs
chr05	T	5p15.3	4	625000	40906	nofs	nofs
chr05	T	5p15.3	4	875000	20455	nofs	nofs
chr05	T	5p15.3	4	1125000	36401	nofs	nofs
chr05	T	5p15.3	4	1375000	31019	nofs	nofs
chr05	T	5p15.3	4	1625000	27330	nofs	nofs

chr05	T	5p15.3	4	1875000	11839	nofs	nofs
chr05	T	5p15.3	4	2125000	14552	nofs	nofs
chr05	T	5p15.3	4	2375000	12665	nofs	nofs
chr05	T	5p15.3	4	2625000	10960	nofs	nofs
chr05	T	5p15.3	4	2875000	8435	nofs	nofs
chr05	T	5p15.3	4	3125000	5960	nofs	nofs
chr05	E	5p15.3	4	3375000	10463	nofs	nofs
chr05	E	5p15.3	4	3625000	9147	nofs	nofs
chr05	E	5p15.3	4	3875000	8412	nofs	nofs
chr05	E	5p15.3	4	4125000	6945	nofs	nofs
chr05	E	5p15.3	4	4375000	3406	nofs	nofs
chr05	E	5p15.3	4	4625000	4850	nofs	nofs
chr05	E	5p15.3	4	4875000	5914	nofs	nofs
chr05	E	5p15.3	4	5125000	3169	nofs	nofs
chr05	E	5p15.3	4	5375000	3837	nofs	nofs
chr05	E	5p15.3	4	5625000	3520	nofs	nofs
chr05	E	5p15.3	4	5875000	4443	nofs	nofs
chr05	E	5p15.3	4	6125000	5895	nofs	nofs
chr05	E	5p15.3	4	6375000	6178	nofs	nofs
chr05	E	5p15.3	4	6625000	6723	nofs	nofs
chr05	E	5p15.3	4	6875000	3385	nofs	nofs
chr05	E	5p15.3	4	7125000	3035	nofs	nofs
chr05	E	5p15.3	4	7375000	6856	nofs	nofs
chr05	E	5p15.3	4	7625000	2245	nofs	nofs
chr05	E	5p15.3	4	7875000	3106	nofs	nofs
chr05	E	5p15.3	4	8125000	3695	nofs	nofs
chr05	E	5p15.3	4	8375000	2321	nofs	nofs
chr05	E	5p15.3	4	8625000	3095	nofs	nofs
chr05	E	5p15.3	4	8875000	3793	nofs	nofs
chr05	E	5p15.3	4	9125000	2489	nofs	nofs
chr05	E	5p15.3	4	9375000	3634	nofs	nofs
chr05	E	5p15.3	4	9625000	2014	nofs	nofs
chr05	E	5p15.3	4	9875000	3133	nofs	nofs
chr05	E	5p15.2	2	10125000	2570	nofs	nofs
chr05	E	5p15.2	2	10375000	4903	nofs	nofs
chr05	E	5p15.2	2	10625000	7701	nofs	nofs

chr05	E	5p15.2	2	10875000	3539	nofs	nofs
chr05	E	5p15.2	2	11125000	4137	nofs	nofs
chr05	E	5p15.2	2	11375000	4103	nofs	nofs
chr05	E	5p15.2	2	11625000	3002	nofs	nofs
chr05	E	5p15.2	2	11875000	3801	nofs	nofs
chr05	E	5p15.2	2	12125000	4053	nofs	nofs
chr05	E	5p15.2	2	12375000	2563	nofs	nofs
chr05	E	5p15.2	2	12625000	2898	nofs	nofs
chr05	E	5p15.2	2	12875000	2672	nofs	nofs
chr05	E	5p15.2	2	13125000	2711	nofs	nofs
chr05	E	5p15.2	2	13375000	4565	nofs	nofs
chr05	E	5p15.2	2	13625000	2415	nofs	nofs
chr05	E	5p15.2	2	13875000	2432	nofs	nofs
chr05	E	5p15.2	2	14125000	3102	nofs	nofs
chr05	E	5p15.2	2	14375000	6260	nofs	nofs
chr05	E	5p15.2	2	14625000	4616	nofs	nofs
chr05	E	5p15.2	2	14875000	2119	nofs	nofs
chr05	E	5p15.2	2	15125000	3156	nofs	nofs
chr05	E	5p15.1	1	15375000	2864	nofs	nofs
chr05	E	5p15.1	1	15625000	2441	nofs	nofs
chr05	E	5p15.1	1	15875000	2132	nofs	nofs
chr05	E	5p15.1	1	16125000	1593	nofs	nofs
chr05	E	5p15.1	1	16375000	2312	nofs	nofs
chr05	E	5p15.1	1	16625000	3122	nofs	nofs
chr05	E	5p15.1	1	16875000	3071	nofs	nofs
chr05	E	5p15.1	1	17125000	6920	nofs	nofs
chr05	E	5p15.1	1	17375000	3846	nofs	nofs
chr05	E	5p15.1	1	17625000	1561	nofs	nofs
chr05	E	5p15.1	1	17875000	3329	nofs	nofs
chr05	E	5p15.1	1	18125000	2002	nofs	nofs
chr05	E	5p15.1	1	18375000	3631	nofs	nofs
chr05	E	5p15.1	1	18625000	2355	nofs	nofs
chr05	B	5p14	.	18875000	2512	fs	fs common
chr05	B	5p14	.	19125000	2164	fs	fs common
chr05	B	5p14	.	19375000	5429	fs	fs common
chr05	B	5p14	.	19625000	3325	fs	fs common

chr05	B	5p14	.	19875000	3519	fs	fs common
chr05	B	5p14	.	20125000	2689	fs	fs common
chr05	B	5p14	.	20375000	2455	fs	fs common
chr05	B	5p14	.	20625000	3453	fs	fs common
chr05	B	5p14	.	20875000	1716	fs	fs common
chr05	B	5p14	.	21125000	3235	fs	fs common
chr05	B	5p14	.	21375000	3167	fs	fs common
chr05	B	5p14	.	21625000	4106	fs	fs common
chr05	B	5p14	.	21875000	4476	fs	fs common
chr05	B	5p14	.	22125000	3512	fs	fs common
chr05	B	5p14	.	22375000	3138	fs	fs common
chr05	B	5p14	.	22625000	2327	fs	fs common
chr05	B	5p14	.	22875000	2188	fs	fs common
chr05	B	5p14	.	23125000	1932	fs	fs common
chr05	B	5p14	.	23375000	2446	fs	fs common
chr05	B	5p14	.	23625000	3995	fs	fs common
chr05	B	5p14	.	23875000	17335	fs	fs common
chr05	B	5p14	.	24125000	3401	fs	fs common
chr05	B	5p14	.	24375000	3973	fs	fs common
chr05	B	5p14	.	24625000	2091	fs	fs common
chr05	B	5p14	.	24875000	2742	fs	fs common
chr05	B	5p14	.	25125000	2877	fs	fs common
chr05	B	5p14	.	25375000	3348	fs	fs common
chr05	B	5p14	.	25625000	3747	fs	fs common
chr05	B	5p14	.	25875000	2219	fs	fs common
chr05	B	5p14	.	26125000	3401	fs	fs common
chr05	B	5p14	.	26375000	7802	fs	fs common
chr05	B	5p14	.	26625000	2105	fs	fs common
chr05	B	5p14	.	26875000	3388	fs	fs common
chr05	B	5p14	.	27125000	2588	fs	fs common
chr05	B	5p14	.	27375000	3091	fs	fs common
chr05	B	5p14	.	27625000	1686	fs	fs common
chr05	B	5p14	.	27875000	3976	fs	fs common
chr05	B	5p14	.	28125000	2865	fs	fs common
chr05	B	5p14	.	28375000	4616	fs	fs common
chr05	B	5p14	.	28625000	3266	fs	fs common

chr05	B	5p14	.	28875000	3408	fs	fs common
chr05	B	5p14	.	29125000	2243	fs	fs common
chr05	B	5p14	.	29375000	3141	fs	fs common
chr05	B	5p14	.	29625000	2068	fs	fs common
chr05	B	5p14	.	29875000	3167	fs	fs common
chr05	B	5p13	.	30125000	2538	fs	fs common
chr05	B	5p13	.	30375000	2704	fs	fs common
chr05	B	5p13	.	30625000	2019	fs	fs common
chr05	B	5p13	.	30875000	1766	fs	fs common
chr05	B	5p13	.	31125000	2126	fs	fs common
chr05	B	5p13	.	31375000	2580	fs	fs common
chr05	B	5p13	.	31625000	3115	fs	fs common
chr05	B	5p13	.	31875000	5166	fs	fs common
chr05	B	5p13	.	32125000	7452	fs	fs common
chr05	B	5p13	.	32375000	2780	fs	fs common
chr05	B	5p13	.	32625000	3729	fs	fs common
chr05	B	5p13	.	32875000	2875	fs	fs common
chr05	B	5p13	.	33125000	1411	fs	fs common
chr05	B	5p13	.	33375000	2713	fs	fs common
chr05	B	5p13	.	33625000	3958	fs	fs common
chr05	B	5p13	.	33875000	2355	fs	fs common
chr05	B	5p13	.	34125000	2589	fs	fs common
chr05	B	5p13	.	34375000	4064	fs	fs common
chr05	B	5p13	.	34625000	1859	fs	fs common
chr05	B	5p13	.	34875000	2265	fs	fs common
chr05	B	5p13	.	35125000	2540	fs	fs common
chr05	B	5p13	.	35375000	1761	fs	fs common
chr05	B	5p13	.	35625000	4254	fs	fs common
chr05	B	5p13	.	35875000	2466	fs	fs common
chr05	B	5p13	.	36125000	2678	fs	fs common
chr05	B	5p13	.	36375000	1685	fs	fs common
chr05	B	5p13	.	36625000	1501	fs	fs common
chr05	B	5p13	.	36875000	2092	fs	fs common
chr05	B	5p13	.	37125000	2356	fs	fs common
chr05	B	5p13	.	37375000	4327	fs	fs common
chr05	B	5p13	.	37625000	5217	fs	fs common

chr05	B	5p13	.	37875000	2110	fs	fs common
chr05	B	5p13	.	38125000	2721	fs	fs common
chr05	B	5p13	.	38375000	1933	fs	fs common
chr05	B	5p13	.	38625000	1944	fs	fs common
chr05	B	5p13	.	38875000	4680	fs	fs common
chr05	B	5p13	.	39125000	2115	fs	fs common
chr05	B	5p13	.	39375000	3220	fs	fs common
chr05	B	5p13	.	39625000	3803	fs	fs common
chr05	B	5p13	.	39875000	2467	fs	fs common
chr05	B	5p13	.	40125000	3074	fs	fs common
chr05	B	5p13	.	40375000	2188	fs	fs common
chr05	B	5p13	.	40625000	1915	fs	fs common
chr05	B	5p13	.	40875000	3668	fs	fs common
chr05	B	5p13	.	41125000	999	fs	fs common
chr05	B	5p13	.	41375000	1375	fs	fs common
chr05	B	5p13	.	41625000	3223	fs	fs common
chr05	B	5p13	.	41875000	1383	fs	fs common
chr05	B	5p13	.	42125000	3080	fs	fs common
chr05	B	5p13	.	42375000	2518	fs	fs common
chr05	E	5p12	2	42625000	3045	nofs	nofs
chr05	E	5p12	2	42875000	1761	nofs	nofs
chr05	E	5p12	2	43125000	10309	nofs	nofs
chr05	C	5p12	2	43375000	2691	nofs	nofs
chr05	C	5p12	2	43625000	3589	nofs	nofs
chr05	C	5p12	2	43875000	3399	nofs	nofs
chr05	C	5p12	2	44125000	2279	nofs	nofs
chr05	C	5p12	2	44375000	2143	nofs	nofs
chr05	C	5p12	2	44625000	2073	nofs	nofs
chr05	C	5p12	2	44875000	2977	nofs	nofs
chr05	C	5p11	.	45125000	2815	nofs	nofs
chr05	C	5p11	.	45375000	7324	nofs	nofs
chr05	C	5p11	.	45625000	2390	nofs	nofs
chr05	C	5p11	.	45875000	45029	nofs	nofs
chr05	C	5p11	.	46125000	176923	nofs	nofs
chr05	C	5p11	.	46375000	163591	nofs	nofs
chr05	C		.	46625000	0	nofs	nofs

chr05	C		.	46875000	0	nofs	nofs
chr05	C		.	47125000	0	nofs	nofs
chr05	C		.	47375000	0	nofs	nofs
chr05	C		.	47625000	0	nofs	nofs
chr05	C		.	47875000	0	nofs	nofs
chr05	C		.	48125000	0	nofs	nofs
chr05	C		.	48375000	0	nofs	nofs
chr05	C		.	48625000	0	nofs	nofs
chr05	C		.	48875000	0	nofs	nofs
chr05	C		.	49125000	0	nofs	nofs
chr05	C	5q11.1	.	49375000	46021	nofs	nofs
chr05	C	5q11.1	.	49625000	78032	nofs	nofs
chr05	C	5q11.1	.	49875000	5028	nofs	nofs
chr05	C	5q11.1	.	50125000	3376	nofs	nofs
chr05	C	5q11.1	.	50375000	2505	nofs	nofs
chr05	C	5q11.1	.	50625000	1931	nofs	nofs
chr05	C	5q11.2	3	50875000	2988	nofs	nofs
chr05	C	5q11.2	3	51125000	2366	nofs	nofs
chr05	C	5q11.2	3	51375000	2236	nofs	nofs
chr05	C	5q11.2	3	51625000	2563	nofs	nofs
chr05	C	5q11.2	3	51875000	2075	nofs	nofs
chr05	C	5q11.2	3	52125000	3850	nofs	nofs
chr05	C	5q11.2	3	52375000	1449	nofs	nofs
chr05	E	5q11.2	3	52625000	2231	nofs	nofs
chr05	E	5q11.2	3	52875000	1948	nofs	nofs
chr05	E	5q11.2	3	53125000	2521	nofs	nofs
chr05	E	5q11.2	3	53375000	2175	nofs	nofs
chr05	E	5q11.2	3	53625000	2318	nofs	nofs
chr05	E	5q11.2	3	53875000	3536	nofs	nofs
chr05	E	5q11.2	3	54125000	1577	nofs	nofs
chr05	E	5q11.2	3	54375000	3002	nofs	nofs
chr05	E	5q11.2	3	54625000	2057	nofs	nofs
chr05	E	5q11.2	3	54875000	2831	nofs	nofs
chr05	E	5q11.2	3	55125000	4366	nofs	nofs
chr05	E	5q11.2	3	55375000	5073	nofs	nofs
chr05	E	5q11.2	3	55625000	4069	nofs	nofs

chr05	E	5q11.2	3	55875000	2145	nofs	nofs
chr05	E	5q11.2	3	56125000	1319	nofs	nofs
chr05	E	5q11.2	3	56375000	4149	nofs	nofs
chr05	E	5q11.2	3	56625000	4384	nofs	nofs
chr05	E	5q11.2	3	56875000	1525	nofs	nofs
chr05	E	5q11.2	3	57125000	1889	nofs	nofs
chr05	E	5q11.2	3	57375000	2017	nofs	nofs
chr05	E	5q11.2	3	57625000	2417	nofs	nofs
chr05	E	5q11.2	3	57875000	6945	nofs	nofs
chr05	E	5q11.2	3	58125000	2676	nofs	nofs
chr05	E	5q11.2	3	58375000	2082	nofs	nofs
chr05	E	5q11.2	3	58625000	3338	nofs	nofs
chr05	E	5q11.2	3	58875000	1777	nofs	nofs
chr05	E	5q12	1	59125000	3052	nofs	nofs
chr05	E	5q12	1	59375000	1840	nofs	nofs
chr05	E	5q12	1	59625000	2057	nofs	nofs
chr05	E	5q12	1	59875000	4462	nofs	nofs
chr05	E	5q12	1	60125000	2780	nofs	nofs
chr05	E	5q12	1	60375000	3083	nofs	nofs
chr05	E	5q12	1	60625000	3494	nofs	nofs
chr05	E	5q12	1	60875000	1659	nofs	nofs
chr05	E	5q12	1	61125000	2220	nofs	nofs
chr05	E	5q12	1	61375000	2340	nofs	nofs
chr05	E	5q12	1	61625000	3219	nofs	nofs
chr05	E	5q12	1	61875000	3432	nofs	nofs
chr05	E	5q12	1	62125000	1348	nofs	nofs
chr05	E	5q12	1	62375000	3014	nofs	nofs
chr05	E	5q12	1	62625000	1454	nofs	nofs
chr05	E	5q12	1	62875000	2038	nofs	nofs
chr05	E	5q12	1	63125000	1801	nofs	nofs
chr05	E	5q12	1	63375000	1943	nofs	nofs
chr05	E	5q12	1	63625000	2458	nofs	nofs
chr05	E	5q12	1	63875000	2512	nofs	nofs
chr05	E	5q12	1	64125000	2380	nofs	nofs
chr05	E	5q12	1	64375000	1859	nofs	nofs
chr05	E	5q12	1	64625000	3167	nofs	nofs

chr05	E	5q12	1	64875000	2553	nofs	nofs
chr05	E	5q12	1	65125000	4086	nofs	nofs
chr05	E	5q12	1	65375000	3985	nofs	nofs
chr05	E	5q12	1	65625000	2511	nofs	nofs
chr05	E	5q12	1	65875000	2631	nofs	nofs
chr05	E	5q12	1	66125000	1877	nofs	nofs
chr05	E	5q12	1	66375000	1881	nofs	nofs
chr05	E	5q12	1	66625000	5210	nofs	nofs
chr05	E	5q13.1	1	66875000	2010	nofs	nofs
chr05	E	5q13.1	1	67125000	2278	nofs	nofs
chr05	E	5q13.1	1	67375000	2660	nofs	nofs
chr05	E	5q13.1	1	67625000	2948	nofs	nofs
chr05	E	5q13.1	1	67875000	1367	nofs	nofs
chr05	E	5q13.1	1	68125000	1863	nofs	nofs
chr05	E	5q13.1	1	68375000	5908	nofs	nofs
chr05	E	5q13.2	3	68625000	3438	nofs	nofs
chr05	E	5q13.2	3	68875000	4379	nofs	nofs
chr05	E	5q13.2	3	69125000	3387	nofs	nofs
chr05	E	5q13.2	3	69375000	3100	nofs	nofs
chr05	E	5q13.2	3	69625000	3625	nofs	nofs
chr05	E	5q13.2	3	69875000	2916	nofs	nofs
chr05	E	5q13.2	3	70125000	3039	nofs	nofs
chr05	E	5q13.2	3	70375000	2750	nofs	nofs
chr05	E	5q13.2	3	70625000	2853	nofs	nofs
chr05	E	5q13.2	3	70875000	6154	nofs	nofs
chr05	E	5q13.2	3	71125000	3340	nofs	nofs
chr05	E	5q13.2	3	71375000	1702	nofs	nofs
chr05	E	5q13.2	3	71625000	1841	nofs	nofs
chr05	E	5q13.2	3	71875000	1977	nofs	nofs
chr05	E	5q13.2	3	72125000	1498	nofs	nofs
chr05	E	5q13.2	3	72375000	2510	nofs	nofs
chr05	E	5q13.2	3	72625000	2109	nofs	nofs
chr05	E	5q13.2	3	72875000	2611	nofs	nofs
chr05	E	5q13.2	3	73125000	3055	nofs	nofs
chr05	E	5q13.2	3	73375000	2181	nofs	nofs
chr05	E	5q13.3	3	73625000	1959	nofs	nofs

chr05	E	5q13.3	3	73875000	2168	nofs	nofs
chr05	E	5q13.3	3	74125000	2597	nofs	nofs
chr05	E	5q13.3	3	74375000	2920	nofs	nofs
chr05	E	5q13.3	3	74625000	4066	nofs	nofs
chr05	E	5q13.3	3	74875000	1670	nofs	nofs
chr05	E	5q13.3	3	75125000	1886	nofs	nofs
chr05	E	5q13.3	3	75375000	2449	nofs	nofs
chr05	E	5q13.3	3	75625000	4040	nofs	nofs
chr05	E	5q13.3	3	75875000	2398	nofs	nofs
chr05	E	5q13.3	3	76125000	4821	nofs	nofs
chr05	E	5q13.3	3	76375000	4702	nofs	nofs
chr05	E	5q13.3	3	76625000	1110	nofs	nofs
chr05	E	5q13.3	3	76875000	1438	nofs	nofs
chr05	E	5q14	2	77125000	2436	nofs	nofs
chr05	E	5q14	2	77375000	3810	nofs	nofs
chr05	E	5q14	2	77625000	2770	nofs	nofs
chr05	E	5q14	2	77875000	2452	nofs	nofs
chr05	E	5q14	2	78125000	2235	nofs	nofs
chr05	E	5q14	2	78375000	3568	nofs	nofs
chr05	E	5q14	2	78625000	4011	nofs	nofs
chr05	E	5q14	2	78875000	3180	nofs	nofs
chr05	E	5q14	2	79125000	2242	nofs	nofs
chr05	E	5q14	2	79375000	1686	nofs	nofs
chr05	E	5q14	2	79625000	5384	nofs	nofs
chr05	E	5q14	2	79875000	3951	nofs	nofs
chr05	E	5q14	2	80125000	3549	nofs	nofs
chr05	E	5q14	2	80375000	3046	nofs	nofs
chr05	E	5q14	2	80625000	2744	nofs	nofs
chr05	E	5q14	2	80875000	2251	nofs	nofs
chr05	E	5q14	2	81125000	1356	nofs	nofs
chr05	E	5q14	2	81375000	4582	nofs	nofs
chr05	E	5q14	2	81625000	2204	nofs	nofs
chr05	E	5q14	2	81875000	3982	nofs	nofs
chr05	E	5q14	2	82125000	2305	nofs	nofs
chr05	E	5q14	2	82375000	2182	nofs	nofs
chr05	E	5q14	2	82625000	1293	nofs	nofs

chr05	E	5q14	2	82875000	2225	nofs	nofs
chr05	E	5q14	2	83125000	2226	nofs	nofs
chr05	E	5q14	2	83375000	2632	nofs	nofs
chr05	E	5q14	2	83625000	3198	nofs	nofs
chr05	E	5q14	2	83875000	1832	nofs	nofs
chr05	E	5q14	2	84125000	4241	nofs	nofs
chr05	E	5q14	2	84375000	1809	nofs	nofs
chr05	E	5q14	2	84625000	2117	nofs	nofs
chr05	E	5q14	2	84875000	2829	nofs	nofs
chr05	E	5q14	2	85125000	2784	nofs	nofs
chr05	E	5q14	2	85375000	3062	nofs	nofs
chr05	E	5q14	2	85625000	3184	nofs	nofs
chr05	E	5q14	2	85875000	4281	nofs	nofs
chr05	E	5q14	2	86125000	1919	nofs	nofs
chr05	E	5q14	2	86375000	6264	nofs	nofs
chr05	E	5q14	2	86625000	4047	nofs	nofs
chr05	E	5q14	2	86875000	2722	nofs	nofs
chr05	E	5q14	2	87125000	1516	nofs	nofs
chr05	E	5q14	2	87375000	3112	nofs	nofs
chr05	E	5q14	2	87625000	1554	nofs	nofs
chr05	E	5q14	2	87875000	2942	nofs	nofs
chr05	E	5q14	2	88125000	1995	nofs	nofs
chr05	E	5q14	2	88375000	2408	nofs	nofs
chr05	E	5q14	2	88625000	3718	nofs	nofs
chr05	E	5q14	2	88875000	1691	nofs	nofs
chr05	E	5q14	2	89125000	1876	nofs	nofs
chr05	E	5q14	2	89375000	2028	nofs	nofs
chr05	E	5q14	2	89625000	1638	nofs	nofs
chr05	E	5q14	2	89875000	4358	nofs	nofs
chr05	E	5q14	2	90125000	2321	nofs	nofs
chr05	E	5q14	2	90375000	2436	nofs	nofs
chr05	E	5q14	2	90625000	2814	nofs	nofs
chr05	E	5q14	2	90875000	2085	nofs	nofs
chr05	E	5q14	2	91125000	1792	nofs	nofs
chr05	E	5q14	2	91375000	1841	nofs	nofs
chr05	E	5q14	2	91625000	2142	nofs	nofs

chr05	E	5q14	2	91875000	2129	nofs	nofs
chr05	E	5q15	3	92125000	2112	fs	fs common
chr05	E	5q15	3	92375000	1127	fs	fs common
chr05	E	5q15	3	92625000	1161	fs	fs common
chr05	E	5q15	3	92875000	2762	fs	fs common
chr05	E	5q15	3	93125000	1299	fs	fs common
chr05	E	5q15	3	93375000	2894	fs	fs common
chr05	E	5q15	3	93625000	1725	fs	fs common
chr05	E	5q15	3	93875000	2398	fs	fs common
chr05	E	5q15	3	94125000	1795	fs	fs common
chr05	E	5q15	3	94375000	2101	fs	fs common
chr05	E	5q15	3	94625000	2368	fs	fs common
chr05	E	5q15	3	94875000	1418	fs	fs common
chr05	E	5q15	3	95125000	1543	fs	fs common
chr05	E	5q15	3	95375000	2347	fs	fs common
chr05	E	5q15	3	95625000	1847	fs	fs common
chr05	E	5q15	3	95875000	2369	fs	fs common
chr05	E	5q15	3	96125000	1953	fs	fs common
chr05	E	5q15	3	96375000	2347	fs	fs common
chr05	E	5q15	3	96625000	3270	fs	fs common
chr05	E	5q15	3	96875000	2479	fs	fs common
chr05	E	5q15	3	97125000	3395	fs	fs common
chr05	E	5q15	3	97375000	1934	fs	fs common
chr05	E	5q21	2	97625000	2194	fs	fs common
chr05	E	5q21	2	97875000	951	fs	fs common
chr05	E	5q21	2	98125000	1902	fs	fs common
chr05	E	5q21	2	98375000	1698	fs	fs common
chr05	E	5q21	2	98625000	35477	fs	fs common
chr05	E	5q21	2	98875000	4383	fs	fs common
chr05	E	5q21	2	99125000	3525	fs	fs common
chr05	E	5q21	2	99375000	2068	fs	fs common
chr05	E	5q21	2	99625000	3200	fs	fs common
chr05	E	5q21	2	99875000	4233	fs	fs common
chr05	E	5q21	2	100000000	2805	fs	fs common
chr05	E	5q21	2	100000000	2788	fs	fs common
chr05	E	5q21	2	101000000	2589	fs	fs common

chr05	E	5q21	2	101000000	2926	fs	fs common
chr05	E	5q21	2	101000000	1769	fs	fs common
chr05	E	5q21	2	101000000	2207	fs	fs common
chr05	E	5q21	2	102000000	2593	fs	fs common
chr05	E	5q21	2	102000000	1761	fs	fs common
chr05	E	5q21	2	102000000	2043	fs	fs common
chr05	E	5q21	2	102000000	1835	fs	fs common
chr05	E	5q21	2	103000000	2195	fs	fs common
chr05	E	5q21	2	103000000	1743	fs	fs common
chr05	E	5q21	2	103000000	2102	fs	fs common
chr05	E	5q21	2	103000000	3284	fs	fs common
chr05	E	5q21	2	104000000	2080	fs	fs common
chr05	E	5q21	2	104000000	1805	fs	fs common
chr05	E	5q21	2	104000000	4037	fs	fs common
chr05	E	5q21	2	104000000	2031	fs	fs common
chr05	E	5q21	2	105000000	2694	fs	fs common
chr05	E	5q21	2	105000000	2053	fs	fs common
chr05	E	5q21	2	105000000	3603	fs	fs common
chr05	E	5q21	2	105000000	3281	fs	fs common
chr05	E	5q21	2	106000000	3294	fs	fs common
chr05	E	5q21	2	106000000	6545	fs	fs common
chr05	E	5q21	2	106000000	2201	fs	fs common
chr05	E	5q21	2	106000000	2148	fs	fs common
chr05	E	5q21	2	107000000	1917	fs	fs common
chr05	E	5q21	2	107000000	2381	fs	fs common
chr05	E	5q21	2	107000000	1952	fs	fs common
chr05	E	5q21	2	107000000	2010	fs	fs common
chr05	E	5q21	2	108000000	2189	fs	fs common
chr05	E	5q21	2	108000000	3382	fs	fs common
chr05	E	5q21	2	108000000	2118	fs	fs common
chr05	E	5q21	2	108000000	2440	fs	fs common
chr05	E	5q21	2	109000000	5477	fs	fs common
chr05	E	5q21	2	109000000	1821	fs	fs common
chr05	E	5q21	2	109000000	1384	fs	fs common
chr05	E	5q21	2	109000000	1897	fs	fs common
chr05	E	5q21	2	110000000	1667	fs	fs common

chr05	E	5q22	2	110000000	2139	nofs	nofs
chr05	E	5q22	2	110000000	2002	nofs	nofs
chr05	E	5q22	2	110000000	1517	nofs	nofs
chr05	E	5q22	2	111000000	1836	nofs	nofs
chr05	E	5q22	2	111000000	2031	nofs	nofs
chr05	E	5q22	2	111000000	2155	nofs	nofs
chr05	E	5q22	2	111000000	1824	nofs	nofs
chr05	E	5q22	2	112000000	1334	nofs	nofs
chr05	E	5q22	2	112000000	989	nofs	nofs
chr05	E	5q22	2	112000000	1454	nofs	nofs
chr05	E	5q22	2	112000000	2891	nofs	nofs
chr05	E	5q22	2	113000000	1579	nofs	nofs
chr05	E	5q22	2	113000000	4304	nofs	nofs
chr05	E	5q22	2	113000000	1138	nofs	nofs
chr05	E	5q22	2	113000000	1855	nofs	nofs
chr05	E	5q22	2	114000000	2414	nofs	nofs
chr05	E	5q22	2	114000000	1785	nofs	nofs
chr05	E	5q22	2	114000000	1827	nofs	nofs
chr05	E	5q22	2	114000000	3302	nofs	nofs
chr05	E	5q22	2	115000000	2870	nofs	nofs
chr05	E	5q22	2	115000000	1694	nofs	nofs
chr05	E	5q22	2	115000000	1328	nofs	nofs
chr05	E	5q22	2	115000000	2077	nofs	nofs
chr05	B	5q23.1	.	116000000	2070	nofs	nofs
chr05	B	5q23.1	.	116000000	1535	nofs	nofs
chr05	B	5q23.1	.	116000000	2793	nofs	nofs
chr05	B	5q23.1	.	116000000	4430	nofs	nofs
chr05	B	5q23.1	.	117000000	2339	nofs	nofs
chr05	B	5q23.1	.	117000000	4673	nofs	nofs
chr05	B	5q23.1	.	117000000	2313	nofs	nofs
chr05	B	5q23.1	.	117000000	3769	nofs	nofs
chr05	B	5q23.1	.	118000000	1926	nofs	nofs
chr05	B	5q23.1	.	118000000	9041	nofs	nofs
chr05	B	5q23.1	.	118000000	2146	nofs	nofs
chr05	B	5q23.1	.	118000000	5416	nofs	nofs
chr05	B	5q23.1	.	119000000	2101	nofs	nofs

chr05	B	5q23.1	.	119000000	2890	nofs	nofs
chr05	B	5q23.1	.	119000000	2225	nofs	nofs
chr05	B	5q23.1	.	119000000	4121	nofs	nofs
chr05	B	5q23.1	.	120000000	2301	nofs	nofs
chr05	B	5q23.1	.	120000000	2218	nofs	nofs
chr05	B	5q23.1	.	120000000	2444	nofs	nofs
chr05	B	5q23.1	.	120000000	3119	nofs	nofs
chr05	B	5q23.1	.	121000000	1849	nofs	nofs
chr05	B	5q23.1	.	121000000	2835	nofs	nofs
chr05	B	5q23.1	.	121000000	1632	nofs	nofs
chr05	B	5q23.1	.	121000000	1376	nofs	nofs
chr05	B	5q23.1	.	122000000	1741	nofs	nofs
chr05	B	5q23.2	.	122000000	1961	nofs	nofs
chr05	B	5q23.2	.	122000000	3211	nofs	nofs
chr05	B	5q23.2	.	122000000	2365	nofs	nofs
chr05	B	5q23.2	.	123000000	1606	nofs	nofs
chr05	B	5q23.2	.	123000000	2284	nofs	nofs
chr05	B	5q23.2	.	123000000	2677	nofs	nofs
chr05	B	5q23.2	.	123000000	2003	nofs	nofs
chr05	B	5q23.2	.	124000000	2830	nofs	nofs
chr05	B	5q23.2	.	124000000	2010	nofs	nofs
chr05	B	5q23.2	.	124000000	2546	nofs	nofs
chr05	B	5q23.2	.	124000000	4891	nofs	nofs
chr05	B	5q23.2	.	125000000	2596	nofs	nofs
chr05	B	5q23.2	.	125000000	4052	nofs	nofs
chr05	B	5q23.2	.	125000000	2079	nofs	nofs
chr05	B	5q23.2	.	125000000	1344	nofs	nofs
chr05	B	5q23.2	.	126000000	2800	nofs	nofs
chr05	B	5q23.2	.	126000000	3523	nofs	nofs
chr05	B	5q23.2	.	126000000	7358	nofs	nofs
chr05	B	5q23.2	.	126000000	1385	nofs	nofs
chr05	B	5q23.2	.	127000000	2165	nofs	nofs
chr05	B	5q23.2	.	127000000	2801	nofs	nofs
chr05	B	5q23.2	.	127000000	1958	nofs	nofs
chr05	B	5q23.2	.	127000000	2439	nofs	nofs
chr05	B	5q23.2	.	128000000	1944	nofs	nofs

chr05	E	5q23.3	4	128000000	2539	nofs	nofs
chr05	E	5q23.3	4	128000000	1986	nofs	nofs
chr05	E	5q23.3	4	128000000	2327	nofs	nofs
chr05	E	5q23.3	4	129000000	1218	nofs	nofs
chr05	E	5q23.3	4	129000000	3082	nofs	nofs
chr05	E	5q23.3	4	129000000	1288	nofs	nofs
chr05	E	5q23.3	4	129000000	1530	nofs	nofs
chr05	E	5q23.3	4	130000000	1815	nofs	nofs
chr05	E	5q23.3	4	130000000	3025	nofs	nofs
chr05	E	5q23.3	4	130000000	2284	nofs	nofs
chr05	E	5q23.3	4	130000000	1640	nofs	nofs
chr05	E	5q23.3	4	131000000	3676	nofs	nofs
chr05	E	5q23.3	4	131000000	4067	nofs	nofs
chr05	E	5q23.3	4	131000000	2570	nofs	nofs
chr05	E	5q23.3	4	131000000	1465	nofs	nofs
chr05	E	5q23.3	4	132000000	3152	nofs	nofs
chr05	E	5q23.3	4	132000000	1710	nofs	nofs
chr05	E	5q23.3	4	132000000	2426	nofs	nofs
chr05	E	5q23.3	4	132000000	4573	nofs	nofs
chr05	E	5q31.1	2	133000000	2703	fs	fs common
chr05	E	5q31.1	2	133000000	2669	fs	fs common
chr05	E	5q31.1	2	133000000	2657	fs	fs common
chr05	E	5q31.1	2	133000000	3455	fs	fs common
chr05	E	5q31.1	2	134000000	3116	fs	fs common
chr05	E	5q31.1	2	134000000	5472	fs	fs common
chr05	E	5q31.1	2	134000000	6223	fs	fs common
chr05	E	5q31.1	2	134000000	1388	fs	fs common
chr05	E	5q31.1	2	135000000	1058	fs	fs common
chr05	E	5q31.1	2	135000000	2953	fs	fs common
chr05	E	5q31.1	2	135000000	35180	fs	fs common
chr05	E	5q31.1	2	135000000	1712	fs	fs common
chr05	E	5q31.1	2	136000000	1868	fs	fs common
chr05	E	5q31.1	2	136000000	2868	fs	fs common
chr05	E	5q31.1	2	136000000	1595	fs	fs common
chr05	E	5q31.1	2	136000000	1392	fs	fs common
chr05	E	5q31.1	2	137000000	1560	fs	fs common

chr05	E	5q31.1	2	137000000	3720	fs	fs common
chr05	E	5q31.1	2	137000000	1643	fs	fs common
chr05	E	5q31.1	2	137000000	4481	fs	fs common
chr05	E	5q31.1	2	138000000	6602	fs	fs common
chr05	E	5q31.1	2	138000000	2832	fs	fs common
chr05	E	5q31.1	2	138000000	4883	fs	fs common
chr05	E	5q31.1	2	138000000	2438	fs	fs common
chr05	E	5q31.1	2	139000000	4845	fs	fs common
chr05	E	5q31.1	2	139000000	7418	fs	fs common
chr05	E	5q31.1	2	139000000	4608	fs	fs common
chr05	E	5q31.1	2	139000000	2022	fs	fs common
chr05	E	5q31.1	2	140000000	2714	fs	fs common
chr05	E	5q31.1	2	140000000	3161	fs	fs common
chr05	E	5q31.1	2	140000000	1679	fs	fs common
chr05	E	5q31.1	2	140000000	1745	fs	fs common
chr05	E	5q31.1	2	141000000	1986	fs	fs common
chr05	E	5q31.1	2	141000000	1946	fs	fs common
chr05	E	5q31.1	2	141000000	2222	fs	fs common
chr05	E	5q31.1	2	141000000	3814	fs	fs common
chr05	E	5q31.1	2	142000000	4168	fs	fs common
chr05	E	5q31.1	2	142000000	3291	fs	fs common
chr05	E	5q31.1	2	142000000	1601	fs	fs common
chr05	E	5q31.1	2	142000000	2935	fs	fs common
chr05	E	5q31.1	2	143000000	2931	fs	fs common
chr05	E	5q31.1	2	143000000	2570	fs	fs common
chr05	E	5q31.1	2	143000000	1610	fs	fs common
chr05	E	5q31.1	2	143000000	2606	fs	fs common
chr05	E	5q31.1	2	144000000	2221	fs	fs common
chr05	E	5q31.1	2	144000000	3015	fs	fs common
chr05	E	5q31.1	2	144000000	2374	fs	fs common
chr05	E	5q31.1	2	144000000	1827	fs	fs common
chr05	E	5q32	4	145000000	4130	nofs	nofs
chr05	E	5q32	4	145000000	1439	nofs	nofs
chr05	E	5q32	4	145000000	4504	nofs	nofs
chr05	E	5q32	4	145000000	2221	nofs	nofs
chr05	E	5q32	4	146000000	3526	nofs	nofs

chr05	E	5q32	4	146000000	1955	nofs	nofs
chr05	E	5q32	4	146000000	2465	nofs	nofs
chr05	E	5q32	4	146000000	2626	nofs	nofs
chr05	E	5q32	4	147000000	7185	nofs	nofs
chr05	E	5q32	4	147000000	1992	nofs	nofs
chr05	E	5q32	4	147000000	2868	nofs	nofs
chr05	E	5q32	4	147000000	2969	nofs	nofs
chr05	E	5q32	4	148000000	3344	nofs	nofs
chr05	E	5q32	4	148000000	3127	nofs	nofs
chr05	E	5q32	4	148000000	3627	nofs	nofs
chr05	E	5q32	4	148000000	1398	nofs	nofs
chr05	E	5q32	4	149000000	1806	nofs	nofs
chr05	E	5q32	4	149000000	3283	nofs	nofs
chr05	E	5q32	4	149000000	7662	nofs	nofs
chr05	E	5q32	4	149000000	2123	nofs	nofs
chr05	E	5q32	4	150000000	2103	nofs	nofs
chr05	E	5q32	4	150000000	4168	nofs	nofs
chr05	E	5q33.1	3	150000000	2002	nofs	nofs
chr05	E	5q33.1	3	150000000	3914	nofs	nofs
chr05	E	5q33.1	3	151000000	3863	nofs	nofs
chr05	E	5q33.1	3	151000000	3690	nofs	nofs
chr05	E	5q33.1	3	151000000	3587	nofs	nofs
chr05	E	5q33.1	3	151000000	1337	nofs	nofs
chr05	E	5q33.1	3	152000000	1506	nofs	nofs
chr05	E	5q33.1	3	152000000	2958	nofs	nofs
chr05	E	5q33.1	3	152000000	3434	nofs	nofs
chr05	E	5q33.1	3	152000000	1122	nofs	nofs
chr05	E	5q33.1	3	153000000	3239	nofs	nofs
chr05	E	5q33.1	3	153000000	2452	nofs	nofs
chr05	E	5q33.2	2	153000000	3383	nofs	nofs
chr05	E	5q33.2	2	153000000	1606	nofs	nofs
chr05	E	5q33.2	2	154000000	1916	nofs	nofs
chr05	E	5q33.2	2	154000000	2842	nofs	nofs
chr05	E	5q33.2	2	154000000	5543	nofs	nofs
chr05	E	5q33.2	2	154000000	3395	nofs	nofs
chr05	E	5q33.2	2	155000000	1995	nofs	nofs

chr05	E	5q33.2	2	155000000	2009	nofs	nofs
chr05	E	5q33.2	2	155000000	2122	nofs	nofs
chr05	E	5q33.2	2	155000000	1781	nofs	nofs
chr05	E	5q33.2	2	156000000	1183	nofs	nofs
chr05	E	5q33.3	1	156000000	2654	nofs	nofs
chr05	E	5q33.3	1	156000000	1738	nofs	nofs
chr05	E	5q33.3	1	156000000	4962	nofs	nofs
chr05	E	5q33.3	1	157000000	1460	nofs	nofs
chr05	E	5q33.3	1	157000000	2736	nofs	nofs
chr05	E	5q33.3	1	157000000	2873	nofs	nofs
chr05	E	5q33.3	1	157000000	3584	nofs	nofs
chr05	E	5q33.3	1	158000000	2314	nofs	nofs
chr05	E	5q33.3	1	158000000	3975	nofs	nofs
chr05	E	5q33.3	1	158000000	1525	nofs	nofs
chr05	E	5q33.3	1	158000000	3075	nofs	nofs
chr05	E	5q33.3	1	159000000	1899	nofs	nofs
chr05	E	5q33.3	1	159000000	1637	nofs	nofs
chr05	E	5q33.3	1	159000000	2395	nofs	nofs
chr05	E	5q33.3	1	159000000	2962	nofs	nofs
chr05	E	5q33.3	1	160000000	3852	nofs	nofs
chr05	E	5q33.3	1	160000000	3574	nofs	nofs
chr05	E	5q34	1	160000000	4009	nofs	nofs
chr05	E	5q34	1	160000000	2582	nofs	nofs
chr05	E	5q34	1	161000000	2354	nofs	nofs
chr05	E	5q34	1	161000000	1781	nofs	nofs
chr05	E	5q34	1	161000000	2956	nofs	nofs
chr05	E	5q34	1	161000000	2685	nofs	nofs
chr05	E	5q34	1	162000000	3188	nofs	nofs
chr05	E	5q34	1	162000000	2214	nofs	nofs
chr05	E	5q34	1	162000000	3618	nofs	nofs
chr05	E	5q34	1	162000000	3856	nofs	nofs
chr05	E	5q34	1	163000000	2606	nofs	nofs
chr05	E	5q34	1	163000000	4050	nofs	nofs
chr05	E	5q34	1	163000000	1858	nofs	nofs
chr05	E	5q34	1	163000000	2364	nofs	nofs
chr05	E	5q34	1	164000000	3054	nofs	nofs

chr05	E	5q34	1	164000000	1754	nofs	nofs
chr05	E	5q34	1	164000000	3380	nofs	nofs
chr05	E	5q34	1	164000000	2347	nofs	nofs
chr05	E	5q34	1	165000000	2105	nofs	nofs
chr05	E	5q34	1	165000000	2897	nofs	nofs
chr05	E	5q34	1	165000000	2739	nofs	nofs
chr05	E	5q34	1	165000000	3718	nofs	nofs
chr05	E	5q34	1	166000000	1991	nofs	nofs
chr05	E	5q34	1	166000000	1756	nofs	nofs
chr05	E	5q34	1	166000000	4387	nofs	nofs
chr05	E	5q34	1	166000000	3106	nofs	nofs
chr05	E	5q34	1	167000000	2782	nofs	nofs
chr05	E	5q34	1	167000000	3054	nofs	nofs
chr05	E	5q34	1	167000000	5493	nofs	nofs
chr05	E	5q34	1	167000000	3590	nofs	nofs
chr05	E	5q34	1	168000000	3635	nofs	nofs
chr05	E	5q34	1	168000000	2549	nofs	nofs
chr05	E	5q34	1	168000000	3716	nofs	nofs
chr05	E	5q34	1	168000000	2552	nofs	nofs
chr05	E	5q35.1	1	169000000	3373	fs	fs rare
chr05	E	5q35.1	1	169000000	2173	fs	fs rare
chr05	E	5q35.1	1	169000000	3524	fs	fs rare
chr05	E	5q35.1	1	169000000	2006	fs	fs rare
chr05	E	5q35.1	1	170000000	2824	fs	fs rare
chr05	E	5q35.1	1	170000000	3028	fs	fs rare
chr05	E	5q35.1	1	170000000	3526	fs	fs rare
chr05	E	5q35.1	1	170000000	1988	fs	fs rare
chr05	E	5q35.1	1	171000000	1510	fs	fs rare
chr05	E	5q35.1	1	171000000	5227	fs	fs rare
chr05	E	5q35.1	1	171000000	3623	fs	fs rare
chr05	E	5q35.1	1	171000000	3903	fs	fs rare
chr05	E	5q35.1	1	172000000	4695	fs	fs rare
chr05	E	5q35.1	1	172000000	5105	fs	fs rare
chr05	E	5q35.1	1	172000000	3861	fs	fs rare
chr05	E	5q35.1	1	172000000	5032	fs	fs rare
chr05	E	5q35.1	1	173000000	5225	fs	fs rare

chr05	E	5q35.2	2	173000000	3593	fs	fs rare
chr05	E	5q35.2	2	173000000	3799	fs	fs rare
chr05	E	5q35.2	2	173000000	4569	fs	fs rare
chr05	E	5q35.2	2	174000000	3732	fs	fs rare
chr05	E	5q35.2	2	174000000	6095	fs	fs rare
chr05	E	5q35.2	2	174000000	4522	fs	fs rare
chr05	E	5q35.2	2	174000000	3957	fs	fs rare
chr05	E	5q35.2	2	175000000	4077	fs	fs rare
chr05	E	5q35.2	2	175000000	3605	fs	fs rare
chr05	E	5q35.2	2	175000000	1852	fs	fs rare
chr05	E	5q35.2	2	175000000	4507	fs	fs rare
chr05	E	5q35.2	2	176000000	6994	fs	fs rare
chr05	E	5q35.2	2	176000000	7016	fs	fs rare
chr05	E	5q35.2	2	176000000	8821	fs	fs rare
chr05	E	5q35.2	2	176000000	6220	fs	fs rare
chr05	E	5q35.3	4	177000000	4926	fs	fs rare
chr05	E	5q35.3	4	177000000	3456	fs	fs rare
chr05	E	5q35.3	4	177000000	5601	fs	fs rare
chr05	E	5q35.3	4	177000000	11076	fs	fs rare
chr05	T	5q35.3	4	178000000	7273	fs	fs rare
chr05	T	5q35.3	4	178000000	7430	fs	fs rare
chr05	T	5q35.3	4	178000000	2950	fs	fs rare
chr05	T	5q35.3	4	178000000	6140	fs	fs rare
chr05	T	5q35.3	4	179000000	9664	fs	fs rare
chr05	T	5q35.3	4	179000000	9947	fs	fs rare
chr05	T	5q35.3	4	179000000	8773	fs	fs rare
chr05	T	5q35.3	4	179000000	3776	fs	fs rare
chr05	T	5q35.3	4	180000000	7057	fs	fs rare
chr05	T	5q35.3	4	180000000	11807	fs	fs rare
chr05	T	5q35.3	4	180000000	6358	fs	fs rare
chr05	T	5q35.3	4	180000000	9734	fs	fs rare
chr05	T	5q35.3	4	181000000	6743	fs	fs rare
chr06	T	6p25	.	125000	5776	nofs	nofs
chr06	T	6p25	.	375000	13055	nofs	nofs
chr06	T	6p25	.	625000	7831	nofs	nofs
chr06	T	6p25	.	875000	9689	nofs	nofs

chr06	T	6p25	.	1125000	6332	nofs	nofs
chr06	T	6p25	.	1375000	10775	nofs	nofs
chr06	T	6p25	.	1625000	2963	nofs	nofs
chr06	T	6p25	.	1875000	4138	nofs	nofs
chr06	T	6p25	.	2125000	3327	nofs	nofs
chr06	T	6p25	.	2375000	4742	nofs	nofs
chr06	T	6p25	.	2625000	3455	nofs	nofs
chr06	T	6p25	.	2875000	7759	nofs	nofs
chr06	T	6p25	.	3125000	8943	nofs	nofs
chr06	B	6p25	.	3375000	3764	nofs	nofs
chr06	B	6p25	.	3625000	3833	nofs	nofs
chr06	B	6p25	.	3875000	2477	nofs	nofs
chr06	B	6p25	.	4125000	3852	fs	fs common
chr06	B	6p25	.	4375000	2418	fs	fs common
chr06	B	6p25	.	4625000	2762	fs	fs common
chr06	B	6p25	.	4875000	6971	fs	fs common
chr06	B	6p25	.	5125000	2731	fs	fs common
chr06	B	6p25	.	5375000	2078	fs	fs common
chr06	B	6p25	.	5625000	2211	fs	fs common
chr06	B	6p25	.	5875000	4657	fs	fs common
chr06	B	6p25	.	6125000	2700	fs	fs common
chr06	B	6p25	.	6375000	2560	fs	fs common
chr06	B	6p25	.	6625000	4906	fs	fs common
chr06	B	6p25	.	6875000	3406	fs	fs common
chr06	B	6p25	.	7125000	4384	fs	fs common
chr06	B	6p24	.	7375000	4220	nofs	nofs
chr06	B	6p24	.	7625000	2124	nofs	nofs
chr06	B	6p24	.	7875000	1892	nofs	nofs
chr06	B	6p24	.	8125000	3600	nofs	nofs
chr06	B	6p24	.	8375000	1594	nofs	nofs
chr06	B	6p24	.	8625000	3994	nofs	nofs
chr06	B	6p24	.	8875000	3582	nofs	nofs
chr06	B	6p24	.	9125000	2730	nofs	nofs
chr06	B	6p24	.	9375000	2368	nofs	nofs
chr06	B	6p24	.	9625000	2400	nofs	nofs
chr06	B	6p24	.	9875000	3602	nofs	nofs

chr06	B	6p24	.	10125000	2387	nofs	nofs
chr06	B	6p24	.	10375000	4471	nofs	nofs
chr06	B	6p24	.	10625000	5431	nofs	nofs
chr06	B	6p24	.	10875000	4934	nofs	nofs
chr06	B	6p24	.	11125000	2389	nofs	nofs
chr06	B	6p24	.	11375000	3243	nofs	nofs
chr06	B	6p24	.	11625000	3242	nofs	nofs
chr06	B	6p24	.	11875000	2288	nofs	nofs
chr06	B	6p24	.	12125000	3764	nofs	nofs
chr06	B	6p24	.	12375000	3917	nofs	nofs
chr06	B	6p24	.	12625000	2151	nofs	nofs
chr06	B	6p24	.	12875000	3631	nofs	nofs
chr06	B	6p24	.	13125000	2853	nofs	nofs
chr06	B	6p24	.	13375000	2383	nofs	nofs
chr06	B	6p23	.	13625000	4761	fs	fs rare
chr06	B	6p23	.	13875000	5177	fs	fs rare
chr06	B	6p23	.	14125000	3779	fs	fs rare
chr06	B	6p23	.	14375000	2819	fs	fs rare
chr06	B	6p23	.	14625000	2108	fs	fs rare
chr06	B	6p23	.	14875000	2662	fs	fs rare
chr06	B	6p23	.	15125000	4454	fs	fs rare
chr06	B	6p23	.	15375000	6211	fs	fs rare
chr06	E	6p22.3	3	15625000	2697	nofs	nofs
chr06	E	6p22.3	3	15875000	2729	nofs	nofs
chr06	E	6p22.3	3	16125000	3479	nofs	nofs
chr06	E	6p22.3	3	16375000	5211	nofs	nofs
chr06	E	6p22.3	3	16625000	2436	nofs	nofs
chr06	E	6p22.3	3	16875000	2249	nofs	nofs
chr06	E	6p22.3	3	17125000	2794	nofs	nofs
chr06	E	6p22.3	3	17375000	2966	nofs	nofs
chr06	E	6p22.3	3	17625000	3433	nofs	nofs
chr06	E	6p22.3	3	17875000	5805	nofs	nofs
chr06	E	6p22.3	3	18125000	4053	nofs	nofs
chr06	E	6p22.3	3	18375000	3082	nofs	nofs
chr06	E	6p22.3	3	18625000	3160	nofs	nofs
chr06	E	6p22.3	3	18875000	5471	nofs	nofs

chr06	E	6p22.3	3	19125000	2387	nofs	nofs
chr06	E	6p22.3	3	19375000	3155	nofs	nofs
chr06	E	6p22.3	3	19625000	2665	nofs	nofs
chr06	E	6p22.3	3	19875000	2358	nofs	nofs
chr06	E	6p22.3	3	20125000	4153	nofs	nofs
chr06	E	6p22.3	3	20375000	4782	nofs	nofs
chr06	E	6p22.3	3	20625000	3202	nofs	nofs
chr06	E	6p22.3	3	20875000	2335	nofs	nofs
chr06	E	6p22.3	3	21125000	1478	nofs	nofs
chr06	E	6p22.3	3	21375000	2284	nofs	nofs
chr06	E	6p22.3	3	21625000	4140	nofs	nofs
chr06	E	6p22.3	3	21875000	5703	nofs	nofs
chr06	E	6p22.3	3	22125000	4891	nofs	nofs
chr06	E	6p22.3	3	22375000	2828	nofs	nofs
chr06	E	6p22.3	3	22625000	2755	nofs	nofs
chr06	E	6p22.3	3	22875000	3644	nofs	nofs
chr06	E	6p22.3	3	23125000	2273	nofs	nofs
chr06	E	6p22.3	3	23375000	3653	nofs	nofs
chr06	E	6p22.3	3	23625000	3033	nofs	nofs
chr06	E	6p22.3	3	23875000	3477	nofs	nofs
chr06	E	6p22.2	1	24125000	3330	fs	fs common
chr06	E	6p22.2	1	24375000	4011	fs	fs common
chr06	E	6p22.2	1	24625000	2521	fs	fs common
chr06	E	6p22.2	1	24875000	2367	fs	fs common
chr06	E	6p22.2	1	25125000	2305	fs	fs common
chr06	E	6p22.2	1	25375000	2619	fs	fs common
chr06	E	6p22.2	1	25625000	2240	fs	fs common
chr06	E	6p22.2	1	25875000	1983	fs	fs common
chr06	E	6p22.2	1	26125000	2776	fs	fs common
chr06	E	6p22.2	1	26375000	2628	fs	fs common
chr06	E	6p22.1	4	26625000	1651	nofs	nofs
chr06	E	6p22.1	4	26875000	4212	nofs	nofs
chr06	E	6p22.1	4	27125000	4706	nofs	nofs
chr06	E	6p22.1	4	27375000	4104	nofs	nofs
chr06	E	6p22.1	4	27625000	7826	nofs	nofs
chr06	E	6p22.1	4	27875000	4955	nofs	nofs

chr06	E	6p22.1	4	28125000	4237	nofs	nofs
chr06	E	6p22.1	4	28375000	3341	nofs	nofs
chr06	E	6p22.1	4	28625000	3951	nofs	nofs
chr06	E	6p22.1	4	28875000	4269	nofs	nofs
chr06	E	6p22.1	4	29125000	4434	nofs	nofs
chr06	E	6p22.1	4	29375000	1536	nofs	nofs
chr06	E	6p22.1	4	29625000	1531	nofs	nofs
chr06	E	6p22.1	4	29875000	5047	nofs	nofs
chr06	E	6p21.3	5	30125000	3096	nofs	nofs
chr06	E	6p21.3	5	30375000	2811	nofs	nofs
chr06	E	6p21.3	5	30625000	4755	nofs	nofs
chr06	E	6p21.3	5	30875000	5298	nofs	nofs
chr06	E	6p21.3	5	31125000	16075	nofs	nofs
chr06	E	6p21.3	5	31375000	2260	nofs	nofs
chr06	E	6p21.3	5	31625000	7233	nofs	nofs
chr06	E	6p21.3	5	31875000	4566	nofs	nofs
chr06	E	6p21.3	5	32125000	4296	nofs	nofs
chr06	E	6p21.3	5	32375000	4014	nofs	nofs
chr06	E	6p21.3	5	32625000	6068	nofs	nofs
chr06	E	6p21.3	5	32875000	1787	nofs	nofs
chr06	E	6p21.3	5	33125000	3045	nofs	nofs
chr06	E	6p21.3	5	33375000	5854	nofs	nofs
chr06	E	6p21.3	5	33625000	5407	nofs	nofs
chr06	E	6p21.3	5	33875000	2652	nofs	nofs
chr06	E	6p21.3	5	34125000	6915	nofs	nofs
chr06	E	6p21.3	5	34375000	6604	nofs	nofs
chr06	E	6p21.3	5	34625000	3019	nofs	nofs
chr06	E	6p21.3	5	34875000	3190	nofs	nofs
chr06	E	6p21.3	5	35125000	2269	nofs	nofs
chr06	E	6p21.3	5	35375000	5228	nofs	nofs
chr06	E	6p21.3	5	35625000	1966	nofs	nofs
chr06	E	6p21.3	5	35875000	3463	nofs	nofs
chr06	E	6p21.3	5	36125000	2285	nofs	nofs
chr06	E	6p21.3	5	36375000	3200	nofs	nofs
chr06	E	6p21.3	5	36625000	6072	nofs	nofs
chr06	E	6p21.3	5	36875000	2881	nofs	nofs

chr06	E	6p21.2	4	37125000	5179	nofs	nofs
chr06	E	6p21.2	4	37375000	3880	nofs	nofs
chr06	E	6p21.2	4	37625000	2613	nofs	nofs
chr06	E	6p21.2	4	37875000	2813	nofs	nofs
chr06	E	6p21.2	4	38125000	3120	nofs	nofs
chr06	E	6p21.2	4	38375000	1457	nofs	nofs
chr06	E	6p21.2	4	38625000	1822	nofs	nofs
chr06	E	6p21.2	4	38875000	2914	nofs	nofs
chr06	E	6p21.2	4	39125000	2840	nofs	nofs
chr06	E	6p21.2	4	39375000	2843	nofs	nofs
chr06	E	6p21.2	4	39625000	1902	nofs	nofs
chr06	E	6p21.2	4	39875000	2884	nofs	nofs
chr06	E	6p21.2	4	40125000	981	nofs	nofs
chr06	E	6p21.2	4	40375000	3095	nofs	nofs
chr06	E	6p21.2	4	40625000	4732	nofs	nofs
chr06	E	6p21.1	1	40875000	2864	nofs	nofs
chr06	E	6p21.1	1	41125000	1573	nofs	nofs
chr06	E	6p21.1	1	41375000	2530	nofs	nofs
chr06	E	6p21.1	1	41625000	3523	nofs	nofs
chr06	E	6p21.1	1	41875000	5261	nofs	nofs
chr06	E	6p21.1	1	42125000	4184	nofs	nofs
chr06	E	6p21.1	1	42375000	2679	nofs	nofs
chr06	E	6p21.1	1	42625000	4772	nofs	nofs
chr06	E	6p21.1	1	42875000	5514	nofs	nofs
chr06	E	6p21.1	1	43125000	4300	nofs	nofs
chr06	E	6p21.1	1	43375000	4090	nofs	nofs
chr06	E	6p21.1	1	43625000	5450	nofs	nofs
chr06	E	6p21.1	1	43875000	4389	nofs	nofs
chr06	E	6p21.1	1	44125000	4977	nofs	nofs
chr06	E	6p21.1	1	44375000	3081	nofs	nofs
chr06	E	6p21.1	1	44625000	2642	nofs	nofs
chr06	E	6p21.1	1	44875000	2771	nofs	nofs
chr06	E	6p21.1	1	45125000	2133	nofs	nofs
chr06	E	6p21.1	1	45375000	2547	nofs	nofs
chr06	E	6p21.1	1	45625000	2168	nofs	nofs
chr06	E	6p21.1	1	45875000	2672	nofs	nofs

chr06	E	6p21.1	1	46125000	2237	nofs	nofs
chr06	E	6p12	4	46375000	1806	nofs	nofs
chr06	E	6p12	4	46625000	3452	nofs	nofs
chr06	E	6p12	4	46875000	4354	nofs	nofs
chr06	E	6p12	4	47125000	3214	nofs	nofs
chr06	E	6p12	4	47375000	2032	nofs	nofs
chr06	E	6p12	4	47625000	3696	nofs	nofs
chr06	E	6p12	4	47875000	1996	nofs	nofs
chr06	E	6p12	4	48125000	1874	nofs	nofs
chr06	E	6p12	4	48375000	3064	nofs	nofs
chr06	E	6p12	4	48625000	2961	nofs	nofs
chr06	E	6p12	4	48875000	4691	nofs	nofs
chr06	E	6p12	4	49125000	4673	nofs	nofs
chr06	E	6p12	4	49375000	2264	nofs	nofs
chr06	E	6p12	4	49625000	3240	nofs	nofs
chr06	E	6p12	4	49875000	3557	nofs	nofs
chr06	E	6p12	4	50125000	2170	nofs	nofs
chr06	E	6p12	4	50375000	1977	nofs	nofs
chr06	E	6p12	4	50625000	2077	nofs	nofs
chr06	E	6p12	4	50875000	4034	nofs	nofs
chr06	E	6p12	4	51125000	2046	nofs	nofs
chr06	E	6p12	4	51375000	2991	nofs	nofs
chr06	E	6p12	4	51625000	2617	nofs	nofs
chr06	E	6p12	4	51875000	2359	nofs	nofs
chr06	E	6p12	4	52125000	2193	nofs	nofs
chr06	E	6p12	4	52375000	2127	nofs	nofs
chr06	E	6p12	4	52625000	3343	nofs	nofs
chr06	E	6p12	4	52875000	3937	nofs	nofs
chr06	E	6p12	4	53125000	3002	nofs	nofs
chr06	E	6p12	4	53375000	2983	nofs	nofs
chr06	E	6p12	4	53625000	3125	nofs	nofs
chr06	E	6p12	4	53875000	1136	nofs	nofs
chr06	E	6p12	4	54125000	2474	nofs	nofs
chr06	E	6p12	4	54375000	2806	nofs	nofs
chr06	E	6p12	4	54625000	2512	nofs	nofs
chr06	E	6p12	4	54875000	2625	nofs	nofs

chr06	E	6p12	4	55125000	3080	nofs	nofs
chr06	E	6p12	4	55375000	1979	nofs	nofs
chr06	E	6p12	4	55625000	2915	nofs	nofs
chr06	C	6p12	4	55875000	3876	nofs	nofs
chr06	C	6p12	4	56125000	3082	nofs	nofs
chr06	C	6p12	4	56375000	1890	nofs	nofs
chr06	C	6p12	4	56625000	1672	nofs	nofs
chr06	C	6p12	4	56875000	3939	nofs	nofs
chr06	C	6p12	4	57125000	1945	nofs	nofs
chr06	C	6p12	4	57375000	2360	nofs	nofs
chr06	C	6p11.2	2	57625000	3112	nofs	nofs
chr06	C	6p11.2	2	57875000	1188	nofs	nofs
chr06	C	6p11.2	2	58125000	31122	nofs	nofs
chr06	C	6p11.2	2	58375000	3216	nofs	nofs
chr06	C	6p11.2	2	58625000	16154	nofs	nofs
chr06	C	6p11.2	2	58875000	38012	nofs	nofs
chr06	C		.	59125000	0	nofs	nofs
chr06	C		.	59375000	0	nofs	nofs
chr06	C		.	59625000	0	nofs	nofs
chr06	C		.	59875000	0	nofs	nofs
chr06	C		.	60125000	0	nofs	nofs
chr06	C		.	60375000	0	nofs	nofs
chr06	C		.	60625000	0	nofs	nofs
chr06	C		.	60875000	0	nofs	nofs
chr06	C		.	61125000	0	nofs	nofs
chr06	C		.	61375000	0	nofs	nofs
chr06	C		.	61625000	0	nofs	nofs
chr06	C	6q11	.	61875000	60521	nofs	nofs
chr06	C	6q11	.	62125000	21126	nofs	nofs
chr06	C	6q11	.	62375000	38423	nofs	nofs
chr06	C	6q11	.	62625000	3672	nofs	nofs
chr06	C	6q11	.	62875000	1729	nofs	nofs
chr06	C	6q11	.	63125000	2053	nofs	nofs
chr06	C	6q12	2	63375000	2638	nofs	nofs
chr06	C	6q12	2	63625000	22524	nofs	nofs
chr06	C	6q12	2	63875000	3550	nofs	nofs

chr06	C	6q12	2	64125000	4437	nofs	nofs
chr06	C	6q12	2	64375000	2680	nofs	nofs
chr06	C	6q12	2	64625000	1090	nofs	nofs
chr06	C	6q12	2	64875000	1980	nofs	nofs
chr06	E	6q12	2	65125000	2349	nofs	nofs
chr06	E	6q12	2	65375000	2098	nofs	nofs
chr06	E	6q12	2	65625000	1584	nofs	nofs
chr06	E	6q12	2	65875000	2726	nofs	nofs
chr06	E	6q12	2	66125000	3267	nofs	nofs
chr06	E	6q12	2	66375000	3498	nofs	nofs
chr06	E	6q12	2	66625000	2659	nofs	nofs
chr06	E	6q12	2	66875000	2354	nofs	nofs
chr06	E	6q12	2	67125000	2861	nofs	nofs
chr06	E	6q12	2	67375000	2647	nofs	nofs
chr06	E	6q12	2	67625000	2738	nofs	nofs
chr06	E	6q12	2	67875000	4971	nofs	nofs
chr06	E	6q12	2	68125000	3814	nofs	nofs
chr06	E	6q12	2	68375000	3370	nofs	nofs
chr06	E	6q12	2	68625000	3627	nofs	nofs
chr06	E	6q12	2	68875000	3032	nofs	nofs
chr06	E	6q12	2	69125000	1362	nofs	nofs
chr06	E	6q12	2	69375000	2369	nofs	nofs
chr06	E	6q12	2	69625000	2761	nofs	nofs
chr06	E	6q12	2	69875000	3722	nofs	nofs
chr06	E	6q12	2	70125000	3555	nofs	nofs
chr06	E	6q13	3	70375000	2398	fs	fs common
chr06	E	6q13	3	70625000	3066	fs	fs common
chr06	E	6q13	3	70875000	1445	fs	fs common
chr06	E	6q13	3	71125000	1595	fs	fs common
chr06	E	6q13	3	71375000	2052	fs	fs common
chr06	E	6q13	3	71625000	1932	fs	fs common
chr06	E	6q13	3	71875000	3091	fs	fs common
chr06	E	6q13	3	72125000	3970	fs	fs common
chr06	E	6q13	3	72375000	2631	fs	fs common
chr06	E	6q13	3	72625000	2454	fs	fs common
chr06	E	6q13	3	72875000	2238	fs	fs common

chr06	E	6q13	3	73125000	2507	fs	fs common
chr06	E	6q13	3	73375000	2673	fs	fs common
chr06	E	6q13	3	73625000	2550	fs	fs common
chr06	E	6q13	3	73875000	3028	fs	fs common
chr06	E	6q13	3	74125000	4291	fs	fs common
chr06	E	6q13	3	74375000	6435	fs	fs common
chr06	E	6q13	3	74625000	2752	fs	fs common
chr06	E	6q13	3	74875000	3028	fs	fs common
chr06	E	6q13	3	75125000	2176	fs	fs common
chr06	E	6q13	3	75375000	2165	fs	fs common
chr06	E	6q13	3	75625000	2316	fs	fs common
chr06	E	6q13	3	75875000	2138	fs	fs common
chr06	E	6q14	3	76125000	2708	nofs	nofs
chr06	E	6q14	3	76375000	2653	nofs	nofs
chr06	E	6q14	3	76625000	2589	nofs	nofs
chr06	E	6q14	3	76875000	1716	nofs	nofs
chr06	E	6q14	3	77125000	1532	nofs	nofs
chr06	E	6q14	3	77375000	2376	nofs	nofs
chr06	E	6q14	3	77625000	2553	nofs	nofs
chr06	E	6q14	3	77875000	2096	nofs	nofs
chr06	E	6q14	3	78125000	1876	nofs	nofs
chr06	E	6q14	3	78375000	5021	nofs	nofs
chr06	E	6q14	3	78625000	1609	nofs	nofs
chr06	E	6q14	3	78875000	2391	nofs	nofs
chr06	E	6q14	3	79125000	2066	nofs	nofs
chr06	E	6q14	3	79375000	2093	nofs	nofs
chr06	E	6q14	3	79625000	1459	nofs	nofs
chr06	E	6q14	3	79875000	2001	nofs	nofs
chr06	E	6q14	3	80125000	1403	nofs	nofs
chr06	E	6q14	3	80375000	2167	nofs	nofs
chr06	E	6q14	3	80625000	1806	nofs	nofs
chr06	E	6q14	3	80875000	2562	nofs	nofs
chr06	E	6q14	3	81125000	1806	nofs	nofs
chr06	E	6q14	3	81375000	3198	nofs	nofs
chr06	E	6q14	3	81625000	2355	nofs	nofs
chr06	E	6q14	3	81875000	2371	nofs	nofs

chr06	E	6q14	3	82125000	3833	nofs	nofs
chr06	E	6q14	3	82375000	2313	nofs	nofs
chr06	E	6q14	3	82625000	2348	nofs	nofs
chr06	E	6q14	3	82875000	1833	nofs	nofs
chr06	E	6q14	3	83125000	2637	nofs	nofs
chr06	E	6q14	3	83375000	2414	nofs	nofs
chr06	E	6q14	3	83625000	1539	nofs	nofs
chr06	E	6q14	3	83875000	1754	nofs	nofs
chr06	E	6q14	3	84125000	3134	nofs	nofs
chr06	E	6q14	3	84375000	2118	nofs	nofs
chr06	E	6q14	3	84625000	1929	nofs	nofs
chr06	E	6q14	3	84875000	3281	nofs	nofs
chr06	E	6q14	3	85125000	2670	nofs	nofs
chr06	E	6q14	3	85375000	2967	nofs	nofs
chr06	E	6q14	3	85625000	2085	nofs	nofs
chr06	E	6q14	3	85875000	1838	nofs	nofs
chr06	E	6q14	3	86125000	2598	nofs	nofs
chr06	E	6q14	3	86375000	2073	nofs	nofs
chr06	E	6q14	3	86625000	2728	nofs	nofs
chr06	E	6q14	3	86875000	1610	nofs	nofs
chr06	E	6q14	3	87125000	2507	nofs	nofs
chr06	E	6q14	3	87375000	3401	nofs	nofs
chr06	E	6q15	1	87625000	2198	fs	fs common
chr06	E	6q15	1	87875000	4825	fs	fs common
chr06	E	6q15	1	88125000	2205	fs	fs common
chr06	E	6q15	1	88375000	2490	fs	fs common
chr06	E	6q15	1	88625000	3706	fs	fs common
chr06	E	6q15	1	88875000	2194	fs	fs common
chr06	E	6q15	1	89125000	2219	fs	fs common
chr06	E	6q15	1	89375000	2126	fs	fs common
chr06	E	6q15	1	89625000	4065	fs	fs common
chr06	E	6q15	1	89875000	3754	fs	fs common
chr06	E	6q15	1	90125000	3080	fs	fs common
chr06	E	6q15	1	90375000	1403	fs	fs common
chr06	E	6q15	1	90625000	3197	fs	fs common
chr06	E	6q15	1	90875000	1542	fs	fs common

chr06	E	6q15	1	91125000	2354	fs	fs common
chr06	E	6q15	1	91375000	1435	fs	fs common
chr06	E	6q15	1	91625000	2491	fs	fs common
chr06	E	6q15	1	91875000	1991	fs	fs common
chr06	E	6q15	1	92125000	2377	fs	fs common
chr06	E	6q16.1	1	92375000	2312	nofs	nofs
chr06	E	6q16.1	1	92625000	2111	nofs	nofs
chr06	E	6q16.1	1	92875000	3352	nofs	nofs
chr06	E	6q16.1	1	93125000	2576	nofs	nofs
chr06	E	6q16.1	1	93375000	2573	nofs	nofs
chr06	E	6q16.1	1	93625000	1663	nofs	nofs
chr06	E	6q16.1	1	93875000	1773	nofs	nofs
chr06	E	6q16.1	1	94125000	1631	nofs	nofs
chr06	E	6q16.1	1	94375000	1863	nofs	nofs
chr06	E	6q16.1	1	94625000	2067	nofs	nofs
chr06	E	6q16.1	1	94875000	2490	nofs	nofs
chr06	E	6q16.1	1	95125000	2445	nofs	nofs
chr06	E	6q16.1	1	95375000	2969	nofs	nofs
chr06	E	6q16.1	1	95625000	2234	nofs	nofs
chr06	E	6q16.1	1	95875000	436	nofs	nofs
chr06	E	6q16.1	1	96125000	4400	nofs	nofs
chr06	E	6q16.1	1	96375000	1636	nofs	nofs
chr06	E	6q16.1	1	96625000	1880	nofs	nofs
chr06	E	6q16.1	1	96875000	1722	nofs	nofs
chr06	E	6q16.1	1	97125000	1582	nofs	nofs
chr06	E	6q16.1	1	97375000	2302	nofs	nofs
chr06	E	6q16.1	1	97625000	1178	nofs	nofs
chr06	E	6q16.1	1	97875000	1761	nofs	nofs
chr06	E	6q16.1	1	98125000	2358	nofs	nofs
chr06	E	6q16.1	1	98375000	2130	nofs	nofs
chr06	E	6q16.1	1	98625000	1800	nofs	nofs
chr06	B	6q16.2	.	98875000	2828	nofs	nofs
chr06	B	6q16.2	.	99125000	2135	nofs	nofs
chr06	B	6q16.2	.	99375000	1705	nofs	nofs
chr06	B	6q16.2	.	99625000	2734	nofs	nofs
chr06	B	6q16.2	.	99875000	2272	nofs	nofs

chr06	E	6q16.3	2	100000000	2540	nofs	nofs
chr06	E	6q16.3	2	100000000	1966	nofs	nofs
chr06	E	6q16.3	2	101000000	1361	nofs	nofs
chr06	E	6q16.3	2	101000000	2821	nofs	nofs
chr06	E	6q16.3	2	101000000	2335	nofs	nofs
chr06	E	6q16.3	2	101000000	1889	nofs	nofs
chr06	E	6q16.3	2	102000000	3839	nofs	nofs
chr06	E	6q16.3	2	102000000	2507	nofs	nofs
chr06	E	6q16.3	2	102000000	2230	nofs	nofs
chr06	E	6q16.3	2	102000000	2980	nofs	nofs
chr06	E	6q16.3	2	103000000	2633	nofs	nofs
chr06	E	6q16.3	2	103000000	3718	nofs	nofs
chr06	E	6q16.3	2	103000000	3446	nofs	nofs
chr06	E	6q16.3	2	103000000	2628	nofs	nofs
chr06	E	6q16.3	2	104000000	2082	nofs	nofs
chr06	E	6q16.3	2	104000000	2709	nofs	nofs
chr06	E	6q16.3	2	104000000	11647	nofs	nofs
chr06	E	6q16.3	2	104000000	3518	nofs	nofs
chr06	E	6q16.3	2	105000000	1375	nofs	nofs
chr06	E	6q16.3	2	105000000	1934	nofs	nofs
chr06	E	6q16.3	2	105000000	2459	nofs	nofs
chr06	B	6q21	.	105000000	3261	fs	fs common
chr06	B	6q21	.	106000000	3505	fs	fs common
chr06	B	6q21	.	106000000	1693	fs	fs common
chr06	B	6q21	.	106000000	1142	fs	fs common
chr06	B	6q21	.	106000000	3441	fs	fs common
chr06	B	6q21	.	107000000	2610	fs	fs common
chr06	B	6q21	.	107000000	2370	fs	fs common
chr06	B	6q21	.	107000000	2254	fs	fs common
chr06	B	6q21	.	107000000	4353	fs	fs common
chr06	B	6q21	.	108000000	4156	fs	fs common
chr06	B	6q21	.	108000000	1701	fs	fs common
chr06	B	6q21	.	108000000	3005	fs	fs common
chr06	B	6q21	.	108000000	4592	fs	fs common
chr06	B	6q21	.	109000000	3201	fs	fs common
chr06	B	6q21	.	109000000	3226	fs	fs common

chr06	B	6q21	.	109000000	1440	fs	fs common
chr06	B	6q21	.	109000000	4625	fs	fs common
chr06	B	6q21	.	110000000	2110	fs	fs common
chr06	B	6q21	.	110000000	1755	fs	fs common
chr06	B	6q21	.	110000000	2048	fs	fs common
chr06	B	6q21	.	110000000	3397	fs	fs common
chr06	B	6q21	.	111000000	2268	fs	fs common
chr06	B	6q21	.	111000000	2362	fs	fs common
chr06	B	6q21	.	111000000	2462	fs	fs common
chr06	B	6q21	.	111000000	3346	fs	fs common
chr06	B	6q21	.	112000000	4051	fs	fs common
chr06	B	6q21	.	112000000	1809	fs	fs common
chr06	B	6q21	.	112000000	2235	fs	fs common
chr06	B	6q21	.	112000000	3458	fs	fs common
chr06	B	6q21	.	113000000	3080	fs	fs common
chr06	B	6q21	.	113000000	2714	fs	fs common
chr06	B	6q21	.	113000000	3388	fs	fs common
chr06	B	6q21	.	113000000	2230	fs	fs common
chr06	B	6q21	.	114000000	2548	fs	fs common
chr06	B	6q21	.	114000000	2818	fs	fs common
chr06	B	6q21	.	114000000	6151	fs	fs common
chr06	B	6q21	.	114000000	2899	fs	fs common
chr06	E	6q22.1	4	115000000	979	nofs	nofs
chr06	E	6q22.1	4	115000000	4187	nofs	nofs
chr06	E	6q22.1	4	115000000	1860	nofs	nofs
chr06	E	6q22.1	4	115000000	2831	nofs	nofs
chr06	E	6q22.1	4	116000000	2669	nofs	nofs
chr06	E	6q22.1	4	116000000	2330	nofs	nofs
chr06	E	6q22.1	4	116000000	1281	nofs	nofs
chr06	E	6q22.1	4	116000000	3658	nofs	nofs
chr06	E	6q22.1	4	117000000	2155	nofs	nofs
chr06	E	6q22.1	4	117000000	1719	nofs	nofs
chr06	E	6q22.1	4	117000000	2401	nofs	nofs
chr06	E	6q22.1	4	117000000	1844	nofs	nofs
chr06	E	6q22.1	4	118000000	1686	nofs	nofs
chr06	E	6q22.1	4	118000000	1513	nofs	nofs

chr06	E	6q22.1	4	118000000	3175	nofs	nofs
chr06	E	6q22.1	4	118000000	3001	nofs	nofs
chr06	B	6q22.2	.	119000000	1928	nofs	nofs
chr06	E	6q22.3	4	119000000	1832	nofs	nofs
chr06	E	6q22.3	4	119000000	2516	nofs	nofs
chr06	E	6q22.3	4	119000000	2216	nofs	nofs
chr06	E	6q22.3	4	120000000	1734	nofs	nofs
chr06	E	6q22.3	4	120000000	2155	nofs	nofs
chr06	E	6q22.3	4	120000000	3281	nofs	nofs
chr06	E	6q22.3	4	120000000	1407	nofs	nofs
chr06	E	6q22.3	4	121000000	2667	nofs	nofs
chr06	E	6q22.3	4	121000000	1666	nofs	nofs
chr06	E	6q22.3	4	121000000	2689	nofs	nofs
chr06	E	6q22.3	4	121000000	2304	nofs	nofs
chr06	E	6q22.3	4	122000000	1619	nofs	nofs
chr06	E	6q22.3	4	122000000	3310	nofs	nofs
chr06	E	6q22.3	4	122000000	2824	nofs	nofs
chr06	E	6q22.3	4	122000000	2427	nofs	nofs
chr06	E	6q22.3	4	123000000	2105	nofs	nofs
chr06	E	6q22.3	4	123000000	1939	nofs	nofs
chr06	E	6q22.3	4	123000000	4512	nofs	nofs
chr06	E	6q22.3	4	123000000	2333	nofs	nofs
chr06	E	6q22.3	4	124000000	3218	nofs	nofs
chr06	E	6q22.3	4	124000000	2360	nofs	nofs
chr06	E	6q22.3	4	124000000	1754	nofs	nofs
chr06	E	6q22.3	4	124000000	2694	nofs	nofs
chr06	E	6q22.3	4	125000000	2182	nofs	nofs
chr06	E	6q22.3	4	125000000	1852	nofs	nofs
chr06	E	6q22.3	4	125000000	1849	nofs	nofs
chr06	E	6q22.3	4	125000000	3115	nofs	nofs
chr06	E	6q22.3	4	126000000	2749	nofs	nofs
chr06	E	6q22.3	4	126000000	3318	nofs	nofs
chr06	E	6q22.3	4	126000000	2858	nofs	nofs
chr06	E	6q22.3	4	126000000	890	nofs	nofs
chr06	E	6q22.3	4	127000000	2289	nofs	nofs
chr06	E	6q22.3	4	127000000	2620	nofs	nofs

chr06	E	6q22.3	4	127000000	2540	nofs	nofs
chr06	E	6q22.3	4	127000000	1369	nofs	nofs
chr06	E	6q22.3	4	128000000	1539	nofs	nofs
chr06	E	6q22.3	4	128000000	2210	nofs	nofs
chr06	E	6q22.3	4	128000000	2672	nofs	nofs
chr06	E	6q22.3	4	128000000	1338	nofs	nofs
chr06	E	6q22.3	4	129000000	1216	nofs	nofs
chr06	E	6q22.3	4	129000000	3589	nofs	nofs
chr06	E	6q22.3	4	129000000	1543	nofs	nofs
chr06	E	6q22.3	4	129000000	1979	nofs	nofs
chr06	E	6q22.3	4	130000000	3209	nofs	nofs
chr06	E	6q22.3	4	130000000	2805	nofs	nofs
chr06	E	6q22.3	4	130000000	2047	nofs	nofs
chr06	E	6q22.3	4	130000000	2326	nofs	nofs
chr06	B	6q23.1	.	131000000	2255	nofs	nofs
chr06	B	6q23.1	.	131000000	1121	nofs	nofs
chr06	B	6q23.1	.	131000000	2539	nofs	nofs
chr06	B	6q23.1	.	131000000	1652	nofs	nofs
chr06	E	6q23.2	2	132000000	3060	nofs	nofs
chr06	E	6q23.2	2	132000000	2692	nofs	nofs
chr06	E	6q23.2	2	132000000	3333	nofs	nofs
chr06	E	6q23.2	2	132000000	2704	nofs	nofs
chr06	E	6q23.2	2	133000000	2781	nofs	nofs
chr06	E	6q23.2	2	133000000	1777	nofs	nofs
chr06	E	6q23.2	2	133000000	2296	nofs	nofs
chr06	E	6q23.2	2	133000000	2142	nofs	nofs
chr06	E	6q23.2	2	134000000	2302	nofs	nofs
chr06	E	6q23.2	2	134000000	2051	nofs	nofs
chr06	E	6q23.2	2	134000000	3631	nofs	nofs
chr06	E	6q23.2	2	134000000	2613	nofs	nofs
chr06	E	6q23.2	2	135000000	4934	nofs	nofs
chr06	E	6q23.2	2	135000000	4374	nofs	nofs
chr06	E	6q23.2	2	135000000	2588	nofs	nofs
chr06	E	6q23.2	2	135000000	2745	nofs	nofs
chr06	B	6q23.3	.	136000000	3243	nofs	nofs
chr06	B	6q23.3	.	136000000	2792	nofs	nofs

chr06	B	6q23.3	.	136000000	2677	nofs	nofs
chr06	B	6q23.3	.	136000000	2800	nofs	nofs
chr06	B	6q23.3	.	137000000	2622	nofs	nofs
chr06	B	6q23.3	.	137000000	2059	nofs	nofs
chr06	B	6q23.3	.	137000000	1477	nofs	nofs
chr06	B	6q23.3	.	137000000	3961	nofs	nofs
chr06	B	6q23.3	.	138000000	2253	nofs	nofs
chr06	B	6q23.3	.	138000000	2737	nofs	nofs
chr06	B	6q23.3	.	138000000	2420	nofs	nofs
chr06	B	6q23.3	.	138000000	3528	nofs	nofs
chr06	B	6q23.3	.	139000000	2564	nofs	nofs
chr06	B	6q23.3	.	139000000	2117	nofs	nofs
chr06	E	6q24	1	139000000	1779	nofs	nofs
chr06	E	6q24	1	139000000	3289	nofs	nofs
chr06	E	6q24	1	140000000	1949	nofs	nofs
chr06	E	6q24	1	140000000	4425	nofs	nofs
chr06	E	6q24	1	140000000	1959	nofs	nofs
chr06	E	6q24	1	140000000	1934	nofs	nofs
chr06	E	6q24	1	141000000	2771	nofs	nofs
chr06	E	6q24	1	141000000	2479	nofs	nofs
chr06	E	6q24	1	141000000	5500	nofs	nofs
chr06	E	6q24	1	141000000	2964	nofs	nofs
chr06	E	6q24	1	142000000	3995	nofs	nofs
chr06	E	6q24	1	142000000	2812	nofs	nofs
chr06	E	6q24	1	142000000	1899	nofs	nofs
chr06	E	6q24	1	142000000	2998	nofs	nofs
chr06	E	6q24	1	143000000	2858	nofs	nofs
chr06	E	6q24	1	143000000	1868	nofs	nofs
chr06	E	6q24	1	143000000	2558	nofs	nofs
chr06	E	6q24	1	143000000	1708	nofs	nofs
chr06	E	6q24	1	144000000	1865	nofs	nofs
chr06	E	6q24	1	144000000	2227	nofs	nofs
chr06	E	6q24	1	144000000	3862	nofs	nofs
chr06	E	6q24	1	144000000	2683	nofs	nofs
chr06	E	6q24	1	145000000	2676	nofs	nofs
chr06	E	6q24	1	145000000	3904	nofs	nofs

chr06	E	6q24	1	145000000	2921	nofs	nofs
chr06	E	6q24	1	145000000	2989	nofs	nofs
chr06	E	6q24	1	146000000	2025	nofs	nofs
chr06	E	6q24	1	146000000	1570	nofs	nofs
chr06	E	6q24	1	146000000	1275	nofs	nofs
chr06	E	6q24	1	146000000	1077	nofs	nofs
chr06	E	6q24	1	147000000	2062	nofs	nofs
chr06	E	6q24	1	147000000	2710	nofs	nofs
chr06	E	6q24	1	147000000	2316	nofs	nofs
chr06	E	6q24	1	147000000	1522	nofs	nofs
chr06	E	6q24	1	148000000	1871	nofs	nofs
chr06	E	6q24	1	148000000	2477	nofs	nofs
chr06	E	6q24	1	148000000	1452	nofs	nofs
chr06	E	6q24	1	148000000	1878	nofs	nofs
chr06	E	6q24	1	149000000	4598	nofs	nofs
chr06	E	6q24	1	149000000	4171	nofs	nofs
chr06	E	6q24	1	149000000	4905	nofs	nofs
chr06	E	6q25.1	2	149000000	2389	nofs	nofs
chr06	E	6q25.1	2	150000000	3022	nofs	nofs
chr06	E	6q25.1	2	150000000	3576	nofs	nofs
chr06	E	6q25.1	2	150000000	5901	nofs	nofs
chr06	E	6q25.1	2	150000000	6698	nofs	nofs
chr06	E	6q25.1	2	151000000	5279	nofs	nofs
chr06	E	6q25.1	2	151000000	3513	nofs	nofs
chr06	E	6q25.1	2	151000000	3303	nofs	nofs
chr06	E	6q25.1	2	151000000	3355	nofs	nofs
chr06	E	6q25.1	2	152000000	4468	nofs	nofs
chr06	E	6q25.1	2	152000000	6155	nofs	nofs
chr06	E	6q25.1	2	152000000	1927	nofs	nofs
chr06	E	6q25.1	2	152000000	3920	nofs	nofs
chr06	E	6q25.1	2	153000000	3159	nofs	nofs
chr06	E	6q25.2	1	153000000	3081	nofs	nofs
chr06	E	6q25.2	1	153000000	3302	nofs	nofs
chr06	E	6q25.2	1	153000000	2302	nofs	nofs
chr06	E	6q25.2	1	154000000	2431	nofs	nofs
chr06	E	6q25.2	1	154000000	2346	nofs	nofs

chr06	E	6q25.2	1	154000000	2158	nofs	nofs
chr06	E	6q25.2	1	154000000	3154	nofs	nofs
chr06	E	6q25.2	1	155000000	3272	nofs	nofs
chr06	E	6q25.2	1	155000000	5646	nofs	nofs
chr06	E	6q25.2	1	155000000	3141	nofs	nofs
chr06	E	6q25.2	1	155000000	6722	nofs	nofs
chr06	E	6q25.2	1	156000000	3872	nofs	nofs
chr06	E	6q25.3	2	156000000	1570	nofs	nofs
chr06	E	6q25.3	2	156000000	5016	nofs	nofs
chr06	E	6q25.3	2	156000000	1622	nofs	nofs
chr06	E	6q25.3	2	157000000	2952	nofs	nofs
chr06	E	6q25.3	2	157000000	3199	nofs	nofs
chr06	E	6q25.3	2	157000000	3504	nofs	nofs
chr06	E	6q25.3	2	157000000	2748	nofs	nofs
chr06	E	6q25.3	2	158000000	5921	nofs	nofs
chr06	E	6q25.3	2	158000000	4445	nofs	nofs
chr06	E	6q25.3	2	158000000	5577	nofs	nofs
chr06	E	6q25.3	2	158000000	4191	nofs	nofs
chr06	E	6q25.3	2	159000000	9381	nofs	nofs
chr06	E	6q25.3	2	159000000	3449	nofs	nofs
chr06	E	6q25.3	2	159000000	4115	nofs	nofs
chr06	E	6q25.3	2	159000000	4240	nofs	nofs
chr06	E	6q25.3	2	160000000	4359	nofs	nofs
chr06	E	6q25.3	2	160000000	4428	nofs	nofs
chr06	E	6q25.3	2	160000000	6852	nofs	nofs
chr06	E	6q25.3	2	160000000	6239	nofs	nofs
chr06	E	6q25.3	2	161000000	6653	nofs	nofs
chr06	E	6q25.3	2	161000000	3212	nofs	nofs
chr06	B	6q26	.	161000000	21107	fs	fs common
chr06	B	6q26	.	161000000	2939	fs	fs common
chr06	B	6q26	.	162000000	5259	fs	fs common
chr06	B	6q26	.	162000000	5656	fs	fs common
chr06	B	6q26	.	162000000	5158	fs	fs common
chr06	B	6q26	.	162000000	5764	fs	fs common
chr06	B	6q26	.	163000000	3765	fs	fs common
chr06	B	6q26	.	163000000	3663	fs	fs common

chr06	B	6q26	.	163000000	2606	fs	fs common
chr06	B	6q26	.	163000000	4856	fs	fs common
chr06	B	6q26	.	164000000	4034	fs	fs common
chr06	B	6q26	.	164000000	1934	fs	fs common
chr06	B	6q26	.	164000000	3854	fs	fs common
chr06	B	6q26	.	164000000	3958	fs	fs common
chr06	B	6q26	.	165000000	3292	fs	fs common
chr06	E	6q27	2	165000000	1539	nofs	nofs
chr06	E	6q27	2	165000000	4287	nofs	nofs
chr06	E	6q27	2	165000000	5860	nofs	nofs
chr06	E	6q27	2	166000000	4211	nofs	nofs
chr06	E	6q27	2	166000000	1973	nofs	nofs
chr06	E	6q27	2	166000000	7028	nofs	nofs
chr06	E	6q27	2	166000000	6631	nofs	nofs
chr06	E	6q27	2	167000000	6516	nofs	nofs
chr06	E	6q27	2	167000000	7987	nofs	nofs
chr06	E	6q27	2	167000000	7741	nofs	nofs
chr06	E	6q27	2	167000000	6854	nofs	nofs
chr06	T	6q27	2	168000000	6566	nofs	nofs
chr06	T	6q27	2	168000000	17292	nofs	nofs
chr06	T	6q27	2	168000000	8856	nofs	nofs
chr06	T	6q27	2	168000000	19659	nofs	nofs
chr06	T	6q27	2	169000000	23797	nofs	nofs
chr06	T	6q27	2	169000000	23694	nofs	nofs
chr06	T	6q27	2	169000000	17819	nofs	nofs
chr06	T	6q27	2	169000000	17863	nofs	nofs
chr06	T	6q27	2	170000000	12375	nofs	nofs
chr06	T	6q27	2	170000000	9548	nofs	nofs
chr06	T	6q27	2	170000000	12533	nofs	nofs
chr06	T	6q27	2	170000000	36649	nofs	nofs
chr06	T	6q27	2	171000000	22576	nofs	nofs
chr07	T	7p22	4	125000	11950	fs	fs common
chr07	T	7p22	4	375000	13457	fs	fs common
chr07	T	7p22	4	625000	22931	fs	fs common
chr07	T	7p22	4	875000	30462	fs	fs common
chr07	T	7p22	4	1125000	21149	fs	fs common

chr07	T	7p22	4	1375000	13048	fs	fs common
chr07	T	7p22	4	1625000	17409	fs	fs common
chr07	T	7p22	4	1875000	18889	fs	fs common
chr07	T	7p22	4	2125000	13408	fs	fs common
chr07	T	7p22	4	2375000	13358	fs	fs common
chr07	T	7p22	4	2625000	6751	fs	fs common
chr07	T	7p22	4	2875000	6098	fs	fs common
chr07	T	7p22	4	3125000	3559	fs	fs common
chr07	E	7p22	4	3375000	4080	fs	fs common
chr07	E	7p22	4	3625000	3353	fs	fs common
chr07	E	7p22	4	3875000	10139	fs	fs common
chr07	E	7p22	4	4125000	12583	fs	fs common
chr07	E	7p22	4	4375000	8263	fs	fs common
chr07	E	7p22	4	4625000	9423	fs	fs common
chr07	E	7p22	4	4875000	6697	fs	fs common
chr07	E	7p22	4	5125000	4578	fs	fs common
chr07	E	7p22	4	5375000	9035	fs	fs common
chr07	E	7p22	4	5625000	6187	fs	fs common
chr07	E	7p22	4	5875000	5602	fs	fs common
chr07	E	7p22	4	6125000	6226	fs	fs common
chr07	E	7p22	4	6375000	6809	fs	fs common
chr07	E	7p22	4	6625000	10267	fs	fs common
chr07	E	7p22	4	6875000	19163	fs	fs common
chr07	E	7p21	4	7125000	1123	nofs	nofs
chr07	E	7p21	4	7375000	3543	nofs	nofs
chr07	E	7p21	4	7625000	1617	nofs	nofs
chr07	E	7p21	4	7875000	2486	nofs	nofs
chr07	E	7p21	4	8125000	2324	nofs	nofs
chr07	E	7p21	4	8375000	1021	nofs	nofs
chr07	E	7p21	4	8625000	1961	nofs	nofs
chr07	E	7p21	4	8875000	2080	nofs	nofs
chr07	E	7p21	4	9125000	1680	nofs	nofs
chr07	E	7p21	4	9375000	1858	nofs	nofs
chr07	E	7p21	4	9625000	4070	nofs	nofs
chr07	E	7p21	4	9875000	2495	nofs	nofs
chr07	E	7p21	4	10125000	2059	nofs	nofs

chr07	E	7p21	4	10375000	4290	nofs	nofs
chr07	E	7p21	4	10625000	2107	nofs	nofs
chr07	E	7p21	4	10875000	3688	nofs	nofs
chr07	E	7p21	4	11125000	1308	nofs	nofs
chr07	E	7p21	4	11375000	1798	nofs	nofs
chr07	E	7p21	4	11625000	1870	nofs	nofs
chr07	E	7p21	4	11875000	1794	nofs	nofs
chr07	E	7p21	4	12125000	1623	nofs	nofs
chr07	E	7p21	4	12375000	5298	nofs	nofs
chr07	E	7p21	4	12625000	3431	nofs	nofs
chr07	E	7p21	4	12875000	3724	nofs	nofs
chr07	E	7p21	4	13125000	3462	nofs	nofs
chr07	E	7p21	4	13375000	2768	nofs	nofs
chr07	E	7p21	4	13625000	2407	nofs	nofs
chr07	E	7p21	4	13875000	1494	nofs	nofs
chr07	E	7p21	4	14125000	3033	nofs	nofs
chr07	E	7p21	4	14375000	2342	nofs	nofs
chr07	E	7p21	4	14625000	2755	nofs	nofs
chr07	E	7p21	4	14875000	3903	nofs	nofs
chr07	E	7p21	4	15125000	3194	nofs	nofs
chr07	E	7p21	4	15375000	3615	nofs	nofs
chr07	E	7p21	4	15625000	2519	nofs	nofs
chr07	E	7p21	4	15875000	2652	nofs	nofs
chr07	E	7p21	4	16125000	1671	nofs	nofs
chr07	E	7p21	4	16375000	1844	nofs	nofs
chr07	E	7p21	4	16625000	3496	nofs	nofs
chr07	E	7p21	4	16875000	2367	nofs	nofs
chr07	E	7p21	4	17125000	2336	nofs	nofs
chr07	E	7p21	4	17375000	1224	nofs	nofs
chr07	E	7p21	4	17625000	2262	nofs	nofs
chr07	E	7p21	4	17875000	2253	nofs	nofs
chr07	E	7p21	4	18125000	2115	nofs	nofs
chr07	E	7p21	4	18375000	2124	nofs	nofs
chr07	E	7p21	4	18625000	3173	nofs	nofs
chr07	E	7p21	4	18875000	1663	nofs	nofs
chr07	E	7p21	4	19125000	3009	nofs	nofs

chr07	E	7p21	4	19375000	3205	nofs	nofs
chr07	E	7p21	4	19625000	1844	nofs	nofs
chr07	E	7p21	4	19875000	2478	nofs	nofs
chr07	E	7p21	4	20125000	3698	nofs	nofs
chr07	E	7p21	4	20375000	4020	nofs	nofs
chr07	E	7p21	4	20625000	2801	nofs	nofs
chr07	E	7p15.3	4	20875000	1888	nofs	nofs
chr07	E	7p15.3	4	21125000	2729	nofs	nofs
chr07	E	7p15.3	4	21375000	2833	nofs	nofs
chr07	E	7p15.3	4	21625000	2036	nofs	nofs
chr07	E	7p15.3	4	21875000	2568	nofs	nofs
chr07	E	7p15.3	4	22125000	1816	nofs	nofs
chr07	E	7p15.3	4	22375000	1914	nofs	nofs
chr07	E	7p15.3	4	22625000	2910	nofs	nofs
chr07	E	7p15.3	4	22875000	4661	nofs	nofs
chr07	E	7p15.3	4	23125000	4055	nofs	nofs
chr07	E	7p15.3	4	23375000	5839	nofs	nofs
chr07	E	7p15.3	4	23625000	3285	nofs	nofs
chr07	E	7p15.3	4	23875000	1887	nofs	nofs
chr07	E	7p15.3	4	24125000	2864	nofs	nofs
chr07	E	7p15.3	4	24375000	1653	nofs	nofs
chr07	E	7p15.3	4	24625000	1678	nofs	nofs
chr07	E	7p15.3	4	24875000	3427	nofs	nofs
chr07	E	7p15.3	4	25125000	2351	nofs	nofs
chr07	E	7p15.3	4	25375000	2216	nofs	nofs
chr07	E	7p15.2	1	25625000	2185	nofs	nofs
chr07	E	7p15.2	1	25875000	3727	nofs	nofs
chr07	E	7p15.2	1	26125000	3676	nofs	nofs
chr07	E	7p15.2	1	26375000	2456	nofs	nofs
chr07	E	7p15.2	1	26625000	2225	nofs	nofs
chr07	E	7p15.2	1	26875000	3810	nofs	nofs
chr07	E	7p15.2	1	27125000	2718	nofs	nofs
chr07	E	7p15.2	1	27375000	1643	nofs	nofs
chr07	E	7p15.2	1	27625000	2406	nofs	nofs
chr07	B	7p15.1	.	27875000	2725	nofs	nofs
chr07	B	7p15.1	.	28125000	2121	nofs	nofs

chr07	B	7p15.1	.	28375000	2342	nofs	nofs
chr07	B	7p15.1	.	28625000	2620	nofs	nofs
chr07	B	7p15.1	.	28875000	1627	nofs	nofs
chr07	B	7p15.1	.	29125000	3095	nofs	nofs
chr07	B	7p15.1	.	29375000	2965	nofs	nofs
chr07	B	7p15.1	.	29625000	3536	nofs	nofs
chr07	B	7p15.1	.	29875000	2689	nofs	nofs
chr07	B	7p15.1	.	30125000	2124	nofs	nofs
chr07	E	7p14	3	30375000	3641	nofs	nofs
chr07	E	7p14	3	30625000	3360	nofs	nofs
chr07	E	7p14	3	30875000	3100	nofs	nofs
chr07	E	7p14	3	31125000	2887	nofs	nofs
chr07	E	7p14	3	31375000	1758	nofs	nofs
chr07	E	7p14	3	31625000	2507	nofs	nofs
chr07	E	7p14	3	31875000	2224	nofs	nofs
chr07	E	7p14	3	32125000	3031	nofs	nofs
chr07	E	7p14	3	32375000	4454	nofs	nofs
chr07	E	7p14	3	32625000	2998	nofs	nofs
chr07	E	7p14	3	32875000	3534	nofs	nofs
chr07	E	7p14	3	33125000	2702	nofs	nofs
chr07	E	7p14	3	33375000	1927	nofs	nofs
chr07	E	7p14	3	33625000	2644	nofs	nofs
chr07	E	7p14	3	33875000	2104	nofs	nofs
chr07	E	7p14	3	34125000	1991	nofs	nofs
chr07	E	7p14	3	34375000	1673	nofs	nofs
chr07	E	7p14	3	34625000	2067	fs	fs common
chr07	E	7p14	3	34875000	3790	fs	fs common
chr07	E	7p14	3	35125000	2834	fs	fs common
chr07	E	7p14	3	35375000	3457	fs	fs common
chr07	E	7p14	3	35625000	2482	fs	fs common
chr07	E	7p14	3	35875000	1666	fs	fs common
chr07	E	7p14	3	36125000	4640	fs	fs common
chr07	E	7p14	3	36375000	4402	fs	fs common
chr07	E	7p14	3	36625000	2259	fs	fs common
chr07	E	7p14	3	36875000	1816	fs	fs common
chr07	E	7p14	3	37125000	4133	fs	fs common

chr07	E	7p14	3	37375000	2645	nofs	nofs
chr07	E	7p14	3	37625000	7857	nofs	nofs
chr07	E	7p14	3	37875000	3216	nofs	nofs
chr07	E	7p14	3	38125000	2439	nofs	nofs
chr07	E	7p14	3	38375000	3028	nofs	nofs
chr07	E	7p14	3	38625000	1465	nofs	nofs
chr07	E	7p14	3	38875000	3241	nofs	nofs
chr07	E	7p14	3	39125000	2312	nofs	nofs
chr07	E	7p14	3	39375000	3963	nofs	nofs
chr07	E	7p14	3	39625000	3196	nofs	nofs
chr07	E	7p14	3	39875000	2548	nofs	nofs
chr07	E	7p14	3	40125000	4247	nofs	nofs
chr07	E	7p14	3	40375000	2092	nofs	nofs
chr07	E	7p14	3	40625000	1971	nofs	nofs
chr07	E	7p14	3	40875000	2529	nofs	nofs
chr07	E	7p14	3	41125000	3962	nofs	nofs
chr07	E	7p14	3	41375000	2045	nofs	nofs
chr07	E	7p14	3	41625000	2050	nofs	nofs
chr07	E	7p14	3	41875000	1875	nofs	nofs
chr07	E	7p14	3	42125000	2776	nofs	nofs
chr07	E	7p14	3	42375000	1725	nofs	nofs
chr07	E	7p14	3	42625000	2790	nofs	nofs
chr07	E	7p14	3	42875000	1577	nofs	nofs
chr07	E	7p14	3	43125000	3581	nofs	nofs
chr07	E	7p13	3	43375000	1864	fs	fs common
chr07	E	7p13	3	43625000	4068	fs	fs common
chr07	E	7p13	3	43875000	3927	fs	fs common
chr07	E	7p13	3	44125000	3772	fs	fs common
chr07	E	7p13	3	44375000	3484	fs	fs common
chr07	E	7p13	3	44625000	7581	fs	fs common
chr07	E	7p13	3	44875000	6061	fs	fs common
chr07	E	7p13	3	45125000	3010	fs	fs common
chr07	E	7p13	3	45375000	3424	fs	fs common
chr07	E	7p13	3	45625000	5572	fs	fs common
chr07	E	7p13	3	45875000	1659	fs	fs common
chr07	E	7p13	3	46125000	2099	fs	fs common

chr07	E	7p13	3	46375000	2293	fs	fs common
chr07	E	7p13	3	46625000	2000	fs	fs common
chr07	E	7p13	3	46875000	4288	fs	fs common
chr07	E	7p12	2	47125000	3993	nofs	nofs
chr07	E	7p12	2	47375000	5631	nofs	nofs
chr07	E	7p12	2	47625000	3499	nofs	nofs
chr07	E	7p12	2	47875000	4435	nofs	nofs
chr07	E	7p12	2	48125000	3937	nofs	nofs
chr07	E	7p12	2	48375000	2323	nofs	nofs
chr07	E	7p12	2	48625000	2070	nofs	nofs
chr07	E	7p12	2	48875000	1609	nofs	nofs
chr07	E	7p12	2	49125000	1841	nofs	nofs
chr07	E	7p12	2	49375000	4044	nofs	nofs
chr07	E	7p12	2	49625000	3462	nofs	nofs
chr07	E	7p12	2	49875000	5259	nofs	nofs
chr07	E	7p12	2	50125000	2655	nofs	nofs
chr07	E	7p12	2	50375000	3959	nofs	nofs
chr07	E	7p12	2	50625000	2137	nofs	nofs
chr07	E	7p12	2	50875000	1964	nofs	nofs
chr07	E	7p12	2	51125000	4028	nofs	nofs
chr07	E	7p12	2	51375000	2595	nofs	nofs
chr07	E	7p12	2	51625000	2583	nofs	nofs
chr07	E	7p12	2	51875000	2805	nofs	nofs
chr07	E	7p12	2	52125000	5138	nofs	nofs
chr07	E	7p12	2	52375000	2715	nofs	nofs
chr07	E	7p12	2	52625000	3597	nofs	nofs
chr07	E	7p12	2	52875000	2849	nofs	nofs
chr07	E	7p12	2	53125000	2862	nofs	nofs
chr07	E	7p12	2	53375000	2330	nofs	nofs
chr07	E	7p12	2	53625000	3222	nofs	nofs
chr07	E	7p11.2	3	53875000	2523	fs	fs rare
chr07	E	7p11.2	3	54125000	4655	fs	fs rare
chr07	E	7p11.2	3	54375000	3380	fs	fs rare
chr07	E	7p11.2	3	54625000	2997	fs	fs rare
chr07	C	7p11.2	3	54875000	2299	fs	fs rare
chr07	C	7p11.2	3	55125000	8881	fs	fs rare

chr07	C	7p11.2	3	55375000	2033	fs	fs rare
chr07	C	7p11.2	3	55625000	6724	fs	fs rare
chr07	C	7p11.2	3	55875000	5549	fs	fs rare
chr07	C	7p11.2	3	56125000	9848	fs	fs rare
chr07	C	7p11.2	3	56375000	4324	fs	fs rare
chr07	C	7p11.1	.	56625000	8100	nofs	nofs
chr07	C	7p11.1	.	56875000	5434	nofs	nofs
chr07	C	7p11.1	.	57125000	8805	nofs	nofs
chr07	C	7p11.1	.	57375000	14394	nofs	nofs
chr07	C	7p11.1	.	57625000	12706	nofs	nofs
chr07	C	7p11.1	.	57875000	105994	nofs	nofs
chr07	C		.	58125000	0	nofs	nofs
chr07	C		.	58375000	0	nofs	nofs
chr07	C		.	58625000	0	nofs	nofs
chr07	C		.	58875000	0	nofs	nofs
chr07	C		.	59125000	0	nofs	nofs
chr07	C		.	59375000	0	nofs	nofs
chr07	C		.	59625000	0	nofs	nofs
chr07	C		.	59875000	0	nofs	nofs
chr07	C		.	60125000	0	nofs	nofs
chr07	C		.	60375000	0	nofs	nofs
chr07	C		.	60625000	0	nofs	nofs
chr07	C	7q11.1	.	60875000	18948	nofs	nofs
chr07	C	7q11.1	.	61125000	151738	nofs	nofs
chr07	C	7q11.1	.	61375000	136256	nofs	nofs
chr07	C	7q11.1	.	61625000	208842	nofs	nofs
chr07	C	7q11.1	.	61875000	115739	nofs	nofs
chr07	C	7q11.1	.	62125000	4586	nofs	nofs
chr07	C	7q11.21	.	62375000	4650	fs	fs common
chr07	C	7q11.21	.	62625000	9255	fs	fs common
chr07	C	7q11.21	.	62875000	5259	fs	fs common
chr07	C	7q11.21	.	63125000	4012	fs	fs common
chr07	C	7q11.21	.	63375000	11994	fs	fs common
chr07	C	7q11.21	.	63625000	9088	fs	fs common
chr07	C	7q11.21	.	63875000	9203	fs	fs common
chr07	B	7q11.21	.	64125000	10361	fs	fs common

chr07	B	7q11.21	.	64375000	12491	fs	fs common
chr07	B	7q11.21	.	64625000	6379	fs	fs common
chr07	B	7q11.21	.	64875000	4083	fs	fs common
chr07	B	7q11.21	.	65125000	5231	fs	fs common
chr07	B	7q11.21	.	65375000	4385	fs	fs common
chr07	B	7q11.21	.	65625000	3830	fs	fs common
chr07	B	7q11.21	.	65875000	6491	fs	fs common
chr07	B	7q11.21	.	66125000	3130	fs	fs common
chr07	B	7q11.21	.	66375000	6849	fs	fs common
chr07	B	7q11.21	.	66625000	7299	fs	fs common
chr07	E	7q11.22	1	66875000	9815	fs	fs common
chr07	E	7q11.22	1	67125000	7884	fs	fs common
chr07	E	7q11.22	1	67375000	12257	fs	fs common
chr07	E	7q11.22	1	67625000	8682	fs	fs common
chr07	E	7q11.22	1	67875000	6965	fs	fs common
chr07	E	7q11.22	1	68125000	5634	fs	fs common
chr07	E	7q11.22	1	68375000	3901	fs	fs common
chr07	E	7q11.22	1	68625000	1786	fs	fs common
chr07	E	7q11.22	1	68875000	3388	fs	fs common
chr07	E	7q11.22	1	69125000	1615	fs	fs common
chr07	E	7q11.22	1	69375000	3419	fs	fs common
chr07	E	7q11.22	1	69625000	3295	fs	fs common
chr07	E	7q11.22	1	69875000	5934	fs	fs common
chr07	E	7q11.22	1	70125000	4893	fs	fs common
chr07	E	7q11.22	1	70375000	3016	fs	fs common
chr07	E	7q11.22	1	70625000	7537	fs	fs common
chr07	E	7q11.22	1	70875000	4075	fs	fs common
chr07	E	7q11.22	1	71125000	5633	fs	fs common
chr07	E	7q11.22	1	71375000	8117	fs	fs common
chr07	E	7q11.22	1	71625000	3356	fs	fs common
chr07	E	7q11.23	6	71875000	5277	fs	fs common
chr07	E	7q11.23	6	72125000	5293	fs	fs common
chr07	E	7q11.23	6	72375000	8661	fs	fs common
chr07	E	7q11.23	6	72625000	8609	fs	fs common
chr07	E	7q11.23	6	72875000	8133	fs	fs common
chr07	E	7q11.23	6	73125000	7128	fs	fs common

chr07	E	7q11.23	6	73375000	7723	fs	fs common
chr07	E	7q11.23	6	73625000	3029	fs	fs common
chr07	E	7q11.23	6	73875000	5025	fs	fs common
chr07	E	7q11.23	6	74125000	3591	fs	fs common
chr07	E	7q11.23	6	74375000	2015	fs	fs common
chr07	E	7q11.23	6	74625000	5259	fs	fs common
chr07	E	7q11.23	6	74875000	6250	fs	fs common
chr07	E	7q11.23	6	75125000	6346	fs	fs common
chr07	E	7q11.23	6	75375000	4459	fs	fs common
chr07	E	7q11.23	6	75625000	5644	fs	fs common
chr07	E	7q11.23	6	75875000	2288	fs	fs common
chr07	E	7q11.23	6	76125000	2360	fs	fs common
chr07	E	7q11.23	6	76375000	4914	fs	fs common
chr07	E	7q11.23	6	76625000	1755	fs	fs common
chr07	E	7q11.23	6	76875000	6306	fs	fs common
chr07	E	7q11.23	6	77125000	1980	fs	fs common
chr07	E	7q21.1	4	77375000	2426	nofs	nofs
chr07	E	7q21.1	4	77625000	1794	nofs	nofs
chr07	E	7q21.1	4	77875000	2263	nofs	nofs
chr07	E	7q21.1	4	78125000	2212	nofs	nofs
chr07	E	7q21.1	4	78375000	3617	nofs	nofs
chr07	E	7q21.1	4	78625000	3446	nofs	nofs
chr07	E	7q21.1	4	78875000	4175	nofs	nofs
chr07	E	7q21.1	4	79125000	3331	nofs	nofs
chr07	E	7q21.1	4	79375000	3312	nofs	nofs
chr07	E	7q21.1	4	79625000	2582	nofs	nofs
chr07	E	7q21.1	4	79875000	3527	nofs	nofs
chr07	E	7q21.1	4	80125000	2828	nofs	nofs
chr07	E	7q21.1	4	80375000	4035	nofs	nofs
chr07	E	7q21.1	4	80625000	2694	nofs	nofs
chr07	E	7q21.1	4	80875000	1819	nofs	nofs
chr07	E	7q21.1	4	81125000	2215	nofs	nofs
chr07	E	7q21.1	4	81375000	2409	nofs	nofs
chr07	E	7q21.1	4	81625000	2756	nofs	nofs
chr07	E	7q21.1	4	81875000	2686	nofs	nofs
chr07	E	7q21.1	4	82125000	3002	nofs	nofs

chr07	E	7q21.1	4	82375000	3904	nofs	nofs
chr07	E	7q21.1	4	82625000	2989	nofs	nofs
chr07	E	7q21.1	4	82875000	2423	nofs	nofs
chr07	E	7q21.1	4	83125000	1764	nofs	nofs
chr07	E	7q21.1	4	83375000	2002	nofs	nofs
chr07	E	7q21.1	4	83625000	3247	nofs	nofs
chr07	E	7q21.1	4	83875000	1978	nofs	nofs
chr07	E	7q21.1	4	84125000	2772	nofs	nofs
chr07	E	7q21.1	4	84375000	2418	nofs	nofs
chr07	E	7q21.1	4	84625000	3254	nofs	nofs
chr07	E	7q21.1	4	84875000	1806	nofs	nofs
chr07	E	7q21.1	4	85125000	2373	nofs	nofs
chr07	E	7q21.1	4	85375000	2808	nofs	nofs
chr07	E	7q21.1	4	85625000	2274	nofs	nofs
chr07	E	7q21.1	4	85875000	1494	nofs	nofs
chr07	E	7q21.1	4	86125000	1375	nofs	nofs
chr07	E	7q21.1	4	86375000	1506	nofs	nofs
chr07	E	7q21.1	4	86625000	1691	nofs	nofs
chr07	E	7q21.1	4	86875000	3410	nofs	nofs
chr07	E	7q21.1	4	87125000	1289	nofs	nofs
chr07	E	7q21.1	4	87375000	1804	nofs	nofs
chr07	E	7q21.1	4	87625000	3978	nofs	nofs
chr07	E	7q21.1	4	87875000	1883	nofs	nofs
chr07	E	7q21.1	4	88125000	2039	nofs	nofs
chr07	E	7q21.1	4	88375000	2557	nofs	nofs
chr07	E	7q21.1	4	88625000	2754	nofs	nofs
chr07	E	7q21.1	4	88875000	2046	nofs	nofs
chr07	E	7q21.1	4	89125000	3539	nofs	nofs
chr07	E	7q21.1	4	89375000	2461	nofs	nofs
chr07	E	7q21.1	4	89625000	2687	nofs	nofs
chr07	E	7q21.1	4	89875000	2406	nofs	nofs
chr07	E	7q21.1	4	90125000	2402	nofs	nofs
chr07	E	7q21.1	4	90375000	2506	nofs	nofs
chr07	E	7q21.1	4	90625000	2806	nofs	nofs
chr07	E	7q21.1	4	90875000	1606	nofs	nofs
chr07	E	7q21.2	1	91125000	3869	fs	fs common

chr07	E	7q21.2	1	91375000	3343	fs	fs common
chr07	E	7q21.2	1	91625000	1671	fs	fs common
chr07	E	7q21.2	1	91875000	2411	fs	fs common
chr07	E	7q21.2	1	92125000	1594	fs	fs common
chr07	E	7q21.2	1	92375000	2991	fs	fs common
chr07	E	7q21.3	5	92625000	1440	nofs	nofs
chr07	E	7q21.3	5	92875000	2896	nofs	nofs
chr07	E	7q21.3	5	93125000	2563	nofs	nofs
chr07	E	7q21.3	5	93375000	3780	nofs	nofs
chr07	E	7q21.3	5	93625000	2762	nofs	nofs
chr07	E	7q21.3	5	93875000	2663	nofs	nofs
chr07	E	7q21.3	5	94125000	3704	nofs	nofs
chr07	E	7q21.3	5	94375000	3159	nofs	nofs
chr07	E	7q21.3	5	94625000	1667	nofs	nofs
chr07	E	7q21.3	5	94875000	2574	nofs	nofs
chr07	E	7q21.3	5	95125000	1142	nofs	nofs
chr07	E	7q21.3	5	95375000	2353	nofs	nofs
chr07	E	7q21.3	5	95625000	2931	nofs	nofs
chr07	E	7q21.3	5	95875000	1630	nofs	nofs
chr07	E	7q21.3	5	96125000	2111	nofs	nofs
chr07	E	7q21.3	5	96375000	2709	nofs	nofs
chr07	E	7q21.3	5	96625000	2357	nofs	nofs
chr07	E	7q21.3	5	96875000	3699	nofs	nofs
chr07	E	7q21.3	5	97125000	6017	nofs	nofs
chr07	E	7q21.3	5	97375000	5807	nofs	nofs
chr07	E	7q21.3	5	97625000	6041	nofs	nofs
chr07	E	7q22	6	97875000	8730	fs	fs common
chr07	E	7q22	6	98125000	7111	fs	fs common
chr07	E	7q22	6	98375000	4287	fs	fs common
chr07	E	7q22	6	98625000	3294	fs	fs common
chr07	E	7q22	6	98875000	18287	fs	fs common
chr07	E	7q22	6	99125000	11024	fs	fs common
chr07	E	7q22	6	99375000	5725	fs	fs common
chr07	E	7q22	6	99625000	4240	fs	fs common
chr07	E	7q22	6	99875000	8224	fs	fs common
chr07	E	7q22	6	100000000	8121	fs	fs common

chr07	E	7q22	6	100000000	17877	fs	fs common
chr07	E	7q22	6	101000000	9094	fs	fs common
chr07	E	7q22	6	101000000	6933	fs	fs common
chr07	E	7q22	6	101000000	5561	fs	fs common
chr07	E	7q22	6	101000000	4403	fs	fs common
chr07	E	7q22	6	102000000	6485	fs	fs common
chr07	E	7q22	6	102000000	4766	fs	fs common
chr07	E	7q22	6	102000000	2199	fs	fs common
chr07	E	7q22	6	102000000	3373	fs	fs common
chr07	E	7q22	6	103000000	2138	fs	fs common
chr07	E	7q22	6	103000000	1230	fs	fs common
chr07	E	7q22	6	103000000	2990	fs	fs common
chr07	E	7q22	6	103000000	1977	fs	fs common
chr07	E	7q22	6	104000000	1863	fs	fs common
chr07	E	7q22	6	104000000	2089	fs	fs common
chr07	E	7q22	6	104000000	5258	fs	fs common
chr07	E	7q22	6	104000000	3027	fs	fs common
chr07	E	7q22	6	105000000	9235	fs	fs common
chr07	E	7q22	6	105000000	4146	fs	fs common
chr07	E	7q22	6	105000000	2273	fs	fs common
chr07	E	7q22	6	105000000	2041	fs	fs common
chr07	E	7q22	6	106000000	1421	fs	fs common
chr07	E	7q22	6	106000000	4963	fs	fs common
chr07	E	7q22	6	106000000	2656	fs	fs common
chr07	E	7q22	6	106000000	2485	fs	fs common
chr07	E	7q22	6	107000000	2517	fs	fs common
chr07	E	7q22	6	107000000	3003	fs	fs common
chr07	E	7q31.1	4	107000000	4268	nofs	nofs
chr07	E	7q31.1	4	107000000	2576	nofs	nofs
chr07	E	7q31.1	4	108000000	1578	nofs	nofs
chr07	E	7q31.1	4	108000000	2638	nofs	nofs
chr07	E	7q31.1	4	108000000	2310	nofs	nofs
chr07	E	7q31.1	4	108000000	2842	nofs	nofs
chr07	E	7q31.1	4	109000000	4205	nofs	nofs
chr07	E	7q31.1	4	109000000	3411	nofs	nofs
chr07	E	7q31.1	4	109000000	2675	nofs	nofs

chr07	E	7q31.1	4	109000000	2167	nofs	nofs
chr07	E	7q31.1	4	110000000	3510	nofs	nofs
chr07	E	7q31.1	4	110000000	1386	nofs	nofs
chr07	E	7q31.1	4	110000000	1354	nofs	nofs
chr07	E	7q31.1	4	110000000	2972	nofs	nofs
chr07	E	7q31.1	4	111000000	2689	nofs	nofs
chr07	E	7q31.1	4	111000000	2636	nofs	nofs
chr07	E	7q31.1	4	111000000	2045	nofs	nofs
chr07	E	7q31.1	4	111000000	1721	nofs	nofs
chr07	E	7q31.1	4	112000000	2243	nofs	nofs
chr07	E	7q31.1	4	112000000	2944	nofs	nofs
chr07	E	7q31.1	4	112000000	4452	nofs	nofs
chr07	E	7q31.1	4	112000000	3536	nofs	nofs
chr07	E	7q31.1	4	113000000	1591	nofs	nofs
chr07	E	7q31.1	4	113000000	1649	nofs	nofs
chr07	E	7q31.1	4	113000000	2355	nofs	nofs
chr07	E	7q31.1	4	113000000	3127	nofs	nofs
chr07	E	7q31.1	4	114000000	3047	nofs	nofs
chr07	E	7q31.1	4	114000000	2070	nofs	nofs
chr07	E	7q31.1	4	114000000	1983	nofs	nofs
chr07	B	7q31.2	.	114000000	2027	fs	fs common
chr07	B	7q31.2	.	115000000	2709	fs	fs common
chr07	B	7q31.2	.	115000000	3071	fs	fs common
chr07	B	7q31.2	.	115000000	1780	fs	fs common
chr07	B	7q31.2	.	115000000	2277	fs	fs common
chr07	B	7q31.2	.	116000000	2369	fs	fs common
chr07	B	7q31.2	.	116000000	3381	fs	fs common
chr07	B	7q31.2	.	116000000	3371	fs	fs common
chr07	B	7q31.2	.	116000000	2537	fs	fs common
chr07	B	7q31.2	.	117000000	1491	fs	fs common
chr07	B	7q31.2	.	117000000	2243	fs	fs common
chr07	B	7q31.2	.	117000000	1744	fs	fs common
chr07	E	7q31.3	1	117000000	2155	nofs	nofs
chr07	E	7q31.3	1	118000000	2347	nofs	nofs
chr07	E	7q31.3	1	118000000	2004	nofs	nofs
chr07	E	7q31.3	1	118000000	3228	nofs	nofs

chr07	E	7q31.3	1	118000000	2177	nofs	nofs
chr07	E	7q31.3	1	119000000	2927	nofs	nofs
chr07	E	7q31.3	1	119000000	1772	nofs	nofs
chr07	E	7q31.3	1	119000000	2505	nofs	nofs
chr07	E	7q31.3	1	119000000	2129	nofs	nofs
chr07	E	7q31.3	1	120000000	2199	nofs	nofs
chr07	E	7q31.3	1	120000000	2417	nofs	nofs
chr07	E	7q31.3	1	120000000	2123	nofs	nofs
chr07	E	7q31.3	1	120000000	1695	nofs	nofs
chr07	E	7q31.3	1	121000000	4258	nofs	nofs
chr07	E	7q31.3	1	121000000	3721	nofs	nofs
chr07	E	7q31.3	1	121000000	2785	nofs	nofs
chr07	E	7q31.3	1	121000000	2680	nofs	nofs
chr07	E	7q31.3	1	122000000	3934	nofs	nofs
chr07	E	7q31.3	1	122000000	3858	nofs	nofs
chr07	E	7q31.3	1	122000000	2356	nofs	nofs
chr07	E	7q31.3	1	122000000	4308	nofs	nofs
chr07	E	7q31.3	1	123000000	1443	nofs	nofs
chr07	E	7q31.3	1	123000000	3685	nofs	nofs
chr07	E	7q31.3	1	123000000	1935	nofs	nofs
chr07	E	7q31.3	1	123000000	2133	nofs	nofs
chr07	E	7q31.3	1	124000000	1960	nofs	nofs
chr07	E	7q31.3	1	124000000	3055	nofs	nofs
chr07	E	7q31.3	1	124000000	1959	nofs	nofs
chr07	E	7q31.3	1	124000000	2260	nofs	nofs
chr07	E	7q31.3	1	125000000	3595	nofs	nofs
chr07	E	7q31.3	1	125000000	3358	nofs	nofs
chr07	E	7q31.3	1	125000000	2688	nofs	nofs
chr07	E	7q31.3	1	125000000	3001	nofs	nofs
chr07	E	7q31.3	1	126000000	2995	nofs	nofs
chr07	E	7q31.3	1	126000000	1812	nofs	nofs
chr07	E	7q32	1	126000000	2080	nofs	nofs
chr07	E	7q32	1	126000000	2653	nofs	nofs
chr07	E	7q32	1	127000000	2019	nofs	nofs
chr07	E	7q32	1	127000000	3126	nofs	nofs
chr07	E	7q32	1	127000000	1681	nofs	nofs

chr07	E	7q32	1	127000000	3458	nofs	nofs
chr07	E	7q32	1	128000000	3906	nofs	nofs
chr07	E	7q32	1	128000000	4667	nofs	nofs
chr07	E	7q32	1	128000000	2588	nofs	nofs
chr07	E	7q32	1	128000000	3275	nofs	nofs
chr07	E	7q32	1	129000000	3166	nofs	nofs
chr07	E	7q32	1	129000000	2452	nofs	nofs
chr07	E	7q32	1	129000000	4654	nofs	nofs
chr07	E	7q32	1	129000000	3800	nofs	nofs
chr07	E	7q32	1	130000000	2620	nofs	nofs
chr07	E	7q32	1	130000000	2118	fs	fs common
chr07	E	7q32	1	130000000	3287	fs	fs common
chr07	E	7q32	1	130000000	3381	fs	fs common
chr07	E	7q32	1	131000000	3133	fs	fs common
chr07	E	7q32	1	131000000	5004	fs	fs common
chr07	E	7q32	1	131000000	5446	fs	fs common
chr07	E	7q32	1	131000000	3955	fs	fs common
chr07	E	7q32	1	132000000	5534	fs	fs common
chr07	E	7q32	1	132000000	3706	fs	fs common
chr07	E	7q33	2	132000000	3290	nofs	nofs
chr07	E	7q33	2	132000000	2448	nofs	nofs
chr07	E	7q33	2	133000000	1345	nofs	nofs
chr07	E	7q33	2	133000000	2554	nofs	nofs
chr07	E	7q33	2	133000000	2263	nofs	nofs
chr07	E	7q33	2	133000000	2649	nofs	nofs
chr07	E	7q33	2	134000000	2748	nofs	nofs
chr07	E	7q33	2	134000000	2455	nofs	nofs
chr07	E	7q33	2	134000000	2145	nofs	nofs
chr07	E	7q33	2	134000000	2561	nofs	nofs
chr07	E	7q33	2	135000000	3939	nofs	nofs
chr07	E	7q33	2	135000000	3962	nofs	nofs
chr07	E	7q33	2	135000000	3445	nofs	nofs
chr07	E	7q33	2	135000000	3232	nofs	nofs
chr07	E	7q33	2	136000000	2966	nofs	nofs
chr07	E	7q33	2	136000000	2613	nofs	nofs
chr07	E	7q33	2	136000000	4111	nofs	nofs

chr07	E	7q33	2	136000000	3094	nofs	nofs
chr07	E	7q33	2	137000000	2717	nofs	nofs
chr07	E	7q33	2	137000000	2290	nofs	nofs
chr07	E	7q34	3	137000000	3187	nofs	nofs
chr07	E	7q34	3	137000000	4983	nofs	nofs
chr07	E	7q34	3	138000000	3694	nofs	nofs
chr07	E	7q34	3	138000000	5090	nofs	nofs
chr07	E	7q34	3	138000000	3402	nofs	nofs
chr07	E	7q34	3	138000000	3735	nofs	nofs
chr07	E	7q34	3	139000000	5549	nofs	nofs
chr07	E	7q34	3	139000000	1991	nofs	nofs
chr07	E	7q34	3	139000000	4187	nofs	nofs
chr07	E	7q34	3	139000000	4691	nofs	nofs
chr07	E	7q34	3	140000000	5191	nofs	nofs
chr07	E	7q34	3	140000000	2601	nofs	nofs
chr07	E	7q34	3	140000000	3065	nofs	nofs
chr07	E	7q34	3	140000000	2478	nofs	nofs
chr07	E	7q34	3	141000000	2546	nofs	nofs
chr07	E	7q34	3	141000000	2220	nofs	nofs
chr07	E	7q34	3	141000000	6825	nofs	nofs
chr07	E	7q34	3	141000000	3608	nofs	nofs
chr07	E	7q34	3	142000000	2316	nofs	nofs
chr07	E	7q34	3	142000000	1685	nofs	nofs
chr07	E	7q34	3	142000000	4380	nofs	nofs
chr07	E	7q34	3	142000000	3104	nofs	nofs
chr07	E	7q34	3	143000000	3210	nofs	nofs
chr07	E	7q34	3	143000000	1944	nofs	nofs
chr07	E	7q35	2	143000000	3158	nofs	nofs
chr07	E	7q35	2	143000000	2757	nofs	nofs
chr07	E	7q35	2	144000000	2263	nofs	nofs
chr07	E	7q35	2	144000000	3916	nofs	nofs
chr07	E	7q35	2	144000000	3682	nofs	nofs
chr07	E	7q35	2	144000000	4290	nofs	nofs
chr07	E	7q35	2	145000000	1338	nofs	nofs
chr07	E	7q35	2	145000000	3135	nofs	nofs
chr07	E	7q35	2	145000000	2900	nofs	nofs

chr07	E	7q35	2	145000000	3541	nofs	nofs
chr07	E	7q35	2	146000000	3404	nofs	nofs
chr07	E	7q35	2	146000000	5236	nofs	nofs
chr07	E	7q35	2	146000000	3980	nofs	nofs
chr07	E	7q35	2	146000000	3564	nofs	nofs
chr07	E	7q35	2	147000000	2866	nofs	nofs
chr07	E	7q35	2	147000000	1910	nofs	nofs
chr07	E	7q35	2	147000000	2331	nofs	nofs
chr07	E	7q35	2	147000000	4779	nofs	nofs
chr07	E	7q36	5	148000000	4633	fs	fs common
chr07	E	7q36	5	148000000	3376	fs	fs common
chr07	E	7q36	5	148000000	4949	fs	fs common
chr07	E	7q36	5	148000000	3757	fs	fs common
chr07	E	7q36	5	149000000	4139	fs	fs common
chr07	E	7q36	5	149000000	4511	fs	fs common
chr07	E	7q36	5	149000000	9998	fs	fs common
chr07	E	7q36	5	149000000	4706	fs	fs common
chr07	E	7q36	5	150000000	3068	fs	fs common
chr07	E	7q36	5	150000000	2840	fs	fs common
chr07	E	7q36	5	150000000	5582	fs	fs common
chr07	E	7q36	5	150000000	4127	fs	fs common
chr07	E	7q36	5	151000000	4404	fs	fs common
chr07	E	7q36	5	151000000	7308	fs	fs common
chr07	E	7q36	5	151000000	5092	fs	fs common
chr07	E	7q36	5	151000000	3543	fs	fs common
chr07	E	7q36	5	152000000	7146	fs	fs common
chr07	E	7q36	5	152000000	5035	fs	fs common
chr07	E	7q36	5	152000000	7199	fs	fs common
chr07	E	7q36	5	152000000	3836	fs	fs common
chr07	E	7q36	5	153000000	3439	fs	fs common
chr07	E	7q36	5	153000000	4187	fs	fs common
chr07	E	7q36	5	153000000	7703	fs	fs common
chr07	E	7q36	5	153000000	6787	fs	fs common
chr07	E	7q36	5	154000000	1860	fs	fs common
chr07	E	7q36	5	154000000	10581	fs	fs common
chr07	E	7q36	5	154000000	7076	fs	fs common

chr07	E	7q36	5	154000000	9471	fs	fs common
chr07	E	7q36	5	155000000	13700	fs	fs common
chr07	E	7q36	5	155000000	7928	fs	fs common
chr07	E	7q36	5	155000000	10793	fs	fs common
chr07	T	7q36	5	155000000	12313	fs	fs common
chr07	T	7q36	5	156000000	9073	fs	fs common
chr07	T	7q36	5	156000000	6924	fs	fs common
chr07	T	7q36	5	156000000	3883	fs	fs common
chr07	T	7q36	5	156000000	5788	fs	fs common
chr07	T	7q36	5	157000000	10219	fs	fs common
chr07	T	7q36	5	157000000	27977	fs	fs common
chr07	T	7q36	5	157000000	49788	fs	fs common
chr07	T	7q36	5	157000000	38602	fs	fs common
chr07	T	7q36	5	158000000	49864	fs	fs common
chr07	T	7q36	5	158000000	21711	fs	fs common
chr07	T	7q36	5	158000000	19432	fs	fs common
chr07	T	7q36	5	158000000	29421	fs	fs common
chr08	T	8p23.3	1	125000	19177	nofs	nofs
chr08	T	8p23.3	1	375000	10501	nofs	nofs
chr08	T	8p23.3	1	625000	25187	nofs	nofs
chr08	T	8p23.3	1	875000	32777	nofs	nofs
chr08	T	8p23.3	1	1125000	35217	nofs	nofs
chr08	T	8p23.3	1	1375000	49944	nofs	nofs
chr08	T	8p23.3	1	1625000	23510	nofs	nofs
chr08	T	8p23.3	1	1875000	25550	nofs	nofs
chr08	T	8p23.3	1	2125000	14446	nofs	nofs
chr08	T	8p23.3	1	2375000	7763	nofs	nofs
chr08	T	8p23.2	.	2625000	6046	nofs	nofs
chr08	T	8p23.2	.	2875000	7006	nofs	nofs
chr08	T	8p23.2	.	3125000	7187	nofs	nofs
chr08	B	8p23.2	.	3375000	7365	nofs	nofs
chr08	B	8p23.2	.	3625000	7929	nofs	nofs
chr08	B	8p23.2	.	3875000	3782	nofs	nofs
chr08	B	8p23.2	.	4125000	4064	nofs	nofs
chr08	B	8p23.2	.	4375000	4351	nofs	nofs
chr08	B	8p23.2	.	4625000	3263	nofs	nofs

chr08	B	8p23.2	.	4875000	3988	nofs	nofs
chr08	B	8p23.2	.	5125000	3695	nofs	nofs
chr08	B	8p23.2	.	5375000	2207	nofs	nofs
chr08	B	8p23.2	.	5625000	2485	nofs	nofs
chr08	B	8p23.2	.	5875000	6514	nofs	nofs
chr08	E	8p23.1	5	6125000	6123	nofs	nofs
chr08	E	8p23.1	5	6375000	5828	nofs	nofs
chr08	E	8p23.1	5	6625000	4598	nofs	nofs
chr08	E	8p23.1	5	6875000	4465	nofs	nofs
chr08	E	8p23.1	5	7125000	2143	nofs	nofs
chr08	E	8p23.1	5	7375000	2242	nofs	nofs
chr08	E	8p23.1	5	7625000	2130	nofs	nofs
chr08	E	8p23.1	5	7875000	2723	nofs	nofs
chr08	E	8p23.1	5	8125000	2921	nofs	nofs
chr08	E	8p23.1	5	8375000	2912	nofs	nofs
chr08	E	8p23.1	5	8625000	4671	nofs	nofs
chr08	E	8p23.1	5	8875000	2312	nofs	nofs
chr08	E	8p23.1	5	9125000	3408	nofs	nofs
chr08	E	8p23.1	5	9375000	2692	nofs	nofs
chr08	E	8p23.1	5	9625000	5425	nofs	nofs
chr08	E	8p23.1	5	9875000	3063	nofs	nofs
chr08	E	8p23.1	5	10125000	1644	nofs	nofs
chr08	E	8p23.1	5	10375000	2915	nofs	nofs
chr08	E	8p23.1	5	10625000	7866	nofs	nofs
chr08	E	8p23.1	5	10875000	3839	nofs	nofs
chr08	E	8p23.1	5	11125000	3486	nofs	nofs
chr08	E	8p23.1	5	11375000	4917	nofs	nofs
chr08	E	8p23.1	5	11625000	3160	nofs	nofs
chr08	E	8p23.1	5	11875000	6066	nofs	nofs
chr08	E	8p23.1	5	12125000	1791	nofs	nofs
chr08	E	8p23.1	5	12375000	4829	nofs	nofs
chr08	E	8p23.1	5	12625000	4807	nofs	nofs
chr08	E	8p22	5	12875000	4446	nofs	nofs
chr08	E	8p22	5	13125000	3637	nofs	nofs
chr08	E	8p22	5	13375000	2896	nofs	nofs
chr08	E	8p22	5	13625000	2472	nofs	nofs

chr08	E	8p22	5	13875000	2291	nofs	nofs
chr08	E	8p22	5	14125000	2494	nofs	nofs
chr08	E	8p22	5	14375000	2205	nofs	nofs
chr08	E	8p22	5	14625000	2692	nofs	nofs
chr08	E	8p22	5	14875000	4683	nofs	nofs
chr08	E	8p22	5	15125000	1457	nofs	nofs
chr08	E	8p22	5	15375000	3841	nofs	nofs
chr08	E	8p22	5	15625000	2056	nofs	nofs
chr08	E	8p22	5	15875000	3519	nofs	nofs
chr08	E	8p22	5	16125000	4026	nofs	nofs
chr08	E	8p22	5	16375000	2873	nofs	nofs
chr08	E	8p22	5	16625000	4425	nofs	nofs
chr08	E	8p22	5	16875000	1890	nofs	nofs
chr08	E	8p22	5	17125000	1681	nofs	nofs
chr08	E	8p22	5	17375000	2582	nofs	nofs
chr08	E	8p22	5	17625000	2810	nofs	nofs
chr08	E	8p22	5	17875000	4319	nofs	nofs
chr08	E	8p22	5	18125000	2102	nofs	nofs
chr08	E	8p22	5	18375000	2202	nofs	nofs
chr08	E	8p22	5	18625000	2071	nofs	nofs
chr08	E	8p22	5	18875000	2089	nofs	nofs
chr08	E	8p21.3	5	19125000	4601	nofs	nofs
chr08	E	8p21.3	5	19375000	3893	nofs	nofs
chr08	E	8p21.3	5	19625000	2442	nofs	nofs
chr08	E	8p21.3	5	19875000	2752	nofs	nofs
chr08	E	8p21.3	5	20125000	3337	nofs	nofs
chr08	E	8p21.3	5	20375000	4591	nofs	nofs
chr08	E	8p21.3	5	20625000	2013	nofs	nofs
chr08	E	8p21.3	5	20875000	4077	nofs	nofs
chr08	E	8p21.3	5	21125000	6959	nofs	nofs
chr08	E	8p21.3	5	21375000	2661	nofs	nofs
chr08	E	8p21.3	5	21625000	4007	nofs	nofs
chr08	E	8p21.3	5	21875000	3357	nofs	nofs
chr08	E	8p21.3	5	22125000	2791	nofs	nofs
chr08	E	8p21.3	5	22375000	3634	nofs	nofs
chr08	E	8p21.3	5	22625000	4556	nofs	nofs

chr08	E	8p21.3	5	22875000	2491	nofs	nofs
chr08	E	8p21.3	5	23125000	3948	nofs	nofs
chr08	E	8p21.3	5	23375000	3193	nofs	nofs
chr08	E	8p21.2	2	23625000	2689	nofs	nofs
chr08	E	8p21.2	2	23875000	3470	nofs	nofs
chr08	E	8p21.2	2	24125000	1595	nofs	nofs
chr08	E	8p21.2	2	24375000	2431	nofs	nofs
chr08	E	8p21.2	2	24625000	2166	nofs	nofs
chr08	E	8p21.2	2	24875000	3196	nofs	nofs
chr08	E	8p21.2	2	25125000	6408	nofs	nofs
chr08	E	8p21.2	2	25375000	3278	nofs	nofs
chr08	E	8p21.2	2	25625000	2707	nofs	nofs
chr08	E	8p21.2	2	25875000	2385	nofs	nofs
chr08	E	8p21.2	2	26125000	4648	nofs	nofs
chr08	E	8p21.2	2	26375000	3748	nofs	nofs
chr08	E	8p21.2	2	26625000	2985	nofs	nofs
chr08	E	8p21.2	2	26875000	1613	nofs	nofs
chr08	E	8p21.2	2	27125000	3524	nofs	nofs
chr08	E	8p21.2	2	27375000	3456	nofs	nofs
chr08	E	8p21.1	3	27625000	3085	nofs	nofs
chr08	E	8p21.1	3	27875000	4299	nofs	nofs
chr08	E	8p21.1	3	28125000	2335	nofs	nofs
chr08	E	8p21.1	3	28375000	2693	nofs	nofs
chr08	E	8p21.1	3	28625000	2007	nofs	nofs
chr08	E	8p21.1	3	28875000	2453	nofs	nofs
chr08	E	8p12	5	29125000	15660	nofs	nofs
chr08	E	8p12	5	29375000	3050	nofs	nofs
chr08	E	8p12	5	29625000	4108	nofs	nofs
chr08	E	8p12	5	29875000	1788	nofs	nofs
chr08	E	8p12	5	30125000	5882	nofs	nofs
chr08	E	8p12	5	30375000	3478	nofs	nofs
chr08	E	8p12	5	30625000	4594	nofs	nofs
chr08	E	8p12	5	30875000	3953	nofs	nofs
chr08	E	8p12	5	31125000	1900	nofs	nofs
chr08	E	8p12	5	31375000	2243	nofs	nofs
chr08	E	8p12	5	31625000	1615	nofs	nofs

chr08	E	8p12	5	31875000	1824	nofs	nofs
chr08	E	8p12	5	32125000	2491	nofs	nofs
chr08	E	8p12	5	32375000	2993	nofs	nofs
chr08	E	8p12	5	32625000	2155	nofs	nofs
chr08	E	8p12	5	32875000	1148	nofs	nofs
chr08	E	8p12	5	33125000	1689	nofs	nofs
chr08	E	8p12	5	33375000	5315	nofs	nofs
chr08	E	8p12	5	33625000	5049	nofs	nofs
chr08	E	8p12	5	33875000	2286	nofs	nofs
chr08	E	8p12	5	34125000	2260	nofs	nofs
chr08	E	8p12	5	34375000	2577	nofs	nofs
chr08	E	8p12	5	34625000	4096	nofs	nofs
chr08	E	8p12	5	34875000	3330	nofs	nofs
chr08	E	8p12	5	35125000	5062	nofs	nofs
chr08	E	8p12	5	35375000	2143	nofs	nofs
chr08	E	8p12	5	35625000	1826	nofs	nofs
chr08	E	8p12	5	35875000	2564	nofs	nofs
chr08	E	8p12	5	36125000	2850	nofs	nofs
chr08	E	8p12	5	36375000	3185	nofs	nofs
chr08	E	8p12	5	36625000	2700	nofs	nofs
chr08	E	8p12	5	36875000	2835	nofs	nofs
chr08	E	8p12	5	37125000	2463	nofs	nofs
chr08	E	8p12	5	37375000	5597	nofs	nofs
chr08	E	8p12	5	37625000	4302	nofs	nofs
chr08	E	8p12	5	37875000	5975	nofs	nofs
chr08	E	8p12	5	38125000	5116	nofs	nofs
chr08	E	8p12	5	38375000	3815	nofs	nofs
chr08	E	8p11.2	1	38625000	5009	nofs	nofs
chr08	E	8p11.2	1	38875000	3685	nofs	nofs
chr08	E	8p11.2	1	39125000	3008	nofs	nofs
chr08	E	8p11.2	1	39375000	4310	nofs	nofs
chr08	E	8p11.2	1	39625000	2561	nofs	nofs
chr08	E	8p11.2	1	39875000	3132	nofs	nofs
chr08	E	8p11.2	1	40125000	3319	nofs	nofs
chr08	E	8p11.2	1	40375000	2665	nofs	nofs
chr08	E	8p11.2	1	40625000	2451	nofs	nofs

chr08	E	8p11.2	1	40875000	4802	nofs	nofs
chr08	C	8p11.2	1	41125000	3438	nofs	nofs
chr08	C	8p11.2	1	41375000	3337	nofs	nofs
chr08	C	8p11.2	1	41625000	4649	nofs	nofs
chr08	C	8p11.2	1	41875000	3091	nofs	nofs
chr08	C	8p11.2	1	42125000	4694	nofs	nofs
chr08	C	8p11.2	1	42375000	3967	nofs	nofs
chr08	C	8p11.2	1	42625000	4651	nofs	nofs
chr08	C	8p11.1	.	42875000	4484	nofs	nofs
chr08	C	8p11.1	.	43125000	9288	nofs	nofs
chr08	C	8p11.1	.	43125000	9288	nofs	nofs
chr08	C	8p11.1	.	43375000	2184	nofs	nofs
chr08	C	8p11.1	.	43625000	160901	nofs	nofs
chr08	C	8p11.1	.	43875000	170122	nofs	nofs
chr08	C		.	44125000	0	nofs	nofs
chr08	C		.	44375000	0	nofs	nofs
chr08	C		.	44625000	0	nofs	nofs
chr08	C		.	44875000	0	nofs	nofs
chr08	C		.	45125000	0	nofs	nofs
chr08	C		.	45375000	0	nofs	nofs
chr08	C		.	45625000	0	nofs	nofs
chr08	C		.	45875000	0	nofs	nofs
chr08	C		.	46125000	0	nofs	nofs
chr08	C		.	46375000	0	nofs	nofs
chr08	C		.	46625000	0	nofs	nofs
chr08	C	8q11.1	.	46875000	39220	nofs	nofs
chr08	C	8q11.1	.	47125000	96603	nofs	nofs
chr08	C	8q11.1	.	47375000	98378	nofs	nofs
chr08	C	8q11.1	.	47625000	58806	nofs	nofs
chr08	C	8q11.1	.	47875000	3018	nofs	nofs
chr08	C	8q11.1	.	48125000	3864	nofs	nofs
chr08	C	8q11.21	3	48375000	1962	nofs	nofs
chr08	C	8q11.21	3	48625000	1903	nofs	nofs
chr08	C	8q11.21	3	48875000	3782	nofs	nofs
chr08	C	8q11.21	3	49125000	4774	nofs	nofs
chr08	C	8q11.21	3	49375000	3258	nofs	nofs

chr08	C	8q11.21	3	49625000	1304	nofs	nofs
chr08	C	8q11.21	3	49875000	3666	nofs	nofs
chr08	E	8q11.21	3	50125000	3668	nofs	nofs
chr08	E	8q11.21	3	50375000	3629	nofs	nofs
chr08	E	8q11.21	3	50625000	4021	nofs	nofs
chr08	E	8q11.21	3	50875000	3082	nofs	nofs
chr08	E	8q11.21	3	51125000	1972	nofs	nofs
chr08	E	8q11.21	3	51375000	2156	nofs	nofs
chr08	E	8q11.21	3	51625000	3129	nofs	nofs
chr08	E	8q11.21	3	51875000	2098	nofs	nofs
chr08	E	8q11.21	3	52125000	3433	nofs	nofs
chr08	E	8q11.21	3	52375000	4438	nofs	nofs
chr08	B	8q11.22	.	52625000	5375	nofs	nofs
chr08	E	8q11.23	1	52875000	4173	nofs	nofs
chr08	E	8q11.23	1	53125000	1951	nofs	nofs
chr08	E	8q11.23	1	53375000	1677	nofs	nofs
chr08	E	8q11.23	1	53625000	4115	nofs	nofs
chr08	E	8q11.23	1	53875000	2655	nofs	nofs
chr08	E	8q11.23	1	54125000	3608	nofs	nofs
chr08	E	8q11.23	1	54375000	2435	nofs	nofs
chr08	E	8q11.23	1	54625000	4058	nofs	nofs
chr08	E	8q11.23	1	54875000	1887	nofs	nofs
chr08	E	8q11.23	1	55125000	6563	nofs	nofs
chr08	E	8q11.23	1	55375000	2244	nofs	nofs
chr08	E	8q11.23	1	55625000	3882	nofs	nofs
chr08	E	8q12	3	55875000	3823	nofs	nofs
chr08	E	8q12	3	56125000	1870	nofs	nofs
chr08	E	8q12	3	56375000	1779	nofs	nofs
chr08	E	8q12	3	56625000	3047	nofs	nofs
chr08	E	8q12	3	56875000	4395	nofs	nofs
chr08	E	8q12	3	57125000	4226	nofs	nofs
chr08	E	8q12	3	57375000	2977	nofs	nofs
chr08	E	8q12	3	57625000	2394	nofs	nofs
chr08	E	8q12	3	57875000	3021	nofs	nofs
chr08	E	8q12	3	58125000	3138	nofs	nofs
chr08	E	8q12	3	58375000	5901	nofs	nofs

chr08	E	8q12	3	58625000	4312	nofs	nofs
chr08	E	8q12	3	58875000	1687	nofs	nofs
chr08	E	8q12	3	59125000	2208	nofs	nofs
chr08	E	8q12	3	59375000	1904	nofs	nofs
chr08	E	8q12	3	59625000	2602	nofs	nofs
chr08	E	8q12	3	59875000	2631	nofs	nofs
chr08	E	8q12	3	60125000	2573	nofs	nofs
chr08	E	8q12	3	60375000	1731	nofs	nofs
chr08	E	8q12	3	60625000	3245	nofs	nofs
chr08	E	8q12	3	60875000	2130	nofs	nofs
chr08	E	8q12	3	61125000	2009	nofs	nofs
chr08	E	8q12	3	61375000	1946	nofs	nofs
chr08	E	8q12	3	61625000	4896	nofs	nofs
chr08	E	8q12	3	61875000	1873	nofs	nofs
chr08	E	8q12	3	62125000	2405	nofs	nofs
chr08	E	8q12	3	62375000	2012	nofs	nofs
chr08	E	8q12	3	62625000	1692	nofs	nofs
chr08	E	8q12	3	62875000	3141	nofs	nofs
chr08	E	8q12	3	63125000	1708	nofs	nofs
chr08	E	8q12	3	63375000	4649	nofs	nofs
chr08	E	8q12	3	63625000	2432	nofs	nofs
chr08	E	8q12	3	63875000	2134	nofs	nofs
chr08	E	8q12	3	64125000	2491	nofs	nofs
chr08	E	8q12	3	64375000	2730	nofs	nofs
chr08	E	8q12	3	64625000	2127	nofs	nofs
chr08	E	8q12	3	64875000	2185	nofs	nofs
chr08	E	8q12	3	65125000	3607	nofs	nofs
chr08	E	8q12	3	65375000	2844	nofs	nofs
chr08	E	8q12	3	65625000	2569	nofs	nofs
chr08	E	8q12	3	65875000	3672	nofs	nofs
chr08	E	8q13	2	66125000	1985	nofs	nofs
chr08	E	8q13	2	66375000	1979	nofs	nofs
chr08	E	8q13	2	66625000	3967	nofs	nofs
chr08	E	8q13	2	66875000	2375	nofs	nofs
chr08	E	8q13	2	67125000	2207	nofs	nofs
chr08	E	8q13	2	67375000	4026	nofs	nofs

chr08	E	8q13	2	67625000	3897	nofs	nofs
chr08	E	8q13	2	67875000	3439	nofs	nofs
chr08	E	8q13	2	68125000	2967	nofs	nofs
chr08	E	8q13	2	68375000	3607	nofs	nofs
chr08	E	8q13	2	68625000	2737	nofs	nofs
chr08	E	8q13	2	68875000	1915	nofs	nofs
chr08	E	8q13	2	69125000	1886	nofs	nofs
chr08	E	8q13	2	69375000	2736	nofs	nofs
chr08	E	8q13	2	69625000	1720	nofs	nofs
chr08	E	8q13	2	69875000	3183	nofs	nofs
chr08	E	8q13	2	70125000	2210	nofs	nofs
chr08	E	8q13	2	70375000	6541	nofs	nofs
chr08	E	8q13	2	70625000	3182	nofs	nofs
chr08	E	8q13	2	70875000	3540	nofs	nofs
chr08	E	8q13	2	71125000	2551	nofs	nofs
chr08	E	8q13	2	71375000	1611	nofs	nofs
chr08	E	8q13	2	71625000	2815	nofs	nofs
chr08	E	8q13	2	71875000	2392	nofs	nofs
chr08	E	8q13	2	72125000	1737	nofs	nofs
chr08	E	8q13	2	72375000	1581	nofs	nofs
chr08	E	8q13	2	72625000	1514	nofs	nofs
chr08	E	8q13	2	72875000	4473	nofs	nofs
chr08	E	8q13	2	73125000	2007	nofs	nofs
chr08	E	8q13	2	73375000	2992	nofs	nofs
chr08	E	8q13	2	73625000	2043	nofs	nofs
chr08	E	8q13	2	73875000	1219	nofs	nofs
chr08	E	8q13	2	74125000	4958	nofs	nofs
chr08	E	8q21.1	3	74375000	1772	nofs	nofs
chr08	E	8q21.1	3	74625000	2308	nofs	nofs
chr08	E	8q21.1	3	74875000	4773	nofs	nofs
chr08	E	8q21.1	3	75125000	2695	nofs	nofs
chr08	E	8q21.1	3	75375000	2136	nofs	nofs
chr08	E	8q21.1	3	75625000	2149	nofs	nofs
chr08	E	8q21.1	3	75875000	2286	nofs	nofs
chr08	E	8q21.1	3	76125000	2419	nofs	nofs
chr08	E	8q21.1	3	76375000	3382	nofs	nofs

chr08	E	8q21.1	3	76625000	2837	nofs	nofs
chr08	E	8q21.1	3	76875000	1861	nofs	nofs
chr08	E	8q21.1	3	77125000	3234	nofs	nofs
chr08	E	8q21.1	3	77375000	2625	nofs	nofs
chr08	E	8q21.1	3	77625000	3226	nofs	nofs
chr08	E	8q21.1	3	77875000	2340	nofs	nofs
chr08	E	8q21.1	3	78125000	1232	nofs	nofs
chr08	E	8q21.1	3	78375000	3799	nofs	nofs
chr08	E	8q21.1	3	78625000	2784	nofs	nofs
chr08	E	8q21.1	3	78875000	2453	nofs	nofs
chr08	E	8q21.1	3	79125000	2086	nofs	nofs
chr08	E	8q21.1	3	79375000	2904	nofs	nofs
chr08	E	8q21.1	3	79625000	2261	nofs	nofs
chr08	E	8q21.1	3	79875000	3057	nofs	nofs
chr08	E	8q21.1	3	80125000	1767	nofs	nofs
chr08	E	8q21.1	3	80375000	2661	nofs	nofs
chr08	E	8q21.1	3	80625000	1467	nofs	nofs
chr08	E	8q21.1	3	80875000	2920	nofs	nofs
chr08	E	8q21.1	3	81125000	2496	nofs	nofs
chr08	E	8q21.1	3	81375000	4980	nofs	nofs
chr08	E	8q21.1	3	81625000	1797	nofs	nofs
chr08	E	8q21.1	3	81875000	1573	nofs	nofs
chr08	E	8q21.1	3	82125000	2636	nofs	nofs
chr08	E	8q21.1	3	82375000	1225	nofs	nofs
chr08	E	8q21.1	3	82625000	1614	nofs	nofs
chr08	E	8q21.1	3	82875000	3649	nofs	nofs
chr08	E	8q21.1	3	83125000	3195	nofs	nofs
chr08	E	8q21.1	3	83375000	13865	nofs	nofs
chr08	E	8q21.1	3	83625000	3547	nofs	nofs
chr08	E	8q21.1	3	83875000	3753	nofs	nofs
chr08	E	8q21.1	3	84125000	3147	nofs	nofs
chr08	E	8q21.1	3	84375000	2510	nofs	nofs
chr08	E	8q21.1	3	84625000	3166	nofs	nofs
chr08	E	8q21.1	3	84875000	2828	nofs	nofs
chr08	E	8q21.2	2	85125000	1912	nofs	nofs
chr08	E	8q21.2	2	85375000	2042	nofs	nofs

chr08	E	8q21.2	2	85625000	2077	nofs	nofs
chr08	E	8q21.2	2	85875000	1561	nofs	nofs
chr08	E	8q21.2	2	86125000	685	nofs	nofs
chr08	E	8q21.2	2	86375000	3527	nofs	nofs
chr08	E	8q21.2	2	86625000	1999	nofs	nofs
chr08	E	8q21.2	2	86875000	3596	nofs	nofs
chr08	E	8q21.2	2	87125000	4994	nofs	nofs
chr08	B	8q21.3	.	87375000	3224	nofs	nofs
chr08	B	8q21.3	.	87625000	2587	nofs	nofs
chr08	B	8q21.3	.	87875000	3377	nofs	nofs
chr08	B	8q21.3	.	88125000	3946	nofs	nofs
chr08	B	8q21.3	.	88375000	2650	nofs	nofs
chr08	B	8q21.3	.	88625000	2071	nofs	nofs
chr08	B	8q21.3	.	88875000	2441	nofs	nofs
chr08	B	8q21.3	.	89125000	1613	nofs	nofs
chr08	B	8q21.3	.	89375000	2633	nofs	nofs
chr08	B	8q21.3	.	89625000	1911	nofs	nofs
chr08	B	8q21.3	.	89875000	2734	nofs	nofs
chr08	B	8q21.3	.	90125000	2717	nofs	nofs
chr08	B	8q21.3	.	90375000	2239	nofs	nofs
chr08	B	8q21.3	.	90625000	2265	nofs	nofs
chr08	B	8q21.3	.	90875000	2889	nofs	nofs
chr08	B	8q21.3	.	91125000	3056	nofs	nofs
chr08	B	8q21.3	.	91375000	1939	nofs	nofs
chr08	B	8q21.3	.	91625000	1537	nofs	nofs
chr08	B	8q21.3	.	91875000	2473	nofs	nofs
chr08	B	8q21.3	.	92125000	2246	nofs	nofs
chr08	B	8q21.3	.	92375000	2233	nofs	nofs
chr08	B	8q21.3	.	92625000	1190	nofs	nofs
chr08	B	8q21.3	.	92875000	3069	nofs	nofs
chr08	B	8q21.3	.	93125000	2180	nofs	nofs
chr08	B	8q21.3	.	93375000	3288	nofs	nofs
chr08	E	8q22.1	3	93625000	2424	fs	fs common
chr08	E	8q22.1	3	93875000	1659	fs	fs common
chr08	E	8q22.1	3	94125000	2571	fs	fs common
chr08	E	8q22.1	3	94375000	2234	fs	fs common

chr08	E	8q22.1	3	94625000	6387	fs	fs common
chr08	E	8q22.1	3	94875000	6738	fs	fs common
chr08	E	8q22.1	3	95125000	2853	fs	fs common
chr08	E	8q22.1	3	95375000	2608	fs	fs common
chr08	E	8q22.1	3	95625000	3284	fs	fs common
chr08	E	8q22.1	3	95875000	3858	fs	fs common
chr08	E	8q22.1	3	96125000	2534	fs	fs common
chr08	E	8q22.1	3	96375000	2089	fs	fs common
chr08	E	8q22.1	3	96625000	2553	fs	fs common
chr08	E	8q22.1	3	96875000	2460	fs	fs common
chr08	E	8q22.1	3	97125000	2284	fs	fs common
chr08	E	8q22.1	3	97375000	2621	fs	fs common
chr08	E	8q22.1	3	97625000	2715	fs	fs common
chr08	E	8q22.1	3	97875000	2274	fs	fs common
chr08	E	8q22.1	3	98125000	2105	fs	fs common
chr08	E	8q22.1	3	98375000	2376	fs	fs common
chr08	E	8q22.1	3	98625000	3380	fs	fs common
chr08	E	8q22.1	3	98875000	5675	fs	fs common
chr08	E	8q22.1	3	99125000	3484	fs	fs common
chr08	B	8q22.2	.	99375000	2396	nofs	nofs
chr08	B	8q22.2	.	99625000	1992	nofs	nofs
chr08	B	8q22.2	.	99875000	3407	nofs	nofs
chr08	B	8q22.2	.	100000000	3076	nofs	nofs
chr08	B	8q22.2	.	100000000	2020	nofs	nofs
chr08	B	8q22.2	.	101000000	1759	nofs	nofs
chr08	B	8q22.2	.	101000000	2304	nofs	nofs
chr08	B	8q22.2	.	101000000	3762	nofs	nofs
chr08	B	8q22.2	.	101000000	2016	nofs	nofs
chr08	E	8q22.3	1	102000000	2611	fs	fs rare
chr08	E	8q22.3	1	102000000	5116	fs	fs rare
chr08	E	8q22.3	1	102000000	4356	fs	fs rare
chr08	E	8q22.3	1	102000000	3143	fs	fs rare
chr08	E	8q22.3	1	103000000	5026	fs	fs rare
chr08	E	8q22.3	1	103000000	1712	fs	fs rare
chr08	E	8q22.3	1	103000000	2040	fs	fs rare
chr08	E	8q22.3	1	103000000	1364	fs	fs rare

chr08	E	8q22.3	1	104000000	4017	fs	fs rare
chr08	E	8q22.3	1	104000000	4673	fs	fs rare
chr08	E	8q22.3	1	104000000	3830	fs	fs rare
chr08	E	8q22.3	1	104000000	2357	fs	fs rare
chr08	E	8q22.3	1	105000000	1885	fs	fs rare
chr08	E	8q22.3	1	105000000	2108	fs	fs rare
chr08	E	8q22.3	1	105000000	2729	fs	fs rare
chr08	E	8q22.3	1	105000000	1993	fs	fs rare
chr08	E	8q22.3	1	106000000	3586	fs	fs rare
chr08	E	8q22.3	1	106000000	1770	fs	fs rare
chr08	E	8q23	1	106000000	1845	nofs	nofs
chr08	E	8q23	1	106000000	3504	nofs	nofs
chr08	E	8q23	1	107000000	2901	nofs	nofs
chr08	E	8q23	1	107000000	3688	nofs	nofs
chr08	E	8q23	1	107000000	2374	nofs	nofs
chr08	E	8q23	1	107000000	2608	nofs	nofs
chr08	E	8q23	1	108000000	1820	nofs	nofs
chr08	E	8q23	1	108000000	3766	nofs	nofs
chr08	E	8q23	1	108000000	1551	nofs	nofs
chr08	E	8q23	1	108000000	3596	nofs	nofs
chr08	E	8q23	1	109000000	22860	nofs	nofs
chr08	E	8q23	1	109000000	2204	nofs	nofs
chr08	E	8q23	1	109000000	2309	nofs	nofs
chr08	E	8q23	1	109000000	1787	nofs	nofs
chr08	E	8q23	1	110000000	1647	nofs	nofs
chr08	E	8q23	1	110000000	2012	nofs	nofs
chr08	E	8q23	1	110000000	1427	nofs	nofs
chr08	E	8q23	1	110000000	1582	nofs	nofs
chr08	E	8q23	1	111000000	1781	nofs	nofs
chr08	E	8q23	1	111000000	2004	nofs	nofs
chr08	E	8q23	1	111000000	2623	nofs	nofs
chr08	E	8q23	1	111000000	2270	nofs	nofs
chr08	E	8q23	1	112000000	2572	nofs	nofs
chr08	E	8q23	1	112000000	2829	nofs	nofs
chr08	E	8q23	1	112000000	2404	nofs	nofs
chr08	E	8q23	1	112000000	2512	nofs	nofs

chr08	E	8q23	1	113000000	2963	nofs	nofs
chr08	E	8q23	1	113000000	2162	nofs	nofs
chr08	E	8q23	1	113000000	2373	nofs	nofs
chr08	E	8q23	1	113000000	2277	nofs	nofs
chr08	E	8q23	1	114000000	1911	nofs	nofs
chr08	E	8q23	1	114000000	2680	nofs	nofs
chr08	E	8q23	1	114000000	2118	nofs	nofs
chr08	E	8q23	1	114000000	2576	nofs	nofs
chr08	E	8q23	1	115000000	2539	nofs	nofs
chr08	E	8q23	1	115000000	2276	nofs	nofs
chr08	E	8q23	1	115000000	1594	nofs	nofs
chr08	E	8q23	1	115000000	2996	nofs	nofs
chr08	E	8q23	1	116000000	2367	nofs	nofs
chr08	E	8q23	1	116000000	2327	nofs	nofs
chr08	E	8q23	1	116000000	2733	nofs	nofs
chr08	E	8q23	1	116000000	1866	nofs	nofs
chr08	E	8q23	1	117000000	3295	nofs	nofs
chr08	E	8q23	1	117000000	3084	nofs	nofs
chr08	E	8q23	1	117000000	1908	nofs	nofs
chr08	E	8q23	1	117000000	4341	nofs	nofs
chr08	E	8q23	1	118000000	2403	nofs	nofs
chr08	E	8q24.1	2	118000000	3732	fs	fs common and rare
chr08	E	8q24.1	2	118000000	2905	fs	fs common and rare
chr08	E	8q24.1	2	118000000	1343	fs	fs common and rare
chr08	E	8q24.1	2	119000000	2473	fs	fs common and rare
chr08	E	8q24.1	2	119000000	2578	fs	fs common and rare
chr08	E	8q24.1	2	119000000	2780	fs	fs common and rare
chr08	E	8q24.1	2	119000000	1603	fs	fs common and rare
chr08	E	8q24.1	2	120000000	1558	fs	fs common and rare
chr08	E	8q24.1	2	120000000	1517	fs	fs common and rare
chr08	E	8q24.1	2	120000000	1828	fs	fs common and rare
chr08	E	8q24.1	2	120000000	1773	fs	fs common and rare
chr08	E	8q24.1	2	121000000	4820	fs	fs common and rare
chr08	E	8q24.1	2	121000000	3719	fs	fs common and rare
chr08	E	8q24.1	2	121000000	2699	fs	fs common and rare
chr08	E	8q24.1	2	121000000	2720	fs	fs common and rare

chr08	E	8q24.1	2	122000000	2751	fs	fs common and rare
chr08	E	8q24.1	2	122000000	2176	fs	fs common and rare
chr08	E	8q24.1	2	122000000	3522	fs	fs common and rare
chr08	E	8q24.1	2	122000000	3139	fs	fs common and rare
chr08	E	8q24.1	2	123000000	3396	fs	fs common and rare
chr08	E	8q24.1	2	123000000	2448	fs	fs common and rare
chr08	E	8q24.1	2	123000000	3069	fs	fs common and rare
chr08	E	8q24.1	2	123000000	2275	fs	fs common and rare
chr08	E	8q24.1	2	124000000	3031	fs	fs common and rare
chr08	E	8q24.1	2	124000000	1961	fs	fs common and rare
chr08	E	8q24.1	2	124000000	3255	fs	fs common and rare
chr08	E	8q24.1	2	124000000	3950	fs	fs common and rare
chr08	E	8q24.1	2	125000000	3900	fs	fs common and rare
chr08	E	8q24.1	2	125000000	2361	fs	fs common and rare
chr08	E	8q24.1	2	125000000	1572	fs	fs common and rare
chr08	E	8q24.1	2	125000000	4542	fs	fs common and rare
chr08	E	8q24.1	2	126000000	3123	fs	fs common and rare
chr08	E	8q24.1	2	126000000	3295	fs	fs common and rare
chr08	E	8q24.1	2	126000000	1960	fs	fs common and rare
chr08	E	8q24.1	2	126000000	3151	fs	fs common and rare
chr08	E	8q24.1	2	127000000	3897	fs	fs common and rare
chr08	E	8q24.1	2	127000000	2229	fs	fs common and rare
chr08	E	8q24.1	2	127000000	2309	fs	fs common and rare
chr08	E	8q24.1	2	127000000	2729	fs	fs common and rare
chr08	B	8q24.2	.	128000000	2524	nofs	nofs
chr08	B	8q24.2	.	128000000	2837	nofs	nofs
chr08	B	8q24.2	.	128000000	2368	nofs	nofs
chr08	B	8q24.2	.	128000000	3683	nofs	nofs
chr08	B	8q24.2	.	129000000	2531	nofs	nofs
chr08	B	8q24.2	.	129000000	5468	nofs	nofs
chr08	B	8q24.2	.	129000000	4053	nofs	nofs
chr08	B	8q24.2	.	129000000	4106	nofs	nofs
chr08	B	8q24.2	.	130000000	2497	nofs	nofs
chr08	B	8q24.2	.	130000000	3135	nofs	nofs
chr08	B	8q24.2	.	130000000	1927	nofs	nofs
chr08	B	8q24.2	.	130000000	1962	nofs	nofs

chr08	B	8q24.2	.	131000000	4546	nofs	nofs
chr08	B	8q24.2	.	131000000	2926	nofs	nofs
chr08	B	8q24.2	.	131000000	4430	nofs	nofs
chr08	B	8q24.2	.	131000000	1737	nofs	nofs
chr08	B	8q24.2	.	132000000	4898	nofs	nofs
chr08	B	8q24.2	.	132000000	3859	nofs	nofs
chr08	B	8q24.2	.	132000000	1770	nofs	nofs
chr08	B	8q24.2	.	132000000	3489	nofs	nofs
chr08	B	8q24.2	.	133000000	1926	nofs	nofs
chr08	B	8q24.2	.	133000000	2070	nofs	nofs
chr08	B	8q24.2	.	133000000	2447	nofs	nofs
chr08	B	8q24.2	.	133000000	2668	nofs	nofs
chr08	B	8q24.2	.	134000000	2440	nofs	nofs
chr08	B	8q24.2	.	134000000	2930	nofs	nofs
chr08	B	8q24.2	.	134000000	3059	nofs	nofs
chr08	B	8q24.2	.	134000000	6020	nofs	nofs
chr08	B	8q24.2	.	135000000	3702	nofs	nofs
chr08	B	8q24.2	.	135000000	5529	nofs	nofs
chr08	B	8q24.2	.	135000000	2375	nofs	nofs
chr08	B	8q24.2	.	135000000	3127	nofs	nofs
chr08	B	8q24.2	.	136000000	9564	nofs	nofs
chr08	B	8q24.2	.	136000000	1976	nofs	nofs
chr08	B	8q24.2	.	136000000	2164	nofs	nofs
chr08	B	8q24.2	.	136000000	2941	nofs	nofs
chr08	B	8q24.2	.	137000000	2457	nofs	nofs
chr08	B	8q24.2	.	137000000	2960	nofs	nofs
chr08	B	8q24.2	.	137000000	2347	nofs	nofs
chr08	B	8q24.2	.	137000000	2839	nofs	nofs
chr08	B	8q24.2	.	138000000	2127	nofs	nofs
chr08	B	8q24.2	.	138000000	2417	nofs	nofs
chr08	B	8q24.2	.	138000000	4532	nofs	nofs
chr08	B	8q24.2	.	138000000	5369	nofs	nofs
chr08	B	8q24.2	.	139000000	2898	nofs	nofs
chr08	B	8q24.2	.	139000000	4585	nofs	nofs
chr08	B	8q24.2	.	139000000	2546	nofs	nofs
chr08	B	8q24.2	.	139000000	3341	nofs	nofs

chr08	B	8q24.2	.	140000000	3737	nofs	nofs
chr08	B	8q24.3	.	140000000	7774	fs	fs common
chr08	B	8q24.3	.	140000000	3415	fs	fs common
chr08	B	8q24.3	.	140000000	2189	fs	fs common
chr08	B	8q24.3	.	141000000	1695	fs	fs common
chr08	B	8q24.3	.	141000000	11623	fs	fs common
chr08	B	8q24.3	.	141000000	8245	fs	fs common
chr08	B	8q24.3	.	141000000	7455	fs	fs common
chr08	B	8q24.3	.	142000000	6072	fs	fs common
chr08	B	8q24.3	.	142000000	2658	fs	fs common
chr08	B	8q24.3	.	142000000	3992	fs	fs common
chr08	B	8q24.3	.	142000000	8929	fs	fs common
chr08	B	8q24.3	.	143000000	19431	fs	fs common
chr08	B	8q24.3	.	143000000	13360	fs	fs common
chr08	T	8q24.3	.	143000000	20925	fs	fs common
chr08	T	8q24.3	.	143000000	26395	fs	fs common
chr08	T	8q24.3	.	144000000	18742	fs	fs common
chr08	T	8q24.3	.	144000000	13605	fs	fs common
chr08	T	8q24.3	.	144000000	10008	fs	fs common
chr08	T	8q24.3	.	144000000	23721	fs	fs common
chr08	T	8q24.3	.	145000000	11171	fs	fs common
chr08	T	8q24.3	.	145000000	10687	fs	fs common
chr08	T	8q24.3	.	145000000	5848	fs	fs common
chr08	T	8q24.3	.	145000000	9382	fs	fs common
chr08	T	8q24.3	.	146000000	11639	fs	fs common
chr08	T	8q24.3	.	146000000	20902	fs	fs common
chr08	T	8q24.3	.	146000000	8437	fs	fs common
chr09	T	9p24	4	125000	5034	nofs	nofs
chr09	T	9p24	4	375000	6546	nofs	nofs
chr09	T	9p24	4	625000	3175	nofs	nofs
chr09	T	9p24	4	875000	3771	nofs	nofs
chr09	T	9p24	4	1125000	1480	nofs	nofs
chr09	T	9p24	4	1375000	1307	nofs	nofs
chr09	T	9p24	4	1625000	1478	nofs	nofs
chr09	T	9p24	4	1875000	2912	nofs	nofs
chr09	T	9p24	4	2125000	1853	nofs	nofs

chr09	T	9p24	4	2375000	2480	nofs	nofs
chr09	T	9p24	4	2625000	2610	nofs	nofs
chr09	T	9p24	4	2875000	1160	nofs	nofs
chr09	T	9p24	4	3125000	2180	nofs	nofs
chr09	E	9p24	4	3375000	4657	nofs	nofs
chr09	E	9p24	4	3625000	1590	nofs	nofs
chr09	E	9p24	4	3875000	2009	nofs	nofs
chr09	E	9p24	4	4125000	1535	nofs	nofs
chr09	E	9p24	4	4375000	2436	nofs	nofs
chr09	E	9p24	4	4625000	3031	nofs	nofs
chr09	E	9p24	4	4875000	1780	nofs	nofs
chr09	E	9p24	4	5125000	1209	nofs	nofs
chr09	E	9p24	4	5375000	3969	nofs	nofs
chr09	E	9p24	4	5625000	2036	nofs	nofs
chr09	E	9p24	4	5875000	2015	nofs	nofs
chr09	E	9p24	4	6125000	2455	nofs	nofs
chr09	E	9p24	4	6375000	3166	nofs	nofs
chr09	E	9p24	4	6625000	5288	nofs	nofs
chr09	E	9p24	4	6875000	4066	nofs	nofs
chr09	E	9p24	4	7125000	2317	nofs	nofs
chr09	E	9p24	4	7375000	1668	nofs	nofs
chr09	E	9p24	4	7625000	1611	nofs	nofs
chr09	E	9p24	4	7875000	1282	nofs	nofs
chr09	E	9p24	4	8125000	1751	nofs	nofs
chr09	E	9p24	4	8375000	2231	nofs	nofs
chr09	E	9p24	4	8625000	1797	nofs	nofs
chr09	E	9p24	4	8875000	2787	nofs	nofs
chr09	E	9p24	4	9125000	1777	nofs	nofs
chr09	E	9p23	1	9375000	1972	nofs	nofs
chr09	E	9p23	1	9625000	1516	nofs	nofs
chr09	E	9p23	1	9875000	2795	nofs	nofs
chr09	E	9p23	1	10125000	3187	nofs	nofs
chr09	E	9p23	1	10375000	2639	nofs	nofs
chr09	E	9p23	1	10625000	2478	nofs	nofs
chr09	E	9p23	1	10875000	3237	nofs	nofs
chr09	E	9p23	1	11125000	2383	nofs	nofs

chr09	E	9p23	1	11375000	2553	nofs	nofs
chr09	E	9p23	1	11625000	1948	nofs	nofs
chr09	E	9p23	1	11875000	3459	nofs	nofs
chr09	E	9p23	1	12125000	2687	nofs	nofs
chr09	E	9p23	1	12375000	2971	nofs	nofs
chr09	E	9p23	1	12625000	2486	nofs	nofs
chr09	E	9p23	1	12875000	1987	nofs	nofs
chr09	E	9p23	1	13125000	2128	nofs	nofs
chr09	E	9p23	1	13375000	1274	nofs	nofs
chr09	E	9p23	1	13625000	1886	nofs	nofs
chr09	E	9p23	1	13875000	2483	nofs	nofs
chr09	E	9p23	1	14125000	3243	nofs	nofs
chr09	B	9p22	.	14375000	1935	nofs	nofs
chr09	B	9p22	.	14625000	1655	nofs	nofs
chr09	B	9p22	.	14875000	6047	nofs	nofs
chr09	B	9p22	.	15125000	2962	nofs	nofs
chr09	B	9p22	.	15375000	4176	nofs	nofs
chr09	B	9p22	.	15625000	1975	nofs	nofs
chr09	B	9p22	.	15875000	1797	nofs	nofs
chr09	B	9p22	.	16125000	1538	nofs	nofs
chr09	B	9p22	.	16375000	2102	nofs	nofs
chr09	B	9p22	.	16625000	1684	nofs	nofs
chr09	B	9p22	.	16875000	4186	nofs	nofs
chr09	B	9p22	.	17125000	3042	nofs	nofs
chr09	B	9p22	.	17375000	1976	nofs	nofs
chr09	B	9p22	.	17625000	1919	nofs	nofs
chr09	B	9p22	.	17875000	2151	nofs	nofs
chr09	B	9p22	.	18125000	2096	nofs	nofs
chr09	B	9p22	.	18375000	2097	nofs	nofs
chr09	B	9p22	.	18625000	1519	nofs	nofs
chr09	B	9p22	.	18875000	3279	nofs	nofs
chr09	B	9p22	.	19125000	4152	nofs	nofs
chr09	B	9p22	.	19375000	4117	nofs	nofs
chr09	B	9p22	.	19625000	2186	nofs	nofs
chr09	E	9p21	5	19875000	2531	fs	fs common and rare
chr09	E	9p21	5	20125000	1995	fs	fs common and rare

chr09	E	9p21	5	20375000	1505	fs	fs common and rare
chr09	E	9p21	5	20625000	2328	fs	fs common and rare
chr09	E	9p21	5	20875000	3381	fs	fs common and rare
chr09	E	9p21	5	21125000	2391	fs	fs common and rare
chr09	E	9p21	5	21375000	1918	fs	fs common and rare
chr09	E	9p21	5	21625000	1831	fs	fs common and rare
chr09	E	9p21	5	21875000	2351	fs	fs common and rare
chr09	E	9p21	5	22125000	2006	fs	fs common and rare
chr09	E	9p21	5	22375000	3463	fs	fs common and rare
chr09	E	9p21	5	22625000	2204	fs	fs common and rare
chr09	E	9p21	5	22875000	2380	fs	fs common and rare
chr09	E	9p21	5	23125000	1314	fs	fs common and rare
chr09	E	9p21	5	23375000	2296	fs	fs common and rare
chr09	E	9p21	5	23625000	1744	fs	fs common and rare
chr09	E	9p21	5	23875000	2639	fs	fs common and rare
chr09	E	9p21	5	24125000	1693	fs	fs common and rare
chr09	E	9p21	5	24375000	2020	fs	fs common and rare
chr09	E	9p21	5	24625000	2389	fs	fs common and rare
chr09	E	9p21	5	24875000	2304	fs	fs common and rare
chr09	E	9p21	5	25125000	4405	fs	fs common and rare
chr09	E	9p21	5	25375000	3382	fs	fs common and rare
chr09	E	9p21	5	25625000	3104	fs	fs common and rare
chr09	E	9p21	5	25875000	3296	fs	fs common and rare
chr09	E	9p21	5	26125000	3521	fs	fs common and rare
chr09	E	9p21	5	26375000	3387	fs	fs common and rare
chr09	E	9p21	5	26625000	2270	fs	fs common and rare
chr09	E	9p21	5	26875000	2876	fs	fs common and rare
chr09	E	9p21	5	27125000	1854	fs	fs common and rare
chr09	E	9p21	5	27375000	1738	fs	fs common and rare
chr09	E	9p21	5	27625000	1899	fs	fs common and rare
chr09	E	9p21	5	27875000	1028	fs	fs common and rare
chr09	E	9p21	5	28125000	3399	fs	fs common and rare
chr09	E	9p21	5	28375000	3549	fs	fs common and rare
chr09	E	9p21	5	28625000	2410	fs	fs common and rare
chr09	E	9p21	5	28875000	3492	fs	fs common and rare
chr09	E	9p21	5	29125000	2931	fs	fs common and rare

chr09	E	9p21	5	29375000	2082	fs	fs common and rare
chr09	E	9p21	5	29625000	2122	fs	fs common and rare
chr09	E	9p21	5	29875000	2683	fs	fs common and rare
chr09	E	9p21	5	30125000	2852	fs	fs common and rare
chr09	E	9p21	5	30375000	2555	fs	fs common and rare
chr09	E	9p21	5	30625000	1865	fs	fs common and rare
chr09	E	9p21	5	30875000	2528	fs	fs common and rare
chr09	E	9p21	5	31125000	2288	fs	fs common and rare
chr09	E	9p21	5	31375000	3280	fs	fs common and rare
chr09	E	9p21	5	31625000	1878	fs	fs common and rare
chr09	E	9p21	5	31875000	1331	fs	fs common and rare
chr09	E	9p21	5	32125000	1191	fs	fs common and rare
chr09	E	9p21	5	32375000	2783	fs	fs common and rare
chr09	E	9p21	5	32625000	1967	fs	fs common and rare
chr09	E	9p21	5	32875000	3743	fs	fs common and rare
chr09	E	9p21	5	33125000	3086	fs	fs common and rare
chr09	E	9p21	5	33375000	2870	fs	fs common and rare
chr09	E	9p13	2	33625000	4152	nofs	nofs
chr09	E	9p13	2	33875000	4184	nofs	nofs
chr09	E	9p13	2	34125000	6198	nofs	nofs
chr09	E	9p13	2	34375000	2883	nofs	nofs
chr09	E	9p13	2	34625000	3210	nofs	nofs
chr09	E	9p13	2	34875000	2753	nofs	nofs
chr09	E	9p13	2	35125000	4862	nofs	nofs
chr09	E	9p13	2	35375000	5553	nofs	nofs
chr09	E	9p13	2	35625000	1816	nofs	nofs
chr09	E	9p13	2	35875000	5032	nofs	nofs
chr09	E	9p13	2	36125000	2524	nofs	nofs
chr09	E	9p13	2	36375000	3576	nofs	nofs
chr09	E	9p13	2	36625000	5383	nofs	nofs
chr09	E	9p13	2	36875000	3792	nofs	nofs
chr09	E	9p13	2	37125000	4692	nofs	nofs
chr09	E	9p13	2	37375000	3032	nofs	nofs
chr09	E	9p13	2	37625000	4067	nofs	nofs
chr09	E	9p13	2	37875000	2490	nofs	nofs
chr09	E	9p13	2	38125000	4160	nofs	nofs

chr09	E	9p13	2	38375000	4006	nofs	nofs
chr09	E	9p13	2	38625000	3482	nofs	nofs
chr09	E	9p13	2	38875000	2429	nofs	nofs
chr09	E	9p13	2	39125000	2455	nofs	nofs
chr09	E	9p13	2	39375000	2697	nofs	nofs
chr09	E	9p13	2	39625000	3110	nofs	nofs
chr09	E	9p13	2	39875000	1136	nofs	nofs
chr09	E	9p13	2	40125000	2061	nofs	nofs
chr09	E	9p13	2	40375000	2528	nofs	nofs
chr09	E	9p13	2	40625000	3090	nofs	nofs
chr09	E	9p13	2	40875000	2147	nofs	nofs
chr09	E	9p13	2	41125000	2713	nofs	nofs
chr09	E	9p13	2	41375000	1807	nofs	nofs
chr09	B	9p12	.	41625000	3025	nofs	nofs
chr09	B	9p12	.	41875000	1250	nofs	nofs
chr09	B	9p12	.	42125000	2774	nofs	nofs
chr09	B	9p12	.	42375000	1582	nofs	nofs
chr09	B	9p12	.	42625000	1223	nofs	nofs
chr09	C	9p12	.	42875000	1777	nofs	nofs
chr09	C	9p12	.	43125000	2376	nofs	nofs
chr09	C	9p12	.	43375000	2865	nofs	nofs
chr09	C	9p12	.	43625000	1216	nofs	nofs
chr09	C	9p12	.	43875000	9834	nofs	nofs
chr09	C	9p12	.	44125000	6590	nofs	nofs
chr09	C	9p12	.	44375000	7392	nofs	nofs
chr09	C	9p11	.	44625000	2136	nofs	nofs
chr09	C	9p11	.	44875000	3615	nofs	nofs
chr09	C	9p11	.	45125000	1123	nofs	nofs
chr09	C	9p11	.	45375000	5925	nofs	nofs
chr09	C	9p11	.	45625000	1324	nofs	nofs
chr09	C	9p11	.	45875000	833	nofs	nofs
chr09	C		.	46125000	0	nofs	nofs
chr09	C		.	46375000	0	nofs	nofs
chr09	C		.	46625000	0	nofs	nofs
chr09	C		.	46875000	0	nofs	nofs
chr09	C		.	47125000	0	nofs	nofs

chr09	C	.	47375000	0	nofs	nofs
chr09	C	.	47625000	0	nofs	nofs
chr09	C	.	47875000	0	nofs	nofs
chr09	C	.	48125000	0	nofs	nofs
chr09	C	.	48375000	0	nofs	nofs
chr09	C	.	48625000	0	nofs	nofs
chr09	C	.	48875000	0	nofs	nofs
chr09	C	.	49125000	0	nofs	nofs
chr09	C	.	49375000	0	nofs	nofs
chr09	C	.	49625000	0	nofs	nofs
chr09	C	.	49875000	0	nofs	nofs
chr09	C	.	50125000	0	nofs	nofs
chr09	C	.	50375000	0	nofs	nofs
chr09	C	.	50625000	0	nofs	nofs
chr09	C	.	50875000	0	nofs	nofs
chr09	C	.	51125000	0	nofs	nofs
chr09	C	.	51375000	0	nofs	nofs
chr09	C	.	51625000	0	nofs	nofs
chr09	C	.	51875000	0	nofs	nofs
chr09	C	.	52125000	0	nofs	nofs
chr09	C	.	52375000	0	nofs	nofs
chr09	C	.	52625000	0	nofs	nofs
chr09	C	.	52875000	0	nofs	nofs
chr09	C	.	53125000	0	nofs	nofs
chr09	C	.	53375000	0	nofs	nofs
chr09	C	.	53625000	0	nofs	nofs
chr09	C	.	53875000	0	nofs	nofs
chr09	C	.	54125000	0	nofs	nofs
chr09	C	.	54375000	0	nofs	nofs
chr09	C	.	54625000	0	nofs	nofs
chr09	C	.	54875000	0	nofs	nofs
chr09	C	.	55125000	0	nofs	nofs
chr09	C	.	55375000	0	nofs	nofs
chr09	C	.	55625000	0	nofs	nofs
chr09	C	.	55875000	0	nofs	nofs
chr09	C	.	56125000	0	nofs	nofs

chr09	C		.	56375000	0	nofs	nofs
chr09	C		.	56625000	0	nofs	nofs
chr09	C		.	56875000	0	nofs	nofs
chr09	C		.	57125000	0	nofs	nofs
chr09	C		.	57375000	0	nofs	nofs
chr09	C		.	57625000	0	nofs	nofs
chr09	C		.	57875000	0	nofs	nofs
chr09	C		.	58125000	0	nofs	nofs
chr09	C		.	58375000	0	nofs	nofs
chr09	C		.	58625000	0	nofs	nofs
chr09	C		.	58875000	0	nofs	nofs
chr09	C		.	59125000	0	nofs	nofs
chr09	C		.	59375000	0	nofs	nofs
chr09	C		.	59625000	0	nofs	nofs
chr09	C		.	59875000	0	nofs	nofs
chr09	C		.	60125000	0	nofs	nofs
chr09	C		.	60375000	0	nofs	nofs
chr09	C		.	60625000	0	nofs	nofs
chr09	C		.	60875000	0	nofs	nofs
chr09	C		.	61125000	0	nofs	nofs
chr09	C		.	61375000	0	nofs	nofs
chr09	C		.	61625000	0	nofs	nofs
chr09	C		.	61875000	0	nofs	nofs
chr09	C		.	62125000	0	nofs	nofs
chr09	C		.	62375000	0	nofs	nofs
chr09	C		.	62625000	0	nofs	nofs
chr09	C		.	62875000	0	nofs	nofs
chr09	C		.	63125000	0	nofs	nofs
chr09	C		.	63375000	0	nofs	nofs
chr09	C		.	63625000	0	nofs	nofs
chr09	C		.	63875000	0	nofs	nofs
chr09	C	9q12	.	64125000	1490	fs	fs common
chr09	C	9q12	.	64375000	53271	fs	fs common
chr09	C	9q12	.	64625000	92238	fs	fs common
chr09	C	9q12	.	64875000	16611	fs	fs common
chr09	C	9q12	.	65125000	490	fs	fs common

chr09	C	9q12	.	65375000	2085	fs	fs common
chr09	C	9q12	.	65625000	41828	fs	fs common
chr09	C	9q12	.	65875000	14185	fs	fs common
chr09	C	9q12	.	66125000	6261	fs	fs common
chr09	C	9q12	.	66375000	1785	fs	fs common
chr09	C	9q12	.	66625000	6970	fs	fs common
chr09	C	9q12	.	66875000	3978	fs	fs common
chr09	C	9q12	.	67125000	16887	fs	fs common
chr09	B	9q12	.	67375000	1009	fs	fs common
chr09	B	9q13	.	67625000	47025	nofs	nofs
chr09	B	9q13	.	67875000	19161	nofs	nofs
chr09	B	9q13	.	68125000	7683	nofs	nofs
chr09	B	9q13	.	68375000	1969	nofs	nofs
chr09	B	9q13	.	68625000	1966	nofs	nofs
chr09	B	9q21.1	.	68875000	2366	nofs	nofs
chr09	B	9q21.1	.	69125000	3455	nofs	nofs
chr09	B	9q21.1	.	69375000	2541	nofs	nofs
chr09	B	9q21.1	.	69625000	2360	nofs	nofs
chr09	B	9q21.1	.	69875000	1876	nofs	nofs
chr09	B	9q21.1	.	70125000	1769	nofs	nofs
chr09	B	9q21.1	.	70375000	2205	nofs	nofs
chr09	B	9q21.1	.	70625000	2473	nofs	nofs
chr09	B	9q21.1	.	70875000	2097	nofs	nofs
chr09	B	9q21.1	.	71125000	2487	nofs	nofs
chr09	B	9q21.1	.	71375000	3088	nofs	nofs
chr09	B	9q21.1	.	71625000	3248	nofs	nofs
chr09	B	9q21.1	.	71875000	2303	nofs	nofs
chr09	B	9q21.1	.	72125000	2671	nofs	nofs
chr09	B	9q21.1	.	72375000	4363	nofs	nofs
chr09	B	9q21.1	.	72625000	5127	nofs	nofs
chr09	B	9q21.1	.	72875000	2105	nofs	nofs
chr09	B	9q21.1	.	73125000	2256	nofs	nofs
chr09	B	9q21.1	.	73375000	1939	nofs	nofs
chr09	B	9q21.1	.	73625000	1792	nofs	nofs
chr09	B	9q21.1	.	73875000	2726	nofs	nofs
chr09	B	9q21.1	.	74125000	4589	nofs	nofs

chr09	B	9q21.1	.	74375000	1930	nofs	nofs
chr09	B	9q21.1	.	74625000	2945	nofs	nofs
chr09	B	9q21.1	.	74875000	2665	nofs	nofs
chr09	B	9q21.1	.	75125000	2711	nofs	nofs
chr09	B	9q21.1	.	75375000	3355	nofs	nofs
chr09	B	9q21.1	.	75625000	1707	nofs	nofs
chr09	B	9q21.1	.	75875000	2616	nofs	nofs
chr09	B	9q21.1	.	76125000	5111	nofs	nofs
chr09	B	9q21.1	.	76375000	2433	nofs	nofs
chr09	E	9q21.2	2	76625000	2607	nofs	nofs
chr09	E	9q21.2	2	76875000	3797	nofs	nofs
chr09	E	9q21.2	2	77125000	1717	nofs	nofs
chr09	E	9q21.2	2	77375000	2202	nofs	nofs
chr09	E	9q21.2	2	77625000	5312	nofs	nofs
chr09	E	9q21.2	2	77875000	1252	nofs	nofs
chr09	E	9q21.2	2	78125000	4112	nofs	nofs
chr09	E	9q21.2	2	78375000	2894	nofs	nofs
chr09	E	9q21.3	4	78625000	2282	nofs	nofs
chr09	E	9q21.3	4	78875000	2801	nofs	nofs
chr09	E	9q21.3	4	79125000	2464	nofs	nofs
chr09	E	9q21.3	4	79375000	1666	nofs	nofs
chr09	E	9q21.3	4	79625000	2947	nofs	nofs
chr09	E	9q21.3	4	79875000	3227	nofs	nofs
chr09	E	9q21.3	4	80125000	2136	nofs	nofs
chr09	E	9q21.3	4	80375000	2129	nofs	nofs
chr09	E	9q21.3	4	80625000	3183	nofs	nofs
chr09	E	9q21.3	4	80875000	2993	nofs	nofs
chr09	E	9q21.3	4	81125000	1703	nofs	nofs
chr09	E	9q21.3	4	81375000	1619	nofs	nofs
chr09	E	9q21.3	4	81625000	6592	nofs	nofs
chr09	E	9q21.3	4	81875000	2750	nofs	nofs
chr09	E	9q21.3	4	82125000	1496	nofs	nofs
chr09	E	9q21.3	4	82375000	3039	nofs	nofs
chr09	E	9q21.3	4	82625000	2524	nofs	nofs
chr09	E	9q21.3	4	82875000	2461	nofs	nofs
chr09	E	9q21.3	4	83125000	3917	nofs	nofs

chr09	E	9q21.3	4	83375000	3631	nofs	nofs
chr09	E	9q21.3	4	83625000	3626	nofs	nofs
chr09	E	9q21.3	4	83875000	3053	nofs	nofs
chr09	E	9q21.3	4	84125000	2953	nofs	nofs
chr09	E	9q21.3	4	84375000	5058	nofs	nofs
chr09	E	9q21.3	4	84625000	2545	nofs	nofs
chr09	E	9q21.3	4	84875000	1940	nofs	nofs
chr09	E	9q21.3	4	85125000	3039	nofs	nofs
chr09	E	9q21.3	4	85375000	3689	nofs	nofs
chr09	E	9q21.3	4	85625000	2418	nofs	nofs
chr09	E	9q21.3	4	85875000	5885	nofs	nofs
chr09	E	9q21.3	4	86125000	2940	nofs	nofs
chr09	E	9q21.3	4	86375000	3708	nofs	nofs
chr09	E	9q21.3	4	86625000	2299	nofs	nofs
chr09	E	9q21.3	4	86875000	2198	nofs	nofs
chr09	E	9q21.3	4	87125000	2997	nofs	nofs
chr09	E	9q21.3	4	87375000	6028	nofs	nofs
chr09	E	9q22.1	3	87625000	6466	fs	fs common
chr09	E	9q22.1	3	87875000	3061	fs	fs common
chr09	E	9q22.1	3	88125000	7697	fs	fs common
chr09	E	9q22.1	3	88375000	4727	fs	fs common
chr09	E	9q22.1	3	88625000	5902	fs	fs common
chr09	E	9q22.1	3	88875000	5381	fs	fs common
chr09	E	9q22.2	2	89125000	5946	nofs	nofs
chr09	E	9q22.2	2	89375000	4094	nofs	nofs
chr09	E	9q22.2	2	89625000	3937	nofs	nofs
chr09	E	9q22.2	2	89875000	2791	nofs	nofs
chr09	E	9q22.2	2	90125000	4952	nofs	nofs
chr09	E	9q22.2	2	90375000	2581	nofs	nofs
chr09	E	9q22.2	2	90625000	6996	nofs	nofs
chr09	E	9q22.2	2	90875000	3924	nofs	nofs
chr09	E	9q22.3	4	91125000	1683	nofs	nofs
chr09	E	9q22.3	4	91375000	3911	nofs	nofs
chr09	E	9q22.3	4	91625000	6948	nofs	nofs
chr09	E	9q22.3	4	91875000	2705	nofs	nofs
chr09	E	9q22.3	4	92125000	3167	nofs	nofs

chr09	E	9q22.3	4	92375000	2434	nofs	nofs
chr09	E	9q22.3	4	92625000	3985	nofs	nofs
chr09	E	9q22.3	4	92875000	4321	nofs	nofs
chr09	E	9q22.3	4	93125000	5006	nofs	nofs
chr09	E	9q22.3	4	93375000	3549	nofs	nofs
chr09	E	9q22.3	4	93625000	7156	nofs	nofs
chr09	E	9q22.3	4	93875000	2344	nofs	nofs
chr09	E	9q22.3	4	94125000	3402	nofs	nofs
chr09	E	9q22.3	4	94375000	3332	nofs	nofs
chr09	E	9q22.3	4	94625000	3812	nofs	nofs
chr09	E	9q22.3	4	94875000	3478	nofs	nofs
chr09	E	9q22.3	4	95125000	3466	nofs	nofs
chr09	E	9q22.3	4	95375000	4661	nofs	nofs
chr09	E	9q22.3	4	95625000	3514	nofs	nofs
chr09	E	9q22.3	4	95875000	3708	nofs	nofs
chr09	E	9q22.3	4	96125000	4253	nofs	nofs
chr09	E	9q22.3	4	96375000	3420	nofs	nofs
chr09	E	9q22.3	4	96625000	5656	nofs	nofs
chr09	E	9q22.3	4	96875000	3041	nofs	nofs
chr09	E	9q22.3	4	97125000	3460	nofs	nofs
chr09	E	9q22.3	4	97375000	2386	nofs	nofs
chr09	E	9q22.3	4	97625000	2540	nofs	nofs
chr09	E	9q22.3	4	97875000	3285	nofs	nofs
chr09	E	9q22.3	4	98125000	3293	nofs	nofs
chr09	E	9q22.3	4	98375000	3854	nofs	nofs
chr09	E	9q22.3	4	98625000	1836	nofs	nofs
chr09	E	9q22.3	4	98875000	2730	nofs	nofs
chr09	E	9q22.3	4	99125000	3768	nofs	nofs
chr09	E	9q22.3	4	99375000	2628	nofs	nofs
chr09	E	9q22.3	4	99625000	3171	nofs	nofs
chr09	E	9q31	1	99875000	3366	nofs	nofs
chr09	E	9q31	1	100000000	2378	nofs	nofs
chr09	E	9q31	1	100000000	3110	nofs	nofs
chr09	E	9q31	1	101000000	2648	nofs	nofs
chr09	E	9q31	1	101000000	2169	nofs	nofs
chr09	E	9q31	1	101000000	2064	nofs	nofs

chr09	E	9q31	1	101000000	2728	nofs	nofs
chr09	E	9q31	1	102000000	2078	nofs	nofs
chr09	E	9q31	1	102000000	1928	nofs	nofs
chr09	E	9q31	1	102000000	2567	nofs	nofs
chr09	E	9q31	1	102000000	3511	nofs	nofs
chr09	E	9q31	1	103000000	3274	nofs	nofs
chr09	E	9q31	1	103000000	5647	nofs	nofs
chr09	E	9q31	1	103000000	2801	nofs	nofs
chr09	E	9q31	1	103000000	2392	nofs	nofs
chr09	E	9q31	1	104000000	2288	nofs	nofs
chr09	E	9q31	1	104000000	3082	nofs	nofs
chr09	E	9q31	1	104000000	4034	nofs	nofs
chr09	E	9q31	1	104000000	3457	nofs	nofs
chr09	E	9q31	1	105000000	1796	nofs	nofs
chr09	E	9q31	1	105000000	3251	nofs	nofs
chr09	E	9q31	1	105000000	2513	nofs	nofs
chr09	E	9q31	1	105000000	1448	nofs	nofs
chr09	E	9q31	1	106000000	20470	nofs	nofs
chr09	E	9q31	1	106000000	2159	nofs	nofs
chr09	E	9q31	1	106000000	2702	nofs	nofs
chr09	E	9q31	1	106000000	2212	nofs	nofs
chr09	E	9q31	1	107000000	2182	nofs	nofs
chr09	E	9q31	1	107000000	3733	nofs	nofs
chr09	E	9q31	1	107000000	4964	nofs	nofs
chr09	E	9q31	1	107000000	3224	nofs	nofs
chr09	E	9q31	1	108000000	6330	nofs	nofs
chr09	E	9q31	1	108000000	2359	nofs	nofs
chr09	E	9q31	1	108000000	2645	nofs	nofs
chr09	E	9q31	1	108000000	2715	nofs	nofs
chr09	E	9q31	1	109000000	1952	nofs	nofs
chr09	E	9q31	1	109000000	1941	nofs	nofs
chr09	E	9q31	1	109000000	1745	nofs	nofs
chr09	E	9q31	1	109000000	2273	nofs	nofs
chr09	E	9q31	1	110000000	2792	nofs	nofs
chr09	E	9q31	1	110000000	4134	nofs	nofs
chr09	E	9q31	1	110000000	3941	nofs	nofs

chr09	E	9q31	1	110000000	2363	nofs	nofs
chr09	E	9q31	1	111000000	4572	nofs	nofs
chr09	E	9q31	1	111000000	2934	nofs	nofs
chr09	E	9q31	1	111000000	3587	nofs	nofs
chr09	E	9q31	1	111000000	5250	nofs	nofs
chr09	E	9q31	1	112000000	3043	nofs	nofs
chr09	E	9q31	1	112000000	3404	nofs	nofs
chr09	E	9q32	1	112000000	3638	fs	fs common and rare
chr09	E	9q32	1	112000000	3880	fs	fs common and rare
chr09	E	9q32	1	113000000	4335	fs	fs common and rare
chr09	E	9q32	1	113000000	3980	fs	fs common and rare
chr09	E	9q32	1	113000000	2215	fs	fs common and rare
chr09	E	9q32	1	113000000	3122	fs	fs common and rare
chr09	E	9q32	1	114000000	6161	fs	fs common and rare
chr09	E	9q32	1	114000000	2726	fs	fs common and rare
chr09	E	9q32	1	114000000	3800	fs	fs common and rare
chr09	E	9q32	1	114000000	2886	fs	fs common and rare
chr09	E	9q32	1	115000000	2620	fs	fs common and rare
chr09	E	9q32	1	115000000	2985	fs	fs common and rare
chr09	E	9q33	4	115000000	3369	nofs	nofs
chr09	E	9q33	4	115000000	3401	nofs	nofs
chr09	E	9q33	4	116000000	2420	nofs	nofs
chr09	E	9q33	4	116000000	2369	nofs	nofs
chr09	E	9q33	4	116000000	2153	nofs	nofs
chr09	E	9q33	4	116000000	3057	nofs	nofs
chr09	E	9q33	4	117000000	3517	nofs	nofs
chr09	E	9q33	4	117000000	2882	nofs	nofs
chr09	E	9q33	4	117000000	2406	nofs	nofs
chr09	E	9q33	4	117000000	2361	nofs	nofs
chr09	E	9q33	4	118000000	2275	nofs	nofs
chr09	E	9q33	4	118000000	2199	nofs	nofs
chr09	E	9q33	4	118000000	2813	nofs	nofs
chr09	E	9q33	4	118000000	2238	nofs	nofs
chr09	E	9q33	4	119000000	3334	nofs	nofs
chr09	E	9q33	4	119000000	2919	nofs	nofs
chr09	E	9q33	4	119000000	2062	nofs	nofs

chr09	E	9q33	4	119000000	3299	nofs	nofs
chr09	E	9q33	4	120000000	2031	nofs	nofs
chr09	E	9q33	4	120000000	2027	nofs	nofs
chr09	E	9q33	4	120000000	3270	nofs	nofs
chr09	E	9q33	4	120000000	3839	nofs	nofs
chr09	E	9q33	4	121000000	3028	nofs	nofs
chr09	E	9q33	4	121000000	2531	nofs	nofs
chr09	E	9q33	4	121000000	1715	nofs	nofs
chr09	E	9q33	4	121000000	3104	nofs	nofs
chr09	E	9q33	4	122000000	4073	nofs	nofs
chr09	E	9q33	4	122000000	2602	nofs	nofs
chr09	E	9q33	4	122000000	2731	nofs	nofs
chr09	E	9q33	4	122000000	2384	nofs	nofs
chr09	E	9q33	4	123000000	4716	nofs	nofs
chr09	E	9q33	4	123000000	2733	nofs	nofs
chr09	E	9q33	4	123000000	5561	nofs	nofs
chr09	E	9q33	4	123000000	2708	nofs	nofs
chr09	E	9q33	4	124000000	1558	nofs	nofs
chr09	E	9q33	4	124000000	6486	nofs	nofs
chr09	E	9q33	4	124000000	1840	nofs	nofs
chr09	E	9q33	4	124000000	3483	nofs	nofs
chr09	E	9q33	4	125000000	5112	nofs	nofs
chr09	E	9q33	4	125000000	4310	nofs	nofs
chr09	E	9q33	4	125000000	3019	nofs	nofs
chr09	E	9q33	4	125000000	4887	nofs	nofs
chr09	E	9q33	4	126000000	3900	nofs	nofs
chr09	E	9q33	4	126000000	4235	nofs	nofs
chr09	E	9q33	4	126000000	3272	nofs	nofs
chr09	E	9q33	4	126000000	3524	nofs	nofs
chr09	E	9q33	4	127000000	5121	nofs	nofs
chr09	E	9q33	4	127000000	1564	nofs	nofs
chr09	E	9q33	4	127000000	1872	nofs	nofs
chr09	E	9q33	4	127000000	6030	nofs	nofs
chr09	E	9q34.1	3	128000000	4750	nofs	nofs
chr09	E	9q34.1	3	128000000	5963	nofs	nofs
chr09	E	9q34.1	3	128000000	5602	nofs	nofs

chr09	E	9q34.1	3	128000000	6157	nofs	nofs
chr09	E	9q34.1	3	129000000	8248	nofs	nofs
chr09	E	9q34.1	3	129000000	7938	nofs	nofs
chr09	E	9q34.1	3	129000000	9004	nofs	nofs
chr09	E	9q34.1	3	129000000	7687	nofs	nofs
chr09	E	9q34.1	3	130000000	4602	nofs	nofs
chr09	E	9q34.1	3	130000000	2941	nofs	nofs
chr09	E	9q34.1	3	130000000	3257	nofs	nofs
chr09	E	9q34.1	3	130000000	6018	nofs	nofs
chr09	E	9q34.1	3	131000000	5304	nofs	nofs
chr09	E	9q34.1	3	131000000	7910	nofs	nofs
chr09	E	9q34.1	3	131000000	3991	nofs	nofs
chr09	E	9q34.1	3	131000000	7638	nofs	nofs
chr09	E	9q34.1	3	132000000	2641	nofs	nofs
chr09	E	9q34.1	3	132000000	3634	nofs	nofs
chr09	E	9q34.1	3	132000000	4863	nofs	nofs
chr09	E	9q34.1	3	132000000	6255	nofs	nofs
chr09	E	9q34.1	3	133000000	4061	nofs	nofs
chr09	E	9q34.1	3	133000000	4031	nofs	nofs
chr09	E	9q34.1	3	133000000	7693	nofs	nofs
chr09	B	9q34.2	.	133000000	8502	nofs	nofs
chr09	B	9q34.2	.	134000000	9795	nofs	nofs
chr09	B	9q34.2	.	134000000	14278	nofs	nofs
chr09	B	9q34.2	.	134000000	4364	nofs	nofs
chr09	B	9q34.2	.	134000000	7762	nofs	nofs
chr09	E	9q34.3	1	135000000	18610	nofs	nofs
chr09	E	9q34.3	1	135000000	21200	nofs	nofs
chr09	T	9q34.3	1	135000000	14898	nofs	nofs
chr09	T	9q34.3	1	135000000	15447	nofs	nofs
chr09	T	9q34.3	1	136000000	12999	nofs	nofs
chr09	T	9q34.3	1	136000000	13204	nofs	nofs
chr09	T	9q34.3	1	136000000	8524	nofs	nofs
chr09	T	9q34.3	1	136000000	7357	nofs	nofs
chr09	T	9q34.3	1	137000000	8704	nofs	nofs
chr09	T	9q34.3	1	137000000	18333	nofs	nofs
chr09	T	9q34.3	1	137000000	9148	nofs	nofs

chr09	T	9q34.3	1	137000000	14520	nofs	nofs
chr09	T	9q34.3	1	138000000	34092	nofs	nofs
chr09	T	9q34.3	1	138000000	17202	nofs	nofs
chr09	T	9q34.3	1	138000000	16993	nofs	nofs
chr10	T	10p15	2	125000	4689	nofs	nofs
chr10	T	10p15	2	375000	28467	nofs	nofs
chr10	T	10p15	2	625000	31756	nofs	nofs
chr10	T	10p15	2	875000	21254	nofs	nofs
chr10	T	10p15	2	1125000	18044	nofs	nofs
chr10	T	10p15	2	1375000	22386	nofs	nofs
chr10	T	10p15	2	1625000	25883	nofs	nofs
chr10	T	10p15	2	1875000	7330	nofs	nofs
chr10	T	10p15	2	2125000	9367	nofs	nofs
chr10	T	10p15	2	2375000	9521	nofs	nofs
chr10	T	10p15	2	2625000	11442	nofs	nofs
chr10	T	10p15	2	2875000	7258	nofs	nofs
chr10	T	10p15	2	3125000	10126	nofs	nofs
chr10	E	10p15	2	3375000	15518	nofs	nofs
chr10	E	10p15	2	3625000	7318	nofs	nofs
chr10	E	10p15	2	3875000	4510	nofs	nofs
chr10	E	10p15	2	4125000	4706	nofs	nofs
chr10	E	10p15	2	4375000	4593	nofs	nofs
chr10	E	10p15	2	4625000	2653	nofs	nofs
chr10	E	10p15	2	4875000	3680	nofs	nofs
chr10	E	10p15	2	5125000	2151	nofs	nofs
chr10	E	10p15	2	5375000	5343	nofs	nofs
chr10	E	10p15	2	5625000	8666	nofs	nofs
chr10	E	10p15	2	5875000	3187	nofs	nofs
chr10	E	10p15	2	6125000	4703	nofs	nofs
chr10	E	10p15	2	6375000	6915	nofs	nofs
chr10	E	10p15	2	6625000	4103	nofs	nofs
chr10	E	10p14	2	6875000	6769	nofs	nofs
chr10	E	10p14	2	7125000	4869	nofs	nofs
chr10	E	10p14	2	7375000	4957	nofs	nofs
chr10	E	10p14	2	7625000	7234	nofs	nofs
chr10	E	10p14	2	7875000	4961	nofs	nofs

chr10	E	10p14	2	8125000	5696	nofs	nofs
chr10	E	10p14	2	8375000	7259	nofs	nofs
chr10	E	10p14	2	8625000	3704	nofs	nofs
chr10	E	10p14	2	8875000	3561	nofs	nofs
chr10	E	10p14	2	9125000	3571	nofs	nofs
chr10	E	10p14	2	9375000	5571	nofs	nofs
chr10	E	10p14	2	9625000	3118	nofs	nofs
chr10	E	10p14	2	9875000	3263	nofs	nofs
chr10	E	10p14	2	10125000	2415	nofs	nofs
chr10	E	10p14	2	10375000	4864	nofs	nofs
chr10	E	10p14	2	10625000	2391	nofs	nofs
chr10	E	10p14	2	10875000	2657	nofs	nofs
chr10	E	10p14	2	11125000	2704	nofs	nofs
chr10	E	10p14	2	11375000	5386	nofs	nofs
chr10	E	10p14	2	11625000	2725	nofs	nofs
chr10	E	10p14	2	11875000	4481	nofs	nofs
chr10	E	10p14	2	12125000	3835	nofs	nofs
chr10	E	10p14	2	12375000	6424	nofs	nofs
chr10	E	10p13	3	12625000	6223	nofs	nofs
chr10	E	10p13	3	12875000	5000	nofs	nofs
chr10	E	10p13	3	13125000	3291	nofs	nofs
chr10	E	10p13	3	13375000	5423	nofs	nofs
chr10	E	10p13	3	13625000	4575	nofs	nofs
chr10	E	10p13	3	13875000	4193	nofs	nofs
chr10	E	10p13	3	14125000	5004	nofs	nofs
chr10	E	10p13	3	14375000	2457	nofs	nofs
chr10	E	10p13	3	14625000	2446	nofs	nofs
chr10	E	10p13	3	14875000	3704	nofs	nofs
chr10	E	10p13	3	15125000	7449	nofs	nofs
chr10	E	10p13	3	15375000	4199	nofs	nofs
chr10	E	10p13	3	15625000	2856	nofs	nofs
chr10	E	10p13	3	15875000	3246	nofs	nofs
chr10	E	10p13	3	16125000	1761	nofs	nofs
chr10	E	10p13	3	16375000	3002	nofs	nofs
chr10	E	10p13	3	16625000	2714	nofs	nofs
chr10	E	10p13	3	16875000	3841	nofs	nofs

chr10	E	10p13	3	17125000	2913	nofs	nofs
chr10	E	10p13	3	17375000	4912	nofs	nofs
chr10	E	10p12.3	2	17625000	4447	nofs	nofs
chr10	E	10p12.3	2	17875000	2862	nofs	nofs
chr10	E	10p12.3	2	18125000	3189	nofs	nofs
chr10	E	10p12.3	2	18375000	3711	nofs	nofs
chr10	E	10p12.3	2	18625000	2635	nofs	nofs
chr10	E	10p12.3	2	18875000	22924	nofs	nofs
chr10	E	10p12.3	2	19125000	4127	nofs	nofs
chr10	E	10p12.3	2	19375000	2684	nofs	nofs
chr10	E	10p12.3	2	19625000	6443	nofs	nofs
chr10	E	10p12.3	2	19875000	3083	nofs	nofs
chr10	E	10p12.3	2	20125000	4125	nofs	nofs
chr10	E	10p12.3	2	20375000	4431	nofs	nofs
chr10	E	10p12.3	2	20625000	2115	nofs	nofs
chr10	E	10p12.3	2	20875000	2562	nofs	nofs
chr10	E	10p12.3	2	21125000	1371	nofs	nofs
chr10	E	10p12.3	2	21375000	2031	nofs	nofs
chr10	E	10p12.3	2	21625000	5549	nofs	nofs
chr10	E	10p12.3	2	21875000	3406	nofs	nofs
chr10	E	10p12.3	2	22125000	4000	nofs	nofs
chr10	E	10p12.3	2	22375000	1734	nofs	nofs
chr10	E	10p12.3	2	22625000	2465	nofs	nofs
chr10	B	10p12.2	.	22875000	2001	nofs	nofs
chr10	B	10p12.2	.	23125000	1915	nofs	nofs
chr10	B	10p12.2	.	23375000	4429	nofs	nofs
chr10	B	10p12.2	.	23625000	4382	nofs	nofs
chr10	B	10p12.2	.	23875000	2673	nofs	nofs
chr10	B	10p12.2	.	24125000	3576	nofs	nofs
chr10	B	10p12.2	.	24375000	1966	nofs	nofs
chr10	E	10p12.1	4	24625000	3413	nofs	nofs
chr10	E	10p12.1	4	24875000	4431	nofs	nofs
chr10	E	10p12.1	4	25125000	3496	nofs	nofs
chr10	E	10p12.1	4	25375000	3280	nofs	nofs
chr10	E	10p12.1	4	25625000	2072	nofs	nofs
chr10	E	10p12.1	4	25875000	1769	nofs	nofs

chr10	E	10p12.1	4	26125000	4434	nofs	nofs
chr10	E	10p12.1	4	26375000	2335	nofs	nofs
chr10	E	10p12.1	4	26625000	3105	nofs	nofs
chr10	E	10p12.1	4	26875000	3425	nofs	nofs
chr10	E	10p12.1	4	27125000	3760	nofs	nofs
chr10	E	10p12.1	4	27375000	3159	nofs	nofs
chr10	E	10p12.1	4	27625000	4880	nofs	nofs
chr10	E	10p12.1	4	27875000	4268	nofs	nofs
chr10	E	10p12.1	4	28125000	4118	nofs	nofs
chr10	E	10p12.1	4	28375000	3086	nofs	nofs
chr10	E	10p12.1	4	28625000	3985	nofs	nofs
chr10	E	10p12.1	4	28875000	3247	nofs	nofs
chr10	E	10p12.1	4	29125000	5896	nofs	nofs
chr10	E	10p12.1	4	29375000	2995	nofs	nofs
chr10	E	10p11.2	5	29625000	3989	nofs	nofs
chr10	E	10p11.2	5	29875000	3534	nofs	nofs
chr10	E	10p11.2	5	30125000	4643	nofs	nofs
chr10	E	10p11.2	5	30375000	5028	nofs	nofs
chr10	E	10p11.2	5	30625000	3348	nofs	nofs
chr10	E	10p11.2	5	30875000	3918	nofs	nofs
chr10	E	10p11.2	5	31125000	5876	nofs	nofs
chr10	E	10p11.2	5	31375000	2640	nofs	nofs
chr10	E	10p11.2	5	31625000	2481	nofs	nofs
chr10	E	10p11.2	5	31875000	2308	nofs	nofs
chr10	E	10p11.2	5	32125000	2219	nofs	nofs
chr10	E	10p11.2	5	32375000	4873	nofs	nofs
chr10	E	10p11.2	5	32625000	4216	nofs	nofs
chr10	E	10p11.2	5	32875000	3241	nofs	nofs
chr10	E	10p11.2	5	33125000	2709	nofs	nofs
chr10	E	10p11.2	5	33375000	3800	nofs	nofs
chr10	E	10p11.2	5	33625000	2197	nofs	nofs
chr10	E	10p11.2	5	33875000	3619	nofs	nofs
chr10	E	10p11.2	5	34125000	3411	nofs	nofs
chr10	E	10p11.2	5	34375000	4138	nofs	nofs
chr10	E	10p11.2	5	34625000	2598	nofs	nofs
chr10	E	10p11.2	5	34875000	3198	nofs	nofs

chr10	E	10p11.2	5	35125000	3979	nofs	nofs
chr10	E	10p11.2	5	35375000	3263	nofs	nofs
chr10	E	10p11.2	5	35625000	5189	nofs	nofs
chr10	E	10p11.2	5	35875000	6336	nofs	nofs
chr10	E	10p11.2	5	36125000	3255	nofs	nofs
chr10	C	10p11.2	5	36375000	1598	nofs	nofs
chr10	C	10p11.2	5	36625000	3056	nofs	nofs
chr10	C	10p11.2	5	36875000	2911	nofs	nofs
chr10	C	10p11.2	5	37125000	4693	nofs	nofs
chr10	C	10p11.2	5	37375000	2971	nofs	nofs
chr10	C	10p11.2	5	37625000	2458	nofs	nofs
chr10	C	10p11.2	5	37875000	2537	nofs	nofs
chr10	C	10p11.2	5	38125000	3507	nofs	nofs
chr10	C	10p11.2	5	38375000	4723	nofs	nofs
chr10	C	10p11.2	5	38375000	4723	nofs	nofs
chr10	C	10p11.2	5	38625000	3059	nofs	nofs
chr10	C	10p11.2	5	38875000	69385	nofs	nofs
chr10	C	10p11.2	5	39125000	81912	nofs	nofs
chr10	C		.	39375000	0	nofs	nofs
chr10	C		.	39625000	0	nofs	nofs
chr10	C		.	39875000	0	nofs	nofs
chr10	C		.	40125000	0	nofs	nofs
chr10	C		.	40375000	0	nofs	nofs
chr10	C		.	40625000	0	nofs	nofs
chr10	C		.	40875000	0	nofs	nofs
chr10	C		.	41125000	0	nofs	nofs
chr10	C		.	41375000	0	nofs	nofs
chr10	C	10q11.2	6	41625000	73304	fs	fs common
chr10	C	10q11.2	6	41875000	109487	fs	fs common
chr10	C	10q11.2	6	42125000	35915	fs	fs common
chr10	C	10q11.2	6	42375000	3185	fs	fs common
chr10	C	10q11.2	6	42625000	3990	fs	fs common
chr10	C	10q11.2	6	42875000	3424	fs	fs common
chr10	C	10q11.2	6	43125000	5337	fs	fs common
chr10	C	10q11.2	6	43375000	7245	fs	fs common
chr10	C	10q11.2	6	43625000	4054	fs	fs common

chr10	C	10q11.2	6	43875000	2007	fs	fs common
chr10	C	10q11.2	6	44125000	2700	fs	fs common
chr10	C	10q11.2	6	44375000	1666	fs	fs common
chr10	C	10q11.2	6	44625000	4386	fs	fs common
chr10	E	10q11.2	6	44875000	4510	fs	fs common
chr10	E	10q11.2	6	45125000	5564	fs	fs common
chr10	E	10q11.2	6	45375000	2849	fs	fs common
chr10	E	10q11.2	6	45625000	6028	fs	fs common
chr10	E	10q11.2	6	45875000	1648	fs	fs common
chr10	E	10q11.2	6	46125000	3269	fs	fs common
chr10	E	10q11.2	6	46375000	4138	fs	fs common
chr10	E	10q11.2	6	46625000	3790	fs	fs common
chr10	E	10q11.2	6	46875000	758	fs	fs common
chr10	E	10q11.2	6	47125000	7027	fs	fs common
chr10	E	10q11.2	6	47375000	775	fs	fs common
chr10	E	10q11.2	6	47625000	1004	fs	fs common
chr10	E	10q11.2	6	47875000	7238	fs	fs common
chr10	E	10q11.2	6	48125000	5013	fs	fs common
chr10	E	10q11.2	6	48375000	2776	fs	fs common
chr10	E	10q11.2	6	48625000	3077	fs	fs common
chr10	E	10q11.2	6	48875000	2117	fs	fs common
chr10	E	10q11.2	6	49125000	1864	fs	fs common
chr10	E	10q11.2	6	49375000	1769	fs	fs common
chr10	E	10q11.2	6	49625000	5634	fs	fs common
chr10	E	10q11.2	6	49875000	3603	fs	fs common
chr10	E	10q11.2	6	50125000	3014	fs	fs common
chr10	E	10q11.2	6	50375000	2753	fs	fs common
chr10	E	10q11.2	6	50625000	4207	fs	fs common
chr10	E	10q11.2	6	50875000	4790	fs	fs common
chr10	E	10q11.2	6	51125000	2919	fs	fs common
chr10	E	10q11.2	6	51375000	5086	fs	fs common
chr10	E	10q11.2	6	51625000	2174	fs	fs common
chr10	E	10q11.2	6	51875000	2422	fs	fs common
chr10	E	10q11.2	6	52125000	1403	fs	fs common
chr10	E	10q11.2	6	52375000	2645	fs	fs common
chr10	E	10q11.2	6	52625000	2624	fs	fs common

chr10	E	10q21.1	4	52875000	2861	fs	fs common
chr10	E	10q21.1	4	53125000	2819	fs	fs common
chr10	E	10q21.1	4	53375000	2785	fs	fs common
chr10	E	10q21.1	4	53625000	3155	fs	fs common
chr10	E	10q21.1	4	53875000	2225	fs	fs common
chr10	E	10q21.1	4	54125000	2065	fs	fs common
chr10	E	10q21.1	4	54375000	2643	fs	fs common
chr10	E	10q21.1	4	54625000	1372	fs	fs common
chr10	E	10q21.1	4	54875000	3238	fs	fs common
chr10	E	10q21.1	4	55125000	2362	fs	fs common
chr10	E	10q21.1	4	55375000	3290	fs	fs common
chr10	E	10q21.1	4	55625000	3061	fs	fs common
chr10	E	10q21.1	4	55875000	2903	fs	fs common
chr10	E	10q21.1	4	56125000	2960	fs	fs common
chr10	E	10q21.1	4	56375000	4419	fs	fs common
chr10	E	10q21.1	4	56625000	1763	fs	fs common
chr10	E	10q21.1	4	56875000	4839	fs	fs common
chr10	E	10q21.1	4	57125000	1740	fs	fs common
chr10	E	10q21.1	4	57375000	2555	fs	fs common
chr10	E	10q21.1	4	57625000	3273	fs	fs common
chr10	E	10q21.1	4	57875000	2297	fs	fs common
chr10	E	10q21.1	4	58125000	2252	fs	fs common
chr10	E	10q21.1	4	58375000	1944	fs	fs common
chr10	E	10q21.1	4	58625000	2260	fs	fs common
chr10	E	10q21.1	4	58875000	4966	fs	fs common
chr10	E	10q21.1	4	59125000	3681	fs	fs common
chr10	E	10q21.1	4	59375000	2349	fs	fs common
chr10	E	10q21.1	4	59625000	3408	fs	fs common
chr10	E	10q21.1	4	59875000	2822	fs	fs common
chr10	E	10q21.1	4	60125000	2547	fs	fs common
chr10	E	10q21.1	4	60375000	1648	fs	fs common
chr10	E	10q21.1	4	60625000	2673	fs	fs common
chr10	E	10q21.1	4	60875000	2069	fs	fs common
chr10	E	10q21.2	2	61125000	3450	fs	fs common
chr10	E	10q21.2	2	61375000	2489	fs	fs common
chr10	E	10q21.2	2	61625000	2267	fs	fs common

chr10	E	10q21.2	2	61875000	2274	fs	fs common
chr10	E	10q21.2	2	62125000	1867	fs	fs common
chr10	E	10q21.2	2	62375000	1967	fs	fs common
chr10	E	10q21.2	2	62625000	1616	fs	fs common
chr10	E	10q21.2	2	62875000	3171	fs	fs common
chr10	E	10q21.2	2	63125000	2453	fs	fs common
chr10	E	10q21.2	2	63375000	2123	fs	fs common
chr10	E	10q21.2	2	63625000	2500	fs	fs common
chr10	E	10q21.2	2	63875000	3026	fs	fs common
chr10	E	10q21.2	2	64125000	2031	fs	fs common
chr10	E	10q21.3	2	64375000	4395	fs	fs common
chr10	E	10q21.3	2	64625000	2508	fs	fs common
chr10	E	10q21.3	2	64875000	2695	fs	fs common
chr10	E	10q21.3	2	65125000	2860	fs	fs common
chr10	E	10q21.3	2	65375000	1272	fs	fs common
chr10	E	10q21.3	2	65625000	2298	fs	fs common
chr10	E	10q21.3	2	65875000	3342	fs	fs common
chr10	E	10q21.3	2	66125000	2328	fs	fs common
chr10	E	10q21.3	2	66375000	2244	fs	fs common
chr10	E	10q21.3	2	66625000	3251	fs	fs common
chr10	E	10q21.3	2	66875000	2312	fs	fs common
chr10	E	10q21.3	2	67125000	2141	fs	fs common
chr10	E	10q21.3	2	67375000	1728	fs	fs common
chr10	E	10q21.3	2	67625000	2189	fs	fs common
chr10	E	10q21.3	2	67875000	2364	fs	fs common
chr10	E	10q21.3	2	68125000	2133	fs	fs common
chr10	E	10q21.3	2	68375000	1696	fs	fs common
chr10	E	10q21.3	2	68625000	1406	fs	fs common
chr10	E	10q21.3	2	68875000	2169	fs	fs common
chr10	E	10q21.3	2	69125000	5556	fs	fs common
chr10	E	10q21.3	2	69375000	3658	fs	fs common
chr10	E	10q21.3	2	69625000	3021	fs	fs common
chr10	E	10q21.3	2	69875000	4988	fs	fs common
chr10	E	10q21.3	2	70125000	5920	fs	fs common
chr10	E	10q21.3	2	70375000	6012	fs	fs common
chr10	E	10q22.1	2	70625000	2416	fs	fs common

chr10	E	10q22.1	2	70875000	2798	fs	fs common
chr10	E	10q22.1	2	71125000	2707	fs	fs common
chr10	E	10q22.1	2	71375000	4141	fs	fs common
chr10	E	10q22.1	2	71625000	4749	fs	fs common
chr10	E	10q22.1	2	71875000	4526	fs	fs common
chr10	E	10q22.1	2	72125000	6069	fs	fs common
chr10	E	10q22.1	2	72375000	4632	fs	fs common
chr10	E	10q22.1	2	72625000	2175	fs	fs common
chr10	E	10q22.1	2	72875000	2409	fs	fs common
chr10	E	10q22.1	2	73125000	3605	fs	fs common
chr10	E	10q22.1	2	73375000	4042	fs	fs common
chr10	E	10q22.1	2	73625000	4494	fs	fs common
chr10	E	10q22.1	2	73875000	2854	fs	fs common
chr10	E	10q22.1	2	74125000	3786	fs	fs common
chr10	E	10q22.1	2	74375000	2431	fs	fs common
chr10	E	10q22.1	2	74625000	2800	fs	fs common
chr10	E	10q22.2	3	74875000	2552	nofs	nofs
chr10	E	10q22.2	3	75125000	5117	nofs	nofs
chr10	E	10q22.2	3	75375000	2810	nofs	nofs
chr10	E	10q22.2	3	75625000	2319	nofs	nofs
chr10	E	10q22.2	3	75875000	1868	nofs	nofs
chr10	E	10q22.2	3	76125000	2302	nofs	nofs
chr10	E	10q22.2	3	76375000	1983	nofs	nofs
chr10	E	10q22.2	3	76625000	2846	nofs	nofs
chr10	E	10q22.2	3	76875000	4531	nofs	nofs
chr10	E	10q22.2	3	77125000	3068	nofs	nofs
chr10	E	10q22.2	3	77375000	2785	nofs	nofs
chr10	E	10q22.2	3	77625000	2380	nofs	nofs
chr10	E	10q22.3	3	77875000	1629	nofs	nofs
chr10	E	10q22.3	3	78125000	4536	nofs	nofs
chr10	E	10q22.3	3	78375000	2716	nofs	nofs
chr10	E	10q22.3	3	78625000	2143	nofs	nofs
chr10	E	10q22.3	3	78875000	3986	nofs	nofs
chr10	E	10q22.3	3	79125000	3669	nofs	nofs
chr10	E	10q22.3	3	79375000	2009	nofs	nofs
chr10	E	10q22.3	3	79625000	3294	nofs	nofs

chr10	E	10q22.3	3	79875000	2775	nofs	nofs
chr10	E	10q22.3	3	80125000	2477	nofs	nofs
chr10	E	10q22.3	3	80375000	2098	nofs	nofs
chr10	E	10q22.3	3	80625000	2380	nofs	nofs
chr10	E	10q22.3	3	80875000	6912	nofs	nofs
chr10	E	10q22.3	3	81125000	3872	nofs	nofs
chr10	E	10q22.3	3	81375000	5048	nofs	nofs
chr10	E	10q22.3	3	81625000	2450	nofs	nofs
chr10	E	10q22.3	3	81875000	3012	nofs	nofs
chr10	E	10q22.3	3	82125000	2708	nofs	nofs
chr10	E	10q22.3	3	82375000	5977	nofs	nofs
chr10	E	10q22.3	3	82625000	1779	nofs	nofs
chr10	E	10q22.3	3	82875000	1650	nofs	nofs
chr10	E	10q23.1	1	83125000	5057	nofs	nofs
chr10	E	10q23.1	1	83375000	3809	nofs	nofs
chr10	E	10q23.1	1	83625000	2231	nofs	nofs
chr10	E	10q23.1	1	83875000	2002	nofs	nofs
chr10	E	10q23.1	1	84125000	2689	nofs	nofs
chr10	E	10q23.1	1	84375000	2982	nofs	nofs
chr10	E	10q23.1	1	84625000	2608	nofs	nofs
chr10	E	10q23.1	1	84875000	1530	nofs	nofs
chr10	E	10q23.1	1	85125000	3075	nofs	nofs
chr10	E	10q23.1	1	85375000	1633	nofs	nofs
chr10	E	10q23.1	1	85625000	1947	nofs	nofs
chr10	E	10q23.1	1	85875000	3371	nofs	nofs
chr10	E	10q23.1	1	86125000	4065	nofs	nofs
chr10	E	10q23.1	1	86375000	4389	nofs	nofs
chr10	E	10q23.1	1	86625000	1938	nofs	nofs
chr10	E	10q23.2	4	86875000	2072	nofs	nofs
chr10	E	10q23.2	4	87125000	2414	nofs	nofs
chr10	E	10q23.2	4	87375000	2423	nofs	nofs
chr10	E	10q23.2	4	87625000	3272	nofs	nofs
chr10	E	10q23.2	4	87875000	4692	nofs	nofs
chr10	E	10q23.2	4	88125000	4016	nofs	nofs
chr10	E	10q23.2	4	88375000	3673	nofs	nofs
chr10	E	10q23.2	4	88625000	2854	nofs	nofs

chr10	E	10q23.2	4	88875000	5111	nofs	nofs
chr10	E	10q23.3	4	89125000	2440	fs	fs rare
chr10	E	10q23.3	4	89375000	2536	fs	fs rare
chr10	E	10q23.3	4	89625000	1994	fs	fs rare
chr10	E	10q23.3	4	89875000	2599	fs	fs rare
chr10	E	10q23.3	4	90125000	1379	fs	fs rare
chr10	E	10q23.3	4	90375000	2741	fs	fs rare
chr10	E	10q23.3	4	90625000	2180	fs	fs rare
chr10	E	10q23.3	4	90875000	3287	fs	fs rare
chr10	E	10q23.3	4	91125000	3080	fs	fs rare
chr10	E	10q23.3	4	91375000	3251	fs	fs rare
chr10	E	10q23.3	4	91625000	3762	fs	fs rare
chr10	E	10q23.3	4	91875000	2016	fs	fs rare
chr10	E	10q23.3	4	92125000	1895	fs	fs rare
chr10	E	10q23.3	4	92375000	1548	fs	fs rare
chr10	E	10q23.3	4	92625000	2644	fs	fs rare
chr10	E	10q23.3	4	92875000	2752	fs	fs rare
chr10	E	10q23.3	4	93125000	2913	fs	fs rare
chr10	E	10q23.3	4	93375000	1671	fs	fs rare
chr10	E	10q23.3	4	93625000	1996	fs	fs rare
chr10	E	10q23.3	4	93875000	4934	fs	fs rare
chr10	E	10q23.3	4	94125000	6631	fs	fs rare
chr10	E	10q23.3	4	94375000	6630	fs	fs rare
chr10	E	10q23.3	4	94625000	4299	fs	fs rare
chr10	E	10q23.3	4	94875000	2884	fs	fs rare
chr10	E	10q23.3	4	95125000	4189	fs	fs rare
chr10	E	10q23.3	4	95375000	2032	fs	fs rare
chr10	E	10q23.3	4	95625000	3207	fs	fs rare
chr10	E	10q23.3	4	95875000	2221	fs	fs rare
chr10	E	10q23.3	4	96125000	2392	fs	fs rare
chr10	E	10q23.3	4	96375000	1878	fs	fs rare
chr10	E	10q23.3	4	96625000	2003	fs	fs rare
chr10	E	10q23.3	4	96875000	1068	fs	fs rare
chr10	E	10q23.3	4	97125000	1258	fs	fs rare
chr10	E	10q23.3	4	97375000	2990	fs	fs rare
chr10	E	10q24.1	2	97625000	3557	nofs	nofs

chr10	E	10q24.1	2	97875000	2317	nofs	nofs
chr10	E	10q24.1	2	98125000	2308	nofs	nofs
chr10	E	10q24.1	2	98375000	2249	nofs	nofs
chr10	E	10q24.1	2	98625000	6691	nofs	nofs
chr10	E	10q24.1	2	98875000	3616	nofs	nofs
chr10	E	10q24.1	2	99125000	7019	nofs	nofs
chr10	E	10q24.1	2	99375000	4456	nofs	nofs
chr10	E	10q24.2	2	99625000	3382	nofs	nofs
chr10	E	10q24.2	2	99875000	3503	nofs	nofs
chr10	E	10q24.2	2	100000000	2874	nofs	nofs
chr10	E	10q24.2	2	100000000	1482	nofs	nofs
chr10	E	10q24.2	2	101000000	1558	nofs	nofs
chr10	E	10q24.2	2	101000000	2332	nofs	nofs
chr10	E	10q24.2	2	101000000	2083	nofs	nofs
chr10	E	10q24.2	2	101000000	2691	nofs	nofs
chr10	E	10q24.2	2	102000000	5311	nofs	nofs
chr10	E	10q24.2	2	102000000	3463	nofs	nofs
chr10	E	10q24.3	2	102000000	4638	nofs	nofs
chr10	E	10q24.3	2	102000000	3668	nofs	nofs
chr10	E	10q24.3	2	103000000	2611	nofs	nofs
chr10	E	10q24.3	2	103000000	3031	nofs	nofs
chr10	E	10q24.3	2	103000000	5031	nofs	nofs
chr10	E	10q24.3	2	103000000	2266	nofs	nofs
chr10	E	10q24.3	2	104000000	2418	nofs	nofs
chr10	E	10q24.3	2	104000000	5353	nofs	nofs
chr10	E	10q24.3	2	104000000	2890	nofs	nofs
chr10	E	10q24.3	2	104000000	4493	nofs	nofs
chr10	E	10q24.3	2	105000000	5424	nofs	nofs
chr10	E	10q24.3	2	105000000	3569	nofs	nofs
chr10	E	10q24.3	2	105000000	5063	nofs	nofs
chr10	E	10q24.3	2	105000000	3556	nofs	nofs
chr10	E	10q24.3	2	106000000	1995	nofs	nofs
chr10	E	10q25.1	1	106000000	2249	nofs	nofs
chr10	E	10q25.1	1	106000000	2289	nofs	nofs
chr10	E	10q25.1	1	106000000	3255	nofs	nofs
chr10	E	10q25.1	1	107000000	2313	nofs	nofs

chr10	E	10q25.1	1	107000000	3237	nofs	nofs
chr10	E	10q25.1	1	107000000	2584	nofs	nofs
chr10	E	10q25.1	1	107000000	3802	nofs	nofs
chr10	E	10q25.1	1	108000000	2376	nofs	nofs
chr10	E	10q25.1	1	108000000	3108	nofs	nofs
chr10	E	10q25.1	1	108000000	3131	nofs	nofs
chr10	E	10q25.1	1	108000000	2417	nofs	nofs
chr10	E	10q25.1	1	109000000	2263	nofs	nofs
chr10	E	10q25.1	1	109000000	1582	nofs	nofs
chr10	E	10q25.1	1	109000000	1660	nofs	nofs
chr10	E	10q25.1	1	109000000	1473	nofs	nofs
chr10	E	10q25.1	1	110000000	2677	nofs	nofs
chr10	E	10q25.1	1	110000000	1833	nofs	nofs
chr10	E	10q25.1	1	110000000	2892	nofs	nofs
chr10	E	10q25.1	1	110000000	4313	nofs	nofs
chr10	E	10q25.1	1	111000000	3304	nofs	nofs
chr10	E	10q25.1	1	111000000	3070	nofs	nofs
chr10	E	10q25.1	1	111000000	1847	nofs	nofs
chr10	E	10q25.1	1	111000000	1403	nofs	nofs
chr10	E	10q25.1	1	112000000	2300	nofs	nofs
chr10	E	10q25.1	1	112000000	2333	nofs	nofs
chr10	B	10q25.2	.	112000000	4807	fs	fs common and rare
chr10	B	10q25.2	.	112000000	1841	fs	fs common and rare
chr10	B	10q25.2	.	113000000	2886	fs	fs common and rare
chr10	B	10q25.2	.	113000000	2457	fs	fs common and rare
chr10	B	10q25.2	.	113000000	3189	fs	fs common and rare
chr10	B	10q25.2	.	113000000	2908	fs	fs common and rare
chr10	B	10q25.2	.	114000000	2276	fs	fs common and rare
chr10	B	10q25.2	.	114000000	1811	fs	fs common and rare
chr10	B	10q25.2	.	114000000	4651	fs	fs common and rare
chr10	B	10q25.2	.	114000000	2747	fs	fs common and rare
chr10	B	10q25.2	.	115000000	3906	fs	fs common and rare
chr10	B	10q25.2	.	115000000	2922	fs	fs common and rare
chr10	B	10q25.3	.	115000000	1771	nofs	nofs
chr10	B	10q25.3	.	115000000	2668	nofs	nofs
chr10	B	10q25.3	.	116000000	3531	nofs	nofs

chr10	B	10q25.3	.	116000000	2833	nofs	nofs
chr10	B	10q25.3	.	116000000	3345	nofs	nofs
chr10	B	10q25.3	.	116000000	3952	nofs	nofs
chr10	B	10q25.3	.	117000000	4691	nofs	nofs
chr10	B	10q25.3	.	117000000	2142	nofs	nofs
chr10	B	10q25.3	.	117000000	2031	nofs	nofs
chr10	B	10q25.3	.	117000000	2407	nofs	nofs
chr10	B	10q25.3	.	118000000	1623	nofs	nofs
chr10	B	10q25.3	.	118000000	2399	nofs	nofs
chr10	B	10q25.3	.	118000000	3213	nofs	nofs
chr10	B	10q25.3	.	118000000	4152	nofs	nofs
chr10	B	10q25.3	.	119000000	2210	nofs	nofs
chr10	E	10q26.1	3	119000000	3027	fs	fs common
chr10	E	10q26.1	3	119000000	3543	fs	fs common
chr10	E	10q26.1	3	119000000	2164	fs	fs common
chr10	E	10q26.1	3	120000000	3184	fs	fs common
chr10	E	10q26.1	3	120000000	3626	fs	fs common
chr10	E	10q26.1	3	120000000	3914	fs	fs common
chr10	E	10q26.1	3	120000000	2591	fs	fs common
chr10	E	10q26.1	3	121000000	4127	fs	fs common
chr10	E	10q26.1	3	121000000	5009	fs	fs common
chr10	E	10q26.1	3	121000000	7804	fs	fs common
chr10	E	10q26.1	3	121000000	6021	fs	fs common
chr10	E	10q26.1	3	122000000	3910	fs	fs common
chr10	E	10q26.1	3	122000000	2183	fs	fs common
chr10	E	10q26.1	3	122000000	2776	fs	fs common
chr10	E	10q26.1	3	122000000	2953	fs	fs common
chr10	E	10q26.1	3	123000000	3157	fs	fs common
chr10	E	10q26.1	3	123000000	3160	fs	fs common
chr10	E	10q26.1	3	123000000	3174	fs	fs common
chr10	E	10q26.1	3	123000000	3303	fs	fs common
chr10	E	10q26.1	3	124000000	3074	fs	fs common
chr10	E	10q26.1	3	124000000	3956	fs	fs common
chr10	E	10q26.1	3	124000000	1973	fs	fs common
chr10	E	10q26.1	3	124000000	5361	fs	fs common
chr10	E	10q26.1	3	125000000	2651	fs	fs common

chr10	E	10q26.1	3	125000000	2813	fs	fs common
chr10	E	10q26.1	3	125000000	3745	fs	fs common
chr10	E	10q26.1	3	125000000	3125	fs	fs common
chr10	E	10q26.1	3	126000000	5481	fs	fs common
chr10	E	10q26.1	3	126000000	4486	fs	fs common
chr10	E	10q26.1	3	126000000	5278	fs	fs common
chr10	E	10q26.1	3	126000000	3443	fs	fs common
chr10	E	10q26.1	3	127000000	2556	fs	fs common
chr10	E	10q26.1	3	127000000	2766	fs	fs common
chr10	E	10q26.1	3	127000000	2799	fs	fs common
chr10	E	10q26.1	3	127000000	1941	fs	fs common
chr10	E	10q26.2	1	128000000	2321	nofs	nofs
chr10	E	10q26.2	1	128000000	6255	nofs	nofs
chr10	E	10q26.2	1	128000000	3871	nofs	nofs
chr10	E	10q26.2	1	128000000	2249	nofs	nofs
chr10	E	10q26.2	1	129000000	4499	nofs	nofs
chr10	E	10q26.2	1	129000000	3082	nofs	nofs
chr10	E	10q26.2	1	129000000	5647	nofs	nofs
chr10	E	10q26.2	1	129000000	2690	nofs	nofs
chr10	E	10q26.2	1	130000000	3549	nofs	nofs
chr10	E	10q26.2	1	130000000	9212	nofs	nofs
chr10	E	10q26.2	1	130000000	4355	nofs	nofs
chr10	E	10q26.2	1	130000000	2740	nofs	nofs
chr10	E	10q26.2	1	131000000	5406	nofs	nofs
chr10	B	10q26.3	.	131000000	5552	nofs	nofs
chr10	B	10q26.3	.	131000000	4166	nofs	nofs
chr10	B	10q26.3	.	131000000	3455	nofs	nofs
chr10	B	10q26.3	.	132000000	5868	nofs	nofs
chr10	B	10q26.3	.	132000000	6697	nofs	nofs
chr10	T	10q26.3	.	132000000	8786	nofs	nofs
chr10	T	10q26.3	.	132000000	10953	nofs	nofs
chr10	T	10q26.3	.	133000000	11892	nofs	nofs
chr10	T	10q26.3	.	133000000	9507	nofs	nofs
chr10	T	10q26.3	.	133000000	10534	nofs	nofs
chr10	T	10q26.3	.	133000000	15867	nofs	nofs
chr10	T	10q26.3	.	134000000	16418	nofs	nofs

chr10	T	10q26.3	.	134000000	22582	nofs	nofs
chr10	T	10q26.3	.	134000000	26234	nofs	nofs
chr10	T	10q26.3	.	134000000	34476	nofs	nofs
chr10	T	10q26.3	.	135000000	30184	nofs	nofs
chr10	T	10q26.3	.	135000000	36370	nofs	nofs
chr10	T	10q26.3	.	135000000	21169	nofs	nofs
chr11	T	11p15.5	8	125000	8898	nofs	nofs
chr11	T	11p15.5	8	375000	31735	nofs	nofs
chr11	T	11p15.5	8	625000	18201	nofs	nofs
chr11	T	11p15.5	8	875000	13201	nofs	nofs
chr11	T	11p15.5	8	1125000	22310	nofs	nofs
chr11	T	11p15.5	8	1375000	26976	nofs	nofs
chr11	T	11p15.5	8	1625000	17691	nofs	nofs
chr11	T	11p15.5	8	1875000	17703	nofs	nofs
chr11	T	11p15.5	8	2125000	12937	nofs	nofs
chr11	T	11p15.5	8	2375000	9540	nofs	nofs
chr11	T	11p15.4	8	2625000	4019	nofs	nofs
chr11	T	11p15.4	8	2875000	5619	nofs	nofs
chr11	T	11p15.4	8	3125000	8957	nofs	nofs
chr11	E	11p15.4	8	3375000	8570	nofs	nofs
chr11	E	11p15.4	8	3625000	4199	nofs	nofs
chr11	E	11p15.4	8	3875000	2224	nofs	nofs
chr11	E	11p15.4	8	4125000	2716	nofs	nofs
chr11	E	11p15.4	8	4375000	2203	nofs	nofs
chr11	E	11p15.4	8	4625000	1461	nofs	nofs
chr11	E	11p15.4	8	4875000	2555	nofs	nofs
chr11	E	11p15.4	8	5125000	2901	nofs	nofs
chr11	E	11p15.4	8	5375000	2349	nofs	nofs
chr11	E	11p15.4	8	5625000	2925	nofs	nofs
chr11	E	11p15.4	8	5875000	2427	nofs	nofs
chr11	E	11p15.4	8	6125000	2125	nofs	nofs
chr11	E	11p15.4	8	6375000	2347	nofs	nofs
chr11	E	11p15.4	8	6625000	1732	nofs	nofs
chr11	E	11p15.4	8	6875000	1946	nofs	nofs
chr11	E	11p15.4	8	7125000	1951	nofs	nofs
chr11	E	11p15.4	8	7375000	1945	nofs	nofs

chr11	E	11p15.4	8	7625000	2296	nofs	nofs
chr11	E	11p15.4	8	7875000	1715	nofs	nofs
chr11	E	11p15.4	8	8125000	2782	nofs	nofs
chr11	E	11p15.4	8	8375000	1389	nofs	nofs
chr11	E	11p15.4	8	8625000	3327	nofs	nofs
chr11	E	11p15.4	8	8875000	4853	nofs	nofs
chr11	E	11p15.4	8	9125000	3070	nofs	nofs
chr11	E	11p15.4	8	9375000	8471	nofs	nofs
chr11	E	11p15.4	8	9625000	5743	nofs	nofs
chr11	E	11p15.4	8	9875000	3254	nofs	nofs
chr11	E	11p15.4	8	10125000	2515	nofs	nofs
chr11	E	11p15.4	8	10375000	2370	nofs	nofs
chr11	E	11p15.4	8	10625000	2915	nofs	nofs
chr11	B	11p15.3	.	10875000	3236	nofs	nofs
chr11	B	11p15.3	.	11125000	2250	nofs	nofs
chr11	B	11p15.3	.	11375000	1293	nofs	nofs
chr11	B	11p15.3	.	11625000	2668	nofs	nofs
chr11	B	11p15.3	.	11875000	2775	nofs	nofs
chr11	B	11p15.3	.	12125000	1644	nofs	nofs
chr11	B	11p15.3	.	12375000	1606	nofs	nofs
chr11	B	11p15.3	.	12625000	1810	nofs	nofs
chr11	E	11p15.2	1	12875000	1605	nofs	nofs
chr11	E	11p15.2	1	13125000	1901	nofs	nofs
chr11	E	11p15.2	1	13375000	2530	nofs	nofs
chr11	E	11p15.2	1	13625000	1566	nofs	nofs
chr11	E	11p15.2	1	13875000	1197	nofs	nofs
chr11	E	11p15.2	1	14125000	3053	nofs	nofs
chr11	E	11p15.2	1	14375000	2399	nofs	nofs
chr11	E	11p15.2	1	14625000	3504	nofs	nofs
chr11	E	11p15.2	1	14875000	5042	nofs	nofs
chr11	E	11p15.2	1	15125000	2884	nofs	nofs
chr11	E	11p15.2	1	15375000	2415	nofs	nofs
chr11	E	11p15.2	1	15625000	1913	nofs	nofs
chr11	E	11p15.2	1	15875000	2534	nofs	nofs
chr11	E	11p15.2	1	16125000	2457	nofs	nofs
chr11	E	11p15.1	4	16375000	1602	fs	fs common and rare

chr11	E	11p15.1	4	16625000	1738	fs	fs common and rare
chr11	E	11p15.1	4	16875000	3913	fs	fs common and rare
chr11	E	11p15.1	4	17125000	10418	fs	fs common and rare
chr11	E	11p15.1	4	17375000	3284	fs	fs common and rare
chr11	E	11p15.1	4	17625000	3815	fs	fs common and rare
chr11	E	11p15.1	4	17875000	1906	fs	fs common and rare
chr11	E	11p15.1	4	18125000	4591	fs	fs common and rare
chr11	E	11p15.1	4	18375000	3536	fs	fs common and rare
chr11	E	11p15.1	4	18625000	4428	fs	fs common and rare
chr11	E	11p15.1	4	18875000	1560	fs	fs common and rare
chr11	E	11p15.1	4	19125000	1456	fs	fs common and rare
chr11	E	11p15.1	4	19375000	1385	fs	fs common and rare
chr11	E	11p15.1	4	19625000	1620	fs	fs common and rare
chr11	E	11p15.1	4	19875000	3365	fs	fs common and rare
chr11	E	11p15.1	4	20125000	1839	fs	fs common and rare
chr11	E	11p15.1	4	20375000	2598	fs	fs common and rare
chr11	E	11p15.1	4	20625000	4013	fs	fs common and rare
chr11	E	11p15.1	4	20875000	2962	fs	fs common and rare
chr11	E	11p15.1	4	21125000	1668	fs	fs common and rare
chr11	E	11p15.1	4	21375000	2747	fs	fs common and rare
chr11	E	11p15.1	4	21625000	2079	fs	fs common and rare
chr11	E	11p14	4	21875000	2142	nofs	nofs
chr11	E	11p14	4	22125000	2359	nofs	nofs
chr11	E	11p14	4	22375000	3423	nofs	nofs
chr11	E	11p14	4	22625000	2912	nofs	nofs
chr11	E	11p14	4	22875000	2965	nofs	nofs
chr11	E	11p14	4	23125000	2196	nofs	nofs
chr11	E	11p14	4	23375000	2335	nofs	nofs
chr11	E	11p14	4	23625000	2926	nofs	nofs
chr11	E	11p14	4	23875000	4079	nofs	nofs
chr11	E	11p14	4	24125000	2240	nofs	nofs
chr11	E	11p14	4	24375000	2377	nofs	nofs
chr11	E	11p14	4	24625000	3767	nofs	nofs
chr11	E	11p14	4	24875000	5163	nofs	nofs
chr11	E	11p14	4	25125000	4116	nofs	nofs
chr11	E	11p14	4	25375000	2161	nofs	nofs

chr11	E	11p14	4	25625000	4141	nofs	nofs
chr11	E	11p14	4	25875000	3298	nofs	nofs
chr11	E	11p14	4	26125000	3020	fs	fs common
chr11	E	11p14	4	26375000	2737	fs	fs common
chr11	E	11p14	4	26625000	2981	fs	fs common
chr11	E	11p14	4	26875000	2316	fs	fs common
chr11	E	11p14	4	27125000	2334	fs	fs common
chr11	E	11p14	4	27375000	1927	fs	fs common
chr11	E	11p14	4	27625000	2093	nofs	nofs
chr11	E	11p14	4	27875000	2147	nofs	nofs
chr11	E	11p14	4	28125000	1361	nofs	nofs
chr11	E	11p14	4	28375000	3049	nofs	nofs
chr11	E	11p14	4	28625000	1883	nofs	nofs
chr11	E	11p14	4	28875000	2986	nofs	nofs
chr11	E	11p14	4	29125000	2353	nofs	nofs
chr11	E	11p14	4	29375000	3100	nofs	nofs
chr11	E	11p14	4	29625000	3020	nofs	nofs
chr11	E	11p14	4	29875000	2008	nofs	nofs
chr11	E	11p14	4	30125000	2343	nofs	nofs
chr11	E	11p14	4	30375000	1645	nofs	nofs
chr11	E	11p14	4	30625000	1999	nofs	nofs
chr11	E	11p14	4	30875000	3246	nofs	nofs
chr11	E	11p13	2	31125000	1368	fs	fs common
chr11	E	11p13	2	31375000	2131	fs	fs common
chr11	E	11p13	2	31625000	1708	fs	fs common
chr11	E	11p13	2	31875000	2624	fs	fs common
chr11	E	11p13	2	32125000	2462	fs	fs common
chr11	E	11p13	2	32375000	3261	fs	fs common
chr11	E	11p13	2	32625000	4302	fs	fs common
chr11	E	11p13	2	32875000	3033	fs	fs common
chr11	E	11p13	2	33125000	3614	fs	fs common
chr11	E	11p13	2	33375000	4157	fs	fs common
chr11	E	11p13	2	33625000	1629	fs	fs common
chr11	E	11p13	2	33875000	3682	fs	fs common
chr11	E	11p13	2	34125000	4191	fs	fs common
chr11	E	11p13	2	34375000	3567	fs	fs common

chr11	E	11p13	2	34625000	1762	fs	fs common
chr11	E	11p13	2	34875000	2237	fs	fs common
chr11	E	11p13	2	35125000	2641	fs	fs common
chr11	E	11p13	2	35375000	1829	fs	fs common
chr11	E	11p13	2	35625000	2042	fs	fs common
chr11	E	11p13	2	35875000	1492	fs	fs common
chr11	E	11p13	2	36125000	3701	fs	fs common
chr11	E	11p13	2	36375000	4473	fs	fs common
chr11	B	11p12	.	36625000	1701	nofs	nofs
chr11	B	11p12	.	36875000	1766	nofs	nofs
chr11	B	11p12	.	37125000	1994	nofs	nofs
chr11	B	11p12	.	37375000	4829	nofs	nofs
chr11	B	11p12	.	37625000	4861	nofs	nofs
chr11	B	11p12	.	37875000	2452	nofs	nofs
chr11	B	11p12	.	38125000	2995	nofs	nofs
chr11	B	11p12	.	38375000	2225	nofs	nofs
chr11	B	11p12	.	38625000	2232	nofs	nofs
chr11	B	11p12	.	38875000	2482	nofs	nofs
chr11	B	11p12	.	39125000	3732	nofs	nofs
chr11	B	11p12	.	39375000	3124	nofs	nofs
chr11	B	11p12	.	39625000	4465	nofs	nofs
chr11	B	11p12	.	39875000	1400	nofs	nofs
chr11	B	11p12	.	40125000	1431	nofs	nofs
chr11	B	11p12	.	40375000	1953	nofs	nofs
chr11	B	11p12	.	40625000	1672	nofs	nofs
chr11	B	11p12	.	40875000	2866	nofs	nofs
chr11	B	11p12	.	41125000	1444	nofs	nofs
chr11	B	11p12	.	41375000	4275	nofs	nofs
chr11	B	11p12	.	41625000	5110	nofs	nofs
chr11	B	11p12	.	41875000	3090	nofs	nofs
chr11	B	11p12	.	42125000	2371	nofs	nofs
chr11	B	11p12	.	42375000	2100	nofs	nofs
chr11	B	11p12	.	42625000	2022	nofs	nofs
chr11	B	11p12	.	42875000	4386	nofs	nofs
chr11	B	11p12	.	43125000	12303	nofs	nofs
chr11	B	11p12	.	43375000	3380	nofs	nofs

chr11	E	11p11.2	1	43625000	2043	nofs	nofs
chr11	E	11p11.2	1	43875000	2461	nofs	nofs
chr11	E	11p11.2	1	44125000	2004	nofs	nofs
chr11	E	11p11.2	1	44375000	3206	nofs	nofs
chr11	E	11p11.2	1	44625000	3405	nofs	nofs
chr11	E	11p11.2	1	44875000	3184	nofs	nofs
chr11	E	11p11.2	1	45125000	3244	nofs	nofs
chr11	E	11p11.2	1	45375000	2539	nofs	nofs
chr11	E	11p11.2	1	45625000	3426	nofs	nofs
chr11	E	11p11.2	1	45875000	2316	nofs	nofs
chr11	E	11p11.2	1	46125000	2415	nofs	nofs
chr11	E	11p11.2	1	46375000	3288	nofs	nofs
chr11	E	11p11.2	1	46625000	3080	nofs	nofs
chr11	E	11p11.2	1	46875000	2897	nofs	nofs
chr11	E	11p11.2	1	47125000	4689	nofs	nofs
chr11	E	11p11.2	1	47375000	3439	nofs	nofs
chr11	E	11p11.2	1	47625000	6935	nofs	nofs
chr11	E	11p11.2	1	47875000	4572	nofs	nofs
chr11	E	11p11.2	1	48125000	3843	nofs	nofs
chr11	C	11p11.2	1	48375000	1323	nofs	nofs
chr11	C	11p11.2	1	48625000	81404	nofs	nofs
chr11	C	11p11.12	1	48875000	127407	nofs	nofs
chr11	C	11p11.12	1	49125000	2749	nofs	nofs
chr11	C	11p11.12	1	49375000	2642	nofs	nofs
chr11	C	11p11.12	1	49625000	2389	nofs	nofs
chr11	C	11p11.12	1	49875000	5892	nofs	nofs
chr11	C	11p11.12	1	50125000	11229	nofs	nofs
chr11	C	11p11.12	1	50375000	148952	nofs	nofs
chr11	C	11p11.12	1	50625000	192312	nofs	nofs
chr11	C	11p11.12	1	50875000	41109	nofs	nofs
chr11	C	11p11.12	1	51125000	166893	nofs	nofs
chr11	C	11p11.12	1	51375000	27739	nofs	nofs
chr11	C		.	51625000	0	nofs	nofs
chr11	C		.	51875000	0	nofs	nofs
chr11	C		.	52125000	0	nofs	nofs
chr11	C		.	52375000	0	nofs	nofs

chr11	C		.	52625000	0	nofs	nofs
chr11	C		.	52875000	0	nofs	nofs
chr11	C		.	53125000	0	nofs	nofs
chr11	C		.	53375000	0	nofs	nofs
chr11	C		.	53625000	0	nofs	nofs
chr11	C		.	53875000	0	nofs	nofs
chr11	C		.	54125000	0	nofs	nofs
chr11	C	11q11	.	54375000	37996	nofs	nofs
chr11	C	11q11	.	54625000	197463	nofs	nofs
chr11	C	11q11	.	54875000	32427	nofs	nofs
chr11	C	11q11	.	55125000	2175	nofs	nofs
chr11	C	11q11	.	55375000	2829	nofs	nofs
chr11	C	11q11	.	55625000	4258	nofs	nofs
chr11	C	11q11	.	55875000	3066	nofs	nofs
chr11	C	11q11	.	56125000	3324	nofs	nofs
chr11	C	11q11	.	56375000	2746	nofs	nofs
chr11	C	11q11	.	56625000	3096	nofs	nofs
chr11	C	11q12	4	56875000	2746	nofs	nofs
chr11	C	11q12	4	57125000	4872	nofs	nofs
chr11	C	11q12	4	57375000	2518	nofs	nofs
chr11	E	11q12	4	57625000	2436	nofs	nofs
chr11	E	11q12	4	57875000	2140	nofs	nofs
chr11	E	11q12	4	58125000	8855	nofs	nofs
chr11	E	11q12	4	58375000	1462	nofs	nofs
chr11	E	11q12	4	58625000	1592	nofs	nofs
chr11	E	11q12	4	58875000	1926	nofs	nofs
chr11	E	11q12	4	59125000	4463	nofs	nofs
chr11	E	11q12	4	59375000	2386	nofs	nofs
chr11	E	11q12	4	59625000	3938	nofs	nofs
chr11	E	11q12	4	59875000	3275	nofs	nofs
chr11	E	11q12	4	60125000	6380	nofs	nofs
chr11	E	11q12	4	60375000	3793	nofs	nofs
chr11	E	11q12	4	60625000	7298	nofs	nofs
chr11	E	11q12	4	60875000	3406	nofs	nofs
chr11	E	11q12	4	61125000	4603	nofs	nofs
chr11	E	11q12	4	61375000	3526	nofs	nofs

chr11	E	11q12	4	61625000	3766	nofs	nofs
chr11	E	11q12	4	61875000	5741	nofs	nofs
chr11	E	11q12	4	62125000	13287	nofs	nofs
chr11	E	11q12	4	62375000	5637	nofs	nofs
chr11	E	11q12	4	62625000	2721	nofs	nofs
chr11	E	11q12	4	62875000	2342	nofs	nofs
chr11	E	11q13.1	1	63125000	3149	fs	fs common and rare
chr11	E	11q13.1	1	63375000	5642	fs	fs common and rare
chr11	E	11q13.1	1	63625000	7896	fs	fs common and rare
chr11	E	11q13.1	1	63875000	6969	fs	fs common and rare
chr11	E	11q13.1	1	64125000	8063	fs	fs common and rare
chr11	E	11q13.1	1	64375000	4049	fs	fs common and rare
chr11	E	11q13.1	1	64625000	3242	fs	fs common and rare
chr11	E	11q13.1	1	64875000	8779	fs	fs common and rare
chr11	E	11q13.1	1	65125000	3993	fs	fs common and rare
chr11	E	11q13.1	1	65375000	6054	fs	fs common and rare
chr11	E	11q13.1	1	65625000	2987	fs	fs common and rare
chr11	B	11q13.2	.	65875000	5019	fs	fs common and rare
chr11	B	11q13.2	.	66125000	4364	fs	fs common and rare
chr11	B	11q13.2	.	66375000	2777	fs	fs common and rare
chr11	B	11q13.2	.	66625000	5890	fs	fs common and rare
chr11	B	11q13.2	.	66875000	2867	fs	fs common and rare
chr11	B	11q13.2	.	67125000	6515	fs	fs common and rare
chr11	B	11q13.2	.	67375000	4564	fs	fs common and rare
chr11	B	11q13.2	.	67625000	4040	fs	fs common and rare
chr11	B	11q13.2	.	67875000	7575	fs	fs common and rare
chr11	B	11q13.2	.	68125000	5089	fs	fs common and rare
chr11	E	11q13.3	5	68375000	7643	fs	fs common and rare
chr11	E	11q13.3	5	68625000	10933	fs	fs common and rare
chr11	E	11q13.3	5	68875000	9012	fs	fs common and rare
chr11	E	11q13.3	5	69125000	6590	fs	fs common and rare
chr11	E	11q13.3	5	69375000	9256	fs	fs common and rare
chr11	E	11q13.3	5	69625000	12168	fs	fs common and rare
chr11	E	11q13.3	5	69875000	5804	fs	fs common and rare
chr11	E	11q13.3	5	70125000	4703	fs	fs common and rare
chr11	E	11q13.4	7	70375000	11162	fs	fs common and rare

chr11	E	11q13.4	7	70625000	7326	fs	fs common and rare
chr11	E	11q13.4	7	70875000	16613	fs	fs common and rare
chr11	E	11q13.4	7	71125000	6440	fs	fs common and rare
chr11	E	11q13.4	7	71375000	4311	fs	fs common and rare
chr11	E	11q13.4	7	71625000	4322	fs	fs common and rare
chr11	E	11q13.4	7	71875000	5325	fs	fs common and rare
chr11	E	11q13.4	7	72125000	2681	fs	fs common and rare
chr11	E	11q13.4	7	72375000	2992	fs	fs common and rare
chr11	E	11q13.4	7	72625000	3450	fs	fs common and rare
chr11	E	11q13.4	7	72875000	3155	fs	fs common and rare
chr11	E	11q13.4	7	73125000	4289	fs	fs common and rare
chr11	E	11q13.4	7	73375000	5753	fs	fs common and rare
chr11	E	11q13.4	7	73625000	3495	fs	fs common and rare
chr11	E	11q13.4	7	73875000	3063	fs	fs common and rare
chr11	E	11q13.4	7	74125000	1134	fs	fs common and rare
chr11	E	11q13.4	7	74375000	2271	fs	fs common and rare
chr11	E	11q13.4	7	74625000	2984	fs	fs common and rare
chr11	E	11q13.4	7	74875000	2641	fs	fs common and rare
chr11	E	11q13.5	2	75125000	1392	fs	fs common and rare
chr11	E	11q13.5	2	75375000	3319	fs	fs common and rare
chr11	E	11q13.5	2	75625000	3593	fs	fs common and rare
chr11	E	11q13.5	2	75875000	3277	fs	fs common and rare
chr11	E	11q13.5	2	76125000	4972	fs	fs common and rare
chr11	E	11q13.5	2	76375000	4710	fs	fs common and rare
chr11	E	11q13.5	2	76625000	2660	fs	fs common and rare
chr11	E	11q13.5	2	76875000	3638	fs	fs common and rare
chr11	E	11q13.5	2	77125000	2248	fs	fs common and rare
chr11	E	11q13.5	2	77375000	3603	fs	fs common and rare
chr11	E	11q14.1	1	77625000	2834	nofs	nofs
chr11	E	11q14.1	1	77875000	1885	nofs	nofs
chr11	E	11q14.1	1	78125000	1617	nofs	nofs
chr11	E	11q14.1	1	78375000	2302	nofs	nofs
chr11	E	11q14.1	1	78625000	5152	nofs	nofs
chr11	E	11q14.1	1	78875000	2430	nofs	nofs
chr11	E	11q14.1	1	79125000	3331	nofs	nofs
chr11	E	11q14.1	1	79375000	1689	nofs	nofs

chr11	E	11q14.1	1	79625000	2284	nofs	nofs
chr11	E	11q14.1	1	79875000	2862	nofs	nofs
chr11	E	11q14.1	1	80125000	2071	nofs	nofs
chr11	E	11q14.1	1	80375000	3040	nofs	nofs
chr11	E	11q14.1	1	80625000	2625	nofs	nofs
chr11	E	11q14.1	1	80875000	3958	nofs	nofs
chr11	E	11q14.1	1	81125000	2059	nofs	nofs
chr11	E	11q14.1	1	81375000	3567	nofs	nofs
chr11	E	11q14.1	1	81625000	2619	nofs	nofs
chr11	E	11q14.1	1	81875000	2479	nofs	nofs
chr11	E	11q14.1	1	82125000	3039	nofs	nofs
chr11	E	11q14.1	1	82375000	2044	nofs	nofs
chr11	E	11q14.1	1	82625000	3795	nofs	nofs
chr11	E	11q14.1	1	82875000	3325	nofs	nofs
chr11	E	11q14.1	1	83125000	2771	nofs	nofs
chr11	E	11q14.1	1	83375000	2050	nofs	nofs
chr11	E	11q14.1	1	83625000	2306	nofs	nofs
chr11	E	11q14.1	1	83875000	1879	nofs	nofs
chr11	E	11q14.1	1	84125000	2516	nofs	nofs
chr11	E	11q14.1	1	84375000	1691	nofs	nofs
chr11	E	11q14.1	1	84625000	1538	nofs	nofs
chr11	E	11q14.1	1	84875000	2571	nofs	nofs
chr11	E	11q14.1	1	85125000	2474	nofs	nofs
chr11	E	11q14.1	1	85375000	2879	nofs	nofs
chr11	B	11q14.2	.	85625000	4625	fs	fs common
chr11	B	11q14.2	.	85875000	1476	fs	fs common
chr11	B	11q14.2	.	86125000	7921	fs	fs common
chr11	B	11q14.2	.	86375000	4696	fs	fs common
chr11	B	11q14.2	.	86625000	2699	fs	fs common
chr11	B	11q14.2	.	86875000	2065	fs	fs common
chr11	B	11q14.2	.	87125000	2683	fs	fs common
chr11	B	11q14.2	.	87375000	4495	fs	fs common
chr11	B	11q14.2	.	87625000	3526	fs	fs common
chr11	B	11q14.2	.	87875000	2016	fs	fs common
chr11	E	11q14.3	2	88125000	2139	nofs	nofs
chr11	E	11q14.3	2	88375000	2028	nofs	nofs

chr11	E	11q14.3	2	88625000	2889	nofs	nofs
chr11	E	11q14.3	2	88875000	3530	nofs	nofs
chr11	E	11q14.3	2	89125000	3339	nofs	nofs
chr11	E	11q14.3	2	89375000	11099	nofs	nofs
chr11	E	11q14.3	2	89625000	3159	nofs	nofs
chr11	E	11q14.3	2	89875000	2031	nofs	nofs
chr11	E	11q14.3	2	90125000	1849	nofs	nofs
chr11	E	11q14.3	2	90375000	2749	nofs	nofs
chr11	E	11q14.3	2	90625000	3176	nofs	nofs
chr11	E	11q14.3	2	90875000	3178	nofs	nofs
chr11	E	11q14.3	2	91125000	1176	nofs	nofs
chr11	E	11q14.3	2	91375000	1544	nofs	nofs
chr11	E	11q14.3	2	91625000	3192	nofs	nofs
chr11	E	11q14.3	2	91875000	2311	nofs	nofs
chr11	E	11q14.3	2	92125000	1509	nofs	nofs
chr11	B	11q21	.	92375000	4459	nofs	nofs
chr11	B	11q21	.	92625000	1927	nofs	nofs
chr11	B	11q21	.	92875000	3111	nofs	nofs
chr11	B	11q21	.	93125000	1786	nofs	nofs
chr11	B	11q21	.	93375000	3582	nofs	nofs
chr11	B	11q21	.	93625000	2799	nofs	nofs
chr11	B	11q21	.	93875000	1342	nofs	nofs
chr11	B	11q21	.	94125000	1222	nofs	nofs
chr11	B	11q21	.	94375000	2393	nofs	nofs
chr11	B	11q21	.	94625000	3488	nofs	nofs
chr11	B	11q21	.	94875000	2446	nofs	nofs
chr11	B	11q21	.	95125000	1512	nofs	nofs
chr11	B	11q21	.	95375000	3491	nofs	nofs
chr11	B	11q21	.	95625000	1523	nofs	nofs
chr11	B	11q21	.	95875000	2202	nofs	nofs
chr11	B	11q21	.	96125000	1849	nofs	nofs
chr11	B	11q21	.	96375000	2243	nofs	nofs
chr11	E	11q22.1	2	96625000	1904	nofs	nofs
chr11	E	11q22.1	2	96875000	2837	nofs	nofs
chr11	E	11q22.1	2	97125000	3556	nofs	nofs
chr11	E	11q22.1	2	97375000	2831	nofs	nofs

chr11	E	11q22.1	2	97625000	2510	nofs	nofs
chr11	E	11q22.1	2	97875000	3734	nofs	nofs
chr11	E	11q22.1	2	98125000	3874	nofs	nofs
chr11	E	11q22.1	2	98375000	1938	nofs	nofs
chr11	E	11q22.1	2	98625000	2615	nofs	nofs
chr11	E	11q22.1	2	98875000	2278	nofs	nofs
chr11	E	11q22.1	2	99125000	2289	nofs	nofs
chr11	E	11q22.1	2	99375000	1716	nofs	nofs
chr11	E	11q22.1	2	99625000	1457	nofs	nofs
chr11	E	11q22.1	2	99875000	1502	nofs	nofs
chr11	E	11q22.1	2	100000000	3335	nofs	nofs
chr11	E	11q22.1	2	100000000	1811	nofs	nofs
chr11	E	11q22.1	2	101000000	3913	nofs	nofs
chr11	E	11q22.1	2	101000000	2513	nofs	nofs
chr11	E	11q22.1	2	101000000	1753	nofs	nofs
chr11	E	11q22.2	1	101000000	2535	nofs	nofs
chr11	E	11q22.2	1	102000000	1874	nofs	nofs
chr11	E	11q22.2	1	102000000	2015	nofs	nofs
chr11	E	11q22.2	1	102000000	3553	nofs	nofs
chr11	E	11q22.2	1	102000000	2802	nofs	nofs
chr11	E	11q22.3	4	103000000	1849	nofs	nofs
chr11	E	11q22.3	4	103000000	1857	nofs	nofs
chr11	E	11q22.3	4	103000000	2164	nofs	nofs
chr11	E	11q22.3	4	103000000	3069	nofs	nofs
chr11	E	11q22.3	4	104000000	3215	nofs	nofs
chr11	E	11q22.3	4	104000000	4764	nofs	nofs
chr11	E	11q22.3	4	104000000	2680	nofs	nofs
chr11	E	11q22.3	4	104000000	2509	nofs	nofs
chr11	E	11q22.3	4	105000000	2958	nofs	nofs
chr11	E	11q22.3	4	105000000	2273	nofs	nofs
chr11	E	11q22.3	4	105000000	2305	nofs	nofs
chr11	E	11q22.3	4	105000000	1836	nofs	nofs
chr11	E	11q22.3	4	106000000	2882	nofs	nofs
chr11	E	11q22.3	4	106000000	2171	nofs	nofs
chr11	E	11q22.3	4	106000000	4421	nofs	nofs
chr11	E	11q22.3	4	106000000	2458	nofs	nofs

chr11	E	11q22.3	4	107000000	2085	nofs	nofs
chr11	E	11q22.3	4	107000000	1883	nofs	nofs
chr11	E	11q22.3	4	107000000	4187	nofs	nofs
chr11	E	11q22.3	4	107000000	4205	nofs	nofs
chr11	E	11q22.3	4	108000000	1768	nofs	nofs
chr11	E	11q22.3	4	108000000	2508	nofs	nofs
chr11	E	11q22.3	4	108000000	1645	nofs	nofs
chr11	E	11q22.3	4	108000000	2857	nofs	nofs
chr11	E	11q22.3	4	109000000	3791	nofs	nofs
chr11	E	11q22.3	4	109000000	2827	nofs	nofs
chr11	E	11q22.3	4	109000000	1738	nofs	nofs
chr11	E	11q22.3	4	109000000	3018	nofs	nofs
chr11	E	11q22.3	4	110000000	2523	nofs	nofs
chr11	E	11q22.3	4	110000000	2893	nofs	nofs
chr11	E	11q22.3	4	110000000	1238	nofs	nofs
chr11	E	11q23.1	1	110000000	2811	nofs	nofs
chr11	E	11q23.1	1	111000000	2965	nofs	nofs
chr11	E	11q23.1	1	111000000	1875	nofs	nofs
chr11	E	11q23.1	1	111000000	2048	nofs	nofs
chr11	E	11q23.1	1	111000000	4214	nofs	nofs
chr11	E	11q23.1	1	112000000	4096	nofs	nofs
chr11	E	11q23.1	1	112000000	1727	nofs	nofs
chr11	E	11q23.1	1	112000000	2154	nofs	nofs
chr11	E	11q23.1	1	112000000	2186	nofs	nofs
chr11	E	11q23.1	1	113000000	2088	nofs	nofs
chr11	E	11q23.1	1	113000000	2301	nofs	nofs
chr11	B	11q23.2	.	113000000	3234	nofs	nofs
chr11	B	11q23.2	.	113000000	2107	nofs	nofs
chr11	B	11q23.2	.	114000000	2560	nofs	nofs
chr11	B	11q23.2	.	114000000	23797	nofs	nofs
chr11	B	11q23.2	.	114000000	6376	nofs	nofs
chr11	B	11q23.2	.	114000000	2119	nofs	nofs
chr11	B	11q23.3	.	115000000	3345	fs	fs common and rare
chr11	B	11q23.3	.	115000000	2155	fs	fs common and rare
chr11	B	11q23.3	.	115000000	4425	fs	fs common and rare
chr11	B	11q23.3	.	115000000	1753	fs	fs common and rare

chr11	B	11q23.3	.	116000000	3583	fs	fs common and rare
chr11	B	11q23.3	.	116000000	2016	fs	fs common and rare
chr11	B	11q23.3	.	116000000	5342	fs	fs common and rare
chr11	B	11q23.3	.	116000000	3093	fs	fs common and rare
chr11	B	11q23.3	.	117000000	3257	fs	fs common and rare
chr11	B	11q23.3	.	117000000	3043	fs	fs common and rare
chr11	B	11q23.3	.	117000000	4358	fs	fs common and rare
chr11	B	11q23.3	.	117000000	2808	fs	fs common and rare
chr11	B	11q23.3	.	118000000	4455	fs	fs common and rare
chr11	B	11q23.3	.	118000000	4103	fs	fs common and rare
chr11	B	11q23.3	.	118000000	3649	fs	fs common and rare
chr11	B	11q23.3	.	118000000	5002	fs	fs common and rare
chr11	B	11q23.3	.	119000000	3726	fs	fs common and rare
chr11	B	11q23.3	.	119000000	4267	fs	fs common and rare
chr11	B	11q23.3	.	119000000	2756	fs	fs common and rare
chr11	B	11q23.3	.	119000000	6980	fs	fs common and rare
chr11	B	11q23.3	.	120000000	3144	fs	fs common and rare
chr11	B	11q23.3	.	120000000	1378	fs	fs common and rare
chr11	B	11q23.3	.	120000000	2776	fs	fs common and rare
chr11	B	11q23.3	.	120000000	2916	fs	fs common and rare
chr11	B	11q23.3	.	121000000	2980	fs	fs common and rare
chr11	B	11q23.3	.	121000000	3666	fs	fs common and rare
chr11	B	11q23.3	.	121000000	2533	fs	fs common and rare
chr11	E	11q24	1	121000000	5462	nofs	nofs
chr11	E	11q24	1	122000000	2515	nofs	nofs
chr11	E	11q24	1	122000000	4116	nofs	nofs
chr11	E	11q24	1	122000000	3093	nofs	nofs
chr11	E	11q24	1	122000000	3308	nofs	nofs
chr11	E	11q24	1	123000000	4077	nofs	nofs
chr11	E	11q24	1	123000000	3323	nofs	nofs
chr11	E	11q24	1	123000000	2285	nofs	nofs
chr11	E	11q24	1	123000000	2748	nofs	nofs
chr11	E	11q24	1	124000000	2846	nofs	nofs
chr11	E	11q24	1	124000000	3267	nofs	nofs
chr11	E	11q24	1	124000000	1600	nofs	nofs
chr11	E	11q24	1	124000000	2889	nofs	nofs

chr11	E	11q24	1	125000000	3268	nofs	nofs
chr11	E	11q24	1	125000000	3739	nofs	nofs
chr11	E	11q24	1	125000000	3269	nofs	nofs
chr11	E	11q24	1	125000000	2098	nofs	nofs
chr11	E	11q24	1	126000000	3545	nofs	nofs
chr11	E	11q24	1	126000000	5291	nofs	nofs
chr11	E	11q24	1	126000000	2918	nofs	nofs
chr11	E	11q24	1	126000000	4468	nofs	nofs
chr11	E	11q24	1	127000000	3086	nofs	nofs
chr11	E	11q24	1	127000000	4060	nofs	nofs
chr11	E	11q24	1	127000000	2591	nofs	nofs
chr11	E	11q24	1	127000000	3501	nofs	nofs
chr11	E	11q24	1	128000000	2272	nofs	nofs
chr11	E	11q24	1	128000000	2331	nofs	nofs
chr11	E	11q24	1	128000000	2893	nofs	nofs
chr11	E	11q24	1	128000000	2811	nofs	nofs
chr11	E	11q24	1	129000000	2895	nofs	nofs
chr11	E	11q24	1	129000000	3422	nofs	nofs
chr11	E	11q24	1	129000000	4928	nofs	nofs
chr11	E	11q24	1	129000000	4986	nofs	nofs
chr11	E	11q24	1	130000000	2021	nofs	nofs
chr11	E	11q24	1	130000000	3998	nofs	nofs
chr11	B	11q25	.	130000000	2348	nofs	nofs
chr11	B	11q25	.	130000000	2272	nofs	nofs
chr11	B	11q25	.	131000000	4945	nofs	nofs
chr11	B	11q25	.	131000000	2916	nofs	nofs
chr11	T	11q25	.	131000000	6224	nofs	nofs
chr11	T	11q25	.	131000000	4887	nofs	nofs
chr11	T	11q25	.	132000000	2476	nofs	nofs
chr11	T	11q25	.	132000000	3308	nofs	nofs
chr11	T	11q25	.	132000000	2590	nofs	nofs
chr11	T	11q25	.	132000000	3480	nofs	nofs
chr11	T	11q25	.	133000000	3313	nofs	nofs
chr11	T	11q25	.	133000000	5513	nofs	nofs
chr11	T	11q25	.	133000000	4265	nofs	nofs
chr11	T	11q25	.	133000000	8962	nofs	nofs

chr11	T	11q25	.	134000000	8140	nofs	nofs
chr11	T	11q25	.	134000000	13643	nofs	nofs
chr11	T	11q25	.	134000000	11852	nofs	nofs
chr12	T	12p13.3	3	125000	7021	nofs	nofs
chr12	T	12p13.3	3	375000	9579	nofs	nofs
chr12	T	12p13.3	3	625000	9151	nofs	nofs
chr12	T	12p13.3	3	875000	4033	nofs	nofs
chr12	T	12p13.3	3	1125000	12362	nofs	nofs
chr12	T	12p13.3	3	1375000	3174	nofs	nofs
chr12	T	12p13.3	3	1625000	5240	nofs	nofs
chr12	T	12p13.3	3	1875000	7012	nofs	nofs
chr12	T	12p13.3	3	2125000	4748	nofs	nofs
chr12	T	12p13.3	3	2375000	4487	nofs	nofs
chr12	T	12p13.3	3	2625000	7035	nofs	nofs
chr12	T	12p13.3	3	2875000	5446	nofs	nofs
chr12	T	12p13.3	3	3125000	5998	nofs	nofs
chr12	E	12p13.3	3	3375000	2177	nofs	nofs
chr12	E	12p13.3	3	3625000	3794	nofs	nofs
chr12	E	12p13.3	3	3875000	2786	nofs	nofs
chr12	E	12p13.3	3	4125000	3346	nofs	nofs
chr12	E	12p13.3	3	4375000	2169	nofs	nofs
chr12	E	12p13.3	3	4625000	2440	nofs	nofs
chr12	E	12p13.3	3	4875000	3769	nofs	nofs
chr12	E	12p13.3	3	5125000	2664	nofs	nofs
chr12	E	12p13.3	3	5375000	2707	nofs	nofs
chr12	E	12p13.3	3	5625000	5164	nofs	nofs
chr12	E	12p13.3	3	5875000	8837	nofs	nofs
chr12	E	12p13.3	3	6125000	3025	nofs	nofs
chr12	E	12p13.3	3	6375000	6540	nofs	nofs
chr12	E	12p13.3	3	6625000	3073	nofs	nofs
chr12	E	12p13.3	3	6875000	3760	nofs	nofs
chr12	E	12p13.3	3	7125000	5557	nofs	nofs
chr12	E	12p13.3	3	7375000	3641	nofs	nofs
chr12	E	12p13.3	3	7625000	4174	nofs	nofs
chr12	E	12p13.3	3	7875000	8686	nofs	nofs
chr12	E	12p13.3	3	8125000	5003	nofs	nofs

chr12	E	12p13.3	3	8375000	4596	nofs	nofs
chr12	E	12p13.3	3	8625000	2525	nofs	nofs
chr12	E	12p13.3	3	8875000	3629	nofs	nofs
chr12	E	12p13.3	3	9125000	4036	nofs	nofs
chr12	E	12p13.3	3	9375000	7346	nofs	nofs
chr12	E	12p13.3	3	9625000	3754	nofs	nofs
chr12	E	12p13.3	3	9875000	3450	nofs	nofs
chr12	E	12p13.3	3	10125000	2652	nofs	nofs
chr12	B	12p13.2	.	10375000	3146	nofs	nofs
chr12	B	12p13.2	.	10625000	3253	nofs	nofs
chr12	B	12p13.2	.	10875000	2428	nofs	nofs
chr12	B	12p13.2	.	11125000	1903	nofs	nofs
chr12	B	12p13.2	.	11375000	5106	nofs	nofs
chr12	B	12p13.2	.	11625000	2858	nofs	nofs
chr12	B	12p13.2	.	11875000	1770	nofs	nofs
chr12	B	12p13.2	.	12125000	3420	nofs	nofs
chr12	B	12p13.2	.	12375000	4758	nofs	nofs
chr12	B	12p13.2	.	12625000	4238	nofs	nofs
chr12	B	12p13.2	.	12875000	4341	nofs	nofs
chr12	B	12p13.1	.	13125000	4526	nofs	nofs
chr12	B	12p13.1	.	13375000	1781	nofs	nofs
chr12	B	12p13.1	.	13625000	3100	nofs	nofs
chr12	B	12p13.1	.	13875000	1173	nofs	nofs
chr12	B	12p13.1	.	14125000	4849	nofs	nofs
chr12	B	12p13.1	.	14375000	4639	nofs	nofs
chr12	B	12p13.1	.	14625000	2777	nofs	nofs
chr12	B	12p13.1	.	14875000	1952	nofs	nofs
chr12	B	12p12.3	.	15125000	1799	nofs	nofs
chr12	B	12p12.3	.	15375000	2048	nofs	nofs
chr12	B	12p12.3	.	15625000	2282	nofs	nofs
chr12	B	12p12.3	.	15875000	1652	nofs	nofs
chr12	B	12p12.3	.	16125000	1734	nofs	nofs
chr12	B	12p12.3	.	16375000	2841	nofs	nofs
chr12	B	12p12.3	.	16625000	1733	nofs	nofs
chr12	B	12p12.3	.	16875000	2112	nofs	nofs
chr12	B	12p12.3	.	17125000	2255	nofs	nofs

chr12	B	12p12.3	.	17375000	2610	nofs	nofs
chr12	B	12p12.3	.	17625000	2078	nofs	nofs
chr12	B	12p12.3	.	17875000	2107	nofs	nofs
chr12	B	12p12.3	.	18125000	5762	nofs	nofs
chr12	B	12p12.3	.	18375000	2840	nofs	nofs
chr12	B	12p12.3	.	18625000	1478	nofs	nofs
chr12	B	12p12.3	.	18875000	2511	nofs	nofs
chr12	B	12p12.3	.	19125000	2305	nofs	nofs
chr12	B	12p12.3	.	19375000	3303	nofs	nofs
chr12	B	12p12.3	.	19625000	3176	nofs	nofs
chr12	B	12p12.3	.	19875000	2795	nofs	nofs
chr12	B	12p12.2	.	20125000	4501	nofs	nofs
chr12	B	12p12.2	.	20375000	4059	nofs	nofs
chr12	B	12p12.2	.	20625000	2162	nofs	nofs
chr12	B	12p12.2	.	20875000	3680	nofs	nofs
chr12	B	12p12.1	.	21125000	1337	nofs	nofs
chr12	B	12p12.1	.	21375000	2262	nofs	nofs
chr12	B	12p12.1	.	21625000	3147	nofs	nofs
chr12	B	12p12.1	.	21875000	1637	nofs	nofs
chr12	B	12p12.1	.	22125000	2671	nofs	nofs
chr12	B	12p12.1	.	22375000	3765	nofs	nofs
chr12	B	12p12.1	.	22625000	3045	nofs	nofs
chr12	B	12p12.1	.	22875000	3160	nofs	nofs
chr12	B	12p12.1	.	23125000	3256	nofs	nofs
chr12	B	12p12.1	.	23375000	1525	nofs	nofs
chr12	B	12p12.1	.	23625000	2310	nofs	nofs
chr12	B	12p12.1	.	23875000	3625	nofs	nofs
chr12	B	12p12.1	.	24125000	2808	nofs	nofs
chr12	B	12p12.1	.	24375000	1964	nofs	nofs
chr12	B	12p12.1	.	24625000	1512	nofs	nofs
chr12	B	12p12.1	.	24875000	2930	nofs	nofs
chr12	B	12p12.1	.	25125000	3295	nofs	nofs
chr12	B	12p12.1	.	25375000	2886	nofs	nofs
chr12	B	12p12.1	.	25625000	1784	nofs	nofs
chr12	B	12p12.1	.	25875000	2027	nofs	nofs
chr12	B	12p12.1	.	26125000	2285	nofs	nofs

chr12	B	12p12.1	.	26375000	1862	nofs	nofs
chr12	E	12p11.2	1	26625000	3396	nofs	nofs
chr12	E	12p11.2	1	26875000	4014	nofs	nofs
chr12	E	12p11.2	1	27125000	2613	nofs	nofs
chr12	E	12p11.2	1	27375000	2825	nofs	nofs
chr12	E	12p11.2	1	27625000	1475	nofs	nofs
chr12	E	12p11.2	1	27875000	3207	nofs	nofs
chr12	E	12p11.2	1	28125000	3890	nofs	nofs
chr12	E	12p11.2	1	28375000	2925	nofs	nofs
chr12	E	12p11.2	1	28625000	1792	nofs	nofs
chr12	E	12p11.2	1	28875000	2669	nofs	nofs
chr12	E	12p11.2	1	29125000	4026	nofs	nofs
chr12	E	12p11.2	1	29375000	3548	nofs	nofs
chr12	E	12p11.2	1	29625000	2862	nofs	nofs
chr12	E	12p11.2	1	29875000	2038	nofs	nofs
chr12	E	12p11.2	1	30125000	2289	nofs	nofs
chr12	E	12p11.2	1	30375000	1960	nofs	nofs
chr12	E	12p11.2	1	30625000	1266	nofs	nofs
chr12	E	12p11.2	1	30875000	2169	nofs	nofs
chr12	E	12p11.2	1	31125000	6232	nofs	nofs
chr12	E	12p11.2	1	31375000	4738	nofs	nofs
chr12	C	12p11.2	1	31625000	3969	nofs	nofs
chr12	C	12p11.2	1	31875000	5116	nofs	nofs
chr12	C	12p11.2	1	32125000	5453	nofs	nofs
chr12	C	12p11.2	1	32375000	2967	nofs	nofs
chr12	C	12p11.2	1	32625000	2321	nofs	nofs
chr12	C	12p11.2	1	32875000	4261	nofs	nofs
chr12	C	12p11.2	1	33125000	2953	nofs	nofs
chr12	C	12p11.1	.	33375000	2922	nofs	nofs
chr12	C	12p11.1	.	33625000	2064	nofs	nofs
chr12	C	12p11.1	.	33875000	2136	nofs	nofs
chr12	C	12p11.1	.	34125000	3621	nofs	nofs
chr12	C	12p11.1	.	34375000	123919	nofs	nofs
chr12	C	12p11.1	.	34625000	174716	nofs	nofs
chr12	C		.	34875000	0	nofs	nofs
chr12	C		.	35125000	0	nofs	nofs

chr12	C		.	35375000	0	nofs	nofs
chr12	C		.	35625000	0	nofs	nofs
chr12	C		.	35875000	0	nofs	nofs
chr12	C	12q11	.	36125000	6975	nofs	nofs
chr12	C	12q11	.	36375000	195951	nofs	nofs
chr12	C	12q11	.	36625000	193880	nofs	nofs
chr12	C	12q11	.	36875000	5340	nofs	nofs
chr12	C	12q11	.	37125000	2807	nofs	nofs
chr12	C	12q12	.	37375000	1403	nofs	nofs
chr12	C	12q12	.	37625000	2069	nofs	nofs
chr12	C	12q12	.	37875000	1978	nofs	nofs
chr12	C	12q12	.	38125000	3201	nofs	nofs
chr12	C	12q12	.	38375000	5539	nofs	nofs
chr12	C	12q12	.	38625000	2897	nofs	nofs
chr12	C	12q12	.	38875000	2932	nofs	nofs
chr12	C	12q12	.	39125000	11980	nofs	nofs
chr12	B	12q12	.	39375000	2415	nofs	nofs
chr12	B	12q12	.	39625000	3824	nofs	nofs
chr12	B	12q12	.	39875000	1970	nofs	nofs
chr12	B	12q12	.	40125000	1869	nofs	nofs
chr12	B	12q12	.	40375000	1940	nofs	nofs
chr12	B	12q12	.	40625000	2041	nofs	nofs
chr12	B	12q12	.	40875000	4560	nofs	nofs
chr12	B	12q12	.	41125000	3785	nofs	nofs
chr12	B	12q12	.	41375000	2681	nofs	nofs
chr12	B	12q12	.	41625000	1437	nofs	nofs
chr12	B	12q12	.	41875000	2057	nofs	nofs
chr12	B	12q12	.	42125000	1666	nofs	nofs
chr12	B	12q12	.	42375000	3538	nofs	nofs
chr12	B	12q12	.	42625000	2463	nofs	nofs
chr12	B	12q12	.	42875000	3047	nofs	nofs
chr12	B	12q12	.	43125000	2786	nofs	nofs
chr12	B	12q12	.	43375000	1845	nofs	nofs
chr12	B	12q12	.	43625000	2361	nofs	nofs
chr12	B	12q12	.	43875000	5675	nofs	nofs
chr12	B	12q12	.	44125000	1996	nofs	nofs

chr12	B	12q12	.	44375000	3492	nofs	nofs
chr12	E	12q13.1	1	44625000	2899	fs	fs rare
chr12	E	12q13.1	1	44875000	2758	fs	fs rare
chr12	E	12q13.1	1	45125000	1740	fs	fs rare
chr12	E	12q13.1	1	45375000	1743	fs	fs rare
chr12	E	12q13.1	1	45625000	2401	fs	fs rare
chr12	E	12q13.1	1	45875000	2471	fs	fs rare
chr12	E	12q13.1	1	46125000	2095	fs	fs rare
chr12	E	12q13.1	1	46375000	3777	fs	fs rare
chr12	E	12q13.1	1	46625000	4466	fs	fs rare
chr12	E	12q13.1	1	46875000	2921	fs	fs rare
chr12	E	12q13.1	1	47125000	4213	fs	fs rare
chr12	E	12q13.1	1	47375000	4883	fs	fs rare
chr12	E	12q13.1	1	47625000	5150	fs	fs rare
chr12	E	12q13.1	1	47875000	7198	fs	fs rare
chr12	E	12q13.1	1	48125000	5521	fs	fs rare
chr12	E	12q13.1	1	48375000	2095	fs	fs rare
chr12	E	12q13.1	1	48625000	5676	fs	fs rare
chr12	E	12q13.1	1	48875000	5534	fs	fs rare
chr12	E	12q13.1	1	49125000	6847	fs	fs rare
chr12	E	12q13.1	1	49375000	2255	fs	fs rare
chr12	E	12q13.1	1	49625000	4728	fs	fs rare
chr12	E	12q13.1	1	49875000	4509	fs	fs rare
chr12	E	12q13.1	1	50125000	2609	fs	fs rare
chr12	E	12q13.1	1	50375000	2112	fs	fs rare
chr12	E	12q13.1	1	50625000	1804	fs	fs rare
chr12	E	12q13.1	1	50875000	8651	fs	fs rare
chr12	E	12q13.1	1	51125000	2572	fs	fs rare
chr12	E	12q13.1	1	51375000	3145	fs	fs rare
chr12	E	12q13.1	1	51625000	5610	fs	fs rare
chr12	E	12q13.1	1	51875000	4116	fs	fs rare
chr12	E	12q13.1	1	52125000	5661	fs	fs rare
chr12	E	12q13.1	1	52375000	5345	fs	fs rare
chr12	E	12q13.1	1	52625000	3000	fs	fs rare
chr12	E	12q13.1	1	52875000	2377	fs	fs rare
chr12	E	12q13.1	1	53125000	4442	fs	fs rare

chr12	E	12q13.2	4	53375000	2453	nofs	nofs
chr12	E	12q13.2	4	53625000	1244	nofs	nofs
chr12	E	12q13.2	4	53875000	2329	nofs	nofs
chr12	E	12q13.2	4	54125000	3010	nofs	nofs
chr12	E	12q13.2	4	54375000	3528	nofs	nofs
chr12	E	12q13.2	4	54625000	3566	nofs	nofs
chr12	E	12q13.2	4	54875000	4475	nofs	nofs
chr12	E	12q13.3	3	55125000	6320	nofs	nofs
chr12	E	12q13.3	3	55375000	8698	nofs	nofs
chr12	E	12q13.3	3	55625000	3822	nofs	nofs
chr12	E	12q13.3	3	55875000	4687	nofs	nofs
chr12	E	12q13.3	3	56125000	6644	nofs	nofs
chr12	E	12q13.3	3	56375000	2921	nofs	nofs
chr12	E	12q14	1	56625000	3673	nofs	nofs
chr12	E	12q14	1	56875000	2185	nofs	nofs
chr12	E	12q14	1	57125000	2331	nofs	nofs
chr12	E	12q14	1	57375000	1986	nofs	nofs
chr12	E	12q14	1	57625000	2477	nofs	nofs
chr12	E	12q14	1	57875000	1894	nofs	nofs
chr12	E	12q14	1	58125000	20554	nofs	nofs
chr12	E	12q14	1	58375000	2193	nofs	nofs
chr12	E	12q14	1	58625000	2851	nofs	nofs
chr12	E	12q14	1	58875000	2057	nofs	nofs
chr12	E	12q14	1	59125000	2439	nofs	nofs
chr12	E	12q14	1	59375000	3118	nofs	nofs
chr12	E	12q14	1	59625000	3433	nofs	nofs
chr12	E	12q14	1	59875000	1927	nofs	nofs
chr12	E	12q14	1	60125000	1315	nofs	nofs
chr12	E	12q14	1	60375000	2044	nofs	nofs
chr12	E	12q14	1	60625000	2924	nofs	nofs
chr12	E	12q14	1	60875000	1886	nofs	nofs
chr12	E	12q14	1	61125000	1925	nofs	nofs
chr12	E	12q14	1	61375000	1766	nofs	nofs
chr12	E	12q14	1	61625000	1893	nofs	nofs
chr12	E	12q14	1	61875000	2145	nofs	nofs
chr12	E	12q14	1	62125000	3216	nofs	nofs

chr12	E	12q14	1	62375000	2853	nofs	nofs
chr12	E	12q14	1	62625000	2871	nofs	nofs
chr12	E	12q14	1	62875000	2908	nofs	nofs
chr12	E	12q14	1	63125000	4147	nofs	nofs
chr12	E	12q14	1	63375000	3799	nofs	nofs
chr12	E	12q14	1	63625000	1616	nofs	nofs
chr12	E	12q14	1	63875000	2446	nofs	nofs
chr12	E	12q14	1	64125000	1170	nofs	nofs
chr12	E	12q14	1	64375000	2324	nofs	nofs
chr12	E	12q14	1	64625000	4471	nofs	nofs
chr12	E	12q14	1	64875000	4776	nofs	nofs
chr12	E	12q14	1	65125000	1323	nofs	nofs
chr12	E	12q14	1	65375000	1857	nofs	nofs
chr12	E	12q14	1	65625000	1417	nofs	nofs
chr12	E	12q14	1	65875000	2063	nofs	nofs
chr12	E	12q14	1	66125000	1972	nofs	nofs
chr12	B	12q15	.	66375000	2086	nofs	nofs
chr12	B	12q15	.	66625000	1378	nofs	nofs
chr12	B	12q15	.	66875000	1642	nofs	nofs
chr12	B	12q15	.	67125000	2581	nofs	nofs
chr12	B	12q15	.	67375000	2653	nofs	nofs
chr12	B	12q15	.	67625000	2748	nofs	nofs
chr12	B	12q15	.	67875000	4852	nofs	nofs
chr12	B	12q15	.	68125000	3224	nofs	nofs
chr12	B	12q15	.	68375000	3406	nofs	nofs
chr12	B	12q15	.	68625000	3417	nofs	nofs
chr12	B	12q15	.	68875000	4419	nofs	nofs
chr12	B	12q15	.	69125000	1734	nofs	nofs
chr12	B	12q15	.	69375000	2584	nofs	nofs
chr12	B	12q15	.	69625000	8038	nofs	nofs
chr12	B	12q15	.	69875000	2315	nofs	nofs
chr12	E	12q21.1	1	70125000	3111	nofs	nofs
chr12	E	12q21.1	1	70375000	2833	nofs	nofs
chr12	E	12q21.1	1	70625000	1583	nofs	nofs
chr12	E	12q21.1	1	70875000	2551	nofs	nofs
chr12	E	12q21.1	1	71125000	1784	nofs	nofs

chr12	E	12q21.1	1	71375000	2229	nofs	nofs
chr12	E	12q21.1	1	71625000	2511	nofs	nofs
chr12	E	12q21.1	1	71875000	2321	nofs	nofs
chr12	E	12q21.1	1	72125000	2282	nofs	nofs
chr12	E	12q21.1	1	72375000	3749	nofs	nofs
chr12	E	12q21.1	1	72625000	3300	nofs	nofs
chr12	E	12q21.1	1	72875000	2151	nofs	nofs
chr12	E	12q21.1	1	73125000	1829	nofs	nofs
chr12	E	12q21.1	1	73375000	4553	nofs	nofs
chr12	E	12q21.1	1	73625000	2680	nofs	nofs
chr12	E	12q21.1	1	73875000	3237	nofs	nofs
chr12	E	12q21.1	1	74125000	1764	nofs	nofs
chr12	E	12q21.1	1	74375000	1660	nofs	nofs
chr12	E	12q21.2	1	74625000	3392	nofs	nofs
chr12	E	12q21.2	1	74875000	95	nofs	nofs
chr12	E	12q21.2	1	75125000	2879	nofs	nofs
chr12	E	12q21.2	1	75375000	1706	nofs	nofs
chr12	E	12q21.2	1	75625000	3120	nofs	nofs
chr12	E	12q21.2	1	75875000	2203	nofs	nofs
chr12	E	12q21.2	1	76125000	2118	nofs	nofs
chr12	E	12q21.2	1	76375000	1973	nofs	nofs
chr12	E	12q21.2	1	76625000	2006	nofs	nofs
chr12	E	12q21.2	1	76875000	2520	nofs	nofs
chr12	E	12q21.2	1	77125000	2791	nofs	nofs
chr12	E	12q21.2	1	77375000	2024	nofs	nofs
chr12	E	12q21.2	1	77625000	4367	nofs	nofs
chr12	E	12q21.2	1	77875000	2658	nofs	nofs
chr12	E	12q21.2	1	78125000	3700	nofs	nofs
chr12	E	12q21.2	1	78375000	22994	nofs	nofs
chr12	E	12q21.2	1	78625000	2374	nofs	nofs
chr12	E	12q21.2	1	78875000	2614	nofs	nofs
chr12	B	12q21.3	.	79125000	3515	fs	fs common
chr12	B	12q21.3	.	79375000	2570	fs	fs common
chr12	B	12q21.3	.	79625000	2513	fs	fs common
chr12	B	12q21.3	.	79875000	2589	fs	fs common
chr12	B	12q21.3	.	80125000	2298	fs	fs common

chr12	B	12q21.3	.	80375000	2919	fs	fs common
chr12	B	12q21.3	.	80625000	2561	fs	fs common
chr12	B	12q21.3	.	80875000	3799	fs	fs common
chr12	B	12q21.3	.	81125000	1863	fs	fs common
chr12	B	12q21.3	.	81375000	2768	fs	fs common
chr12	B	12q21.3	.	81625000	5838	fs	fs common
chr12	B	12q21.3	.	81875000	4025	fs	fs common
chr12	B	12q21.3	.	82125000	2564	fs	fs common
chr12	B	12q21.3	.	82375000	2180	fs	fs common
chr12	B	12q21.3	.	82625000	2703	fs	fs common
chr12	B	12q21.3	.	82875000	2612	fs	fs common
chr12	B	12q21.3	.	83125000	2705	fs	fs common
chr12	B	12q21.3	.	83375000	1560	fs	fs common
chr12	B	12q21.3	.	83625000	3459	fs	fs common
chr12	B	12q21.3	.	83875000	2068	fs	fs common
chr12	B	12q21.3	.	84125000	3605	fs	fs common
chr12	B	12q21.3	.	84375000	1976	fs	fs common
chr12	B	12q21.3	.	84625000	1897	fs	fs common
chr12	B	12q21.3	.	84875000	3269	fs	fs common
chr12	B	12q21.3	.	85125000	3092	fs	fs common
chr12	B	12q21.3	.	85375000	4638	fs	fs common
chr12	B	12q21.3	.	85625000	2854	fs	fs common
chr12	B	12q21.3	.	85875000	3201	fs	fs common
chr12	B	12q21.3	.	86125000	2115	fs	fs common
chr12	B	12q21.3	.	86375000	3240	fs	fs common
chr12	B	12q21.3	.	86625000	1551	fs	fs common
chr12	B	12q21.3	.	86875000	1946	fs	fs common
chr12	B	12q21.3	.	87125000	2451	fs	fs common
chr12	B	12q21.3	.	87375000	2393	fs	fs common
chr12	B	12q21.3	.	87625000	6672	fs	fs common
chr12	B	12q21.3	.	87875000	2634	fs	fs common
chr12	B	12q21.3	.	88125000	2413	fs	fs common
chr12	B	12q21.3	.	88375000	2296	fs	fs common
chr12	B	12q21.3	.	88625000	1969	fs	fs common
chr12	B	12q21.3	.	88875000	5326	fs	fs common
chr12	B	12q21.3	.	89125000	2336	fs	fs common

chr12	B	12q21.3	.	89375000	3612	fs	fs common
chr12	B	12q21.3	.	89625000	3808	fs	fs common
chr12	B	12q21.3	.	89875000	2138	fs	fs common
chr12	B	12q21.3	.	90125000	2439	fs	fs common
chr12	B	12q21.3	.	90375000	1825	fs	fs common
chr12	B	12q21.3	.	90625000	3297	fs	fs common
chr12	B	12q21.3	.	90875000	2086	fs	fs common
chr12	B	12q21.3	.	91125000	3031	fs	fs common
chr12	B	12q21.3	.	91375000	3133	fs	fs common
chr12	E	12q22	1	91625000	3667	nofs	nofs
chr12	E	12q22	1	91875000	3034	nofs	nofs
chr12	E	12q22	1	92125000	3206	nofs	nofs
chr12	E	12q22	1	92375000	3243	nofs	nofs
chr12	E	12q22	1	92625000	1678	nofs	nofs
chr12	E	12q22	1	92875000	2689	nofs	nofs
chr12	E	12q22	1	93125000	2396	nofs	nofs
chr12	E	12q22	1	93375000	2641	nofs	nofs
chr12	E	12q22	1	93625000	2160	nofs	nofs
chr12	E	12q22	1	93875000	2339	nofs	nofs
chr12	E	12q22	1	94125000	5747	nofs	nofs
chr12	E	12q22	1	94375000	3701	nofs	nofs
chr12	E	12q22	1	94625000	6712	nofs	nofs
chr12	E	12q22	1	94875000	4998	nofs	nofs
chr12	E	12q23	5	95125000	3740	nofs	nofs
chr12	E	12q23	5	95375000	3342	nofs	nofs
chr12	E	12q23	5	95625000	4176	nofs	nofs
chr12	E	12q23	5	95875000	2416	nofs	nofs
chr12	E	12q23	5	96125000	2466	nofs	nofs
chr12	E	12q23	5	96375000	2582	nofs	nofs
chr12	E	12q23	5	96625000	2546	nofs	nofs
chr12	E	12q23	5	96875000	4998	nofs	nofs
chr12	E	12q23	5	97125000	1672	nofs	nofs
chr12	E	12q23	5	97375000	2560	nofs	nofs
chr12	E	12q23	5	97625000	2082	nofs	nofs
chr12	E	12q23	5	97875000	2609	nofs	nofs
chr12	E	12q23	5	98125000	2333	nofs	nofs

chr12	E	12q23	5	98375000	1874	nofs	nofs
chr12	E	12q23	5	98625000	1605	nofs	nofs
chr12	E	12q23	5	98875000	1642	nofs	nofs
chr12	E	12q23	5	99125000	4528	nofs	nofs
chr12	E	12q23	5	99375000	2514	nofs	nofs
chr12	E	12q23	5	99625000	2046	nofs	nofs
chr12	E	12q23	5	99875000	2215	nofs	nofs
chr12	E	12q23	5	100000000	2579	nofs	nofs
chr12	E	12q23	5	100000000	3871	nofs	nofs
chr12	E	12q23	5	101000000	1558	nofs	nofs
chr12	E	12q23	5	101000000	4475	nofs	nofs
chr12	E	12q23	5	101000000	1279	nofs	nofs
chr12	E	12q23	5	101000000	1864	nofs	nofs
chr12	E	12q23	5	102000000	3137	nofs	nofs
chr12	E	12q23	5	102000000	3407	nofs	nofs
chr12	E	12q23	5	102000000	2988	nofs	nofs
chr12	E	12q23	5	102000000	2024	nofs	nofs
chr12	E	12q23	5	103000000	3278	nofs	nofs
chr12	E	12q23	5	103000000	4354	nofs	nofs
chr12	E	12q23	5	103000000	2571	nofs	nofs
chr12	E	12q23	5	103000000	3456	nofs	nofs
chr12	E	12q23	5	104000000	2487	nofs	nofs
chr12	E	12q23	5	104000000	2908	nofs	nofs
chr12	E	12q23	5	104000000	2281	nofs	nofs
chr12	E	12q23	5	104000000	3010	nofs	nofs
chr12	E	12q23	5	105000000	2595	nofs	nofs
chr12	E	12q23	5	105000000	2496	nofs	nofs
chr12	E	12q23	5	105000000	2828	nofs	nofs
chr12	E	12q23	5	105000000	1752	nofs	nofs
chr12	E	12q23	5	106000000	1833	nofs	nofs
chr12	E	12q23	5	106000000	3052	nofs	nofs
chr12	E	12q23	5	106000000	3432	nofs	nofs
chr12	E	12q23	5	106000000	3814	nofs	nofs
chr12	E	12q23	5	107000000	2501	nofs	nofs
chr12	E	12q23	5	107000000	7079	nofs	nofs
chr12	E	12q23	5	107000000	3877	nofs	nofs

chr12	E	12q23	5	107000000	5099	nofs	nofs
chr12	E	12q23	5	108000000	5602	nofs	nofs
chr12	E	12q24.1	5	108000000	3155	fs	fs common and rare
chr12	E	12q24.1	5	108000000	3961	fs	fs common and rare
chr12	E	12q24.1	5	108000000	2679	fs	fs common and rare
chr12	E	12q24.1	5	109000000	7182	fs	fs common and rare
chr12	E	12q24.1	5	109000000	3470	fs	fs common and rare
chr12	E	12q24.1	5	109000000	6440	fs	fs common and rare
chr12	E	12q24.1	5	109000000	4837	fs	fs common and rare
chr12	E	12q24.1	5	110000000	5873	fs	fs common and rare
chr12	E	12q24.1	5	110000000	6335	fs	fs common and rare
chr12	E	12q24.1	5	110000000	5472	fs	fs common and rare
chr12	E	12q24.1	5	110000000	2980	fs	fs common and rare
chr12	E	12q24.1	5	111000000	6993	fs	fs common and rare
chr12	E	12q24.1	5	111000000	5015	fs	fs common and rare
chr12	E	12q24.1	5	111000000	5293	fs	fs common and rare
chr12	E	12q24.1	5	111000000	7373	fs	fs common and rare
chr12	E	12q24.1	5	112000000	3629	fs	fs common and rare
chr12	E	12q24.1	5	112000000	4306	fs	fs common and rare
chr12	E	12q24.1	5	112000000	4189	fs	fs common and rare
chr12	E	12q24.1	5	112000000	8996	fs	fs common and rare
chr12	E	12q24.1	5	113000000	5403	fs	fs common and rare
chr12	E	12q24.1	5	113000000	5473	fs	fs common and rare
chr12	B	12q24.2	.	113000000	3235	fs	fs common and rare
chr12	B	12q24.2	.	113000000	6182	fs	fs common and rare
chr12	B	12q24.2	.	114000000	7220	fs	fs common and rare
chr12	B	12q24.2	.	114000000	3056	fs	fs common and rare
chr12	B	12q24.2	.	114000000	4573	fs	fs common and rare
chr12	B	12q24.2	.	114000000	3032	fs	fs common and rare
chr12	B	12q24.2	.	115000000	3254	fs	fs common and rare
chr12	B	12q24.2	.	115000000	2515	fs	fs common and rare
chr12	B	12q24.2	.	115000000	2876	fs	fs common and rare
chr12	B	12q24.2	.	115000000	5124	fs	fs common and rare
chr12	B	12q24.2	.	116000000	5113	fs	fs common and rare
chr12	B	12q24.2	.	116000000	2089	fs	fs common and rare
chr12	B	12q24.2	.	116000000	7010	fs	fs common and rare

chr12	B	12q24.2	.	116000000	5656	fs	fs common and rare
chr12	B	12q24.2	.	117000000	6737	fs	fs common and rare
chr12	B	12q24.2	.	117000000	4830	fs	fs common and rare
chr12	B	12q24.2	.	117000000	5263	fs	fs common and rare
chr12	B	12q24.2	.	117000000	4252	fs	fs common and rare
chr12	B	12q24.2	.	118000000	4926	fs	fs common and rare
chr12	B	12q24.2	.	118000000	2920	fs	fs common and rare
chr12	B	12q24.2	.	118000000	4518	fs	fs common and rare
chr12	B	12q24.2	.	118000000	5293	fs	fs common and rare
chr12	B	12q24.2	.	119000000	3039	fs	fs common and rare
chr12	B	12q24.2	.	119000000	4965	fs	fs common and rare
chr12	B	12q24.2	.	119000000	6326	fs	fs common and rare
chr12	E	12q24.31	3	119000000	5589	fs	fs common
chr12	E	12q24.31	3	120000000	3685	fs	fs common
chr12	E	12q24.31	3	120000000	9909	fs	fs common
chr12	E	12q24.31	3	120000000	4450	fs	fs common
chr12	E	12q24.31	3	120000000	5722	fs	fs common
chr12	E	12q24.31	3	121000000	7658	fs	fs common
chr12	E	12q24.31	3	121000000	6060	fs	fs common
chr12	E	12q24.31	3	121000000	3824	fs	fs common
chr12	E	12q24.31	3	121000000	4260	fs	fs common
chr12	E	12q24.31	3	122000000	5683	fs	fs common
chr12	E	12q24.31	3	122000000	4978	fs	fs common
chr12	E	12q24.31	3	122000000	2195	fs	fs common
chr12	E	12q24.31	3	122000000	7545	fs	fs common
chr12	E	12q24.31	3	123000000	6653	fs	fs common
chr12	E	12q24.31	3	123000000	5803	fs	fs common
chr12	E	12q24.31	3	123000000	5429	fs	fs common
chr12	E	12q24.31	3	123000000	12654	fs	fs common
chr12	E	12q24.31	3	124000000	7374	fs	fs common
chr12	E	12q24.31	3	124000000	6701	fs	fs common
chr12	E	12q24.31	3	124000000	7612	fs	fs common
chr12	E	12q24.31	3	124000000	4433	fs	fs common
chr12	E	12q24.31	3	125000000	6957	fs	fs common
chr12	E	12q24.31	3	125000000	3722	fs	fs common
chr12	E	12q24.31	3	125000000	3573	fs	fs common

chr12	E	12q24.31	3	125000000	4522	fs	fs common
chr12	B	12q24.32	.	126000000	5793	fs	fs common
chr12	B	12q24.32	.	126000000	5108	fs	fs common
chr12	B	12q24.32	.	126000000	5641	fs	fs common
chr12	B	12q24.32	.	126000000	5640	fs	fs common
chr12	B	12q24.32	.	127000000	6882	fs	fs common
chr12	B	12q24.32	.	127000000	3307	fs	fs common
chr12	B	12q24.32	.	127000000	10533	fs	fs common
chr12	B	12q24.32	.	127000000	6703	fs	fs common
chr12	B	12q24.32	.	128000000	12591	fs	fs common
chr12	B	12q24.32	.	128000000	8075	fs	fs common
chr12	B	12q24.32	.	128000000	10217	fs	fs common
chr12	B	12q24.32	.	128000000	9407	fs	fs common
chr12	B	12q24.33	.	129000000	6337	fs	fs common
chr12	B	12q24.33	.	129000000	9706	fs	fs common
chr12	T	12q24.33	.	129000000	8387	fs	fs common
chr12	T	12q24.33	.	129000000	11494	fs	fs common
chr12	T	12q24.33	.	130000000	11108	fs	fs common
chr12	T	12q24.33	.	130000000	12687	fs	fs common
chr12	T	12q24.33	.	130000000	15267	fs	fs common
chr12	T	12q24.33	.	130000000	21202	fs	fs common
chr12	T	12q24.33	.	131000000	10301	fs	fs common
chr12	T	12q24.33	.	131000000	16140	fs	fs common
chr12	T	12q24.33	.	131000000	12218	fs	fs common
chr12	T	12q24.33	.	131000000	38463	fs	fs common
chr12	T	12q24.33	.	132000000	41097	fs	fs common
chr12	T	12q24.33	.	132000000	25187	fs	fs common
chr12	T	12q24.33	.	132000000	18340	fs	fs common
chr13	C	13p13	.	125000	0	nofs	nofs
chr13	C	13p13	.	375000	0	nofs	nofs
chr13	C	13p13	.	625000	0	nofs	nofs
chr13	C	13p13	.	875000	0	nofs	nofs
chr13	C	13p13	.	1125000	0	nofs	nofs
chr13	C	13p13	.	1375000	0	nofs	nofs
chr13	C	13p13	.	1625000	0	nofs	nofs
chr13	C	13p13	.	1875000	0	nofs	nofs

chr13	C	13p13	.	2125000	0	nofs	nofs
chr13	C	13p13	.	2375000	0	nofs	nofs
chr13	C	13p13	.	2625000	0	nofs	nofs
chr13	C	13p13	.	2875000	0	nofs	nofs
chr13	C	13p13	.	3125000	0	nofs	nofs
chr13	C	13p13	.	3375000	0	nofs	nofs
chr13	C	13p13	.	3625000	0	nofs	nofs
chr13	C	13p13	.	3875000	0	nofs	nofs
chr13	C	13p12	.	4125000	0	nofs	nofs
chr13	C	13p12	.	4375000	0	nofs	nofs
chr13	C	13p12	.	4625000	0	nofs	nofs
chr13	C	13p12	.	4875000	0	nofs	nofs
chr13	C	13p12	.	5125000	0	nofs	nofs
chr13	C	13p12	.	5375000	0	nofs	nofs
chr13	C	13p12	.	5625000	0	nofs	nofs
chr13	C	13p12	.	5875000	0	nofs	nofs
chr13	C	13p12	.	6125000	0	nofs	nofs
chr13	C	13p12	.	6375000	0	nofs	nofs
chr13	C	13p12	.	6625000	0	nofs	nofs
chr13	C	13p12	.	6875000	0	nofs	nofs
chr13	C	13p12	.	7125000	0	nofs	nofs
chr13	C	13p12	.	7375000	0	nofs	nofs
chr13	C	13p12	.	7625000	0	nofs	nofs
chr13	C	13p12	.	7875000	0	nofs	nofs
chr13	C	13p12	.	8125000	0	nofs	nofs
chr13	C	13p12	.	8375000	0	nofs	nofs
chr13	C	13p13	.	8625000	0	nofs	nofs
chr13	C	13p11	.	8875000	0	nofs	nofs
chr13	C	13p11	.	9125000	0	nofs	nofs
chr13	C	13p11	.	9375000	0	nofs	nofs
chr13	C	13p11	.	9625000	0	nofs	nofs
chr13	C	13p11	.	9875000	0	nofs	nofs
chr13	C	13p11	.	10125000	0	nofs	nofs
chr13	C	13p11	.	10375000	0	nofs	nofs
chr13	C	13p11	.	10625000	0	nofs	nofs
chr13	C	13p11	.	10875000	0	nofs	nofs

chr13	C	13p11	.	11125000	0	nofs	nofs
chr13	C	13p11	.	11375000	0	nofs	nofs
chr13	C	13p11	.	11625000	0	nofs	nofs
chr13	C	13p11	.	11875000	0	nofs	nofs
chr13	C	13p11	.	12125000	0	nofs	nofs
chr13	C	13p11	.	12375000	0	nofs	nofs
chr13	C	13p11	.	12625000	0	nofs	nofs
chr13	C	13p11	.	12875000	0	nofs	nofs
chr13	C	13p11	.	13125000	0	nofs	nofs
chr13	C	13p11	.	13375000	0	nofs	nofs
chr13	C	13p11	.	13625000	0	nofs	nofs
chr13	C	13p11	.	13875000	0	nofs	nofs
chr13	C	13p11	.	14125000	0	nofs	nofs
chr13	C	13p11	.	14375000	0	nofs	nofs
chr13	C	13p11	.	14625000	0	nofs	nofs
chr13	C	13p11	.	14875000	0	nofs	nofs
chr13	C	13p11	.	15125000	0	nofs	nofs
chr13	C	13p11	.	15375000	0	nofs	nofs
chr13	C	13p11	.	15625000	0	nofs	nofs
chr13	C	13p11	.	15875000	0	nofs	nofs
chr13	C	13p11	.	16125000	0	nofs	nofs
chr13	C		.	16375000	0	nofs	nofs
chr13	C		.	16625000	0	nofs	nofs
chr13	C		.	16875000	0	nofs	nofs
chr13	C		.	17125000	0	nofs	nofs
chr13	C		.	17375000	0	nofs	nofs
chr13	C		.	17625000	0	nofs	nofs
chr13	C	13q11	.	17875000	12608	nofs	nofs
chr13	C	13q11	.	18125000	3495	nofs	nofs
chr13	C	13q12.1	3	18375000	7832	nofs	nofs
chr13	C	13q12.1	3	18625000	5807	nofs	nofs
chr13	C	13q12.1	3	18875000	2151	nofs	nofs
chr13	C	13q12.1	3	19125000	2404	nofs	nofs
chr13	C	13q12.1	3	19375000	5815	nofs	nofs
chr13	C	13q12.1	3	19625000	2772	nofs	nofs
chr13	C	13q12.1	3	19875000	6956	nofs	nofs

chr13	C	13q12.1	3	20125000	4047	nofs	nofs
chr13	C	13q12.1	3	20375000	6047	nofs	nofs
chr13	C	13q12.1	3	20625000	5245	nofs	nofs
chr13	C	13q12.1	3	20875000	4899	nofs	nofs
chr13	E	13q12.1	3	21125000	1758	nofs	nofs
chr13	E	13q12.1	3	21375000	8056	nofs	nofs
chr13	E	13q12.1	3	21625000	1951	nofs	nofs
chr13	E	13q12.1	3	21875000	1782	nofs	nofs
chr13	E	13q12.1	3	22125000	3794	nofs	nofs
chr13	E	13q12.1	3	22375000	4708	nofs	nofs
chr13	E	13q12.1	3	22625000	5486	nofs	nofs
chr13	E	13q12.1	3	22875000	4816	nofs	nofs
chr13	E	13q12.1	3	23125000	3582	nofs	nofs
chr13	E	13q12.1	3	23375000	2354	nofs	nofs
chr13	E	13q12.1	3	23625000	2153	nofs	nofs
chr13	E	13q12.1	3	23875000	7279	nofs	nofs
chr13	E	13q12.1	3	24125000	4026	nofs	nofs
chr13	E	13q12.1	3	24375000	3213	nofs	nofs
chr13	E	13q12.1	3	24625000	3144	nofs	nofs
chr13	E	13q12.1	3	24875000	4355	nofs	nofs
chr13	E	13q12.1	3	25125000	2049	nofs	nofs
chr13	E	13q12.1	3	25375000	2370	nofs	nofs
chr13	E	13q12.1	3	25625000	2057	nofs	nofs
chr13	E	13q12.1	3	25875000	2738	nofs	nofs
chr13	E	13q12.1	3	26125000	2157	nofs	nofs
chr13	E	13q12.1	3	26375000	3750	nofs	nofs
chr13	E	13q12.1	3	26625000	4078	nofs	nofs
chr13	B	13q12.2	.	26875000	5091	nofs	nofs
chr13	B	13q12.2	.	27125000	2590	nofs	nofs
chr13	B	13q12.2	.	27375000	5152	nofs	nofs
chr13	B	13q12.2	.	27625000	2850	nofs	nofs
chr13	B	13q12.2	.	27875000	2754	nofs	nofs
chr13	B	13q12.3	.	28125000	2275	nofs	nofs
chr13	B	13q12.3	.	28375000	2512	nofs	nofs
chr13	B	13q12.3	.	28625000	1393	nofs	nofs
chr13	B	13q12.3	.	28875000	3694	nofs	nofs

chr13	B	13q12.3	.	29125000	3779	nofs	nofs
chr13	B	13q12.3	.	29375000	2906	nofs	nofs
chr13	B	13q12.3	.	29625000	2463	nofs	nofs
chr13	B	13q12.3	.	29875000	3131	nofs	nofs
chr13	B	13q12.3	.	30125000	2786	nofs	nofs
chr13	B	13q12.3	.	30375000	6235	nofs	nofs
chr13	B	13q12.3	.	30625000	3191	nofs	nofs
chr13	B	13q12.3	.	30875000	2942	nofs	nofs
chr13	E	13q13	2	31125000	2817	nofs	nofs
chr13	E	13q13	2	31375000	2619	nofs	nofs
chr13	E	13q13	2	31625000	2182	nofs	nofs
chr13	E	13q13	2	31875000	1787	nofs	nofs
chr13	E	13q13	2	32125000	4199	nofs	nofs
chr13	E	13q13	2	32375000	2793	nofs	nofs
chr13	E	13q13	2	32625000	2481	nofs	nofs
chr13	E	13q13	2	32875000	2595	fs	fs common and rare
chr13	E	13q13	2	33125000	2950	fs	fs common and rare
chr13	E	13q13	2	33375000	2628	fs	fs common and rare
chr13	E	13q13	2	33625000	2519	fs	fs common and rare
chr13	E	13q13	2	33875000	2839	fs	fs common and rare
chr13	E	13q13	2	34125000	3718	fs	fs common and rare
chr13	E	13q13	2	34375000	3093	fs	fs common and rare
chr13	E	13q13	2	34625000	2119	fs	fs common and rare
chr13	E	13q13	2	34875000	1816	nofs	nofs
chr13	E	13q13	2	35125000	4995	nofs	nofs
chr13	E	13q13	2	35375000	2450	nofs	nofs
chr13	E	13q13	2	35625000	1173	nofs	nofs
chr13	E	13q13	2	35875000	3257	nofs	nofs
chr13	E	13q13	2	36125000	1560	nofs	nofs
chr13	E	13q13	2	36375000	1391	nofs	nofs
chr13	E	13q13	2	36625000	1951	nofs	nofs
chr13	E	13q13	2	36875000	2962	nofs	nofs
chr13	E	13q13	2	37125000	1758	nofs	nofs
chr13	E	13q13	2	37375000	2138	nofs	nofs
chr13	E	13q13	2	37625000	2844	nofs	nofs
chr13	E	13q13	2	37875000	2120	nofs	nofs

chr13	E	13q13	2	38125000	3144	nofs	nofs
chr13	E	13q13	2	38375000	3088	nofs	nofs
chr13	E	13q13	2	38625000	2035	nofs	nofs
chr13	E	13q13	2	38875000	3175	nofs	nofs
chr13	E	13q13	2	39125000	2065	nofs	nofs
chr13	E	13q14.1	5	39375000	2206	nofs	nofs
chr13	E	13q14.1	5	39625000	2598	nofs	nofs
chr13	E	13q14.1	5	39875000	2866	nofs	nofs
chr13	E	13q14.1	5	40125000	2655	nofs	nofs
chr13	E	13q14.1	5	40375000	4191	nofs	nofs
chr13	E	13q14.1	5	40625000	2189	nofs	nofs
chr13	E	13q14.1	5	40875000	11366	nofs	nofs
chr13	E	13q14.1	5	41125000	2630	nofs	nofs
chr13	E	13q14.1	5	41375000	3715	nofs	nofs
chr13	E	13q14.1	5	41625000	3103	nofs	nofs
chr13	E	13q14.1	5	41875000	2063	nofs	nofs
chr13	E	13q14.1	5	42125000	2167	nofs	nofs
chr13	E	13q14.1	5	42375000	2430	nofs	nofs
chr13	E	13q14.1	5	42625000	898	nofs	nofs
chr13	E	13q14.1	5	42875000	2561	nofs	nofs
chr13	E	13q14.1	5	43125000	1842	nofs	nofs
chr13	E	13q14.1	5	43375000	2012	nofs	nofs
chr13	E	13q14.1	5	43625000	2610	nofs	nofs
chr13	E	13q14.1	5	43875000	2480	nofs	nofs
chr13	E	13q14.1	5	44125000	3610	nofs	nofs
chr13	E	13q14.1	5	44375000	2427	nofs	nofs
chr13	E	13q14.1	5	44625000	3363	nofs	nofs
chr13	E	13q14.1	5	44875000	10770	nofs	nofs
chr13	E	13q14.1	5	45125000	2021	nofs	nofs
chr13	E	13q14.1	5	45375000	2602	nofs	nofs
chr13	E	13q14.1	5	45625000	2230	nofs	nofs
chr13	E	13q14.1	5	45875000	4782	nofs	nofs
chr13	E	13q14.1	5	46125000	2648	nofs	nofs
chr13	E	13q14.2	2	46375000	1336	nofs	nofs
chr13	E	13q14.2	2	46625000	3087	nofs	nofs
chr13	E	13q14.2	2	46875000	1550	nofs	nofs

chr13	E	13q14.2	2	47125000	1120	nofs	nofs
chr13	E	13q14.2	2	47375000	1779	nofs	nofs
chr13	E	13q14.2	2	47625000	2046	nofs	nofs
chr13	E	13q14.2	2	47875000	3000	nofs	nofs
chr13	E	13q14.2	2	48125000	2139	nofs	nofs
chr13	E	13q14.2	2	48375000	2526	nofs	nofs
chr13	E	13q14.2	2	48625000	2067	nofs	nofs
chr13	E	13q14.2	2	48875000	4141	nofs	nofs
chr13	E	13q14.2	2	49125000	5317	nofs	nofs
chr13	E	13q14.2	2	49375000	5258	nofs	nofs
chr13	E	13q14.2	2	49625000	2411	nofs	nofs
chr13	E	13q14.2	2	49875000	1673	nofs	nofs
chr13	E	13q14.3	4	50125000	1905	nofs	nofs
chr13	E	13q14.3	4	50375000	1664	nofs	nofs
chr13	E	13q14.3	4	50625000	2886	nofs	nofs
chr13	E	13q14.3	4	50875000	2204	nofs	nofs
chr13	E	13q14.3	4	51125000	3286	nofs	nofs
chr13	E	13q14.3	4	51375000	1916	nofs	nofs
chr13	E	13q14.3	4	51625000	3306	nofs	nofs
chr13	E	13q14.3	4	51875000	2598	nofs	nofs
chr13	E	13q14.3	4	52125000	2294	nofs	nofs
chr13	E	13q14.3	4	52375000	2736	nofs	nofs
chr13	E	13q14.3	4	52625000	1708	nofs	nofs
chr13	E	13q14.3	4	52875000	3106	nofs	nofs
chr13	E	13q14.3	4	53125000	1626	nofs	nofs
chr13	E	13q14.3	4	53375000	2418	nofs	nofs
chr13	E	13q14.3	4	53625000	1579	nofs	nofs
chr13	E	13q14.3	4	53875000	3536	nofs	nofs
chr13	E	13q14.3	4	54125000	2057	nofs	nofs
chr13	E	13q14.3	4	54375000	3302	nofs	nofs
chr13	B	13q21	.	54625000	2803	fs	fs common
chr13	B	13q21	.	54875000	2800	fs	fs common
chr13	B	13q21	.	55125000	2547	fs	fs common
chr13	B	13q21	.	55375000	2152	fs	fs common
chr13	B	13q21	.	55625000	2936	fs	fs common
chr13	B	13q21	.	55875000	2185	fs	fs common

chr13	B	13q21	.	56125000	3277	fs	fs common
chr13	B	13q21	.	56375000	2069	fs	fs common
chr13	B	13q21	.	56625000	2536	fs	fs common
chr13	B	13q21	.	56875000	2993	fs	fs common
chr13	B	13q21	.	57125000	2608	fs	fs common
chr13	B	13q21	.	57375000	3114	fs	fs common
chr13	B	13q21	.	57625000	2317	fs	fs common
chr13	B	13q21	.	57875000	2715	fs	fs common
chr13	B	13q21	.	58125000	3627	fs	fs common
chr13	B	13q21	.	58375000	3234	fs	fs common
chr13	B	13q21	.	58625000	1689	fs	fs common
chr13	B	13q21	.	58875000	2693	fs	fs common
chr13	B	13q21	.	59125000	1714	fs	fs common
chr13	B	13q21	.	59375000	1412	fs	fs common
chr13	B	13q21	.	59625000	2381	fs	fs common
chr13	B	13q21	.	59875000	2178	fs	fs common
chr13	B	13q21	.	60125000	1791	fs	fs common
chr13	B	13q21	.	60375000	2906	fs	fs common
chr13	B	13q21	.	60625000	13353	fs	fs common
chr13	B	13q21	.	60875000	2294	fs	fs common
chr13	B	13q21	.	61125000	2572	fs	fs common
chr13	B	13q21	.	61375000	6929	fs	fs common
chr13	B	13q21	.	61625000	3744	fs	fs common
chr13	B	13q21	.	61875000	2897	fs	fs common
chr13	B	13q21	.	62125000	3160	fs	fs common
chr13	B	13q21	.	62375000	2395	fs	fs common
chr13	B	13q21	.	62625000	3496	fs	fs common
chr13	B	13q21	.	62875000	3865	fs	fs common
chr13	B	13q21	.	63125000	6715	fs	fs common
chr13	B	13q21	.	63375000	6086	fs	fs common
chr13	B	13q21	.	63625000	2694	fs	fs common
chr13	B	13q21	.	63875000	2849	fs	fs common
chr13	B	13q21	.	64125000	2108	fs	fs common
chr13	B	13q21	.	64375000	2088	fs	fs common
chr13	B	13q21	.	64625000	1876	fs	fs common
chr13	B	13q21	.	64875000	3922	fs	fs common

chr13	B	13q21	.	65125000	1786	fs	fs common
chr13	B	13q21	.	65375000	2712	fs	fs common
chr13	B	13q21	.	65625000	2467	fs	fs common
chr13	B	13q21	.	65875000	2229	fs	fs common
chr13	B	13q21	.	66125000	2338	fs	fs common
chr13	B	13q21	.	66375000	2102	fs	fs common
chr13	B	13q21	.	66625000	2390	fs	fs common
chr13	B	13q21	.	66875000	1641	fs	fs common
chr13	B	13q21	.	67125000	4298	fs	fs common
chr13	B	13q21	.	67375000	3223	fs	fs common
chr13	B	13q21	.	67625000	2668	fs	fs common
chr13	B	13q21	.	67875000	1713	fs	fs common
chr13	B	13q21	.	68125000	2525	fs	fs common
chr13	B	13q21	.	68375000	2408	fs	fs common
chr13	B	13q21	.	68625000	2683	fs	fs common
chr13	B	13q21	.	68875000	995	fs	fs common
chr13	B	13q21	.	69125000	2338	fs	fs common
chr13	B	13q21	.	69375000	2458	fs	fs common
chr13	B	13q21	.	69625000	2356	fs	fs common
chr13	B	13q21	.	69875000	2191	fs	fs common
chr13	B	13q21	.	70125000	3065	fs	fs common
chr13	B	13q21	.	70375000	1953	fs	fs common
chr13	B	13q21	.	70625000	2892	fs	fs common
chr13	B	13q21	.	70875000	2531	fs	fs common
chr13	B	13q21	.	71125000	4332	fs	fs common
chr13	B	13q21	.	71375000	3259	fs	fs common
chr13	B	13q21	.	71625000	1087	fs	fs common
chr13	B	13q21	.	71875000	1307	fs	fs common
chr13	B	13q21	.	72125000	2013	fs	fs common
chr13	B	13q22	.	72375000	3003	nofs	nofs
chr13	B	13q22	.	72625000	4780	nofs	nofs
chr13	B	13q22	.	72875000	1713	nofs	nofs
chr13	B	13q22	.	73125000	2953	nofs	nofs
chr13	B	13q22	.	73375000	1856	nofs	nofs
chr13	B	13q22	.	73625000	1726	nofs	nofs
chr13	B	13q22	.	73875000	1525	nofs	nofs

chr13	B	13q22	.	74125000	2308	nofs	nofs
chr13	B	13q22	.	74375000	3258	nofs	nofs
chr13	B	13q22	.	74625000	1420	nofs	nofs
chr13	B	13q22	.	74875000	2387	nofs	nofs
chr13	B	13q22	.	75125000	2029	nofs	nofs
chr13	B	13q22	.	75375000	1437	nofs	nofs
chr13	B	13q22	.	75625000	2651	nofs	nofs
chr13	B	13q22	.	75875000	4382	nofs	nofs
chr13	B	13q22	.	76125000	1607	nofs	nofs
chr13	B	13q22	.	76375000	2346	nofs	nofs
chr13	B	13q22	.	76625000	2450	nofs	nofs
chr13	B	13q22	.	76875000	2897	nofs	nofs
chr13	B	13q22	.	77125000	4143	nofs	nofs
chr13	B	13q22	.	77375000	2431	nofs	nofs
chr13	B	13q22	.	77625000	2293	nofs	nofs
chr13	E	13q31	1	77875000	2253	nofs	nofs
chr13	E	13q31	1	78125000	2629	nofs	nofs
chr13	E	13q31	1	78375000	903	nofs	nofs
chr13	E	13q31	1	78625000	2379	nofs	nofs
chr13	E	13q31	1	78875000	3331	nofs	nofs
chr13	E	13q31	1	79125000	1616	nofs	nofs
chr13	E	13q31	1	79375000	1854	nofs	nofs
chr13	E	13q31	1	79625000	3062	nofs	nofs
chr13	E	13q31	1	79875000	4292	nofs	nofs
chr13	E	13q31	1	80125000	5236	nofs	nofs
chr13	E	13q31	1	80375000	2089	nofs	nofs
chr13	E	13q31	1	80625000	2015	nofs	nofs
chr13	E	13q31	1	80875000	2380	nofs	nofs
chr13	E	13q31	1	81125000	2002	nofs	nofs
chr13	E	13q31	1	81375000	2397	nofs	nofs
chr13	E	13q31	1	81625000	3622	nofs	nofs
chr13	E	13q31	1	81875000	2506	nofs	nofs
chr13	E	13q31	1	82125000	4328	nofs	nofs
chr13	E	13q31	1	82375000	3187	nofs	nofs
chr13	E	13q31	1	82625000	2369	nofs	nofs
chr13	E	13q31	1	82875000	2825	nofs	nofs

chr13	E	13q31	1	83125000	2471	nofs	nofs
chr13	E	13q31	1	83375000	1900	nofs	nofs
chr13	E	13q31	1	83625000	3806	nofs	nofs
chr13	E	13q31	1	83875000	2398	nofs	nofs
chr13	E	13q31	1	84125000	3595	nofs	nofs
chr13	E	13q31	1	84375000	2340	nofs	nofs
chr13	E	13q31	1	84625000	2312	nofs	nofs
chr13	E	13q31	1	84875000	3946	nofs	nofs
chr13	E	13q31	1	85125000	3153	nofs	nofs
chr13	E	13q31	1	85375000	2054	nofs	nofs
chr13	E	13q31	1	85625000	2814	nofs	nofs
chr13	E	13q31	1	85875000	3822	nofs	nofs
chr13	E	13q31	1	86125000	2900	nofs	nofs
chr13	E	13q31	1	86375000	2134	nofs	nofs
chr13	E	13q31	1	86625000	4204	nofs	nofs
chr13	E	13q31	1	86875000	2618	nofs	nofs
chr13	E	13q31	1	87125000	5375	nofs	nofs
chr13	E	13q31	1	87375000	3187	nofs	nofs
chr13	E	13q31	1	87625000	1995	nofs	nofs
chr13	E	13q31	1	87875000	3308	nofs	nofs
chr13	E	13q31	1	88125000	3052	nofs	nofs
chr13	E	13q31	1	88375000	3015	nofs	nofs
chr13	E	13q31	1	88625000	2984	nofs	nofs
chr13	E	13q31	1	88875000	3277	nofs	nofs
chr13	E	13q31	1	89125000	2787	nofs	nofs
chr13	E	13q31	1	89375000	2614	nofs	nofs
chr13	E	13q31	1	89625000	2339	nofs	nofs
chr13	E	13q31	1	89875000	2426	nofs	nofs
chr13	E	13q31	1	90125000	2126	nofs	nofs
chr13	E	13q31	1	90375000	2476	nofs	nofs
chr13	E	13q31	1	90625000	2345	nofs	nofs
chr13	E	13q31	1	90875000	2323	nofs	nofs
chr13	E	13q31	1	91125000	2816	nofs	nofs
chr13	E	13q31	1	91375000	3231	nofs	nofs
chr13	E	13q31	1	91625000	2608	nofs	nofs
chr13	E	13q31	1	91875000	2906	nofs	nofs

chr13	E	13q31	1	92125000	2830	nofs	nofs
chr13	E	13q31	1	92375000	2125	nofs	nofs
chr13	E	13q31	1	92625000	5156	nofs	nofs
chr13	E	13q31	1	92875000	2903	nofs	nofs
chr13	E	13q31	1	93125000	2064	nofs	nofs
chr13	E	13q31	1	93375000	3017	nofs	nofs
chr13	E	13q31	1	93625000	2766	nofs	nofs
chr13	E	13q31	1	93875000	2989	nofs	nofs
chr13	B	13q32	.	94125000	2110	fs	fs common
chr13	B	13q32	.	94375000	2294	fs	fs common
chr13	B	13q32	.	94625000	2944	fs	fs common
chr13	B	13q32	.	94875000	3764	fs	fs common
chr13	B	13q32	.	95125000	3729	fs	fs common
chr13	B	13q32	.	95375000	2390	fs	fs common
chr13	B	13q32	.	95625000	2541	fs	fs common
chr13	B	13q32	.	95875000	2225	fs	fs common
chr13	B	13q32	.	96125000	1938	fs	fs common
chr13	B	13q32	.	96375000	1801	fs	fs common
chr13	B	13q32	.	96625000	2271	fs	fs common
chr13	B	13q32	.	96875000	2691	fs	fs common
chr13	B	13q32	.	97125000	2006	fs	fs common
chr13	B	13q32	.	97375000	3160	fs	fs common
chr13	B	13q32	.	97625000	5183	fs	fs common
chr13	B	13q32	.	97875000	2956	fs	fs common
chr13	B	13q32	.	98125000	5815	fs	fs common
chr13	B	13q32	.	98375000	1934	fs	fs common
chr13	B	13q32	.	98625000	3119	fs	fs common
chr13	B	13q32	.	98875000	3795	fs	fs common
chr13	B	13q32	.	99125000	2591	fs	fs common
chr13	B	13q32	.	99375000	4227	fs	fs common
chr13	B	13q32	.	99625000	2384	fs	fs common
chr13	B	13q32	.	99875000	2856	fs	fs common
chr13	B	13q32	.	100000000	2438	fs	fs common
chr13	B	13q32	.	100000000	3488	fs	fs common
chr13	B	13q32	.	101000000	2108	fs	fs common
chr13	E	13q33	3	101000000	2780	nofs	nofs

chr13	E	13q33	3	101000000	2755	nofs	nofs
chr13	E	13q33	3	101000000	1760	nofs	nofs
chr13	E	13q33	3	102000000	3255	nofs	nofs
chr13	E	13q33	3	102000000	2055	nofs	nofs
chr13	E	13q33	3	102000000	3504	nofs	nofs
chr13	E	13q33	3	102000000	1843	nofs	nofs
chr13	E	13q33	3	103000000	1827	nofs	nofs
chr13	E	13q33	3	103000000	2686	nofs	nofs
chr13	E	13q33	3	103000000	2470	nofs	nofs
chr13	E	13q33	3	103000000	2555	nofs	nofs
chr13	E	13q33	3	104000000	3764	nofs	nofs
chr13	E	13q33	3	104000000	3451	nofs	nofs
chr13	E	13q33	3	104000000	5323	nofs	nofs
chr13	E	13q33	3	104000000	2269	nofs	nofs
chr13	E	13q33	3	105000000	1981	nofs	nofs
chr13	E	13q33	3	105000000	2125	nofs	nofs
chr13	E	13q33	3	105000000	4131	nofs	nofs
chr13	E	13q33	3	105000000	2791	nofs	nofs
chr13	E	13q33	3	106000000	2576	nofs	nofs
chr13	E	13q33	3	106000000	2141	nofs	nofs
chr13	E	13q33	3	106000000	2904	nofs	nofs
chr13	E	13q33	3	106000000	2829	nofs	nofs
chr13	E	13q33	3	107000000	2515	nofs	nofs
chr13	E	13q33	3	107000000	2746	nofs	nofs
chr13	E	13q33	3	107000000	2732	nofs	nofs
chr13	E	13q33	3	107000000	2637	nofs	nofs
chr13	E	13q33	3	108000000	4401	nofs	nofs
chr13	E	13q33	3	108000000	3228	nofs	nofs
chr13	E	13q33	3	108000000	3083	nofs	nofs
chr13	E	13q33	3	108000000	7107	nofs	nofs
chr13	E	13q33	3	109000000	6830	nofs	nofs
chr13	E	13q33	3	109000000	6992	nofs	nofs
chr13	E	13q33	3	109000000	6938	nofs	nofs
chr13	B	13q34	.	109000000	3373	nofs	nofs
chr13	B	13q34	.	110000000	5887	nofs	nofs
chr13	B	13q34	.	110000000	9584	nofs	nofs

chr13	B	13q34	.	110000000	5571	nofs	nofs
chr13	B	13q34	.	110000000	4360	nofs	nofs
chr13	B	13q34	.	111000000	5632	nofs	nofs
chr13	T	13q34	.	111000000	14866	nofs	nofs
chr13	T	13q34	.	111000000	10404	nofs	nofs
chr13	T	13q34	.	111000000	0	nofs	nofs
chr13	T	13q34	.	112000000	10789	nofs	nofs
chr13	T	13q34	.	112000000	38323	nofs	nofs
chr13	T	13q34	.	112000000	29516	nofs	nofs
chr13	T	13q34	.	112000000	19252	nofs	nofs
chr13	T	13q34	.	113000000	22420	nofs	nofs
chr13	T	13q34	.	113000000	21856	nofs	nofs
chr13	T	13q34	.	113000000	27454	nofs	nofs
chr13	T	13q34	.	113000000	15138	nofs	nofs
chr13	T	13q34	.	114000000	22258	nofs	nofs
chr13	T	13q34	.	114000000	43062	nofs	nofs
chr14	C	14p13	.	125000	0	nofs	nofs
chr14	C	14p13	.	375000	0	nofs	nofs
chr14	C	14p13	.	625000	0	nofs	nofs
chr14	C	14p13	.	875000	0	nofs	nofs
chr14	C	14p13	.	1125000	0	nofs	nofs
chr14	C	14p13	.	1375000	0	nofs	nofs
chr14	C	14p13	.	1625000	0	nofs	nofs
chr14	C	14p13	.	1875000	0	nofs	nofs
chr14	C	14p13	.	2125000	0	nofs	nofs
chr14	C	14p13	.	2375000	0	nofs	nofs
chr14	C	14p13	.	2625000	0	nofs	nofs
chr14	C	14p13	.	2875000	0	nofs	nofs
chr14	C	14p13	.	3125000	0	nofs	nofs
chr14	C	14p12	.	3375000	0	nofs	nofs
chr14	C	14p12	.	3625000	0	nofs	nofs
chr14	C	14p12	.	3875000	0	nofs	nofs
chr14	C	14p12	.	4125000	0	nofs	nofs
chr14	C	14p12	.	4375000	0	nofs	nofs
chr14	C	14p12	.	4625000	0	nofs	nofs
chr14	C	14p12	.	4875000	0	nofs	nofs

chr14	C	14p12	.	5125000	0	nofs	nofs
chr14	C	14p12	.	5375000	0	nofs	nofs
chr14	C	14p12	.	5625000	0	nofs	nofs
chr14	C	14p12	.	5875000	0	nofs	nofs
chr14	C	14p12	.	6125000	0	nofs	nofs
chr14	C	14p12	.	6375000	0	nofs	nofs
chr14	C	14p12	.	6625000	0	nofs	nofs
chr14	C	14p12	.	6875000	0	nofs	nofs
chr14	C	14p11	.	7125000	0	nofs	nofs
chr14	C	14p11	.	7375000	0	nofs	nofs
chr14	C	14p11	.	7625000	0	nofs	nofs
chr14	C	14p11	.	7875000	0	nofs	nofs
chr14	C	14p11	.	8125000	0	nofs	nofs
chr14	C	14p11	.	8375000	0	nofs	nofs
chr14	C	14p11	.	8625000	0	nofs	nofs
chr14	C	14p11	.	8875000	0	nofs	nofs
chr14	C	14p11	.	9125000	0	nofs	nofs
chr14	C	14p11	.	9375000	0	nofs	nofs
chr14	C	14p11	.	9625000	0	nofs	nofs
chr14	C	14p11	.	9875000	0	nofs	nofs
chr14	C	14p11	.	10125000	0	nofs	nofs
chr14	C	14p11	.	10375000	0	nofs	nofs
chr14	C	14p11	.	10625000	0	nofs	nofs
chr14	C	14p11	.	10875000	0	nofs	nofs
chr14	C	14p11	.	11125000	0	nofs	nofs
chr14	C	14p11	.	11375000	0	nofs	nofs
chr14	C	14p11	.	11625000	0	nofs	nofs
chr14	C	14p11	.	11875000	0	nofs	nofs
chr14	C	14p11	.	12125000	0	nofs	nofs
chr14	C	14p11	.	12375000	0	nofs	nofs
chr14	C	14p11	.	12625000	0	nofs	nofs
chr14	C	14p11	.	12875000	0	nofs	nofs
chr14	C	14p11	.	13125000	0	nofs	nofs
chr14	C	14p11	.	13375000	0	nofs	nofs
chr14	C	14p11	.	13625000	0	nofs	nofs
chr14	C	14p11	.	13875000	0	nofs	nofs

chr14	C	14p11	.	14125000	0	nofs	nofs
chr14	C	14p11	.	14375000	0	nofs	nofs
chr14	C	14p11	.	14625000	0	nofs	nofs
chr14	C	14p11	.	14875000	0	nofs	nofs
chr14	C	14p11	.	15125000	0	nofs	nofs
chr14	C		.	15375000	0	nofs	nofs
chr14	C		.	15625000	0	nofs	nofs
chr14	C		.	15875000	0	nofs	nofs
chr14	C		.	16125000	0	nofs	nofs
chr14	C		.	16375000	0	nofs	nofs
chr14	C		.	16625000	0	nofs	nofs
chr14	C		.	16875000	0	nofs	nofs
chr14	C		.	17125000	0	nofs	nofs
chr14	C		.	17375000	0	nofs	nofs
chr14	C		.	17625000	0	nofs	nofs
chr14	C		.	17875000	0	nofs	nofs
chr14	C	14q11.1	.	18125000	57074	nofs	nofs
chr14	C	14q11.1	.	18375000	125591	nofs	nofs
chr14	C	14q11.1	.	18625000	5483	nofs	nofs
chr14	C	14q11.1	.	18875000	6088	nofs	nofs
chr14	C	14q11.2	3	19125000	4109	nofs	nofs
chr14	C	14q11.2	3	19375000	3645	nofs	nofs
chr14	C	14q11.2	3	19625000	2150	nofs	nofs
chr14	C	14q11.2	3	19875000	5681	nofs	nofs
chr14	C	14q11.2	3	20125000	4301	nofs	nofs
chr14	C	14q11.2	3	20375000	3280	nofs	nofs
chr14	C	14q11.2	3	20625000	3872	nofs	nofs
chr14	C	14q11.2	3	20875000	5258	nofs	nofs
chr14	C	14q11.2	3	21125000	5035	nofs	nofs
chr14	E	14q11.2	3	21375000	1567	nofs	nofs
chr14	E	14q11.2	3	21625000	2503	nofs	nofs
chr14	E	14q11.2	3	21875000	1655	nofs	nofs
chr14	E	14q11.2	3	22125000	7155	nofs	nofs
chr14	E	14q11.2	3	22375000	6805	nofs	nofs
chr14	E	14q11.2	3	22625000	6377	nofs	nofs
chr14	E	14q11.2	3	22875000	3110	nofs	nofs

chr14	E	14q11.2	3	23125000	3929	nofs	nofs
chr14	E	14q11.2	3	23375000	3650	nofs	nofs
chr14	E	14q11.2	3	23625000	2111	nofs	nofs
chr14	E	14q11.2	3	23875000	2393	nofs	nofs
chr14	E	14q11.2	3	24125000	3370	nofs	nofs
chr14	E	14q11.2	3	24375000	1383	nofs	nofs
chr14	B	14q12	.	24625000	2282	nofs	nofs
chr14	B	14q12	.	24875000	1739	nofs	nofs
chr14	B	14q12	.	25125000	2198	nofs	nofs
chr14	B	14q12	.	25375000	3461	nofs	nofs
chr14	B	14q12	.	25625000	1827	nofs	nofs
chr14	B	14q12	.	25875000	2273	nofs	nofs
chr14	B	14q12	.	26125000	1264	nofs	nofs
chr14	B	14q12	.	26375000	1937	nofs	nofs
chr14	B	14q12	.	26625000	2326	nofs	nofs
chr14	B	14q12	.	26875000	2202	nofs	nofs
chr14	B	14q12	.	27125000	1743	nofs	nofs
chr14	B	14q12	.	27375000	2469	nofs	nofs
chr14	B	14q12	.	27625000	2025	nofs	nofs
chr14	B	14q12	.	27875000	1948	nofs	nofs
chr14	B	14q12	.	28125000	2284	nofs	nofs
chr14	B	14q12	.	28375000	2385	nofs	nofs
chr14	B	14q12	.	28625000	4251	nofs	nofs
chr14	B	14q12	.	28875000	2751	nofs	nofs
chr14	B	14q12	.	29125000	3206	nofs	nofs
chr14	B	14q12	.	29375000	3148	nofs	nofs
chr14	B	14q12	.	29625000	2068	nofs	nofs
chr14	B	14q12	.	29875000	1549	nofs	nofs
chr14	B	14q12	.	30125000	1825	nofs	nofs
chr14	B	14q12	.	30375000	2156	nofs	nofs
chr14	B	14q12	.	30625000	2721	nofs	nofs
chr14	B	14q12	.	30875000	5444	nofs	nofs
chr14	B	14q12	.	31125000	3648	nofs	nofs
chr14	B	14q12	.	31375000	2840	nofs	nofs
chr14	B	14q12	.	31625000	2975	nofs	nofs
chr14	B	14q12	.	31875000	2707	nofs	nofs

chr14	B	14q12	.	32125000	1993	nofs	nofs
chr14	E	14q13	1	32375000	3002	nofs	nofs
chr14	E	14q13	1	32625000	1374	nofs	nofs
chr14	E	14q13	1	32875000	1269	nofs	nofs
chr14	E	14q13	1	33125000	679	nofs	nofs
chr14	E	14q13	1	33375000	2363	nofs	nofs
chr14	E	14q13	1	33625000	4102	nofs	nofs
chr14	E	14q13	1	33875000	5352	nofs	nofs
chr14	E	14q13	1	34125000	7947	nofs	nofs
chr14	E	14q13	1	34375000	4913	nofs	nofs
chr14	E	14q13	1	34625000	4831	nofs	nofs
chr14	E	14q13	1	34875000	2846	nofs	nofs
chr14	E	14q13	1	35125000	2490	nofs	nofs
chr14	E	14q13	1	35375000	4751	nofs	nofs
chr14	E	14q13	1	35625000	3537	nofs	nofs
chr14	E	14q13	1	35875000	1948	nofs	nofs
chr14	E	14q13	1	36125000	1295	nofs	nofs
chr14	E	14q13	1	36375000	1903	nofs	nofs
chr14	E	14q13	1	36625000	2031	nofs	nofs
chr14	E	14q13	1	36875000	1542	nofs	nofs
chr14	E	14q21	4	37125000	2810	nofs	nofs
chr14	E	14q21	4	37375000	2094	nofs	nofs
chr14	E	14q21	4	37625000	3396	nofs	nofs
chr14	E	14q21	4	37875000	1635	nofs	nofs
chr14	E	14q21	4	38125000	1720	nofs	nofs
chr14	E	14q21	4	38375000	2381	nofs	nofs
chr14	E	14q21	4	38625000	4019	nofs	nofs
chr14	E	14q21	4	38875000	3589	nofs	nofs
chr14	E	14q21	4	39125000	1271	nofs	nofs
chr14	E	14q21	4	39375000	1644	nofs	nofs
chr14	E	14q21	4	39625000	2277	nofs	nofs
chr14	E	14q21	4	39875000	1698	nofs	nofs
chr14	E	14q21	4	40125000	2338	nofs	nofs
chr14	E	14q21	4	40375000	2850	nofs	nofs
chr14	E	14q21	4	40625000	2381	nofs	nofs
chr14	E	14q21	4	40875000	2353	nofs	nofs

chr14	E	14q21	4	41125000	4771	nofs	nofs
chr14	E	14q21	4	41375000	2730	nofs	nofs
chr14	E	14q21	4	41625000	4194	nofs	nofs
chr14	E	14q21	4	41875000	2541	nofs	nofs
chr14	E	14q21	4	42125000	3790	nofs	nofs
chr14	E	14q21	4	42375000	2913	nofs	nofs
chr14	E	14q21	4	42625000	3062	nofs	nofs
chr14	E	14q21	4	42875000	2277	nofs	nofs
chr14	E	14q21	4	43125000	2900	nofs	nofs
chr14	E	14q21	4	43375000	2257	nofs	nofs
chr14	E	14q21	4	43625000	2663	nofs	nofs
chr14	E	14q21	4	43875000	2496	nofs	nofs
chr14	E	14q21	4	44125000	4358	nofs	nofs
chr14	E	14q21	4	44375000	3578	nofs	nofs
chr14	E	14q21	4	44625000	3019	nofs	nofs
chr14	E	14q21	4	44875000	2982	nofs	nofs
chr14	E	14q21	4	45125000	2633	nofs	nofs
chr14	E	14q21	4	45375000	17752	nofs	nofs
chr14	E	14q21	4	45625000	1812	nofs	nofs
chr14	E	14q21	4	45875000	1932	nofs	nofs
chr14	E	14q21	4	46125000	2591	nofs	nofs
chr14	E	14q21	4	46375000	1668	nofs	nofs
chr14	E	14q21	4	46625000	2213	nofs	nofs
chr14	E	14q21	4	46875000	2489	nofs	nofs
chr14	E	14q21	4	47125000	3961	nofs	nofs
chr14	E	14q21	4	47375000	1558	nofs	nofs
chr14	E	14q21	4	47625000	2239	nofs	nofs
chr14	E	14q21	4	47875000	1465	nofs	nofs
chr14	E	14q21	4	48125000	2777	nofs	nofs
chr14	E	14q21	4	48375000	2961	nofs	nofs
chr14	E	14q21	4	48625000	1157	nofs	nofs
chr14	E	14q21	4	48875000	1515	nofs	nofs
chr14	E	14q21	4	49125000	5337	nofs	nofs
chr14	E	14q21	4	49375000	3254	nofs	nofs
chr14	E	14q21	4	49625000	2599	nofs	nofs
chr14	E	14q21	4	49875000	3022	nofs	nofs

chr14	E	14q21	4	50125000	2308	nofs	nofs
chr14	E	14q21	4	50375000	2681	nofs	nofs
chr14	E	14q21	4	50625000	1776	nofs	nofs
chr14	E	14q21	4	50875000	2623	nofs	nofs
chr14	E	14q22	3	51125000	2495	nofs	nofs
chr14	E	14q22	3	51375000	6743	nofs	nofs
chr14	E	14q22	3	51625000	2491	nofs	nofs
chr14	E	14q22	3	51875000	2056	nofs	nofs
chr14	E	14q22	3	52125000	3216	nofs	nofs
chr14	E	14q22	3	52375000	3056	nofs	nofs
chr14	E	14q22	3	52625000	3481	nofs	nofs
chr14	E	14q22	3	52875000	2256	nofs	nofs
chr14	E	14q22	3	53125000	2036	nofs	nofs
chr14	E	14q22	3	53375000	1978	nofs	nofs
chr14	E	14q22	3	53625000	2761	nofs	nofs
chr14	E	14q22	3	53875000	2732	nofs	nofs
chr14	E	14q22	3	54125000	2364	nofs	nofs
chr14	E	14q22	3	54375000	2596	nofs	nofs
chr14	E	14q22	3	54625000	2644	nofs	nofs
chr14	E	14q22	3	54875000	3768	nofs	nofs
chr14	E	14q22	3	55125000	1973	nofs	nofs
chr14	E	14q22	3	55375000	2219	nofs	nofs
chr14	E	14q22	3	55625000	1941	nofs	nofs
chr14	E	14q22	3	55875000	1417	nofs	nofs
chr14	E	14q22	3	56125000	1813	nofs	nofs
chr14	E	14q22	3	56375000	2915	nofs	nofs
chr14	E	14q22	3	56625000	3180	nofs	nofs
chr14	E	14q22	3	56875000	2265	nofs	nofs
chr14	E	14q22	3	57125000	1652	nofs	nofs
chr14	E	14q22	3	57375000	2277	nofs	nofs
chr14	E	14q23	1	57625000	1982	fs	fs common
chr14	E	14q23	1	57875000	1933	fs	fs common
chr14	E	14q23	1	58125000	1807	fs	fs common
chr14	E	14q23	1	58375000	2662	fs	fs common
chr14	E	14q23	1	58625000	2661	fs	fs common
chr14	E	14q23	1	58875000	1901	fs	fs common

chr14	E	14q23	1	59125000	1220	fs	fs common
chr14	E	14q23	1	59375000	3429	fs	fs common
chr14	E	14q23	1	59625000	1305	fs	fs common
chr14	E	14q23	1	59875000	3879	fs	fs common
chr14	E	14q23	1	60125000	2843	fs	fs common
chr14	E	14q23	1	60375000	2412	fs	fs common
chr14	E	14q23	1	60625000	2566	fs	fs common
chr14	E	14q23	1	60875000	2048	fs	fs common
chr14	E	14q23	1	61125000	1374	fs	fs common
chr14	E	14q23	1	61375000	1583	fs	fs common
chr14	E	14q23	1	61625000	1663	fs	fs common
chr14	E	14q23	1	61875000	1835	fs	fs common
chr14	E	14q23	1	62125000	1244	fs	fs common
chr14	E	14q23	1	62375000	2532	fs	fs common
chr14	E	14q23	1	62625000	3094	fs	fs common
chr14	E	14q23	1	62875000	3253	fs	fs common
chr14	E	14q23	1	63125000	4069	fs	fs common
chr14	E	14q23	1	63375000	6467	fs	fs common
chr14	E	14q23	1	63625000	3762	fs	fs common
chr14	E	14q23	1	63875000	2810	fs	fs common
chr14	E	14q23	1	64125000	2849	fs	fs common
chr14	E	14q23	1	64375000	4370	fs	fs common
chr14	E	14q23	1	64625000	2301	fs	fs common
chr14	E	14q23	1	64875000	3887	fs	fs common
chr14	E	14q23	1	65125000	4338	fs	fs common
chr14	E	14q23	1	65375000	3806	fs	fs common
chr14	E	14q23	1	65625000	1533	fs	fs common
chr14	E	14q23	1	65875000	1560	fs	fs common
chr14	E	14q23	1	66125000	2681	fs	fs common
chr14	E	14q23	1	66375000	1895	fs	fs common
chr14	E	14q23	1	66625000	3871	fs	fs common
chr14	E	14q23	1	66875000	2883	fs	fs common
chr14	E	14q23	1	67125000	2191	fs	fs common
chr14	B	14q24.1	.	67375000	2123	fs	fs common
chr14	B	14q24.1	.	67625000	2269	fs	fs common
chr14	B	14q24.1	.	67875000	2670	fs	fs common

chr14	B	14q24.1	.	68125000	2910	fs	fs common
chr14	B	14q24.1	.	68375000	2915	fs	fs common
chr14	B	14q24.1	.	68625000	2617	fs	fs common
chr14	B	14q24.1	.	68875000	2271	fs	fs common
chr14	B	14q24.1	.	69125000	3103	fs	fs common
chr14	B	14q24.1	.	69375000	3727	fs	fs common
chr14	B	14q24.2	.	69625000	2103	nofs	nofs
chr14	B	14q24.2	.	69875000	3638	nofs	nofs
chr14	B	14q24.2	.	70125000	3290	nofs	nofs
chr14	B	14q24.2	.	70375000	3351	nofs	nofs
chr14	B	14q24.2	.	70625000	2294	nofs	nofs
chr14	B	14q24.2	.	70875000	3094	nofs	nofs
chr14	B	14q24.2	.	71125000	4044	nofs	nofs
chr14	B	14q24.2	.	71375000	2554	nofs	nofs
chr14	B	14q24.2	.	71625000	1892	nofs	nofs
chr14	B	14q24.2	.	71875000	1318	nofs	nofs
chr14	B	14q24.2	.	72125000	1970	nofs	nofs
chr14	B	14q24.2	.	72375000	3767	nofs	nofs
chr14	B	14q24.2	.	72625000	5261	nofs	nofs
chr14	B	14q24.2	.	72875000	3317	nofs	nofs
chr14	B	14q24.3	.	73125000	5102	nofs	nofs
chr14	B	14q24.3	.	73375000	3058	nofs	nofs
chr14	B	14q24.3	.	73625000	4511	nofs	nofs
chr14	B	14q24.3	.	73875000	5404	nofs	nofs
chr14	B	14q24.3	.	74125000	3035	nofs	nofs
chr14	B	14q24.3	.	74375000	2769	nofs	nofs
chr14	B	14q24.3	.	74625000	5359	nofs	nofs
chr14	B	14q24.3	.	74875000	3304	nofs	nofs
chr14	B	14q24.3	.	75125000	3182	nofs	nofs
chr14	B	14q24.3	.	75375000	2527	nofs	nofs
chr14	B	14q24.3	.	75625000	1311	nofs	nofs
chr14	B	14q24.3	.	75875000	4681	nofs	nofs
chr14	B	14q24.3	.	76125000	3659	nofs	nofs
chr14	B	14q24.3	.	76375000	7578	nofs	nofs
chr14	B	14q24.3	.	76625000	3720	nofs	nofs
chr14	B	14q24.3	.	76875000	3638	nofs	nofs

chr14	B	14q24.3	.	77125000	4642	nofs	nofs
chr14	B	14q24.3	.	77375000	2922	nofs	nofs
chr14	B	14q24.3	.	77625000	2483	nofs	nofs
chr14	B	14q24.3	.	77875000	1600	nofs	nofs
chr14	B	14q24.3	.	78125000	3128	nofs	nofs
chr14	B	14q24.3	.	78375000	2095	nofs	nofs
chr14	B	14q31	.	78625000	1756	nofs	nofs
chr14	B	14q31	.	78875000	3181	nofs	nofs
chr14	B	14q31	.	79125000	1864	nofs	nofs
chr14	B	14q31	.	79375000	2941	nofs	nofs
chr14	B	14q31	.	79625000	1528	nofs	nofs
chr14	B	14q31	.	79875000	2438	nofs	nofs
chr14	B	14q31	.	80125000	1886	nofs	nofs
chr14	B	14q31	.	80375000	2115	nofs	nofs
chr14	B	14q31	.	80625000	1246	nofs	nofs
chr14	B	14q31	.	80875000	3606	nofs	nofs
chr14	B	14q31	.	81125000	4078	nofs	nofs
chr14	B	14q31	.	81375000	3057	nofs	nofs
chr14	B	14q31	.	81625000	2065	nofs	nofs
chr14	B	14q31	.	81875000	2558	nofs	nofs
chr14	B	14q31	.	82125000	4331	nofs	nofs
chr14	B	14q31	.	82375000	4504	nofs	nofs
chr14	B	14q31	.	82625000	2956	nofs	nofs
chr14	B	14q31	.	82875000	2974	nofs	nofs
chr14	B	14q31	.	83125000	1955	nofs	nofs
chr14	B	14q31	.	83375000	2060	nofs	nofs
chr14	B	14q31	.	83625000	4123	nofs	nofs
chr14	B	14q31	.	83875000	2470	nofs	nofs
chr14	B	14q31	.	84125000	5105	nofs	nofs
chr14	B	14q31	.	84375000	3616	nofs	nofs
chr14	B	14q31	.	84625000	2465	nofs	nofs
chr14	B	14q31	.	84875000	2320	nofs	nofs
chr14	B	14q31	.	85125000	2400	nofs	nofs
chr14	B	14q31	.	85375000	5636	nofs	nofs
chr14	B	14q31	.	85625000	2864	nofs	nofs
chr14	B	14q31	.	85875000	5114	nofs	nofs

chr14	B	14q31	.	86125000	2438	nofs	nofs
chr14	B	14q31	.	86375000	3144	nofs	nofs
chr14	B	14q31	.	86625000	2858	nofs	nofs
chr14	B	14q31	.	86875000	2807	nofs	nofs
chr14	B	14q31	.	87125000	1908	nofs	nofs
chr14	B	14q31	.	87375000	2289	nofs	nofs
chr14	B	14q31	.	87625000	1521	nofs	nofs
chr14	B	14q31	.	87875000	1366	nofs	nofs
chr14	B	14q31	.	88125000	2804	nofs	nofs
chr14	B	14q32.1	.	88375000	3862	nofs	nofs
chr14	B	14q32.1	.	88625000	2335	nofs	nofs
chr14	B	14q32.1	.	88875000	5185	nofs	nofs
chr14	B	14q32.1	.	89125000	3910	nofs	nofs
chr14	B	14q32.1	.	89375000	3043	nofs	nofs
chr14	B	14q32.1	.	89625000	2890	nofs	nofs
chr14	B	14q32.1	.	89875000	2901	nofs	nofs
chr14	B	14q32.1	.	90125000	4333	nofs	nofs
chr14	B	14q32.1	.	90375000	3151	nofs	nofs
chr14	B	14q32.1	.	90625000	5000	nofs	nofs
chr14	B	14q32.1	.	90875000	3769	nofs	nofs
chr14	B	14q32.1	.	91125000	5689	nofs	nofs
chr14	B	14q32.1	.	91375000	2574	nofs	nofs
chr14	B	14q32.1	.	91625000	3042	nofs	nofs
chr14	B	14q32.1	.	91875000	3861	nofs	nofs
chr14	B	14q32.1	.	92125000	5990	nofs	nofs
chr14	B	14q32.1	.	92375000	4249	nofs	nofs
chr14	B	14q32.1	.	92625000	6459	nofs	nofs
chr14	B	14q32.1	.	92875000	2136	nofs	nofs
chr14	B	14q32.1	.	93125000	2140	nofs	nofs
chr14	B	14q32.1	.	93375000	3900	nofs	nofs
chr14	B	14q32.1	.	93625000	3310	nofs	nofs
chr14	B	14q32.1	.	93875000	3244	nofs	nofs
chr14	B	14q32.1	.	94125000	6379	nofs	nofs
chr14	B	14q32.1	.	94375000	6398	nofs	nofs
chr14	B	14q32.1	.	94625000	3762	nofs	nofs
chr14	B	14q32.1	.	94875000	6545	nofs	nofs

chr14	B	14q32.1	.	95125000	3671	nofs	nofs
chr14	B	14q32.1	.	95375000	2942	nofs	nofs
chr14	B	14q32.2	.	95625000	3451	nofs	nofs
chr14	B	14q32.2	.	95875000	3812	nofs	nofs
chr14	B	14q32.2	.	96125000	3488	nofs	nofs
chr14	B	14q32.2	.	96375000	3900	nofs	nofs
chr14	B	14q32.2	.	96625000	5370	nofs	nofs
chr14	B	14q32.2	.	96875000	3689	nofs	nofs
chr14	B	14q32.2	.	97125000	1809	nofs	nofs
chr14	B	14q32.2	.	97375000	3824	nofs	nofs
chr14	B	14q32.2	.	97625000	3815	nofs	nofs
chr14	B	14q32.2	.	97875000	3611	nofs	nofs
chr14	B	14q32.2	.	98125000	2872	nofs	nofs
chr14	B	14q32.2	.	98375000	3537	nofs	nofs
chr14	B	14q32.2	.	98625000	3817	nofs	nofs
chr14	B	14q32.2	.	98875000	5430	nofs	nofs
chr14	B	14q32.2	.	99125000	5525	nofs	nofs
chr14	B	14q32.2	.	99375000	3182	nofs	nofs
chr14	B	14q32.2	.	99625000	6600	nofs	nofs
chr14	B	14q32.2	.	99875000	3130	nofs	nofs
chr14	B	14q32.2	.	100000000	6188	nofs	nofs
chr14	B	14q32.2	.	100000000	5564	nofs	nofs
chr14	E	14q32.3	1	101000000	5792	nofs	nofs
chr14	E	14q32.3	1	101000000	5916	nofs	nofs
chr14	E	14q32.3	1	101000000	7619	nofs	nofs
chr14	E	14q32.3	1	101000000	7202	nofs	nofs
chr14	E	14q32.3	1	102000000	4816	nofs	nofs
chr14	E	14q32.3	1	102000000	4822	nofs	nofs
chr14	E	14q32.3	1	102000000	8068	nofs	nofs
chr14	E	14q32.3	1	102000000	7144	nofs	nofs
chr14	E	14q32.3	1	103000000	7685	nofs	nofs
chr14	E	14q32.3	1	103000000	7923	nofs	nofs
chr14	T	14q32.3	1	103000000	4391	nofs	nofs
chr14	T	14q32.3	1	103000000	3805	nofs	nofs
chr14	T	14q32.3	1	104000000	8464	nofs	nofs
chr14	T	14q32.3	1	104000000	14647	nofs	nofs

chr14	T	14q32.3	1	104000000	11267	nofs	nofs
chr14	T	14q32.3	1	104000000	28270	nofs	nofs
chr14	T	14q32.3	1	105000000	12601	nofs	nofs
chr14	T	14q32.3	1	105000000	25081	nofs	nofs
chr14	T	14q32.3	1	105000000	33501	nofs	nofs
chr14	T	14q32.3	1	105000000	20486	nofs	nofs
chr14	T	14q32.3	1	106000000	7999	nofs	nofs
chr14	T	14q32.3	1	106000000	2957	nofs	nofs
chr14	T	14q32.3	1	106000000	4799	nofs	nofs
chr15	C	15p13	.	125000	0	nofs	nofs
chr15	C	15p13	.	375000	0	nofs	nofs
chr15	C	15p13	.	625000	0	nofs	nofs
chr15	C	15p13	.	875000	0	nofs	nofs
chr15	C	15p13	.	1125000	0	nofs	nofs
chr15	C	15p13	.	1375000	0	nofs	nofs
chr15	C	15p13	.	1625000	0	nofs	nofs
chr15	C	15p13	.	1875000	0	nofs	nofs
chr15	C	15p13	.	2125000	0	nofs	nofs
chr15	C	15p13	.	2375000	0	nofs	nofs
chr15	C	15p13	.	2625000	0	nofs	nofs
chr15	C	15p13	.	2875000	0	nofs	nofs
chr15	C	15p13	.	3125000	0	nofs	nofs
chr15	C	15p13	.	3375000	0	nofs	nofs
chr15	C	15p13	.	3625000	0	nofs	nofs
chr15	C	15p12	.	3875000	0	nofs	nofs
chr15	C	15p12	.	4125000	0	nofs	nofs
chr15	C	15p12	.	4375000	0	nofs	nofs
chr15	C	15p12	.	4625000	0	nofs	nofs
chr15	C	15p12	.	4875000	0	nofs	nofs
chr15	C	15p12	.	5125000	0	nofs	nofs
chr15	C	15p12	.	5375000	0	nofs	nofs
chr15	C	15p12	.	5625000	0	nofs	nofs
chr15	C	15p12	.	5875000	0	nofs	nofs
chr15	C	15p12	.	6125000	0	nofs	nofs
chr15	C	15p12	.	6375000	0	nofs	nofs
chr15	C	15p12	.	6625000	0	nofs	nofs

chr15	C	15p12	.	6875000	0	nofs	nofs
chr15	C	15p12	.	7125000	0	nofs	nofs
chr15	C	15p12	.	7375000	0	nofs	nofs
chr15	C	15p12	.	7625000	0	nofs	nofs
chr15	C	15p12	.	7875000	0	nofs	nofs
chr15	C	15p11	.	8125000	0	nofs	nofs
chr15	C	15p11	.	8375000	0	nofs	nofs
chr15	C	15p11	.	8625000	0	nofs	nofs
chr15	C	15p11	.	8875000	0	nofs	nofs
chr15	C	15p11	.	9125000	0	nofs	nofs
chr15	C	15p11	.	9375000	0	nofs	nofs
chr15	C	15p11	.	9625000	0	nofs	nofs
chr15	C	15p11	.	9875000	0	nofs	nofs
chr15	C	15p11	.	10125000	0	nofs	nofs
chr15	C	15p11	.	10375000	0	nofs	nofs
chr15	C	15p11	.	10625000	0	nofs	nofs
chr15	C	15p11	.	10875000	0	nofs	nofs
chr15	C	15p11	.	11125000	0	nofs	nofs
chr15	C	15p11	.	11375000	0	nofs	nofs
chr15	C	15p11	.	11625000	0	nofs	nofs
chr15	C	15p11	.	11875000	0	nofs	nofs
chr15	C	15p11	.	12125000	0	nofs	nofs
chr15	C	15p11	.	12375000	0	nofs	nofs
chr15	C	15p11	.	12625000	0	nofs	nofs
chr15	C	15p11	.	12875000	0	nofs	nofs
chr15	C	15p11	.	13125000	0	nofs	nofs
chr15	C	15p11	.	13375000	0	nofs	nofs
chr15	C	15p11	.	13625000	0	nofs	nofs
chr15	C	15p11	.	13875000	0	nofs	nofs
chr15	C	15p11	.	14125000	0	nofs	nofs
chr15	C	15p11	.	14375000	0	nofs	nofs
chr15	C	15p11	.	14625000	0	nofs	nofs
chr15	C		.	14875000	0	nofs	nofs
chr15	C		.	15125000	0	nofs	nofs
chr15	C		.	15375000	0	nofs	nofs
chr15	C		.	15625000	0	nofs	nofs

chr15	C		.	15875000	0	nofs	nofs
chr15	C		.	16125000	0	nofs	nofs
chr15	C		.	16375000	0	nofs	nofs
chr15	C		.	16625000	0	nofs	nofs
chr15	C		.	16875000	0	nofs	nofs
chr15	C		.	17125000	0	nofs	nofs
chr15	C		.	17375000	0	nofs	nofs
chr15	C	15q11.1	.	17625000	0	nofs	nofs
chr15	C	15q11.1	.	17875000	0	nofs	nofs
chr15	C	15q11.1	.	18125000	0	nofs	nofs
chr15	C	15q11.2	2	18375000	43081	nofs	nofs
chr15	C	15q11.2	2	18625000	19324	nofs	nofs
chr15	C	15q11.2	2	18875000	13468	nofs	nofs
chr15	C	15q11.2	2	19125000	3809	nofs	nofs
chr15	C	15q11.2	2	19375000	3386	nofs	nofs
chr15	C	15q11.2	2	19625000	16408	nofs	nofs
chr15	C	15q11.2	2	19875000	5752	nofs	nofs
chr15	C	15q11.2	2	20125000	5746	nofs	nofs
chr15	C	15q11.2	2	20375000	16050	nofs	nofs
chr15	C	15q11.2	2	20625000	6450	nofs	nofs
chr15	C	15q11.2	2	20875000	6555	nofs	nofs
chr15	C	15q11.2	2	21125000	5172	nofs	nofs
chr15	C	15q11.2	2	21375000	4312	nofs	nofs
chr15	E	15q11.2	2	21625000	2647	nofs	nofs
chr15	E	15q11.2	2	21875000	2766	nofs	nofs
chr15	E	15q11.2	2	22125000	1955	nofs	nofs
chr15	E	15q11.2	2	22375000	1828	nofs	nofs
chr15	E	15q11.2	2	22625000	4560	nofs	nofs
chr15	E	15q11.2	2	22875000	1660	nofs	nofs
chr15	E	15q11.2	2	23125000	2160	nofs	nofs
chr15	E	15q11.2	2	23375000	2331	nofs	nofs
chr15	E	15q12	1	23625000	3871	nofs	nofs
chr15	E	15q12	1	23875000	5083	nofs	nofs
chr15	E	15q12	1	24125000	3270	nofs	nofs
chr15	E	15q12	1	24375000	1981	nofs	nofs
chr15	E	15q12	1	24625000	4050	nofs	nofs

chr15	E	15q12	1	24875000	2757	nofs	nofs
chr15	E	15q12	1	25125000	4640	nofs	nofs
chr15	E	15q12	1	25375000	4676	nofs	nofs
chr15	E	15q12	1	25625000	4463	nofs	nofs
chr15	E	15q12	1	25875000	4784	nofs	nofs
chr15	E	15q13	7	26125000	7705	nofs	nofs
chr15	E	15q13	7	26375000	5174	nofs	nofs
chr15	E	15q13	7	26625000	7604	nofs	nofs
chr15	E	15q13	7	26875000	3831	nofs	nofs
chr15	E	15q13	7	27125000	3163	nofs	nofs
chr15	E	15q13	7	27375000	2634	nofs	nofs
chr15	E	15q13	7	27625000	4018	nofs	nofs
chr15	E	15q13	7	27875000	1204	nofs	nofs
chr15	E	15q13	7	28125000	5293	nofs	nofs
chr15	E	15q13	7	28375000	3534	nofs	nofs
chr15	E	15q13	7	28625000	5308	nofs	nofs
chr15	E	15q13	7	28875000	6737	nofs	nofs
chr15	E	15q13	7	29125000	3853	nofs	nofs
chr15	E	15q13	7	29375000	3809	nofs	nofs
chr15	E	15q13	7	29625000	3057	nofs	nofs
chr15	E	15q13	7	29875000	2159	nofs	nofs
chr15	E	15q13	7	30125000	2968	nofs	nofs
chr15	E	15q13	7	30375000	2659	nofs	nofs
chr15	E	15q13	7	30625000	5229	nofs	nofs
chr15	E	15q13	7	30875000	2257	nofs	nofs
chr15	E	15q13	7	31125000	2049	nofs	nofs
chr15	E	15q13	7	31375000	2584	nofs	nofs
chr15	E	15q14	1	31625000	1104	nofs	nofs
chr15	E	15q14	1	31875000	2519	nofs	nofs
chr15	E	15q14	1	32125000	3482	nofs	nofs
chr15	E	15q14	1	32375000	3441	nofs	nofs
chr15	E	15q14	1	32625000	2764	nofs	nofs
chr15	E	15q14	1	32875000	1956	nofs	nofs
chr15	E	15q14	1	33125000	4955	nofs	nofs
chr15	E	15q14	1	33375000	2800	nofs	nofs
chr15	E	15q14	1	33625000	3332	nofs	nofs

chr15	E	15q14	1	33875000	1905	nofs	nofs
chr15	E	15q14	1	34125000	1157	nofs	nofs
chr15	E	15q14	1	34375000	2015	nofs	nofs
chr15	E	15q14	1	34625000	2185	nofs	nofs
chr15	E	15q14	1	34875000	1837	nofs	nofs
chr15	E	15q14	1	35125000	2635	nofs	nofs
chr15	E	15q14	1	35375000	2436	nofs	nofs
chr15	E	15q14	1	35625000	2023	nofs	nofs
chr15	E	15q14	1	35875000	1917	nofs	nofs
chr15	E	15q14	1	36125000	2451	nofs	nofs
chr15	E	15q14	1	36375000	1464	nofs	nofs
chr15	E	15q14	1	36625000	1497	nofs	nofs
chr15	E	15q14	1	36875000	1606	nofs	nofs
chr15	E	15q14	1	37125000	2140	nofs	nofs
chr15	E	15q14	1	37375000	1988	nofs	nofs
chr15	E	15q14	1	37625000	1227	nofs	nofs
chr15	E	15q14	1	37875000	3214	nofs	nofs
chr15	E	15q15	2	38125000	7100	nofs	nofs
chr15	E	15q15	2	38375000	1872	nofs	nofs
chr15	E	15q15	2	38625000	2820	nofs	nofs
chr15	E	15q15	2	38875000	4964	nofs	nofs
chr15	E	15q15	2	39125000	3826	nofs	nofs
chr15	E	15q15	2	39375000	7869	nofs	nofs
chr15	E	15q15	2	39625000	5403	nofs	nofs
chr15	E	15q15	2	39875000	2959	nofs	nofs
chr15	E	15q15	2	40125000	2687	nofs	nofs
chr15	E	15q15	2	40375000	2091	nofs	nofs
chr15	E	15q15	2	40625000	2943	nofs	nofs
chr15	E	15q15	2	40875000	2886	nofs	nofs
chr15	E	15q15	2	41125000	5091	nofs	nofs
chr15	E	15q15	2	41375000	2262	nofs	nofs
chr15	E	15q15	2	41625000	3333	nofs	nofs
chr15	E	15q15	2	41875000	3864	nofs	nofs
chr15	E	15q15	2	42125000	3559	nofs	nofs
chr15	E	15q15	2	42375000	3846	nofs	nofs
chr15	E	15q21.1	2	42625000	1831	nofs	nofs

chr15	E	15q21.1	2	42875000	2775	nofs	nofs
chr15	E	15q21.1	2	43125000	2665	nofs	nofs
chr15	E	15q21.1	2	43375000	2937	nofs	nofs
chr15	E	15q21.1	2	43625000	5891	nofs	nofs
chr15	E	15q21.1	2	43875000	3315	nofs	nofs
chr15	E	15q21.1	2	44125000	3135	nofs	nofs
chr15	E	15q21.1	2	44375000	1117	nofs	nofs
chr15	E	15q21.1	2	44625000	1706	nofs	nofs
chr15	E	15q21.1	2	44875000	1311	nofs	nofs
chr15	E	15q21.1	2	45125000	4125	nofs	nofs
chr15	E	15q21.1	2	45375000	2309	nofs	nofs
chr15	E	15q21.1	2	45625000	1788	nofs	nofs
chr15	E	15q21.1	2	45875000	1396	nofs	nofs
chr15	E	15q21.1	2	46125000	2318	nofs	nofs
chr15	E	15q21.1	2	46375000	1986	nofs	nofs
chr15	E	15q21.1	2	46625000	2397	nofs	nofs
chr15	E	15q21.1	2	46875000	1737	nofs	nofs
chr15	E	15q21.1	2	47125000	2255	nofs	nofs
chr15	E	15q21.1	2	47375000	3207	nofs	nofs
chr15	E	15q21.1	2	47625000	2640	nofs	nofs
chr15	B	15q21.2	.	47875000	1788	nofs	nofs
chr15	B	15q21.2	.	48125000	3754	nofs	nofs
chr15	B	15q21.2	.	48375000	3135	nofs	nofs
chr15	B	15q21.2	.	48625000	3306	nofs	nofs
chr15	B	15q21.2	.	48875000	4967	nofs	nofs
chr15	B	15q21.2	.	49125000	1174	nofs	nofs
chr15	B	15q21.2	.	49375000	2591	nofs	nofs
chr15	B	15q21.2	.	49625000	2394	nofs	nofs
chr15	B	15q21.2	.	49875000	3064	nofs	nofs
chr15	B	15q21.2	.	50125000	3568	nofs	nofs
chr15	B	15q21.2	.	50375000	2449	nofs	nofs
chr15	B	15q21.2	.	50625000	2701	nofs	nofs
chr15	B	15q21.2	.	50875000	1961	nofs	nofs
chr15	B	15q21.3	.	51125000	2747	nofs	nofs
chr15	B	15q21.3	.	51375000	1875	nofs	nofs
chr15	B	15q21.3	.	51625000	1600	nofs	nofs

chr15	B	15q21.3	.	51875000	1317	nofs	nofs
chr15	B	15q21.3	.	52125000	1684	nofs	nofs
chr15	B	15q21.3	.	52375000	1409	nofs	nofs
chr15	B	15q21.3	.	52625000	3593	nofs	nofs
chr15	B	15q21.3	.	52875000	1984	nofs	nofs
chr15	B	15q21.3	.	53125000	2060	nofs	nofs
chr15	B	15q21.3	.	53375000	3761	nofs	nofs
chr15	B	15q21.3	.	53625000	6184	nofs	nofs
chr15	B	15q21.3	.	53875000	2090	nofs	nofs
chr15	B	15q21.3	.	54125000	4135	nofs	nofs
chr15	B	15q21.3	.	54375000	1705	nofs	nofs
chr15	B	15q21.3	.	54625000	2529	nofs	nofs
chr15	B	15q21.3	.	54875000	2178	nofs	nofs
chr15	B	15q21.3	.	55125000	2872	nofs	nofs
chr15	B	15q21.3	.	55375000	4332	nofs	nofs
chr15	B	15q21.3	.	55625000	1361	nofs	nofs
chr15	B	15q21.3	.	55875000	4605	nofs	nofs
chr15	B	15q21.3	.	56125000	1089	nofs	nofs
chr15	B	15q21.3	.	56375000	2452	nofs	nofs
chr15	B	15q21.3	.	56625000	2639	nofs	nofs
chr15	B	15q21.3	.	56875000	3308	nofs	nofs
chr15	B	15q22.1	.	57125000	3220	fs	fs common
chr15	B	15q22.2	.	57375000	2892	fs	fs common
chr15	B	15q22.2	.	57625000	4901	fs	fs common
chr15	B	15q22.2	.	57875000	3498	fs	fs common
chr15	B	15q22.2	.	58125000	2385	fs	fs common
chr15	B	15q22.2	.	58375000	3526	fs	fs common
chr15	B	15q22.2	.	58625000	2417	fs	fs common
chr15	B	15q22.2	.	58875000	2375	fs	fs common
chr15	B	15q22.2	.	59125000	3410	fs	fs common
chr15	B	15q22.2	.	59375000	1431	fs	fs common
chr15	B	15q22.2	.	59625000	1595	fs	fs common
chr15	B	15q22.2	.	59875000	2051	fs	fs common
chr15	B	15q22.2	.	60125000	1950	fs	fs common
chr15	B	15q22.2	.	60375000	4131	fs	fs common
chr15	B	15q22.2	.	60625000	2196	fs	fs common

chr15	B	15q22.2	.	60875000	2442	fs	fs common
chr15	B	15q22.2	.	61125000	2366	fs	fs common
chr15	E	15q22.3	2	61375000	2331	fs	fs common
chr15	E	15q22.3	2	61625000	1627	fs	fs common
chr15	E	15q22.3	2	61875000	1612	fs	fs common
chr15	E	15q22.3	2	62125000	2047	fs	fs common
chr15	E	15q22.3	2	62375000	3901	fs	fs common
chr15	E	15q22.3	2	62625000	3284	fs	fs common
chr15	E	15q22.3	2	62875000	4013	fs	fs common
chr15	E	15q22.3	2	63125000	6969	fs	fs common
chr15	E	15q22.3	2	63375000	2467	fs	fs common
chr15	E	15q22.3	2	63625000	3803	fs	fs common
chr15	E	15q22.3	2	63875000	3693	fs	fs common
chr15	E	15q22.3	2	64125000	2532	fs	fs common
chr15	E	15q22.3	2	64375000	3270	fs	fs common
chr15	E	15q22.3	2	64625000	4110	fs	fs common
chr15	E	15q22.3	2	64875000	1869	fs	fs common
chr15	E	15q22.3	2	65125000	3736	fs	fs common
chr15	E	15q22.3	2	65375000	2595	fs	fs common
chr15	E	15q23	3	65625000	2156	nofs	nofs
chr15	E	15q23	3	65875000	1929	nofs	nofs
chr15	E	15q23	3	66125000	4179	nofs	nofs
chr15	E	15q23	3	66375000	6893	nofs	nofs
chr15	E	15q23	3	66625000	2328	nofs	nofs
chr15	E	15q23	3	66875000	5033	nofs	nofs
chr15	E	15q23	3	67125000	3531	nofs	nofs
chr15	E	15q23	3	67375000	5382	nofs	nofs
chr15	E	15q23	3	67625000	2461	nofs	nofs
chr15	E	15q23	3	67875000	2255	nofs	nofs
chr15	E	15q23	3	68125000	3124	nofs	nofs
chr15	E	15q23	3	68375000	4567	nofs	nofs
chr15	E	15q23	3	68625000	6040	nofs	nofs
chr15	E	15q23	3	68875000	4177	nofs	nofs
chr15	E	15q23	3	69125000	2095	nofs	nofs
chr15	E	15q23	3	69375000	2565	nofs	nofs
chr15	E	15q23	3	69625000	2873	nofs	nofs

chr15	E	15q23	3	69875000	4744	nofs	nofs
chr15	E	15q23	3	70125000	7398	nofs	nofs
chr15	E	15q23	3	70375000	3057	nofs	nofs
chr15	E	15q24	6	70625000	2517	nofs	nofs
chr15	E	15q24	6	70875000	3811	nofs	nofs
chr15	E	15q24	6	71125000	1761	nofs	nofs
chr15	E	15q24	6	71375000	3796	nofs	nofs
chr15	E	15q24	6	71625000	2431	nofs	nofs
chr15	E	15q24	6	71875000	2608	nofs	nofs
chr15	E	15q24	6	72125000	4079	nofs	nofs
chr15	E	15q24	6	72375000	2946	nofs	nofs
chr15	E	15q24	6	72625000	3427	nofs	nofs
chr15	E	15q24	6	72875000	7491	nofs	nofs
chr15	E	15q24	6	73125000	5046	nofs	nofs
chr15	E	15q24	6	73375000	3037	nofs	nofs
chr15	E	15q24	6	73625000	6065	nofs	nofs
chr15	E	15q24	6	73875000	2122	nofs	nofs
chr15	E	15q24	6	74125000	2185	nofs	nofs
chr15	E	15q24	6	74375000	2159	nofs	nofs
chr15	E	15q24	6	74625000	2150	nofs	nofs
chr15	E	15q24	6	74875000	1668	nofs	nofs
chr15	E	15q24	6	75125000	3090	nofs	nofs
chr15	E	15q24	6	75375000	1944	nofs	nofs
chr15	E	15q24	6	75625000	5208	nofs	nofs
chr15	E	15q24	6	75875000	4053	nofs	nofs
chr15	E	15q25	7	76125000	3756	nofs	nofs
chr15	E	15q25	7	76375000	2803	nofs	nofs
chr15	E	15q25	7	76625000	1952	nofs	nofs
chr15	E	15q25	7	76875000	2384	nofs	nofs
chr15	E	15q25	7	77125000	5247	nofs	nofs
chr15	E	15q25	7	77375000	2963	nofs	nofs
chr15	E	15q25	7	77625000	4816	nofs	nofs
chr15	E	15q25	7	77875000	2680	nofs	nofs
chr15	E	15q25	7	78125000	4350	nofs	nofs
chr15	E	15q25	7	78375000	2072	nofs	nofs
chr15	E	15q25	7	78625000	3230	nofs	nofs

chr15	E	15q25	7	78875000	2764	nofs	nofs
chr15	E	15q25	7	79125000	2665	nofs	nofs
chr15	E	15q25	7	79375000	3886	nofs	nofs
chr15	E	15q25	7	79625000	2920	nofs	nofs
chr15	E	15q25	7	79875000	4784	nofs	nofs
chr15	E	15q25	7	80125000	2156	nofs	nofs
chr15	E	15q25	7	80375000	3228	nofs	nofs
chr15	E	15q25	7	80625000	5442	nofs	nofs
chr15	E	15q25	7	80875000	5799	nofs	nofs
chr15	E	15q25	7	81125000	3161	nofs	nofs
chr15	E	15q25	7	81375000	3187	nofs	nofs
chr15	E	15q25	7	81625000	4495	nofs	nofs
chr15	E	15q25	7	81875000	2112	nofs	nofs
chr15	E	15q25	7	82125000	2258	nofs	nofs
chr15	E	15q25	7	82375000	2964	nofs	nofs
chr15	E	15q25	7	82625000	3144	nofs	nofs
chr15	E	15q25	7	82875000	3206	nofs	nofs
chr15	E	15q25	7	83125000	3444	nofs	nofs
chr15	E	15q25	7	83375000	1853	nofs	nofs
chr15	E	15q25	7	83625000	4518	nofs	nofs
chr15	E	15q25	7	83875000	2228	nofs	nofs
chr15	E	15q25	7	84125000	4162	nofs	nofs
chr15	E	15q25	7	84375000	2467	nofs	nofs
chr15	E	15q25	7	84625000	2443	nofs	nofs
chr15	E	15q25	7	84875000	1712	nofs	nofs
chr15	E	15q25	7	85125000	2559	nofs	nofs
chr15	E	15q25	7	85375000	3739	nofs	nofs
chr15	E	15q25	7	85625000	2114	nofs	nofs
chr15	E	15q25	7	85875000	2264	nofs	nofs
chr15	E	15q25	7	86125000	2357	nofs	nofs
chr15	E	15q25	7	86375000	1826	nofs	nofs
chr15	E	15q25	7	86625000	2898	nofs	nofs
chr15	E	15q25	7	86875000	4263	nofs	nofs
chr15	E	15q26.1	5	87125000	5322	nofs	nofs
chr15	E	15q26.1	5	87375000	4517	nofs	nofs
chr15	E	15q26.1	5	87625000	3045	nofs	nofs

chr15	E	15q26.1	5	87875000	6158	nofs	nofs
chr15	E	15q26.1	5	88125000	8359	nofs	nofs
chr15	E	15q26.1	5	88375000	3816	nofs	nofs
chr15	E	15q26.1	5	88625000	4143	nofs	nofs
chr15	E	15q26.1	5	88875000	4106	nofs	nofs
chr15	E	15q26.1	5	89125000	4830	nofs	nofs
chr15	E	15q26.1	5	89375000	3879	nofs	nofs
chr15	E	15q26.1	5	89625000	3560	nofs	nofs
chr15	E	15q26.1	5	89875000	1849	nofs	nofs
chr15	E	15q26.1	5	90125000	4868	nofs	nofs
chr15	E	15q26.1	5	90375000	2309	nofs	nofs
chr15	E	15q26.1	5	90625000	2624	nofs	nofs
chr15	E	15q26.1	5	90875000	2984	nofs	nofs
chr15	E	15q26.1	5	91125000	4460	nofs	nofs
chr15	E	15q26.1	5	91375000	2162	nofs	nofs
chr15	E	15q26.1	5	91625000	5374	nofs	nofs
chr15	E	15q26.1	5	91875000	2678	nofs	nofs
chr15	E	15q26.1	5	92125000	2893	nofs	nofs
chr15	B	15q26.2	.	92375000	4496	nofs	nofs
chr15	B	15q26.2	.	92625000	5477	nofs	nofs
chr15	B	15q26.2	.	92875000	1470	nofs	nofs
chr15	B	15q26.2	.	93125000	2347	nofs	nofs
chr15	B	15q26.2	.	93375000	2638	nofs	nofs
chr15	B	15q26.2	.	93625000	3589	nofs	nofs
chr15	B	15q26.2	.	93875000	2387	nofs	nofs
chr15	B	15q26.2	.	94125000	2831	nofs	nofs
chr15	B	15q26.2	.	94375000	2749	nofs	nofs
chr15	B	15q26.2	.	94625000	2102	nofs	nofs
chr15	B	15q26.2	.	94875000	2591	nofs	nofs
chr15	B	15q26.2	.	95125000	3520	nofs	nofs
chr15	B	15q26.2	.	95375000	3156	nofs	nofs
chr15	B	15q26.2	.	95625000	2463	nofs	nofs
chr15	B	15q26.2	.	95875000	3089	nofs	nofs
chr15	B	15q26.2	.	96125000	3206	nofs	nofs
chr15	E	15q26.3	2	96375000	2640	nofs	nofs
chr15	E	15q26.3	2	96625000	2668	nofs	nofs

chr15	E	15q26.3	2	96875000	4957	nofs	nofs
chr15	T	15q26.3	2	97125000	1854	nofs	nofs
chr15	T	15q26.3	2	97375000	2334	nofs	nofs
chr15	T	15q26.3	2	97625000	1498	nofs	nofs
chr15	T	15q26.3	2	97875000	2848	nofs	nofs
chr15	T	15q26.3	2	98125000	3082	nofs	nofs
chr15	T	15q26.3	2	98375000	4639	nofs	nofs
chr15	T	15q26.3	2	98625000	5457	nofs	nofs
chr15	T	15q26.3	2	98875000	9418	nofs	nofs
chr15	T	15q26.3	2	99125000	5263	nofs	nofs
chr15	T	15q26.3	2	99375000	5963	nofs	nofs
chr15	T	15q26.3	2	99625000	4564	nofs	nofs
chr15	T	15q26.3	2	99875000	3056	nofs	nofs
chr15	T	15q26.3	2	100000000	17056	nofs	nofs
chr16	T	16p13.3	2	125000	9150	nofs	nofs
chr16	T	16p13.3	2	375000	17512	nofs	nofs
chr16	T	16p13.3	2	625000	18581	nofs	nofs
chr16	T	16p13.3	2	875000	32188	nofs	nofs
chr16	T	16p13.3	2	1125000	16849	nofs	nofs
chr16	T	16p13.3	2	1375000	13854	nofs	nofs
chr16	T	16p13.3	2	1625000	13694	nofs	nofs
chr16	T	16p13.3	2	1875000	6380	nofs	nofs
chr16	T	16p13.3	2	2125000	7767	nofs	nofs
chr16	T	16p13.3	2	2375000	6408	nofs	nofs
chr16	T	16p13.3	2	2625000	2479	nofs	nofs
chr16	T	16p13.3	2	2875000	7316	nofs	nofs
chr16	T	16p13.3	2	3125000	8932	nofs	nofs
chr16	E	16p13.3	2	3375000	3383	nofs	nofs
chr16	E	16p13.3	2	3625000	4968	nofs	nofs
chr16	E	16p13.3	2	3875000	4481	nofs	nofs
chr16	E	16p13.3	2	4125000	6884	nofs	nofs
chr16	E	16p13.3	2	4375000	5349	nofs	nofs
chr16	E	16p13.3	2	4625000	6725	nofs	nofs
chr16	E	16p13.3	2	4875000	6097	nofs	nofs
chr16	E	16p13.3	2	5125000	4720	nofs	nofs
chr16	E	16p13.3	2	5375000	3836	nofs	nofs

chr16	E	16p13.3	2	5625000	5202	nofs	nofs
chr16	E	16p13.3	2	5875000	4900	nofs	nofs
chr16	E	16p13.3	2	6125000	4759	nofs	nofs
chr16	E	16p13.3	2	6375000	5671	nofs	nofs
chr16	B	16p13.2	.	6625000	5868	nofs	nofs
chr16	B	16p13.2	.	6875000	6937	nofs	nofs
chr16	B	16p13.2	.	7125000	4897	nofs	nofs
chr16	B	16p13.2	.	7375000	5096	nofs	nofs
chr16	B	16p13.2	.	7625000	2070	nofs	nofs
chr16	B	16p13.2	.	7875000	3950	nofs	nofs
chr16	B	16p13.2	.	8125000	3561	nofs	nofs
chr16	B	16p13.2	.	8375000	4101	nofs	nofs
chr16	B	16p13.2	.	8625000	7637	nofs	nofs
chr16	B	16p13.2	.	8875000	4241	nofs	nofs
chr16	B	16p13.2	.	9125000	5232	nofs	nofs
chr16	B	16p13.2	.	9375000	7068	nofs	nofs
chr16	B	16p13.2	.	9625000	4402	nofs	nofs
chr16	B	16p13.2	.	9875000	3571	nofs	nofs
chr16	B	16p13.2	.	10125000	2970	nofs	nofs
chr16	E	16p13.1	3	10375000	4276	nofs	nofs
chr16	E	16p13.1	3	10625000	18419	nofs	nofs
chr16	E	16p13.1	3	10875000	4097	nofs	nofs
chr16	E	16p13.1	3	11125000	2385	nofs	nofs
chr16	E	16p13.1	3	11375000	4968	nofs	nofs
chr16	E	16p13.1	3	11625000	4203	nofs	nofs
chr16	E	16p13.1	3	11875000	5827	nofs	nofs
chr16	E	16p13.1	3	12125000	4368	nofs	nofs
chr16	E	16p13.1	3	12375000	7392	nofs	nofs
chr16	E	16p13.1	3	12625000	1970	nofs	nofs
chr16	E	16p13.1	3	12875000	4182	nofs	nofs
chr16	E	16p13.1	3	13125000	4906	nofs	nofs
chr16	E	16p13.1	3	13375000	3668	nofs	nofs
chr16	E	16p13.1	3	13625000	3034	nofs	nofs
chr16	E	16p13.1	3	13875000	4993	nofs	nofs
chr16	E	16p13.1	3	14125000	2315	nofs	nofs
chr16	E	16p13.1	3	14375000	4262	nofs	nofs

chr16	E	16p13.1	3	14625000	5393	nofs	nofs
chr16	E	16p13.1	3	14875000	8326	fs	fs rare
chr16	E	16p13.1	3	15125000	5022	fs	fs rare
chr16	E	16p13.1	3	15375000	5611	fs	fs rare
chr16	E	16p13.1	3	15625000	3896	fs	fs rare
chr16	E	16p13.1	3	15875000	5873	fs	fs rare
chr16	E	16p13.1	3	16125000	10450	fs	fs rare
chr16	E	16p13.1	3	16375000	6738	fs	fs rare
chr16	E	16p13.1	3	16625000	4968	fs	fs rare
chr16	E	16p13.1	3	16875000	4980	fs	fs rare
chr16	E	16p12	1	17125000	5337	nofs	nofs
chr16	E	16p12	1	17375000	2723	nofs	nofs
chr16	E	16p12	1	17625000	4779	nofs	nofs
chr16	E	16p12	1	17875000	2953	nofs	nofs
chr16	E	16p12	1	18125000	4761	nofs	nofs
chr16	E	16p12	1	18375000	10070	nofs	nofs
chr16	E	16p12	1	18625000	4615	nofs	nofs
chr16	E	16p12	1	18875000	4580	nofs	nofs
chr16	E	16p12	1	19125000	3665	nofs	nofs
chr16	E	16p12	1	19375000	5317	nofs	nofs
chr16	E	16p12	1	19625000	2756	nofs	nofs
chr16	E	16p12	1	19875000	3496	nofs	nofs
chr16	E	16p12	1	20125000	3703	nofs	nofs
chr16	E	16p12	1	20375000	5126	nofs	nofs
chr16	E	16p12	1	20625000	4504	nofs	nofs
chr16	E	16p12	1	20875000	3473	nofs	nofs
chr16	E	16p12	1	21125000	3513	nofs	nofs
chr16	E	16p12	1	21375000	9473	nofs	nofs
chr16	E	16p12	1	21625000	4084	nofs	nofs
chr16	E	16p12	1	21875000	5239	fs	fs rare
chr16	E	16p12	1	22125000	4502	fs	fs rare
chr16	E	16p12	1	22375000	6261	fs	fs rare
chr16	E	16p12	1	22625000	5042	fs	fs rare
chr16	E	16p12	1	22875000	4161	fs	fs rare
chr16	E	16p12	1	23125000	6191	fs	fs rare
chr16	E	16p12	1	23375000	5633	fs	fs rare

chr16	E	16p12	1	23625000	5490	fs	fs rare
chr16	E	16p12	1	23875000	5980	fs	fs rare
chr16	E	16p12	1	24125000	5577	fs	fs rare
chr16	E	16p12	1	24375000	3985	fs	fs rare
chr16	E	16p12	1	24625000	4153	fs	fs rare
chr16	E	16p12	1	24875000	5697	fs	fs rare
chr16	E	16p12	1	25125000	2886	fs	fs rare
chr16	E	16p12	1	25375000	2339	fs	fs rare
chr16	E	16p12	1	25625000	4422	fs	fs rare
chr16	E	16p12	1	25875000	5220	fs	fs rare
chr16	E	16p12	1	26125000	4683	fs	fs rare
chr16	E	16p12	1	26375000	3941	fs	fs rare
chr16	E	16p12	1	26625000	7682	fs	fs rare
chr16	E	16p12	1	26875000	5610	fs	fs rare
chr16	E	16p12	1	27125000	5494	fs	fs rare
chr16	E	16p12	1	27375000	2242	fs	fs rare
chr16	E	16p12	1	27625000	5566	fs	fs rare
chr16	E	16p11.2	2	27875000	5712	nofs	nofs
chr16	E	16p11.2	2	28125000	5193	nofs	nofs
chr16	E	16p11.2	2	28375000	9934	nofs	nofs
chr16	E	16p11.2	2	28625000	8160	nofs	nofs
chr16	E	16p11.2	2	28875000	7233	nofs	nofs
chr16	E	16p11.2	2	29125000	15367	nofs	nofs
chr16	E	16p11.2	2	29375000	8600	nofs	nofs
chr16	E	16p11.2	2	29625000	7792	nofs	nofs
chr16	E	16p11.2	2	29875000	4139	nofs	nofs
chr16	E	16p11.2	2	30125000	10609	nofs	nofs
chr16	E	16p11.2	2	30375000	6342	nofs	nofs
chr16	E	16p11.2	2	30625000	6245	nofs	nofs
chr16	E	16p11.2	2	30875000	5560	nofs	nofs
chr16	E	16p11.2	2	31125000	9311	nofs	nofs
chr16	E	16p11.2	2	31375000	4570	nofs	nofs
chr16	E	16p11.2	2	31625000	3583	nofs	nofs
chr16	E	16p11.2	2	31875000	6914	nofs	nofs
chr16	C	16p11.2	2	32125000	11798	nofs	nofs
chr16	C	16p11.2	2	32375000	94712	nofs	nofs

chr16	C	16p11.2	2	32625000	19960	nofs	nofs
chr16	C	16p11.2	2	32875000	9670	nofs	nofs
chr16	C	16p11.2	2	33125000	18407	nofs	nofs
chr16	C	16p11.2	2	33375000	7380	nofs	nofs
chr16	C	16p11.2	2	33625000	6377	nofs	nofs
chr16	C	16p11.2	2	33875000	101635	nofs	nofs
chr16	C	16p11.2	2	34125000	18500	nofs	nofs
chr16	C	16p11.2	2	34375000	2472	nofs	nofs
chr16	C	16p11.2	2	34625000	10296	nofs	nofs
chr16	C	16p11.2	2	34875000	58013	nofs	nofs
chr16	C	16p11.2	2	35125000	101204	nofs	nofs
chr16	C		.	35375000	0	nofs	nofs
chr16	C		.	35625000	0	nofs	nofs
chr16	C		.	35875000	0	nofs	nofs
chr16	C		.	36125000	0	nofs	nofs
chr16	C		.	36375000	0	nofs	nofs
chr16	C		.	36625000	0	nofs	nofs
chr16	C		.	36875000	0	nofs	nofs
chr16	C		.	37125000	0	nofs	nofs
chr16	C		.	37375000	0	nofs	nofs
chr16	C		.	37625000	0	nofs	nofs
chr16	C		.	37875000	0	nofs	nofs
chr16	C		.	38125000	0	nofs	nofs
chr16	C		.	38375000	0	nofs	nofs
chr16	C		.	38625000	0	nofs	nofs
chr16	C		.	38875000	0	nofs	nofs
chr16	C		.	39125000	0	nofs	nofs
chr16	C		.	39375000	0	nofs	nofs
chr16	C		.	39625000	0	nofs	nofs
chr16	C		.	39875000	0	nofs	nofs
chr16	C		.	40125000	0	nofs	nofs
chr16	C		.	40375000	0	nofs	nofs
chr16	C		.	40625000	0	nofs	nofs
chr16	C		.	40875000	0	nofs	nofs
chr16	C		.	41125000	0	nofs	nofs
chr16	C		.	41375000	0	nofs	nofs

chr16	C		.	41625000	0	nofs	nofs
chr16	C	16q11	.	41875000	0	nofs	nofs
chr16	C	16q11	.	42125000	0	nofs	nofs
chr16	C	16q11	.	42375000	0	nofs	nofs
chr16	C	16q11	.	42625000	0	nofs	nofs
chr16	C	16q11	.	42875000	0	nofs	nofs
chr16	C	16q11	.	43125000	0	nofs	nofs
chr16	C	16q11	.	43375000	0	nofs	nofs
chr16	C	16q11	.	43625000	0	nofs	nofs
chr16	C	16q11	.	43875000	0	nofs	nofs
chr16	C	16q11	.	44125000	0	nofs	nofs
chr16	C	16q11	.	44375000	0	nofs	nofs
chr16	C	16q11	.	44625000	0	nofs	nofs
chr16	C	16q12.1	1	44875000	56484	nofs	nofs
chr16	C	16q12.1	1	45125000	20250	nofs	nofs
chr16	C	16q12.1	1	45375000	5047	nofs	nofs
chr16	C	16q12.1	1	45625000	4899	nofs	nofs
chr16	C	16q12.1	1	45875000	2701	nofs	nofs
chr16	C	16q12.1	1	46125000	2834	nofs	nofs
chr16	C	16q12.1	1	46375000	2979	nofs	nofs
chr16	C	16q12.1	1	46625000	3508	nofs	nofs
chr16	C	16q12.1	1	46875000	2233	nofs	nofs
chr16	C	16q12.1	1	47125000	4137	nofs	nofs
chr16	C	16q12.1	1	47375000	2681	nofs	nofs
chr16	C	16q12.1	1	47625000	6349	nofs	nofs
chr16	C	16q12.1	1	47875000	2679	nofs	nofs
chr16	E	16q12.1	1	48125000	5919	nofs	nofs
chr16	E	16q12.1	1	48375000	4766	nofs	nofs
chr16	E	16q12.1	1	48625000	3832	nofs	nofs
chr16	E	16q12.1	1	48875000	1942	nofs	nofs
chr16	E	16q12.1	1	49125000	5258	nofs	nofs
chr16	E	16q12.1	1	49375000	2496	nofs	nofs
chr16	E	16q12.1	1	49625000	4942	nofs	nofs
chr16	E	16q12.1	1	49875000	3782	nofs	nofs
chr16	E	16q12.1	1	50125000	2795	nofs	nofs
chr16	E	16q12.1	1	50375000	2873	nofs	nofs

chr16	E	16q12.1	1	50625000	2403	nofs	nofs
chr16	E	16q12.1	1	50875000	3872	nofs	nofs
chr16	E	16q12.1	1	51125000	2356	nofs	nofs
chr16	E	16q12.2	1	51375000	4127	nofs	nofs
chr16	E	16q12.2	1	51625000	4182	nofs	nofs
chr16	E	16q12.2	1	51875000	1950	nofs	nofs
chr16	E	16q12.2	1	52125000	3589	nofs	nofs
chr16	E	16q12.2	1	52375000	2817	nofs	nofs
chr16	E	16q12.2	1	52625000	2410	nofs	nofs
chr16	E	16q12.2	1	52875000	3592	nofs	nofs
chr16	E	16q12.2	1	53125000	2649	nofs	nofs
chr16	E	16q12.2	1	53375000	3676	nofs	nofs
chr16	E	16q12.2	1	53625000	3377	nofs	nofs
chr16	E	16q12.2	1	53875000	2631	nofs	nofs
chr16	E	16q12.2	1	54125000	3156	nofs	nofs
chr16	E	16q12.2	1	54375000	5809	nofs	nofs
chr16	E	16q12.2	1	54625000	3452	nofs	nofs
chr16	E	16q12.2	1	54875000	2317	nofs	nofs
chr16	E	16q12.2	1	55125000	2825	nofs	nofs
chr16	E	16q13	2	55375000	4945	nofs	nofs
chr16	E	16q13	2	55625000	5117	nofs	nofs
chr16	E	16q13	2	55875000	6678	nofs	nofs
chr16	E	16q13	2	56125000	5407	nofs	nofs
chr16	E	16q13	2	56375000	7535	nofs	nofs
chr16	E	16q13	2	56625000	5271	nofs	nofs
chr16	E	16q13	2	56875000	4874	nofs	nofs
chr16	E	16q21	2	57125000	4189	nofs	nofs
chr16	E	16q21	2	57375000	4881	nofs	nofs
chr16	E	16q21	2	57625000	4077	nofs	nofs
chr16	E	16q21	2	57875000	2953	nofs	nofs
chr16	E	16q21	2	58125000	2557	nofs	nofs
chr16	E	16q21	2	58375000	2860	nofs	nofs
chr16	E	16q21	2	58625000	2379	nofs	nofs
chr16	E	16q21	2	58875000	4680	nofs	nofs
chr16	E	16q21	2	59125000	2867	nofs	nofs
chr16	E	16q21	2	59375000	4599	nofs	nofs

chr16	E	16q21	2	59625000	2756	nofs	nofs
chr16	E	16q21	2	59875000	2054	nofs	nofs
chr16	E	16q21	2	60125000	4364	nofs	nofs
chr16	E	16q21	2	60375000	2625	nofs	nofs
chr16	E	16q21	2	60625000	4430	nofs	nofs
chr16	E	16q21	2	60875000	2899	nofs	nofs
chr16	E	16q21	2	61125000	2282	nofs	nofs
chr16	E	16q21	2	61375000	2423	nofs	nofs
chr16	E	16q21	2	61625000	3453	nofs	nofs
chr16	E	16q21	2	61875000	2815	nofs	nofs
chr16	E	16q21	2	62125000	2870	nofs	nofs
chr16	E	16q21	2	62375000	2057	nofs	nofs
chr16	E	16q21	2	62625000	3077	nofs	nofs
chr16	E	16q21	2	62875000	2912	nofs	nofs
chr16	E	16q21	2	63125000	3323	nofs	nofs
chr16	E	16q21	2	63375000	2752	nofs	nofs
chr16	E	16q21	2	63625000	4298	nofs	nofs
chr16	E	16q21	2	63875000	3477	nofs	nofs
chr16	E	16q22	5	64125000	4081	fs	fs common and rare
chr16	E	16q22	5	64375000	2896	fs	fs common and rare
chr16	E	16q22	5	64625000	3080	fs	fs common and rare
chr16	E	16q22	5	64875000	3639	fs	fs common and rare
chr16	E	16q22	5	65125000	3655	fs	fs common and rare
chr16	E	16q22	5	65375000	7021	fs	fs common and rare
chr16	E	16q22	5	65625000	4314	fs	fs common and rare
chr16	E	16q22	5	65875000	4260	fs	fs common and rare
chr16	E	16q22	5	66125000	4486	fs	fs common and rare
chr16	E	16q22	5	66375000	2809	fs	fs common and rare
chr16	E	16q22	5	66625000	3666	fs	fs common and rare
chr16	E	16q22	5	66875000	2646	fs	fs common and rare
chr16	E	16q22	5	67125000	3920	fs	fs common and rare
chr16	E	16q22	5	67375000	3241	fs	fs common and rare
chr16	E	16q22	5	67625000	3544	fs	fs common and rare
chr16	E	16q22	5	67875000	3300	fs	fs common and rare
chr16	E	16q22	5	68125000	3522	fs	fs common and rare
chr16	E	16q22	5	68375000	2581	fs	fs common and rare

chr16	E	16q22	5	68625000	4873	fs	fs common and rare
chr16	E	16q22	5	68875000	7063	fs	fs common and rare
chr16	E	16q22	5	69125000	4508	fs	fs common and rare
chr16	E	16q22	5	69375000	2737	fs	fs common and rare
chr16	E	16q22	5	69625000	2106	nofs	nofs
chr16	E	16q22	5	69875000	3244	nofs	nofs
chr16	E	16q22	5	70125000	4404	nofs	nofs
chr16	E	16q22	5	70375000	3148	nofs	nofs
chr16	E	16q22	5	70625000	2286	nofs	nofs
chr16	E	16q22	5	70875000	3200	nofs	nofs
chr16	E	16q22	5	71125000	2575	nofs	nofs
chr16	E	16q22	5	71375000	2346	nofs	nofs
chr16	E	16q22	5	71625000	2957	nofs	nofs
chr16	E	16q22	5	71875000	5157	nofs	nofs
chr16	E	16q22	5	72125000	3033	nofs	nofs
chr16	E	16q22	5	72375000	2640	nofs	nofs
chr16	E	16q22	5	72625000	4576	nofs	nofs
chr16	E	16q22	5	72875000	3838	nofs	nofs
chr16	E	16q22	5	73125000	4234	nofs	nofs
chr16	E	16q23	4	73375000	2947	nofs	nofs
chr16	E	16q23	4	73625000	2298	nofs	nofs
chr16	E	16q23	4	73875000	2805	nofs	nofs
chr16	E	16q23	4	74125000	3135	nofs	nofs
chr16	E	16q23	4	74375000	3472	nofs	nofs
chr16	E	16q23	4	74625000	2571	nofs	nofs
chr16	E	16q23	4	74875000	2691	nofs	nofs
chr16	E	16q23	4	75125000	2939	nofs	nofs
chr16	E	16q23	4	75375000	1515	nofs	nofs
chr16	E	16q23	4	75625000	2469	nofs	nofs
chr16	E	16q23	4	75875000	3051	nofs	nofs
chr16	E	16q23	4	76125000	1586	nofs	nofs
chr16	E	16q23	4	76375000	10757	nofs	nofs
chr16	E	16q23	4	76625000	2692	nofs	nofs
chr16	E	16q23	4	76875000	3638	nofs	nofs
chr16	E	16q23	4	77125000	2611	nofs	nofs
chr16	E	16q23	4	77375000	3311	nofs	nofs

chr16	E	16q23	4	77625000	3479	nofs	nofs
chr16	E	16q23	4	77875000	4347	nofs	nofs
chr16	E	16q23	4	78125000	3671	nofs	nofs
chr16	E	16q23	4	78375000	4095	nofs	nofs
chr16	E	16q23	4	78625000	2534	fs	fs common
chr16	E	16q23	4	78875000	4662	fs	fs common
chr16	E	16q23	4	79125000	4204	fs	fs common
chr16	E	16q23	4	79375000	3743	fs	fs common
chr16	E	16q23	4	79625000	2359	fs	fs common
chr16	E	16q23	4	79875000	4338	fs	fs common
chr16	E	16q23	4	80125000	7014	fs	fs common
chr16	E	16q23	4	80375000	6783	fs	fs common
chr16	E	16q23	4	80625000	3500	fs	fs common
chr16	E	16q23	4	80875000	4957	nofs	nofs
chr16	E	16q23	4	81125000	2075	nofs	nofs
chr16	E	16q23	4	81375000	2660	nofs	nofs
chr16	E	16q23	4	81625000	2760	nofs	nofs
chr16	E	16q23	4	81875000	2263	nofs	nofs
chr16	E	16q23	4	82125000	4783	nofs	nofs
chr16	E	16q23	4	82375000	3644	nofs	nofs
chr16	E	16q23	4	82625000	10877	nofs	nofs
chr16	B	16q24	.	82875000	4005	nofs	nofs
chr16	B	16q24	.	83125000	4198	nofs	nofs
chr16	B	16q24	.	83375000	3944	nofs	nofs
chr16	B	16q24	.	83625000	5874	nofs	nofs
chr16	B	16q24	.	83875000	12829	nofs	nofs
chr16	B	16q24	.	84125000	10709	nofs	nofs
chr16	B	16q24	.	84375000	8772	nofs	nofs
chr16	B	16q24	.	84625000	10178	nofs	nofs
chr16	B	16q24	.	84875000	8495	nofs	nofs
chr16	B	16q24	.	85125000	6494	nofs	nofs
chr16	B	16q24	.	85375000	11882	nofs	nofs
chr16	T	16q24	.	85625000	10026	nofs	nofs
chr16	T	16q24	.	85875000	9034	nofs	nofs
chr16	T	16q24	.	86125000	10161	nofs	nofs
chr16	T	16q24	.	86375000	13907	nofs	nofs

chr16	T	16q24	.	86625000	17174	nofs	nofs
chr16	T	16q24	.	86875000	28651	nofs	nofs
chr16	T	16q24	.	87125000	16926	nofs	nofs
chr16	T	16q24	.	87375000	14038	nofs	nofs
chr16	T	16q24	.	87625000	34844	nofs	nofs
chr16	T	16q24	.	87875000	20925	nofs	nofs
chr16	T	16q24	.	88125000	23746	nofs	nofs
chr16	T	16q24	.	88375000	18823	nofs	nofs
chr16	T	16q24	.	88625000	16251	nofs	nofs
chr17	T	17p13	4	125000	45186	nofs	nofs
chr17	T	17p13	4	375000	35790	nofs	nofs
chr17	T	17p13	4	625000	23393	nofs	nofs
chr17	T	17p13	4	875000	27540	nofs	nofs
chr17	T	17p13	4	1125000	20324	nofs	nofs
chr17	T	17p13	4	1375000	7217	nofs	nofs
chr17	T	17p13	4	1625000	8970	nofs	nofs
chr17	T	17p13	4	1875000	6793	nofs	nofs
chr17	T	17p13	4	2125000	7415	nofs	nofs
chr17	T	17p13	4	2375000	7025	nofs	nofs
chr17	T	17p13	4	2625000	5499	nofs	nofs
chr17	T	17p13	4	2875000	6721	nofs	nofs
chr17	T	17p13	4	3125000	4108	nofs	nofs
chr17	E	17p13	4	3375000	6325	nofs	nofs
chr17	E	17p13	4	3625000	10679	nofs	nofs
chr17	E	17p13	4	3875000	6981	nofs	nofs
chr17	E	17p13	4	4125000	6516	nofs	nofs
chr17	E	17p13	4	4375000	6604	nofs	nofs
chr17	E	17p13	4	4625000	7140	nofs	nofs
chr17	E	17p13	4	4875000	8503	nofs	nofs
chr17	E	17p13	4	5125000	6204	nofs	nofs
chr17	E	17p13	4	5375000	3319	nofs	nofs
chr17	E	17p13	4	5625000	5445	nofs	nofs
chr17	E	17p13	4	5875000	3561	nofs	nofs
chr17	E	17p13	4	6125000	5775	nofs	nofs
chr17	E	17p13	4	6375000	6587	nofs	nofs
chr17	E	17p13	4	6625000	4537	nofs	nofs

chr17	E	17p13	4	6875000	4817	nofs	nofs
chr17	E	17p13	4	7125000	5165	nofs	nofs
chr17	E	17p13	4	7375000	5118	nofs	nofs
chr17	E	17p13	4	7625000	3982	nofs	nofs
chr17	E	17p13	4	7875000	3287	nofs	nofs
chr17	E	17p13	4	8125000	5559	nofs	nofs
chr17	E	17p13	4	8375000	2895	nofs	nofs
chr17	E	17p13	4	8625000	7158	nofs	nofs
chr17	E	17p13	4	8875000	4133	nofs	nofs
chr17	E	17p13	4	9125000	4773	nofs	nofs
chr17	E	17p13	4	9375000	4552	nofs	nofs
chr17	E	17p13	4	9625000	5076	nofs	nofs
chr17	E	17p13	4	9875000	4721	nofs	nofs
chr17	E	17p13	4	10125000	3658	nofs	nofs
chr17	E	17p13	4	10375000	3204	nofs	nofs
chr17	E	17p13	4	10625000	3695	nofs	nofs
chr17	E	17p12	1	10875000	2695	fs	fs rare
chr17	E	17p12	1	11125000	3408	fs	fs rare
chr17	E	17p12	1	11375000	3360	fs	fs rare
chr17	E	17p12	1	11625000	3451	fs	fs rare
chr17	E	17p12	1	11875000	3579	fs	fs rare
chr17	E	17p12	1	12125000	5818	fs	fs rare
chr17	E	17p12	1	12375000	5037	fs	fs rare
chr17	E	17p12	1	12625000	3686	fs	fs rare
chr17	E	17p12	1	12875000	3355	fs	fs rare
chr17	E	17p12	1	13125000	3903	fs	fs rare
chr17	E	17p12	1	13375000	5264	fs	fs rare
chr17	E	17p12	1	13625000	5692	fs	fs rare
chr17	E	17p12	1	13875000	2212	fs	fs rare
chr17	E	17p12	1	14125000	2069	fs	fs rare
chr17	E	17p12	1	14375000	3887	fs	fs rare
chr17	E	17p12	1	14625000	1805	fs	fs rare
chr17	E	17p12	1	14875000	1877	fs	fs rare
chr17	E	17p12	1	15125000	2506	fs	fs rare
chr17	E	17p12	1	15375000	4472	fs	fs rare
chr17	E	17p12	1	15625000	4157	fs	fs rare

chr17	E	17p12	1	15875000	5086	fs	fs rare
chr17	E	17p11.2	4	16125000	5977	nofs	nofs
chr17	E	17p11.2	4	16375000	4233	nofs	nofs
chr17	E	17p11.2	4	16625000	2611	nofs	nofs
chr17	E	17p11.2	4	16875000	3046	nofs	nofs
chr17	E	17p11.2	4	17125000	5289	nofs	nofs
chr17	E	17p11.2	4	17375000	6010	nofs	nofs
chr17	E	17p11.2	4	17625000	2528	nofs	nofs
chr17	E	17p11.2	4	17875000	5349	nofs	nofs
chr17	E	17p11.2	4	18125000	4169	nofs	nofs
chr17	E	17p11.2	4	18375000	2688	nofs	nofs
chr17	E	17p11.2	4	18625000	3877	nofs	nofs
chr17	C	17p11.2	4	18875000	4739	nofs	nofs
chr17	C	17p11.2	4	19125000	5641	nofs	nofs
chr17	C	17p11.2	4	19375000	5246	nofs	nofs
chr17	C	17p11.2	4	19625000	5056	nofs	nofs
chr17	C	17p11.2	4	19875000	3641	nofs	nofs
chr17	C	17p11.2	4	20125000	1892	nofs	nofs
chr17	C	17p11.2	4	20375000	4257	nofs	nofs
chr17	C	17p11.2	4	20625000	4671	nofs	nofs
chr17	C	17p11.2	4	20875000	5825	nofs	nofs
chr17	C	17p11.2	4	21125000	4016	nofs	nofs
chr17	C	17p11.2	4	21375000	3852	nofs	nofs
chr17	C	17p11.1	.	21375000	3852	nofs	nofs
chr17	C	17p11.1	.	21625000	31117	nofs	nofs
chr17	C		.	21875000	3709	nofs	nofs
chr17	C		.	22125000	54097	nofs	nofs
chr17	C		.	22375000	8711	nofs	nofs
chr17	C	17q11.1	.	22625000	26410	nofs	nofs
chr17	C	17q11.1	.	22875000	2578	nofs	nofs
chr17	C	17q11.1	.	23125000	2027	nofs	nofs
chr17	C	17q11.2	5	23375000	2729	nofs	nofs
chr17	C	17q11.2	5	23625000	5549	nofs	nofs
chr17	C	17q11.2	5	23875000	8689	nofs	nofs
chr17	C	17q11.2	5	24125000	5199	nofs	nofs
chr17	C	17q11.2	5	24375000	2346	nofs	nofs

chr17	C	17q11.2	5	24625000	4666	nofs	nofs
chr17	C	17q11.2	5	24875000	3228	nofs	nofs
chr17	C	17q11.2	5	25125000	5068	nofs	nofs
chr17	C	17q11.2	5	25375000	3087	nofs	nofs
chr17	C	17q11.2	5	25625000	4125	nofs	nofs
chr17	E	17q11.2	5	25875000	3253	nofs	nofs
chr17	E	17q11.2	5	26125000	6641	nofs	nofs
chr17	E	17q11.2	5	26375000	6084	nofs	nofs
chr17	E	17q11.2	5	26625000	1769	nofs	nofs
chr17	E	17q11.2	5	26875000	3983	nofs	nofs
chr17	E	17q11.2	5	27125000	4955	nofs	nofs
chr17	E	17q11.2	5	27375000	7171	nofs	nofs
chr17	E	17q11.2	5	27625000	5813	nofs	nofs
chr17	E	17q11.2	5	27875000	3228	nofs	nofs
chr17	E	17q11.2	5	28125000	1912	nofs	nofs
chr17	E	17q11.2	5	28375000	4831	nofs	nofs
chr17	E	17q11.2	5	28625000	3566	nofs	nofs
chr17	E	17q11.2	5	28875000	2366	nofs	nofs
chr17	E	17q12	3	29125000	2572	nofs	nofs
chr17	E	17q12	3	29375000	2684	nofs	nofs
chr17	E	17q12	3	29625000	2509	nofs	nofs
chr17	E	17q12	3	29875000	2255	nofs	nofs
chr17	E	17q12	3	30125000	3432	nofs	nofs
chr17	E	17q12	3	30375000	1876	nofs	nofs
chr17	E	17q12	3	30625000	3319	nofs	nofs
chr17	E	17q12	3	30875000	4047	nofs	nofs
chr17	E	17q12	3	31125000	2240	nofs	nofs
chr17	E	17q12	3	31375000	3868	nofs	nofs
chr17	E	17q12	3	31625000	2187	nofs	nofs
chr17	E	17q12	3	31875000	1604	nofs	nofs
chr17	E	17q12	3	32125000	4919	nofs	nofs
chr17	E	17q12	3	32375000	4142	nofs	nofs
chr17	E	17q12	3	32625000	1971	nofs	nofs
chr17	E	17q12	3	32875000	3275	nofs	nofs
chr17	E	17q12	3	33125000	2376	nofs	nofs
chr17	E	17q12	3	33375000	2874	nofs	nofs

chr17	E	17q12	3	33625000	4870	nofs	nofs
chr17	E	17q12	3	33875000	3860	nofs	nofs
chr17	E	17q12	3	34125000	5823	nofs	nofs
chr17	E	17q12	3	34375000	5633	nofs	nofs
chr17	E	17q12	3	34625000	6433	nofs	nofs
chr17	E	17q12	3	34875000	5536	nofs	nofs
chr17	E	17q12	3	35125000	3364	nofs	nofs
chr17	E	17q21.1	1	35375000	6649	nofs	nofs
chr17	E	17q21.1	1	35625000	7172	nofs	nofs
chr17	E	17q21.2	3	35875000	2369	nofs	nofs
chr17	E	17q21.2	3	36125000	2291	nofs	nofs
chr17	E	17q21.2	3	36375000	6509	nofs	nofs
chr17	E	17q21.2	3	36625000	9276	nofs	nofs
chr17	E	17q21.2	3	36875000	2926	nofs	nofs
chr17	E	17q21.2	3	37125000	7801	nofs	nofs
chr17	E	17q21.2	3	37375000	6817	nofs	nofs
chr17	E	17q21.2	3	37625000	5489	nofs	nofs
chr17	E	17q21.2	3	37875000	6145	nofs	nofs
chr17	E	17q21.2	3	38125000	5559	nofs	nofs
chr17	E	17q21.2	3	38375000	6675	nofs	nofs
chr17	E	17q21.3	6	38625000	4018	nofs	nofs
chr17	E	17q21.3	6	38875000	6026	nofs	nofs
chr17	E	17q21.3	6	39125000	3812	nofs	nofs
chr17	E	17q21.3	6	39375000	5484	nofs	nofs
chr17	E	17q21.3	6	39625000	6682	nofs	nofs
chr17	E	17q21.3	6	39875000	5132	nofs	nofs
chr17	E	17q21.3	6	40125000	5074	nofs	nofs
chr17	E	17q21.3	6	40375000	8049	nofs	nofs
chr17	E	17q21.3	6	40625000	5162	nofs	nofs
chr17	E	17q21.3	6	40875000	4282	nofs	nofs
chr17	E	17q21.3	6	41125000	3935	nofs	nofs
chr17	E	17q21.3	6	41375000	4304	nofs	nofs
chr17	E	17q21.3	6	41625000	4471	nofs	nofs
chr17	E	17q21.3	6	41875000	2382	nofs	nofs
chr17	E	17q21.3	6	42125000	2701	nofs	nofs
chr17	E	17q21.3	6	42375000	2841	nofs	nofs

chr17	E	17q21.3	6	42625000	3011	nofs	nofs
chr17	E	17q21.3	6	42875000	5914	nofs	nofs
chr17	E	17q21.3	6	43125000	2683	nofs	nofs
chr17	E	17q21.3	6	43375000	2722	nofs	nofs
chr17	E	17q21.3	6	43625000	3935	nofs	nofs
chr17	E	17q21.3	6	43875000	3048	nofs	nofs
chr17	E	17q21.3	6	44125000	5486	nofs	nofs
chr17	E	17q21.3	6	44375000	7075	nofs	nofs
chr17	E	17q21.3	6	44625000	5116	nofs	nofs
chr17	E	17q21.3	6	44875000	3559	nofs	nofs
chr17	E	17q21.3	6	45125000	2743	nofs	nofs
chr17	E	17q21.3	6	45375000	3475	nofs	nofs
chr17	E	17q21.3	6	45625000	3674	nofs	nofs
chr17	E	17q21.3	6	45875000	2173	nofs	nofs
chr17	E	17q21.3	6	46125000	2945	nofs	nofs
chr17	E	17q21.3	6	46375000	4299	nofs	nofs
chr17	E	17q21.3	6	46625000	4940	nofs	nofs
chr17	E	17q21.3	6	46875000	1798	nofs	nofs
chr17	E	17q21.3	6	47125000	2516	nofs	nofs
chr17	E	17q21.3	6	47375000	1852	nofs	nofs
chr17	E	17q21.3	6	47625000	2268	nofs	nofs
chr17	B	17q22	.	47875000	2123	nofs	nofs
chr17	B	17q22	.	48125000	2579	nofs	nofs
chr17	B	17q22	.	48375000	2188	nofs	nofs
chr17	B	17q22	.	48625000	2132	nofs	nofs
chr17	B	17q22	.	48875000	3234	nofs	nofs
chr17	B	17q22	.	49125000	2094	nofs	nofs
chr17	B	17q22	.	49375000	2136	nofs	nofs
chr17	B	17q22	.	49625000	3032	nofs	nofs
chr17	B	17q22	.	49875000	2038	nofs	nofs
chr17	B	17q22	.	50125000	1599	nofs	nofs
chr17	B	17q22	.	50375000	1990	nofs	nofs
chr17	B	17q22	.	50625000	1619	nofs	nofs
chr17	B	17q22	.	50875000	2868	nofs	nofs
chr17	E	17q23	5	51125000	2810	fs	fs common
chr17	E	17q23	5	51375000	2160	fs	fs common

chr17	E	17q23	5	51625000	2821	fs	fs common
chr17	E	17q23	5	51875000	1808	nofs	nofs
chr17	E	17q23	5	52125000	7193	nofs	nofs
chr17	E	17q23	5	52375000	4348	nofs	nofs
chr17	E	17q23	5	52625000	2866	nofs	nofs
chr17	E	17q23	5	52875000	2309	nofs	nofs
chr17	E	17q23	5	53125000	2688	nofs	nofs
chr17	E	17q23	5	53375000	2968	nofs	nofs
chr17	E	17q23	5	53625000	4494	nofs	nofs
chr17	E	17q23	5	53875000	2328	nofs	nofs
chr17	E	17q23	5	54125000	4752	nofs	nofs
chr17	E	17q23	5	54375000	3696	nofs	nofs
chr17	E	17q23	5	54625000	3984	nofs	nofs
chr17	E	17q23	5	54875000	4667	nofs	nofs
chr17	E	17q23	5	55125000	2584	nofs	nofs
chr17	E	17q23	5	55375000	6556	nofs	nofs
chr17	E	17q23	5	55625000	3274	nofs	nofs
chr17	E	17q23	5	55875000	5632	nofs	nofs
chr17	E	17q23	5	56125000	4767	nofs	nofs
chr17	E	17q23	5	56375000	1708	nofs	nofs
chr17	E	17q23	5	56625000	1859	nofs	nofs
chr17	E	17q23	5	56875000	3168	nofs	nofs
chr17	E	17q23	5	57125000	2556	nofs	nofs
chr17	E	17q23	5	57375000	2132	nofs	nofs
chr17	E	17q23	5	57625000	3849	nofs	nofs
chr17	E	17q23	5	57875000	2934	nofs	nofs
chr17	E	17q23	5	58125000	4941	nofs	nofs
chr17	E	17q23	5	58375000	2810	nofs	nofs
chr17	E	17q23	5	58625000	2056	nofs	nofs
chr17	E	17q23	5	58875000	4370	nofs	nofs
chr17	E	17q23	5	59125000	1673	nofs	nofs
chr17	E	17q23	5	59375000	4244	nofs	nofs
chr17	E	17q23	5	59625000	2709	nofs	nofs
chr17	E	17q23	5	59875000	3217	nofs	nofs
chr17	E	17q24	7	60125000	5545	nofs	nofs
chr17	E	17q24	7	60375000	3324	nofs	nofs

chr17	E	17q24	7	60625000	4750	nofs	nofs
chr17	E	17q24	7	60875000	6379	nofs	nofs
chr17	E	17q24	7	61125000	3256	nofs	nofs
chr17	E	17q24	7	61375000	4029	nofs	nofs
chr17	E	17q24	7	61625000	3430	nofs	nofs
chr17	E	17q24	7	61875000	2545	nofs	nofs
chr17	E	17q24	7	62125000	3674	nofs	nofs
chr17	E	17q24	7	62375000	4001	nofs	nofs
chr17	E	17q24	7	62625000	5864	nofs	nofs
chr17	E	17q24	7	62875000	4368	nofs	nofs
chr17	E	17q24	7	63125000	4159	nofs	nofs
chr17	E	17q24	7	63375000	4728	nofs	nofs
chr17	E	17q24	7	63625000	4115	nofs	nofs
chr17	E	17q24	7	63875000	3462	nofs	nofs
chr17	E	17q24	7	64125000	3982	nofs	nofs
chr17	E	17q24	7	64375000	3705	nofs	nofs
chr17	E	17q24	7	64625000	2976	nofs	nofs
chr17	E	17q24	7	64875000	3409	nofs	nofs
chr17	E	17q24	7	65125000	3327	nofs	nofs
chr17	E	17q24	7	65375000	3174	nofs	nofs
chr17	E	17q24	7	65625000	3784	nofs	nofs
chr17	E	17q24	7	65875000	2531	nofs	nofs
chr17	E	17q24	7	66125000	1942	nofs	nofs
chr17	E	17q24	7	66375000	5042	nofs	nofs
chr17	E	17q24	7	66625000	3932	nofs	nofs
chr17	E	17q24	7	66875000	4069	nofs	nofs
chr17	E	17q24	7	67125000	6075	nofs	nofs
chr17	E	17q24	7	67375000	3946	nofs	nofs
chr17	E	17q24	7	67625000	4740	nofs	nofs
chr17	E	17q24	7	67875000	3163	nofs	nofs
chr17	E	17q24	7	68125000	3718	nofs	nofs
chr17	E	17q24	7	68375000	3448	nofs	nofs
chr17	E	17q25	1	68625000	4743	nofs	nofs
chr17	E	17q25	1	68875000	4397	nofs	nofs
chr17	E	17q25	1	69125000	7285	nofs	nofs
chr17	E	17q25	1	69375000	3368	nofs	nofs

chr17	E	17q25	1	69625000	3797	nofs	nofs
chr17	E	17q25	1	69875000	4608	nofs	nofs
chr17	E	17q25	1	70125000	8546	nofs	nofs
chr17	E	17q25	1	70375000	5079	nofs	nofs
chr17	E	17q25	1	70625000	5230	nofs	nofs
chr17	E	17q25	1	70875000	7916	nofs	nofs
chr17	E	17q25	1	71125000	5673	nofs	nofs
chr17	E	17q25	1	71375000	5868	nofs	nofs
chr17	E	17q25	1	71625000	4369	nofs	nofs
chr17	E	17q25	1	71875000	5543	nofs	nofs
chr17	E	17q25	1	72125000	6749	nofs	nofs
chr17	E	17q25	1	72375000	4533	nofs	nofs
chr17	E	17q25	1	72625000	3991	nofs	nofs
chr17	E	17q25	1	72875000	8590	nofs	nofs
chr17	E	17q25	1	73125000	8285	nofs	nofs
chr17	E	17q25	1	73375000	9739	nofs	nofs
chr17	E	17q25	1	73625000	6585	nofs	nofs
chr17	E	17q25	1	73875000	6783	nofs	nofs
chr17	E	17q25	1	74125000	4716	nofs	nofs
chr17	E	17q25	1	74375000	4394	nofs	nofs
chr17	E	17q25	1	74625000	6929	nofs	nofs
chr17	E	17q25	1	74875000	9186	nofs	nofs
chr17	E	17q25	1	75125000	7588	nofs	nofs
chr17	T	17q25	1	75375000	6983	nofs	nofs
chr17	T	17q25	1	75625000	5766	nofs	nofs
chr17	T	17q25	1	75875000	11521	nofs	nofs
chr17	T	17q25	1	76125000	12712	nofs	nofs
chr17	T	17q25	1	76375000	14960	nofs	nofs
chr17	T	17q25	1	76625000	11209	nofs	nofs
chr17	T	17q25	1	76875000	6988	nofs	nofs
chr17	T	17q25	1	77125000	7605	nofs	nofs
chr17	T	17q25	1	77375000	4341	nofs	nofs
chr17	T	17q25	1	77625000	15617	nofs	nofs
chr17	T	17q25	1	77875000	20154	nofs	nofs
chr17	T	17q25	1	78125000	13774	nofs	nofs
chr17	T	17q25	1	78375000	18989	nofs	nofs

chr18	T	18p11.32	1	125000	12930	nofs	nofs
chr18	T	18p11.32	1	375000	1722	nofs	nofs
chr18	T	18p11.32	1	625000	5835	nofs	nofs
chr18	T	18p11.32	1	875000	2758	nofs	nofs
chr18	T	18p11.32	1	1125000	2487	nofs	nofs
chr18	T	18p11.32	1	1375000	2576	nofs	nofs
chr18	T	18p11.32	1	1625000	4335	nofs	nofs
chr18	T	18p11.32	1	1875000	3506	nofs	nofs
chr18	T	18p11.32	1	2125000	1888	nofs	nofs
chr18	T	18p11.32	1	2375000	2349	nofs	nofs
chr18	T	18p11.32	1	2625000	2479	nofs	nofs
chr18	T	18p11.32	1	2875000	2898	nofs	nofs
chr18	T	18p11.31	.	3125000	2770	nofs	nofs
chr18	B	18p11.31	.	3375000	2444	nofs	nofs
chr18	B	18p11.31	.	3625000	6192	nofs	nofs
chr18	B	18p11.31	.	3875000	2421	nofs	nofs
chr18	B	18p11.31	.	4125000	2403	nofs	nofs
chr18	B	18p11.31	.	4375000	1698	nofs	nofs
chr18	B	18p11.31	.	4625000	2373	nofs	nofs
chr18	B	18p11.31	.	4875000	1803	nofs	nofs
chr18	B	18p11.31	.	5125000	2526	nofs	nofs
chr18	B	18p11.31	.	5375000	3813	nofs	nofs
chr18	B	18p11.31	.	5625000	2187	nofs	nofs
chr18	B	18p11.31	.	5875000	2396	nofs	nofs
chr18	B	18p11.31	.	6125000	2024	nofs	nofs
chr18	B	18p11.31	.	6375000	3729	nofs	nofs
chr18	B	18p11.31	.	6625000	2263	nofs	nofs
chr18	B	18p11.31	.	6875000	2886	nofs	nofs
chr18	E	18p11.2	4	7125000	3691	nofs	nofs
chr18	E	18p11.2	4	7375000	3121	nofs	nofs
chr18	E	18p11.2	4	7625000	1939	nofs	nofs
chr18	E	18p11.2	4	7875000	2428	nofs	nofs
chr18	E	18p11.2	4	8125000	1890	nofs	nofs
chr18	E	18p11.2	4	8375000	2012	nofs	nofs
chr18	E	18p11.2	4	8625000	2623	nofs	nofs
chr18	E	18p11.2	4	8875000	2303	nofs	nofs

chr18	E	18p11.2	4	9125000	2516	nofs	nofs
chr18	E	18p11.2	4	9375000	4449	nofs	nofs
chr18	E	18p11.2	4	9625000	2575	nofs	nofs
chr18	E	18p11.2	4	9875000	6086	nofs	nofs
chr18	E	18p11.2	4	10125000	2548	nofs	nofs
chr18	E	18p11.2	4	10375000	3814	nofs	nofs
chr18	E	18p11.2	4	10625000	4066	nofs	nofs
chr18	E	18p11.2	4	10875000	3482	nofs	nofs
chr18	E	18p11.2	4	11125000	4512	nofs	nofs
chr18	E	18p11.2	4	11375000	2202	nofs	nofs
chr18	E	18p11.2	4	11625000	6405	nofs	nofs
chr18	E	18p11.2	4	11875000	7884	nofs	nofs
chr18	E	18p11.2	4	12125000	3343	nofs	nofs
chr18	C	18p11.2	4	12375000	5219	nofs	nofs
chr18	C	18p11.2	4	12625000	3127	nofs	nofs
chr18	C	18p11.2	4	12875000	6536	nofs	nofs
chr18	C	18p11.2	4	13125000	2885	nofs	nofs
chr18	C	18p11.2	4	13375000	6296	nofs	nofs
chr18	C	18p11.2	4	13625000	6094	nofs	nofs
chr18	C	18p11.2	4	13875000	3506	nofs	nofs
chr18	C	18p11.2	4	14125000	7056	nofs	nofs
chr18	C	18p11.2	4	14375000	2793	nofs	nofs
chr18	C	18p11.2	4	14625000	4082	nofs	nofs
chr18	C	18p11.2	4	14875000	8463	nofs	nofs
chr18	C	18p11.2	4	15125000	15501	nofs	nofs
chr18	C	18p11.2	4	15375000	30167	nofs	nofs
chr18	C	18p11.1	.	15625000	0	nofs	nofs
chr18	C	18p11.1	.	15875000	0	nofs	nofs
chr18	C		.	16125000	0	nofs	nofs
chr18	C		.	16375000	0	nofs	nofs
chr18	C		.	16625000	0	nofs	nofs
chr18	C	18q11.1	.	16875000	12757	nofs	nofs
chr18	C	18q11.1	.	17125000	2975	nofs	nofs
chr18	C	18q11.1	.	17375000	3760	nofs	nofs
chr18	C	18q11.1	.	17625000	2583	nofs	nofs
chr18	C	18q11.2	2	17875000	3854	nofs	nofs

chr18	C	18q11.2	2	18125000	4531	nofs	nofs
chr18	C	18q11.2	2	18375000	1748	nofs	nofs
chr18	C	18q11.2	2	18625000	2993	nofs	nofs
chr18	C	18q11.2	2	18875000	3618	nofs	nofs
chr18	C	18q11.2	2	19125000	3076	nofs	nofs
chr18	C	18q11.2	2	19375000	3743	nofs	nofs
chr18	C	18q11.2	2	19625000	2224	nofs	nofs
chr18	C	18q11.2	2	19875000	2289	nofs	nofs
chr18	E	18q11.2	2	20125000	2452	nofs	nofs
chr18	E	18q11.2	2	20375000	1902	nofs	nofs
chr18	E	18q11.2	2	20625000	2728	nofs	nofs
chr18	E	18q11.2	2	20875000	3392	nofs	nofs
chr18	E	18q11.2	2	21125000	1885	nofs	nofs
chr18	E	18q11.2	2	21375000	1679	nofs	nofs
chr18	E	18q11.2	2	21625000	1625	nofs	nofs
chr18	E	18q11.2	2	21875000	2275	nofs	nofs
chr18	E	18q11.2	2	22125000	3990	nofs	nofs
chr18	E	18q11.2	2	22375000	2595	nofs	nofs
chr18	E	18q11.2	2	22625000	6014	nofs	nofs
chr18	E	18q11.2	2	22875000	1670	nofs	nofs
chr18	E	18q11.2	2	23125000	2632	nofs	nofs
chr18	B	18q12.1	.	23375000	1423	nofs	nofs
chr18	B	18q12.1	.	23625000	3266	nofs	nofs
chr18	B	18q12.1	.	23875000	2958	nofs	nofs
chr18	B	18q12.1	.	24125000	1827	nofs	nofs
chr18	B	18q12.1	.	24375000	2257	nofs	nofs
chr18	B	18q12.1	.	24625000	3295	nofs	nofs
chr18	B	18q12.1	.	24875000	1638	nofs	nofs
chr18	B	18q12.1	.	25125000	3063	nofs	nofs
chr18	B	18q12.1	.	25375000	4834	nofs	nofs
chr18	B	18q12.1	.	25625000	3463	nofs	nofs
chr18	B	18q12.1	.	25875000	2530	nofs	nofs
chr18	B	18q12.1	.	26125000	2675	nofs	nofs
chr18	B	18q12.1	.	26375000	3938	nofs	nofs
chr18	B	18q12.1	.	26625000	2636	nofs	nofs
chr18	B	18q12.1	.	26875000	3040	nofs	nofs

chr18	B	18q12.1	.	27125000	2947	nofs	nofs
chr18	B	18q12.1	.	27375000	1692	nofs	nofs
chr18	B	18q12.1	.	27625000	2345	nofs	nofs
chr18	B	18q12.1	.	27875000	6428	nofs	nofs
chr18	B	18q12.1	.	28125000	2385	nofs	nofs
chr18	B	18q12.1	.	28375000	1516	nofs	nofs
chr18	B	18q12.1	.	28625000	3270	nofs	nofs
chr18	B	18q12.1	.	28875000	1954	nofs	nofs
chr18	B	18q12.1	.	29125000	2919	nofs	nofs
chr18	B	18q12.1	.	29375000	1477	nofs	nofs
chr18	B	18q12.1	.	29625000	3306	nofs	nofs
chr18	B	18q12.1	.	29875000	1787	nofs	nofs
chr18	B	18q12.1	.	30125000	2417	nofs	nofs
chr18	B	18q12.1	.	30375000	1464	nofs	nofs
chr18	B	18q12.1	.	30625000	1501	nofs	nofs
chr18	E	18q12.2	2	30875000	2639	fs	fs common
chr18	E	18q12.2	2	31125000	4347	fs	fs common
chr18	E	18q12.2	2	31375000	2061	fs	fs common
chr18	E	18q12.2	2	31625000	1454	fs	fs common
chr18	E	18q12.2	2	31875000	3112	fs	fs common
chr18	E	18q12.2	2	32125000	2014	fs	fs common
chr18	E	18q12.2	2	32375000	2072	fs	fs common
chr18	E	18q12.2	2	32625000	2405	fs	fs common
chr18	E	18q12.2	2	32875000	1801	fs	fs common
chr18	E	18q12.2	2	33125000	3410	fs	fs common
chr18	E	18q12.2	2	33375000	2372	fs	fs common
chr18	E	18q12.2	2	33625000	2608	fs	fs common
chr18	E	18q12.2	2	33875000	1694	fs	fs common
chr18	E	18q12.2	2	34125000	1777	fs	fs common
chr18	E	18q12.2	2	34375000	2711	fs	fs common
chr18	E	18q12.2	2	34625000	2062	fs	fs common
chr18	E	18q12.2	2	34875000	2868	fs	fs common
chr18	E	18q12.2	2	35125000	3939	fs	fs common
chr18	E	18q12.2	2	35375000	2907	fs	fs common
chr18	E	18q12.2	2	35625000	4128	fs	fs common
chr18	E	18q12.3	4	35875000	2913	nofs	nofs

chr18	E	18q12.3	4	36125000	2610	nofs	nofs
chr18	E	18q12.3	4	36375000	3448	nofs	nofs
chr18	E	18q12.3	4	36625000	3245	nofs	nofs
chr18	E	18q12.3	4	36875000	2132	nofs	nofs
chr18	E	18q12.3	4	37125000	2635	nofs	nofs
chr18	E	18q12.3	4	37375000	2976	nofs	nofs
chr18	E	18q12.3	4	37625000	1659	nofs	nofs
chr18	E	18q12.3	4	37875000	2207	nofs	nofs
chr18	E	18q12.3	4	38125000	1724	nofs	nofs
chr18	E	18q12.3	4	38375000	2746	nofs	nofs
chr18	E	18q12.3	4	38625000	1931	nofs	nofs
chr18	E	18q12.3	4	38875000	2208	nofs	nofs
chr18	E	18q12.3	4	39125000	2816	nofs	nofs
chr18	E	18q12.3	4	39375000	2725	nofs	nofs
chr18	E	18q12.3	4	39625000	2032	nofs	nofs
chr18	E	18q12.3	4	39875000	2965	nofs	nofs
chr18	E	18q12.3	4	40125000	3612	nofs	nofs
chr18	E	18q12.3	4	40375000	3130	nofs	nofs
chr18	E	18q12.3	4	40625000	2521	nofs	nofs
chr18	E	18q12.3	4	40875000	1624	nofs	nofs
chr18	E	18q12.3	4	41125000	2660	nofs	nofs
chr18	E	18q12.3	4	41375000	1405	nofs	nofs
chr18	E	18q12.3	4	41625000	2338	nofs	nofs
chr18	E	18q12.3	4	41875000	4249	nofs	nofs
chr18	E	18q21.1	4	42125000	4784	nofs	nofs
chr18	E	18q21.1	4	42375000	3149	nofs	nofs
chr18	E	18q21.1	4	42625000	2911	nofs	nofs
chr18	E	18q21.1	4	42875000	2097	nofs	nofs
chr18	E	18q21.1	4	43125000	4829	nofs	nofs
chr18	E	18q21.1	4	43375000	3046	nofs	nofs
chr18	E	18q21.1	4	43625000	3382	nofs	nofs
chr18	E	18q21.1	4	43875000	4591	nofs	nofs
chr18	E	18q21.1	4	44125000	3303	nofs	nofs
chr18	E	18q21.1	4	44375000	5371	nofs	nofs
chr18	E	18q21.1	4	44625000	4947	nofs	nofs
chr18	E	18q21.1	4	44875000	5346	nofs	nofs

chr18	E	18q21.1	4	45125000	4038	nofs	nofs
chr18	E	18q21.1	4	45375000	4507	nofs	nofs
chr18	E	18q21.1	4	45625000	2531	nofs	nofs
chr18	E	18q21.1	4	45875000	1882	nofs	nofs
chr18	E	18q21.1	4	46125000	3133	nofs	nofs
chr18	E	18q21.1	4	46375000	1676	nofs	nofs
chr18	E	18q21.1	4	46625000	2464	nofs	nofs
chr18	E	18q21.1	4	46875000	4288	nofs	nofs
chr18	E	18q21.1	4	47125000	3749	nofs	nofs
chr18	E	18q21.1	4	47375000	2826	nofs	nofs
chr18	E	18q21.1	4	47625000	1311	nofs	nofs
chr18	E	18q21.1	4	47875000	2033	nofs	nofs
chr18	E	18q21.1	4	48125000	2914	nofs	nofs
chr18	E	18q21.1	4	48375000	2796	nofs	nofs
chr18	E	18q21.1	4	48625000	1981	nofs	nofs
chr18	B	18q21.2	.	48875000	3426	nofs	nofs
chr18	B	18q21.2	.	49125000	3887	nofs	nofs
chr18	B	18q21.2	.	49375000	1806	nofs	nofs
chr18	B	18q21.2	.	49625000	2317	nofs	nofs
chr18	B	18q21.2	.	49875000	2304	nofs	nofs
chr18	B	18q21.2	.	50125000	3558	nofs	nofs
chr18	B	18q21.2	.	50375000	1605	nofs	nofs
chr18	B	18q21.2	.	50625000	3141	nofs	nofs
chr18	B	18q21.2	.	50875000	2297	nofs	nofs
chr18	B	18q21.2	.	51125000	1901	nofs	nofs
chr18	B	18q21.2	.	51375000	2969	nofs	nofs
chr18	B	18q21.2	.	51625000	1894	nofs	nofs
chr18	B	18q21.2	.	51875000	1315	nofs	nofs
chr18	B	18q21.2	.	52125000	3998	nofs	nofs
chr18	B	18q21.2	.	52375000	2716	nofs	nofs
chr18	E	18q21.3	4	52625000	1698	fs	fs common
chr18	E	18q21.3	4	52875000	2408	fs	fs common
chr18	E	18q21.3	4	53125000	4120	fs	fs common
chr18	E	18q21.3	4	53375000	6220	fs	fs common
chr18	E	18q21.3	4	53625000	3933	fs	fs common
chr18	E	18q21.3	4	53875000	5400	fs	fs common

chr18	E	18q21.3	4	54125000	4214	fs	fs common
chr18	E	18q21.3	4	54375000	5020	fs	fs common
chr18	E	18q21.3	4	54625000	1894	fs	fs common
chr18	E	18q21.3	4	54875000	3724	fs	fs common
chr18	E	18q21.3	4	55125000	4476	fs	fs common
chr18	E	18q21.3	4	55375000	1821	fs	fs common
chr18	E	18q21.3	4	55625000	5397	fs	fs common
chr18	E	18q21.3	4	55875000	2461	fs	fs common
chr18	E	18q21.3	4	56125000	1351	fs	fs common
chr18	E	18q21.3	4	56375000	2254	fs	fs common
chr18	E	18q21.3	4	56625000	2263	fs	fs common
chr18	E	18q21.3	4	56875000	4128	fs	fs common
chr18	E	18q21.3	4	57125000	1797	fs	fs common
chr18	E	18q21.3	4	57375000	1988	fs	fs common
chr18	E	18q21.3	4	57625000	2240	fs	fs common
chr18	E	18q21.3	4	57875000	5463	fs	fs common
chr18	E	18q21.3	4	58125000	1609	fs	fs common
chr18	E	18q21.3	4	58375000	3097	fs	fs common
chr18	E	18q21.3	4	58625000	3416	fs	fs common
chr18	E	18q21.3	4	58875000	4225	fs	fs common
chr18	E	18q21.3	4	59125000	3099	fs	fs common
chr18	E	18q21.3	4	59375000	3887	fs	fs common
chr18	E	18q21.3	4	59625000	3088	fs	fs common
chr18	E	18q21.3	4	59875000	4675	fs	fs common
chr18	E	18q22	4	60125000	1946	fs	fs rare
chr18	E	18q22	4	60375000	3477	fs	fs rare
chr18	E	18q22	4	60625000	2191	fs	fs rare
chr18	E	18q22	4	60875000	2816	fs	fs rare
chr18	E	18q22	4	61125000	2702	fs	fs rare
chr18	E	18q22	4	61375000	3549	fs	fs rare
chr18	E	18q22	4	61625000	4110	fs	fs rare
chr18	E	18q22	4	61875000	3464	fs	fs rare
chr18	E	18q22	4	62125000	2275	fs	fs rare
chr18	E	18q22	4	62375000	2825	fs	fs rare
chr18	E	18q22	4	62625000	2822	fs	fs rare
chr18	E	18q22	4	62875000	3780	fs	fs rare

chr18	E	18q22	4	63125000	4425	fs	fs rare
chr18	E	18q22	4	63375000	2615	fs	fs rare
chr18	E	18q22	4	63625000	2911	fs	fs rare
chr18	E	18q22	4	63875000	2533	fs	fs rare
chr18	E	18q22	4	64125000	3098	fs	fs rare
chr18	E	18q22	4	64375000	2984	fs	fs rare
chr18	E	18q22	4	64625000	3584	fs	fs rare
chr18	E	18q22	4	64875000	3339	fs	fs rare
chr18	E	18q22	4	65125000	2085	nofs	nofs
chr18	E	18q22	4	65375000	3558	nofs	nofs
chr18	E	18q22	4	65625000	3430	nofs	nofs
chr18	E	18q22	4	65875000	3774	nofs	nofs
chr18	E	18q22	4	66125000	3308	nofs	nofs
chr18	E	18q22	4	66375000	3397	nofs	nofs
chr18	E	18q22	4	66625000	1766	nofs	nofs
chr18	E	18q22	4	66875000	3043	nofs	nofs
chr18	E	18q22	4	67125000	3359	nofs	nofs
chr18	E	18q22	4	67375000	3780	nofs	nofs
chr18	E	18q22	4	67625000	1975	nofs	nofs
chr18	E	18q22	4	67875000	1883	nofs	nofs
chr18	E	18q22	4	68125000	3240	nofs	nofs
chr18	E	18q22	4	68375000	2909	nofs	nofs
chr18	E	18q22	4	68625000	2420	nofs	nofs
chr18	E	18q22	4	68875000	2304	nofs	nofs
chr18	E	18q22	4	69125000	3661	nofs	nofs
chr18	E	18q22	4	69375000	3106	nofs	nofs
chr18	E	18q22	4	69625000	2577	nofs	nofs
chr18	E	18q22	4	69875000	3569	nofs	nofs
chr18	E	18q22	4	70125000	5236	nofs	nofs
chr18	E	18q22	4	70375000	4213	nofs	nofs
chr18	E	18q22	4	70625000	2497	nofs	nofs
chr18	E	18q22	4	70875000	8618	nofs	nofs
chr18	E	18q22	4	71125000	4369	nofs	nofs
chr18	E	18q22	4	71375000	2587	nofs	nofs
chr18	E	18q23	1	71625000	4387	nofs	nofs
chr18	E	18q23	1	71875000	3033	nofs	nofs

chr18	E	18q23	1	72125000	8140	nofs	nofs
chr18	E	18q23	1	72375000	10148	nofs	nofs
chr18	T	18q23	1	72625000	10398	nofs	nofs
chr18	T	18q23	1	72875000	9922	nofs	nofs
chr18	T	18q23	1	73125000	5682	nofs	nofs
chr18	T	18q23	1	73375000	6506	nofs	nofs
chr18	T	18q23	1	73625000	9145	nofs	nofs
chr18	T	18q23	1	73875000	9596	nofs	nofs
chr18	T	18q23	1	74125000	9569	nofs	nofs
chr18	T	18q23	1	74375000	26053	nofs	nofs
chr18	T	18q23	1	74625000	29856	nofs	nofs
chr18	T	18q23	1	74875000	20960	nofs	nofs
chr18	T	18q23	1	75125000	20374	nofs	nofs
chr18	T	18q23	1	75375000	34164	nofs	nofs
chr18	T	18q23	1	75625000	32364	nofs	nofs
chr19	T	19p13.3	4	125000	10359	fs	fs rare
chr19	T	19p13.3	4	375000	53056	fs	fs rare
chr19	T	19p13.3	4	625000	19058	fs	fs rare
chr19	T	19p13.3	4	875000	18961	fs	fs rare
chr19	T	19p13.3	4	1125000	16430	fs	fs rare
chr19	T	19p13.3	4	1375000	11604	fs	fs rare
chr19	T	19p13.3	4	1625000	13328	fs	fs rare
chr19	T	19p13.3	4	1875000	11631	fs	fs rare
chr19	T	19p13.3	4	2125000	9985	fs	fs rare
chr19	T	19p13.3	4	2375000	18872	fs	fs rare
chr19	T	19p13.3	4	2625000	15803	fs	fs rare
chr19	T	19p13.3	4	2875000	14025	fs	fs rare
chr19	T	19p13.3	4	3125000	17568	fs	fs rare
chr19	E	19p13.3	4	3375000	16009	fs	fs rare
chr19	E	19p13.3	4	3625000	11995	fs	fs rare
chr19	E	19p13.3	4	3875000	9045	fs	fs rare
chr19	E	19p13.3	4	4125000	11278	fs	fs rare
chr19	E	19p13.3	4	4375000	14170	fs	fs rare
chr19	E	19p13.3	4	4625000	9935	fs	fs rare
chr19	E	19p13.3	4	4875000	11525	fs	fs rare
chr19	E	19p13.3	4	5125000	9521	fs	fs rare

chr19	E	19p13.3	4	5375000	8927	fs	fs rare
chr19	E	19p13.3	4	5625000	9006	fs	fs rare
chr19	E	19p13.3	4	5875000	6896	fs	fs rare
chr19	E	19p13.3	4	6125000	7878	fs	fs rare
chr19	E	19p13.3	4	6375000	8140	fs	fs rare
chr19	E	19p13.3	4	6625000	13414	fs	fs rare
chr19	E	19p13.3	4	6875000	9585	fs	fs rare
chr19	E	19p13.2	4	7125000	13454	fs	fs rare
chr19	E	19p13.2	4	7375000	12583	fs	fs rare
chr19	E	19p13.2	4	7625000	11427	fs	fs rare
chr19	E	19p13.2	4	7875000	8312	fs	fs rare
chr19	E	19p13.2	4	8125000	11694	fs	fs rare
chr19	E	19p13.2	4	8375000	9710	fs	fs rare
chr19	E	19p13.2	4	8625000	12866	fs	fs rare
chr19	E	19p13.2	4	8875000	7076	fs	fs rare
chr19	E	19p13.2	4	9125000	8802	fs	fs rare
chr19	E	19p13.2	4	9375000	5950	fs	fs rare
chr19	E	19p13.2	4	9625000	8247	fs	fs rare
chr19	E	19p13.2	4	9875000	11870	fs	fs rare
chr19	E	19p13.2	4	10125000	7594	fs	fs rare
chr19	E	19p13.2	4	10375000	9712	fs	fs rare
chr19	E	19p13.2	4	10625000	8002	fs	fs rare
chr19	E	19p13.2	4	10875000	5564	fs	fs rare
chr19	E	19p13.2	4	11125000	8463	fs	fs rare
chr19	E	19p13.2	4	11375000	7401	fs	fs rare
chr19	E	19p13.2	4	11625000	8192	fs	fs rare
chr19	E	19p13.2	4	11875000	11842	fs	fs rare
chr19	E	19p13.2	4	12125000	12704	fs	fs rare
chr19	E	19p13.2	4	12375000	11269	fs	fs rare
chr19	E	19p13.2	4	12625000	5448	fs	fs rare
chr19	E	19p13.1	5	12875000	5654	fs	fs rare
chr19	E	19p13.1	5	13125000	5990	fs	fs rare
chr19	E	19p13.1	5	13375000	6940	fs	fs rare
chr19	E	19p13.1	5	13625000	9799	fs	fs rare
chr19	E	19p13.1	5	13875000	9158	fs	fs rare
chr19	E	19p13.1	5	14125000	8462	fs	fs rare

chr19	E	19p13.1	5	14375000	7238	fs	fs rare
chr19	E	19p13.1	5	14625000	10168	fs	fs rare
chr19	E	19p13.1	5	14875000	3194	fs	fs rare
chr19	E	19p13.1	5	15125000	6714	fs	fs rare
chr19	E	19p13.1	5	15375000	5661	fs	fs rare
chr19	E	19p13.1	5	15625000	9097	fs	fs rare
chr19	E	19p13.1	5	15875000	12970	fs	fs rare
chr19	E	19p13.1	5	16125000	8526	fs	fs rare
chr19	E	19p13.1	5	16375000	6427	fs	fs rare
chr19	E	19p13.1	5	16625000	7211	fs	fs rare
chr19	E	19p13.1	5	16875000	8802	fs	fs rare
chr19	E	19p13.1	5	17125000	5543	fs	fs rare
chr19	E	19p13.1	5	17375000	10722	fs	fs rare
chr19	E	19p13.1	5	17625000	8175	fs	fs rare
chr19	E	19p13.1	5	17875000	8771	fs	fs rare
chr19	E	19p13.1	5	18125000	7694	fs	fs rare
chr19	E	19p13.1	5	18375000	5573	fs	fs rare
chr19	E	19p13.1	5	18625000	7165	fs	fs rare
chr19	E	19p13.1	5	18875000	7142	fs	fs rare
chr19	E	19p13.1	5	19125000	6094	fs	fs rare
chr19	E	19p13.1	5	19375000	5174	fs	fs rare
chr19	E	19p13.1	5	19625000	9069	fs	fs rare
chr19	E	19p13.1	5	19875000	6945	fs	fs rare
chr19	E	19p12	2	20125000	8525	nofs	nofs
chr19	E	19p12	2	20375000	6044	nofs	nofs
chr19	E	19p12	2	20625000	8518	nofs	nofs
chr19	E	19p12	2	20875000	56807	nofs	nofs
chr19	E	19p12	2	21125000	9543	nofs	nofs
chr19	C	19p12	2	21375000	13536	nofs	nofs
chr19	C	19p12	2	21625000	8275	nofs	nofs
chr19	C	19p12	2	21875000	10835	nofs	nofs
chr19	C	19p12	2	22125000	8215	nofs	nofs
chr19	C	19p12	2	22375000	10282	nofs	nofs
chr19	C	19p12	2	22625000	9922	nofs	nofs
chr19	C	19p12	2	22875000	7190	nofs	nofs
chr19	C	19p12	2	23125000	8776	nofs	nofs

chr19	C	19p12	2	23375000	12099	nofs	nofs
chr19	C	19p12	2	23625000	7863	nofs	nofs
chr19	C	19p12	2	23875000	9896	nofs	nofs
chr19	C	19p12	2	24125000	50175	nofs	nofs
chr19	C	19p12	2	24375000	144443	nofs	nofs
chr19	C	19p11	.	24625000	0	nofs	nofs
chr19	C	19p11	.	24875000	0	nofs	nofs
chr19	C	19p11	.	25125000	0	nofs	nofs
chr19	C	19p11	.	25375000	0	nofs	nofs
chr19	C	19p11	.	25625000	0	nofs	nofs
chr19	C	19p11	.	25875000	0	nofs	nofs
chr19	C	19p11	.	26125000	0	nofs	nofs
chr19	C	19p11	.	26375000	0	nofs	nofs
chr19	C	19p11	.	26625000	0	nofs	nofs
chr19	C	19p11	.	26875000	0	nofs	nofs
chr19	C	19p11	.	27125000	0	nofs	nofs
chr19	C	19p11	.	27375000	0	nofs	nofs
chr19	C	19p11	.	27625000	0	nofs	nofs
chr19	C	19p11	.	27875000	0	nofs	nofs
chr19	C	19p11	.	28125000	0	nofs	nofs
chr19	C	19p11	.	28375000	0	nofs	nofs
chr19	C		.	28625000	0	nofs	nofs
chr19	C		.	28875000	0	nofs	nofs
chr19	C		.	29125000	0	nofs	nofs
chr19	C		.	29375000	0	nofs	nofs
chr19	C		.	29625000	0	nofs	nofs
chr19	C		.	29875000	0	nofs	nofs
chr19	C		.	30125000	0	nofs	nofs
chr19	C	19q11	.	30375000	0	nofs	nofs
chr19	C	19q11	.	30625000	0	nofs	nofs
chr19	C	19q11	.	30875000	0	nofs	nofs
chr19	C	19q11	.	31125000	0	nofs	nofs
chr19	C	19q11	.	31375000	0	nofs	nofs
chr19	C	19q12	0	31625000	0	nofs	nofs
chr19	C	19q12	1	31875000	0	nofs	nofs
chr19	C	19q12	2	32125000	0	nofs	nofs

chr19	C	19q12	3	32375000	62067	nofs	nofs
chr19	C	19q12	3	32625000	212462	nofs	nofs
chr19	C	19q12	3	32875000	101346	nofs	nofs
chr19	C	19q12	3	33125000	6981	nofs	nofs
chr19	C	19q12	3	33375000	4168	nofs	nofs
chr19	C	19q12	3	33625000	5218	nofs	nofs
chr19	C	19q12	3	33875000	4422	nofs	nofs
chr19	C	19q12	3	34125000	5607	nofs	nofs
chr19	C	19q12	3	34375000	6912	nofs	nofs
chr19	C	19q12	3	34625000	11670	nofs	nofs
chr19	C	19q12	3	34875000	7436	nofs	nofs
chr19	C	19q12	3	35125000	9081	nofs	nofs
chr19	C	19q12	3	35375000	7290	nofs	nofs
chr19	E	19q12	3	35625000	7886	nofs	nofs
chr19	E	19q12	3	35875000	4548	nofs	nofs
chr19	E	19q12	3	36125000	3425	nofs	nofs
chr19	E	19q12	3	36375000	4422	nofs	nofs
chr19	E	19q12	3	36625000	4613	nofs	nofs
chr19	E	19q12	3	36875000	6220	nofs	nofs
chr19	E	19q13.1	1	37125000	4734	fs	fs common
chr19	E	19q13.1	1	37375000	5991	fs	fs common
chr19	E	19q13.1	1	37625000	3763	fs	fs common
chr19	E	19q13.1	1	37875000	7873	fs	fs common
chr19	E	19q13.1	1	38125000	5678	fs	fs common
chr19	E	19q13.1	1	38375000	6028	fs	fs common
chr19	E	19q13.1	1	38625000	4867	fs	fs common
chr19	E	19q13.1	1	38875000	5057	fs	fs common
chr19	E	19q13.1	1	39125000	5815	fs	fs common
chr19	E	19q13.1	1	39375000	6875	fs	fs common
chr19	E	19q13.1	1	39625000	5837	fs	fs common
chr19	E	19q13.1	1	39875000	3642	fs	fs common
chr19	E	19q13.1	1	40125000	4712	fs	fs common
chr19	E	19q13.1	1	40375000	6212	fs	fs common
chr19	E	19q13.1	1	40625000	5069	fs	fs common
chr19	E	19q13.1	1	40875000	4916	fs	fs common
chr19	E	19q13.1	1	41125000	7913	fs	fs common

chr19	E	19q13.1	1	41375000	6278	fs	fs common
chr19	E	19q13.1	1	41625000	6899	fs	fs common
chr19	E	19q13.1	1	41875000	9747	fs	fs common
chr19	E	19q13.1	1	42125000	7666	fs	fs common
chr19	E	19q13.1	1	42375000	6826	fs	fs common
chr19	E	19q13.1	1	42625000	7104	fs	fs common
chr19	E	19q13.1	1	42875000	9980	fs	fs common
chr19	E	19q13.1	1	43125000	3304	fs	fs common
chr19	E	19q13.1	1	43375000	6500	fs	fs common
chr19	E	19q13.2	1	43625000	5996	fs	fs common
chr19	E	19q13.2	1	43875000	7678	fs	fs common
chr19	E	19q13.2	1	44125000	7876	fs	fs common
chr19	E	19q13.2	1	44375000	7042	fs	fs common
chr19	E	19q13.2	1	44625000	4869	fs	fs common
chr19	E	19q13.2	1	44875000	1407	fs	fs common
chr19	E	19q13.2	1	45125000	7281	fs	fs common
chr19	E	19q13.2	1	45375000	7883	fs	fs common
chr19	E	19q13.2	1	45625000	7270	fs	fs common
chr19	E	19q13.2	1	45875000	5652	fs	fs common
chr19	E	19q13.2	1	46125000	5960	fs	fs common
chr19	E	19q13.2	1	46375000	7406	fs	fs common
chr19	E	19q13.2	1	46625000	30550	fs	fs common
chr19	E	19q13.2	1	46875000	2992	fs	fs common
chr19	E	19q13.2	1	47125000	11476	fs	fs common
chr19	E	19q13.2	1	47375000	4085	fs	fs common
chr19	E	19q13.2	1	47625000	3880	fs	fs common
chr19	E	19q13.2	1	47875000	3465	fs	fs common
chr19	E	19q13.2	1	48125000	1319	fs	fs common
chr19	E	19q13.3	3	48375000	1783	fs	fs common
chr19	E	19q13.3	3	48625000	8796	fs	fs common
chr19	E	19q13.3	3	48875000	8163	fs	fs common
chr19	E	19q13.3	3	49125000	5627	fs	fs common
chr19	E	19q13.3	3	49375000	8183	fs	fs common
chr19	E	19q13.3	3	49625000	8844	fs	fs common
chr19	E	19q13.3	3	49875000	14109	fs	fs common
chr19	E	19q13.3	3	50125000	7847	fs	fs common

chr19	E	19q13.3	3	50375000	7223	fs	fs common
chr19	E	19q13.3	3	50625000	5248	fs	fs common
chr19	E	19q13.3	3	50875000	7047	fs	fs common
chr19	E	19q13.3	3	51125000	4545	fs	fs common
chr19	E	19q13.3	3	51375000	4940	fs	fs common
chr19	E	19q13.3	3	51625000	6637	fs	fs common
chr19	E	19q13.3	3	51875000	6482	fs	fs common
chr19	E	19q13.3	3	52125000	6048	fs	fs common
chr19	E	19q13.3	3	52375000	9447	fs	fs common
chr19	E	19q13.3	3	52625000	6366	fs	fs common
chr19	E	19q13.3	3	52875000	6320	fs	fs common
chr19	E	19q13.3	3	53125000	9162	fs	fs common
chr19	E	19q13.3	3	53375000	5028	fs	fs common
chr19	E	19q13.3	3	53625000	10399	fs	fs common
chr19	E	19q13.3	3	53875000	10479	fs	fs common
chr19	E	19q13.3	3	54125000	10879	fs	fs common
chr19	E	19q13.3	3	54375000	10694	fs	fs common
chr19	E	19q13.3	3	54625000	15924	fs	fs common
chr19	E	19q13.3	3	54875000	9053	fs	fs common
chr19	E	19q13.3	3	55125000	3746	fs	fs common
chr19	E	19q13.3	3	55375000	8532	fs	fs common
chr19	E	19q13.3	3	55625000	11353	fs	fs common
chr19	E	19q13.3	3	55875000	7402	fs	fs common
chr19	E	19q13.4	3	56125000	13617	fs	fs common
chr19	E	19q13.4	3	56375000	3808	fs	fs common
chr19	E	19q13.4	3	56625000	7342	fs	fs common
chr19	E	19q13.4	3	56875000	4939	fs	fs common
chr19	E	19q13.4	3	57125000	6887	fs	fs common
chr19	E	19q13.4	3	57375000	7362	fs	fs common
chr19	E	19q13.4	3	57625000	10547	fs	fs common
chr19	E	19q13.4	3	57875000	11724	fs	fs common
chr19	E	19q13.4	3	58125000	10381	fs	fs common
chr19	E	19q13.4	3	58375000	11992	fs	fs common
chr19	E	19q13.4	3	58625000	10529	fs	fs common
chr19	E	19q13.4	3	58875000	9319	fs	fs common
chr19	E	19q13.4	3	59125000	15672	fs	fs common

chr19	E	19q13.4	3	59375000	10073	fs	fs common
chr19	E	19q13.4	3	59625000	12260	fs	fs common
chr19	E	19q13.4	3	59875000	11137	fs	fs common
chr19	E	19q13.4	3	60125000	9991	fs	fs common
chr19	E	19q13.4	3	60375000	17497	fs	fs common
chr19	T	19q13.4	3	60625000	17858	fs	fs common
chr19	T	19q13.4	3	60875000	5340	fs	fs common
chr19	T	19q13.4	3	61125000	9264	fs	fs common
chr19	T	19q13.4	3	61375000	14200	fs	fs common
chr19	T	19q13.4	3	61625000	7513	fs	fs common
chr19	T	19q13.4	3	61875000	11343	fs	fs common
chr19	T	19q13.4	3	62125000	7804	fs	fs common
chr19	T	19q13.4	3	62375000	7956	fs	fs common
chr19	T	19q13.4	3	62625000	12708	fs	fs common
chr19	T	19q13.4	3	62875000	9330	fs	fs common
chr19	T	19q13.4	3	63125000	9818	fs	fs common
chr19	T	19q13.4	3	63375000	7345	fs	fs common
chr19	T	19q13.4	3	63625000	12761	fs	fs common
chr20	T	20p13	5	125000	4034	nofs	nofs
chr20	T	20p13	5	375000	2971	nofs	nofs
chr20	T	20p13	5	625000	4187	nofs	nofs
chr20	T	20p13	5	875000	3028	nofs	nofs
chr20	T	20p13	5	1125000	7559	nofs	nofs
chr20	T	20p13	5	1375000	1700	nofs	nofs
chr20	T	20p13	5	1625000	2609	nofs	nofs
chr20	T	20p13	5	1875000	3690	nofs	nofs
chr20	T	20p13	5	2125000	2567	nofs	nofs
chr20	T	20p13	5	2375000	3317	nofs	nofs
chr20	T	20p13	5	2625000	2601	nofs	nofs
chr20	T	20p13	5	2875000	6985	nofs	nofs
chr20	T	20p13	5	3125000	6164	nofs	nofs
chr20	E	20p13	5	3375000	4667	nofs	nofs
chr20	E	20p13	5	3625000	3493	nofs	nofs
chr20	E	20p13	5	3875000	4952	nofs	nofs
chr20	E	20p13	5	4125000	4292	nofs	nofs
chr20	E	20p13	5	4375000	2464	nofs	nofs

chr20	E	20p13	5	4625000	2463	nofs	nofs
chr20	E	20p13	5	4875000	4668	nofs	nofs
chr20	E	20p12	2	5125000	2132	nofs	nofs
chr20	E	20p12	2	5375000	2839	nofs	nofs
chr20	E	20p12	2	5625000	4041	nofs	nofs
chr20	E	20p12	2	5875000	2635	nofs	nofs
chr20	E	20p12	2	6125000	3539	nofs	nofs
chr20	E	20p12	2	6375000	2719	nofs	nofs
chr20	E	20p12	2	6625000	2953	nofs	nofs
chr20	E	20p12	2	6875000	1460	nofs	nofs
chr20	E	20p12	2	7125000	2535	nofs	nofs
chr20	E	20p12	2	7375000	2742	nofs	nofs
chr20	E	20p12	2	7625000	2667	nofs	nofs
chr20	E	20p12	2	7875000	2473	nofs	nofs
chr20	E	20p12	2	8125000	2002	nofs	nofs
chr20	E	20p12	2	8375000	2791	nofs	nofs
chr20	E	20p12	2	8625000	3783	nofs	nofs
chr20	E	20p12	2	8875000	2391	fs	fs common
chr20	E	20p12	2	9125000	3334	fs	fs common
chr20	E	20p12	2	9375000	2443	fs	fs common
chr20	E	20p12	2	9625000	2238	fs	fs common
chr20	E	20p12	2	9875000	3347	fs	fs common
chr20	E	20p12	2	10125000	2497	fs	fs common
chr20	E	20p12	2	10375000	2975	fs	fs common
chr20	E	20p12	2	10625000	2260	fs	fs common
chr20	E	20p12	2	10875000	3006	fs	fs common
chr20	E	20p12	2	11125000	2339	fs	fs common
chr20	E	20p12	2	11375000	2849	fs	fs common
chr20	E	20p12	2	11625000	1735	fs	fs common
chr20	E	20p12	2	11875000	2093	fs	fs common
chr20	E	20p12	2	12125000	1508	nofs	nofs
chr20	E	20p12	2	12375000	1720	nofs	nofs
chr20	E	20p12	2	12625000	2327	nofs	nofs
chr20	E	20p12	2	12875000	2618	nofs	nofs
chr20	E	20p12	2	13125000	2655	nofs	nofs
chr20	E	20p12	2	13375000	1889	nofs	nofs

chr20	E	20p12	2	13625000	4570	nofs	nofs
chr20	E	20p12	2	13875000	2764	nofs	nofs
chr20	E	20p12	2	14125000	2247	nofs	nofs
chr20	E	20p12	2	14375000	1390	nofs	nofs
chr20	E	20p12	2	14625000	2295	nofs	nofs
chr20	E	20p12	2	14875000	4165	nofs	nofs
chr20	E	20p12	2	15125000	2792	nofs	nofs
chr20	E	20p12	2	15375000	1858	nofs	nofs
chr20	E	20p12	2	15625000	2765	nofs	nofs
chr20	E	20p12	2	15875000	2029	nofs	nofs
chr20	E	20p12	2	16125000	7276	nofs	nofs
chr20	E	20p12	2	16375000	2018	nofs	nofs
chr20	E	20p12	2	16625000	1620	nofs	nofs
chr20	E	20p12	2	16875000	2366	nofs	nofs
chr20	E	20p12	2	17125000	2914	nofs	nofs
chr20	E	20p12	2	17375000	4184	nofs	nofs
chr20	E	20p12	2	17625000	3630	nofs	nofs
chr20	E	20p11.2	2	17875000	5208	fs	fs rare
chr20	E	20p11.2	2	18125000	5612	fs	fs rare
chr20	E	20p11.2	2	18375000	2331	fs	fs rare
chr20	E	20p11.2	2	18625000	2990	fs	fs rare
chr20	E	20p11.2	2	18875000	1863	fs	fs rare
chr20	E	20p11.2	2	19125000	2608	fs	fs rare
chr20	E	20p11.2	2	19375000	2614	fs	fs rare
chr20	E	20p11.2	2	19625000	3786	fs	fs rare
chr20	E	20p11.2	2	19875000	3838	fs	fs rare
chr20	E	20p11.2	2	20125000	2640	fs	fs rare
chr20	E	20p11.2	2	20375000	5357	fs	fs rare
chr20	E	20p11.2	2	20625000	1879	fs	fs rare
chr20	E	20p11.2	2	20875000	2848	fs	fs rare
chr20	E	20p11.2	2	21125000	2947	fs	fs rare
chr20	E	20p11.2	2	21375000	2655	fs	fs rare
chr20	E	20p11.2	2	21625000	3223	nofs	nofs
chr20	E	20p11.2	2	21875000	2612	nofs	nofs
chr20	E	20p11.2	2	22125000	3582	nofs	nofs
chr20	E	20p11.2	2	22375000	3035	nofs	nofs

chr20	E	20p11.2	2	22625000	2937	nofs	nofs
chr20	E	20p11.2	2	22875000	1959	nofs	nofs
chr20	E	20p11.2	2	23125000	4760	nofs	nofs
chr20	E	20p11.2	2	23375000	4019	nofs	nofs
chr20	C	20p11.2	2	23625000	4118	nofs	nofs
chr20	C	20p11.2	2	23875000	3387	nofs	nofs
chr20	C	20p11.2	2	24125000	3429	nofs	nofs
chr20	C	20p11.2	2	24375000	6939	nofs	nofs
chr20	C	20p11.2	2	24625000	8444	nofs	nofs
chr20	C	20p11.2	2	24875000	5760	nofs	nofs
chr20	C	20p11.2	2	25125000	4323	nofs	nofs
chr20	C	20p11.2	2	25375000	6617	nofs	nofs
chr20	C	20p11.2	2	25625000	4935	nofs	nofs
chr20	C	20p11.2	2	25875000	10987	nofs	nofs
chr20	C	20p11.2	2	26125000	45676	nofs	nofs
chr20	C	20p11.2	2	26375000	17569	nofs	nofs
chr20	C	20p11.1	.	26625000	0	nofs	nofs
chr20	C	20p11.1	.	26875000	0	nofs	nofs
chr20	C	20p11.1	.	27125000	0	nofs	nofs
chr20	C		.	27375000	0	nofs	nofs
chr20	C		.	27625000	0	nofs	nofs
chr20	C		.	27875000	0	nofs	nofs
chr20	C	20q11.1	.	28125000	7134	nofs	nofs
chr20	C	20q11.1	.	28375000	2309	nofs	nofs
chr20	C	20q11.1	.	28625000	0	nofs	nofs
chr20	C	20q11.1	.	28875000	0	nofs	nofs
chr20	C	20q11.1	.	29125000	0	nofs	nofs
chr20	C	20q11.2	2	29375000	32354	nofs	nofs
chr20	C	20q11.2	2	29625000	8225	nofs	nofs
chr20	C	20q11.2	2	29875000	4931	nofs	nofs
chr20	C	20q11.2	2	30125000	8523	nofs	nofs
chr20	C	20q11.2	2	30375000	3848	nofs	nofs
chr20	C	20q11.2	2	30625000	6862	nofs	nofs
chr20	C	20q11.2	2	30875000	5740	nofs	nofs
chr20	C	20q11.2	2	31125000	6437	nofs	nofs
chr20	E	20q11.2	2	31375000	4926	nofs	nofs

chr20	E	20q11.2	2	31625000	4768	nofs	nofs
chr20	E	20q11.2	2	31875000	6020	nofs	nofs
chr20	E	20q11.2	2	32125000	3872	nofs	nofs
chr20	E	20q11.2	2	32375000	7258	nofs	nofs
chr20	E	20q11.2	2	32625000	5278	nofs	nofs
chr20	E	20q11.2	2	32875000	2224	nofs	nofs
chr20	E	20q11.2	2	33125000	4777	nofs	nofs
chr20	E	20q11.2	2	33375000	3902	nofs	nofs
chr20	E	20q11.2	2	33625000	5270	nofs	nofs
chr20	E	20q11.2	2	33875000	3693	nofs	nofs
chr20	E	20q11.2	2	34125000	4347	nofs	nofs
chr20	E	20q11.2	2	34375000	3172	nofs	nofs
chr20	E	20q11.2	2	34625000	4771	nofs	nofs
chr20	E	20q11.2	2	34875000	3837	nofs	nofs
chr20	E	20q11.2	2	35125000	9521	nofs	nofs
chr20	E	20q11.2	2	35375000	5030	nofs	nofs
chr20	E	20q11.2	2	35625000	3104	nofs	nofs
chr20	E	20q11.2	2	35875000	2700	nofs	nofs
chr20	E	20q11.2	2	36125000	3050	nofs	nofs
chr20	E	20q11.2	2	36375000	6060	nofs	nofs
chr20	E	20q11.2	2	36625000	7362	nofs	nofs
chr20	E	20q11.2	2	36875000	4017	nofs	nofs
chr20	E	20q11.2	2	37125000	2748	nofs	nofs
chr20	B	20q12	.	37375000	1575	nofs	nofs
chr20	B	20q12	.	37625000	3313	nofs	nofs
chr20	B	20q12	.	37875000	1915	nofs	nofs
chr20	B	20q12	.	38125000	2690	nofs	nofs
chr20	B	20q12	.	38375000	2977	nofs	nofs
chr20	B	20q12	.	38625000	2729	nofs	nofs
chr20	B	20q12	.	38875000	1604	nofs	nofs
chr20	B	20q12	.	39125000	2320	nofs	nofs
chr20	B	20q12	.	39375000	2196	nofs	nofs
chr20	B	20q12	.	39625000	2924	nofs	nofs
chr20	B	20q12	.	39875000	2845	nofs	nofs
chr20	B	20q12	.	40125000	1590	nofs	nofs
chr20	B	20q12	.	40375000	2431	nofs	nofs

chr20	B	20q12	.	40625000	2746	nofs	nofs
chr20	B	20q12	.	40875000	1829	nofs	nofs
chr20	E	20q13.1	1	41125000	4068	nofs	nofs
chr20	E	20q13.1	1	41375000	3906	nofs	nofs
chr20	E	20q13.1	1	41625000	3012	nofs	nofs
chr20	E	20q13.1	1	41875000	1990	nofs	nofs
chr20	E	20q13.1	1	42125000	4236	nofs	nofs
chr20	E	20q13.1	1	42375000	3701	nofs	nofs
chr20	E	20q13.1	1	42625000	3360	nofs	nofs
chr20	E	20q13.1	1	42875000	4009	nofs	nofs
chr20	E	20q13.1	1	43125000	2248	nofs	nofs
chr20	E	20q13.1	1	43375000	5217	nofs	nofs
chr20	E	20q13.1	1	43625000	3292	nofs	nofs
chr20	E	20q13.1	1	43875000	3862	nofs	nofs
chr20	E	20q13.1	1	44125000	3316	nofs	nofs
chr20	E	20q13.1	1	44375000	2829	nofs	nofs
chr20	E	20q13.1	1	44625000	4191	nofs	nofs
chr20	E	20q13.1	1	44875000	3517	nofs	nofs
chr20	E	20q13.1	1	45125000	3084	nofs	nofs
chr20	E	20q13.1	1	45375000	6584	nofs	nofs
chr20	E	20q13.1	1	45625000	4644	nofs	nofs
chr20	E	20q13.1	1	45875000	6090	nofs	nofs
chr20	E	20q13.1	1	46125000	7352	nofs	nofs
chr20	E	20q13.1	1	46375000	4283	nofs	nofs
chr20	E	20q13.1	1	46625000	10778	nofs	nofs
chr20	E	20q13.1	1	46875000	4558	nofs	nofs
chr20	E	20q13.1	1	47125000	3212	nofs	nofs
chr20	E	20q13.1	1	47375000	2645	nofs	nofs
chr20	E	20q13.1	1	47625000	5049	nofs	nofs
chr20	E	20q13.1	1	47875000	3296	nofs	nofs
chr20	E	20q13.1	1	48125000	2993	nofs	nofs
chr20	E	20q13.1	1	48375000	3067	nofs	nofs
chr20	E	20q13.1	1	48625000	3973	nofs	nofs
chr20	E	20q13.1	1	48875000	3043	nofs	nofs
chr20	E	20q13.1	1	49125000	4836	nofs	nofs
chr20	E	20q13.1	1	49375000	4534	nofs	nofs

chr20	B	20q13.2	.	49625000	3974	nofs	nofs
chr20	B	20q13.2	.	49875000	4883	nofs	nofs
chr20	B	20q13.2	.	50125000	4613	nofs	nofs
chr20	B	20q13.2	.	50375000	4541	nofs	nofs
chr20	B	20q13.2	.	50625000	4149	nofs	nofs
chr20	B	20q13.2	.	50875000	3918	nofs	nofs
chr20	B	20q13.2	.	51125000	4052	nofs	nofs
chr20	B	20q13.2	.	51375000	3248	nofs	nofs
chr20	B	20q13.2	.	51625000	5284	nofs	nofs
chr20	B	20q13.2	.	51875000	6010	nofs	nofs
chr20	B	20q13.2	.	52125000	3399	nofs	nofs
chr20	B	20q13.2	.	52375000	2284	nofs	nofs
chr20	B	20q13.2	.	52625000	2463	nofs	nofs
chr20	B	20q13.2	.	52875000	1957	nofs	nofs
chr20	B	20q13.2	.	53125000	3239	nofs	nofs
chr20	B	20q13.2	.	53375000	2653	nofs	nofs
chr20	B	20q13.2	.	53625000	4621	nofs	nofs
chr20	B	20q13.3	.	53875000	4312	nofs	nofs
chr20	B	20q13.3	.	54125000	2352	nofs	nofs
chr20	B	20q13.3	.	54375000	4183	nofs	nofs
chr20	B	20q13.3	.	54625000	6678	nofs	nofs
chr20	B	20q13.3	.	54875000	5513	nofs	nofs
chr20	B	20q13.3	.	55125000	7738	nofs	nofs
chr20	B	20q13.3	.	55375000	3347	nofs	nofs
chr20	B	20q13.3	.	55625000	5016	nofs	nofs
chr20	B	20q13.3	.	55875000	6820	nofs	nofs
chr20	B	20q13.3	.	56125000	3507	nofs	nofs
chr20	B	20q13.3	.	56375000	4466	nofs	nofs
chr20	B	20q13.3	.	56625000	6748	nofs	nofs
chr20	B	20q13.3	.	56875000	4037	nofs	nofs
chr20	B	20q13.3	.	57125000	2488	nofs	nofs
chr20	B	20q13.3	.	57375000	7372	nofs	nofs
chr20	B	20q13.3	.	57625000	6927	nofs	nofs
chr20	B	20q13.3	.	57875000	3210	nofs	nofs
chr20	B	20q13.3	.	58125000	3735	nofs	nofs
chr20	B	20q13.3	.	58375000	4082	nofs	nofs

chr20	B	20q13.3	.	58625000	4554	nofs	nofs
chr20	B	20q13.3	.	58875000	7349	nofs	nofs
chr20	T	20q13.3	.	59125000	9671	nofs	nofs
chr20	T	20q13.3	.	59375000	17724	nofs	nofs
chr20	T	20q13.3	.	59625000	15209	nofs	nofs
chr20	T	20q13.3	.	59875000	27410	nofs	nofs
chr20	T	20q13.3	.	60125000	8991	nofs	nofs
chr20	T	20q13.3	.	60375000	14269	nofs	nofs
chr20	T	20q13.3	.	60625000	9281	nofs	nofs
chr20	T	20q13.3	.	60875000	12282	nofs	nofs
chr20	T	20q13.3	.	61125000	15318	nofs	nofs
chr20	T	20q13.3	.	61375000	18268	nofs	nofs
chr20	T	20q13.3	.	61625000	23969	nofs	nofs
chr20	T	20q13.3	.	61875000	18615	nofs	nofs
chr20	T	20q13.3	.	62125000	28141	nofs	nofs
chr21	C	21p13	.	125000	0	nofs	nofs
chr21	C	21p13	.	375000	0	nofs	nofs
chr21	C	21p13	.	625000	0	nofs	nofs
chr21	C	21p13	.	875000	0	nofs	nofs
chr21	C	21p13	.	1125000	0	nofs	nofs
chr21	C	21p13	.	1375000	0	nofs	nofs
chr21	C	21p13	.	1625000	0	nofs	nofs
chr21	C	21p13	.	1875000	0	nofs	nofs
chr21	C	21p13	.	2125000	0	nofs	nofs
chr21	C	21p12	.	2375000	0	nofs	nofs
chr21	C	21p12	.	2625000	0	nofs	nofs
chr21	C	21p12	.	2875000	0	nofs	nofs
chr21	C	21p12	.	3125000	0	nofs	nofs
chr21	C	21p12	.	3375000	0	nofs	nofs
chr21	C	21p12	.	3625000	0	nofs	nofs
chr21	C	21p12	.	3875000	0	nofs	nofs
chr21	C	21p12	.	4125000	0	nofs	nofs
chr21	C	21p12	.	4375000	0	nofs	nofs
chr21	C	21p12	.	4625000	0	nofs	nofs
chr21	C	21p12	.	4875000	0	nofs	nofs
chr21	C	21p12	.	5125000	0	nofs	nofs

chr21	C	21p12	.	5375000	0	nofs	nofs
chr21	C	21p12	.	5625000	0	nofs	nofs
chr21	C	21p12	.	5875000	0	nofs	nofs
chr21	C	21p12	.	6125000	0	nofs	nofs
chr21	C	21p12	.	6375000	0	nofs	nofs
chr21	C	21p11.2	.	6625000	0	nofs	nofs
chr21	C	21p11.2	.	6875000	0	nofs	nofs
chr21	C	21p11.2	.	7125000	0	nofs	nofs
chr21	C	21p11.2	.	7375000	0	nofs	nofs
chr21	C	21p11.2	.	7625000	0	nofs	nofs
chr21	C	21p11.2	.	7875000	0	nofs	nofs
chr21	C	21p11.2	.	8125000	0	nofs	nofs
chr21	C	21p11.2	.	8375000	0	nofs	nofs
chr21	C	21p11.2	.	8625000	0	nofs	nofs
chr21	C	21p11.2	.	8875000	0	nofs	nofs
chr21	C	21p11.2	.	9125000	0	nofs	nofs
chr21	C	21p11.2	.	9375000	0	nofs	nofs
chr21	C	21p11.1	.	9625000	28164	nofs	nofs
chr21	C	21p11.1	.	9875000	121269	nofs	nofs
chr21	C	21p11.1	.	10125000	12008	nofs	nofs
chr21	C		.	10375000	0	nofs	nofs
chr21	C		.	10625000	0	nofs	nofs
chr21	C		.	10875000	0	nofs	nofs
chr21	C		.	11125000	0	nofs	nofs
chr21	C		.	11375000	0	nofs	nofs
chr21	C		.	11625000	0	nofs	nofs
chr21	C		.	11875000	0	nofs	nofs
chr21	C		.	12125000	0	nofs	nofs
chr21	C	21q11.1	.	12375000	0	nofs	nofs
chr21	C	21q11.1	.	12625000	0	nofs	nofs
chr21	C	21q11.1	.	12875000	0	nofs	nofs
chr21	C	21q11.1	.	13125000	0	nofs	nofs
chr21	C	21q11.2	1	13375000	44824	nofs	nofs
chr21	C	21q11.2	1	13625000	6272	nofs	nofs
chr21	C	21q11.2	1	13875000	4187	nofs	nofs
chr21	C	21q11.2	1	14125000	3428	nofs	nofs

chr21	C	21q11.2	1	14375000	5677	nofs	nofs
chr21	C	21q11.2	1	14625000	2527	nofs	nofs
chr21	C	21q11.2	1	14875000	2608	nofs	nofs
chr21	C	21q11.2	1	15125000	3031	nofs	nofs
chr21	C	21q11.2	1	15375000	2813	nofs	nofs
chr21	C	21q21	.	15625000	2526	nofs	nofs
chr21	C	21q21	.	15875000	2761	nofs	nofs
chr21	C	21q21	.	16125000	3421	nofs	nofs
chr21	C	21q21	.	16375000	3865	nofs	nofs
chr21	B	21q21	.	16625000	4184	nofs	nofs
chr21	B	21q21	.	16875000	2243	nofs	nofs
chr21	B	21q21	.	17125000	2201	nofs	nofs
chr21	B	21q21	.	17375000	4537	nofs	nofs
chr21	B	21q21	.	17625000	3511	nofs	nofs
chr21	B	21q21	.	17875000	2921	nofs	nofs
chr21	B	21q21	.	18125000	3036	nofs	nofs
chr21	B	21q21	.	18375000	2384	nofs	nofs
chr21	B	21q21	.	18625000	4859	nofs	nofs
chr21	B	21q21	.	18875000	2949	nofs	nofs
chr21	B	21q21	.	19125000	1875	nofs	nofs
chr21	B	21q21	.	19375000	1837	nofs	nofs
chr21	B	21q21	.	19625000	3017	nofs	nofs
chr21	B	21q21	.	19875000	2586	nofs	nofs
chr21	B	21q21	.	20125000	1910	nofs	nofs
chr21	B	21q21	.	20375000	2363	nofs	nofs
chr21	B	21q21	.	20625000	4848	nofs	nofs
chr21	B	21q21	.	20875000	3206	nofs	nofs
chr21	B	21q21	.	21125000	2986	nofs	nofs
chr21	B	21q21	.	21375000	2299	nofs	nofs
chr21	B	21q21	.	21625000	3658	nofs	nofs
chr21	B	21q21	.	21875000	2477	nofs	nofs
chr21	B	21q21	.	22125000	3336	nofs	nofs
chr21	B	21q21	.	22375000	1763	nofs	nofs
chr21	B	21q21	.	22625000	3885	nofs	nofs
chr21	B	21q21	.	22875000	3036	nofs	nofs
chr21	B	21q21	.	23125000	2625	nofs	nofs

chr21	B	21q21	.	23375000	3541	nofs	nofs
chr21	B	21q21	.	23625000	4945	nofs	nofs
chr21	B	21q21	.	23875000	2842	nofs	nofs
chr21	B	21q21	.	24125000	4752	nofs	nofs
chr21	B	21q21	.	24375000	2351	nofs	nofs
chr21	B	21q21	.	24625000	3288	nofs	nofs
chr21	B	21q21	.	24875000	2734	nofs	nofs
chr21	B	21q21	.	25125000	1946	nofs	nofs
chr21	B	21q21	.	25375000	2459	nofs	nofs
chr21	B	21q21	.	25625000	1817	nofs	nofs
chr21	B	21q21	.	25875000	2916	nofs	nofs
chr21	B	21q21	.	26125000	1540	nofs	nofs
chr21	B	21q21	.	26375000	3079	nofs	nofs
chr21	B	21q21	.	26625000	2203	nofs	nofs
chr21	B	21q21	.	26875000	1963	nofs	nofs
chr21	B	21q21	.	27125000	2700	nofs	nofs
chr21	B	21q21	.	27375000	2863	nofs	nofs
chr21	B	21q21	.	27625000	2181	nofs	nofs
chr21	B	21q21	.	27875000	3721	nofs	nofs
chr21	B	21q21	.	28125000	2518	nofs	nofs
chr21	B	21q21	.	28375000	2703	nofs	nofs
chr21	B	21q21	.	28625000	2727	nofs	nofs
chr21	B	21q21	.	28875000	3492	nofs	nofs
chr21	B	21q21	.	29125000	4458	nofs	nofs
chr21	B	21q21	.	29375000	4584	nofs	nofs
chr21	B	21q21	.	29625000	3319	nofs	nofs
chr21	B	21q21	.	29875000	3350	nofs	nofs
chr21	B	21q21	.	30125000	2951	nofs	nofs
chr21	B	21q21	.	30375000	2586	nofs	nofs
chr21	E	21q22.1	1	30625000	3367	nofs	nofs
chr21	E	21q22.1	1	30875000	2707	nofs	nofs
chr21	E	21q22.1	1	31125000	4322	nofs	nofs
chr21	E	21q22.1	1	31375000	3851	nofs	nofs
chr21	E	21q22.1	1	31625000	2885	nofs	nofs
chr21	E	21q22.1	1	31875000	5674	nofs	nofs
chr21	E	21q22.1	1	32125000	6523	nofs	nofs

chr21	E	21q22.1	1	32375000	3464	nofs	nofs
chr21	E	21q22.1	1	32625000	3681	nofs	nofs
chr21	E	21q22.1	1	32875000	6531	nofs	nofs
chr21	E	21q22.1	1	33125000	3509	nofs	nofs
chr21	E	21q22.1	1	33375000	4461	nofs	nofs
chr21	E	21q22.1	1	33625000	5753	nofs	nofs
chr21	E	21q22.1	1	33875000	4830	nofs	nofs
chr21	E	21q22.1	1	34125000	2085	nofs	nofs
chr21	E	21q22.1	1	34375000	3831	nofs	nofs
chr21	E	21q22.1	1	34625000	3259	nofs	nofs
chr21	E	21q22.1	1	34875000	3125	nofs	nofs
chr21	E	21q22.1	1	35125000	3850	nofs	nofs
chr21	E	21q22.1	1	35375000	2649	nofs	nofs
chr21	E	21q22.1	1	35625000	2334	nofs	nofs
chr21	E	21q22.1	1	35875000	3111	nofs	nofs
chr21	E	21q22.1	1	36125000	3676	nofs	nofs
chr21	E	21q22.1	1	36375000	7418	nofs	nofs
chr21	E	21q22.1	1	36625000	5016	nofs	nofs
chr21	E	21q22.1	1	36875000	4882	nofs	nofs
chr21	E	21q22.1	1	37125000	2214	nofs	nofs
chr21	E	21q22.1	1	37375000	2896	nofs	nofs
chr21	E	21q22.1	1	37625000	3103	nofs	nofs
chr21	E	21q22.1	1	37875000	3152	nofs	nofs
chr21	E	21q22.1	1	38125000	3582	nofs	nofs
chr21	E	21q22.1	1	38375000	4841	nofs	nofs
chr21	E	21q22.1	1	38625000	3595	nofs	nofs
chr21	B	21q22.2	.	38875000	4142	nofs	nofs
chr21	B	21q22.2	.	39125000	3998	nofs	nofs
chr21	B	21q22.2	.	39375000	4366	nofs	nofs
chr21	B	21q22.2	.	39625000	3196	nofs	nofs
chr21	B	21q22.2	.	39875000	4419	nofs	nofs
chr21	B	21q22.2	.	40125000	4220	nofs	nofs
chr21	B	21q22.2	.	40375000	6245	nofs	nofs
chr21	B	21q22.2	.	40625000	5110	nofs	nofs
chr21	B	21q22.2	.	40875000	5495	nofs	nofs
chr21	B	21q22.2	.	41125000	2916	nofs	nofs

chr21	E	21q22.3	3	41375000	6332	nofs	nofs
chr21	E	21q22.3	3	41625000	3792	nofs	nofs
chr21	E	21q22.3	3	41875000	7939	nofs	nofs
chr21	E	21q22.3	3	42125000	9677	nofs	nofs
chr21	E	21q22.3	3	42375000	10029	nofs	nofs
chr21	E	21q22.3	3	42625000	12293	nofs	nofs
chr21	E	21q22.3	3	42875000	14012	nofs	nofs
chr21	E	21q22.3	3	43125000	10700	nofs	nofs
chr21	E	21q22.3	3	43375000	7693	nofs	nofs
chr21	T	21q22.3	3	43625000	15150	nofs	nofs
chr21	T	21q22.3	3	43875000	9048	nofs	nofs
chr21	T	21q22.3	3	44125000	10892	nofs	nofs
chr21	T	21q22.3	3	44375000	11252	nofs	nofs
chr21	T	21q22.3	3	44625000	19035	nofs	nofs
chr21	T	21q22.3	3	44875000	11716	nofs	nofs
chr21	T	21q22.3	3	45125000	14711	nofs	nofs
chr21	T	21q22.3	3	45375000	8819	nofs	nofs
chr21	T	21q22.3	3	45625000	20893	nofs	nofs
chr21	T	21q22.3	3	45875000	19191	nofs	nofs
chr21	T	21q22.3	3	46125000	21652	nofs	nofs
chr21	T	21q22.3	3	46375000	23524	nofs	nofs
chr21	T	21q22.3	3	46625000	15774	nofs	nofs
chr22	C	22p13	.	125000	0	nofs	nofs
chr22	C	22p13	.	375000	0	nofs	nofs
chr22	C	22p13	.	625000	0	nofs	nofs
chr22	C	22p13	.	875000	0	nofs	nofs
chr22	C	22p13	.	1125000	0	nofs	nofs
chr22	C	22p13	.	1375000	0	nofs	nofs
chr22	C	22p13	.	1625000	0	nofs	nofs
chr22	C	22p13	.	1875000	0	nofs	nofs
chr22	C	22p13	.	2125000	0	nofs	nofs
chr22	C	22p13	.	2375000	0	nofs	nofs
chr22	C	22p13	.	2625000	0	nofs	nofs
chr22	C	22p13	.	2875000	0	nofs	nofs
chr22	C	22p13	.	3125000	0	nofs	nofs
chr22	C	22p12	.	3375000	0	nofs	nofs

chr22	C	22p12	.	3625000	0	nofs	nofs
chr22	C	22p12	.	3875000	0	nofs	nofs
chr22	C	22p12	.	4125000	0	nofs	nofs
chr22	C	22p12	.	4375000	0	nofs	nofs
chr22	C	22p12	.	4625000	0	nofs	nofs
chr22	C	22p12	.	4875000	0	nofs	nofs
chr22	C	22p12	.	5125000	0	nofs	nofs
chr22	C	22p12	.	5375000	0	nofs	nofs
chr22	C	22p12	.	5625000	0	nofs	nofs
chr22	C	22p12	.	5875000	0	nofs	nofs
chr22	C	22p12	.	6125000	0	nofs	nofs
chr22	C	22p12	.	6375000	0	nofs	nofs
chr22	C	22p12	.	6625000	0	nofs	nofs
chr22	C	22p11	.	6875000	0	nofs	nofs
chr22	C	22p11	.	7125000	0	nofs	nofs
chr22	C	22p11	.	7375000	0	nofs	nofs
chr22	C	22p11	.	7625000	0	nofs	nofs
chr22	C	22p11	.	7875000	0	nofs	nofs
chr22	C	22p11	.	8125000	0	nofs	nofs
chr22	C	22p11	.	8375000	0	nofs	nofs
chr22	C	22p11	.	8625000	0	nofs	nofs
chr22	C	22p11	.	8875000	0	nofs	nofs
chr22	C	22p11	.	9125000	0	nofs	nofs
chr22	C	22p11	.	9375000	0	nofs	nofs
chr22	C	22p11	.	9625000	0	nofs	nofs
chr22	C	22p11	.	9875000	0	nofs	nofs
chr22	C	22p11	.	10125000	0	nofs	nofs
chr22	C	22p11	.	10375000	0	nofs	nofs
chr22	C	22p11	.	10625000	0	nofs	nofs
chr22	C	22p11	.	10875000	0	nofs	nofs
chr22	C	22p11	.	11125000	0	nofs	nofs
chr22	C	22p11	.	11375000	0	nofs	nofs
chr22	C	22p11	.	11625000	0	nofs	nofs
chr22	C		.	11875000	0	nofs	nofs
chr22	C		.	12125000	0	nofs	nofs
chr22	C		.	12375000	0	nofs	nofs

chr22	C		.	12625000	0	nofs	nofs
chr22	C		.	12875000	0	nofs	nofs
chr22	C		.	13125000	0	nofs	nofs
chr22	C		.	13375000	0	nofs	nofs
chr22	C		.	13625000	0	nofs	nofs
chr22	C		.	13875000	0	nofs	nofs
chr22	C		.	14125000	0	nofs	nofs
chr22	C	22q11.1	.	14375000	3623	nofs	nofs
chr22	C	22q11.1	.	14625000	3969	nofs	nofs
chr22	C	22q11.1	.	14875000	120937	nofs	nofs
chr22	C	22q11.1	.	15125000	18402	nofs	nofs
chr22	C	22q11.1	.	15375000	15362	nofs	nofs
chr22	C	22q11.1	.	15625000	2445	nofs	nofs
chr22	C	22q11.1	.	15875000	3486	nofs	nofs
chr22	C	22q11.1	.	16125000	7048	nofs	nofs
chr22	C	22q11.2	5	16375000	4249	nofs	nofs
chr22	C	22q11.2	5	16625000	6573	nofs	nofs
chr22	C	22q11.2	5	16875000	5186	nofs	nofs
chr22	C	22q11.2	5	17125000	15225	nofs	nofs
chr22	C	22q11.2	5	17375000	7133	nofs	nofs
chr22	E	22q11.2	5	17625000	3073	nofs	nofs
chr22	E	22q11.2	5	17875000	3901	nofs	nofs
chr22	E	22q11.2	5	18125000	5451	nofs	nofs
chr22	E	22q11.2	5	18375000	4999	nofs	nofs
chr22	E	22q11.2	5	18625000	10851	nofs	nofs
chr22	E	22q11.2	5	18875000	10665	nofs	nofs
chr22	E	22q11.2	5	19125000	17067	nofs	nofs
chr22	E	22q11.2	5	19375000	3635	nofs	nofs
chr22	E	22q11.2	5	19625000	4761	nofs	nofs
chr22	E	22q11.2	5	19875000	7713	nofs	nofs
chr22	E	22q11.2	5	20125000	8909	nofs	nofs
chr22	E	22q11.2	5	20375000	3349	nofs	nofs
chr22	E	22q11.2	5	20625000	3389	nofs	nofs
chr22	E	22q11.2	5	20875000	10413	nofs	nofs
chr22	E	22q11.2	5	21125000	3145	nofs	nofs
chr22	E	22q11.2	5	21375000	3268	nofs	nofs

chr22	E	22q11.2	5	21625000	2786	nofs	nofs
chr22	E	22q11.2	5	21875000	2290	nofs	nofs
chr22	E	22q11.2	5	22125000	9635	nofs	nofs
chr22	E	22q11.2	5	22375000	6117	nofs	nofs
chr22	E	22q11.2	5	22625000	6323	nofs	nofs
chr22	E	22q11.2	5	22875000	2779	nofs	nofs
chr22	E	22q11.2	5	23125000	3887	nofs	nofs
chr22	E	22q11.2	5	23375000	3535	nofs	nofs
chr22	E	22q11.2	5	23625000	4154	nofs	nofs
chr22	E	22q11.2	5	23875000	5654	nofs	nofs
chr22	E	22q11.2	5	24125000	5160	nofs	nofs
chr22	E	22q11.2	5	24375000	2017	nofs	nofs
chr22	E	22q12.1	3	24625000	3931	nofs	nofs
chr22	E	22q12.1	3	24875000	4671	nofs	nofs
chr22	E	22q12.1	3	25125000	5667	nofs	nofs
chr22	E	22q12.1	3	25375000	6746	nofs	nofs
chr22	E	22q12.1	3	25625000	4471	nofs	nofs
chr22	E	22q12.1	3	25875000	4958	nofs	nofs
chr22	E	22q12.1	3	26125000	3934	nofs	nofs
chr22	E	22q12.1	3	26375000	5790	nofs	nofs
chr22	E	22q12.1	3	26625000	2947	nofs	nofs
chr22	E	22q12.1	3	26875000	2058	nofs	nofs
chr22	E	22q12.1	3	27125000	3255	nofs	nofs
chr22	E	22q12.1	3	27375000	4047	nofs	nofs
chr22	E	22q12.1	3	27625000	5155	nofs	nofs
chr22	E	22q12.1	3	27875000	4652	nofs	nofs
chr22	E	22q12.2	5	28125000	6168	fs	fs common
chr22	E	22q12.2	5	28375000	4815	fs	fs common
chr22	E	22q12.2	5	28625000	3209	fs	fs common
chr22	E	22q12.2	5	28875000	3842	fs	fs common
chr22	E	22q12.2	5	29125000	4669	fs	fs common
chr22	E	22q12.2	5	29375000	4166	fs	fs common
chr22	E	22q12.2	5	29625000	3051	fs	fs common
chr22	E	22q12.2	5	29875000	5163	fs	fs common
chr22	E	22q12.2	5	30125000	7018	fs	fs common
chr22	E	22q12.2	5	30375000	8306	fs	fs common

chr22	E	22q12.2	5	30625000	5629	fs	fs common
chr22	E	22q12.3	7	30875000	4713	nofs	nofs
chr22	E	22q12.3	7	31125000	5097	nofs	nofs
chr22	E	22q12.3	7	31375000	5011	nofs	nofs
chr22	E	22q12.3	7	31625000	3272	nofs	nofs
chr22	E	22q12.3	7	31875000	3904	nofs	nofs
chr22	E	22q12.3	7	32125000	2722	nofs	nofs
chr22	E	22q12.3	7	32375000	2089	nofs	nofs
chr22	E	22q12.3	7	32625000	3003	nofs	nofs
chr22	E	22q12.3	7	32875000	3153	nofs	nofs
chr22	E	22q12.3	7	33125000	5432	nofs	nofs
chr22	E	22q12.3	7	33375000	4495	nofs	nofs
chr22	E	22q12.3	7	33625000	2030	nofs	nofs
chr22	E	22q12.3	7	33875000	4640	nofs	nofs
chr22	E	22q12.3	7	34125000	8237	nofs	nofs
chr22	E	22q12.3	7	34375000	9874	nofs	nofs
chr22	E	22q12.3	7	34625000	2327	nofs	nofs
chr22	E	22q12.3	7	34875000	3860	nofs	nofs
chr22	E	22q12.3	7	35125000	3744	nofs	nofs
chr22	E	22q12.3	7	35375000	6211	nofs	nofs
chr22	E	22q12.3	7	35625000	6255	nofs	nofs
chr22	E	22q13.1	2	35875000	6717	fs	fs rare
chr22	E	22q13.1	2	36125000	4349	fs	fs rare
chr22	E	22q13.1	2	36375000	8731	fs	fs rare
chr22	E	22q13.1	2	36625000	4664	fs	fs rare
chr22	E	22q13.1	2	36875000	4752	fs	fs rare
chr22	E	22q13.1	2	37125000	4196	fs	fs rare
chr22	E	22q13.1	2	37375000	5125	fs	fs rare
chr22	E	22q13.1	2	37625000	8134	fs	fs rare
chr22	E	22q13.1	2	37875000	4481	fs	fs rare
chr22	E	22q13.1	2	38125000	5102	fs	fs rare
chr22	E	22q13.1	2	38375000	8070	fs	fs rare
chr22	E	22q13.1	2	38625000	2938	fs	fs rare
chr22	E	22q13.1	2	38875000	2690	fs	fs rare
chr22	E	22q13.1	2	39125000	5996	fs	fs rare
chr22	E	22q13.1	2	39375000	6831	fs	fs rare

chr22	E	22q13.2	2	39625000	3664	fs	fs rare
chr22	E	22q13.2	2	39875000	4375	fs	fs rare
chr22	E	22q13.2	2	40125000	6511	fs	fs rare
chr22	E	22q13.2	2	40375000	5673	fs	fs rare
chr22	E	22q13.2	2	40625000	3560	fs	fs rare
chr22	E	22q13.2	2	40875000	2393	fs	fs rare
chr22	E	22q13.2	2	41125000	5326	fs	fs rare
chr22	E	22q13.2	2	41375000	8029	fs	fs rare
chr22	E	22q13.2	2	41625000	6217	fs	fs rare
chr22	E	22q13.2	2	41875000	6722	fs	fs rare
chr22	E	22q13.2	2	42125000	8540	fs	fs rare
chr22	E	22q13.2	2	42375000	5201	fs	fs rare
chr22	E	22q13.2	2	42625000	6437	fs	fs rare
chr22	E	22q13.3	1	42875000	11583	fs	fs rare
chr22	E	22q13.3	1	43125000	5103	fs	fs rare
chr22	E	22q13.3	1	43375000	12627	fs	fs rare
chr22	E	22q13.3	1	43625000	8268	fs	fs rare
chr22	E	22q13.3	1	43875000	6220	fs	fs rare
chr22	E	22q13.3	1	44125000	3328	fs	fs rare
chr22	E	22q13.3	1	44375000	3676	fs	fs rare
chr22	E	22q13.3	1	44625000	6793	fs	fs rare
chr22	E	22q13.3	1	44875000	4415	fs	fs rare
chr22	E	22q13.3	1	45125000	5353	fs	fs rare
chr22	E	22q13.3	1	45375000	15678	fs	fs rare
chr22	E	22q13.3	1	45625000	8815	fs	fs rare
chr22	E	22q13.3	1	45875000	13582	fs	fs rare
chr22	E	22q13.3	1	46125000	8125	fs	fs rare
chr22	T	22q13.3	1	46375000	7691	fs	fs rare
chr22	T	22q13.3	1	46625000	5526	fs	fs rare
chr22	T	22q13.3	1	46875000	11778	fs	fs rare
chr22	T	22q13.3	1	47125000	13849	fs	fs rare
chr22	T	22q13.3	1	47375000	16541	fs	fs rare
chr22	T	22q13.3	1	47625000	18950	fs	fs rare
chr22	T	22q13.3	1	47875000	16235	fs	fs rare
chr22	T	22q13.3	1	48125000	19595	fs	fs rare
chr22	T	22q13.3	1	48375000	21563	fs	fs rare

chr22	T	22q13.3	1	48625000	10076	fs	fs rare
chr22	T	22q13.3	1	48875000	19055	fs	fs rare
chr22	T	22q13.3	1	49125000	14172	fs	fs rare
chr22	T	22q13.3	1	49375000	12929	fs	fs rare
